# The Protein Architecture of Human Secretory Vesicles Reveals Differential Regulation of Signaling Molecule Secretion by Protein Kinases

**DOI:** 10.1371/journal.pone.0041134

**Published:** 2012-08-16

**Authors:** Steven J. Bark, Jill Wegrzyn, Laurent Taupenot, Michael Ziegler, Daniel T. O'Connor, Qi Ma, Michael Smoot, Trey Ideker, Vivian Hook

**Affiliations:** 1 Skaggs School of Pharmacy and Pharmaceutical Sciences, University of California San Diego, La Jolla, California, United States of America; 2 Department of Medicine, University of California San Diego, La Jolla, California, United States of America; 3 Graduate Program in Bioinformatics and Systems Biology, University of California San Diego, La Jolla, California, United States of America; 4 Departments of Neurosciences and Pharmacology, University of California San Diego, La Jolla, California, United States of America; Yale School of Medicine, United States of America

## Abstract

Secretory vesicles are required for release of chemical messengers to mediate intercellular signaling among human biological systems. It is necessary to define the organization of the protein architecture of the ‘human’ dense core secretory vesicles (DCSV) to understand mechanisms for secretion of signaling molecules essential for cellular regulatory processes. This study, therefore, conducted extensive quantitative proteomics and systems biology analyses of human DCSV purified from human pheochromocytoma. Over 600 human DCSV proteins were identified with quantitative evaluation of over 300 proteins, revealing that most proteins participate in producing peptide hormones and neurotransmitters, enzymes, and the secretory machinery. Systems biology analyses provided a model of interacting DCSV proteins, generating hypotheses for differential intracellular protein kinases A and C signaling pathways. Activation of cellular PKA and PKC pathways resulted in differential secretion of neuropeptides, catecholamines, and β-amyloid of Alzheimer's disease for mediating cell-cell communication. This is the first study to define a model of the protein architecture of human DCSV for human disease and health.

## Introduction

The secretory vesicle organelle is essential for regulated release of chemical messengers that mediate cell-cell communication in the control of biological functions in human health and disease [1]–[3]. The large dense core secretory vesicle (DCSV) organelle is fundamental to neuroendocrine control of physiological functions of human systems through peptide neurotransmitter and hormone biosynthesis, storage, and their regulated secretion [4]–[7].

It is necessary to define the protein architecture of the human DCSV proteome which is required for the DCSV to conduct its exquisite secretory functions for cell-cell communication. Recent advances in mass spectrometry [8], [9] and systems biology [10] provide the basis of this study to define the proteome of human DCSV isolated from the human sympathoadrenal system. These studies were designed to answer two important questions. Firstly, what proteins comprise ‘human’ DCSV and what are their relative quantities? Secondly, what functional organization and protein networks exist among proteins comprising ‘human’ DCSV? Furthermore, definition of the human DCSV proteome data is a necessary resource to guide biological studies in model organisms to elucidate DCSV functions that are relevant to human physiological functions.

To address these questions, this study conducted an extensive quantitative proteomics and systems biology analysis of human dense core secretory vesicles isolated from human pheochromocytoma tissue of the sympathoadrenal system. The extensive proteomics data identified more than 600 proteins and statistical quantitation of 318 proteins. These data reveal the functional protein architecture of human DCSV, including those of soluble and membrane protein components of DCSV. Quantitation illustrated that the primary protein functions of DCSV are involved in biosynthesis of active peptides and catecholamines, regulation of internal DCSV conditions, and the secretory machinery. Organization of the protein architecture of DCSV proteins was assessed by the Cytoscape network visualization and analysis software for systems biology investigation [11]. These analyses of protein networks in DCSV suggested distinct protein kinase A (PKA) and protein kinase C (PKC) pathways for regulating DCSV secretory functions. Testing this hypothesis, cellular activation of PKA and PKC pathways in adrenal medullary chromaffin cells (bovine) in primary culture resulted in differential secretion of the neuropeptides enkephalin and galanin, catecholamine chemical messengers, and the beta-amyloid peptide known to participate in human Alzheimer's disease.

This represents the most comprehensive and detailed study of the human DCSV proteome performed- to-date, providing a model of the protein architecture utilized for DCSV function in human biology. Our data provide an extensive reference source for future analyses of human DCSV components, as well as those in model organisms, which are important for intercellular signaling in human disease.

## Results

### Strategy

The strategy of this study was to utilize human dense core secretory vesicles (DCSV) isolated from human pheochromocytoma as a model of human DCSV. The purified human DCSV was separated into soluble and membrane samples which were fractionated by SDS-PAGE (Fig. S1). Replicates of four lanes for each soluble and membrane sample obtained by SDS-PAGE were generated, and each gel lane was excised into 8 slices of molecular weight ranging from ∼200 kDa to ∼6 kDa. Each gel slice was subjected to tryptic digestion and subjected to nano-LC-MS/MS tandem mass spectrometry, for a total of 64 LC-MS/MS experimental runs which allowed quantitative analyses of proteomic data (sample processing illustrated in Fig. S1). Multiple bioinformatics steps were utilized for data processing of MS/MS data by Spectrum Mill analyses for identification of DCSV proteins, FDR (false discovery rate) analyses for defining quality of identifications, functional evaluation and organization (by GO, KEGG, IntAct, InterPro, SignalP, and TMHMM), NSAF (normalized spectral abundance factor) quantitation of proteins, and Cytoscape analyses to generate a model of protein interaction networks in human DCSV (bioinformatics pipeline is illustrated in Fig. S1). A hypothesis of the model was experimentally evaluated to assess the roles of protein kinases A and C (PKA and PKC, respectively) in regulating secretion of DCSV chemical messengers consisting of neuropeptides, catecholamines, and beta-amyloid. Results demonstrate that the protein architectural model of human DCSV can predict regulatory functions.

### Proteomics of Human Dense Core Secretory Vesicles (DCSV) Reveals Functional Protein Categories

Proteomic data of soluble and membrane fractions from human DCSV identified more than 600 proteins. All of the proteins meet the high level of confidence required for peptide identification, as described in Experimental Procedures S1. Protein identification data for the soluble fraction (listed in Table S1) and the membrane fraction (listed in Table S2) describe the name of the identified protein, accession number, HGNC symbol, peptide sequences identified by tandem mass spectrometry, values of scores and % SPI that meet the confidence levels for protein identification and related protein properties. The peptide identifications were used to define a minimally redundant set of proteins consistent with all of the identified peptides. Isoforms and related proteins were not included unless required by unique peptides, thus reducing the peptide identifications shared among annotated proteins.

Clustering analyses of proteins was conducted to organize proteomic data into functional categories of DCSV proteins (Table S3). These functional categories are composed of proteins for production of neurotransmitters and hormone factors, biochemical processes, regulation of internal conditions of DCSV, secretory mechanisms, morphological features, and other protein categories including cell growth and immune functions (Table 1). Sub-categories within these primary categories were deduced (Table 1). The neurotransmitter and hormone category includes proteins for neuropeptide and neurohumoural factors, enzymes and transporters for neurotransmitters, protease systems, and receptors. Biochemical systems include enzymes, as well as carbohydrate and lipid functions. DCSV functions, including biosynthesis of neuropeptide chemical messengers, require regulation of the internal DCSV environment, achieved by proteins for the regulation of reduction-oxidation, ATPases and nucleotides, and protein folding. Proteins that regulate secretion are key for DCSV function to release chemical messengers to the extracellular environment, for mediating cell-cell communication. Such proteins include those participating in signal transduction composed of GTP-binding proteins, vesicular trafficking and exocytosis, and calcium regulation. DCSV also contains proteins involved in cell adhesion, as well as structural proteins involved in DCSV processes.

**Table 1:** Number of Proteins Identified and Quantitated Among Functional Categories of Human Dense Core Secretory Vesicles (DCSV).

Functional Categories for DCSV	Soluble Proteins	Membrane Proteins
	#Identified (#Quantitated)	#Identified (#Quantitated)
**Neurotransmitters and Neurohumoural Factors**	61 (39)	57 (38)
Neuropeptides, Neurohumoural Factors	8 (8)	8 (8)
Neurotransmitter Enzymes/Transporters	10 (7)	13 (8)
Protease Systems	38 (24)	30 (21)
Receptors	5 (0)	6 (1)
**Biochemical Processes**	43 (29)	58 (36)
Enzymes	18 (10)	36 (19)
Carbohydrate Functions	17 (12)	12 (10)
Lipid Functions	8 (6)	10 (7)
**Internal Conditions of Secretory Vesicles**	38 (23)	124 (85)
Reduction-Oxidation	11 (8)	63 (48)
ATPases and Nucleotide Metabolism	15 (11)	40 (28)
Protein Folding	12 (4)	21 (9)
**Regulated Secretion Mechanisms**	67 (21)	99 (40)
Signal transduction and GTP-Binding Proteins	44 (12)	70 (28)
Vesicular Trafficking and Exocytosis	14 (3)	20 (5)
Calcium Regulation	9 (6)	9 (7)
**Morphological Functions of Secretory Vesicles**	54 (27)	83 (28)
Cell-Adhesion/Cell-Cell Interactions	10 (6)	26 (9)
Structural Proteins	44 (21)	57 (19)
**Other Protein Categories**	21 (12)	26 (16)
Cell Growth and Development	7 (4)	12 (9)
Immune	14 (8)	14 (7)
**Miscellaneous**	28 (17)	52 (21)
Miscellaneous	17 (11)	30 (15)
Unknown	11 (6)	22 (6)

The number of distinct proteins identified and quantitated (in parentheses) in each of the functional categories are indicated for soluble and membrane proteins of the human DCSV.

**Figure 1:**
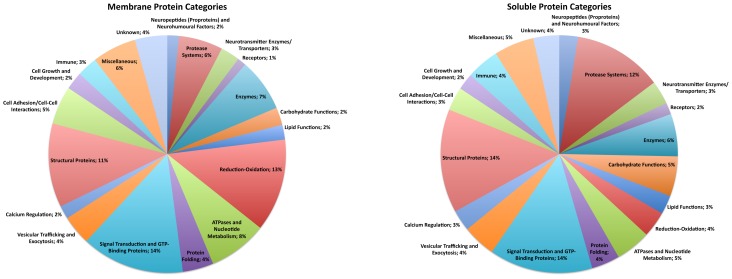
Functional categories of human DCSV soluble and membrane proteins. Pie charts illustrate the relative portion of proteins in each functional category for the soluble (panel A) and membrane (panel B) fractions of human DCSV. Each functional category, with name and percent of the total number of DCSV proteins, of the pie chart is shown as a distinct color. The proteins comprising each functional category are listed in Table S3, and proteomics identification of soluble and membrane proteins of human DCSV are listed in Tables S1 and S2.

Comparison of the distinct proteins identified in the soluble and membrane fractions of human DCSV illustrate similarities and differences in the numbers of proteins in each category (Table 1). A pie chart illustration of the different proteins in soluble and membrane components of DCSV (Figure 1) shows that all categories exist in both soluble and membrane fractions of DCSV. However, some differences in relative proportions of protein numbers in each category were observed (Table 1 and Figure 1).

### Quantitative Analyses of the DCSV Proteome Illustrate Relative Abundances of Protein Functions

Quantitation of proteomic data was achieved by normalized spectral abundance factor (NSAF) analyses [12] based on an isoform-specific algorithm generating a minimally redundant set of protein annotations explaining all of the peptide identifications. Quantitation of a protein required its identification in at least three of the four tandem mass spectrometry replicate experiments, and quantitation was achieved for 318 proteins (Table S4). Natural log transformation of total NSAF and NSAF values for soluble and membrane fraction protein constituents were performed to enable statistical evaluation of differences in protein distribution between these fractions. [12] (Table S4). The quantitated proteins in the soluble and membrane fractions were categorized by function (Table 1). Protein quantitation was achieved for all functional categories. These data illustrate a MS dynamic range of approximately 2–5×10^5^ for protein abundances in this organelle.

**Figure 2:**
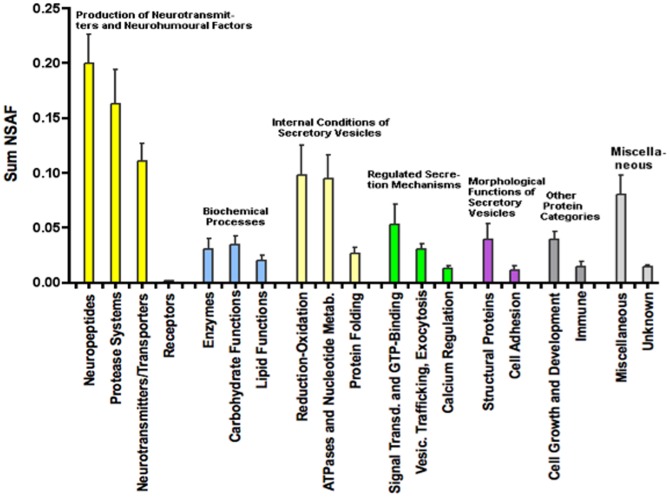
Quantification of protein categories of human dense core secretory vesicles. Relative quantification of proteins in the main functional categories were assessed by normalized spectral abundance factors (NSAF), as described in Experimental Procedures S1. Bar graphs illustrate average NSAF (sum) for each protein group with s.e.m. NSAF values for human DCSV proteins are provided in Table S4.

Comparison of protein abundances in the different categories revealed that those in the neurotansmitter and hormone category represented the most abundant proteins (Figure 2), consistent with the primary function of DCSV to synthesize, store, and secrete such chemical messengers. Proteins that regulate internal conditions of DCSV are also of notable abundance. Similar abundance levels were observed for proteins functioning in biochemical processing and secretory mechanisms, key functions for DCSV biological activities.

Within the neurotransmitter and hormone category, the relative abundances of prohormone processing pathway components utilized for production of peptide hormones and neurotransmitters (neuropeptides) were evaluated (Table 2). The prohormone chromogranin A (CgA) was most abundant compared to proenkephalin and pro-NPY prohormones. Among the prohormone processing enzymes, the prohormone convertase pathway components – prohormone convertases 1 and 2 (PC1/3 and PC2, respectively), and carboxypeptidase E (CPE) – show relative abundances for PC1/3/PC2/CPE of approximately 1/3/6. Following PC1/3, PC2, and CPE, the peptidylglycine-α-amidating monooxygenase enzyme is present for C-terminal amidation of neuropeptides [13], [14]. Endogenous regulators of PC1/3 and PC2 – proSAAS and 7B2, respectively – are present at ratios of PC1/proSAAS and PC2/7B2 of about 1/3.5 and 3/1. It is of interest that the cathepsin L prohormone processing enzyme [7], [15] is present at a level below that which can be detected in this study by MS/MS (Table 2). Protease gene knockout studies, combined with cellular expression, have demonstrated the prominent role of cathepsin L, with PC1/3 and PC2, for producing neuropeptides [7].

**Table 2:** Quantification of Prohormone Processing Components in Human Dense Core Secretory Vesicles (DCSV).

Protein Component	NSAF +/− S.D. (×10^3^)
**Prohormones**	
Chromogranin A	98.5+/−16.0
Chromogranin B	56.3+/−2.0
Proenkephalin	1.5+/−0.5
Pro-Neuropeptide Y	7.4+/−2.2
**Prohormone Processing Enzymes**	
Prohormone convertase 1 (PC1/3)	4.42+/−0.8
Prohormone convertase 2 (PC2)	13.8+/−1.5
ProSAAS (inhibitor of PC1/3)	15.7+/−3.9
7B2 (regulator of PC2)	4.3+/−0.9
Carboxypeptidase E (CPE)	26.7+/−3.2
Peptidylglycine α-amidating monooxygenase	3.3+/−0 1.5
Cathepsin L	<0.03
Aminopeptidase B (AP-B)	<0.03

The NASF values of the relative abundances for prohormone proteins and prohormone processing enzymes are listed as NASF ± S.D. (×10^3^). The prohormone processing enzymes represent two protease pathways consisting of (1) the subtilisin-like prohormone convertases (PC1/3 and PC2) with their endogenous regulators (proSAAS and 7B2 regulators, respectively), combined with carboxypeptidase E (CPE), and the (2) cysteine protease cathepsin L pathway with aminopeptidase B (AP-B).

Quantitation also indicated the high purity of the isolated human DCSV. Comparison of the relative quantities of the known chromogranin DCSV proteins with that of markers for mitochondria, lysosomes, and endoplasmic reticulum indicated the high purity of the human DCSV of ∼99% (Table S5). Based on the relative abundances of the DCSV proteins chromogranins A and B (Table S5), and knowledge that DCSV protein chromogranin A composes ∼46% of DCSV proteins [16], it is estimated that the lysosomal enzyme beta-glucuronidase shows a relative abundance of less than ∼0.3%, and the mitochondrial proteins citrate synthase and fumarate hydratase show relative abundances of less than 0.2% and 0.6%, respectively, of DCSV proteins. Thus, analyses of organelle marker proteins by quantitative mass spectrometry indicate the high purity of the purified human DCSV investigated in this study.

### Protein Interaction Networks in Human DCSV Modeled by Cytoscape

**Figure 3:**
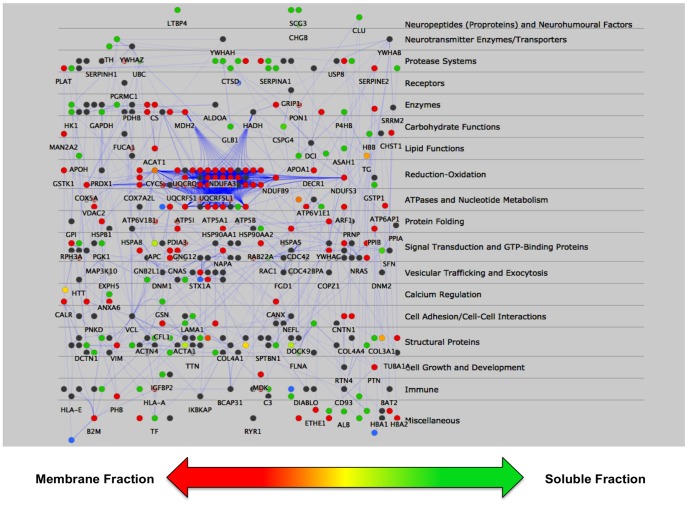
Cytoscape systems biology analyses of the human DCSV proteome. Components of the DCSV proteomics data were analyzed by the Cytoscape systems biology program for predicting protein interaction networks. The functional protein categories are illustrated on the right hand side. Based on quantitative NASF data of the proteins, individual proteins are indicated as predominantly soluble (green circles), predominantly membrane (red circles), or present in both soluble and membrane at similar levels (yellow) (Figure 3). These color-coded protein symbols are those which were quantitated by NSAF. Proteins illustrated by grey circles are those which were identified, but not quantitated since they did not meet the criteria for quantitation in at least 3 out of 4 nano-LC-MS/MS runs.

Protein network interactions among human DCSV proteins were evaluated using Cytoscape, a platform for complex network analysis and visualization [17], [18]. Construction of potential DCSV protein interaction networks was achieved by query of proteins identified by the mass spectrometry data to reported protein-protein interactions annotated in the Michigan Molecular Interaction (MiMI) Database [19], [20]. The complexity of the human DCSV interaction network is illustrated in Figure 3. Illustration of soluble and membrane protein interactions are organized according to functional categories using the Cerebral plugin for Cytoscape [21].

The human DCSV network map (Figure 3) represents a model of the protein architecture of this organelle. The model predicts extensive networks among the functional categories of proteins (outline on the right side of Figure 3). High-density networks are visualized for proteins involved in oxidation-regulation, namely, NADH dehydrogenase (NDUFA3) and ubiquinol-cytochrome c reductases (UQCRQ, UQCRFS1P1, and UQCRFS1). Proteins of the reduction-oxidation proteins interact with proteins involved in protein folding, ATPases that control proton pumps, signal transduction and GTP-binding proteins, and vesicular trafficking and exocytosis. These protein systems support the primary function of DCSV for storage and regulated secretion of chemical messengers known to include neuropeptides, catecholamines, and β-amyloid.

### Protein Network Model for DCSV Predicts Differential PKA and PKC Regulation of Neuropeptide, Catecholamine, and Beta-amyloid Secretion

**Figure 4:**
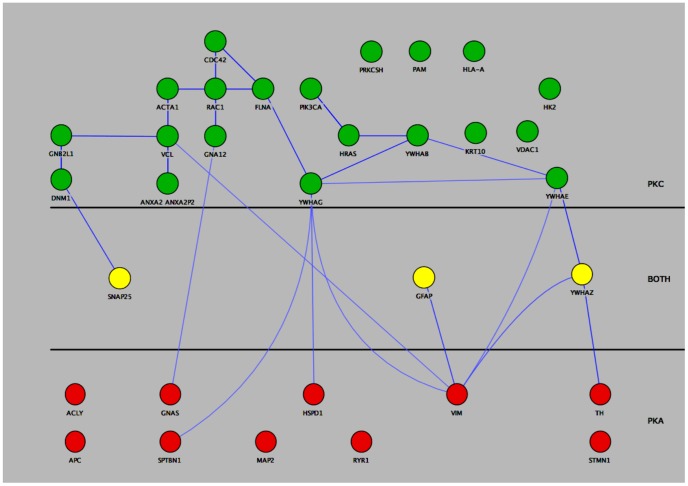
Systems biology analyses distinguishes protein kinase A (PKA) and protein kinase C (PKC) pathways in human DCSV. Human DCSV proteins interacting with PKA or PKC are illustrated in green or red circles, respectively. Proteins interacting with both PKA and PKC are shown in yellow.

Regulated secretion of chemical messengers involves intracellular signaling pathways, especially the protein kinase A (PKA) and protein kinase C (PKC) pathways [22]–[25]. Cytoscape mapping illustrates distinct PKA and PKC interacting networks within the DCSV proteome (Figure 4). A small subset of proteins interact with both PKA and PKC (Figure 4).

To evaluate the roles of PKA and PKC pathways in DCSV secretory functions, the effects of activating PKA or PKC on the stimulated secretion of neuropeptides, catecholamines, and β-amyloid were investigated. These experiments were conducted in neuronal-like chromaffin cells in primary culture from adrenal medulla of *Bos taurus* (bovine) as the model organism. The adrenal medulla is the tissue origin of the human pheochromocytoma from which the human DCSV were isolated.

**Figure 5:**
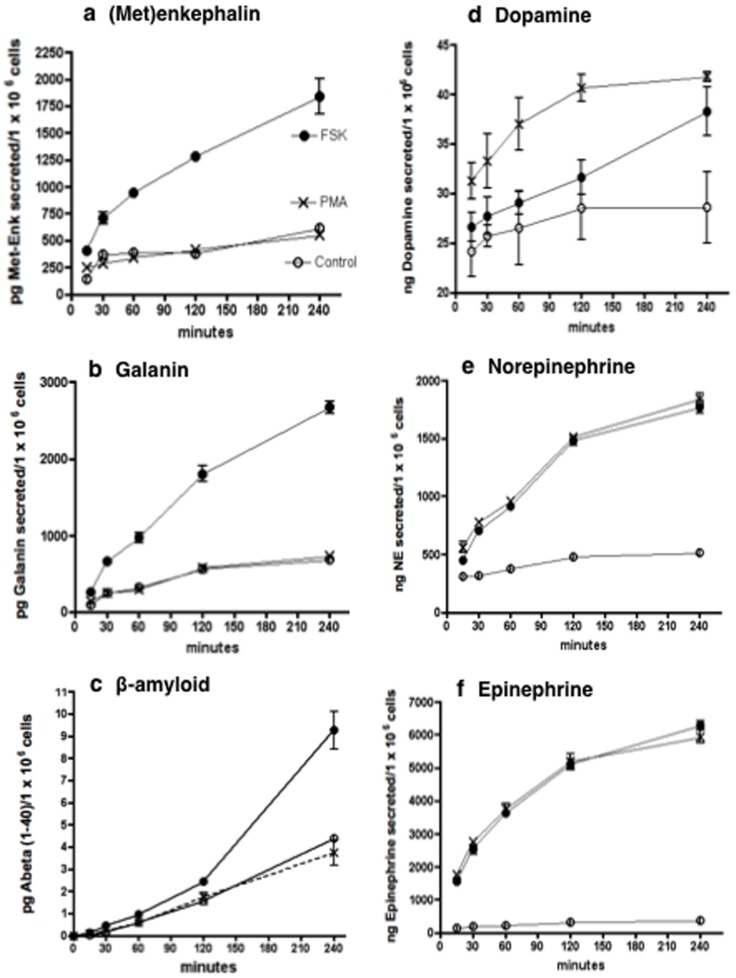
Differential regulation of neuropeptides, catecholamines, and β-amyloid secretion by activation of PKA and PKC in neuronal-like chromaffin cells. Adrenal medullary chromaffin cells in primary culture (bovine) were treated with forskolin that activates protein kinase A (PKA) that activates PKA via adenylyl cyclase stimulation and cAMP production, or with PMA (phorbol myristate acetate) that directly activates PKC. In time-course studies for treatment with forskolin or PMA for 15 minutes to 6 hours, the media was collected for measurement of secreted (Met)enkephalin and galanin neuropeptides (panels a and b, respectively), beta-amyloid peptide (Aβ(1–40), panel c), and the catecholamines dopamine, norepinephrine, and epinephrine (panels d, e, and f, respectively). Data for Control untreated cells (○), forskolin-treated cells (•), and PMA-treated cells (X) are plotted. Data for each time point represents the mean ± s.e.m. (n = 6); error bars are illustrated, and they are often smaller than the symbol.

Chromaffin cells were treated with forskolin, an activator of adenylyl cyclase [26] which produces cAMP which activates PKA, or with PMA (phorbol myristate acetate) that activates PKC [27]. Stimulation was performed in time-course studies that assessed effects on secretion of several chemical messengers known to be stored and secreted from DCSV. Secretion of the neuropeptides (Met)enkephalin and galanin were stimulated by forskolin, but PMA had no effect (Figure 5). Secretion of the neurotoxic beta-amyloid peptide was also stimulated by forskolin, but not by PMA (Figure 5). In contrast, the catecholamines showed different responses to PKA or PKC activation. Dopamine secretion was stimulated by forskolin, and stimulated secretion by PMA occurred to a lower extent (Figure 5). Norepinephrine and epinephrine secretion was dramatically stimulated by both foskolin and PMA (Figure 5). Thus, activation of PKA and PKC pathways result in stimulated secretion of catecholamines, but only activation of the PKA pathway results in increased secretion of two neuropeptides and beta-amyloid.

These data demonstrate that modeling of the human DCSV protein interaction network (Figure 4) can formulate testable hypotheses about regulatory networks involved in control of DCSV secretory functions. Moreover, experimental support for such hypotheses can be acquired from model organisms, such as the bovine chromaffin cell system utilized to assess different regulatory properties of PKA and PKC pathways for controlling secretion of diverse chemical messengers.

## Discussion

This report represents the most comprehensive study of the protein architecture of human dense core secretory vesicles (DCSV), which are critical for regulating neuroendocrine intercellular signaling in human health and disease. Protein interaction networks of human DCSV were defined by extensive quantitative proteomics of human DCSV isolated from human adrenal medullary pheochromocytoma and integrated with protein interactions in the public domain. Proteomics of purified human DCSV identified over 600 proteins with high confidence based on an isoform-specific analysis algorithm to define a minimally redundant set of proteins that explain all peptide mass spectrometry data. Quantitative NSAF analyses [12] provided relative quantitation of 318 proteins, revealing the most abundant proteins of DCSV for neuropeptide production, enzyme activities, regulation of internal DCSV conditions, and the secretory machinery. Cytoscape systems biology analyses predicted protein networks of DCSV, including distinct protein kinase A (PKA) and protein kinase C (PKC) signaling pathways in the DCSV. Experimental data showed that activation of PKA or PKC pathways resulted in differential regulation of neuropeptide, beta-amyloid, and catecholamine secretion from adrenal medullary chromaffin cells. Network mapping of human DCSV provides significant new mechanistic knowledge of the molecular relationships among DCSV proteomic components that are coordinated to control secretion of intercellular signaling molecules in human health and disease.

Quantitative proteomics indicated that DCSV proteins of greatest abundance participate in the biosynthesis of active peptide hormones and neurotransmitters (neuropeptides), which are derived from protein precursors that undergo proteolytic processing. Proteins that regulate internal conditions of DCSV represent a significant portion of DCSV components, which are involved in regulating reduction-oxidation, transport of protons by ATPases to achieve the acidic internal pH, and protein folding. DCSV functions of the regulated secretory machinery and biochemical processes, as well as morphological features, were indicated in these studies.

Relative quantitation of these DCSV proteins was achieved by NSAF analyses [12] directly and after natural log transformation statistical evaluation, yielding relative quantitation of 318 proteins. The use of this minimally-redundant set of protein annotations virtually eliminated peptides shared between identified proteins, thus reducing the necessity of algorithms such as distributive NSAF to account for these peptides in our quantitative data [28]. However, in the case of multiple isoforms of a particular protein, incorporation of distributive NSAF will be important and should be combined with our methods for generating a minimally redundant set of protein annotations.

Definition of the human secretory vesicle cellular systems in this study provides an extensive reference source for future analyses of human DCSV components in human disease and model organisms. The human adrenal pheochromocytoma investigated here provides a model for human DCSV secretory vesicle protein systems that are critical for endocrine and neuronal cell-cell communication mediated by neuropeptides, catecholamines, and related cell-cell messengers [29]–[40]. The value of these systems biology analyses of human DCSV protein components is through generation of hypotheses capable of evaluation in model organisms, as illustrated in this study demonstrating differential PKA and PKC regulation of neuropeptide, catecholamine, and β-amyloid secretion from model neuroendocrine chromaffin cells in primary culture from bovine adrenal medulla. Experimental validation of hypotheses generated from the human adrenal DCSV proteome via investigation of the model bovine adrenal chromaffin cells illustrates how the human proteomic information can successfully guide biological studies in model organisms.

This study addresses the first systems biology analysis of human secretory vesicle proteomics data set of human DCSV components. Several proteomics analyses have provided initial characterization of proteins in DCSV-type secretory vesicles of rat pancreatic secretory granules [32]–[34] and mouse corticotropes from a pituitary cell line [35]. The distinct neuronal synaptic vesicle organelle has undergone proteomics analyses from rat, revealing proteins for trafficking proteins neurotransmitter uptake [36]. These prior studies of regulated secretory vesicles are consistent with data reported in this manuscript. However, such prior studies have not applied systems biology analyses of proteomic data to assess protein regulatory networks.

The systems biology analyses of the human DCSV of this study provides hypotheses for experimental investigation. Secretory regulation by proteins identified in human DCSV can be assessed in model bovine adrenal medullary chromaffin cells that possess the parallel DCSV organelle studied here in human adrenal medullary pheochromocytoma. Proteomics analyses of the bovine DCSV [37] demonstrates its similarity to the human proteomics data of this study. Thus, the bovine chromaffin cell model containing DCSV represents a model of the human DCSV system. Indeed, evaluation in the chromaffin cells revealed that the systems biology prediction of differential protein kinase A (PKA) and protein kinase C (PKC) regulation of secretory molecules was demonstrated by results showing that PKA, but not PKC, stimulates the secretion of enkephalin, galanin, and beta-amyloid peptides in model bovine neuroendocrine adrenal medullary chromaffin cells. In contrast, secretion of the catecholamines norepinephrine, and epinephrine was stimulated by activation of both PKC and PKA, in such model cells. Secretion of dopamine was stimulated primarily by activation of PKC. These data provide support for the hypothesis that distinct PKA and PKC intracellular signaling pathways can selectively control the stimulated secretion of different profiles of intercellular signaling molecules.

The proteomes of several secretory organelles have also been studied in neutrophil secretory vesicles [38], brain clathrin-coated vesicles [39], as well as the liver secretory pathway of rough ER, smooth ER, and Golgi apparatus organelles [40]. Similar and differential protein components exist among these organelles functioning for cellular secretion. Comparison of these mammalian secretory proteomes, as well as from other organisms including yeast [41] through future systems biology analyses can indicate similarities and differences of predicted protein pathways that regulate secretion in diverse systems.

The key cargo of DCSV secretory vesicles is the small molecule intercellular signaling hormones and peptide neurotransmitters. The human DCSV proteome assessed in this study has been shown to contain known and newly identified peptides [42] that are produced, stored, and secreted by DCSV to regulate cell-cell communication. Global peptidomics investigation identified peptides derived from proenkephalin, pro-NPY, proSAAS, CgA, CgB, and SCG2 prohormones by proteolytic processing [42]. The peptidomics data complements the human DCSV proteomics and systems biology analyses conducted here, illustrating the protease pathway components combined with functional protein systems that participate in DCSV for neuropeptide biosynthesis. These data together demonstrate the complexity of the human DCSV proteome to generate small peptides secreted for intercellular signaling.

It should be noted that very low abundance proteins are not identified in these data, based on our biochemical and cellular analyses of DCSV proteins [37]. For example cathepsin L has been well-characterized to be a component of DCSV in neuroendocrine cells (bovine and mouse) demonstrated by purification from isolated bovine DCSV, immunoelectron microscopy of DCSV, and cellular immunofluorescence confocal microscopy [7]. Furthermore, the amyloid precursor protein (APP) is present in DCSV (bovine) which contains the secretases (beta-secretases composed of BACE1 and cathepsin B, and the gamma-secretase component presenilin) for generating neurotoxic beta-amyloid peptides from APP [43]–[46] but these components were not indicated in the proteomics data set of this study, suggesting their low abundance compared to other proteins. Further investigation of the DCSV proteome will reveal lower abundance proteins utilized for DCSV functions.

In conclusion, this extensive proteomics and systems biology investigation is the first to identify the complexity of the functional protein systems utilized in the human secretory vesicle system for production, storage, and regulated secretion of active peptide hormones and neurotransmitters, catecholamines, and beta-amyloid. The DCSV system is essential for human health and participates in human disease.

## Materials and Methods

### Preparation of Soluble and Membrane Proteins from Human Dense Core Secretory Vesicles (DCSV) of Human Pheochromocytoma Tissue

Collection of a human pheochromocytoma sample was conducted according to a protocol approved by the UCSD Human Research Protections Program (HRPP) which is the Institutional Review Board (IRB). This HRPP IRB approval for tissue procurement includes approval at the UCSD Medical Center and Veteran's Admistration hospitals in La Jolla, CA. This is a ‘no risk’ protocol for an existing tissue specimen obtained for other purposes (eg, surgical diagnostic pathology) for surgical specimens obtained initally for non-research use, if the specimen remains anonymous for further use. In this case, the IRB committee formally waives the necessity for written consent by the patient. This IRB approval is #091827X and dated 11/17/2011.

The human pheochromocytoma tissue from the sympathoadrenal system (from surgical specimen, with pathology report of benign tumor) was used for isolation of dense core secretory vesicles (DCSV), also known as chromaffin granules (CG), achieved by differential sucrose density gradient centrifugation as previously described [37], [47]–[50]. The isolated DCSV were lysed by freeze-thawing in 10 mM Tris-HCl, pH 7.5, containing 1∶200 dilution of a protease inhibitor cocktail (Calbiochem, San Diego, CA), and centrifuged at 100,000× g (SW60 rotor) at 4°C for 30 minutes. The resultant supernatant was collected as the soluble fraction, and the pellet was collected as the membrane fraction (and washed two times in the pH 6.0 buffer with protease inhibitors).

The density gradient isolation procedure has been established in the field to yield DCSV of high purity based on assessment of organelle markers and electron microscopy [37], [43], [47]–[54]. The procedure results in purified DCSV that lack biochemical markers for other subcellular organelles of lysosomes (acid phosphatase marker), cytoplasm (lactate dehydrogenase marker), mitochondria (fumarase and glutamate dehydrogenase markers), and endoplasmic reticulum (glucose-6-phosphatase marker). Enzyme markers in the purified DCSV preparation were found to represent 1% or less of total homogenate markers. Furthermore, electron microscopy has confirmed the purity and integrity of the isolated DCSV [43], [54].

### Reduction, Alkylation, and SDS-PAGE with Trypsin Digestion of DCSV proteins

Soluble and membrane samples (400–600 µg protein each) were precipitated using chloroform/methanol [55] and cysteines were reduced and alkylated by TCEP and iodoacetamide. The precipitated soluble protein sample was dissolved in 20 µl 20% acetonitrile, followed by addition of 5 µl of 100 mM TCEP (2-triscarboxyethylphosphine) in 20% acetonitrile (28.7 mg/ml), and incubated at 55°C for 15 min for reduction. Free cysteines were then alkylated by addition of 5 µl iodoacetamide (IAA, 100 mM in 20% acetonitrile, 18 mg/ml) and incubation in the dark for 20 min. The membrane protein sample was reduced and alkylated similarly, except that membrane proteins were placed in 60% acetonitrile (to dissolve proteins) and heated with TCEP at 55°C, followed by alkylation.

The proteins were separated by SDS-PAGE gel electrophoresis using Novex 12% Bis-Tris gels (Life Technologies Novex, Carlsbad, CA). Soluble and membrane samples were each loaded into 4 lanes (∼30–50 µg protein/lane) and subjected to SDS-PAGE. Proteins were stained with Coomassie Blue in 40% methanol/20% acetic acid for 30 minutes, and destained in 10% methanol. Gel lanes were excised into eight slices from ∼200 kDa to ∼6 kDa for in-gel trypsin digestion. All steps used Lo Bind (Eppendorf) low retention tubes [56]. In-gel trypsin digestion was conducted as described previously [57] (detailed description of trypsin digestion is provided in Experimental Procedures S1).

### Nano-LC-MS/MS Tandem Mass Spectrometry

Nano-LC-MS/MS tandem mass spectrometry analyses of soluble and membrane protein samples was conducted in quadruplicate runs on an Agilent XCT Ultra ion trap mass spectrometer coupled to an Agilent 1100 nano-HPLC system with the HPLC-ChipCube. The LC separation utilized an Agilent C18 HPLC chip (Agilent Zorbax C18 Chip, 150 mm×75 µm, 40 nl trap) with solvent A (water with 0.25% formic acid) and solvent B (acetonitrile with 0.25% formic acid). The LC gradient progressed from 3% B to 45% B in 40 minutes, followed by an increase to 95% B in 10 minutes. The mass spectrometer was set for data dependent scanning in MS/MS mode on the three most abundant ions present in the MS precursor ion scan. The exclusion time was set to 0.1 minute, isolation window set to 4 amu, and voltages set to −1850 V (capillary), −500 V (counter electrode) and 1.30 V (fragmentation). Smart ion target was set to 500,000 to correct for background ions. The maximum injection time was set to 100 ms. All other settings were retained as default.

Optimization of mass spectrometry instrument performance was achieved by analyses of standard trypsin digests of BSA (bovine serum albumin) and protein molecular weight standards (cytochrome C, carbonic anhydrase beta-amylase, alcohol dehydrogenase, and BSA, from Sigma-Aldrich, St. Louis, MO). Details of optimized conditions to obtain quality data are provided in Experimental Procedures S1.

### Database Search Parameters

Data searches of MS/MS spectra were processed using the Spectrum Mill database search platform (Agilent Technologies, version A.03.03.078). Database searches used 2.5 amu for precursor mass tolerance and 0.8 amu fragment mass tolerance. The search database was the curated Human RefSeq from NCBI. Protein identification thresholds for Score and %SPI were set according to analysis of a decoy database at ∼1% False Discovery Rate (explained in next two paragraph). Valid Spectrum Mill Scores were graded based on charge state of the peptides: Score ≥13 and %SPI ≥70% for +1 and +2 peptides, Score ≥16 and %SPI ≥70% for +3 peptides. The Score indicates the raw match between the observed spectrum and the theoretical fragmentation peaks from the “identified" peptide and the %SPI indicates the percent of spectral intensity in the observed spectrum that can be accounted for by the theoretical fragments.

These protein identification thresholds for Score and %SPI were set according to analysis of a decoy database [58] constructed by twice random shuffling the curated Human RefSeq using the Decoy.pl script (Matrix Science, LTD, 2006). Decoy searches were conducted for a cross section of 60,000 spectra and compared to those same spectra searched against the authentic Human Refseq. Non-tryptic peptide search criteria resulted in almost all high-scoring peptides being tryptic and high numbers of “random" identifications to the shuffled decoy database. Further tests with OMSSA [59] and X!Tandem [60], demonstrated comparable peptide identifications with Spectrum Mill. High score “random" decoy identifications were compared to the Human RefSeq database using BLAST with optimized parameters for short peptides, which confirmed 5–7 amino acid sequences that were homologous or identical to authentic peptide sequences in the Human RefSeq. We corrected for this homology by manually comparing if the spectra identifying the decoy peptide sequence was consistent with an authentic peptide in the Human RefSeq. Additionally, spectra identifying decoy sequences that also identified a high-scoring peptide in the target RefSeq database were considered. In both cases, we obtained the same cutoff scoring thresholds: Score ≥13 and %SPI ≥70% (+2 peptides) and score ≥16 and %SPI ≥70% (+3 peptides). Similar analysis demonstrated that two or more peptides adequately identify proteins with score ≥10 and %SPI ≥70%.

These methods for protein identifications were validated by 2 computational methods. First, absolute false-positive rates (FPR) were calculated using Mass Spectrometry Generating Function (MS-GF) for a selection of 50 of the lowest scoring peptides that passed the above empirical thresholds [61]. Second, we calculated the False Discovery Rate (FDR) for +2, +3 and total peptide identifications based on decoy database analysis described above [58]. (Figure S2) Peptides were binned by score (width = 1 score unit) and the number in each bin was mapped on a histogram for both decoy and target database identifications. Final FDR was calculated by the formula: FDR = Decoy Identifications/Real Identifications. The calculated FDRs were 1.6e-2 for +3 peptides, 2.65e-3 for +2 peptides, and 1.07e-2 for total peptide identifications.

### Protein Organization and Clustering

Batch Entrez (http://ncbi.nlm.nih.gov/entrez/batchentrez.cgi?db=Protein) was used to generate FASTA formatted protein sequence databases for each GenInfo Identifier (GI) number for proteins identified by the MS experiment. BLASTCLUST was used to perform pairwise comparisons followed by single-linkage clustering of the statistically significant matches (>95% homology over 90% of the sequence length) (http://www.ncbi.nlm.nih.gov/blast/). The protein list is thus the smallest minimally-redundant set of proteins explaining all peptide identifications in the data. Following this analysis, an annotated table of soluble and membrane proteins was compiled.

The functional categories of identified proteins were defined by the gene ontology resource (http://www.geneontology.org). Further information on the function of proteins was obtained through the KEGG and Interact pathway databases, as well as through the MEROPS database. A series of GO terms in each category was acquired though text searching of specific keywords relating to function and localization. In addition, both identified and unidentified protein sequences were queried against the InterPro (http://www.ebi.ac.uk/interpro/) database, SignalP resource (http://www.cbs.dtu.dk/services/SignalP/) and TMHMM resource (http://www.cbs.dtu.dk/services/TMHMM/) in order to define protein family. Analyses were enhanced with Pubmed searches to assess literature information on protein functions.

### Quantitation of Protein Abundances by NSAF

Protein abundances were derived from the Normalized Spectral Abundance Factor (NSAF) method [12]. The NSAF was calculated by the following equation: (NSAF)_K_ = (SpC/MW)_K_/S(SpC/MW). The spectral abundance factor for a protein (K) is the number of spectral counts (SpC) for protein K divided by the molecular weight of protein K. The NSAF is obtained by dividing the spectral abundance factor for protein K by the sum of all spectral abundance factors for all proteins (I) observed in the sample. The NSAF is corrected for differences in protein sizes (as defined by molecular weight) and normalized for the total number of data spectral counts. Quantitation was considered for proteins that satisfy strict identification criteria and were observed in a minimum of three of the four replicate experiments. When proteins were not observed in one replicate measurement, the standard deviations were calculated for the protein NSAF by including a zero for that failed observation. For analyses in Cytoscape, the NSAF was utilized directly as a measurement of abundance.

For statistical analysis and comparison of protein quantities, the NSAF data was transformed to natural log scale and subjected to normality and statistical tests as previously reported [12] Total NSAF, Membrane NSAF and Soluble NSAF measurements were transformed to natural log scale and Gaussian normality of each data set was confirmed by D'Agostino-Pearson and Shapiro-Wilks tests prior to application of Student's T-test methods for statistically evaluating confidence of differences. (StatPlus:Mac 2009, AnalystSoft) For analysis of normality of the soluble and membrane fraction data, significant numbers of non-measurements (found exclusively in either soluble or membrane fraction) skewed the normality of distribution. Therefore, these non-measurements were not included for normality tests.

These quantitative analyses allowed evaluation of the relative abundances of functional proteins in the human DCSV proteome, and provided analyses of organelle marker proteins [62], [63] to demonstrate the high purity of these isolated secretory vesicles.

### Cytoscape Mapping of Protein Interaction Networks in Human DCSV

Visualization of protein functional organization and interaction networks was accomplished using Cytoscape [17], [64]. Proteins identified in LC-MS/MS data were first mapped to gi and Uniprot accession numbers by an in house proteomics pipeline program suite. The output of these data was converted to the Microsoft Excel spreadsheet format to simplify further processing and analysis in Cytoscape. Approximately 15% of proteins identified could not be directly mapped to Uniprot while other proteins exhibited mapping to secondary or multiple Uniprot numbers. To resolve these protein identifications, the gi and Uniprot protein accessions were mapped to Entrez numbers using Synergizer (http://llama.med.harvard.edu/synergizer/translate), then mapped back to Uniprot to provide the primary Uniprot identifiers and improved accession identification for unmapped proteins. Further manual curation and resolution of duplicate Uniprot identifiers enabled assignment for 99% of the 1050 non-redundant set of identified proteins (with approximately 10 proteins remaining unassigned). Identifiers include Uniprot numbers, Entrezgene numbers, and HGNC primary gene symbols. HGNC gene symbols for all proteins identified in these experiments were imported and searched using the MiMI plugin (Michigan Molecular Interaction Database, Version 3.01) within Cytoscape to construct protein-protein interaction networks [17], [19], [20], [65]. The Excel spreadsheet combining all proteomics data for these proteins was then imported into Cytoscape as an attribute file, which enabled each protein in the network to reference its corresponding protein identification and quantitative data. Subnetworks were derived from this “global" data network and include observed functional protein categories Networks were visually organized using Spring Embedded or Unweighted Force Directed Layouts (available natively in Cytoscape) or using the Cerebral plugin [21], [66]. Quantitative information was incorporated into Cytoscape using a NSAF difference map as follows: NSAF-Soluble – NSAF-Membrane = Diff-NSAF. The composite Diff-NSAF was multiplied by 10^3^ and represents the membrane or soluble distribution of the measured proteins. Protein distribution was color-coded with membrane proteins colored red (negative Diff-NSAF) and soluble proteins colored green (positive Diff-NSAF). Proteins equally distributed are yellow color-coded.

### Activation of Protein Kinase A and C in Neuronal-like Chromaffin Cells and Effects on Secretion of Neuropeptides, Catecholamines, and β-Amyloid

Differential effects of activating protein kinases A and C, predicted by Cytoscape analyses of protein interaction networks in the human DCSV, was experimentally assessed in primary cultures of model bovine adrenal medullary chromaffin cells for effects on secretion of several DCSV chemical messenger consisting of neuropeptides, catecholamines (dopamine, norepinephrine, and epinephrine), and β-amyloid. Primary cultures of chromaffin cells were prepared from fresh bovine adrenal medulla as we have previously described [25], [43]. After 5–7 days in culture, cells were treated with forskolin (50 µM) to active protein kinase A (PKA) by stimulating adenylyl cyclase formation of cAMP that activates PKA, or with PMA (100 nM, phorbol myristate acetate) which activates protein kinase C (PKC). Cells were incubated with forskolin or PMA in time course studies of 15, 30, and 60 minutes, and also at 2 and 4 hours. Culture media was collected at each time point. Secretion of DCSV chemical messengers monitored the amounts of secreted (Met)enkephalin and galanin neuropeptides measured by radioimmunoassay (RIA), catecholamines (dopamine, norepinephrine, and epinephrine), and β-amyloid(1–40), as we have previously described [25], [43]. Results are expressed as the mean ± s.e.m. of chemical messenger per volume of media, with n = 6 replicates. Statistical significance was calculated (student's t-test, p<0.05).

## Supporting Information

Experimental Procedures S1 (PDF)

Figure S1 DCSV processing for proteomics and bioinformatics. Human dense core secretory vesicles (DCSV) were purified from human adrenal medullary pheochromocytoma, and soluble and membrane fractions were separated. DCSV were subjected to fractionation by 1-D SDS-PAGE (panel a), with soluble and membrane DCSV samples each run in quadruplicate gel lanes, and 8 slices were excised from each gel lane for in-gel trypsin digestion followed by nano-LC-MS/MS tandem mass spectrometry (panel b). Mass spectrometry data was subjected to bioinformatics analyses to identify peptides and proteins, assess functional organization of proteomics data, obtain NSAF quantification, and assess predicted protein interaction networks (panel c), as described in Experimental Procedures S1.
(PDF)

Figure S2 Peptide identifications from mass spectrometry data analysed for false discovery rates (FDR). Peptide target database and decoy database identification histograms for +2 and +3 peptides are illustrated (panels ‘a’ and ‘b’, respectively). Peptides identified by the Spectrum Mill database search algorithm were segregated by charge state and organized by score into bins of one score unit width (X-axis). The number of peptides within each bin were counted and are represented by the bar height (Y-axis). This was done for peptides identified in database search against the Human RefSeq database (blue bars, true positives) and against a decoy database derived from the Human RefSeq by two stages of randomization (black bars, false positives). The False Discovery Rate (FDR) was calculated by the ratio of false positives over true positives at thresholds: Score ≥13 and SPI ≥70% for +2 peptides and Score ≥16 and SPI ≥70% for +3 peptides. The resulting FDRs are 0.27% for +2 peptides and 1.6% for +3 peptides. The total FDR for all identified peptides is 1.07%.
(PDF)

Table S1 Soluble proteins identified in human dense core secretory vesicles. Addendum: MS/MS spectra of single peptide identifications for soluble DCSV proteins.
(PDF)

Table S2 Membrane proteins identified in human dense core secretory vesicles. Addendum: MS/MS spectra of single peptide identifications for membrane DCSV proteins.
(PDF)

Table S3 Functional organization of proteins in human dense core secretory vesicles.
(PDF)

Table S4 Relative quantitation of human DCSV proteins by normalized spectral abundance factor (NSAF).
(PDF)

Table S5 Quantitation of organelle markers for mitochondria, lysosomes, and endoplasmic reticulum (ER) reveals the high purity of the human dense core secretory vesicles.
(PDF)

## References

[1] Lodish H., Berk A, Matsudaira P, Kaiser CA, Krieger M, et al.. (2004) Molecular Cell Biology. Fifth edition, New York: W. H. Freeman and Company. 701–742.

[2] Siegel GJ, Agranoff BS, Albers RW, Fisher SK, Uhler MD (1999) Basic Neurochemistry. 6^th^ edition, Philadelphia: Lippincott-Raven. 191–400.

[3] Gainer H, Russell JT, Loh YP (1985) The enzymology and intracelular organization of peptide precursor processing: the secretory vesicle hypothesis. Neuroendocrinology 40: 171–184. 3883214 10.1159/000124070

[4] Burgess TL, Kelly RB (1987) Constitutive and regulated secretion of proteins. Annu Rev Cell Biol 3: 243–293. 3318877 10.1146/annurev.cb.03.110187.001331

[5] Kim T, Gondre-Lewis MC, Arnaoutova I, Loh YP (2006) Dense-core secretory granule biogenesis. Physiology 21: 124–133. 16565478 10.1152/physiol.00043.2005

[6] Dikeakos JD, Reudelhuber TL (2007) Sending proteins to dense core secretory granules: still a lot to sort out. J Cell Biol 277: 191–196. 10.1083/jcb.200701024 PMC2064127 17438078

[7] Hook V, Funkelstein L, Lu D, Bark S, Wegrzyn J, et al (2008) Proteases for processing proneuropeptides into peptide neurotransmitters and hormones. Annu Rev Pharmacol Toxicol 48: 393–423. 18184105 10.1146/annurev.pharmtox.48.113006.094812 PMC2731677

[8] Suizdak G (2003) The expanding role of mass spectrometry in biotechnology. San Diego: MCC Press.

[9] Gross JH (2004) Mass spectrometry. Berlin: Springer.

[10] Chuang HY, Hofree M, Ideker T (2010) A decade of systems biology. Annu Rev Cell Devel Biol 26: 721–744. 20604711 10.1146/annurev-cellbio-100109-104122 PMC3371392

[11] Cline MS, Smoot M, Cerami E, Kuchinsky A, Landys N, et al (2007) Integration of biological networks and gene expression data using Cytoscape. Nat Protoc 2: 2366–82. 17947979 10.1038/nprot.2007.324 PMC3685583

[12] Zybailov B, Mosley AL, Sardiu ME, Coleman MK, Florens L, et al (2006) Statistical analysis of membrane proteome expression changes in Saccharomyces cerevisiae. J Proteome Res 5: 2339–2347. 16944946 10.1021/pr060161n

[13] Eipper BA, Stoffers DA, Mains RE (1992) The biosynthesis of neuropeptides: peptide alpha-amidation. Annu Rev Neurosci 15: 57–85. 1575450 10.1146/annurev.ne.15.030192.000421

[14] Yin P, Bousquet-Moore D, Annangudi SP, Southey BR, Mains RE, et al (2011) Probing the production of amidated peptides following genetic and dietary copper manipulations. PLoS One 6: e28679. 22194882 10.1371/journal.pone.0028679 PMC3241674

[15] Yasothornsrikul S, Greenbaum D, Medzihradszky KF, Toneff T, Bundey R, et al (2003) Cathepsin L in secretory vesicles functions as a prohormone-processing enzyme for production of the enkephalin peptide neurotransmitter. Proc Natl Acad Sci U S A 100: 9590–9595. 12869695 10.1073/pnas.1531542100 PMC170962

[16] O'Connor DT, Frigon RP (1984) Chromogranin A, the major catecholamine storage vesicle soluble protein. J Biol Chem 259: 3237–3247. 6421820

[17] Shannon P, Markiel A, Ozier O, Baliga NS, Wang JT, et al (2003) Cytoscape: a software environment for integrated models of biomolecular interaction networks. Genome Res 13: 2498–2504. 14597658 10.1101/gr.1239303 PMC403769

[18] Smoot ME, Ono K, Ruscheinski J, Wang PL, Ideker T (2011) Cytoscape 2.8: new features for data integration and network visualization. Bioinformatics 27: 431–432. 21149340 10.1093/bioinformatics/btq675 PMC3031041

[19] Jayapandian M, Chapman A, Tarcea VG, Yu C, Elkiss A, et al (2007) Michigan Molecular Interactions (MiMI): putting the jigsaw puzzle together. Nucleic Acids Res 35: D566–571. 17130145 10.1093/nar/gkl859 PMC1716720

[20] Gao J, Ade AS, Tarcea VG, Weymouth TE, Mirel BR, et al (2009) Integrating and annotating the interactome using the MiMI plugin for cytoscape. Bioinformatics 25: 137–138. 18812364 10.1093/bioinformatics/btn501 PMC2638934

[21] Barsky A, Gardy JL, Hancock RE, Munzner T (2007) Cerebral: a Cytoscape plugin for layout of and interaction with biological networks using subcellular localization annotation. Bioinformatics 23: 1040–1042. 17309895 10.1093/bioinformatics/btm057

[22] Gorelick F, Pandol S, Thrower E (2008) Protein kinase C in the pancreatic acinar cell. J Gastroenterol Hepatol Suppl 1: S37–41. 10.1111/j.1440-1746.2007.05282.x 18336661

[23] Seino S, Shibasaki T (2005) PKA-dependent and PKA-independent pathways for cAMP-regulated exocytosis. Physiol Rev 85: 1303–1342. 16183914 10.1152/physrev.00001.2005

[24] Takahashi M, Itakura M, Kataoka M (2003) New aspects of neurotransmitter release and exocytosis: regulation of neurotransmitter release by phosphorylation. J Pharmacol Sci 93: 41–45. 14501150 10.1254/jphs.93.41

[25] Hook V, Toneff T, Baylon S, Sei C (2008) Differential activation of enkephalin, galanin, somatostatin, NPY, and VIP neuropeptide production by stimulators of protein kinases A and C in neuroendocrine chromaffin cells. Neuropeptides 42: 503–511. 18619673 10.1016/j.npep.2008.05.001 PMC2745396

[26] Seamon KB, Daly JW, Metzger H, de Souza NJ, Reden J (1983) Structure-activity relationships for activation of adenylate cyclase by the diterpene forskolin and its derivatives. J Med Chem 26: 436–439. 6681845 10.1021/jm00357a021

[27] Goel G, Makkar HP, Francis G, Becker K (2007) Phorbol esters: structure, biological activity, and toxicity in animals. Int J Toxicol 26: 279–288. 17661218 10.1080/10915810701464641

[28] Zhang Y, Wen Z, Washburn MP, Florens L (2010) Refinements to label free proteome quantitation: how to deal witih peptides shared by muiltiple proeins. Anal. Chem 82: 2272–2281. 10.1021/ac9023999 20166708

[29] Kelly RB (1991) Secretory granule and synaptic vesicle formation. Curr Opin Cell Biol 3: 654–660. 1663373 10.1016/0955-0674(91)90037-y

[30] Chidgey MA (1993) Protein targeting to dense-core secretory granules. Bioessays 15: 317–321. 8343142 10.1002/bies.950150505

[31] Winkler H, Fischer-Colbrie R (1998) Regulation of the biosynthesis of large dense-core vesicles in chromaffin cells and neurons. Cell Mol Neurobiol 18: 193–209. 9535290 10.1023/A:1022516919932 PMC11560186

[32] Chen X, Walker AK, Strahler JR, Simon ES, Tomanicek-Volk SL, et al (2006) Organellar proteomics: analysis of pancreatic zymogen granule membranes. Mol Cell Proteomics 5: 306–312. 16278343 10.1074/mcp.M500172-MCP200

[33] Brunner Y, Couté Y, Iezzi M, Foti M, Fukuda M, et al (2007) Proteomics analysis of insulin secretory granules. Mol Cell Proteomics 6: 1007–1017. 17317658 10.1074/mcp.M600443-MCP200

[34] Rindler MJ, Xu CF, Gumper I, Smith NN, Neubert TA (2007) Proteomic analysis of pancreatic zymogen granules: identification of new granule proteins. J Proteome Res 6: 2978–2992. 17583932 10.1021/pr0607029 PMC2582026

[35] Gauthier DJ, Sobota JA, Ferraro F, Mains RE, Lazure C (2008) Flow cytometry-assisted purification and proteomic analysis of the corticotropes dense-core secretory granules. Proteomics 8: 3848–3861. 18704904 10.1002/pmic.200700969 PMC2989539

[36] Takamori S, Holt M, Stenius K, Lemke EA, Grønborg M, et al (2006) Molecular anatomy of a trafficking organelle. Cell 127: 831–846. 17110340 10.1016/j.cell.2006.10.030

[37] Wegrzyn JL, Bark SJ, Funkelstein L, Mosier CA, Yap A, et al (2010) Proteomics of dense core secretory vesicles reveal distinct protein categories for secretion of neuroeffectors for cell-cell communication. J Proteome Res 9: 5002–5024. 20695487 10.1021/pr1003104 PMC2996463

[38] Jethwaney D, Islam MR, Leidal KG, de Bernabe DB, Campbell KP, et al (2007) Proteomic analysis of plasma membrane and secretory vesicles from human neutrophils. Proteome Sci 5: 12. 17692124 10.1186/1477-5956-5-12 PMC2075486

[39] Blondeau F, Ritter B, Allaire PD, Wasiak S, Girard M, et al (2004) Tandem MS analysis of brain clathrin-coated vesicles reveals their critical involvement in synaptic vesicle recycling. Proc Natl Acad Sci USA 101: 3833–3838. 15007177 10.1073/pnas.0308186101 PMC374330

[40] Gilchrist A, Au CE, Hiding J, Bell AW, Fernandez-Rodriguez J, et al (2006) Quantitative proteomics analysis of the secretory pathway. Cell 127: 1265–1281. 17174899 10.1016/j.cell.2006.10.036

[41] Bonifacino JS, Glick BS (2004) The mechanisms of vesicle budding and fusion. Cell 116: 153–166. 14744428 10.1016/s0092-8674(03)01079-1

[42] Gupta N, Bark SJ, Lu WD, Taupenot L, O'Connor DT, et al (2010) Mass spectrometry-based neuropeptidomics of secretory vesicles from human adrenal medullary pheochromocytoma reveals novel peptide products of prohormone processing. J Proteome Res 9: 5065–5075. 20704348 10.1021/pr100358b PMC3000314

[43] Hook VYH, Toneff T, Aaron W, Yasothornsrikul S, Bundey R, et al (2002) β-amyloid peptide in regulated secretory vesicles of chromaffin cells: evidence for multiple cysteine proteolytic activities in distinct pathways for β-secretase activity in chromaffin vesicles. J Neurochem 81: 237–256. 12064471 10.1046/j.1471-4159.2002.00794.x

[44] Hook V, Toneff T, Bogyo M, Greenbaum D, Medzihradszky KF, et al (2005) Inhibition of cathepsin B reduces β-amyloid production in regulated secretory vesicles of neuronal chromaffin cells: evidence for cathepsin B as a candidate β-secretase of Alzheimer's disease. Biol Chem 386: 931–940. 16164418 10.1515/BC.2005.108

[45] Hook V, Kindy M, Hook G (2008) Inhibitors of cathepsin B improve memory and reduce β-Amyloid in transgenic Alzheimer's disease mice expressing the wild type, but not the Swedish mutant, β-secretase site of the amyloid precursor protein. J Biol Chem 283: 7745–7753. 18184658 10.1074/jbc.M708362200

[46] Efthimiopoulos S, Floor E, Georgakopoulos A, Shioi J, Cui W, et al (1998) Enrichment of presenilin 1 peptides in neuronal large dense-core and somatodendritic clathrin-coated vesicles. J Neurochem 71: 2365–2372. 9832134 10.1046/j.1471-4159.1998.71062365.x

[47] Smith AD, Winkler H (1967) A simple method for the isolation of adrenal chromaffin granules on a large scale. Biochem J 103: 480–482. 6032982 10.1042/bj1030480 PMC1270431

[48] Hook VH, Eiden LE (1984) Two peptidases that convert 125I-Lys-Arg-(Met)enkephalin and 125I (Met)enkephalin-Arg6, respectively, to 125I-(Met)enkephalin in bovine adrenal medullary chromaffin granules. FEBS Lett 172: 212–218. 6378657 10.1016/0014-5793(84)81128-x

[49] O'Connor DT, Frigon RP, Deftos LJ (1983) Immunoreactive calcitonin in catecholamine storage vesicles of human pheochromocytoma. J Clin Endocrinol Metab 56: 582–585. 6822655 10.1210/jcem-56-3-582

[50] Parmer RJ, Mahata M, Mahata S, Sebald MT, O'Connor DT, et al (1997) Tissue plasminogen activator (t-PA) is targeted to the regulated secretory 7. pathway. Catecholamine storage vesicles as a reservoir for the rapid release of t-PA. J Biol Chem 272: 1976–82. 8999889 10.1074/jbc.272.3.1976

[51] Gratzl M, Krieger-Brauer H, Ekerdt R (1981) Latent acetylcholinesterase in secretory vesicles isolated from adrenal medulla. Biochim Biophys Acta 649: 355–366. 7317405 10.1016/0005-2736(81)90425-9

[52] Parmer RJ, O'Connor DT (1988) Enkephalins in human pheochromocytomas: localization in immunoreactive, high molecular weight form to the soluble core of chromaffin granules. J Hypertens 6: 187–98. 3361117 10.1097/00004872-198803000-00002

[53] Yasothornsrikul S, Toneff T, Hwang SR, Hook VYH (1998) Arginine and lysine aminopeptidase activities in chromaffin granules of bovine adrenal medulla: relevance to prohormone processing. J Neurochem 70: 153–163. 9422358 10.1046/j.1471-4159.1998.70010153.x

[54] Wegrzyn J, Lee J, Neveu JM, Lane WS, Hook V (2007) Proteomics of neuroendocrine secretory vesicles reveal distinct functional systems for biosynthesis and exocytosis of peptide hormones and neurotransmitters. J Proteome Research 6: 1652–1665. 17408250 10.1021/pr060503p

[55] Wessel D, Flügge UI (1984) A method for the quantitative recovery of protein in dilute solution in the presence of detergents and lipids. Anal Biochem 138: 141–143. 6731838 10.1016/0003-2697(84)90782-6

[56] Bark SJ, Hook V (2007) Differential recovery of peptides from sample tubes and the reproducibility of quantitative proteomic data. J Proteome Research 6: 4511–4516. 17850064 10.1021/pr070294o

[57] Bark SJ, Lu WD, Hook V (2009) Linear and accurate quantitation of proenkephalin-derived peptides by isotopic labeling with internal standards and mass spectrometry. Anal Biochem 389: 18–26. 19289094 10.1016/j.ab.2009.03.010 PMC2731681

[58] Elias JE, Haas W, Faherty BK, Gygi SP (2005) Comparative evaluation of mass spectrometry platforms used in large-scale proteomics investigations. Nat Methods 2: 667–675. 16118637 10.1038/nmeth785

[59] Geer LY, Markey SP, Kowalak JA, Wagner L, Xu M, et al Open mass spectrometry search algorithm. J Proteome Res 3: 958–964. 15473683 10.1021/pr0499491

[60] Craig R, Bevis RC (2004) TANDEM: matching proteins with tandem mass spectra. Bioinformatics 20: 1466–1467. 14976030 10.1093/bioinformatics/bth092

[61] Kim S, Gupta N, Pevzner PA (2008) Spectral probabilities and generating functions of tandem mass spectra: a strike against decoy databases. J Proteome Res 7: 3354–3363. 18597511 10.1021/pr8001244 PMC2689316

[62] Andreyev AY, Shen Z, Guan Z, Ryan A, Fahy E, et al (2010) Application of proteomic marker ensembles to subcellular organelle identification. Molecular and Cellular Proteomics 9: 388–402. 19884172 10.1074/mcp.M900432-MCP200 PMC2830848

[63] Scott MS, Calafell SJ, Thomas DY, Hallett MT (2005) Refining protein subcellular localization. PLoS Comput Biol 1: e66. 16322766 10.1371/journal.pcbi.0010066 PMC1289393

[64] Ideker T, Galitski T, Hood L (2001) A new approach to decoding life: systems biology. Annu. Rev. Genomics Hum Gen 2: 343–372. 10.1146/annurev.genom.2.1.343 11701654

[65] Tarcea VG, Weymouth T, Ade A, Bookvich A, Gao J, et al (2009) Michigan molecular interactions r2: from interacting proteins to pathways. Nucleic Acids Res 37: D642–646. 18978014 10.1093/nar/gkn722 PMC2686565

[66] Suderman M, Hallett M (2007) Tools for visually exploring biological networks. Bioinformatics 23: 2651–2659. 17720984 10.1093/bioinformatics/btm401

