# Supplementary material for: The Protein Architecture of Human Secretory Vesicles Reveals Differential Regulation of Signaling Molecule Secretion by Protein Kinases
Source: PLoS One. 2012 Aug 16;7(8):e41134. doi: 10.1371/journal.pone.0041134 (PMC3420874; doi:10.1371/journal.pone.0041134)
Supplement: Experimental Procedures S1 — (PDF) [file pone.0041134.s001.pdf]

Experimental Procedures S1

In-Gel Tryptic Digestion of DCSV Proteins.

After fractionation of soluble and membrane DCSV proteins by SDS-PAGE, gel slices (diced) were destained using 3 cycles of washing; each cycle consisted of washing in solution 1 (75% 25 mM ammonium bicarbonate/25% acetonitrile) at 55° C for 15 minutes, followed by solution 2 (50% 25 mM ammonium bicarbonate/50% acetonitrile) at 55° C for 15 minutes, and then gel pieces were dried at 30° C in a speedvac for 15 minutes. Trypsin digestion was conducted by adding 176 μl 25 mM ammonium bicarbonate, pH 7.0, 4 μl 0.1 $CaCl_2$, and 20 μl of trypsin stock (20ng/μl trypsin in 25 mM ammonium bicarbonate, pH 7.0, with 400 ng trypsin added (sequencing grade, Promega), followed by incubation at 37° C for 18 hours for digestion. Samples were extracted with 75% water/25% acetonitrile/1% formic acid and then with 50% water/50% acetonitrile/1% formic acid. Extracts and supernatants were combined, concentrated in a speedvac, and stored at -70° C in low binding eppendor tubes [56, 57] prior to analyses.

Optimization of Instrument and Chromatographic Performance.

Optimization of instrument performance was undertaken by analyzing tryptic digests of BSA and protein gel filtration molecular weight standards from Sigma-Aldrich (cytochrome C, carbonic anhydrase beta-amylase, alcohol dehydrogenase and bovine serum albumin). In a representative digest procedure, 25mg of BSA was dissolved in 16ml of 20% acetonitrile in doubly-distilled water. Reduction was performed with 100mM TCEP at 55˚C for 15 minutes followed by cooling to room temperature and alkylation with 100 mM iodoacetamide for 45 minutes in the dark. After alkylation, 126 ml of 25mM ammonium acetate with 1 mM calcium chloride and 50ml of trypsin stock (20 ng trypsin/ml) was added. Digestion was allowed to proceed at 37˚C for 12-18 hours. This procedure was also used for digestion of the protein mixture from gel filtration molecular weight standards. Instrument performance was graded based on quality of analysis of these digests. Often, multiple rounds of calibrations were required to achieve appropriate instrument sensitivity. While performance differs between instruments, the Agilent XCT Ultra ion trap mass spectrometer is capable of 30% sequence coverage and 3 orders of magnitude signal/noise for maximal signals from 30 femtomoles of

injected BSA digest. It is anticipated that other equivalent instruments should achieve this level of performance.

Chromatographic performance was evaluated using these same digested protein samples. Optimization of separation focused on maximizing separation of peptide components across the gradient while retaining sharp peak profiles. Typical elution peak-width-at-half-height measurements were approximately 10 seconds for peptide components. For peptide digests, a gradient of 3%-45% acetonitrile in 40 minutes followed by a rapid ramp to 95% acetonitrile was found to be optimal for a 150 mm x 75 mm Agilent Zorbax C-18 chip. While longer gradient times were tested, they provided only marginal improvement in performance in the Agilent HPLC ChipCube system for samples of the complexity utilized in these experiments. It is emphasized that the chromatographic and instrument systems are linked and both must be optimized for highest performance.
