# Supplementary figures and images for: The Protein Architecture of Human Secretory Vesicles Reveals Differential Regulation of Signaling Molecule Secretion by Protein Kinases
Source: PLoS One. 2012 Aug 16;7(8):e41134. doi: 10.1371/journal.pone.0041134 (PMC3420874; doi:10.1371/journal.pone.0041134)

Figure S1

(a) SDS-PAGE of DCSV

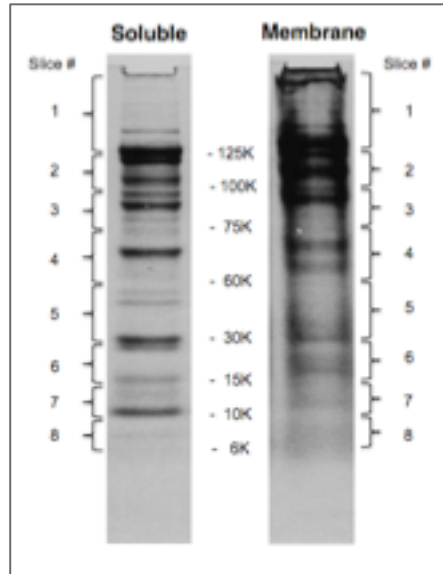

(b) Sample Processing for MS/MS

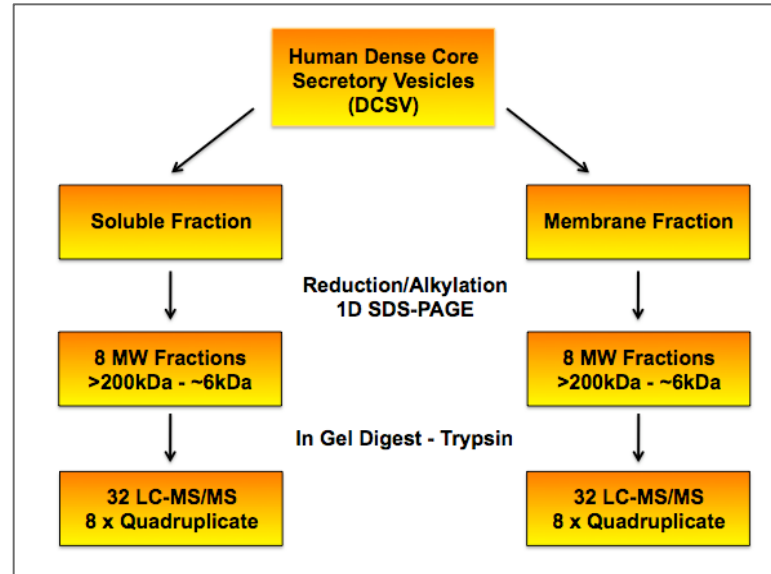

(c) Bioinformatics for Data Processing

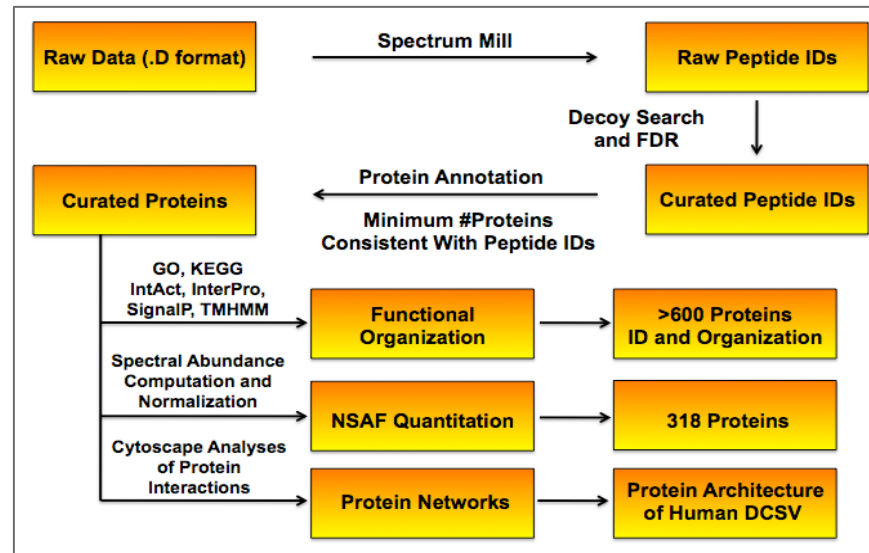

Supplement: Figure S1 — DCSV processing for proteomics and bioinformatics. Human dense core secretory vesicles (DCSV) were purified from human adrenal medullary pheochromocytoma, and soluble and membrane fractions were separated. DCSV were subjected to fractionation by 1-D SDS-PAGE (panel a), with soluble and membrane DCSV samples each run in quadruplicate gel lanes, and 8 slices were excised from each gel lane for in-gel trypsin digestion followed by nano-LC-MS/MS tandem mass spectrometry (panel b). Mass spectrometry data was subjected to bioinformatics analyses to identify peptides and proteins, assess functional organization of proteomics data, obtain NSAF quantification, and assess predicted protein interaction networks (panel c), as described in Experimental Procedures S1. (PDF) [file pone.0041134.s002.pdf]

Figure S2

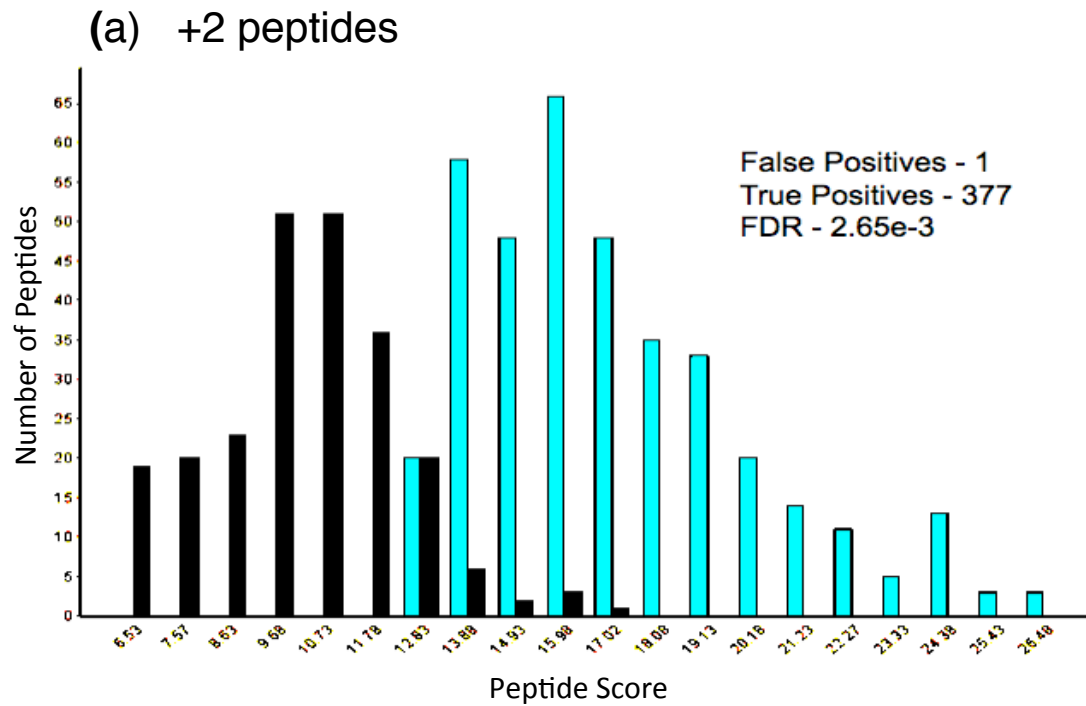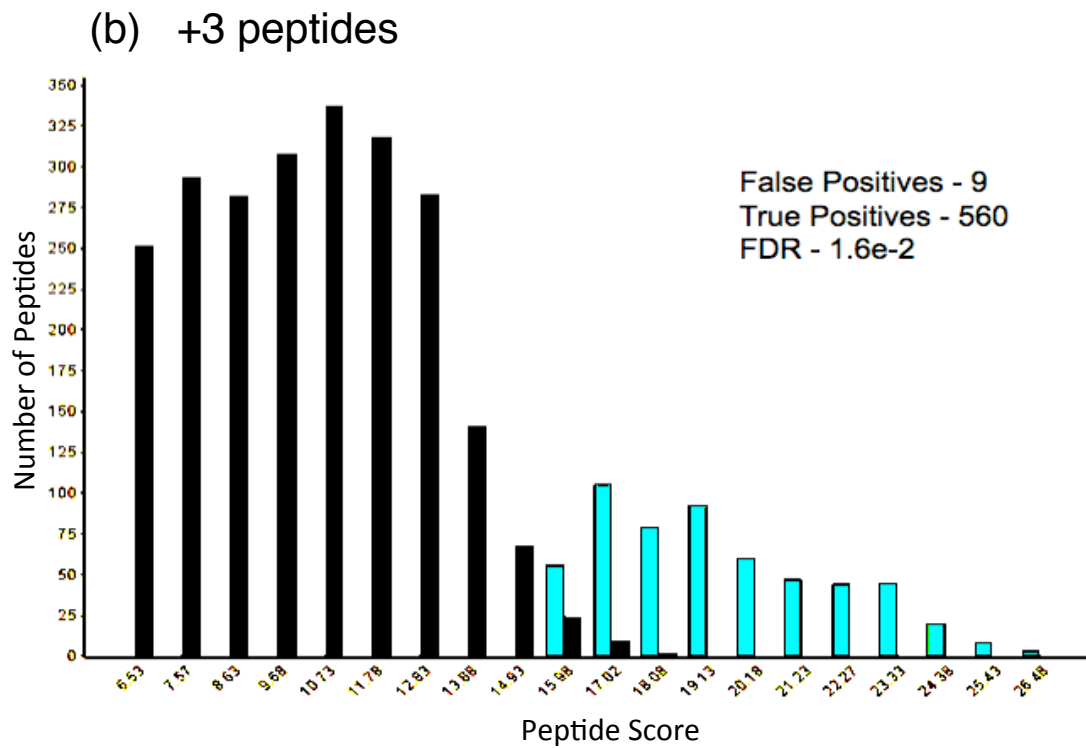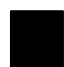

= Decoy Database Peptides

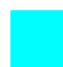

= Target Database Peptides

Supplement: Figure S2 — Peptide identifications from mass spectrometry data analysed for false discovery rates (FDR). Peptide target database and decoy database identification histograms for +2 and +3 peptides are illustrated (panels ‘a’ and ‘b’, respectively). Peptides identified by the Spectrum Mill database search algorithm were segregated by charge state and organized by score into bins of one score unit width (X-axis). The number of peptides within each bin were counted and are represented by the bar height (Y-axis). This was done for peptides identified in database search against the Human RefSeq database (blue bars, true positives) and against a decoy database derived from the Human RefSeq by two stages of randomization (black bars, false positives). The False Discovery Rate (FDR) was calculated by the ratio of false positives over true positives at thresholds: Score ≥13 and SPI ≥70% for +2 peptides and Score ≥16 and SPI ≥70% for +3 peptides. The resulting FDRs are 0.27% for +2 peptides and 1.6% for +3 peptides. The total FDR for all identified peptides is 1.07%. (PDF) [file pone.0041134.s003.pdf]
