# Supplementary material for: The Protein Architecture of Human Secretory Vesicles Reveals Differential Regulation of Signaling Molecule Secretion by Protein Kinases
Source: PLoS One. 2012 Aug 16;7(8):e41134. doi: 10.1371/journal.pone.0041134 (PMC3420874; doi:10.1371/journal.pone.0041134)

Table S1. Soluble Proteins Identified in Human Dense Core Secretory Vesicles (DCSV)

Protein Description	Accession no.	HGNC symbol	Peptide sequences identified	# of Tryptic Peptides	Protein MW	Protein pI	Peptide Score	Peptide % SPI	Peptide pI	Fragment Ions	Parent Charge
acid alpha-glucosidase preproprotein	119393891	GAA	AGYIIPLQGPGLTTTESR	8	105324.4	5.62	18.86	93.7	6.04	25	2
			DFPAMVQELHQGGR	8	105324.4	5.62	16.22	90.9	5.32	25	3
			NHNSLLSLPQEPYSFSEPAQQAMR	8	105324.4	5.62	20.08	90	5.4	25	3
			PLFLEFPK	8	105324.4	5.62	13.08	97.6	6.43	18	2
			QQPMALAVALTK	8	105324.4	5.62	16.06	84	8.75	25	2
			SRPGPRDAQAHPGRPRAVPT	8	105324.4	5.62	11.79	75.9	12	25	3
			WGYSSTAITR	8	105324.4	5.62	11.14	74.5	8.75	25	2
			YMMIVDPAISSSGPAGSYRPYDEGLR	8	105324.4	5.62	19.76	91.6	4.56	25	3
aconitase 2 precursor	4501867	ACO2	NAVTQEFGPVPDTAR	1	85425.9	7.36	12.58	74.8	4.37	25	2
actinin, alpha 1	4501891	ACTN1	AIMTYVSSFYHAFSGAQK	30	103058.1	5.25	17.25	87.1	8.55	25	3
			ALDFIASK	30	103058.1	5.25	15.62	94.2	5.88	25	2
			CQLEINFNTLQTK	30	103058.1	5.25	12.33	78.2	5.99	25	2
			DDPLTNLNTAFDVAEK	30	103058.1	5.25	19.19	92.8	3.84	25	2
			DHSGTLGPEEFK	30	103058.1	5.25	15.92	83.3	4.65	25	3
			DLLLDPAWEK	30	103058.1	5.25	15.93	96.6	4.03	18	2
			EGLLLWCQR	30	103058.1	5.25	12.12	84.7	6.09	25	2
			ELPPDQAEYCIAR	30	103058.1	5.25	12.99	90.9	4.14	25	2
			ETADTDTADQVMASFK	30	103058.1	5.25	16.04	84.8	3.84	25	2
			FAIQDISVEETSAK	30	103058.1	5.25	14.45	81.9	4.14	25	2
			GISQEQMNEFR	30	103058.1	5.25	15.04	86.1	4.53	25	2
			HRPELIDYGK	30	103058.1	5.25	14.25	81.2	6.75	25	3
			HTNYTMEHIR	30	103058.1	5.25	11.14	76.3	6.92	23	3
			ICDQWDNLGALTQK	30	103058.1	5.25	10.89	76.2	4.21	21	3
			LAILGIHNEVSK	30	103058.1	5.25	16.07	93.4	6.75	25	3
			LASDLLEWIR	30	103058.1	5.25	14.7	86	4.37	25	2
			LLETIDQLYLEYAK	30	103058.1	5.25	10.95	73.1	4.14	25	2
			LMLLLEVISGER	30	103058.1	5.25	12.29	71.5	4.53	25	2
			LSNRPAFMPSEGR	30	103058.1	5.25	19.56	87.7	9.6	25	3
			MLDAEDIVGTARPDEK	30	103058.1	5.25	13.45	83.1	4.1	25	3
			MVSDINNAWGCLEQVEK	30	103058.1	5.25	14.82	88.9	4.14	25	2
			QFGAQANVIGPWIQTK	30	103058.1	5.25	11.99	79.8	8.75	25	2
			QKDYETATLSEIK	30	103058.1	5.25	10.69	71.7	4.68	25	2
			TINEVENQILTR	30	103058.1	5.25	18.9	86.3	4.53	25	2
			TIPWLENR	30	103058.1	5.25	10.31	94.5	5.66	17	2
			VEQIAAIAQELNELDYYDSPSVNAR	30	103058.1	5.25	22.26	89.2	3.84	25	3
			VGWEQLLTTIAR	30	103058.1	5.25	17.13	88.3	5.97	25	2
			VIGPWIQTK	30	103058.1	5.25	12.62	81.7	8.72	25	2
			VLAVNQENEQLMEDYEK	30	103058.1	5.25	21.35	95.2	3.91	25	2
			VPENTMHAMQQK	30	103058.1	5.25	14.91	85.6	6.72	25	3
actinin, alpha 2	4501893	ACTN2	CQLEINFNTLQTK	7	103854.4	5.31	12.33	78.2	5.99	25	2
			DLLLDPAWEK	7	103854.4	5.31	15.93	96.6	4.03	18	2
			EGLLLWCQR	7	103854.4	5.31	12.12	84.7	6.09	25	2
			FAIQDISVEETSAK	7	103854.4	5.31	14.45	81.9	4.14	25	2
			HTNYTMEHIR	7	103854.4	5.31	11.14	76.3	6.92	23	3
			LMLLLEVISGER	7	103854.4	5.31	12.29	71.5	4.53	25	2
			TIPWLENR	7	103854.4	5.31	10.31	94.5	5.66	17	2
actinin, alpha 3	4557241	ACTN3	ALDFIASK	6	103294.9	5.4	15.62	94.2	5.88	25	2
			CQLEINFNTLQTK	6	103294.9	5.4	12.33	78.2	5.99	25	2
			DLLLDPAWEK	6	103294.9	5.4	15.93	96.6	4.03	18	2
			EGLLLWCQR	6	103294.9	5.4	12.12	84.7	6.09	25	2
			FAIQDISVEETSAK	6	103294.9	5.4	14.45	81.9	4.14	25	2
			LMLLLEVISGER	6	103294.9	5.4	12.29	71.5	4.53	25	2
actinin, alpha 4	12025678	ACTN4	AIMTYVSSFYHAFSGAQK	14	104854.6	5.27	17.25	87.1	8.55	25	3
			ALDFIASK	14	104854.6	5.27	15.62	94.2	5.88	25	2
			CQLEINFNTLQTK	14	104854.6	5.27	12.33	78.2	5.99	25	2
			DLLLDPAWEK	14	104854.6	5.27	15.93	96.6	4.03	18	2
			EGLLLWCQR	14	104854.6	5.27	12.12	84.7	6.09	25	2
			ELPPDQAEYCIAR	14	104854.6	5.27	12.99	90.9	4.14	25	2
			FAIQDISVEETSAK	14	104854.6	5.27	14.45	81.9	4.14	25	2
			HTNYTMEHIR	14	104854.6	5.27	11.14	76.3	6.92	23	3
			LASDLLEWIR	14	104854.6	5.27	14.7	86	4.37	25	2
			LMLLLEVISGER	14	104854.6	5.27	12.29	71.5	4.53	25	2
			LSGSNPYTTVTPQIINSK	14	104854.6	5.27	10.47	74.8	8.59	25	2
			MAPYQGPDAVPGALDYK	14	104854.6	5.27	16.13	83.7	4.21	25	2
			TINEVENQILTR	14	104854.6	5.27	18.9	86.3	4.53	25	2
			VGWEQLLTTIAR	14	104854.6	5.27	17.13	88.3	5.97	25	2
adenomatosis polyposis coli	53759122	APC	AEIKEKIGTRSAEDPV	2	311647.5	7.92	13.04	77	4.87	25	3
			TRSAEDPVSEVPAVSQHPR	2	311647.5	7.92	12.76	78.9	5.41	25	3
ADP-ribosylation factor 1	4502201	ARF1	QDLPNAMNAAEITDK	1	20696.9	6.31	18.78	90.7	4.03	25	2
ADP-ribosylation factor-like 10C	8922601	ARL8B	DLPNALDEK	4	21539.2	8.67	16.4	90.1	4.03	25	2
			EICCYSISCK	4	21539.2	8.67	13.4	76.1	6.07	25	2
			IWDIGGQPR	4	21539.2	8.67	10.38	84.4	5.84	25	2
			MNLSAIQDR	4	21539.2	8.67	17.34	93	5.59	25	2
AHNAK nucleoprotein isoform 1	61743954	AHNAK	AEGPEVDVNLPK	12	629104.8	5.8	10.25	71.3	4.14	25	2
			GEGPDVDVNLPK	12	629104.8	5.8	14.36	82.8	4.03	25	2
			IDVTAPDVSIEEPEGK	12	629104.8	5.8	13.03	74.2	3.83	25	2
			VDIDAPDVSIEGPDAK	12	629104.8	5.8	12.04	71.1	3.71	21	2
			VDIDVPDVNIEGPEGK	12	629104.8	5.8	16.7	90.6	3.77	25	2
			VDIEGPDVNIEGPEGK	12	629104.8	5.8	16.57	81.5	3.83	25	2
			VDTNAPDLSLEGPEGK	12	629104.8	5.8	18.5	92.7	3.91	25	2
			VDVDVPDVNIEGPDAK	12	629104.8	5.8	15.8	88	3.71	25	2
			VDVEVPDVSLEGPEGK	12	629104.8	5.8	12.15	77.6	3.83	25	2
			VEGDLKGPEVDIK	12	629104.8	5.8	12.83	80.3	4.32	25	3
			VEGDLKGPEVDLK	12	629104.8	5.8	12.83	80.3	4.32	25	3
			VNVEAPDVNLEGLGGK	12	629104.8	5.8	13.17	82.8	4.14	25	2
albumin precursor	4502027	ALB	AQYLQQCPFEDHVK	26	69367.1	5.92	14.94	85.8	5.32	25	3
			AVMDDFAAFVEK	26	69367.1	5.92	20.08	96	4.03	25	2
			EFNAETFTFHADICTLSEK	26	69367.1	5.92	11.03	76	4.4	25	3
			ETYGEMADCCAK	26	69367.1	5.92	14.03	88.1	4.14	25	2
			FKDLGEENFK	26	69367.1	5.92	12.13	77.6	4.68	25	3
			FQNALLVR	26	69367.1	5.92	15.19	92.8	9.75	25	2
			FSALEVDETYVPK	26	69367.1	5.92	19.76	92.6	4.14	25	2
			FYAPELLFFAK	26	69367.1	5.92	17.2	96.9	6	21	2
			HPYFYAPELLFFAK	26	69367.1	5.92	11.21	83.7	6.75	15	3
			KLVAASQAALGL	26	69367.1	5.92	11.69	72.4	8.75	25	2
			KQTALVELVK	26	69367.1	5.92	14.16	88.3	8.59	25	2
			KVPQVSTPTLVEVSR	26	69367.1	5.92	14.1	81.7	8.75	25	3
			LKECCEKPLLEK	26	69367.1	5.92	15.51	90.4	6.21	25	3

			LVNEVTEFAK	26	69367.1	5.92	17.59	94.2	4.53	25	2
			LVRPEVDVM	26	69367.1	5.92	11.35	79	4.37	25	2
			LVRPEVDVMCTAFHDNEETFLK	26	69367.1	5.92	14.31	76.4	4.5	25	3
			MPCAEDYLSVVLNQLCVLHEK	26	69367.1	5.92	12.21	73.7	4.65	25	3
			NECFLQHKDDNPNLPR	26	69367.1	5.92	16.41	82.7	5.38	25	3
			QNCELFEQLGEYK	26	69367.1	5.92	18.83	96.3	4.25	25	2
			QTALVELVK	26	69367.1	5.92	12.91	87.6	6	23	2
			RPCFSALEVDETYVPK	26	69367.1	5.92	23.85	97.9	4.68	25	3
			SLHTLFGDK	26	69367.1	5.92	12.06	85.5	6.46	25	2
			VFDEFKPLVEEPQNLIK	26	69367.1	5.92	19.02	92.5	4.41	25	3
			VHTECCHGDLLECADDR	26	69367.1	5.92	12.49	72.4	4.42	25	3
			VPQVSTPTLVEVSR	26	69367.1	5.92	13.89	71.5	5.97	25	2
			YICENQDSISSK	26	69367.1	5.92	14.14	82.6	4.37	25	2
aldolase A	4557305	ALDOA	GILAADESTGSIAK	5	39420.2	8.3	20.02	93.6	4.37	25	2
			IGEHTPSALAIMENANVLAR	5	39420.2	8.3	11.47	74	5.4	23	3
			PYQYPALTPEQK	5	39420.2	8.3	13.2	75.2	6.41	23	2
			QLLLTADDR	5	39420.2	8.3	11.08	83.7	4.21	25	2
			VNPCIGGVILFHETLYQK	5	39420.2	8.3	14.8	81.2	6.71	25	3
alpha 1 actin precursor	4501881	ACTA1	AVFPSIVGR	4	42051.3	5.23	17.13	88	9.79	25	2
			DLTDYLMK	4	42051.3	5.23	13.9	91.4	4.21	25	2
			EITALAPSTMK	4	42051.3	5.23	12.12	87.5	6.1	25	2
			SYELPDGQVITIGNER	4	42051.3	5.23	21.93	88.7	4.14	25	3
alpha 2 globin	4504345	HBA1 HBA2	MFLSFPTTK	1	15257.6	8.72	13.45	91.3	8.5	25	2
alpha 3 type IV collagen isoform 1 precursor	89142730	COL4A3	SHVIGIKGDKGSMGHPGPKGPPGTAGD	2	161813.4	9.28	13.12	89.1	8.27	25	3
			VPGDPGLR	2	161813.4	9.28	11.03	70.3	5.81	20	2
alpha glucosidase II alpha subunit isoform 2	38202257	GANAB	MMDYLQGSGETPQTDVR	3	106874.5	5.73	16.49	85	4.03	25	2
			SLLLSVNAR	3	106874.5	5.73	11.63	72.1	9.47	25	2
			VSQGSKDPAEGDGAQPEETPR	3	106874.5	5.73	17.4	84.8	4.18	25	3
alpha glucosidase II alpha subunit isoform 3	88900491	GANAB	MMDYLQGSGETPQTDVR	2	109438.5	5.82	16.49	85	4.03	25	2
			SLLLSVNAR	2	109438.5	5.82	11.63	72.1	9.47	25	2
alpha-N-acetylgalactosaminidase precursor	4557781	NAGA	ASALVFFSCR	6	46564.9	4.98	14.14	82.6	8.3	25	2
			INQDPLGIQGR	6	46564.9	4.98	15.66	79	5.84	25	2
			LDGCFSTPEER	6	46564.9	4.98	11.4	80.9	4.14	25	2
			LDNGLLQTPPMGWLAWER	6	46564.9	4.98	15.35	86.9	4.37	25	3
			TISAQNMDILQNPLMIK	6	46564.9	4.98	17.64	94.7	5.5	21	3
			VVQDAQTFAEWK	6	46564.9	4.98	19.15	90.8	4.37	25	2
annexin 5	4502107	ANXA5	GTVTDFPGFDER	3	35936.9	4.94	13.7	81.5	4.03	25	2
			IDRETSGNLEQLLLAVVKSIR	3	35936.9	4.94	10.94	73.9	6.18	25	3
			SEIDLFNIR	3	35936.9	4.94	13.36	81.7	4.37	25	2
annexin A2 isoform 1	50845388	ANXA2 ANXA2P2	DALNIETAIK	5	28766.3	8.53	12.66	77.9	4.37	25	2
			LSLEGDHSTPPSAYGSVK	5	28766.3	8.53	17.71	89.1	5.32	25	3
			QDIAFAYQR	5	28766.3	8.53	11.22	85.2	5.83	25	2
			RAEDGSVIDYELIDQDAR	5	28766.3	8.53	18.06	85.2	3.96	25	3
			SLYYYIQQDTK	5	28766.3	8.53	12.92	78.8	5.55	25	2
annexin VI isoform 1	71773329	ANXA6	DAFVAIVQSVK	4	75873.7	5.42	13.2	78.5	5.84	24	2
			GTVRPANDFNPDADAK	4	75873.7	5.42	13.04	85.2	4.43	20	3
			LIVGLMRPPAYCDAK	4	75873.7	5.42	11.11	85.2	8.2	19	3
			SEIDLLNIR	4	75873.7	5.42	11.09	82.7	4.37	17	2
apolipoprotein A-I preproprotein	4557321	APOA1	DEPPQSPWDR	9	30778	5.56	16.07	91.5	4.03	25	2
			DYVSQFEGSALGK	9	30778	5.56	22.76	98.7	4.37	25	2
			EQLGPVTQEFWDNLEK	9	30778	5.56	12.65	78.2	4	19	3
			LLDNWDSVTSTFSK	9	30778	5.56	17.56	91.8	4.21	25	2
			LSPLGEEMR	9	30778	5.56	15.08	96.1	4.53	18	2
			QGLLPVLESFK	9	30778	5.56	15.2	78.6	6	25	2
			THLAPYSDELR	9	30778	5.56	12.19	74.8	5.29	25	3
			VQPYLDDFQK	9	30778	5.56	10.36	84.8	4.21	18	2
			VSFLSALEEYTK	9	30778	5.56	22.29	98	4.53	25	2
apolipoprotein H precursor	153266841	APOH	FICPLTGLWPINTLK	1	38298.4	8.34	19.43	89.7	8.22	25	2
ARP1 actin-related protein 1 homolog A, centractin alpha	5031569	ACTR1A	AGFAGDQIPK	3	53818.7	6.19	14.45	78.9	5.88	25	2
			AQYYLPDGSTIEIGPSR	3	53818.7	6.19	15.21	70.9	4.37	25	2
			DQLQTFSEEHPVLLTEAPLNPR	3	53818.7	6.19	17.31	77.5	4.4	25	3
atlastin isoform a	19923445	ATL1	ILIPWLLSPESLDIK	1	63543.8	5.82	13.61	83.3	4.37	18	2
ATP synthase, H+ transporting, mitochondrial F1 complex, alpha subunit precursor	4757810	ATP5A1	FENAFLSHVVSQHQALL	2	59750.9	9.16	12.84	81.5	5.98	25	3
			TGAIVDVPVGEELLGR	2	59750.9	9.16	10.34	76.6	4.14	21	2
ATPase, H+ transporting, lysosomal 31kDa, V1 subunit E isoform 2	18087815	ATP6V1E2	AEEEFNIEK	1	149814.4	8.79	16.28	92.7	4.09	25	2
ATPase, H+ transporting, lysosomal 42kDa, V1 subunit C1	4502315	ATP6V1C1	GVTQIDNDLK	1	15735.9	7.02	16.32	85.5	4.21	25	2
ATPase, H+ transporting, lysosomal 50/57kDa, V1 subunit H isoform 2	47717102	ATP6V1H	FLEKSTERETRQ	2	42274.4	6.07	12.87	91.1	6.23	25	3
			LLEVSDDPQVLAVAAHDVGEYVR	2	42274.4	6.07	13.56	77.4	4.1	25	3
ATPase, H+ transporting, lysosomal 56/58kDa, V1 subunit B1	19913426	ATP6V1B1	QIYPPINVLPSLSR	1	56833.4	5.44	17.16	90.3	8.75	25	2
ATPase, H+ transporting, lysosomal 70kD, V1 subunit A, isoform 1	19913424	ATP6V1A	LAEMPADSGYPAYLGAR	5	95756.6	5.35	24.88	96.7	4.37	25	2
			LIKDDFLQQNGYTPYDR	5	95756.6	5.35	11.9	70.4	4.43	25	3
			LPANHPLLTGQR	5	95756.6	5.35	21.56	88.2	9.75	25	3
			TALVANTSNMPVAAR	5	95756.6	5.35	20.94	95.4	9.41	25	2
			VGHSELVGEIIR	5	95756.6	5.35	12.61	71.4	5.4	25	3
ATPase, H+ transporting, lysosomal accessory protein 2	15011918	ATP6AP2	YGEDSEQFR	1	39008.2	5.76	13.23	82.3	4.14	25	2
ATPase, H+ transporting, lysosomal V0 subunit a isoform 1	19913418	ATP6V0A1	ANIPIMDTGENPEVPFPR	5	95756.6	6.22	13.27	72.3	4.14	25	3
			ASLYPCPETPQER	5	95756.6	6.22	15.28	83.8	4.53	25	2
			DLNPDVNVFQR	5	95756.6	6.22	13.34	82.1	4.21	25	2
			NFLELTELK	5	95756.6	6.22	11.05	76.9	4.53	25	2
			QAEIENPLEDPVTGDYVHK	5	95756.6	6.22	12.77	78.3	4.17	25	3
ATPase, H+ transporting, lysosomal, V0 subunit c	4502313	ATP6V0C	SALGAAYGTAK	1	15735.9	7.98	13.65	93.9	8.31	18	2
ATPase, H+ transporting, lysosomal, V0 subunit d1	19913432	ATP6V0D1	AGVLSQADYLNLVQCETLEDLK	7	40329.3	4.89	13.61	72.6	3.92	25	3
			AYLESFYK	7	40329.3	4.89	10.58	93.1	6.04	17	2
			LHLQSTDYGNFLANEASPLTVSVIDDR	7	40329.3	4.89	12.43	72.4	4.22	25	3
			LLFEGAGSNPGDK	7	40329.3	4.89	18.77	92.4	4.37	25	2
			LYPEGLAQLAR	7	40329.3	4.89	18.85	93.8	6	25	2
			NVADYYPEYK	7	40329.3	4.89	17.23	94.6	4.37	25	2
			SIAELVPK	7	40329.3	4.89	10.99	98.6	5.72	13	2
autoimmune regulator isoform 1	4557291	AIRE	GEARLGQQGSVPAPLALPSDPQLH	4	57727	7.53	14.2	80.1	5.32	25	3
			LHRTEIAVAVDSAFPLLHALADH	4	57727	7.53	13.09	75.5	5.73	25	3
			YGRLQPILDSFPKDV	4	57727	7.53	13.02	80.8	5.96	25	3
			YKHLPAPPSAAPLPGLDSSALHPLLCVG	4	57727	7.53	10.38	77.9	6.91	20	3
beta globin	4504349	HBB	EFTPPVQAAYQK	7	15998.5	6.74	15.36	82	6.1	25	2
			FFESFGDLSTPDAVMGNPK	7	15998.5	6.74	17.39	83.8	4.03	25	3
			GTFATLSELHCDK	7	15998.5	6.74	11.94	84.8	5.32	18	3
			LLVVYPWTQR	7	15998.5	6.74	13.55	89.8	8.75	25	2
			TALWGKVNVDEVGGEALGR	7	15998.5	6.74	13.11	81.6	4.68	25	3
			VLGAFSDGLAHLDNLK	7	15998.5	6.74	18.3	95.1	5.21	25	3
			VNVDEVGGEALGR	7	15998.5	6.74	18.78	92.5	4.14	25	3
beta tubulin 1, class VI	13562114	TUBB1	LAVNMVPFPR	1	50327.2	5.06	17.49	92.5	9.75	25	2
c-K-ras2 protein isoform a	15718763	KRAS	SYGIPFIETSAK	3	102380.5	6.33	10.36	77.7	5.72	25	2

			YDPTIEDSYR	3	102380.5	6.33	16.37	94.4	4.03	25	2
			YDPTIEDSYRK	3	102380.5	6.33	10.37	71.7	4.56	25	3
cadherin EGF LAG seven-pass G-type receptor 2	13325064	CELSR2	ARTPMEVTVTVLDVNDN	3	317454.2	5.14	13.31	75	4.03	25	3
			LVPHHDGLRVWCPES	3	317454.2	5.14	11.23	90.5	5.99	17	3
			STHVFRVTAQDHGMPRRSALATL	3	317454.2	5.14	12.3	83.8	11.7	25	3
calreticulin precursor	4757900	CALR	EQFLDGDGWTSR	1	24220.1	4.29	13.81	86.9	4.03	25	2
carboxypeptidase E precursor	4503009	CPE	AASQPGELKDWFVGR	15	53150.9	5.03	19.06	92.2	6.12	25	3
			AYSSFNPAMSDPNRPPCR	15	53150.9	5.03	14.42	83.9	8.26	25	3
			ELLVIELSDNPGVHEPGEPEFK	15	53150.9	5.03	13.33	75.3	4.14	25	3
			FPPEETLK	15	53150.9	5.03	12.49	97.9	4.53	17	2
			IHIMPSLNPDGFEK	15	53150.9	5.03	21.21	93.6	5.32	25	3
			KVAVPYSPAAGVDFELESFSER	15	53150.9	5.03	10.87	78.1	4.41	19	3
			LHGGDLVANYPYDETR	15	53150.9	5.03	17.47	85.4	4.54	25	3
			LLIPGNYK	15	53150.9	5.03	10.05	86.7	8.59	22	2
			LQQEDGISFEYHR	15	53150.9	5.03	14.36	86.2	4.65	25	3
			LTASAPGYLAITK	15	53150.9	5.03	13.09	84	8.59	25	2
			NSLISYLEQIHR	15	53150.9	5.03	14.81	77.6	6.75	25	3
			SGSAHEYSSSPDDAIFQSLAR	15	53150.9	5.03	20.01	88.7	4.53	25	3
			SNAQGIDLNR	15	53150.9	5.03	14.58	78.5	5.55	25	2
			VAVPYSPAAGVDFELESFSER	15	53150.9	5.03	24.12	96.6	4	25	2
			YIGNMHGNEAVGR	15	53150.9	5.03	14.24	88.3	6.75	25	3
cardiac calsequestrin 2	119395727	CASQ2	WIEDVLSGKINT	1	46436	4.22	15.05	80.5	4.37	25	2
CASK interacting protein 1	18079216	CASKIN1	DSEPERDE	1	149814.4	9.21	14.71	83.2	4.04	25	2
cathepsin A precursor	119395729	CTSA	DLECVTNLQEVAR	2	56233.3	6.17	15.63	81.5	4.14	25	2
			YGDSGEQIAGFVK	2	56233.3	6.17	12.21	70.8	4.37	25	2
cathepsin B preproprotein	4503139	CTSB	EQWPQCPTIK	1	37821.8	5.88	14.04	84.5	6.09	25	2
cathepsin D preproprotein	4503143	CTSD	AIGAVPLIQGEYMIPCEK	9	44552.5	6.1	18.6	87.6	4.53	25	2
			DPDAQPGGELMLGGTDSK	9	44552.5	6.1	20.3	93.1	3.84	25	2
			EGCEAIVDTGTSLMVGPVDEVR	9	44552.5	6.1	20.34	92.5	3.83	25	2
			FDGILGMAYPR	9	44552.5	6.1	11.59	73.4	5.83	25	2
			GPIPEVLK	9	44552.5	6.1	12.85	82.9	6	25	2
			IQGEYMIPCEK	9	44552.5	6.1	11.11	78.5	4.53	25	2
			LVDQNIFSF	9	44552.5	6.1	14.75	93.3	3.8	25	2
			VGFAEAAR	9	44552.5	6.1	20.31	95.8	5.97	24	2
			YYTVFDR	9	44552.5	6.1	13.74	89.3	5.83	22	2
cathepsin Z preproprotein	22538442	CTSZ	NSWGEPWGER	3	33868.1	6.7	10.4	76.5	4.53	16	2
			NVDGVNYASITR	3	33868.1	6.7	14.86	100	5.83	14	2
			VGDYGSLSGR	3	33868.1	6.7	16.21	86.8	5.8	25	2
CD14 antigen precursor	4557417	CD14	ITGTMPPLPLEATGLALSSLR	3	40076.4	5.84	13.85	81.1	6	25	2
			LTVGAAQVPAQLLVGALR	3	40076.4	5.84	11.42	70	9.75	25	2
			VLSIAQAHSPAFSCEQVR	3	40076.4	5.84	14.75	85.5	6.71	25	3
CD59 antigen p18-20	10835165	CD59	LQCYNCPNPTADCK	1	14177.4	6.03	13.47	76	5.81	24	2
CD63 antigen isoform A	4502679	CD63	VMSEFNNNFR	1	25636.8	8.14	12.27	85.2	5.97	25	2
CD93 antigen precursor	88758613	CD93	VLAQLLR	1	199527.2	5.27	13.71	94.2	9.72	22	2
cell division cycle 42 isoform 1	4757952	CDC42	CVVVGDGAVGK	3	21258.7	6.16	11.44	78.1	5.83	25	2
			NVFDEAILAALEPPEPK	3	21258.7	6.16	18.34	84.7	4	25	3
			YVECSALTQK	3	21258.7	6.16	14.81	86.8	5.99	25	2
chloride intracellular channel 1	14251209	CLIC1	NSNPALNDNLEK	1	26922.9	5.09	14.71	86.3	4.37	21	2
chondroitin sulfate proteoglycan 4	47419930	CSPG4	QVRLVLGQEELR	2	126485.3	5.26	11.13	74.4	6.14	25	2
			SHLWKNKGLWVPE	2	126485.3	5.26	13.15	71.8	8.33	25	3
chromogranin A precursor	4502805	CHGA	CIVEVISDTLSK	23	50688.7	4.58	17.3	88.6	4.37	25	2
			CIVEVISDTLSKPSPMPVSQECFETLR	23	50688.7	4.58	22.4	96.2	4.41	25	3
			EAVEEPSSKDVMEK	23	50688.7	4.58	12.11	85	4.25	25	3
			EDSLEAGLPLQVR	23	50688.7	4.58	18.87	93.4	4.14	25	2
			EEEEEMAVVPQGLFR	23	50688.7	4.58	15.92	92.5	3.98	25	2
			EKGLSAEPGWQAK	23	50688.7	4.58	12.5	88.2	6.24	24	3
			ELQDLALQGAK	23	50688.7	4.58	17.5	96.2	4.37	25	2
			GEQEHSQQKEEEEEMAVVPQGLFR	23	50688.7	4.58	20.13	92.3	4.35	25	3
			GLSAEPGWQAK	23	50688.7	4.58	17.65	89.7	6	25	2
			GPQAEGDSEGLSQGLVDR	23	50688.7	4.58	19.78	89.4	3.91	25	2
			LEGQEEEEDNRDSSMK	23	50688.7	4.58	16.79	89.9	4	25	3
			LPVNSPMNK	23	50688.7	4.58	15.85	89.4	8.75	25	2
			LPVNSPMNKGDTEVMK	23	50688.7	4.58	20	89.3	6.07	25	3
			PQALPEPMQESK	23	50688.7	4.58	14.26	87.4	4.53	25	2
			PSPMPVSQECFETLR	23	50688.7	4.58	18.91	88.3	4.53	25	2
			RLEGQEEEEDNRDSSMK	23	50688.7	4.58	14.85	76.1	4.25	25	3
			RPEDQELESLSAIEAELEK	23	50688.7	4.58	18.34	93.6	4.06	25	3
			SAIEAELEK	23	50688.7	4.58	12.82	71.7	4.25	25	2
			SEALAVDGAGKPGAEEAQD	23	50688.7	4.58	15.95	87.1	4.02	25	2
			SEALAVDGAGKPGAEEAQDPEGK	23	50688.7	4.58	27.03	96.4	4.08	25	3
			SGEATDGARPQALPEPMQESK	23	50688.7	4.58	27.39	99.4	4.41	25	3
			YPGPQAEGDSEGLSQGLVDR	23	50688.7	4.58	19.39	93.9	3.91	25	2
			YPGPQAEGDSEGLSQGLVDREK	23	50688.7	4.58	15.07	77.4	4.18	25	3
chromogranin B precursor	4502807	CHGB	ADEPQWSLYPSDSQVSEEVK	38	78246.6	5.02	15.88	90.3	3.83	25	2
			ADQTVLTEDEK	38	78246.6	5.02	16.6	92.5	3.91	25	2
			ADQTVLTEDEKK	38	78246.6	5.02	11.48	73.8	4.32	25	3
			APRPQSEESWDEED	38	78246.6	5.02	11.95	78.1	3.94	25	2
			APRPQSEESWDEEDKR	38	78246.6	5.02	16.99	88.4	4.36	25	3
			ASEEEPEYGEEIK	38	78246.6	5.02	18.09	90.9	3.9	25	2
			AYFMSDTREE	38	78246.6	5.02	14.53	96.5	4.43	15	2
			CIIEVLSNALSK	38	78246.6	5.02	26.84	98.9	5.99	25	2
			ERADEPQWSLYPSDSQVSEEVK	38	78246.6	5.02	20.93	93.8	4.08	25	3
			ETTENENTKFEVR	38	78246.6	5.02	11.94	76.5	4.49	25	3
			FNPYYDPLQWK	38	78246.6	5.02	11.62	78.5	5.83	23	2
			GEAGAPGEEDIQGPTK	38	78246.6	5.02	20.37	95	4	25	2
			GEAGAPGEEDIQGPTKADTEK	38	78246.6	5.02	20.73	95.7	4.08	25	3
			GHPQEESEESNVSMASLGE	38	78246.6	5.02	13.63	80.3	4.19	25	2
			GHPQEESEESNVSMASLGEK	38	78246.6	5.02	19.72	91.2	4.32	25	3
			GYPGVQAPEDLEWER	38	78246.6	5.02	11.45	74.2	4	25	2
			HLEEPGETQNAFLNER	38	78246.6	5.02	22.61	98.2	4.48	25	2
			KPFSEDVNWGYE	38	78246.6	5.02	12.41	82.4	4.43	25	2
			KPFSEDVNWGYEK	38	78246.6	5.02	17.94	90.6	4.68	25	3
			KPFSEDVNWGYEKR	38	78246.6	5.02	13.98	77.6	6.18	25	3
			LFNPYYDPLQWK	38	78246.6	5.02	14.6	86.9	5.83	25	2
			LGELFNPYYDPLQWK	38	78246.6	5.02	12.97	75.5	4.37	25	2
			NFFPEYNYDWWEK	38	78246.6	5.02	17.99	89.4	4.14	25	3
			NYLNYGEEGAPGK	38	78246.6	5.02	17.87	92.4	4.53	25	2
			PQSEESWDEEDKR	38	78246.6	5.02	12.75	73.9	4.08	25	3
			RDNMNDNFLEGEEENELTLNEK	38	78246.6	5.02	15.55	72.9	3.94	25	3
			RLGELFNPYYDPLQWK	38	78246.6	5.02	11.79	73.5	6.07	25	3
			SQEESEEGEEDATSEVDK	38	78246.6	5.02	22.19	96.3	3.63	25	2

			SQEESEEGEEDATSEVDKR	38	78246.6	5.02	11.81	85.2	3.89	25	3
			SQREDEEEEEGENYQK	38	78246.6	5.02	18.74	94.9	3.99	25	3
			SSAPPITPECR	38	78246.6	5.02	17.96	93.7	5.72	25	2
			SSQGGSLPSEEK	38	78246.6	5.02	10.21	90	4.53	11	2
			VAQLDQLLH	38	78246.6	5.02	17.76	92.2	5.08	25	2
			VAQLDQLLHY	38	78246.6	5.02	11.07	79.5	5.07	25	2
			VAQLDQLLHYR	38	78246.6	5.02	12.28	82.1	6.71	25	3
			WQQQGDLQDTK	38	78246.6	5.02	11.3	85.6	4.21	25	2
			WQQQGDLQDTKENR	38	78246.6	5.02	13.06	72.2	4.56	25	2
			YLNYGEEGAPGK	38	78246.6	5.02	14.07	84.6	4.53	23	2
clathrin heavy chain 1	4758012	CLTC	AFMTADLPNELIELLEK	28	191615.7	5.48	14.05	75.4	4	25	2
			AVDVFFPPEAQNDFPVAMQISEK	28	191615.7	5.48	13.91	78.1	3.91	25	3
			CNEPAVWSQLAK	28	191615.7	5.48	15.51	82.7	5.99	25	2
			DPHLACVAYER	28	191615.7	5.48	11.17	74.1	5.32	25	3
			GQCDLELINVCNENSLFK	28	191615.7	5.48	15.31	86.6	4.14	25	3
			GQFSTDELVAEVEK	28	191615.7	5.48	11.31	74.4	4	25	2
			IHEGCEEPATHNALAK	28	191615.7	5.48	14.81	81.5	5.32	25	3
			ISGETIFVTAPHEATAGIIGVNR	28	191615.7	5.48	14.24	75.9	5.4	25	3
			IVLDNSVFSEHR	28	191615.7	5.48	12.37	90.4	5.32	25	3
			IYIDSNNNPER	28	191615.7	5.48	17.33	96.8	4.37	22	2
			KFNALFAQGNYSEAAK	28	191615.7	5.48	15.32	83.6	8.5	25	3
			LAELEEFINGPNNAHIQQVGDR	28	191615.7	5.48	11.87	72	4.4	25	3
			LECSEELGDLVK	28	191615.7	5.48	10.79	70.4	4	25	2
			LHIIEVGTPPTGNQPFPK	28	191615.7	5.48	15.04	82.1	6.75	25	3
			LLLPWLEAR	28	191615.7	5.48	12.92	82	6	25	2
			LLYNNVSNFGR	28	191615.7	5.48	16.13	85.4	8.75	25	2
			LPVVIGGLLDVDCSEDVIK	28	191615.7	5.48	20.54	92.3	3.84	25	2
			LTDQLPLIIVCDR	28	191615.7	5.48	11.51	71.3	4.21	25	2
			NLQNLLILTAIK	28	191615.7	5.48	14.96	88.4	8.75	25	2
			NNLAGAEELFAR	28	191615.7	5.48	17.39	82.8	4.53	25	2
			RDPHLACVAYER	28	191615.7	5.48	10.56	77.2	6.74	18	3
			RPLIDQVVQTALSETQDPEEVSVTVK	28	191615.7	5.48	20.66	89.6	4.18	25	3
			SVDPTLALSVYLR	28	191615.7	5.48	15.66	84.2	5.55	25	2
			VANVELYYR	28	191615.7	5.48	18.18	96.1	5.97	25	2
			VDKLDASESLR	28	191615.7	5.48	10.48	72.7	4.56	25	3
			VSQPIEGHAASFAQFK	28	191615.7	5.48	12.92	75.2	6.72	25	3
			VVGAMQLYSVDR	28	191615.7	5.48	15.59	88.4	5.8	25	2
			YESLELCRPVLQQGR	28	191615.7	5.48	11.63	85.9	6.14	25	3
clathrin, heavy polypeptide-like 1	108860681	CLTCL1	AFMTADLPNELIELLEK	9	187003.9	5.55	14.05	75.4	4	25	2
			DPHLACVAYER	9	187003.9	5.55	11.17	74.1	5.32	25	3
			GQFSTDELVAEVEK	9	187003.9	5.55	11.31	74.4	4	25	2
			IVLDNSVFSEHR	9	187003.9	5.55	12.37	90.4	5.32	25	3
			LECSEELGDLVK	9	187003.9	5.55	10.79	70.4	4	25	2
			LTDQLPLIIVCDR	9	187003.9	5.55	11.51	71.3	4.21	25	2
			NLQNLLILTAIK	9	187003.9	5.55	14.96	88.4	8.75	25	2
			RDPHLACVAYER	9	187003.9	5.55	10.56	77.2	6.74	18	3
			VVGAMQLYSVDR	9	187003.9	5.55	15.59	88.4	5.8	25	2
CLIP-associating protein 2	57863301	CLASP2	VALIDRMG	1	60343.9	8.5	14.11	91.6	5.81	20	2
clusterin isoform 1	42716297	CLU	ASSIIDELFQDR	9	57832.9	6.25	18.69	90.2	4.03	25	2
			DQTVSDNELQEMSNQGSK	9	57832.9	6.25	16.46	91.1	3.91	25	3
			EILSVDCSTNNPSQAK	9	57832.9	6.25	13.37	74.8	4.37	25	3
			ELDESLQVAER	9	57832.9	6.25	15.17	84.8	4	25	2
			IDSLLENDR	9	57832.9	6.25	14.26	81.1	4.03	25	2
			KTLLSNLEEAKK	9	57832.9	6.25	11.85	88.3	8.5	19	3
			LFDSDPITVTVPVEVSR	9	57832.9	6.25	13.59	71	4.03	25	2
			TLLSNLEEAK	9	57832.9	6.25	15.98	93	4.53	25	2
			VTTVASHTSDSDVPSGVTEVVVK	9	57832.9	6.25	23.17	93.4	4.54	25	3
coatomer protein complex, subunit zeta 1	7706337	COPZ1	YYDDTYPSVK	1	20198.3	4.69	13.83	100	4.21	14	2
cocaine- and amphetamine-regulated transcript	4757910	CARTPT	ALDIYSAVDDASHEK	2	12829.2	8.55	14.06	93.7	4.22	25	3
			ELIEALQEVLK	2	12829.2	8.55	17.94	93.4	4.25	25	2
cofilin 1 (non-muscle)	5031635	CFL1	EILVGDVGQTVDDPYATFVK	2	18502.6	8.22	11.17	90.4	3.84	13	2
			YALYDATYETK	2	18502.6	8.22	10.38	73.6	4.37	25	2
complement component 3 precursor	115298678	C3	DFDFVPPVVR	10	187149.1	6.02	10.48	75.5	4.21	25	2
			DICEEQVNSLPGSITK	10	187149.1	6.02	16.94	86.6	4.14	25	2
			EPGQDLVVLPLSITTDFIPSFR	10	187149.1	6.02	14.23	81.2	4.03	25	2
			IPIEDGSGEVVLSR	10	187149.1	6.02	11.16	71.2	4.14	25	2
			LTTAKDKNRWEDPGKQLYNVE	10	187149.1	6.02	11.45	83.6	6.32	25	3
			QLYNVEATSYALLALLQLK	10	187149.1	6.02	11.46	77.6	6	25	3
			TGLQEVEVK	10	187149.1	6.02	15.56	82.9	4.53	25	2
			TIYTPGSTVLYR	10	187149.1	6.02	11.9	73.4	8.25	25	2
			VQLSNDFDEYIMAIEQTIK	10	187149.1	6.02	18.86	96.7	3.91	25	3
			VTIKPAPETEK	10	187149.1	6.02	12.93	86.9	6.11	18	3
complement component 7 precursor	45580688	C7	GQSISVTSIRPC	6	57136.5	6.09	11.89	80.6	8.25	25	2
			LLEPHCFPLSLVPTEFCPSPPALK	6	57136.5	6.09	18.34	83.3	5.4	25	3
			NVVYTCNEGYSLIGNPVAR	6	57136.5	6.09	13.09	72.2	5.99	25	3
			SVAVYGQYGGQPCVGNAFETQSCEPTR	6	57136.5	6.09	16.71	85.1	4.53	25	3
			VLFYVDSEK	6	57136.5	6.09	10.37	76.7	4.37	25	2
			YSAWAESVTNLPQVIK	6	57136.5	6.09	13.74	70.2	6	25	2
complement receptor 1 isoform S precursor	86793109	CR1	TIRCTSDPHGNGVWSSPAPRCELSVRAGHCKTPE	2	100895.7	6.61	12.32	70.8	7.71	25	6
			VTYRCNPGSGGRKVFEL	2	100895.7	6.61	13.86	80.9	9.31	25	3
cystatin C precursor	4503107	CST3	ALDFAVGEYNK	4	15799.3	9	14.97	82.6	4.37	25	2
			AVPWQGTMTLSK	4	15799.3	9	14.58	90.7	8.8	25	2
			LVGGPMDASVEEEGVRR	4	15799.3	9	13.3	98	4.41	18	3
			QIYAVPWQGTMTLSK	4	15799.3	9	16.57	86	8.59	25	2
cytochrome b-561	63054828	CYB561	EALLFNLGGK	2	27559.5	8.73	12.51	86.1	6.1	25	2
			RPSQAEEQALSMDFK	2	27559.5	8.73	14.33	83.5	4.68	25	3
cytochrome b5 reductase isoform 1	4503327	CYB5R3	STPAITLESPDIK	1	34235.1	7.18	12.51	71.9	4.37	25	2
cytochrome c	11128019	CYCS	ADLIAYLK	2	101484.8	9.59	14.33	82.8	5.88	25	2
			TGQAPGYSYTAANK	2	101484.8	9.59	19.1	95.1	8.16	25	2
cytokine induced apoptosis inhibitor 1	89274169	CIAPIN1	DNLLFVQITGKKPNFEVGSSRQ	2	161813.4	5.44	12.68	70.7	8.59	25	3
			VQITGKKPNFEVGSS	2	161813.4	5.44	10.45	87	8.56	22	2
cytosolic malate dehydrogenase	5174539	MDH1	FVEGLPINDFSR	4	36426.3	6.91	13.62	85.9	4.37	25	2
			GEFVTTVQQR	4	36426.3	6.91	13.63	75.2	6	25	2
			LGVTANDVK	4	36426.3	6.91	11.82	75	5.84	25	2
			VIVVGNPANTNCLTASK	4	36426.3	6.91	22.46	97.7	8.19	25	3
cytosolic sialic acid 9-O-acetylesterase homolog	24850115	SIAE	ELSNTAAYQSVR	4	58315.2	6.93	11.67	75.4	6.1	25	2
			FFPFGLVQLSSDLSK	4	58315.2	6.93	12.62	78.6	5.84	20	2
			SSDDGFPQIR	4	58315.2	6.93	11.06	70.8	4.21	25	2
			WHQTADFGYVPNPK	4	58315.2	6.93	10.73	89.4	6.74	16	3
dachsous 1 precursor	16933557	DCHS1	SAPRGLDRETTPA	2	346182.5	4.79	10.38	75.3	5.79	20	2
			TGELRARVPFDYEHTESFRLL	2	346182.5	4.79	12.21	89.4	5.5	25	3

dedicator of cytokinesis 9	24308029	DOCK9	ENDPEMLVDLQYSLAKSYAST	2	236447	7.25	12.56	80.4	3.92	25	3
			FREVRLIAISVLKNLLIKHS	2	236447	7.25	12.94	84.4	11	25	3
defensin, alpha 1 preproprotein	4758146	DEFA1	IPACIAGER	1	10201	6.54	14.51	84.2	6	25	2
development- and differentiation-enhancing factor 2	4502249	ASAP2	NAECLKLLLRGKASIEIANESGETPLDI	2	111651.2	6.24	12.86	72	4.58	25	3
			VRSVDRTSLHIVDFL	2	111651.2	6.24	12.67	77.6	6.72	25	3
diablo isoform 1 precursor	9845297	DIABLO	AVYTLTSLYR	2	61589.9	5.68	14.66	83.7	8.63	25	2
			LAEAQIEELR	2	61589.9	5.68	17.58	90.1	4.25	25	2
diablo isoform 3 precursor	21070976	DIABLO	AVYTLTSLYR	2	22284.5	5.7	14.66	83.7	8.63	25	2
			LAEAQIEELR	2	22284.5	5.7	17.58	90.1	4.25	25	2
dickkopf homolog 3 precursor	40548389	DKK3	DCQPGLCCAFQR	8	38390.5	4.59	16.6	84.7	5.82	25	2
			DSECCGDQLCVWGHCTK	8	38390.5	4.59	15.68	90.2	4.54	25	3
			EVEELMEDTQHK	8	38390.5	4.59	13.13	81.6	4.25	25	3
			EVPDEYEVGSFMEEVR	8	38390.5	4.59	12.65	72	3.83	25	2
			GLLFPVCTPLPVEGELCHDPASR	8	38390.5	4.59	11.39	75.7	4.65	25	3
			LLDLITWELEPDGALDR	8	38390.5	4.59	14.82	88.1	3.77	25	2
			SAVEEMEAEEAAAK	8	38390.5	4.59	15.42	72.4	3.98	25	2
			SLTEEMALR	8	38390.5	4.59	16.23	83.1	4.53	25	2
dipeptidyl peptidase 7 preproprotein	62420888	DPP7	AASNIIFSNGNLDPWAGGGIR	10	54341.7	5.91	18.54	84.6	5.88	25	3
			AGARRAPDPGFQER	10	54341.7	5.91	11.57	79.8	9.56	12	3
			APDPGFQER	10	54341.7	5.91	10.94	89.4	4.37	11	2
			ASHPEDPASVVEAR	10	54341.7	5.91	17.1	82.7	4.65	25	3
			DLFLQGAYDTVR	10	54341.7	5.91	19.63	94.4	4.21	25	2
			DVTADFEGQSPK	10	54341.7	5.91	19.02	98.9	4.03	25	2
			LYHSCADPTGCGTGPDAR	10	54341.7	5.91	23.51	97.9	5.21	25	3
			NAFTVLAMMDYPYPTDFLGPLPANPVK	10	54341.7	5.91	15.49	81	4.21	25	3
			SLPFGAQSTQR	10	54341.7	5.91	17.04	90.5	9.47	25	2
			WEFGTCQPLSDEK	10	54341.7	5.91	10.58	70.1	4.14	25	2
dopamine beta-hydroxylase precursor	116534900	DBH	AFYYPEEAGLAFGGPGSSR	24	69065.4	5.97	20.74	91.6	4.53	25	2
			AGPTVVSIGGGK	24	69065.4	5.97	14.32	84.5	8.8	25	2
			ALYSFAPISMH	24	69065.4	5.97	15.73	88.8	6.78	25	2
			CTQLALPPSGIHIF	24	69065.4	5.97	12.46	78.9	6.73	25	2
			DYLIEDGTVHLVYGILEEPFR	24	69065.4	5.97	16.59	87.4	4.17	25	3
			ETAFILTGYCTDK	24	69065.4	5.97	18.69	93.9	4.37	25	2
			EWEIVNQDNHYSPHFQEIR	24	69065.4	5.97	18.09	83.3	4.8	25	3
			FNAGIMELGLVYTPVMAIPPR	24	69065.4	5.97	20.11	89.4	6	25	3
			FNNEDVCTCPQASVSQQFTSVPWNSFNR	24	69065.4	5.97	14.12	83	4.37	25	3
			GILEEPFR	24	69065.4	5.97	14.28	88	4.53	20	2
			GQIHLDPQQDYQLLQVQR	24	69065.4	5.97	12.22	78.4	5.21	25	3
			IQIPSQETTYWCYIK	24	69065.4	5.97	21.94	96.2	5.99	25	2
			KVVSVHPGDVLITSCTYNTEDR	24	69065.4	5.97	14.13	89.8	5.38	18	3
			LEVHYHNPLVIEGR	24	69065.4	5.97	15.31	77.3	6	25	3
			RFNAGIMELGLVYTPVMAIPPR	24	69065.4	5.97	12.82	76.5	8.75	25	3
			SAVDAGFLQK	24	69065.4	5.97	15.52	77.4	5.55	25	2
			SPAGPTVVSIGGGK	24	69065.4	5.97	11.72	70.8	8.47	25	2
			TPEGLTLLFK	24	69065.4	5.97	11.49	82.1	5.66	25	2
			VISTLEEPTPQCPTSQGR	24	69065.4	5.97	16.96	88.5	4.53	25	2
			VQLLKPNIPEPELPSDA	24	69065.4	5.97	17.81	77.7	4.14	25	2
			VQLLKPNIPEPELPSDACTMEVQAPN	24	69065.4	5.97	16.53	79.8	4	25	3
			VVSVHPGDVLITSCTY	24	69065.4	5.97	10.82	70.9	5.08	25	2
			YFHLINR	24	69065.4	5.97	13.5	85.4	8.75	25	2
			YVHYYPQTQLELCK	24	69065.4	5.97	14.43	72.6	6.74	25	3
dynactin 1 isoform 1	13259510	DCTN1	AGEEQQRGAIPGQAPGSVPGPGLVK	5	104854.6	5.61	14.78	71.4	6.19	25	3
			DLETSCSDIR	5	104854.6	5.61	17.22	81.4	4.03	25	2
			ELTNQQEASVER	5	104854.6	5.61	14.93	90.2	4.25	25	2
			GAIPGQAPGSVPGPGLVK	5	104854.6	5.61	12.97	75.8	8.75	25	2
			VTFSCAAGFGQR	5	104854.6	5.61	11.7	78.1	8.22	25	2
dynactin 2	5453629	DCTN2	LLGPDAAINLTDPDGALAK	1	29481	5.06	17.11	84.9	3.93	25	2
dynamin 1 isoform 1	59853099	DNM1	GISPVPINLR	1	97408.8	6.73	16.39	89.1	9.75	25	2
dynamin 2 isoform 1	56549121	DNM2	GISPVPINLR	1	136002	7.04	16.39	89.1	9.75	25	2
dynein, cytoplasmic 1, intermediate chain 2	24307879	DYNC1I2	DLEDKEGEIQAGAK	1	71456.9	5.08	14.6	93.6	4.18	24	3
dynein, cytoplasmic, heavy polypeptide 1	33350932	DYNC1H1	AELGEYIR	42	532410.6	6.01	11.34	72.9	4.53	25	2
			CSFGVTGLKLQGATCNNN	42	532410.6	6.01	13.21	84.9	8.06	25	3
			DFPLNDLLSATELDK	42	532410.6	6.01	16.19	84.1	3.84	25	2
			DFPLNDLLSATELDKIR	42	532410.6	6.01	11.98	78.5	4.23	23	3
			DVLLVAQGEMALEEFLK	42	532410.6	6.01	13.02	78.6	4	19	2
			EDLDKVEPAVIEAQNAVK	42	532410.6	6.01	11.41	80.3	4.18	25	3
			ENFIPTIVNFSAEEISDAIR	42	532410.6	6.01	14.24	87	4	25	3
			EQPWVSVQPR	42	532410.6	6.01	13.08	84.2	6.1	25	2
			FGNPLLVQDVESYDPVLNPVLNR	42	532410.6	6.01	22.24	94.5	4.03	25	3
			GIFEALRPLETLPVEGLIR	42	532410.6	6.01	13.87	78.4	4.79	25	3
			IQFVGACNPPTDPGR	42	532410.6	6.01	18.34	88.4	5.83	25	2
			IQGLTVEQAEAVVR	42	532410.6	6.01	13.04	81.2	4.53	25	2
			ITNQVIYLNPPIEECR	42	532410.6	6.01	13.31	78.2	4.53	22	3
			KLVPLLLEDGGEAPAALEAALEEK	42	532410.6	6.01	17.77	86.3	4.14	25	3
			LAETVFNFQEK	42	532410.6	6.01	11.18	82.6	4.53	25	2
			LLNTFLER	42	532410.6	6.01	12.65	85.5	6	22	2
			LQGEFQLR	42	532410.6	6.01	11.47	85	6	25	2
			LSLSNAISTALPLTQLR	42	532410.6	6.01	22.34	97.1	9.75	25	3
			MNTLLANGEVPGLFEGDEYATLMTQCK	42	532410.6	6.01	14.93	74.6	4	25	3
			MVVLSLPR	42	532410.6	6.01	10.47	86	9.5	25	2
			NYMSNPSYNYEIVNR	42	532410.6	6.01	12.65	76.7	6	25	2
			QDLADVVQVCEGK	42	532410.6	6.01	12.88	76	4.03	25	2
			QLQNISLAAASGGAK	42	532410.6	6.01	13.81	89.6	8.75	25	2
			SACDTVDTWLDDTAK	42	532410.6	6.01	16.17	86.1	3.77	25	2
			SIPLDEGEDEAQR	42	532410.6	6.01	14.24	88.7	3.83	25	2
			SLLQALNEVK	42	532410.6	6.01	13.99	85.6	5.72	25	2
			SVLVSAGNVK	42	532410.6	6.01	15.14	88.7	8.47	25	2
			TFSEILNR	42	532410.6	6.01	10.8	76.2	5.66	25	2
			TFSSIPVSR	42	532410.6	6.01	10.9	76.3	9.41	25	2
			TLINELVK	42	532410.6	6.01	10.68	99	5.66	12	2
			TLMAQSIYGGR	42	532410.6	6.01	12.52	71.4	8.41	25	2
			TPVIDADKPVSSQLR	42	532410.6	6.01	19.92	90.5	5.62	25	3
			TTDLLTDWEK	42	532410.6	6.01	12.46	79.1	4.03	21	2
			TVENIKDPLFR	42	532410.6	6.01	13.53	86.1	5.74	23	3
			VAAPDVVVPTLDTVR	42	532410.6	6.01	15.26	85.4	4.21	25	2
			VAEVLFDAADANAIEEVNLAYENVK	42	532410.6	6.01	18.69	87.6	3.77	25	3
			VDDLLIIEEK	42	532410.6	6.01	12.19	84.1	3.91	25	2
			VLLTTQGVDMISK	42	532410.6	6.01	14.26	79.8	5.81	25	2
			VLRPQVTAVAQQNQGEVPEPQDMK	42	532410.6	6.01	15.11	82.2	4.68	25	3
			VNFLPEIITLSK	42	532410.6	6.01	12.45	80.8	5.97	25	2
			VQVALEELQDLK	42	532410.6	6.01	14.8	83.9	4.14	25	2

			VWEQIDQMK	42	532410.6	6.01	12.12	79.5	4.37	25	2
elastin isoform a	126352440	ELN	FPGVGVLPGVPTGAGVKPK	2	43787.1	10.45	14.84	83.5	10	25	3
			VPGVGLPGVYPGGVLPGAR	2	43787.1	10.45	13.65	75.6	8.72	25	2
elastin microfibril interfacer 1	5901944	EMILIN1	APAPASAPPGPSEELLR	3	106695.7	5.1	13.93	86.4	4.53	25	2
			LDTVAGGLQGLR	3	106695.7	5.1	12.31	73.3	5.84	25	2
			RPPQECCSPELGR	3	106695.7	5.1	11.12	70.5	6.13	22	3
enolase 1	4503571	ENO1	LAMQEFMILPVGAANFR	2	47169.2	7.01	13.34	76.8	6	25	3
			YISPDQLADLYK	2	47169.2	7.01	15.21	88.4	4.21	25	2
epididymal secretory protein E1 precursor	5453678	NPC2	AVVHGILMGVPVPFPIPEPDGCK	4	16570.3	7.57	21.97	92.7	5.32	25	3
			DCGSVDGVIK	4	16570.3	7.57	11.27	74	4.21	25	2
			EVNVSPCPTQPCQLSK	4	16570.3	7.57	16.23	90.5	6.08	25	3
			SGINCPIQK	4	16570.3	7.57	16.9	94	7.94	25	2
epsilon globin	4885393	HBE1	LLVVYPWTQR	1	39456.1	8.67	13.55	89.8	8.75	25	2
ErbB3-binding protein 1	124494254	PA2G4	LVKPGNQNTQVTEAWNK	2	43787.1	6.13	12.65	84.8	8.59	21	3
			TAENATSGETLEENEAGD	2	43787.1	6.13	10.54	73.3	3.61	25	2
estradiol 17 beta-dehydrogenase 8	15277342	HSD17B8	VIGLTQTAARELG	1	26973.9	6.09	13.14	79.3	5.97	25	2
fas ligand	4557329	FASLG	HLQKELAELRE	2	31484.9	9.41	12.16	88.7	5.61	23	3
			RGQSCNNLPLSHKVYMRN	2	31484.9	9.41	13.83	78.2	10.05	25	3
ferritin, light polypeptide	20149498	FTL	LGGPEAGLGEYLFER	1	53879.6	5.51	17.85	85.5	4.25	25	2
filamin A, alpha	116063573	FLNA	AFGPGLQGGSAGSPAR	17	280019.6	5.7	13.49	79.3	9.79	25	2
			ATCAPQHGAPGPGPADASK	17	280019.6	5.7	20.05	90.3	6.78	25	3
			AWGPGLEGGVVGK	17	280019.6	5.7	12.3	71.4	6.05	25	2
			AYGPGIEPTGNMVK	17	280019.6	5.7	10.48	71.6	6.04	25	2
			GDEIPFSPYRVRAVPT	17	280019.6	5.7	10.01	82.9	6.07	14	3
			GLVEPVDVVDNADGTQTVNYVPSR	17	280019.6	5.7	12.86	70.8	3.84	25	3
			GTVEPQLEAR	17	280019.6	5.7	13.87	84.3	4.53	25	2
			IANLQTDLSDGLR	17	280019.6	5.7	13.65	78.7	4.21	23	2
			MDCQECPEGYR	17	280019.6	5.7	17.59	88.5	4.14	25	2
			NTSNAGAGALSVTIDGPSKVKMDC	17	280019.6	5.7	14.02	72.1	5.95	25	3
			SPYTVTVGQACNPSACR	17	280019.6	5.7	16.38	80	7.79	24	3
			TGVAVNKPAEFTVDAK	17	280019.6	5.7	16.42	90.9	5.73	25	3
			VANPSGNLTETYVQDR	17	280019.6	5.7	14.04	77.5	4.37	25	2
			VEPGLGADNSVVR	17	280019.6	5.7	14.84	90.7	4.37	25	2
			VQVQDNEGCPVEALVK	17	280019.6	5.7	13.24	70.1	4.14	25	2
			VTAQGPGLEPSGNIANK	17	280019.6	5.7	12.77	73.3	5.97	25	3
			YGGQPVPNFPSK	17	280019.6	5.7	12.87	70.1	8.59	25	2
filamin B, beta (actin binding protein 278)	105990514	FLNB	KVKAEGPGLSKAGV	3	278165.4	5.47	10.62	72.4	9.7	21	2
			NMLLIGVHGPTTPCEEVSMK	3	278165.4	5.47	11.45	74.9	5.4	25	3
			PFKADIEM	3	278165.4	5.47	11.44	73.6	4.37	25	2
frizzled 4	22547161	FZD4	FLIDSSRFSYPERPII	2	59881.5	7.05	12.04	82.8	6.07	25	3
			LCSVYVPMCTEKINIPIGPCGGMCLSVKRRCEP	2	59881.5	7.05	13.13	82.9	8.41	25	6
fructose-bisphosphate aldolase C	4885063	ALDOC	GVVPLAGTDGETTTQGLDGLSER	1	39456.1	6.41	20.55	94.3	3.91	25	2
fucosidase, alpha-L-1, tissue	119360348	FUCA1	DGLIVPIFQER	1	87134.2	6.37	18.43	94.7	4.37	25	2
G-gamma globin	6715607	HBG2	LLVVYPWTQR	1	25402.4	6.65	13.55	89.8	8.75	25	2
galactosidase, beta 1 isoform a	119372308	GLB1	SLYPLTFIQVK	3	76075.3	6.1	15.1	85.2	8.31	25	2
			TEAVASSLYDILAR	3	76075.3	6.1	16.11	84.3	4.37	25	2
			TVGAALDILCPSGPIK	3	76075.3	6.1	16.98	93.4	5.5	25	2
galectin 3 binding protein	5031863	LGALS3BP	ASHEEVEGLVEK	6	65331.4	5.13	12.48	73	4.48	25	3
			AVDTWSWGER	6	65331.4	5.13	16.37	84.2	4.37	25	2
			ELSEALGQIFDSQR	6	65331.4	5.13	17.38	90.6	4.14	25	2
			GQWGTVCDNLWDLTDASVVCR	6	65331.4	5.13	11.5	73.1	3.93	25	3
			IDITLSSVK	6	65331.4	5.13	12	71.6	5.84	25	2
			SDLAVPSELALLK	6	65331.4	5.13	15.09	90.5	4.37	25	2
gelsolin isoform a precursor	4504165	GSN	TPSAAYLWVGTGASEAEK	1	85697.9	5.9	16.09	80.3	4.53	25	2
glial fibrillary acidic protein	4503979	GFAP	LALDIEIATYR	1	49880.5	5.42	14.67	90	4.37	25	2
glucosamine (N-acetyl)-6-sulfatase precursor	4504061	GNS	AFQNVFAPR	3	62082.5	8.6	17.17	91.6	9.79	25	2
			IQEPNTFPAILR	3	62082.5	8.6	15.09	81.3	6	25	2
			TQMDGMSLLPILR	3	62082.5	8.6	12.34	80.4	5.5	25	2
glucuronidase, beta	4504223	GUSB	SLDPSRPVTFVSNSNYAADK	2	23356	6.54	21.53	94.4	5.68	25	3
			TLPVGIRTVAVTKSQFLINGK	2	23356	6.54	12.54	81.4	11.17	25	3
glutamate receptor interacting protein 1	103472122	GRIP1	LEILPHHQTRLALK	2	116372.1	5.74	11.57	86.8	8.76	25	2
			SSSLKGKPLSEAIHLLQMAGETVTLKIKKQT	2	116372.1	5.74	13.55	74.1	9.82	25	3
glutaminyl-peptide cyclotransferase precursor	6912618	QPCT	QIAEGTSISEMWQNDLQPLLIER	3	40876.8	6.12	13.5	76.9	4	25	3
			SFSNIISTLNPTAK	3	40876.8	6.12	14.31	85.7	8.47	25	2
			VSPSASAWPEEK	3	40876.8	6.12	16.55	89.6	4.53	25	2
glutathione transferase	4504183	GSTP1	ASCLYGQLPK	2	23356	5.43	18.36	91.7	8.24	25	2
			DQQEAALVDMVNDGVEDLR	2	23356	5.43	17.69	91.5	3.66	25	3
glyceraldehyde-3-phosphate dehydrogenase	7669492	GAPDH	AITATQKTVDGPSGKLWRDGRGA	7	36053.4	8.57	10.05	83.3	9.99	23	3
			GALQNIIPASTGAAK	7	36053.4	8.57	15.96	96.7	8.75	16	3
			IISNASCTTNCLAPLAK	7	36053.4	8.57	11.45	84.6	8.06	15	3
			LISWYDNEFGYSNR	7	36053.4	8.57	15.91	79.5	4.37	25	2
			LVINGNPITIFQER	7	36053.4	8.57	14.69	78.4	6	25	3
			VIISAPSADAPMFVMGVNHEK	7	36053.4	8.57	17.08	92.9	5.32	25	3
			VPTANVSVVDLTCR	7	36053.4	8.57	16.7	85.6	5.8	25	2
GM2 ganglioside activator precursor	39995109	GM2A	IESVLSSSGK	2	20838.5	5.17	16.78	94.8	6	25	2
			SEFVVPDLELPSWLTTGNYR	2	20838.5	5.17	17.64	84	4.14	25	3
GNAS complex locus isoform c	117938759	GNAS	EAIETIVAAMSNLVPPVELANPENQFR	2	111025.1	4.91	13.54	75.9	4.09	25	3
			LLLLGAGESGK	2	111025.1	4.91	15.23	90.3	6	25	2
golgi apparatus protein 1	54633312	GLG1	DAHSQGEVVSCLEK	1	137222.8	6.52	15.8	84.8	4.65	25	3
guanine nucleotide binding protein (G protein) alpha 12	42476111	GNA12	ILLLGAGESGK	1	44279.5	9.84	15.23	90.3	6	25	2
guanine nucleotide binding protein (G protein), alpha 14	4758444	GNA14	LLLLGTGESGK	1	41570.9	5.81	14.7	82.9	6	25	2
guanine nucleotide binding protein (G protein), alpha activating activity polypeptide, olfactory type isoform 1	33695153	GNAL	LLLLGAGESGK	1	52455	6.69	15.23	90.3	6	25	2
guanine nucleotide binding protein (G protein), alpha inhibiting activity polypeptide 2	4504041	GNAI2	FGRSREYQLND	4	40451.1	5.34	10.14	72.1	6.19	25	3
			IAQSDYIPTQQDVLR	4	40451.1	5.34	12.79	88	4.21	18	2
			LFDSICNNK	4	40451.1	5.34	12.44	83.7	5.83	25	2
			LLLLGAGESGK	4	40451.1	5.34	15.23	90.3	6	25	2
guanine nucleotide binding protein (G protein), alpha inhibiting activity polypeptide 3	5729850	GNAI3	LFDSICNNK	2	40532.4	5.51	12.44	83.7	5.83	25	2
			LLLLGAGESGK	2	40532.4	5.51	15.23	90.3	6	25	2
guanine nucleotide binding protein (G protein), beta polypeptide 2-like 1	5174447	GNB2L1	DETNYGIPQR	8	35076.9	7.6	19.72	89.7	4.37	25	2
			DGQAMLWDLNEGK	8	35076.9	7.6	19.37	94.1	4.03	25	2
			DVLSVAFSSDNR	8	35076.9	7.6	12.69	78.6	4.21	25	2
			FSPNSSNPIIVSCGWDK	8	35076.9	7.6	17.99	90.5	5.83	25	2
			LWDLTTGTTTR	8	35076.9	7.6	11.01	76.3	5.84	25	2
			LWNTLGVCK	8	35076.9	7.6	11	75.2	8.22	25	2
			VWNLANCK	8	35076.9	7.6	12.08	86.2	8.19	25	2
			YWLCAATGPSIK	8	35076.9	7.6	16.76	93.2	8.2	25	2
guanine nucleotide binding protein (G protein), gamma 4	4758450	GNG4	EDPLIIPVPASENPFR	1	41570.9	6.55	14.47	86.7	4.14	25	2
guanine nucleotide binding protein (G protein), q polypeptide	40254462	GNAQ	LLLLGTGESGK	1	42142.3	5.48	14.7	82.9	6	25	2
guanine nucleotide binding protein, alpha activating polypeptide O	10567816	GNAO1	AMDTLGIEYGDKER	6	40050.8	5.34	10.88	92.5	4.32	20	3
			IGAADYQPTEQDILR	6	40050.8	5.34	16.22	88.7	4.03	25	2
			IIHEDGFSGEDVK	6	40050.8	5.34	10.61	77.5	4.31	25	3

			LFDSICNNK	6	40050.8	5.34	12.44	83.7	5.83	25	2
			LLLLGAGESGK	6	40050.8	5.34	15.23	90.3	6	25	2
			MEDTEPFSAELLSAMMR	6	40050.8	5.34	12.72	81.4	4	25	2
guanine nucleotide binding protein, alpha transducing 3	156139155	GNAT3	LLLLGAGESGK	1	80110.4	5.69	15.23	90.3	6	25	2
guanine nucleotide binding protein, alpha transducing activity polypeptide 1	22027520	GNAT1	LLLLGAGESGK	1	40041	5.4	15.23	90.3	6	25	2
guanine nucleotide binding protein, alpha transducing activity polypeptide 2	20330805	GNAT2	LLLLGAGESGK	1	40176.1	5.11	15.23	90.3	6	25	2
guanine nucleotide exchange factor p532	126131099	HERC1	GRSAVTRRHKFDLAARTLLA	2	43787.1	5.68	12.16	95.9	12	21	3
			NHVREPTLVTGLQGKN	2	43787.1	5.68	14.99	86.7	8.76	25	3
heat shock 70kDa protein 2	13676857	HSPA2	IINEPTAAAIAYGLDKK	2	50327.2	5.56	13.18	92.3	6.07	25	3
			STAGDTHLGGEDFDNR	2	50327.2	5.56	17.78	91.2	4.22	25	3
heat shock 70kDa protein 5	16507237	HSPA5	DAGTIAGLNVMR	8	72333.3	5.07	17.25	91.6	5.84	22	2
			IINEPTAAAIAYGLDKR	8	72333.3	5.07	15.44	86.4	6.07	25	3
			ITPSYVAFTPEGER	8	72333.3	5.07	19.39	89.7	4.53	25	2
			NELESYAYSLK	8	72333.3	5.07	18.04	91.9	4.53	25	2
			NQLTSNPENTVFDAK	8	72333.3	5.07	11.99	71.4	4.37	25	2
			SQIFSTASDNQPTVTIK	8	72333.3	5.07	14.01	81.8	5.55	25	2
			TFAPEEISAMVLTK	8	72333.3	5.07	16.84	75.1	4.53	25	2
			VTHAVVTVPAYFNDAQR	8	72333.3	5.07	15.67	85	6.71	25	3
heat shock 70kDa protein 6 (HSP70B')	34419635	HSPA6	IINEPTAAAIAYGLDR	1	71028.5	5.81	13.16	75	4.37	25	2
heat shock 70kDa protein 8 isoform 1	5729877	HSPA8	DAGTIAGLNVLR	5	70898.4	5.38	18.06	92.4	5.84	25	2
			IINEPTAAAIAYGLDKK	5	70898.4	5.38	13.18	92.3	6.07	25	3
			NQVAMNPTNTVFDAK	5	70898.4	5.38	15.99	80.2	5.84	25	2
			QTQTFTTYSDNQPGVLIQVYEGER	5	70898.4	5.38	15.65	73.1	4.14	25	3
			STAGDTHLGGEDFDNR	5	70898.4	5.38	17.78	91.2	4.22	25	3
heat shock 90kDa protein 1, beta	20149594	HSP90AB1	ADLINNLGTIAK	9	83264.6	4.97	19.06	87.7	5.88	25	2
			DSGKDKKKKTKKIKEKYIDQEE	9	83264.6	4.97	10.06	80.8	9.4	15	3
			ELISNASDALDK	9	83264.6	4.97	13.94	86.8	4.03	25	2
			EQVANSAFVER	9	83264.6	4.97	12.29	89.9	4.53	25	2
			GVVDSEDLPLNISR	9	83264.6	4.97	17.17	83.4	4.03	25	3
			SIYYITGESK	9	83264.6	4.97	15.17	77.1	5.72	24	2
			TLTLVDTGIGMTK	9	83264.6	4.97	14.13	84.1	5.5	25	2
			YESLTDPSKLDSGK	9	83264.6	4.97	10.32	79.8	4.56	24	3
			YIDQEELNK	9	83264.6	4.97	12.9	85.9	4.14	25	2
heat shock protein 90kDa alpha (cytosolic), class A member 1 isoform 1	153792590	HSP90AA1 HSP9	ADLINNLGTIAK	8	98161.6	5.07	19.06	87.7	5.88	25	2
			DQVANSAFVER	8	98161.6	5.07	16.4	86	4.37	25	2
			ELISNSSDALDK	8	98161.6	5.07	12.19	79.6	4.03	25	2
			GVVDSEDLPLNISR	8	98161.6	5.07	17.17	83.4	4.03	25	3
			NPDDITNEEYGEFYK	8	98161.6	5.07	15.71	80.6	3.84	25	2
			TLTIVDTGIGMTK	8	98161.6	5.07	14.13	84.1	5.5	25	2
			YESLTDPSKLDSGK	8	98161.6	5.07	10.32	79.8	4.56	24	3
			YIDQEELNK	8	98161.6	5.07	12.9	85.9	4.14	25	2
hemopexin	11321561	HPX	GGYTLVSGYPK	3	51676.7	6.55	14.88	86.8	8.5	25	2
			LLQDEFPGIPSPLDAAVECHR	3	51676.7	6.55	14.75	75.5	4.31	25	3
			RDVRDYFMPCPGRGHGHR	3	51676.7	6.55	10.77	80.8	9.5	16	3
hexokinase 1 isoform HKI-ta/tb	15991829	HK1	GAALITAVGVR	4	102380.5	6.31	10.48	94.1	9.75	11	2
			GAAMVTAVAYR	4	102380.5	6.31	18.67	93.3	8.75	25	2
			LALLQVR	4	102380.5	6.31	10	85.2	9.75	18	2
			SANLVAATLGAILNR	4	102380.5	6.31	19.58	93.5	9.47	25	3
hexokinase 2	15553127	HK2	GAAMVTAVAYR	2	102380.5	5.71	18.67	93.3	8.75	25	2
			LALLQVR	2	102380.5	5.71	10	85.2	9.75	18	2
hexosaminidase A preproprotein	4504371	HEXB	ALLSAPWYLNR	4	60689.1	5.04	10.61	71.2	8.79	25	2
			DFYVVEPLAFEGTPEQK	4	60689.1	5.04	17.41	78.5	4	25	2
			EDIPVNYMK	4	60689.1	5.04	16.23	91.3	4.37	18	2
			GLLLDTSR	4	60689.1	5.04	13.35	80.6	5.84	25	2
hexosaminidase B preproprotein	4504373	HEXB	DSAYPEELSR	7	63111.7	6.29	16.4	90.4	4.14	23	2
			GIAAQPLYAGYCNHENM	7	63111.7	6.29	12.52	70.8	5.24	25	2
			GILIDTSR	7	63111.7	6.29	13.35	80.6	5.84	25	2
			LAPGTIVEVWK	7	63111.7	6.29	17.1	95.1	6	21	2
			TLDAMAFNK	7	63111.7	6.29	15.39	96.1	5.5	22	2
			VEPLDFGGTQK	7	63111.7	6.29	14.14	78.4	4.37	25	2
			VLPEFDTPGHTLSWGK	7	63111.7	6.29	15.31	88.5	5.32	20	3
high density lipoprotein binding protein	4885409	HDLBP	LGQALTEVYAK	2	141440.4	6.43	14.26	80	6	25	2
			VATLNSEEESDPPTYK	2	141440.4	6.43	10.64	72.7	4	25	2
HtrA serine peptidase 1	4506141	HTRA1	EVPVASGSGFIVSEDGLIVTNAHVVTNK	5	51287.3	8.09	17.46	91.9	4.65	25	3
			IAPAVVHIELFR	5	51287.3	8.09	14.7	87.4	6.75	21	3
			LPVLLLGR	5	51287.3	8.09	14.21	90.7	9.75	22	2
			VTAGISFAIPSDK	5	51287.3	8.09	11.82	79.4	5.81	25	2
			YNFIADVVEK	5	51287.3	8.09	14.39	86.3	4.37	25	2
HtrA serine peptidase 2 isoform 1 preproprotein	7019477	HTRA2	YNFIADVVEK	1	48841.1	10.07	14.39	86.3	4.37	25	2
HtrA serine peptidase 2 isoform 2	21614538	HTRA2	YNFIADVVEK	1	98309.4	10.72	14.39	86.3	4.37	25	2
hypothetical protein LOC124565 isoform a	83921602	SLC38A10	EKPAPGPGLPPEPREQRDVER	3	119762.9	5.51	14.62	80.9	5.01	25	3
			GKARETVENLPPLPLDPVLR	3	119762.9	5.51	13.88	74.9	6.18	25	3
			VENLPPLPLDPVLRA	3	119762.9	5.51	10.49	74	4.37	20	3
hypothetical protein LOC158358	148612838	KIAA2026	ILLEQSLQSHKKLKLTKMRAKKKKKK	3	228088.5	9.18	12.36	76.3	10.92	25	3
			IVVSASRSPA	3	228088.5	9.18	10.92	77.2	9.75	25	2
			SASASAGAQPPVLVSGNDTSSRIM	3	228088.5	9.18	12.51	85.7	5.55	25	3
hypothetical protein LOC196463	27734917	DDHD2	AFIPGGPSPGSR	1	73451.8	6.53	12.19	93.3	9.79	10	2
hypothetical protein LOC345651	63055057	ACTBL2	DLTDYLMK	2	27559.5	5.39	13.9	91.4	4.21	25	2
			SYELPDGQVITIGNER	2	27559.5	5.39	21.93	88.7	4.14	25	3
hypothetical protein LOC643314	149773456	KIAA0754	LATVPKDI	1	265354.9	4.26	14.18	89.7	5.84	22	2
hypothetical protein LOC79802	124248546	HHIPL2	ISEMKVSRA	1	56233.3	9.21	14.03	87.1	8.75	25	2
iduronate-2-sulfatase isoform a precursor	4557659	IDS	GPDGELHANLLCPVDVLDVPEGTLPDK	1	40076.4	5.21	15.37	74.3	3.96	25	3
inhibitor of kappa light polypeptide gene enhancer in B-cells, kinase complex-associated protein	38569394	IKBKAP	CLRDASFKTLQ	2	150255.1	5.61	11.53	73	8.22	25	2
			HWYLKQSLSFSTCGKSK	2	150255.1	5.61	12.66	87.2	9.63	25	3
insulin-like growth factor 2	4504609	IGF2	FFQYDTWK	2	20140.5	9.5	12.77	86.5	5.83	25	2
			SCDLALLETYCATPAK	2	20140.5	9.5	22.64	100	4.37	25	2
insulin-like growth factor binding protein 2, 36kDa	55925576	IGFBP2	GECWCVNPNTGK	5	35137.8	7.48	18.92	92	5.99	25	2
			LAACGPPPVAPPAAVAAVAGGAR	5	35137.8	7.48	17.09	89.4	8.25	25	2
			LIQGAPTIR	5	35137.8	7.48	12.77	92.6	9.75	25	2
			MPCAELVR	5	35137.8	7.48	18.38	91.3	5.75	25	2
			TPCQQELDQVLER	5	35137.8	7.48	18.93	97.2	4.14	25	2
kinectin 1 isoform a	33620775	KTN1	AAGDTTVIENSDVSPETESSEK	25	156276	5.52	17.89	86.5	3.77	25	3
			ALKEEIGNVQLEK	25	156276	5.52	15.56	82.4	4.79	25	3
			AQLNETLTK	25	156276	5.52	11.87	86.4	6.05	25	2
			AQQSLELIQSK	25	156276	5.52	20.44	93.4	6.05	25	2
			DAVSNTTNQLESK	25	156276	5.52	18.39	90.5	4.37	25	2
			DLLKPDQVEGIQK	25	156276	5.52	15.67	86.9	4.56	25	3
			ETMSVSLNQTVTQLQQLLQAVNQQLTK	25	156276	5.52	10.79	70.6	6.1	25	3
			GELTTLIHQLQEK	25	156276	5.52	12.55	85.3	5.4	25	3
			LLEEQLQHEISNK	25	156276	5.52	14.19	92.2	4.75	25	3
			LMQLMESEQK	25	156276	5.52	13.14	76.5	4.53	25	2

			LQALANEQAAAAHELEK	25	156276	5.52	14.27	89	4.75	25	3
			LQTLVSEQPNKDVVEQMEK	25	156276	5.52	13.11	81.3	4.41	25	3
			NWEAMEALASTEK	25	156276	5.52	13.79	82	4.25	25	2
			QLTQEMMTEK	25	156276	5.52	11.87	81.8	4.53	25	2
			QMQSSFTSSEQELER	25	156276	5.52	13.25	83	4.25	25	2
			QQQVEAVELEAK	25	156276	5.52	14.63	94.7	4.25	25	2
			SVEQEENKWK	25	156276	5.52	13.72	93.1	4.78	21	3
			SVLAETEGILQK	25	156276	5.52	20.8	97.4	4.53	25	2
			TMMFSEDEALCVVDLLK	25	156276	5.52	21.37	95.1	3.91	25	2
			TQLLQDVQDENK	25	156276	5.52	13.36	70.8	4.03	25	2
			TVEELLETGLIQVATK	25	156276	5.52	13.61	91.6	4.25	18	2
			VQELQNLLK	25	156276	5.52	16.91	93	5.97	25	2
			VSVPSNLSYGEWLHGFEK	25	156276	5.52	15.72	81.2	5.4	25	3
			WEEVQSYIR	25	156276	5.52	12.17	75.9	4.53	25	2
			WLQDLQEENESLK	25	156276	5.52	16.53	91.3	4	25	2
kinectin 1 isoform b	118498362	KTN1	AAGDTTVIENSDVSPETESSEK	24	150353.3	5.59	17.89	86.5	3.77	25	3
			AQLNETLTK	24	150353.3	5.59	11.87	86.4	6.05	25	2
			AQQSLELIQSK	24	150353.3	5.59	20.44	93.4	6.05	25	2
			DAVSNTTNQLESK	24	150353.3	5.59	18.39	90.5	4.37	25	2
			DLLKPDQVEGIQK	24	150353.3	5.59	15.67	86.9	4.56	25	3
			ETMSVSLNQTVTQLQQLLQAVNQQLTK	24	150353.3	5.59	10.79	70.6	6.1	25	3
			GELTTLIHQLQEK	24	150353.3	5.59	12.55	85.3	5.4	25	3
			LLEEQLQHEISNK	24	150353.3	5.59	14.19	92.2	4.75	25	3
			LMQLMESEQK	24	150353.3	5.59	13.14	76.5	4.53	25	2
			LQALANEQAAAAHELEK	24	150353.3	5.59	14.27	89	4.75	25	3
			LQTLVSEQPNKDVVEQMEK	24	150353.3	5.59	13.11	81.3	4.41	25	3
			NWEAMEALASTEK	24	150353.3	5.59	13.79	82	4.25	25	2
			QLTQEMMTEK	24	150353.3	5.59	11.87	81.8	4.53	25	2
			QMQSSFTSSEQELER	24	150353.3	5.59	13.25	83	4.25	25	2
			QQQVEAVELEAK	24	150353.3	5.59	14.63	94.7	4.25	25	2
			SVEQEENKWK	24	150353.3	5.59	13.72	93.1	4.78	21	3
			SVLAETEGILQK	24	150353.3	5.59	20.8	97.4	4.53	25	2
			TMMFSEDEALCVVDLLK	24	150353.3	5.59	21.37	95.1	3.91	25	2
			TQLLQDVQDENK	24	150353.3	5.59	13.36	70.8	4.03	25	2
			TVEELLETGLIQVATK	24	150353.3	5.59	13.61	91.6	4.25	18	2
			VQELQNLLK	24	150353.3	5.59	16.91	93	5.97	25	2
			VSVPSNLSYGEWLHGFEK	24	150353.3	5.59	15.72	81.2	5.4	25	3
			WEEVQSYIR	24	150353.3	5.59	12.17	75.9	4.53	25	2
			WLQDLQEENESLK	24	150353.3	5.59	16.53	91.3	4	25	2
kinectin 1 isoform c	118498368	KTN1	AAGDTTVIENSDVSPETESSEK	24	150353.3	5.57	17.89	86.5	3.77	25	3
			ALKEEIGNVQLEK	24	150353.3	5.57	15.56	82.4	4.79	25	3
			AQLNETLTK	24	150353.3	5.57	11.87	86.4	6.05	25	2
			AQQSLELIQSK	24	150353.3	5.57	20.44	93.4	6.05	25	2
			DAVSNTTNQLESK	24	150353.3	5.57	18.39	90.5	4.37	25	2
			DLLKPDQVEGIQK	24	150353.3	5.57	15.67	86.9	4.56	25	3
			ETMSVSLNQTVTQLQQLLQAVNQQLTK	24	150353.3	5.57	10.79	70.6	6.1	25	3
			GELTTLIHQLQEK	24	150353.3	5.57	12.55	85.3	5.4	25	3
			LLEEQLQHEISNK	24	150353.3	5.57	14.19	92.2	4.75	25	3
			LMQLMESEQK	24	150353.3	5.57	13.14	76.5	4.53	25	2
			LQALANEQAAAAHELEK	24	150353.3	5.57	14.27	89	4.75	25	3
			LQTLVSEQPNKDVVEQMEK	24	150353.3	5.57	13.11	81.3	4.41	25	3
			QLTQEMMTEK	24	150353.3	5.57	11.87	81.8	4.53	25	2
			QMQSSFTSSEQELER	24	150353.3	5.57	13.25	83	4.25	25	2
			QQQVEAVELEAK	24	150353.3	5.57	14.63	94.7	4.25	25	2
			SVEQEENKWK	24	150353.3	5.57	13.72	93.1	4.78	21	3
			SVLAETEGILQK	24	150353.3	5.57	20.8	97.4	4.53	25	2
			TMMFSEDEALCVVDLLK	24	150353.3	5.57	21.37	95.1	3.91	25	2
			TQLLQDVQDENK	24	150353.3	5.57	13.36	70.8	4.03	25	2
			TVEELLETGLIQVATK	24	150353.3	5.57	13.61	91.6	4.25	18	2
			VQELQNLLK	24	150353.3	5.57	16.91	93	5.97	25	2
			VSVPSNLSYGEWLHGFEK	24	150353.3	5.57	15.72	81.2	5.4	25	3
			WEEVQSYIR	24	150353.3	5.57	12.17	75.9	4.53	25	2
			WLQDLQEENESLK	24	150353.3	5.57	16.53	91.3	4	25	2
lactate dehydrogenase A	5031857	LDHA	VTLTSEEEAR	1	36688.9	8.44	13.96	87.7	4.25	22	2
laminin, alpha 1 precursor	38788416	LAMA1	LELSELRN	1	337086	5.93	13.5	90.7	4.53	25	2
latent transforming growth factor beta binding protein 4 isoform a	110347431	LTBP4	AEAAAPYTVLAQSAPR	4	58484.4	5.27	16.27	85.8	6.04	25	2
			GGECASPLPGLR	4	58484.4	5.27	12.57	82.8	6	25	2
			GPGAPCQDVDECAR	4	58484.4	5.27	14.33	75.1	4.03	25	2
			SRGPGAPCQDVDECAR	4	58484.4	5.27	20.1	90.9	4.56	25	3
mannosidase, alpha, class 2B, member 1 precursor	51873064	MAN2B1	LEMEQVWR	3	113744.7	6.84	10.06	85.3	4.53	21	2
			LQETTLVANQLR	3	113744.7	6.84	10.35	85.2	6	14	2
			TVDQYFYGIK	3	113744.7	6.84	10.43	76.8	5.5	25	2
mannosidase, beta A, lysosomal	84798622	MANBA	GSNWIPADSFQDR	2	100895.7	5.32	11.79	78.8	4.21	25	2
			LPQSTDPLR	2	100895.7	5.32	11.97	75.9	5.84	25	2
mannosyl (alpha-1,6-)-glycoprotein beta-1,2-N-acetylglucosaminyltransferase	4505163	MGAT2	LTVSCLPKFWKVLVPQIPRIFH	2	20140.5	8.99	13.07	77.4	10.06	25	3
			TVSCLPKFWKVL	2	20140.5	8.99	13.02	72.6	9.3	25	3
mel transforming oncogene	16933567	RAB8A	LLLIGDSGVGK	2	346182.5	9.15	15.93	96.2	5.84	21	2
			TITTAYYR	2	346182.5	9.15	12.9	85.7	8.25	25	2
meningioma 1	55956910	MN1	ASERRPP	3	136002	6.22	10.1	72	9.64	16	2
			ETRGAPTPHEKALTSPS	3	136002	6.22	10.74	72.9	6.86	25	3
			LGMNMEPYGFHARGHS	3	136002	6.22	10.25	72.1	6.92	25	2
microfilament and actin filament cross-linker protein isoform a	33188445	MACF1	KTIVQLKPR	2	670140.2	5.27	10.44	75.4	11.17	25	2
			WLAEVEDKLSSVFVKDFKQDVL	2	670140.2	5.27	13.1	72.9	4.44	25	3
microfilament and actin filament cross-linker protein isoform b	33188443	MACF1	QVENQSAQEKVKV	2	670140.2	5.2	12.26	86.1	6.14	25	3
			WLAEVEDKLSSVFVKDFKQDVL	2	670140.2	5.2	13.1	72.9	4.44	25	3
microtubule-associated protein 1B	153945728	MAP1B	ASLTLFCPEEGDWK	6	270635.7	4.73	13.44	73.9	4.14	25	2
			IKVIKKEGKAAEAVAAAVGTGATTAAVMAAAGIA	6	270635.7	4.73	12.47	73.5	9.53	24	3
			LSMKPEPLFR	6	270635.7	4.73	11.94	82.2	8.75	18	3
			NLISPDLGVVFLNVPENLK	6	270635.7	4.73	17.13	85.2	4.37	25	3
			SVGNTIDPVILFQK	6	270635.7	4.73	12.86	77	5.55	25	2
			VLFPGNSTQYNILEGLEK	6	270635.7	4.73	13.11	75.3	4.53	25	2
microtubule-associated protein 2 isoform 1	87578396	MAP2	MRVDDKDKIEGV	1	199527.2	4.82	13.3	78	4.68	25	2
midkine	4505135	MDK	FENWGACDGGTGTK	2	20140.5	9.84	12.08	89.9	4.37	15	2
			YNAQCQETIR	2	20140.5	9.84	11.59	80.8	5.99	25	2
mitochondrial malate dehydrogenase precursor	21735621	MDH2	EGVVECSFVK	5	35503.5	8.92	10.85	79.8	4.53	25	2
			GCDVVVIPAGVPR	5	35503.5	8.92	11.3	81.3	5.83	21	2
			IQEAGTEVVK	5	35503.5	8.92	11.58	88.1	4.53	25	2
			VAVLGASGGIGQPLSLLLK	5	35503.5	8.92	16.19	80.6	8.72	25	2
			VDFPQDQLTALTGR	5	35503.5	8.92	11.58	84.6	4.21	17	2
mitogen-activated protein kinase 10	21735550	MAP3K10	EEELLRAAQEQRFQEE	3	103695	6.56	12.06	77.5	4.37	25	3
			LSSVSDCNSTRSLLRSDSDE	3	103695	6.56	11.11	74.1	4.46	25	2

			LVDLELESFKKDPRQ	3	103695	6.56	13.86	85.8	4.79	25	3
N-acylsphingosine amidohydrolase (acid ceramidase) 1 isoform b	30089930	ASAH1	DAMWIGFLTR	8	53339.5	7.99	15.14	86.2	5.84	25	2
			ESLDVYELDAK	8	53339.5	7.99	13.23	87.4	3.91	25	2
			GAVPWYTINLDLPPYK	8	53339.5	7.99	15.08	78.7	5.83	25	2
			GQFETYLR	8	53339.5	7.99	16.36	89.1	6	25	2
			LPGLLGNFPGPFEEEMK	8	53339.5	7.99	10.44	79.8	4.25	15	3
			NMINTFVPSGK	8	53339.5	7.99	15.76	91	8.75	25	2
			STYPPSGPTYR	8	53339.5	7.99	15.36	95.5	8.31	23	2
			TSQENISFETMYDVLSTKPVLNK	8	53339.5	7.99	13.48	70.8	4.68	25	3
N-ethylmaleimide-sensitive factor attachment protein, alpha	47933379	NAPA	IEEACEIYAR	2	42274.4	5.23	13.8	91.5	4.25	19	2
			YEELFPAFSDSR	2	42274.4	5.23	12.05	81.6	4.14	17	2
N-ethylmaleimide-sensitive factor	156564401	NSF	VLDDGELLVQQTK	1	82594.8	6.52	12.45	76.2	4.03	25	2
NAD(P)H:quinone oxidoreductase type 3, polypeptide A2	49574502	CYB5R1	GPSGLLTYTGK	2	42274.4	9.41	15.08	81.9	8.59	25	2
			VGDVVEFR	2	42274.4	9.41	10.87	74.1	4.37	21	2
nephrocystin isoform 1	46397398	NPHP1	LPLLHSTR	2	57136.5	5.11	13.27	94.9	9.75	21	2
			LSQLLEEGNQFRA	2	57136.5	5.11	11.15	80.3	4.53	25	2
neuroblastoma RAS viral (v-ras) oncogene homolog	4505451	NRAS	SYGIPFIETSAK	3	21229.3	5.01	10.36	77.7	5.72	25	2
			YDPTIEDSYR	3	21229.3	5.01	16.37	94.4	4.03	25	2
			YDPTIEDSYRK	3	21229.3	5.01	10.37	71.7	4.56	25	3
neurofibromin isoform 1	109826564	NF1	GQLLETSGLCIPANNTLFIVSISK	3	319374.2	7.1	10.18	75.2	5.99	25	3
			HTCREGN	3	319374.2	7.1	11.81	71.7	6.74	25	2
			RGNSLASKIMT	3	319374.2	7.1	12.04	79.7	11	25	2
neurofibromin isoform 2	4557793	NF1	GQLLETSGLCIPANNTLFIVSISK	3	317034.4	6.9	10.18	75.2	5.99	25	3
			HTCREGN	3	317034.4	6.9	11.81	71.7	6.74	25	2
			RGNSLASKIMT	3	317034.4	6.9	12.04	79.7	11	25	2
nidogen 1 precursor	115298674	NID1	LPLEGNTMR	5	136377.8	5.12	10.82	82.7	6	25	2
			SRDPDQKGKRNTFQAVLAS	5	136377.8	5.12	10.97	79.7	9.98	22	3
			VAKLDGTQRRVL	5	136377.8	5.12	11.91	71.8	10.83	25	3
			VLFETDLVNPR	5	136377.8	5.12	13.14	82	4.37	25	2
			YALSNSIGPVR	5	136377.8	5.12	16.1	87.4	8.75	25	2
oxygen regulated protein precursor	5453832	HYOU1	AEAGPEGVAPAPEGEKK	4	16570.3	5.16	14.43	93.1	4.49	25	3
			LPATEKPVLLSK	4	16570.3	5.16	17.05	85.3	8.59	25	3
			TLGGLEMELR	4	16570.3	5.16	13.51	77.9	4.53	25	2
			VAIVKPGVPMEIVLNK	4	16570.3	5.16	10.9	71.8	8.56	21	3
palmitoyl-protein thioesterase 1 (ceroid-lipofuscinosis, neuronal 1, infantile)	4506031	PPT1	ETIPLQETSLYTQDR	2	34193.7	6.07	13.89	80.7	4.14	25	2
			LQQGYNAMGFSQGGQFLR	2	34193.7	6.07	12.32	75.4	8.75	25	3
peptidylglycine alpha-amidating monooxygenase isoform a, preproprotein	21070984	PAM	ANILYAWAR	9	96258.2	5.98	18.28	92.5	8.79	25	2
			DCSGVSLHLTR	9	96258.2	5.98	11.8	83.1	6.73	18	2
			GSGGLNLGNFFASR	9	96258.2	5.98	12.04	76.5	9.75	25	2
			IPLLQQPK	9	96258.2	5.98	14.16	95	8.75	20	2
			IPVDEEAFVIDFKPR	9	96258.2	5.98	10.31	70.1	4.32	21	3
			NGQWTLIGR	9	96258.2	5.98	14.03	80.3	9.75	25	2
			QSPQLPQAFYPVGHPVDVSFGDLLAAR	9	96258.2	5.98	21.01	91.3	5.21	25	3
			TIPPEANIPIPVK	9	96258.2	5.98	14.93	90.1	5.66	25	2
			VVNSDISCHYK	9	96258.2	5.98	10.61	72.7	6.7	25	2
peptidylglycine alpha-amidating monooxygenase isoform c, preproprotein	21070980	PAM	ANILYAWAR	9	96258.2	6	18.28	92.5	8.79	25	2
			DCSGVSLHLTR	9	96258.2	6	11.8	83.1	6.73	18	2
			GSGGLNLGNFFASR	9	96258.2	6	12.04	76.5	9.75	25	2
			IPLLQQPK	9	96258.2	6	14.16	95	8.75	20	2
			IPVDEEAFVIDFKPR	9	96258.2	6	10.31	70.1	4.32	21	3
			NGQWTLIGR	9	96258.2	6	14.03	80.3	9.75	25	2
			QSPQLPQAFYPVGHPVDVSFGDLLAAR	9	96258.2	6	21.01	91.3	5.21	25	3
			TIPPEANIPIPVK	9	96258.2	6	14.93	90.1	5.66	25	2
			VVNSDISCHYK	9	96258.2	6	10.61	72.7	6.7	25	2
peptidylprolyl isomerase A (cyclophilin A)-like 4	30102944	PPIAL4G	IIPGFMCQGGDFTR	1	18181.9	9.32	12.75	82	5.83	25	2
peptidylprolyl isomerase B precursor	4758950	PPIB	DFMIQGGDFTR	4	23742.7	9.42	12.6	84.6	4.21	25	2
			DTNGSQFFITTVK	4	23742.7	9.42	11.5	74	5.84	25	2
			TVDNFVALATGEK	4	23742.7	9.42	12.07	77.3	4.37	25	2
			VLEGMEVVR	4	23742.7	9.42	12.02	83.6	4.53	25	2
periaxin isoform 2	116686120	PRX	EPRPAAPEVVESKLKL	2	154905.5	7.22	15.9	91	6.33	25	3
			MPKMTMPKLGRAESPSRGKP	2	154905.5	7.22	17.01	86.6	11.1	25	4
peroxiredoxin 1	4505591	PRDX1	ADEGISFR	3	21229.3	8.27	13.24	84.7	4.37	22	2
			GLFIIDDK	3	21229.3	8.27	10.01	72.7	4.21	20	2
			QITVNDLPVGR	3	21229.3	8.27	11.34	73	5.84	25	2
peroxiredoxin 2 isoform a	32189392	PRDX2	ATAVVDGAFK	5	21892	5.66	12.39	88.8	5.88	23	2
			EGGLGPLNIPLLADVTR	5	21892	5.66	17.9	89.4	4.37	25	2
			GLFIIDGK	5	21892	5.66	11.42	81.8	5.84	25	2
			LSEDYGVLK	5	21892	5.66	12.69	85.1	4.37	24	2
			QITVNDLPVGR	5	21892	5.66	11.34	73	5.84	25	2
peroxiredoxin 2 isoform c	33188454	PRDX2	ATAVVDGAFK	1	15818.6	9.14	12.39	88.8	5.88	23	2
phosphodiesterase 1C, calmodulin-dependent 70kDa	4826894	PDE1C	CDRKSTMVAQSQVGFID	2	72208.7	6.19	14.33	86.4	6.13	25	3
			YKTGVANWLTELE	2	72208.7	6.19	12.63	82.7	4.86	25	3
phosphoglycerate kinase 1	4505763	PGK1	ACANPAAGSVILLENLR	3	44615	8.3	15.15	94.3	6.04	19	3
			AHSSMVGVNLPQK	3	44615	8.3	12.36	85.4	8.8	17	3
			ALESPERPFLAILGGAK	3	44615	8.3	10.45	96.6	6.19	11	3
phospholipase C beta 4 isoform a	33469933	PLCB4	AMGIETSDIADVPSDTSK	1	136136.9	6.37	12.17	70.3	3.84	25	2
phosphoribosyl pyrophosphate amidotransferase proprotein	29570798	PPAT	IPVSDINDK	1	53339.5	6.3	13.86	71.7	4.21	25	2
plasma glutamate carboxypeptidase	7706387	PGCP	AIINLAVYGK	9	51887.9	5.79	16.72	92.9	8.63	25	2
			AIQIMYQNLQQDGLEK	9	51887.9	5.79	22.51	100	4.37	25	3
			GEESAVMLEPR	9	51887.9	5.79	17	93.2	4.25	25	2
			IVVYNQPYINYSR	9	51887.9	5.79	15.43	83	8.5	25	2
			LALLVDTVGPR	9	51887.9	5.79	16.94	92.9	5.84	25	2
			SIYSPHTGIQEYQDGVPK	9	51887.9	5.79	12	79.8	5.3	25	3
			SVASFSIYSPHTGIQEYQDGVPK	9	51887.9	5.79	15.45	92	5.3	25	3
			TYPDTDSFNTVAEITGSK	9	51887.9	5.79	17.39	90.7	4.03	25	2
			VGALASLIR	9	51887.9	5.79	18.21	91.8	9.72	25	2
plasma glutathione peroxidase 3 precursor	6006001	GPX3	NSCPPTSELLGTSDR	2	25402.4	8.26	13.39	70.9	4.37	25	2
			QEPGENSEILPTLK	2	25402.4	8.26	15.95	88.2	4.25	25	2
plasminogen activator inhibitor type 1, member 2	24307907	SERPINE2	ASAATTAILIAR	10	44002.5	9.35	14.21	84.1	9.79	25	2
			DIVTVANAVFVK	10	44002.5	9.35	20.62	95.6	5.84	25	2
			DMIDNLLSPDLIDGVLTR	10	44002.5	9.35	18.82	91.9	3.77	25	3
			DVFQCEVR	10	44002.5	9.35	17.46	90.4	4.37	25	2
			FTAVAQTDLK	10	44002.5	9.35	12.47	89.8	5.84	18	2
			FTAVAQTDLKEPLK	10	44002.5	9.35	16.84	90.1	6.07	25	3
			LAQLSVFR	10	44002.5	9.35	13.55	83.1	9.75	25	2
			NKDVFQCEVR	10	44002.5	9.35	11.17	74.3	6.06	25	3
			SYQVPMLAQLSVFR	10	44002.5	9.35	12.41	75.7	8.46	25	2
			VLGITDMFDSSK	10	44002.5	9.35	16.21	90.1	4.21	25	2
plasminogen activator, tissue type isoform 1 preproprotein	4505861	PLAT	ATCYEDQGISYR	5	62917.5	8.14	16.33	87	4.37	25	2
			LGLGNHNYCR	5	62917.5	8.14	11.01	75.7	8.23	25	3
			TVTDNMLCAGDTR	5	62917.5	8.14	15.87	91.4	4.21	22	2

			VTNYLDWIR	5	62917.5	8.14	13.04	73.2	5.8	25	2
			VYTAQNPSAQALGLGK	5	62917.5	8.14	19.13	89.5	8.56	25	2
PREDICTED: similar to B-cell receptor-associated protein 31 (BCR-associated protein Bap31) (p28 Bap31)	113426081	BCAP31	AENQVLAMR	1	77340.8	8.1	19.25	88.6	6.05	25	2
PREDICTED: similar to CG9007-PA isoform 17	88961345	SETD5	EEGGSNSLVTPTEAGSLDSSGENR	2	95002.8	8.67	13.05	74.5	3.91	25	3
			GKPTILDTIN	2	95002.8	8.67	14.12	80.3	5.84	25	2
PREDICTED: similar to Collagen alpha-1(I) chain precursor	113424259	COL8A1	ACDPPGTRDPLGTRDPPGTRDPP	3	77340.8	8.85	10.98	75.3	4.58	21	3
			TRDPPGARDPPGTRDPP	3	77340.8	8.85	13.37	92.2	5.71	22	3
			TRDPPGTRDPPGARDPP	3	77340.8	8.85	14.36	89.1	5.71	20	3
PREDICTED: similar to Glutamate receptor-interacting protein 1 (GRIP1 protein)	113423398	GRIP1	LEILPHHQTRLALK	2	34588.4	5.99	11.57	86.8	8.76	25	2
			SSSLKGKPLSEAIHLLQMAGETVTLKIKKQT	2	34588.4	5.99	13.55	74.1	9.82	25	3
PREDICTED: similar to Guanine nucleotide-binding protein beta subunit 2-like 1 (Receptor of activated protein kinase C 1) (RACK1)	88984860	GNB2L1	DGQAMLWDLNEGK	2	17623.4	6.39	19.37	94.1	4.03	25	2
			VWNLANCK	2	17623.4	6.39	12.08	86.2	8.19	25	2
PREDICTED: similar to Guanine nucleotide-binding protein G(t), alpha-3 subunit (Gustducin alpha-3 chain)	113419695	GNAT3	LLLLGAGESGK	1	72643.2	9.75	15.23	90.3	6	25	2
PREDICTED: similar to loss of heterozygosity, 11, chromosomal region 2	88966020	VWA5B2	ALGDPATPTEGPR	8	136258.8	6.08	17.71	89.8	4.37	25	2
			ALGPGLGTPTPR	8	136258.8	6.08	14.19	85.6	9.79	25	2
			DSEQSTDALTDPVTDPGPNPSDTAIWR	8	136258.8	6.08	13.23	73.5	3.61	25	3
			EIPALYPGDQLLGYCSLFR	8	136258.8	6.08	13.32	78.7	4.37	22	2
			IFQSSYIR	8	136258.8	6.08	11.27	88.9	8.75	25	2
			SCPLPAPTPAPFK	8	136258.8	6.08	15.78	87.5	7.94	25	2
			SLAWGEPAGSR	8	136258.8	6.08	15.97	90.2	5.72	25	2
			VSSAPSCFTCPVAVDATTR	8	136258.8	6.08	17.29	87.7	5.79	25	2
PREDICTED: similar to Metalloproteinase inhibitor 2 precursor (TIMP-2) (Tissue inhibitor of metalloproteinases 2) (CSC-21K)	113427619	TIMP2	EVDSGNDIYGNPIK	3	29067.8	6.17	12.66	74.8	4.03	24	2
			EVDSGNDIYGNPIKR	3	29067.8	6.17	12.4	72.5	4.56	25	3
			GAAPPKQEFLDIEDP	3	29067.8	6.17	10.43	72.9	3.91	19	2
PREDICTED: similar to odd Oz/ten-m homolog 2 isoform 5	113417289	ODZ2	FNISLGKDALFGVYIRRGLPPSH	2	289131	6.23	12.02	85.9	9.99	25	3
			TQRDYDVL	2	289131	6.23	13.15	71.2	4.21	25	2
PREDICTED: similar to Prostate, ovary, testis expressed protein on chromosome 2 isoform 2	88953571	A26C1A	AVFPSIVGR	2	95002.8	5.83	17.13	88	9.79	25	2
			QEYDESGPSIVHR	2	95002.8	5.83	14.36	87.9	4.65	23	2
PREDICTED: similar to Triosephosphate isomerase (TIM) (Triose-phosphate isomerase) isoform 1	113408766	TPI1	IAVAAQNCYK	3	27110.2	8.82	14.33	87.5	8.2	22	2
			IIYGGSVTGATCK	3	27110.2	8.82	19.48	94.5	8.2	25	2
			SNVSDAVAQSTR	3	27110.2	8.82	21.3	93.8	5.55	25	2
PREDICTED: similar to Ubiquitin-63E CG11624-PA, isoform A	113423966	UBC	IQDKEGIPPDQQR	3	77340.8	8.93	18.95	90.7	4.56	25	3
			TITLEVEPSDTIENVK	3	77340.8	8.93	13.6	79.6	4	25	2
			TLSDYNIQK	3	77340.8	8.93	15.82	93	5.5	25	2
procollagen C-endopeptidase enhancer	4505643	PCOLCE	AQGTLTTPNWPESDYPPGISCSWH	8	47946.7	7.41	15.04	74	4.35	25	3
			FCGTFRPAPLVAPGNQVTLR	8	47946.7	7.41	16.92	83.2	10.35	25	3
			FDLEPDTYCR	8	47946.7	7.41	15.36	90.5	4.03	25	2
			PAPLVAPGNQVTLR	8	47946.7	7.41	15.59	85.4	10.18	25	2
			SQPPEKTEESPSAPDAPTCPK	8	47946.7	7.41	20.62	89.3	4.41	25	3
			TEESPSAPDAPTCPK	8	47946.7	7.41	14.79	90.1	4.14	25	2
			VFDLELHPACR	8	47946.7	7.41	11.15	88.4	5.32	25	3
			YDALEVFAGSGTSGQR	8	47946.7	7.41	21.91	97.5	4.37	25	2
proenkephalin	5453876	PENK	DAEEDDSLANSSDLLK	1	30787.2	5.44	21.74	92.3	3.66	25	2
prolyl 4-hydroxylase, beta subunit precursor	20070125	P4HB	DAPEEEDHVLVLR	4	57116.6	4.76	15.9	84.5	4.17	25	3
			EADDIVNWLK	4	57116.6	4.76	10.77	83.1	4.03	25	2
			QFLQAAEAIDDIPFGITSNSDVFSK	4	57116.6	4.76	14.95	74.6	3.84	25	3
			VDATEESDLAQQYGVR	4	57116.6	4.76	15.28	87.7	3.91	25	2
prolylcarboxypeptidase isoform 2 preproprotein	117306169	PRCP	CTNGVDDMFEPHSWNLK	5	58099.9	7	15.87	82.3	4.54	25	3
			ELSDDCFQQWGVRPR	5	58099.9	7	15.35	86.5	4.56	25	3
			NALDPMSVLLAR	5	58099.9	7	17.42	89.7	5.84	25	2
			PSWITTMYGGK	5	58099.9	7	13	88	9	25	2
			YYGESLPFGDNSFK	5	58099.9	7	14.01	82.8	4.37	25	2
proprotein convertase subtilisin/kexin type 1 inhibitor precursor	7019519	PCSK1N	ALAHLLEAER	9	27372.5	6.22	16.03	87.1	5.4	25	2
			ILAGSADSEGVAAPR	9	27372.5	6.22	10.66	73.4	4.37	25	2
			LDPAALAAQLVPAPVPAAALR	9	27372.5	6.22	15.85	87.5	5.84	25	2
			NSDPALGLDDDPDAPAAQLAR	9	27372.5	6.22	20.93	92.3	3.66	25	3
			PAALAAQLVPAPVPAAALR	9	27372.5	6.22	11.35	84	10.18	20	2
			PALGLDDDPDAPAAQLAR	9	27372.5	6.22	21.33	97.7	3.77	25	2
			PDAPAAQLAR	9	27372.5	6.22	12.6	84.7	6.27	25	2
			PRPPVYDDGPAGPDAEEAGDETPDVDPELLR	9	27372.5	6.22	21.38	90.6	3.69	25	3
			VLAQLLR	9	27372.5	6.22	13.71	94.2	9.72	22	2
proprotein convertase subtilisin/kexin type 1 preproprotein	20336242	PCSK1	ALVDLADPR	4	84152	5.66	14.3	84.1	4.21	25	2
			FGFGLLNAK	4	84152	5.66	13.36	88	8.75	20	2
			LDLHVIPVWQK	4	84152	5.66	16.58	75.1	6.74	25	3
			LNIPYENFYEALEK	4	84152	5.66	12.59	72.1	4.25	25	2
proprotein convertase subtilisin/kexin type 2	20336244	PCSK2	DINEIDINMNDPLFTK	11	70565.6	6.04	22.37	92.5	3.84	25	2
			DMQHLTVLTSK	11	70565.6	6.04	12.18	77.1	6.74	25	3
			ELTLQAMADGVNK	11	70565.6	6.04	16.51	88.5	4.37	25	2
			EWTLMLHGTQSAPYIDQVVR	11	70565.6	6.04	14.19	78.1	5.32	25	3
			FHCVGGSVQDPEK	11	70565.6	6.04	11.21	78.1	5.32	25	2
			FHCVGGSVQDPEKIPSTGK	11	70565.6	6.04	12.78	76.7	6.74	25	3
			KEELEEELDEAVER	11	70565.6	6.04	13.09	77.9	3.99	25	3
			LVLTLTTDACEGK	11	70565.6	6.04	15.04	91	4.37	25	2
			NQLHDEVHQWR	11	70565.6	6.04	13.62	78.4	5.99	25	3
			VGFDKWPF	11	70565.6	6.04	14.7	83.9	5.81	25	2
			YTDDWFNSHGTR	11	70565.6	6.04	11.14	76.4	5.21	25	3
prosaposin isoform b preproprotein	110224476	PSAP	EIVDSYLPVILDIIK	2	58484.4	5.03	18.65	89.5	4.03	25	2
			LPALTVHVTQPK	2	58484.4	5.03	17.1	94.7	8.76	20	3
protease, serine, 1 preproprotein	4506145	PRSS1	VLEGNEQFIN	2	26558.2	6.08	10.87	76.2	3.8	25	2
			YVKWIKNTIAAN	2	26558.2	6.08	13.6	71.2	9.7	25	2
protein disulfide isomerase-associated 3 precursor	21361657	PDIA3	ELSDFISYLQR	3	56782.7	5.99	11.52	76.5	4.37	25	2
			GFPTIYFSPANK	3	56782.7	5.99	10.44	76.5	8.59	25	2
			LAPEYEAAATR	3	56782.7	5.99	17.37	91.7	4.53	25	2
protein tyrosine phosphatase, receptor type, N polypeptide 2 isoform 1 precursor	11386149	COX15	EDPSSTGDGARIHTLLKD	2	51676.7	5.55	12.99	81.8	4.83	25	3
			LLQVPSSAFADVEVLGPAVTFK	2	51676.7	5.55	15.35	92.1	4.37	16	2
protein tyrosine phosphatase, receptor type, N precursor	4506321	PTPRN	AEAPALFSR	3	105848.1	6.66	15.1	87.4	6.05	25	2
			EEAAAVLPQTAH	3	105848.1	6.66	11.17	81.2	4.51	25	2
			GEKPASPAVQPDAALQR	3	105848.1	6.66	18.52	87.4	6.07	25	3
proteolipid protein 2 (colonic epithelium-enriched)	4505893	PLP2	HTAAPTDPADGPV	1	16690.8	6.8	20.94	92.4	4.2	25	2
pyruvate kinase 3 isoform 1	33286418	PKM2	AEGSDVANAVLDGADCIMLSGETAK	6	57937.2	7.95	11.8	72.4	3.77	25	3
			ITLDNAYMEK	6	57937.2	7.95	12.05	81.2	4.37	25	2
			LDIDSPPITAR	6	57937.2	7.95	12.96	71	4.21	25	2
			NTGIICTIGPASR	6	57937.2	7.95	14.3	73.4	8.25	25	2
			TATESFASDPILYRPVAVALDTK	6	57937.2	7.95	13.71	80.9	4.56	25	3
			TLDNAYMEK	6	57937.2	7.95	12.76	85	4.37	25	2
quinoid dihydropteridine reductase	4506359	QDPR	AALDGTPGMIGYGMAK	2	25803.7	6.9	15.83	86.4	5.88	25	2
			NSGMPPGAAAIAVLPVTLDTPMNR	2	25803.7	6.9	13.36	72	5.84	25	3
rabphilin 3A homolog	45267837	RPH3A	MSHPSGPYSQASAAAPQPAAAR	2	143742	8.75	14.04	79.1	8.52	25	3
			SNDYIGGCQLGISAK	2	143742	8.75	10.6	76.3	5.55	25	2
raptor	22094987	RPTOR	PRDLFQKLFRQDLLVASLFR	2	149038.5	6.43	14.84	78.4	10.74	25	3
			SSLNSLIGVSFNSVYTQIWRVL	2	149038.5	6.43	13	71.4	8.46	25	3

ras-like protein TC10	50263042	RHOQ	CVVVGDGAVGK	2	42274.4	5.94	11.44	78.1	5.83	25	2
			YVECSALTQK	2	42274.4	5.94	14.81	86.8	5.99	25	2
ras-related C3 botulinum toxin substrate 1 isoform Rac1b	9845509	RAC1	AVLCPPPVK	4	23467.5	8.87	13.34	74.8	8.27	25	2
			CVVVGDGAVGK	4	23467.5	8.87	11.44	78.1	5.83	25	2
			LTPITYPQGLAMAK	4	23467.5	8.87	17.22	89.8	8.59	25	2
			YLECSALTQR	4	23467.5	8.87	13.04	82.6	5.99	25	2
ras-related GTP-binding protein RAB10	33695095	RAB10	AFLTLAEDILR	2	22469	8.8	15	85.2	4.37	21	2
			LLLIGDSGVGK	2	22469	8.8	15.93	96.2	5.84	21	2
Ras-related protein Rab-11A	4758984	RAB11A	AQIWDTAGQER	3	24393.6	6.12	20.97	95	4.37	25	2
			NEFNLESK	3	24393.6	6.12	11.89	82.1	4.53	25	2
			VVLIGDSGVGK	3	24393.6	6.12	12.19	83.6	5.81	25	2
Ras-related protein Rab-27A	19923264	RAB27A	FLALGDSGVGK	2	24868.2	5.09	14.92	91.6	5.84	25	2
			TSVLYQYTDGK	2	24868.2	5.09	18.06	95	5.5	25	2
receptor accessory protein 5	115430112	REEP5	NCMTDLLAK	1	21493.3	8.26	14.44	83.8	5.83	25	2
reticulon 4 isoform A	24431935	RTN4	GPLPAAPPVAPER	1	236447	4.43	20.76	89.4	6	25	2
reticulon 4 isoform D	47519490	RTN4	GPLPAAPPVAPER	1	42274.4	4.66	20.76	89.4	6	25	2
scribble isoform a	45827731	SCRIB	KVSPTGAAGRDGRLRVGLRLLEVNQQS	2	57136.5	5.03	13.27	87.8	11.54	25	3
			VRVPQAEGPPKRVSLVGADDLRKMQE	2	57136.5	5.03	12.19	85.8	8.56	25	3
secreted modular calcium-binding protein 1 isoform 1	78190498	SMOC1	FSEPDPSHTLEER	2	48262.2	8.58	16.85	89.5	4.4	25	3
			YVMPSCESDAR	2	48262.2	8.58	13.19	81	4.37	21	2
secretogranin II precursor	68160947	SCG2	AAWIPHVENR	19	70941.1	4.67	14.97	91.5	6.79	22	2
			ALEYIENLR	19	70941.1	4.67	20.67	96	4.53	25	2
			ANNIAYEDVVGGEDWNPVEEK	19	70941.1	4.67	18.03	87.7	3.77	25	3
			DQELGEYLAR	19	70941.1	4.67	14.94	79.5	4.14	22	2
			DSLSEEDWMR	19	70941.1	4.67	12.22	76	3.91	25	2
			ELDLPVDLDDISEADLDHPDLFQNR	19	70941.1	4.67	18.89	90	3.69	25	3
			HMQFPPMYEENSR	19	70941.1	4.67	11.73	74.1	5.4	25	3
			IILEALR	19	70941.1	4.67	11.41	100	6	11	2
			LYTDDEDDIYK	19	70941.1	4.67	15.06	79	3.71	25	2
			NLQIPPEDLIEMLK	19	70941.1	4.67	21.93	91.5	4.14	25	3
			NQLLQKEPDLR	19	70941.1	4.67	15.25	74.6	6.07	25	3
			QMAYENLNDK	19	70941.1	4.67	10.98	83.6	4.37	25	2
			SGQLGIQEEDLR	19	70941.1	4.67	14.22	86.5	4.14	25	2
			SGQLGIQEEDLRK	19	70941.1	4.67	14.62	80.8	4.68	25	3
			TNEIVEEQYTPQSLATLESVFQELGK	19	70941.1	4.67	17.17	87.3	3.98	25	3
			TSYFPNPYNQEK	19	70941.1	4.67	17.67	93.8	5.66	25	2
			WIPHVENR	19	70941.1	4.67	11.08	73.3	6.75	25	2
			YPEIINSNQV	19	70941.1	4.67	11.5	81.9	4	25	2
			YPEIINSNQVK	19	70941.1	4.67	14.04	84.9	6	25	2
secretogranin III	19557645	SCG3	ELSAERPLNEQIAEAEEDK	7	53005.6	4.94	15.46	90.7	4.06	22	3
			GILDKEEAEAIKR	7	53005.6	4.94	12.69	82	4.87	17	3
			GSLKDSTKDDNSNPGGKTDEPK	7	53005.6	4.94	10.26	86.3	4.78	17	3
			LNVEDVDSTK	7	53005.6	4.94	11.88	86.6	4.03	21	2
			TYSEDNFEELQYFPNFYALLK	7	53005.6	4.94	12.25	72.1	4	25	3
			VTPMAAIQDGLAK	7	53005.6	4.94	15.68	87.4	5.81	25	2
			YGTISPEEGVSYLENLDEMIALQTK	7	53005.6	4.94	14.61	82.3	3.9	25	3
secretory granule, neuroendocrine protein 1 (7B2 protein)	4506917	SCG5	DFSEDQGYPDPPNPCPVGK	3	23658.7	5.62	21.59	95.8	3.84	25	3
			IVAELTGDNIPK	3	23658.7	5.62	16.24	84.2	4.37	25	2
			VEYPAHQAMN	3	23658.7	5.62	11.31	85.9	5.24	15	2
septin 11	8922712	10-Sep	DTDPDSKPFSLQETYEAK	3	49398.5	6.36	12.11	80.8	4.11	22	3
			FESDPATHNEPGVR	3	49398.5	6.36	17.15	93.5	4.65	25	3
			STLMDTLFNTK	3	49398.5	6.36	14.57	85	5.55	25	2
septin 2	4758158	1-Sep	ASIPFSVVGSNQLIEAK	5	41487.7	6.15	14.29	78.6	6.05	25	2
			KVENEDMNKDQILLEK	5	41487.7	6.15	11.32	76	4.51	25	3
			LTVVDTPGYGDAINCR	5	41487.7	6.15	17.73	87.8	4.21	25	2
			LYPWGVVEVENPEHNDFLK	5	41487.7	6.15	12.36	76.6	4.4	22	3
			STLINSLFLTDLYPER	5	41487.7	6.15	15.68	80.3	4.37	25	2
serine (or cysteine) proteinase inhibitor, clade A (alpha-1 antiproteinase, antitrypsin), member 1	50363217	SERPINA1	ITPNLAEFAFSLYR	3	46736.8	5.37	10.58	72	6	25	2
			LSITGTYDLK	3	46736.8	5.37	12.95	82.3	5.83	24	2
			SVLGQLGITK	3	46736.8	5.37	15.06	93	8.47	22	2
serine (or cysteine) proteinase inhibitor, clade I (neuroserpin), member 1	4826904	SERPINI1	AQLVEEWANSVK	2	46427.2	4.84	11.27	79.3	4.53	23	2
			QEVPLATLEPLVK	2	46427.2	4.84	14.99	79.9	4.53	25	2
sparc/osteonectin, cwcv and kazal-like domains proteoglycan 1 precursor	4759164	SPOCK1	SLLGAFIPR	1	49124.6	5.74	12.61	93.2	9.47	17	2
spectrin beta isoform a	67782321	SPTB	LLSGEDVGQDEGATR	1	77028.9	5.25	14.56	80	3.91	25	2
spectrin, alpha, non-erythrocytic 1 (alpha-fodrin)	154759259	SPTAN1	ALINADELASDVAGAEALLDR	8	270635.7	5.22	12.1	73.7	3.77	25	3
			DLSSVQTLLTK	8	270635.7	5.22	14.06	72.6	5.84	25	2
			DVTGAEALLER	8	270635.7	5.22	13.6	83.7	4.14	22	2
			IAALQAFADQLIAAGHYAK	8	270635.7	5.22	10.82	70.4	6.74	25	3
			LSDDNTIGKEEIQQR	8	270635.7	5.22	13.29	79.2	4.32	25	3
			QEAFLLNEDLGDSLDSVEALLK	8	270635.7	5.22	12.13	73	3.71	25	3
			SQLLGSAHEVQR	8	270635.7	5.22	10.36	86.6	6.47	18	3
			VLETAEDIQER	8	270635.7	5.22	14.25	89	4	25	2
spectrin, beta, non-erythrocytic 1 isoform 1	112382250	SPTBN1	ADLDKWL	1	274610.6	5.39	14.01	86	4.21	25	2
stanniocalcin 1 precursor	4507265	STC1	SLLECDEDTVSTIR	1	27621.6	8.28	14.62	83.2	3.91	25	2
stratifin	5454052	SFN	DSTLIMQLLR	1	30787.2	4.68	12.86	91	5.84	17	2
suppression of tumorigenicity 5 isoform 1	47132529	ST5	GIREKISAWEGRREASPRM	2	126485.3	9.35	12.28	74.7	10.67	25	3
			LSRSQSVSPPPVLSPPRSPIYP	2	126485.3	9.35	12.19	72.2	10.84	25	3
surfactant, pulmonary-associated protein B	33943099	SFTPB	KPLRDPLPDPLLDKL	2	42117.5	5.27	14.31	70.5	6.04	25	3
			LLDKLVLPVLPGALQAR	2	42117.5	5.27	14.71	82.5	8.75	25	3
synaptotagmin I	5032139	SYT1	HDIIGEFK	6	47573.4	8.26	10.27	71.2	5.32	25	2
			LGDICFSLR	6	47573.4	8.26	14.44	90.4	5.83	25	2
			NTLNPYYNESFSFEVPFEQIQK	6	47573.4	8.26	13.54	70.3	4.25	25	3
			TLVMAVYDFDR	6	47573.4	8.26	23.77	98.1	4.21	25	2
			VFVGYNSTGAELR	6	47573.4	8.26	10.36	96.8	5.97	11	2
			VQVVVTVLDYDK	6	47573.4	8.26	14.48	80.2	4.21	25	2
synaptotagmin II	31543670	SYT2	LTVCILEAK	7	46872.5	8.18	14.73	89.9	5.99	25	2
			TLNPAFNETFTFK	7	46872.5	8.18	16.12	90	5.66	25	2
			TLNPYFNESFSFEIPFEQIQK	7	46872.5	8.18	19.96	85.9	4.25	25	3
			TLVMAIYDFDR	7	46872.5	8.18	10.83	76.6	4.21	25	2
			VPMNTVDLGQPIEEWR	7	46872.5	8.18	15.82	89	4.14	18	3
			VPYQELGGK	7	46872.5	8.18	12.83	72.6	5.97	25	2
			VQVVVTVLDYDK	7	46872.5	8.18	14.48	80.2	4.21	25	2
synaptotagmin V	92859638	SYT5	LGDICFSLR	1	161813.4	9.26	14.44	90.4	5.83	25	2
talin 1	16753233	TLN2	AAMEPIVISAK	9	269669.4	5.75	12.27	73.6	6.05	25	2
			AATAPLLEAVDNLSAFASNPEFSSIPAQISPEGR	9	269669.4	5.75	20.21	92.2	4	25	3
			AVSSAIAQLLGEVAQGNENYAGIAAR	9	269669.4	5.75	14.14	73	4.53	25	3
			GSQEKLAQAAQSSVATITRLA	9	269669.4	5.75	10.63	85.5	8.75	14	3
			GTEWVDPEDPTVIAENELLGAAAAIEAAAK	9	269669.4	5.75	17.86	85	3.71	25	3
			GVAALTSDPAVQAIVLDTASDVLDK	9	269669.4	5.75	14.74	78.9	3.77	25	2
			LNEAAAGLNQAATELVQASR	9	269669.4	5.75	10.41	72	4.53	25	3
			VGAIPANALDDGQWSQGLISAAR	9	269669.4	5.75	14.08	77.4	4.21	25	3

			VGDDPAVWQLK	9	269669.4	5.75	14.06	81.5	4.21	25	2
TIP120 protein	21361794	CAND1	KISGSILNELIGLVRSPL	2	136376.5	5.52	15.07	89	8.75	25	3
			LLTIPEAEK	2	136376.5	5.52	12.42	71	4.53	25	2
tissue inhibitor of metalloproteinase 1 precursor	4507509	TIMP1	CNSDLVIR	5	23171	8.46	13.47	87.1	5.83	25	2
			EPGLCTWQSLR	5	23171	8.46	12.78	74.7	6.09	25	2
			FVYTPAMESVCGYFHR	5	23171	8.46	13.33	95.3	6.74	20	3
			GFQALGDAADIR	5	23171	8.46	13.78	80	4.21	25	2
			LQDGLLHITTCSFVAPWNSLSLAQR	5	23171	8.46	15.28	74	6.73	25	3
tissue inhibitor of metalloproteinase 2 precursor	4507511	TIMP2	EVDSGNDIYGNPIK	3	24399.4	7.46	12.66	74.8	4.03	24	2
			EVDSGNDIYGNPIKR	3	24399.4	7.46	12.4	72.5	4.56	25	3
			GAAPPKQEFLDIEDP	3	24399.4	7.46	10.43	72.9	3.91	19	2
tissue inhibitor of metalloproteinase 3 precursor	4507513	TIMP3	CNSDIVIR	4	24145.2	9	13.47	87.1	5.83	25	2
			SCYYLPCFVTSK	4	24145.2	9	10.64	79.1	7.76	18	2
			WDQLTLSQR	4	24145.2	9	11.07	85.9	5.84	17	2
			YQYLLTGR	4	24145.2	9	10.92	90.7	8.59	21	2
titin isoform N2-A	110349719	TTN	ARDHGESLDKA	12	3713692	6.16	10.83	80.5	5.38	25	3
			CKDELAPPTLHLDFRDKLTIRVGEA	12	3713692	6.16	11.5	74.8	5.52	25	3
			EKVPVPRKEVAPPVRVPEVP	12	3713692	6.16	10.47	70.2	8.69	16	3
			ESDGGSKITGYVVEMQTKGSE	12	3713692	6.16	10.64	73.5	4.62	18	3
			ETARFETEISEDDIHANWKL	12	3713692	6.16	10.24	80.8	4.35	22	3
			IPVKVPEVPRKPVPE	12	3713692	6.16	10.01	88.5	8.59	25	3
			KEKPPPAKVPEVPKKPVPEEKVPV	12	3713692	6.16	12.11	92.2	9.31	19	3
			RVSAVNHYGKGEP	12	3713692	6.16	12.59	86.9	8.6	21	3
			SCSTALTVKAPPVFTQKPSPV	12	3713692	6.16	10.68	73.4	9.3	19	3
			SRVPIPTMPIRAVPPEEI	12	3713692	6.16	15.49	71	5.87	25	3
			TRWVPVNKSAIPER	12	3713692	6.16	11.76	76.2	10.83	25	3
			TVVARDDV	12	3713692	6.16	10.26	80.9	4.21	25	2
titin isoform N2-B	110349715	TTN	ARDHGESLDKA	7	3006843.3	6.35	10.83	80.5	5.38	25	3
			CKDELAPPTLHLDFRDKLTIRVGEA	7	3006843.3	6.35	11.5	74.8	5.52	25	3
			ESDGGSKITGYVVEMQTKGSE	7	3006843.3	6.35	10.64	73.5	4.62	18	3
			ETARFETEISEDDIHANWKL	7	3006843.3	6.35	10.24	80.8	4.35	22	3
			RVSAVNHYGKGEP	7	3006843.3	6.35	12.59	86.9	8.6	21	3
			TRWVPVNKSAIPER	7	3006843.3	6.35	11.76	76.2	10.83	25	3
			TVVARDDV	7	3006843.3	6.35	10.26	80.9	4.21	25	2
titin isoform novex-1	110349713	TTN	ARDHGESLDKA	7	3006843.3	6.36	10.83	80.5	5.38	25	3
			CKDELAPPTLHLDFRDKLTIRVGEA	7	3006843.3	6.36	11.5	74.8	5.52	25	3
			ESDGGSKITGYVVEMQTKGSE	7	3006843.3	6.36	10.64	73.5	4.62	18	3
			ETARFETEISEDDIHANWKL	7	3006843.3	6.36	10.24	80.8	4.35	22	3
			RVSAVNHYGKGEP	7	3006843.3	6.36	12.59	86.9	8.6	21	3
			TRWVPVNKSAIPER	7	3006843.3	6.36	11.76	76.2	10.83	25	3
			TVVARDDV	7	3006843.3	6.36	10.26	80.9	4.21	25	2
titin isoform novex-2	110349717	TTN	ARDHGESLDKA	7	3006843.3	6.31	10.83	80.5	5.38	25	3
			CKDELAPPTLHLDFRDKLTIRVGEA	7	3006843.3	6.31	11.5	74.8	5.52	25	3
			ESDGGSKITGYVVEMQTKGSE	7	3006843.3	6.31	10.64	73.5	4.62	18	3
			ETARFETEISEDDIHANWKL	7	3006843.3	6.31	10.24	80.8	4.35	22	3
			RVSAVNHYGKGEP	7	3006843.3	6.31	12.59	86.9	8.6	21	3
			TRWVPVNKSAIPER	7	3006843.3	6.31	11.76	76.2	10.83	25	3
			TVVARDDV	7	3006843.3	6.31	10.26	80.9	4.21	25	2
toll interacting protein	21361619	TOLLIP	AIQDMFPNMDQEVIR	4	30282	5.68	11	88.5	4.03	14	3
			DAAINSLLQMGEEP	4	30282	5.68	18.08	94.4	3.57	25	2
			IAWTHITIPESLR	4	30282	5.68	18.83	93.3	6.75	22	3
			VIHCTVPPGVDSFYLEIFDER	4	30282	5.68	16.88	90	4.31	25	3
tomosyn	31652247	STXBP5	GAASGVVGELAR	1	123465.7	6.72	14.39	77.6	6	25	2
transcobalamin II precursor	21071010	TCN2	LSLEHLNPSIYVGLR	1	47535.3	6.52	17.48	90.8	6.75	25	3
transferrin	4557871	TF	ADRDQYELLCLDNTR	11	77050.4	6.81	15.96	85.7	4.23	25	3
			EGTCPEAPTDECKPVK	11	77050.4	6.81	14.95	83.3	4.41	25	3
			EGYYGYTGAFR	11	77050.4	6.81	13.04	87.7	6.1	25	2
			HSTIFENLANK	11	77050.4	6.81	14.99	75.5	6.75	25	3
			IECVSAETTEDCIAK	11	77050.4	6.81	16.87	74.2	4	25	2
			KPVEEYANCHLAR	11	77050.4	6.81	16.48	79.3	6.75	25	3
			LKCDEWSVNSVGK	11	77050.4	6.81	13.27	82.7	6.06	23	3
			NLNEKDYELLCLDGTR	11	77050.4	6.81	15.39	83.4	4.32	25	3
			SAGWNIPIGLLYCDLPEPR	11	77050.4	6.81	16.2	81.1	4.37	25	3
			SASDLTWDNLK	11	77050.4	6.81	12.98	78.4	4.21	25	2
			SVIPSDGPSVACVK	11	77050.4	6.81	20.2	95.2	5.55	25	2
transgelin 3	56549135	TAGLN3	GFSEEQLR	2	22472.8	6.84	12.53	84.9	4.53	25	2
			LINSLYPPGQEPIPK	2	22472.8	6.84	10.95	75.9	6	25	2
triosephosphate isomerase 1	4507645	TPI1	IAVAAQNCYK	3	26669.6	6.45	14.33	87.5	8.2	22	2
			IIYGGSVTGATCK	3	26669.6	6.45	19.48	94.5	8.2	25	2
			SNVSDAVAQSTR	3	26669.6	6.45	21.3	93.8	5.55	25	2
tripeptidyl-peptidase I preproprotein	5729770	TPP1	ILSGRPPLGFLNPR	7	61248.2	6.01	12.42	85.2	12	25	3
			LFGGNFAHQASVAR	7	61248.2	6.01	15.7	92.3	9.75	25	3
			LITNEIVDYISGGGFSNVFPR	7	61248.2	6.01	18.36	89.1	4.37	25	3
			LYQQHGAGLFDVTR	7	61248.2	6.01	14.93	82	6.74	25	3
			TVSYGDDEDSLSSAYIQR	7	61248.2	6.01	11.42	71.8	3.84	25	2
			VPIPWVSGTSASTPVFGGILSLINEHR	7	61248.2	6.01	19.2	74.8	6.72	25	3
			VVSNRVPIPWVSGTS	7	61248.2	6.01	11.85	84	9.72	23	3
tubulin, alpha 1a	17986283	TUBA1A	AVFVDLEPTVIDEVR	8	50135.9	4.94	12.93	80.7	3.91	25	2
			DVNAAIATIK	8	50135.9	4.94	19.12	93.7	5.84	25	2
			EIIDLVLDR	8	50135.9	4.94	13.22	94.2	4.03	25	2
			IHFPLATYAPVISAEK	8	50135.9	4.94	18.96	87.5	6.75	25	3
			NLDIERPTYTNLNR	8	50135.9	4.94	16.55	89.6	6.07	25	3
			TIGGGDDSFNTFFSETGAGK	8	50135.9	4.94	14.02	80.2	4.03	25	2
			VGINYQPPTVVPGGDLAK	8	50135.9	4.94	20.9	93.9	5.8	25	3
			YMACCLLYR	8	50135.9	4.94	11.05	78	8.04	25	2
tubulin, beta, 4	50592996	MC1R TUBB3	AILVDLEPGTMDSVR	5	50433	4.83	15.49	85	4.03	25	2
			EIVHIQAGQCGNQIGAK	5	50433	4.83	18.77	90.5	6.83	25	3
			IMNTFSVVPSPK	5	50433	4.83	13.62	87.2	8.75	25	2
			LAVNMVPFPR	5	50433	4.83	17.49	92.5	9.75	25	2
			NSSYFVEWIPNNVK	5	50433	4.83	12.86	77.1	6	25	2
tumor rejection antigen (gp96) 1	4507677	HSP90B1	DDEVDVDGTVEEDLGK	10	92469.3	4.76	13.64	78.4	3.54	25	2
			DISTNYYASQK	10	92469.3	4.76	14.76	86.5	5.83	25	2
			EAESSPFVER	10	92469.3	4.76	11.89	70	4.25	25	2
			ELISNASDALDK	10	92469.3	4.76	13.94	86.8	4.03	25	2
			GVVDSDDLPLNVSR	10	92469.3	4.76	20.77	92.3	3.93	25	2
			LTESPCALVASQYGWSGNMER	10	92469.3	4.76	15.29	85	4.53	25	3
			NLLHVTDTGVGMTR	10	92469.3	4.76	16.26	81.3	6.74	25	3
			SGYLLPDTK	10	92469.3	4.76	15.52	88.7	5.55	25	2
			SILFVPTSAPR	10	92469.3	4.76	15.37	93.3	9.47	25	2
			YSQFINFPIYVWSSK	10	92469.3	4.76	13.47	72.6	8.5	25	3
type IV alpha 6 collagen isoform A precursor	148536823	COL4A6	LPGLPGPRGLPGDKGKDGLPGQQ	2	163807.6	9.31	12.5	77	8.59	25	3

			QGIRGSPGLPGASGLPGLKGDNG	2	163807.6	9.31	15.64	86.5	8.75	25	3
tyrosine 3-monooxygenase/tryptophan 5-monooxygenase activation protein, beta polypeptide	4507949	YWHAB	DSTLIMQLLR	1	28082.5	4.76	12.86	91	5.84	17	2
tyrosine 3-monooxygenase/tryptophan 5-monooxygenase activation protein, gamma polypeptide	21464101	YWHAG	DSTLIMQLLR	1	98309.4	4.8	12.86	91	5.84	17	2
tyrosine 3/tryptophan 5 -monooxygenase activation protein, epsilon polypeptide	5803225	YWHAE	AASDIAMTELPPTHPIR	5	29174.1	4.63	17.85	86.1	5.32	25	3
			DSTLIMQLLR	5	29174.1	4.63	12.86	91	5.84	17	2
			EAAENSLVAYK	5	29174.1	4.63	14.17	87.6	4.53	25	2
			EALQDVEDENQ	5	29174.1	4.63	13.6	88.1	3.38	25	2
			LICCDILDVLDK	5	29174.1	4.63	10.63	72	3.93	25	2
tyrosine 3/tryptophan 5 -monooxygenase activation protein, eta polypeptide	4507951	YWHAH	AVTELNEPLSNEDR	2	28218.9	4.76	11.36	71.4	4	24	2
			DSTLIMQLLR	2	28218.9	4.76	12.86	91	5.84	17	2
tyrosine 3/tryptophan 5 -monooxygenase activation protein, zeta polypeptide	4507953	YWHAZ	DSTLIMQLLR	3	27745.3	4.73	12.86	91	5.84	17	2
			FLIPNASQAESK	3	27745.3	4.73	11.33	87.3	6	17	2
			SVTEQGAELSNEER	3	27745.3	4.73	21.12	95.4	4.09	25	2
tyrosine hydroxylase isoform a	88900501	TH	AGGPHLEYFVR	9	58600.5	5.9	14.9	92.9	6.79	21	3
			AVLNLLFSPR	9	58600.5	5.9	17.71	95.7	9.79	25	2
			AYGAGLLSSYGELLHCLSEEPEIR	9	58600.5	5.9	22.05	88.8	4.48	25	3
			DFLASLAFR	9	58600.5	5.9	18.89	97.5	5.84	20	2
			FDPDLDLDHPGFSDQVYR	9	58600.5	5.9	18.58	88.4	3.97	25	3
			FDPYTLAIDVLDSPQAVR	9	58600.5	5.9	21.38	94.6	3.93	25	2
			FSGYREDNIPQLEDVSR	9	58600.5	5.9	17.35	82	4.32	25	3
			TGFQLRPVAGLLSAR	9	58600.5	5.9	14.69	84.4	12	25	3
			VEYTAEEIATWK	9	58600.5	5.9	10.72	75.3	4.25	25	2
ubiquitin and ribosomal protein L40 precursor	4507761	UBA52	IQDKEGIPPDQQR	3	14728.4	9.87	18.95	90.7	4.56	25	3
			TITLEVEPSDTIENVK	3	14728.4	9.87	13.6	79.6	4	25	2
			TLSDYNIQK	3	14728.4	9.87	15.82	93	5.5	25	2
ubiquitin and ribosomal protein S27a precursor	4506713	RPS27A	IQDKEGIPPDQQR	3	17965	9.68	18.95	90.7	4.56	25	3
			TITLEVEPSDTIENVK	3	17965	9.68	13.6	79.6	4	25	2
			TLSDYNIQK	3	17965	9.68	15.82	93	5.5	25	2
ubiquitin C	67191208	zzzzz	IQDKEGIPPDQQR	3	77028.9	7.16	18.95	90.7	4.56	25	3
			TITLEVEPSDTIENVK	3	77028.9	7.16	13.6	79.6	4	25	2
			TLSDYNIQK	3	77028.9	7.16	15.82	93	5.5	25	2
UDP-GlcNAc:betaGal beta-1,3-N-acetylglucosaminyltransferase 1	5802984	B3GNT1	TALASGGVLDASGDYR	2	24608	6.77	15.7	88.7	4.21	23	2
			YEAAVPDPR	2	24608	6.77	14.68	73.7	4.37	25	2
v-Ha-ras Harvey rat sarcoma viral oncogene homolog isofrom 1	4885425	HRAS	YDPTIEDSYR	1	21298.3	5.16	16.37	94.4	4.03	25	2
vacuolar H+ ATPase E1 isoform a	4502317	ATP6V1E1	AEEEFNIEK	3	26145.5	7.7	16.28	92.7	4.09	25	2
			GALFGANANR	3	26145.5	7.7	12.26	75.9	9.75	25	2
			LDLIAQQMMPEVR	3	26145.5	7.7	11.75	75.9	4.37	25	2
vacuolar H+ ATPase E1 isoform c	87159818	ATP6V1E1	AEEEFNIEK	3	100895.7	6.66	16.28	92.7	4.09	25	2
			GALFGANANR	3	100895.7	6.66	12.26	75.9	9.75	25	2
			LDLIAQQMMPEVR	3	100895.7	6.66	11.75	75.9	4.37	25	2
vacuolar H+ATPase B2	19913428	ATP6V1B2	GPVVLAEDFLDIMGQPINPQCR	4	56501	5.57	16.41	83.5	4.03	25	3
			IYPEEMIQTGISAIDGMNSIAR	4	56501	5.57	15	75.2	4.14	25	3
			QIYPPINVLPSLSR	4	56501	5.57	17.16	90.3	8.75	25	2
			YAEIVHLTLPDGTK	4	56501	5.57	10.33	95.5	5.32	14	3
vacuolar protein sorting 35	17999541	VPS35	ILVGTNLVR	3	91707.5	5.32	12.28	86	9.75	25	2
			LSQLEGVNVER	3	91707.5	5.32	13.81	83.8	4.53	25	2
			SEDPDQQYLILNTAR	3	91707.5	5.32	12.74	80.9	4.03	25	2
valosin-containing protein	6005942	VCP	AIANECQANFISIK	7	89322.3	5.14	10.8	73.5	6.04	25	2
			DVDLEFLAK	7	89322.3	5.14	10.61	81.7	4.03	25	2
			ELQELVQYPVEHPDK	7	89322.3	5.14	12.63	83	4.4	25	3
			GVLFYGPPGCGK	7	89322.3	5.14	11.34	74.3	8.2	25	2
			LDQLIYIPLPDEK	7	89322.3	5.14	12.95	77	4.03	25	2
			LGDVISIQPCPDVK	7	89322.3	5.14	12.77	72.5	4.21	25	2
			NAPAIIFIDELDAIAPK	7	89322.3	5.14	17.62	83.5	4.03	25	3
vesicle amine transport protein 1	18379349	VAT1	ACGLNFADLMAR	16	41920.5	5.88	16.42	90.7	5.87	25	2
			AVCGFHLGYLDGEVELVSGVVAR	16	41920.5	5.88	13.49	82	4.65	25	3
			CLVLTGFGGYDK	16	41920.5	5.88	17.81	94.8	5.83	25	2
			EVAEAATGEDASSPPPK	16	41920.5	5.88	21.16	94.5	4	25	2
			IDSVWPFEK	16	41920.5	5.88	17.24	85.8	4.37	25	2
			LLALYNQGH	16	41920.5	5.88	16.33	95.5	6.74	25	2
			LQSRPAAPPAPGPGQLTLR	16	41920.5	5.88	21.11	89.2	12	25	3
			PAAPPAPGPGQLTLR	16	41920.5	5.88	19.67	98	10.18	20	2
			PGPGQLTLR	16	41920.5	5.88	10.99	73.3	10.18	19	2
			PQHPAASEGAAAAAAASPPLLR	16	41920.5	5.88	15.78	86.2	7.18	23	3
			SPPLLRCLVLTGFGGYDK	16	41920.5	5.88	12.55	73.6	7.92	25	3
			TEAASDPQHPAASEGAAAAAASPPLLR	16	41920.5	5.88	13.08	78.6	4.65	25	3
			TVENVTVFGTASASK	16	41920.5	5.88	19.39	90.9	5.66	25	2
			VLLVPGPEK	16	41920.5	5.88	10.94	96.1	5.97	16	2
			VLLVPGPEKEN	16	41920.5	5.88	15.09	88.5	4.53	25	2
			VVTYGMANLLTGPK	16	41920.5	5.88	20.77	96.6	8.56	25	2
vesicle-associated membrane protein 2 (synaptobrevin 2)	7657675	VAMP2	ADALQAGASQFETSAAK	1	60887.4	7.84	17.6	85.9	4.37	25	2
vesicle-associated membrane protein 3 (cellubrevin)	4759300	VAMP3	ADALQAGASQFETSAAK	1	49124.6	8.89	17.6	85.9	4.37	25	2
VGF nerve growth factor inducible precursor	17136078	VGF	APLPPPAPSQFQAR	10	67258.1	4.76	10.52	77.5	9.79	16	2
			AYQGVAAPFPK	10	67258.1	4.76	17.07	85	8.63	25	2
			LADLASDLLLQYLLQGGAR	10	67258.1	4.76	16.67	84.2	4.21	25	2
			MPDSGPLPETHK	10	67258.1	4.76	16.78	88.4	5.3	24	3
			NAPPEPVPPPR	10	67258.1	4.76	15.23	91.4	6	25	2
			RPESALLGGSEAGER	10	67258.1	4.76	23.11	95.5	4.79	25	3
			SPQPPPPAPAPAR	10	67258.1	4.76	12.08	79.1	9.47	25	2
			THLGEALAPLSK	10	67258.1	4.76	15.69	71.5	6.41	25	3
			TLQPPSALR	10	67258.1	4.76	14.57	88.2	9.41	25	2
			VNLESPGPER	10	67258.1	4.76	10.45	83.9	4.53	17	2
vinculin isoform meta-VCL	7669550	VCL	AIPDLTAPVAAVQAAVSNLVR	3	123800	5.5	11.62	86.2	5.88	16	2
			AVAGNISDPGLQK	3	123800	5.5	13.1	74.8	5.88	25	2
			AVAGNISDPGLQKS	3	123800	5.5	11.29	78.9	5.88	22	3
vinculin isoform VCL	4507877	VCL	AIPDLTAPVAAVQAAVSNLVR	3	14728.4	5.83	11.62	86.2	5.88	16	2
			AVAGNISDPGLQK	3	14728.4	5.83	13.1	74.8	5.88	25	2
			AVAGNISDPGLQKS	3	14728.4	5.83	11.29	78.9	5.88	22	3
vomeronasal 1 receptor 5	27777675	VN1R5	DMIISHLSL	2	40778.8	9.35	11.77	84.1	5.08	25	2
			TILLLFHIFTFVFSHRSKSIDMIISHL	2	40778.8	9.35	12.64	94.9	8.49	20	3

Table S1 Addendum.

Tandem mass spectrometry data for single peptide identification of proteins in the soluble fraction of human dense core secretory vesicles (DCSV).

Aconitase 2 Precursor ACO2

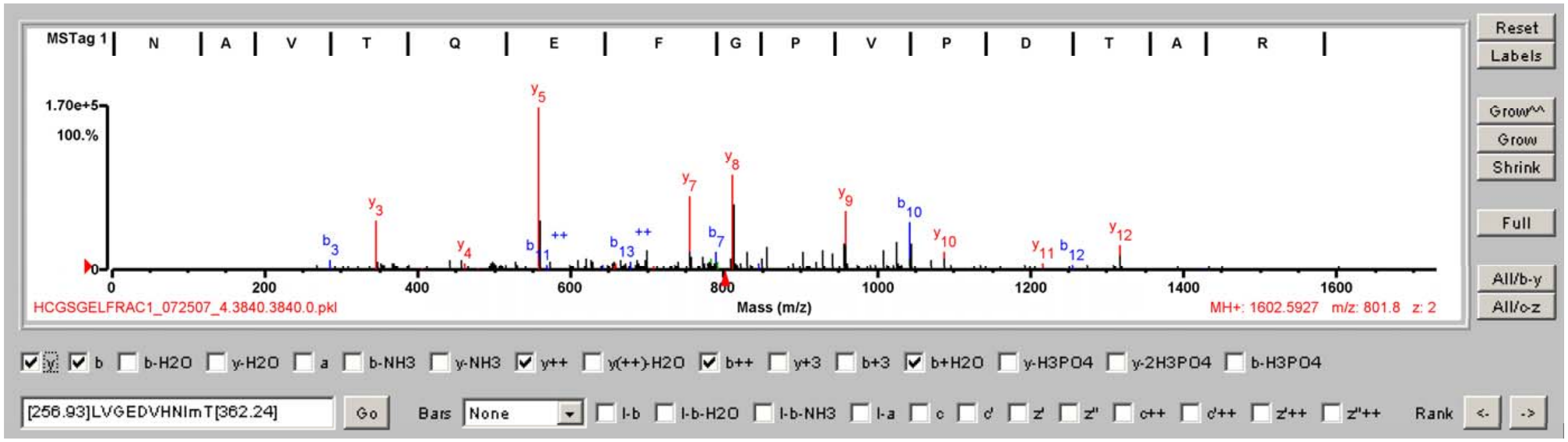

ADP Ribosylation Factor 1 ARF1

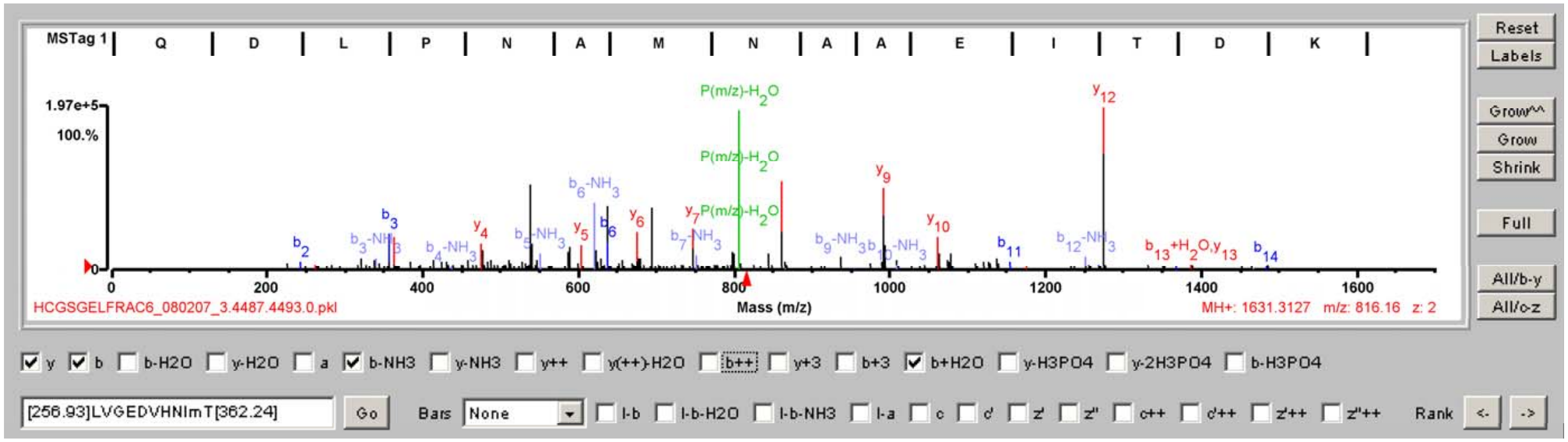

Alpha 2 Globin HBA1, HBA2

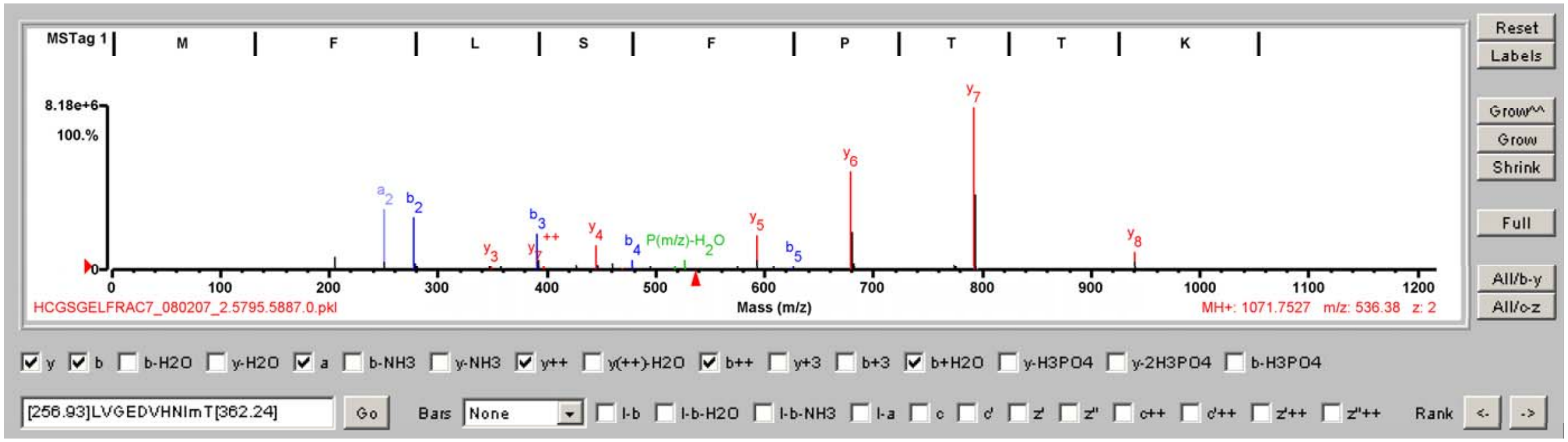

Apolipoprotein H Precursor APOH

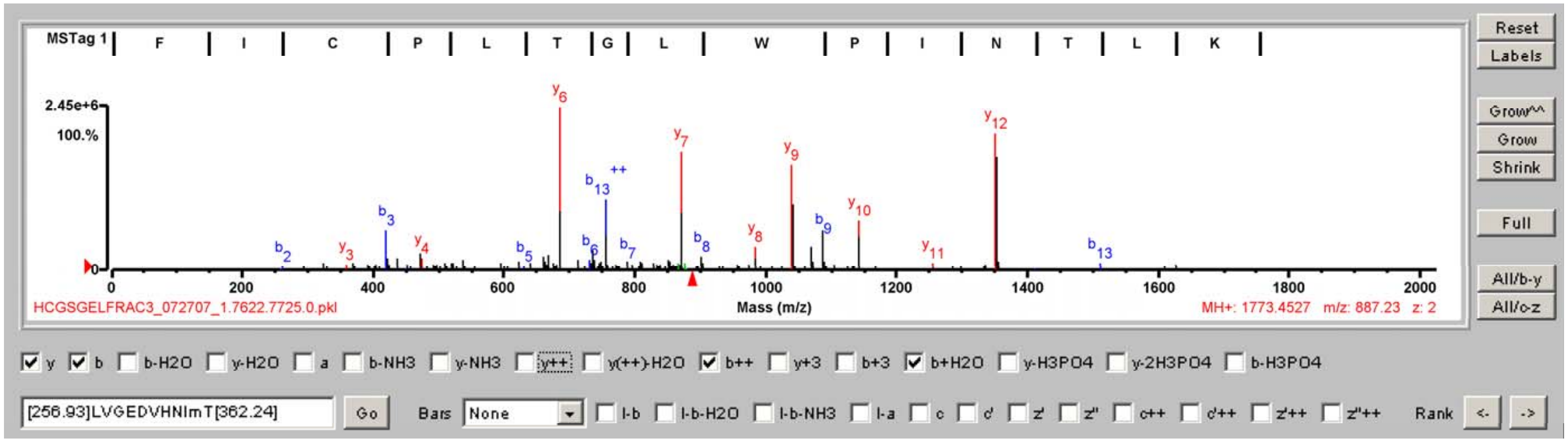

Alastin Isoform A ATL1

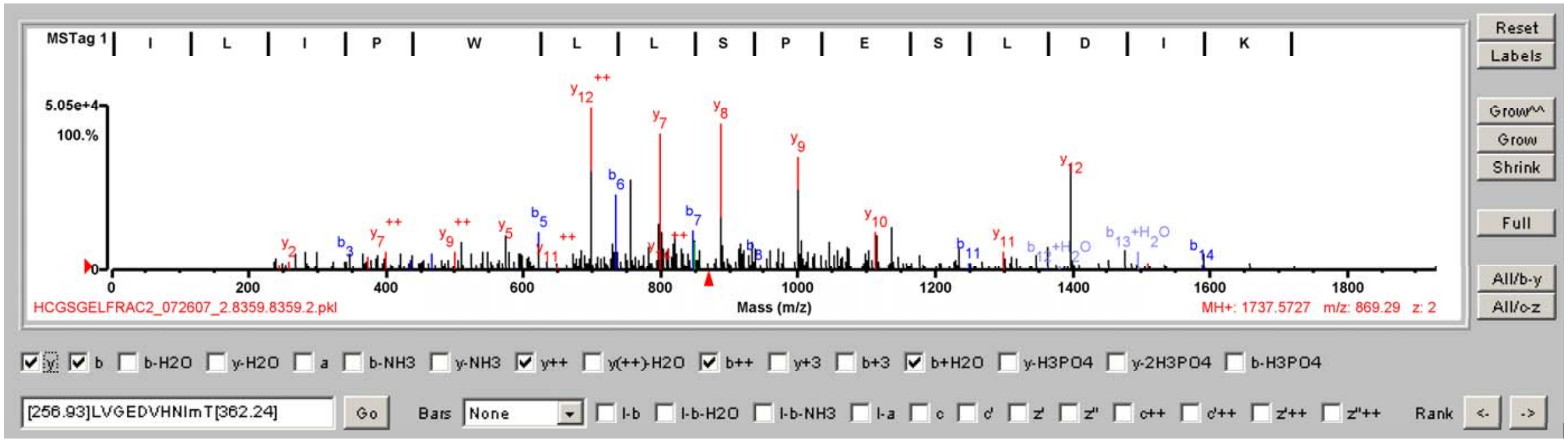

ATPase H+ Transporting Lysosomal 31kDa, V1 Subunit E Isoform 2 ATP6V1E2

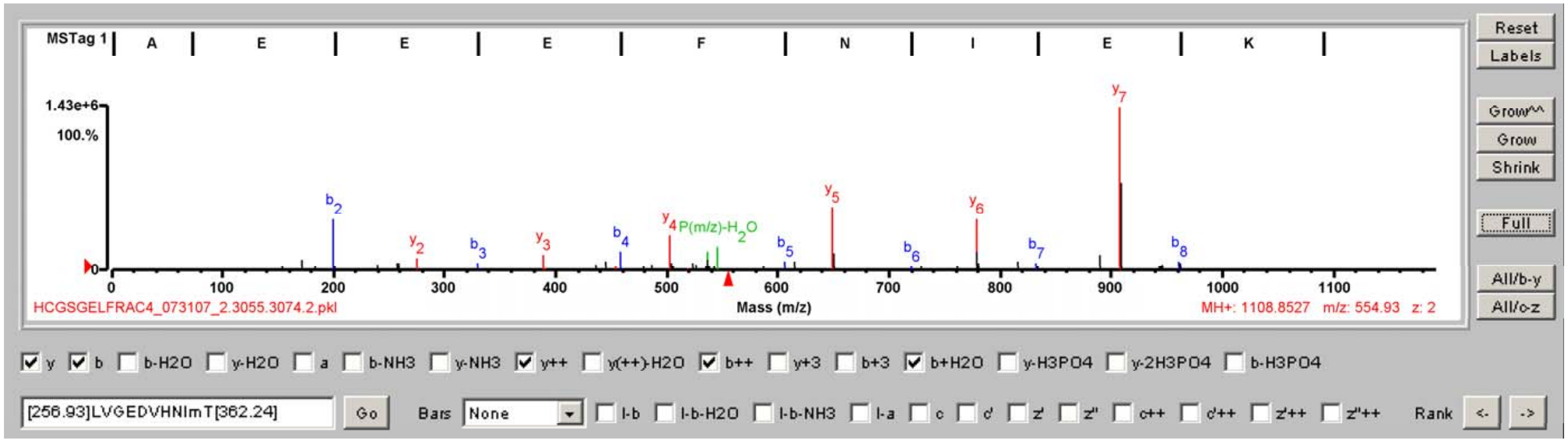

ATPase H+ Transporting Lysosomal 42kDa V1 Subunit C1 ATP6V1C1

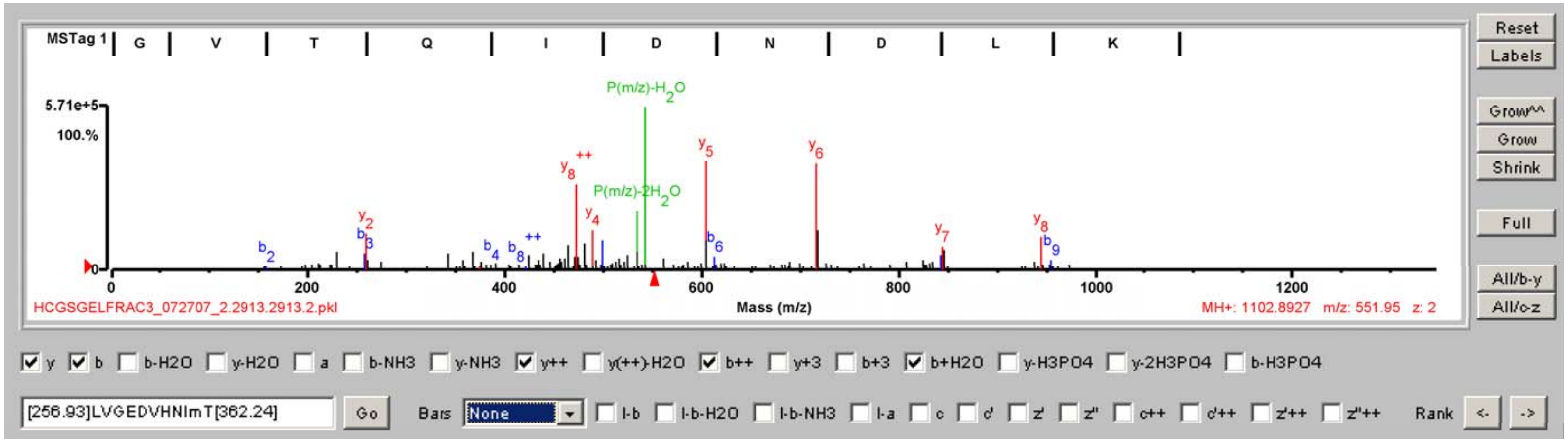

ATPase H+ Transporting Lysosomal 56/58kDa V1 Subunit B1 ATP6V1B1

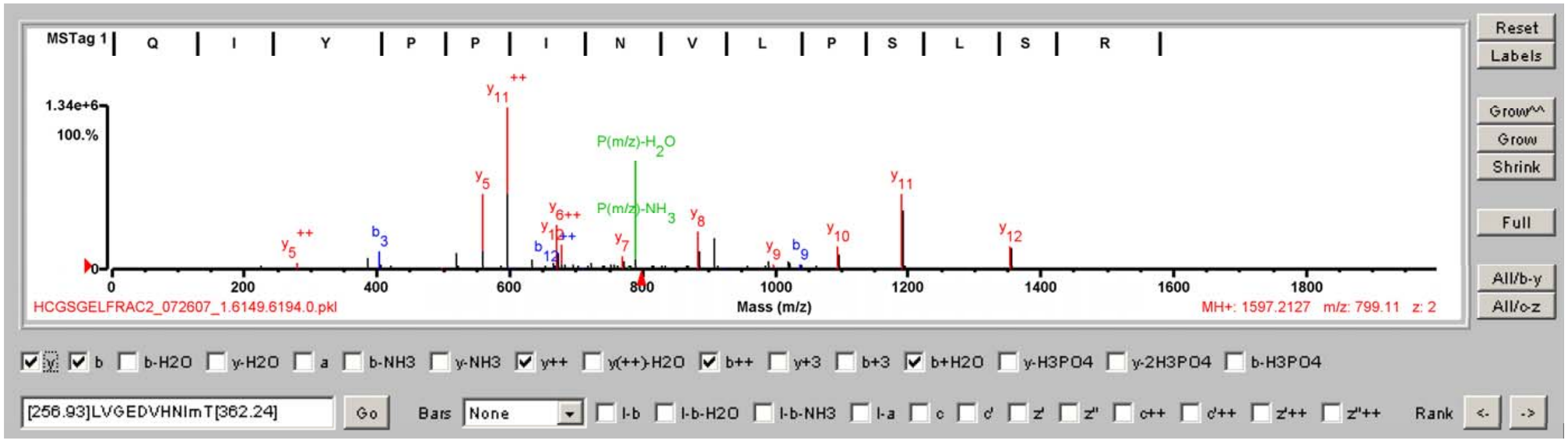

ATPase H+ Transporting Lysosomal Accessory Protein 2 ATP6AP2

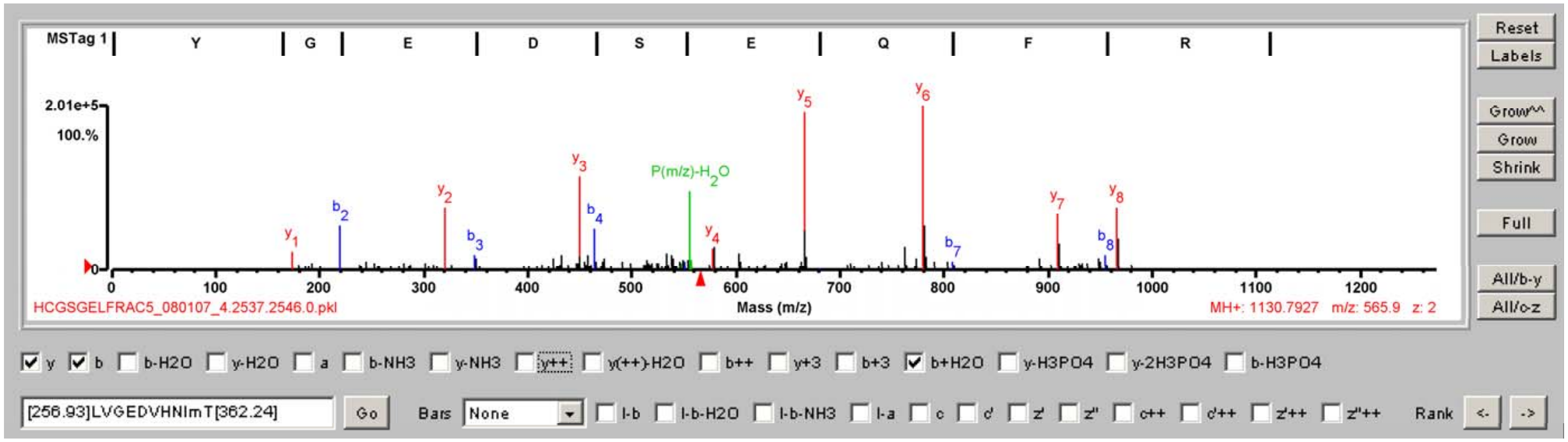

ATPase H+ Transporting Lysosomal V0 Subunit C ATP6V0C

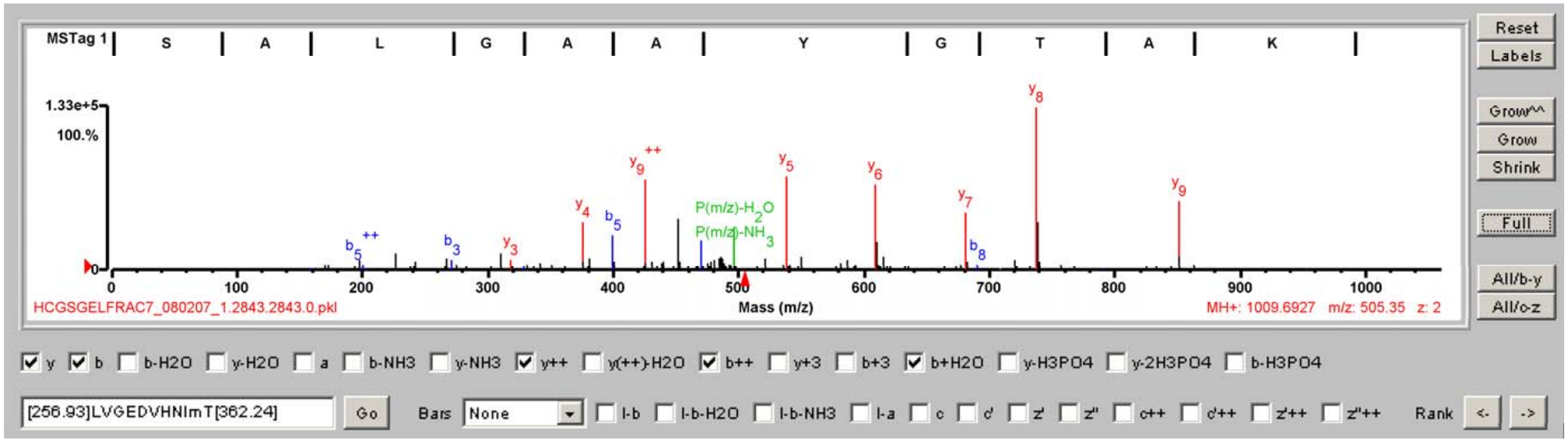

Beta Tubulin 1 Class IV TUBB1

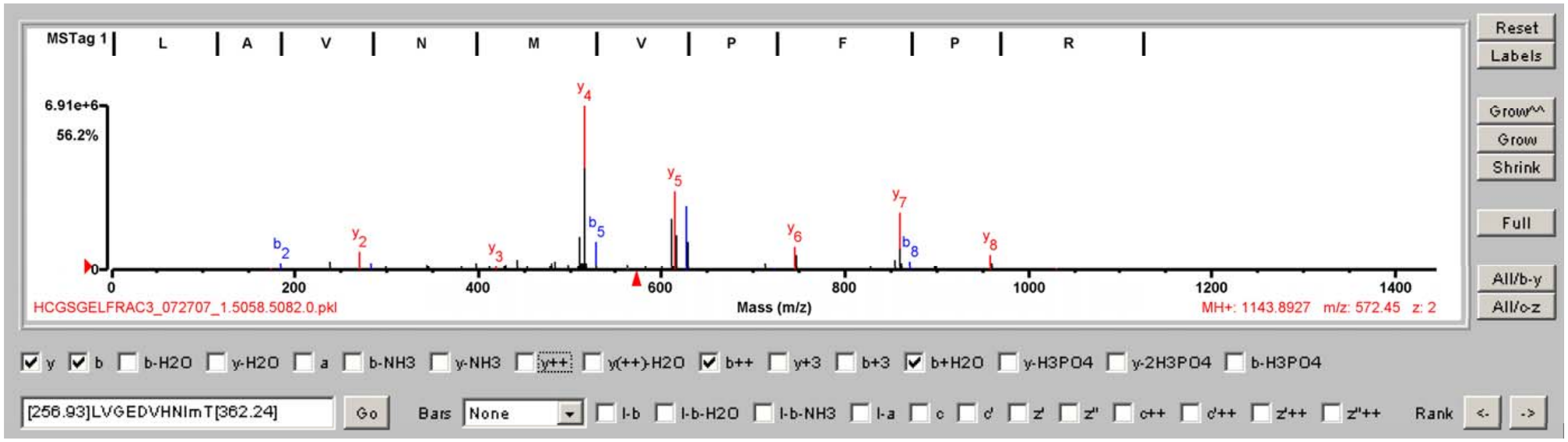

Calreticulin Precursor CALR

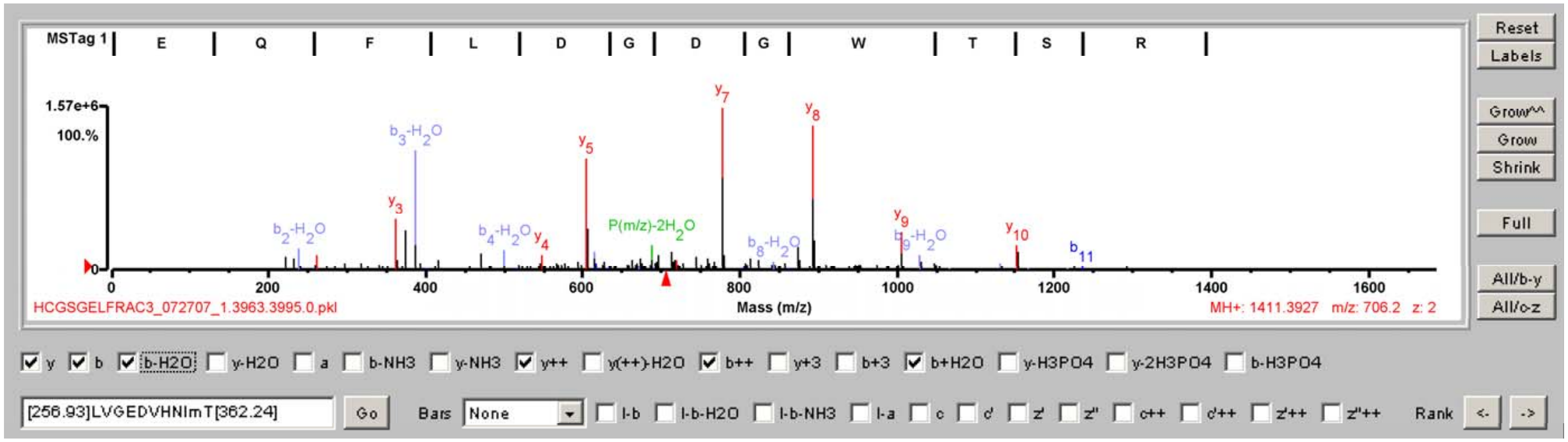

Cardiac Calsequestrin 2 CASQ2

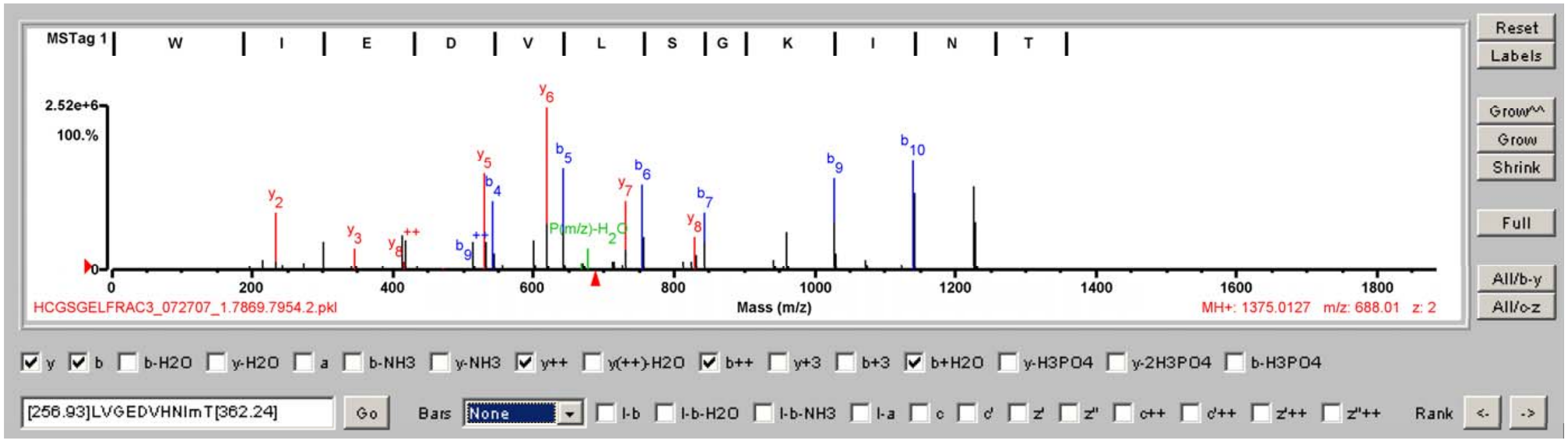

CASK Interacting Protein 1 CASKIN1

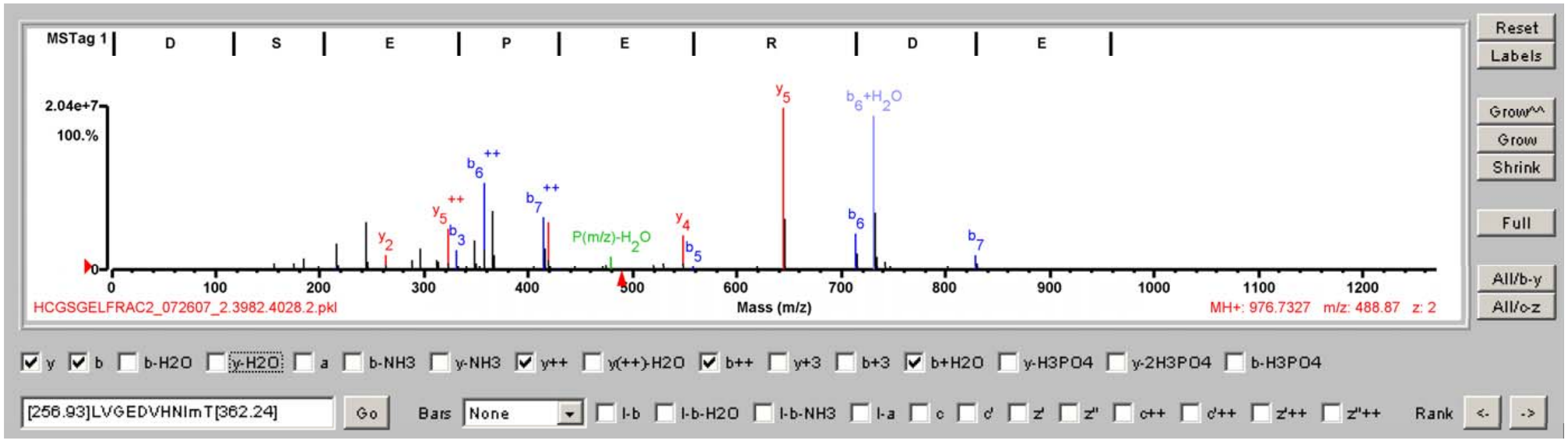

Cathepsin B Preproprotein CTSB

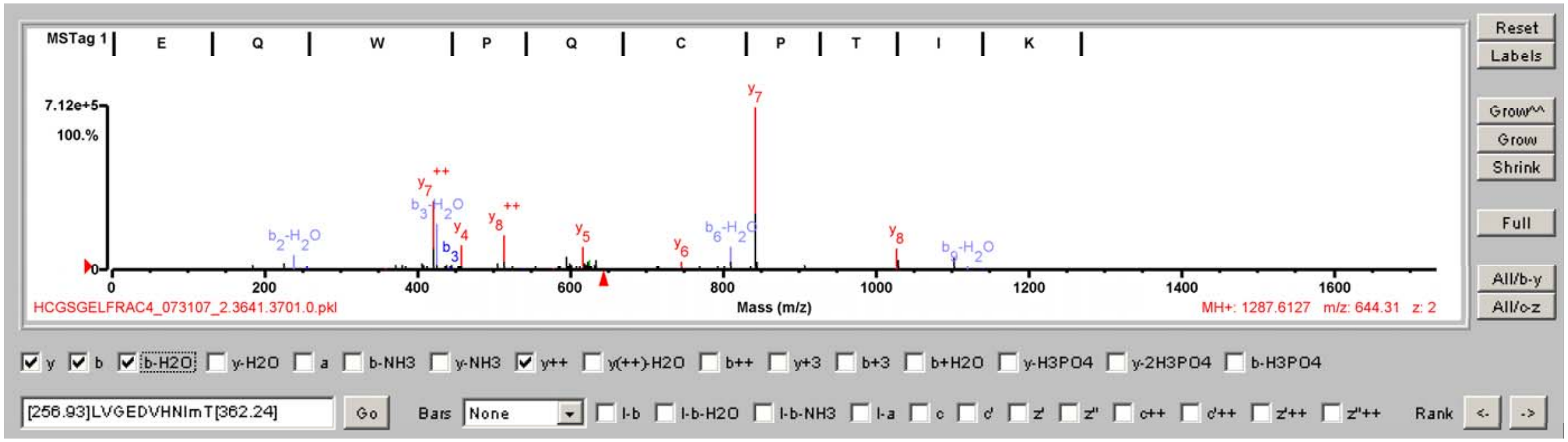

CD59 Antigen p18-20 CD59

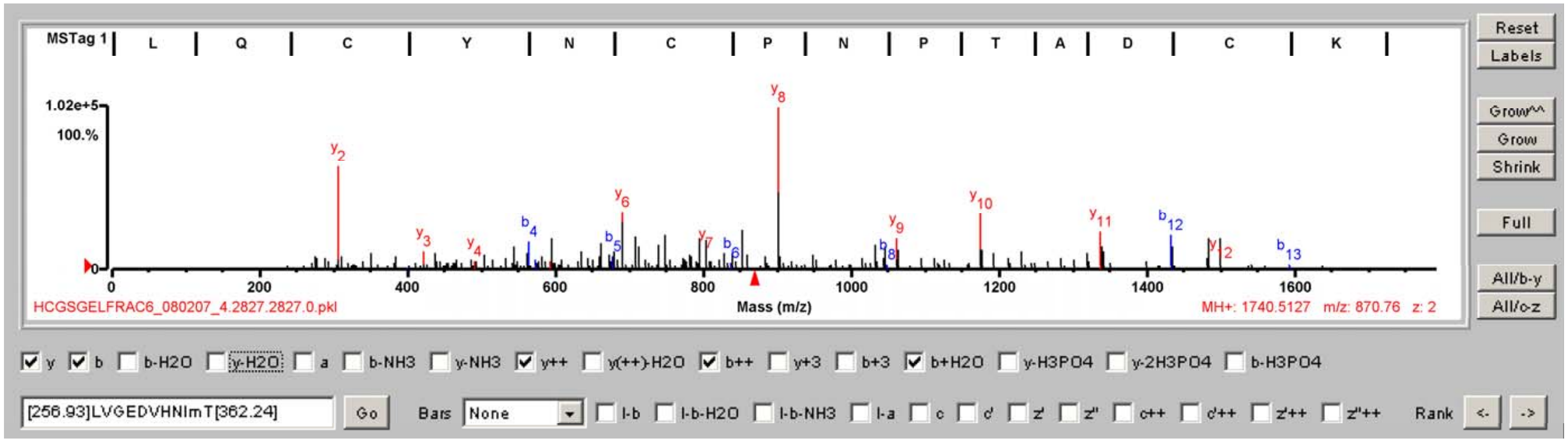

CD63 Antigen Isoform A CD63

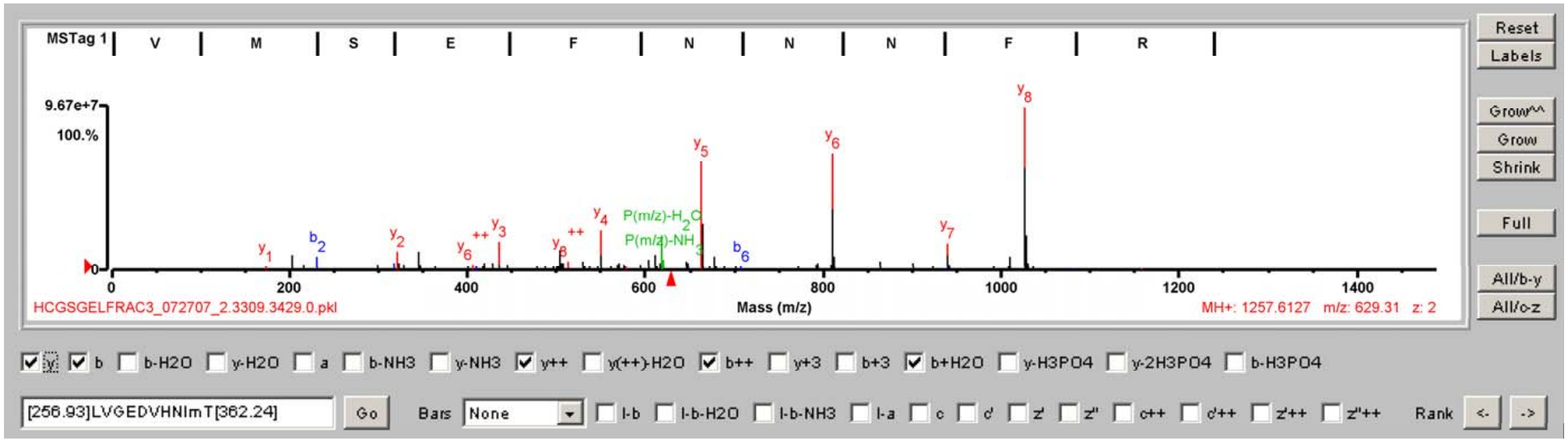

CD93 Antigen Precursor CD93

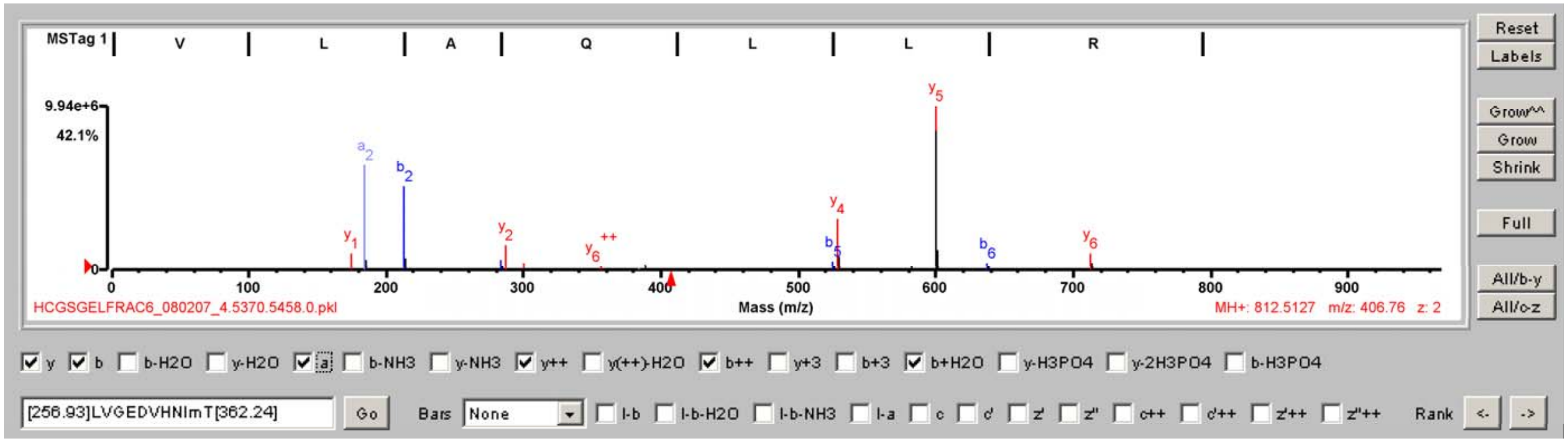

Chloride Intracellular Channel 1 CLIC1

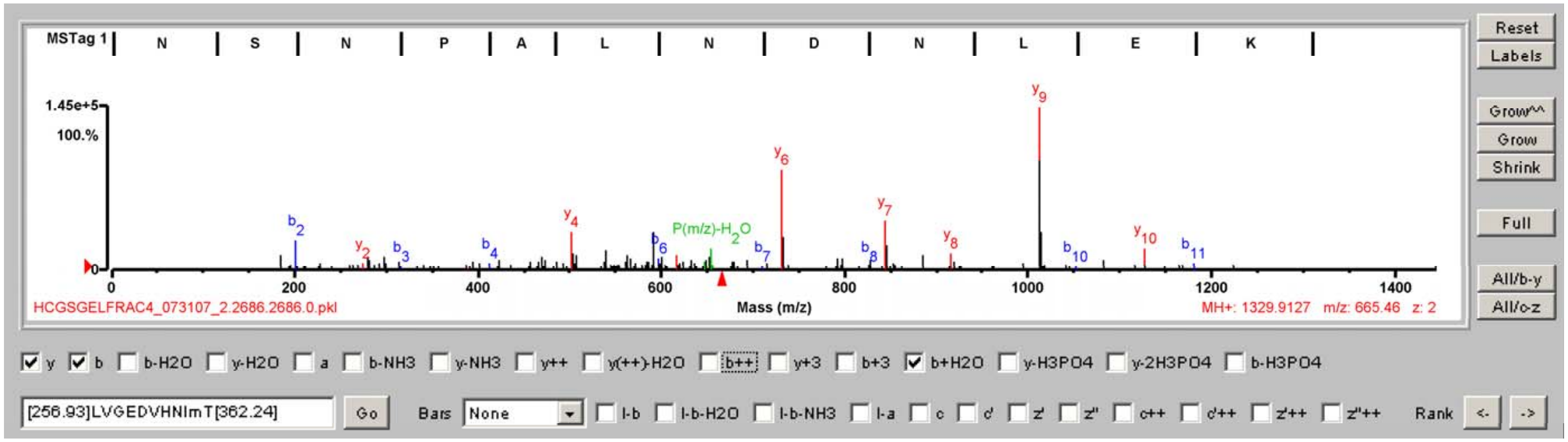

CLIP-Associating Protein 2 CLASP2

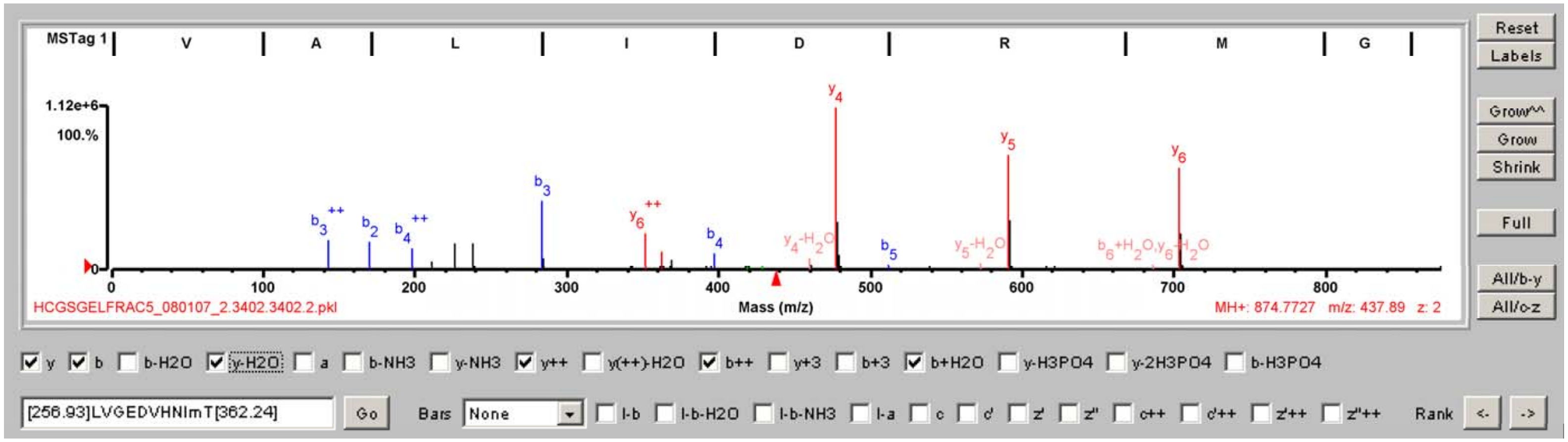

Coatomer Protein Complex Subunit Zeta 1 COPZ1

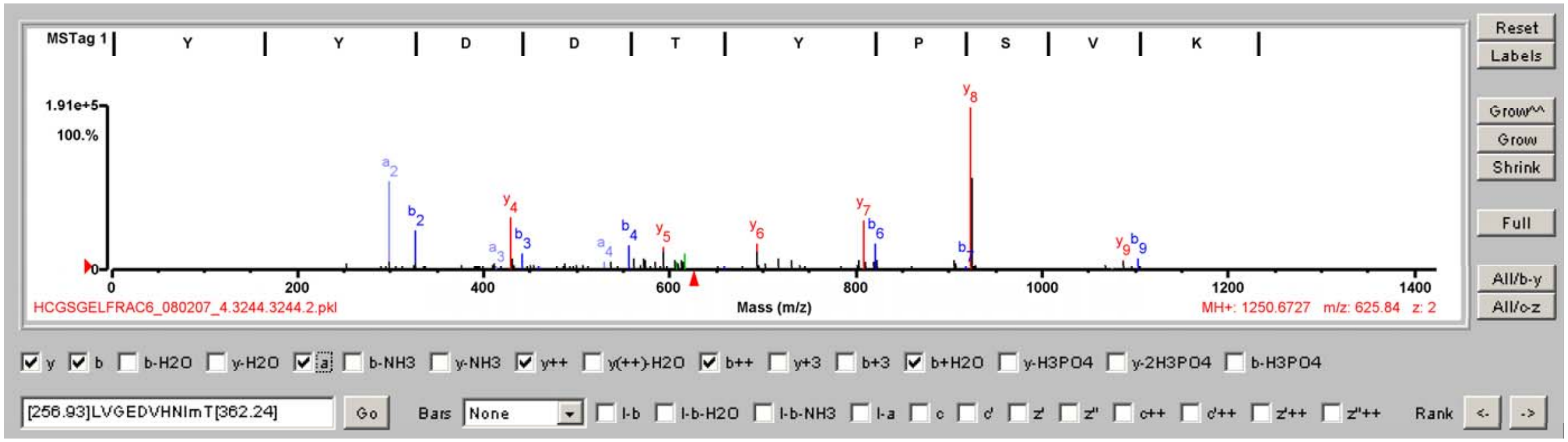

Cytochrome b5 Reductase Isoform 1 CYB5R3

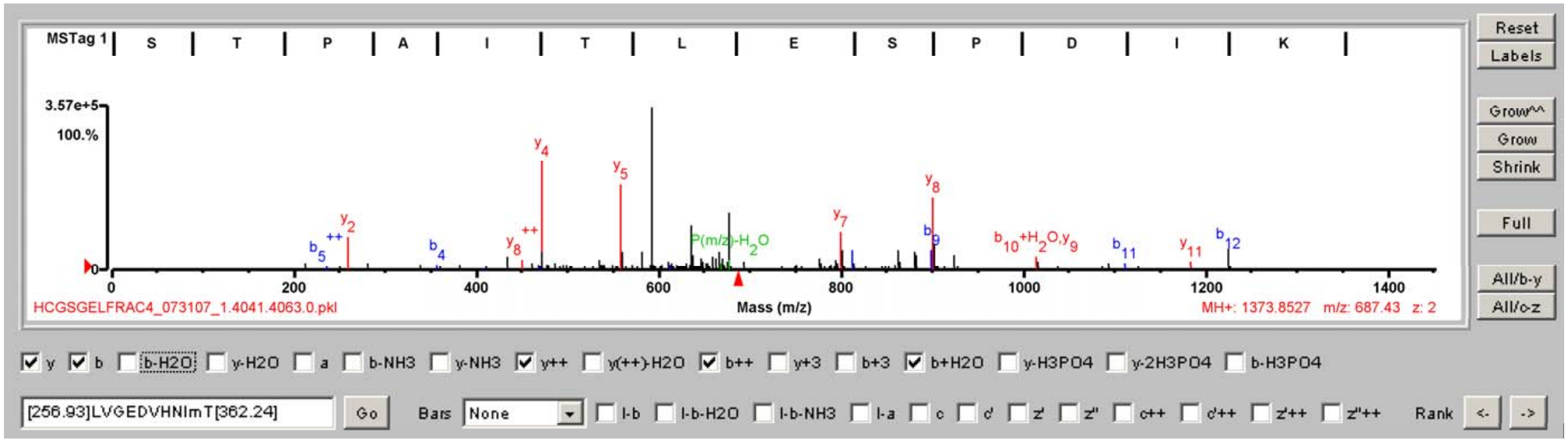

Defensin Alpha 1 Preproprotein DEFA1

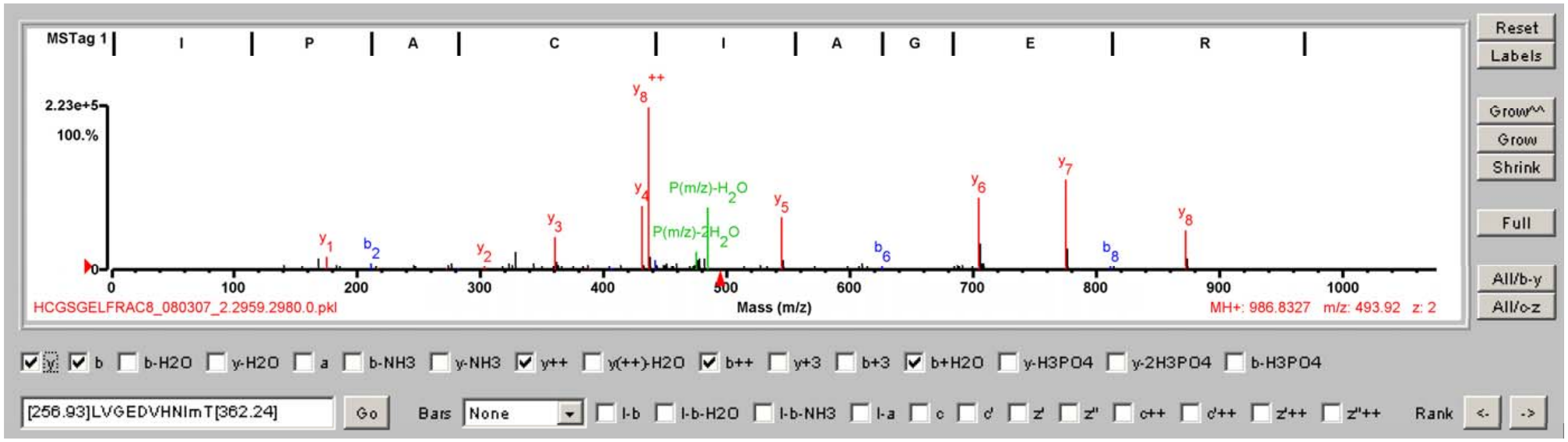

Dynactin 2 DCTN2

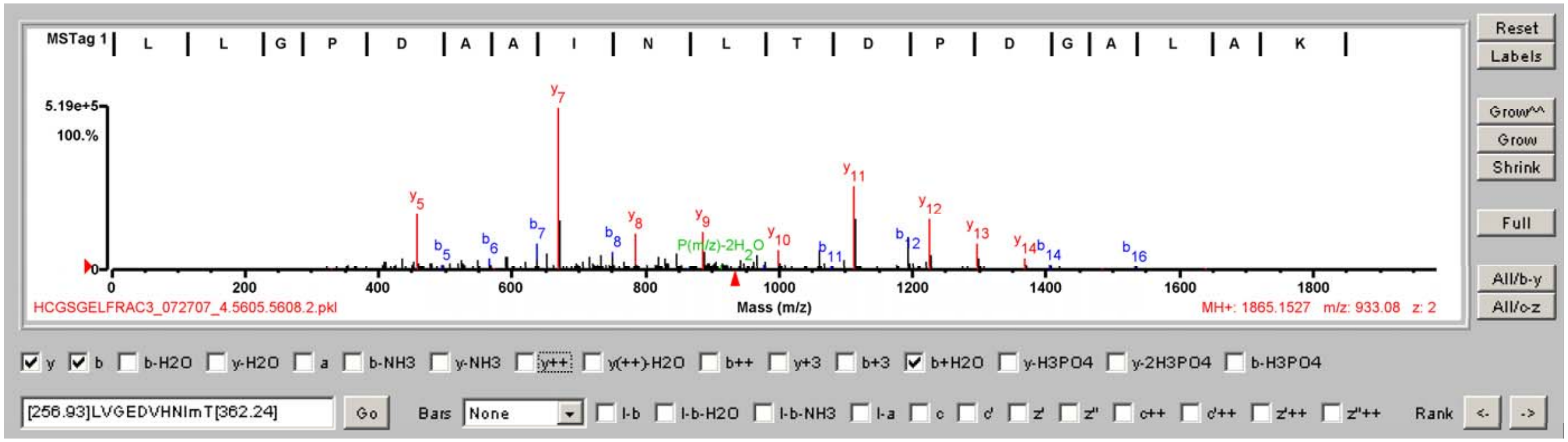

Dynamin 1, Dynamin 2 DNM1 DNM2

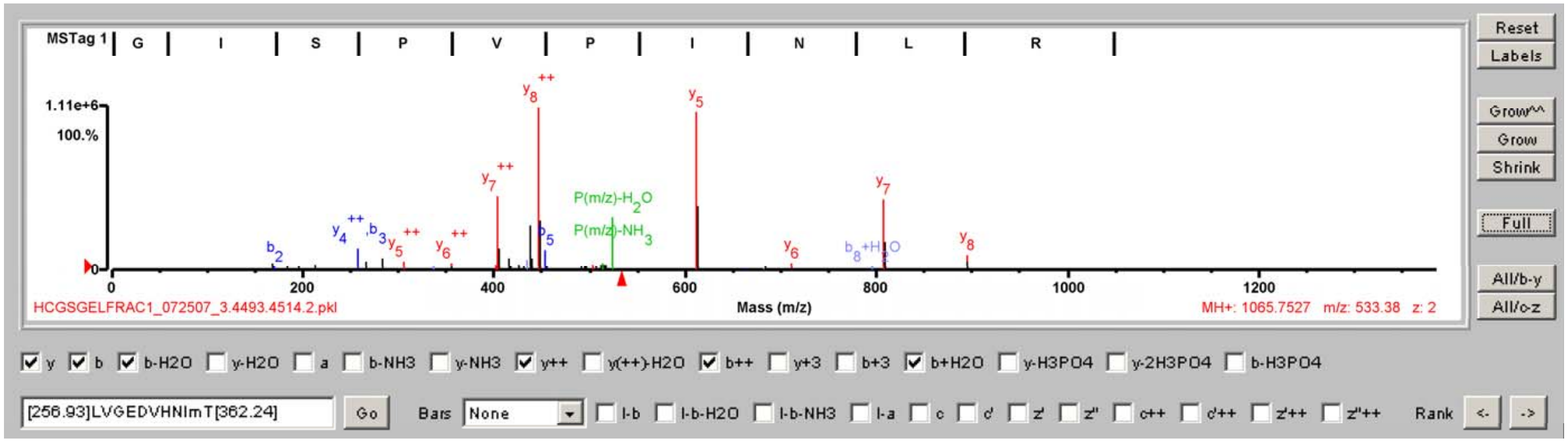

Dynein Cytoplasmic 1 Intermediate Chain 2 DYNC1I2

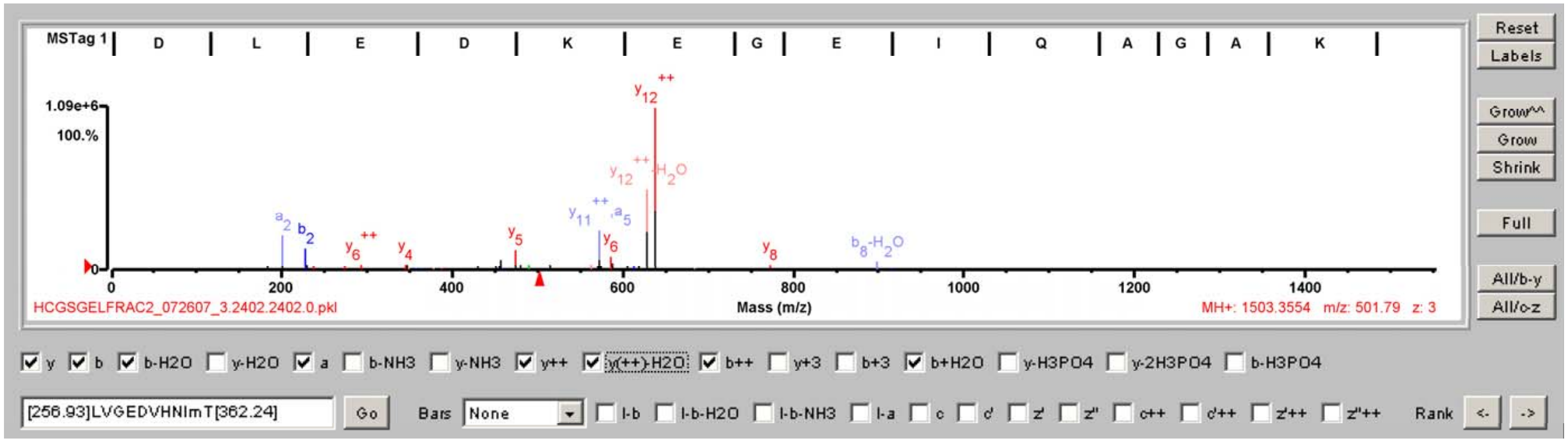

Epsilon Globin HBE1

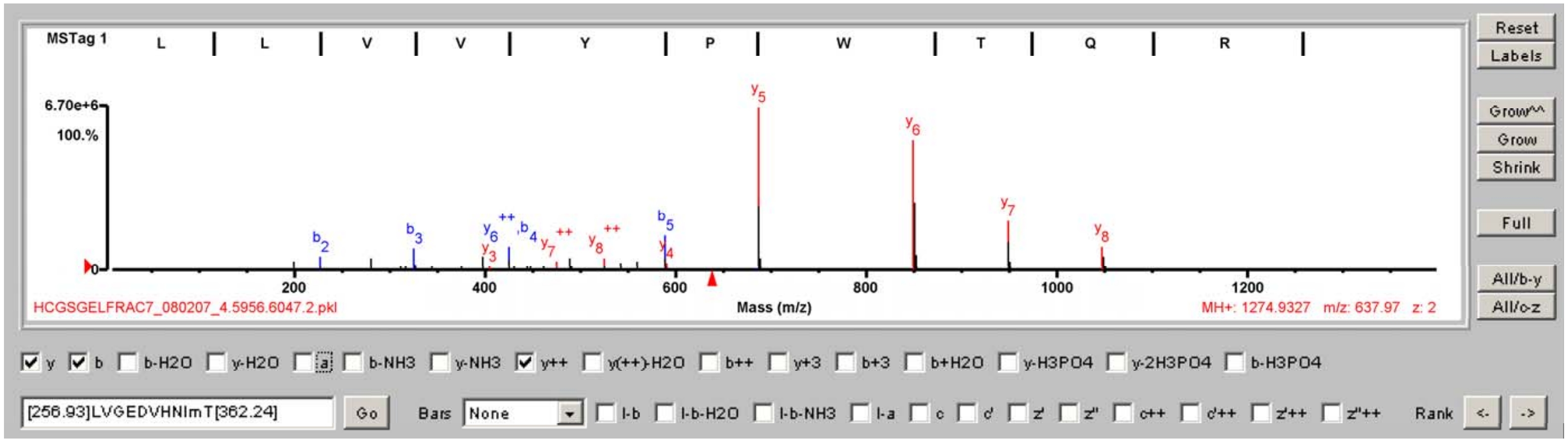

Estradiol 17 Beta-Hydrogenase 8 HSD17B8

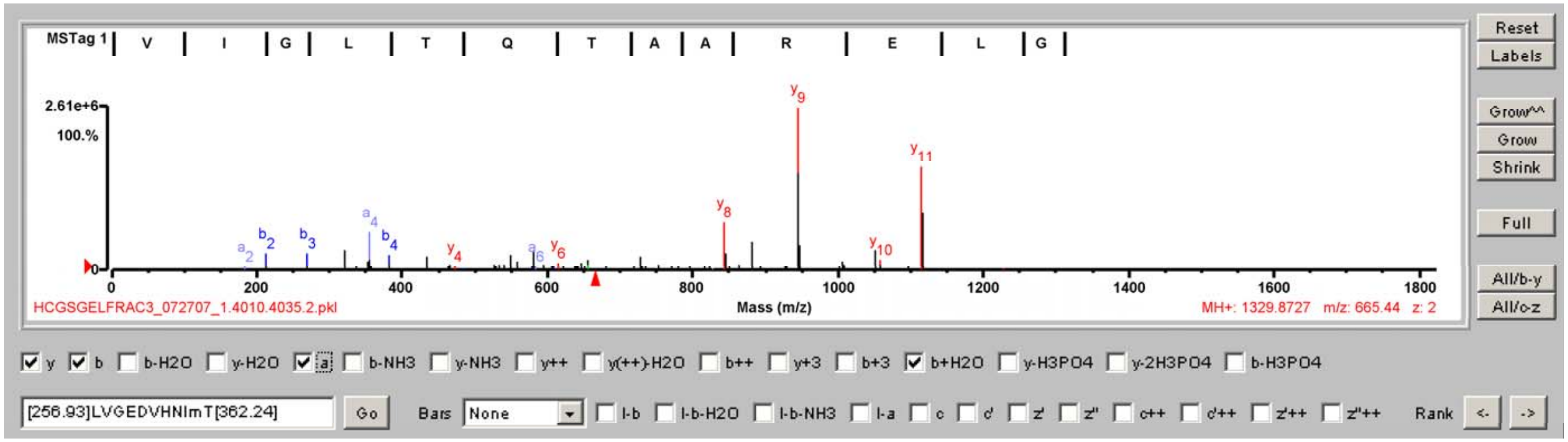

Ferritin Light Polypeptide FTL

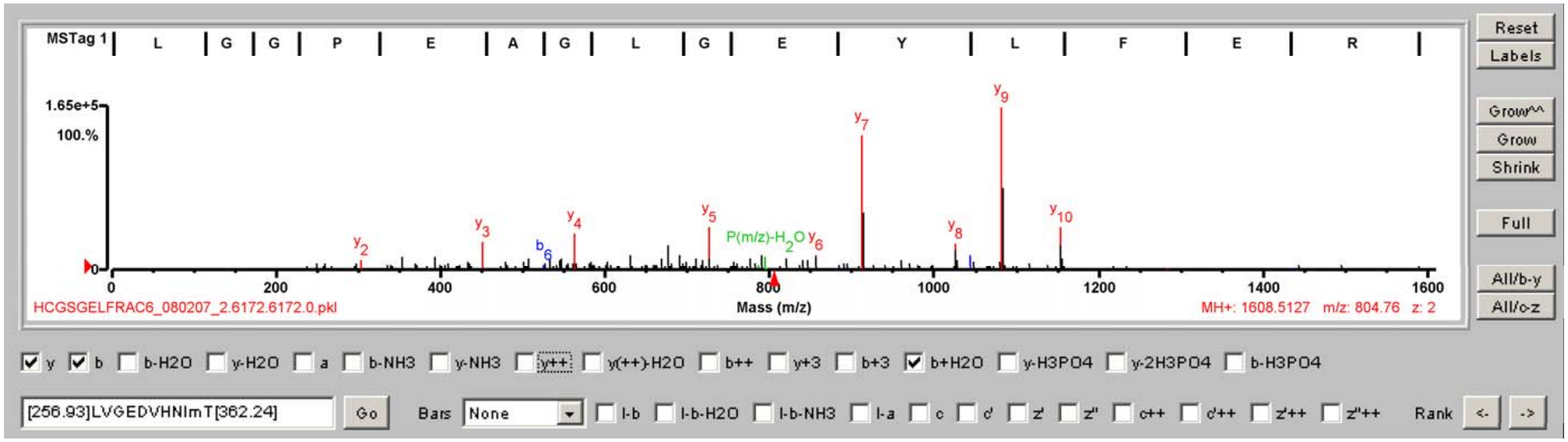

Fructose Bisphosphate Aldolase C ALDOC

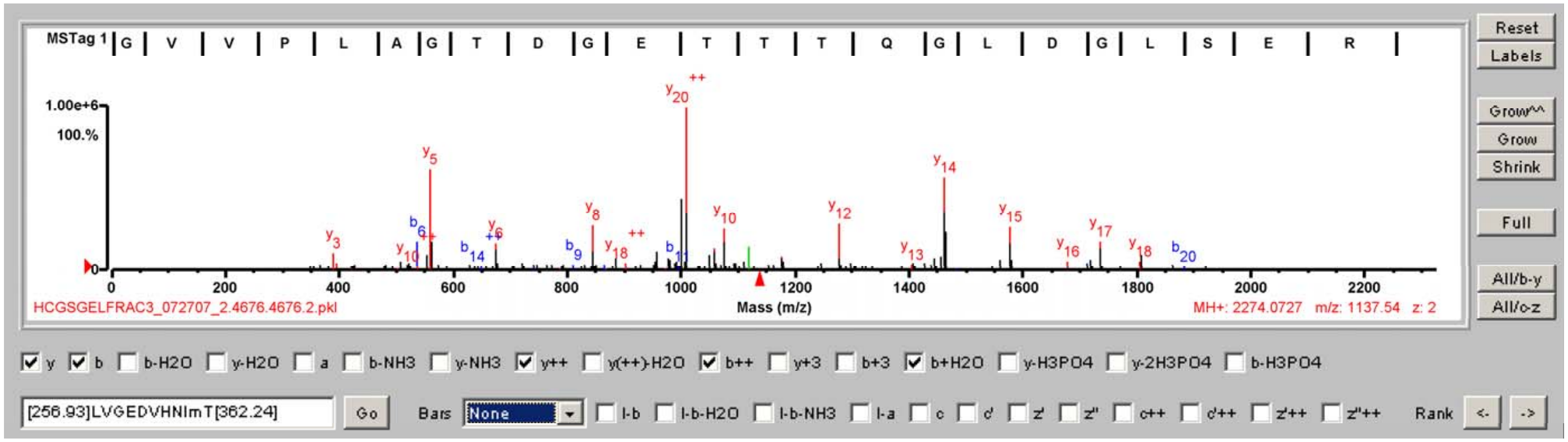

Gelsolin Isoform A Precursor GSN

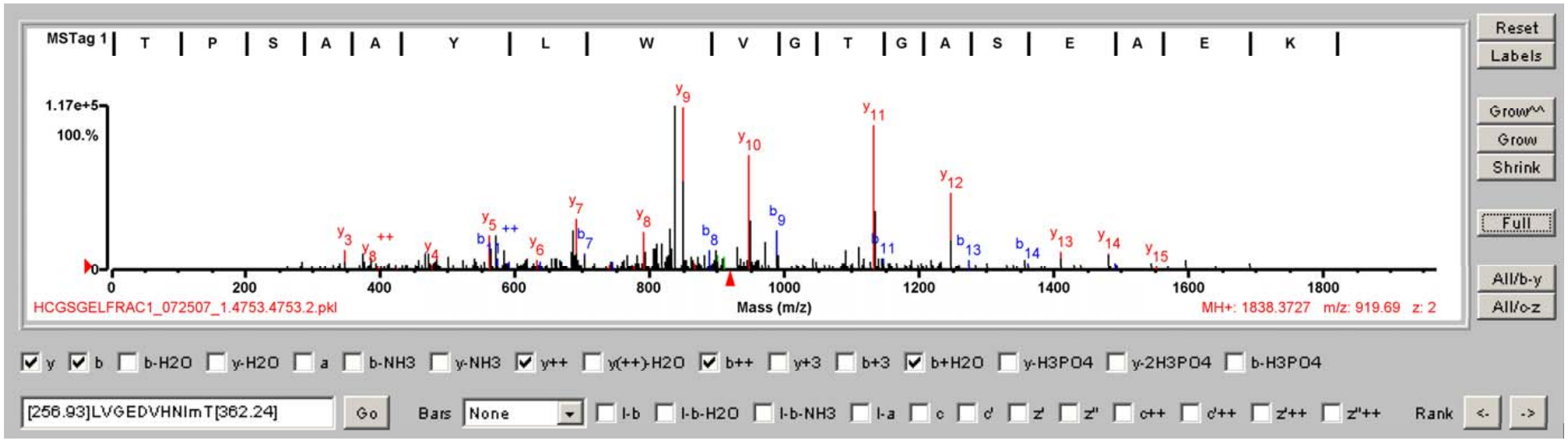

Glial Fibrillary Acidic Protein GFAP

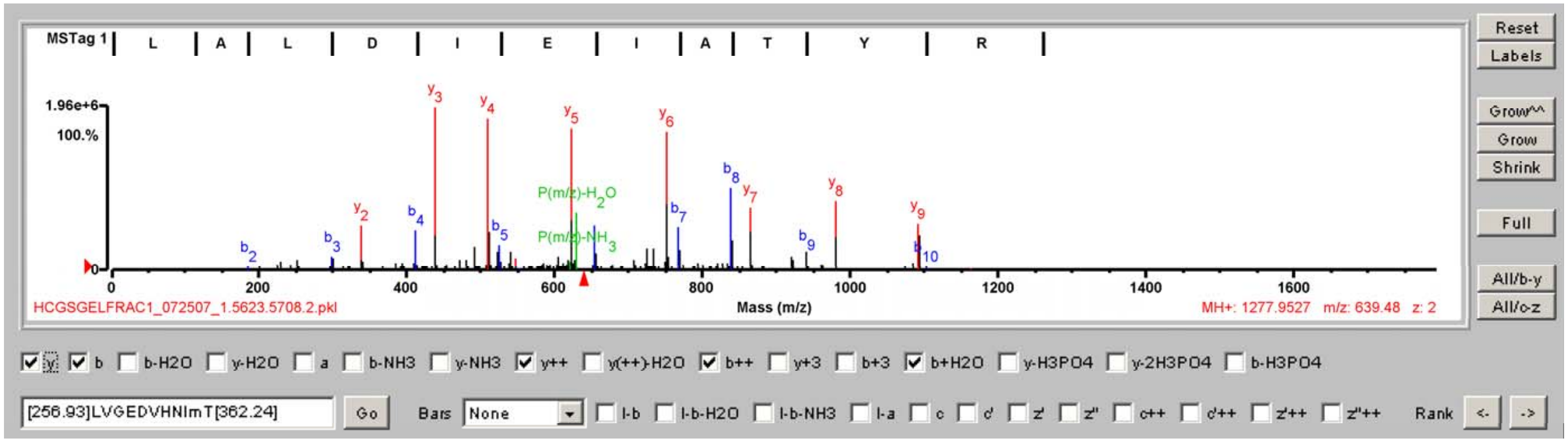

Golgi Apparatus Protein 1

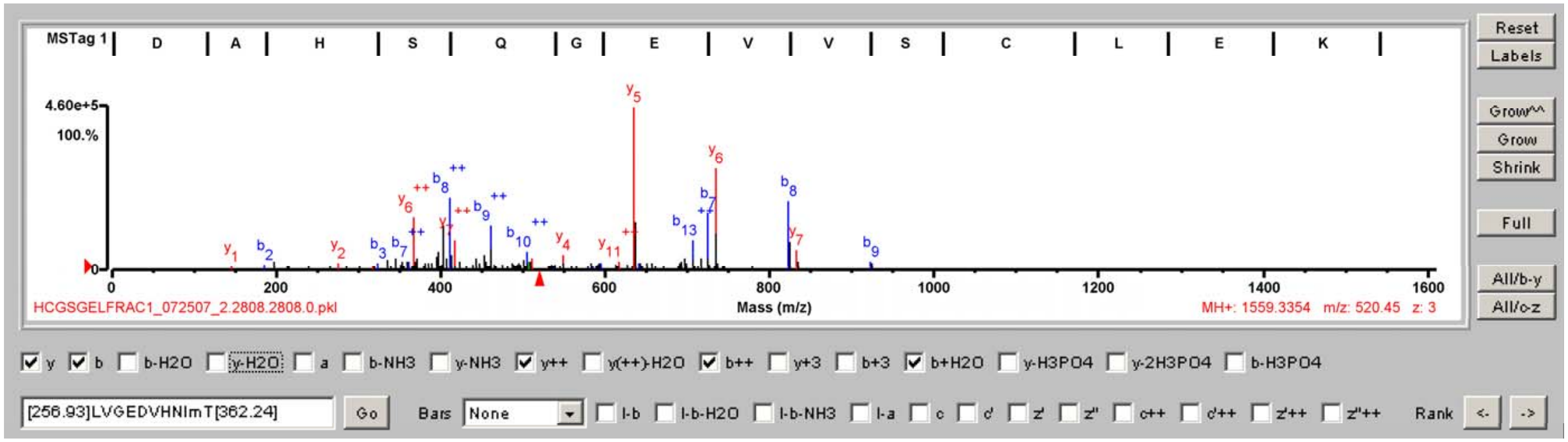

Guanine Nucleotide Binding Protein Alpha 12, 14, Activating GNA12, GNA14, GNAL

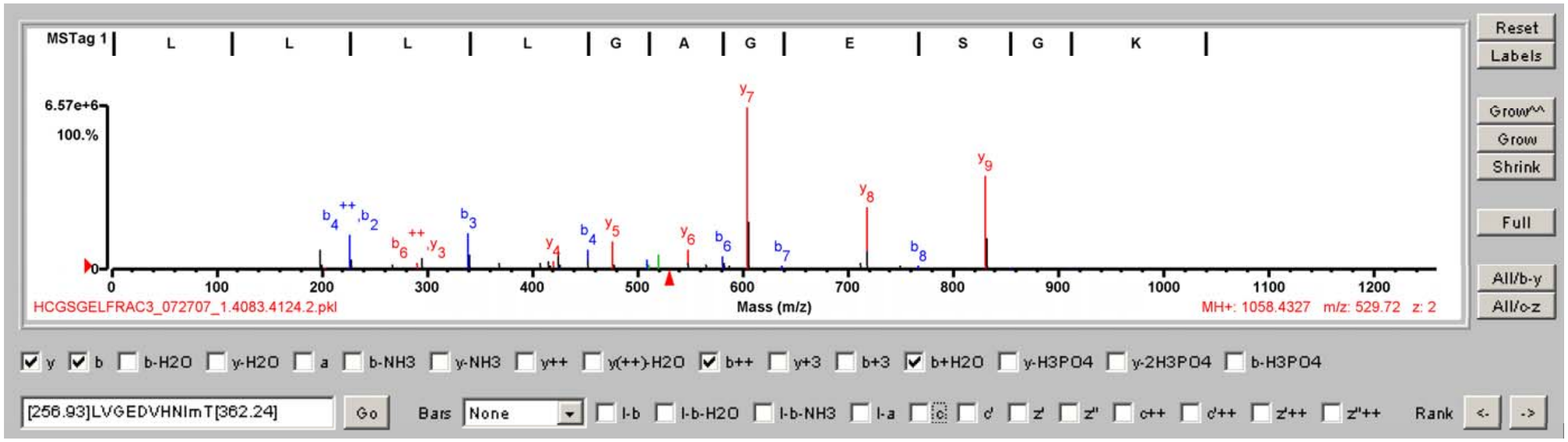

Also matches Guanine Nucleotide Binding Protein Alpha Transducing 3, Polypeptide 1 and Polypeptide 2 GNAT1, GNAT2, GNAT3

Guanine Nucleotide Binding Protein Gamma 4 GNG4

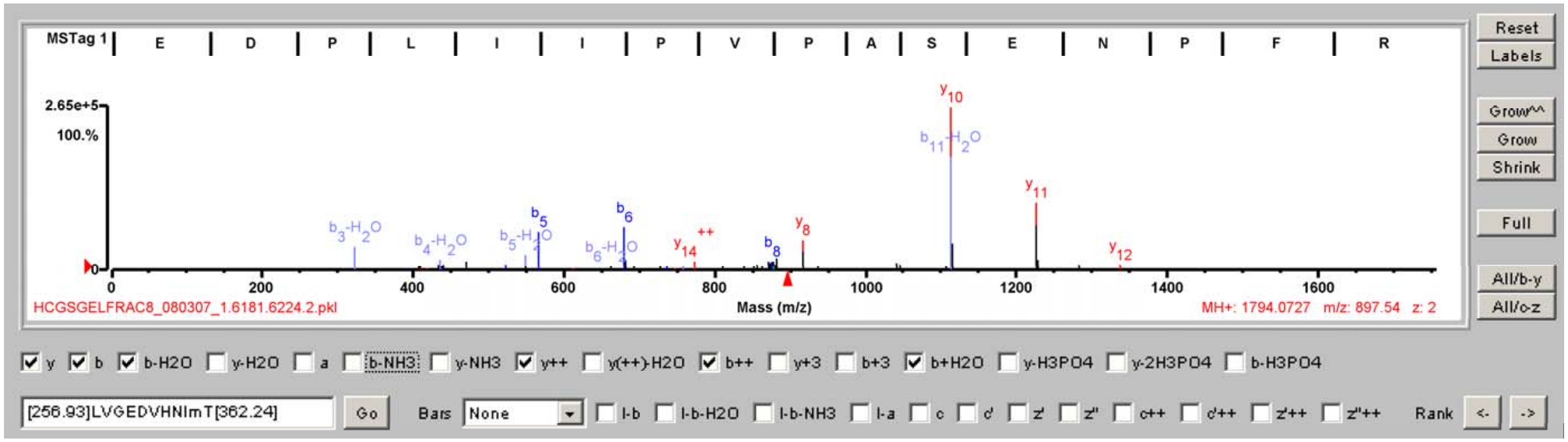

Heat Shock 70kDa Protein 6 HSPA6

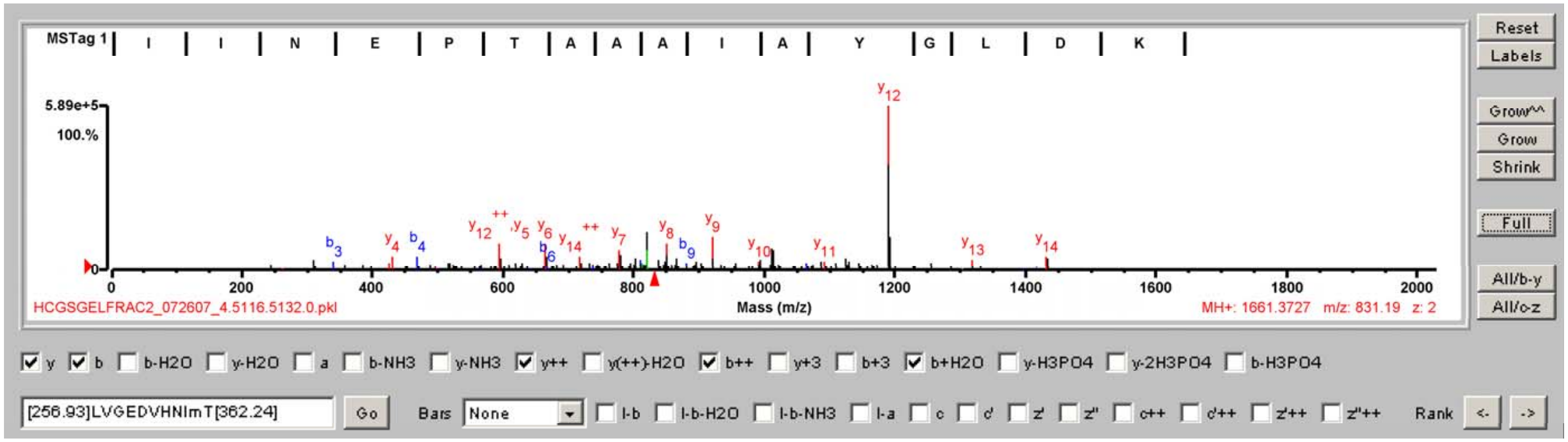

HtrA Serine Peptidase 2 Isoform 1, 2 HTRA2

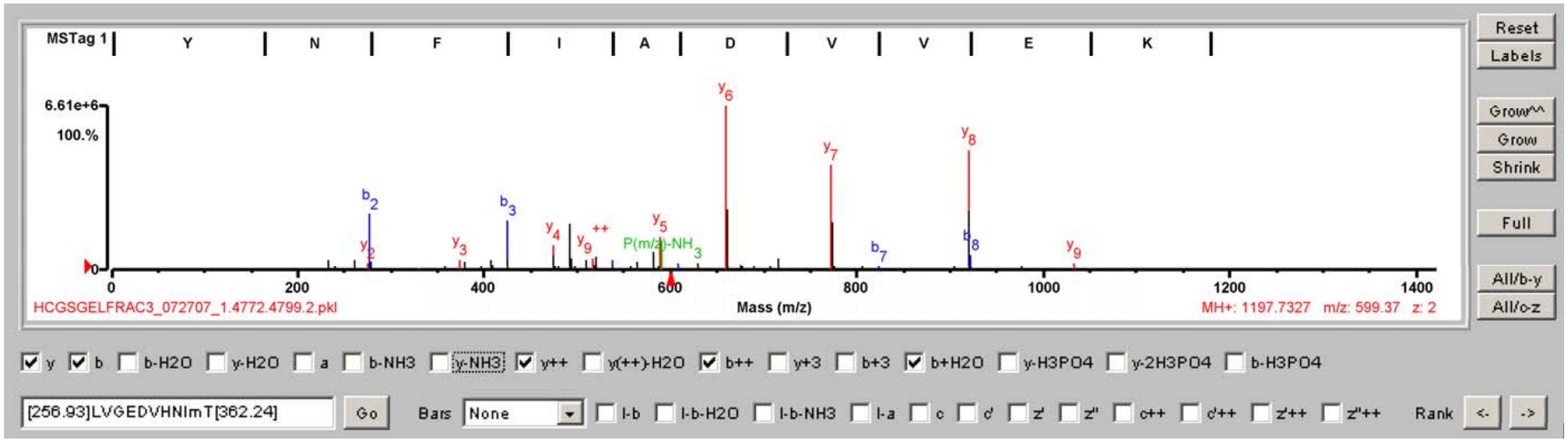

LOC196463 DDHD2

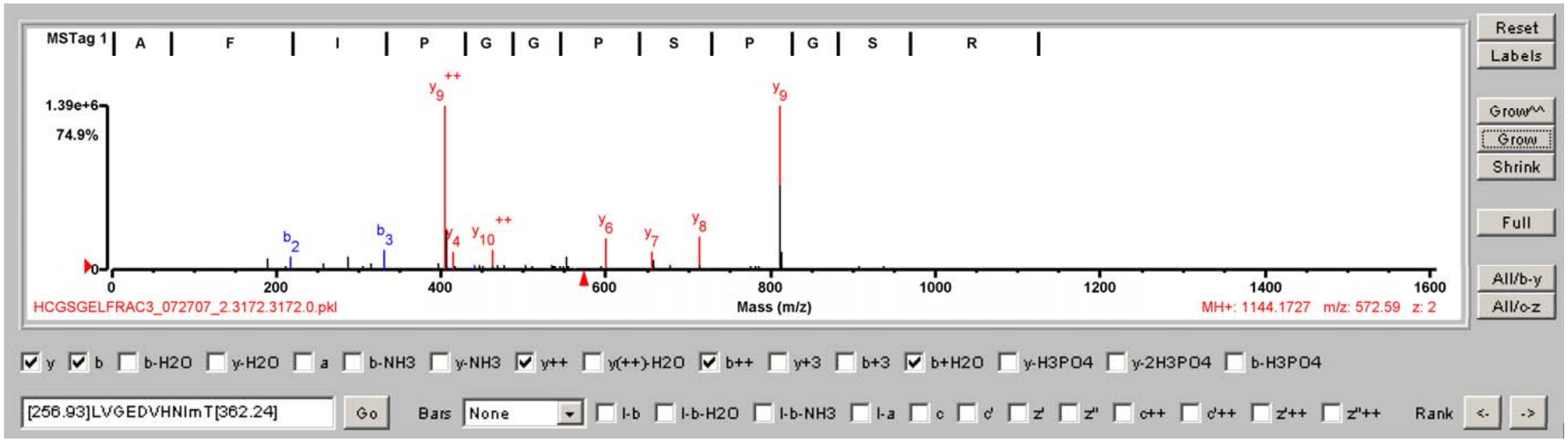

LOC643314 KIAA0754

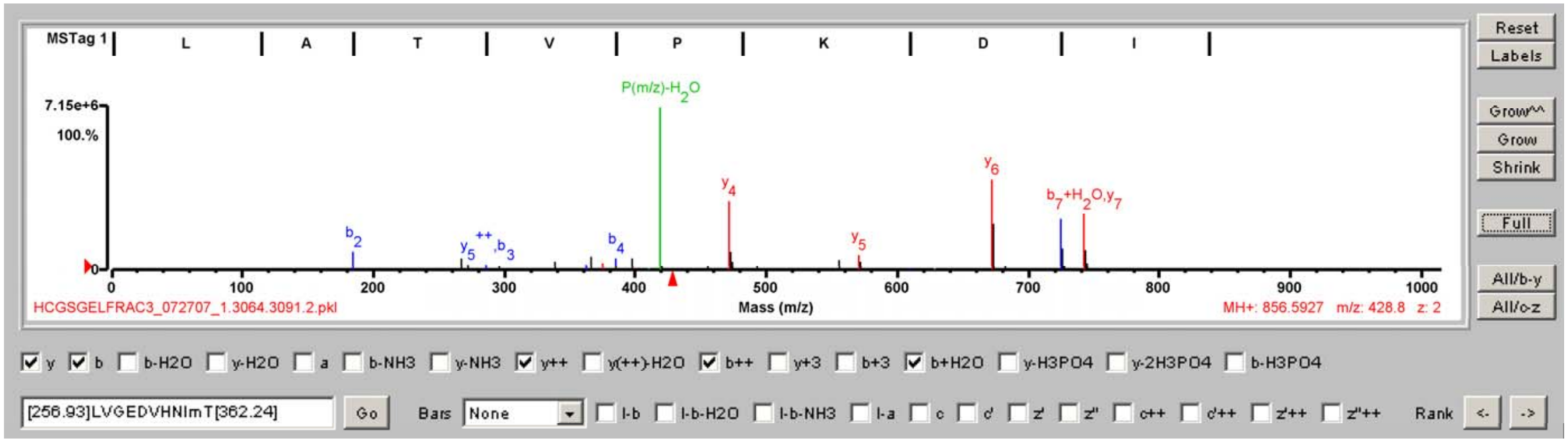

LOC79802 HHIPL2

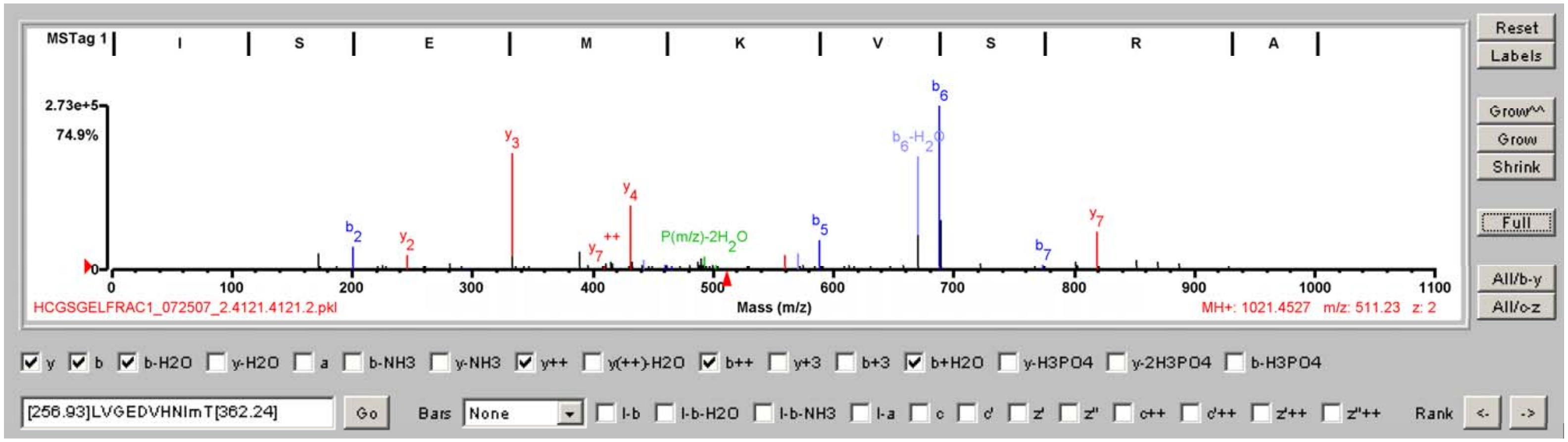

Iduronate-2-Sulfatase Isoform A Precursor IDS

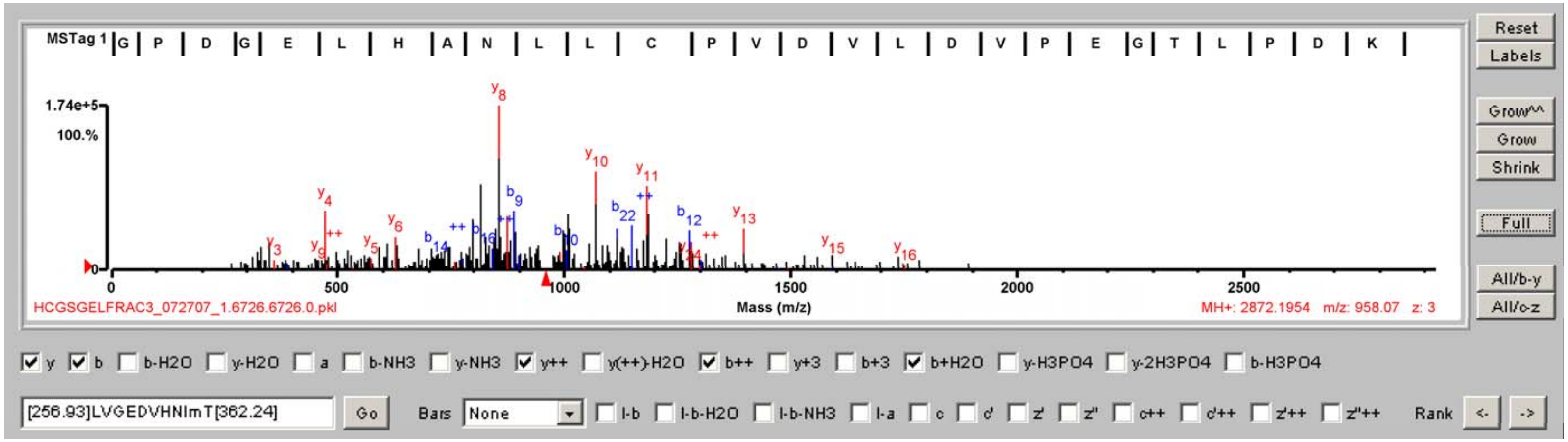

Lactate Dehydrogenase A LDHA

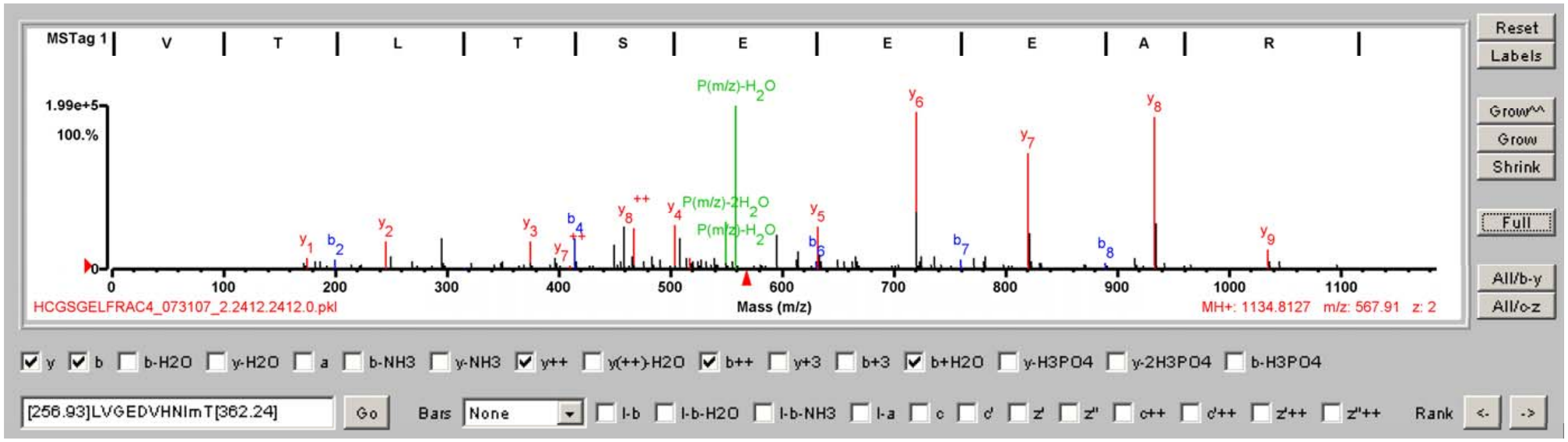

Laminin Alpha 1 Precursor LAMA1

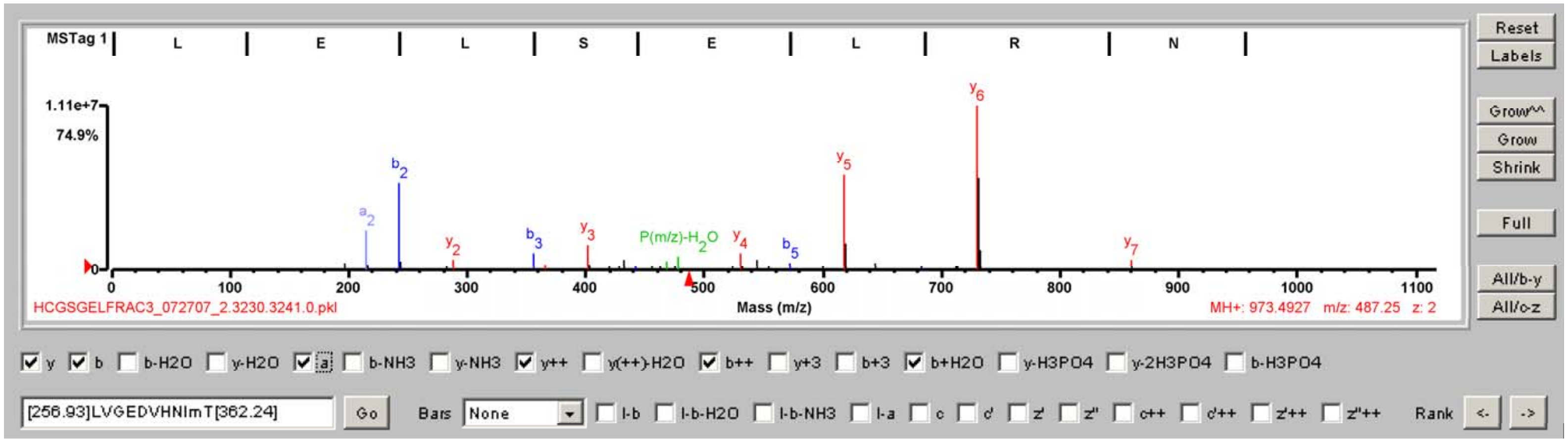

Microtubule-Associated Protein 2 Isoform 1 MAP2

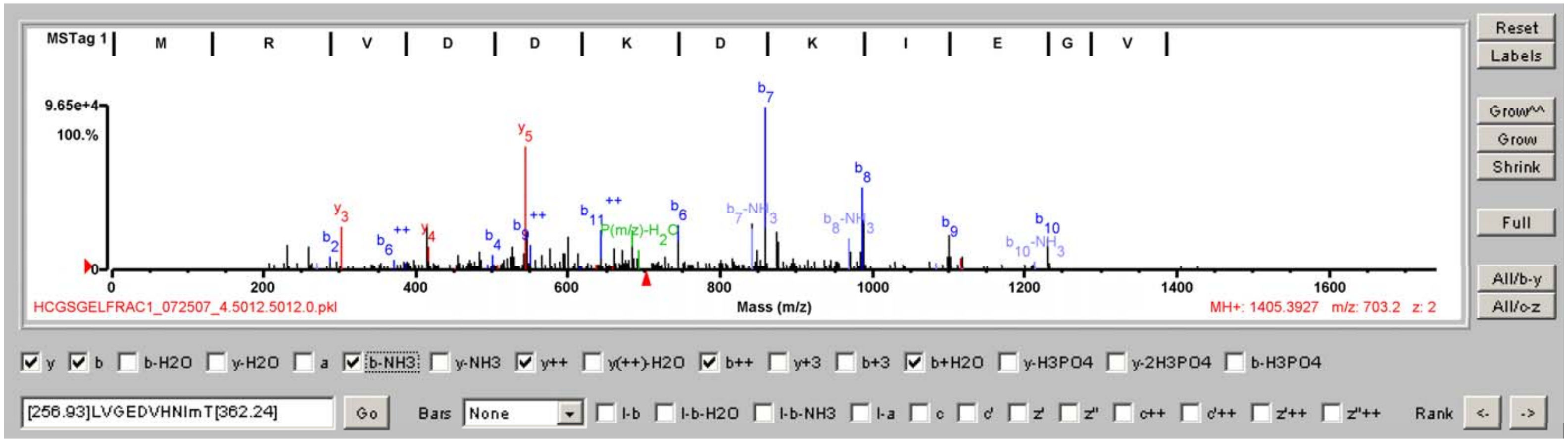

N-Ethylmaleimide-Sensitive Factor NSF

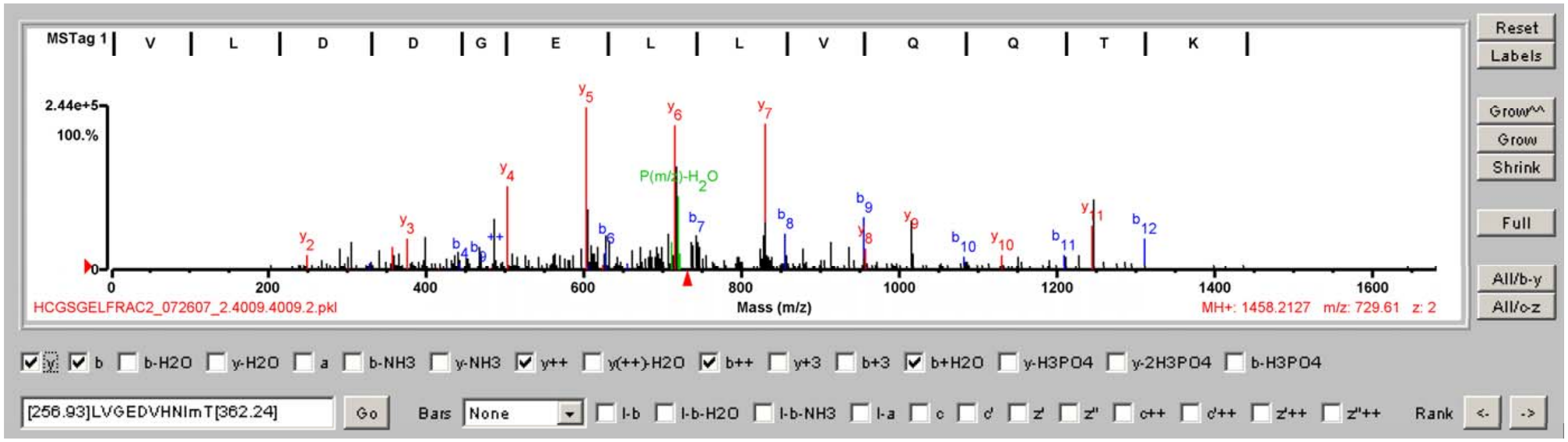

Peptidylprolyl Isomerase A-Like 4 PPIAL4G

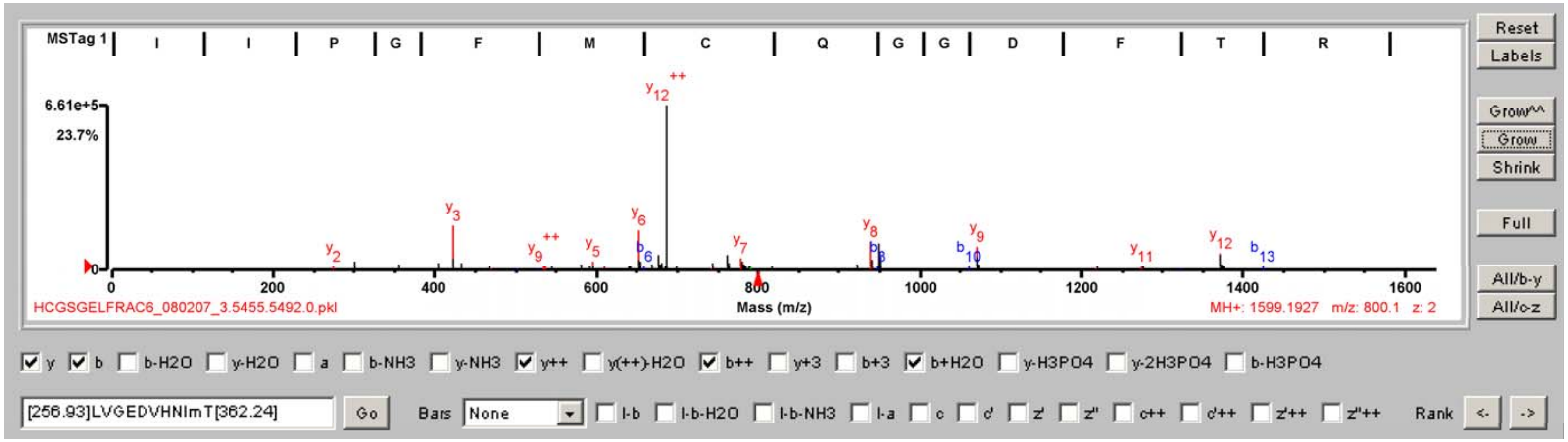

Peroxiredoxin 2 Isoform C PRDX2

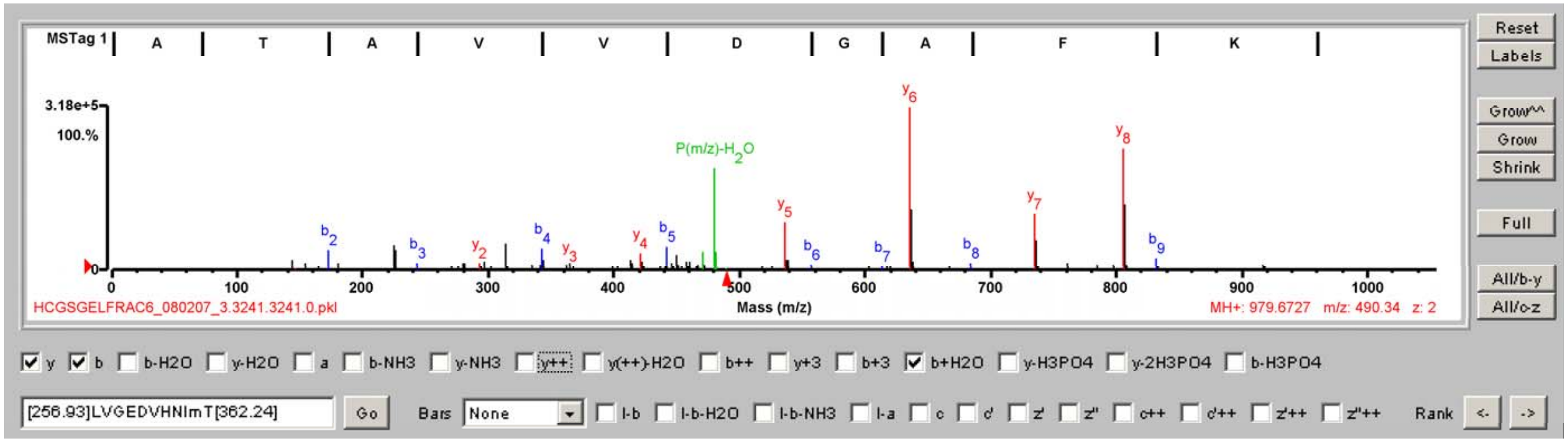

Phosphoribosyl Pyrophosphate Amidotransferase Preprotein PPAT

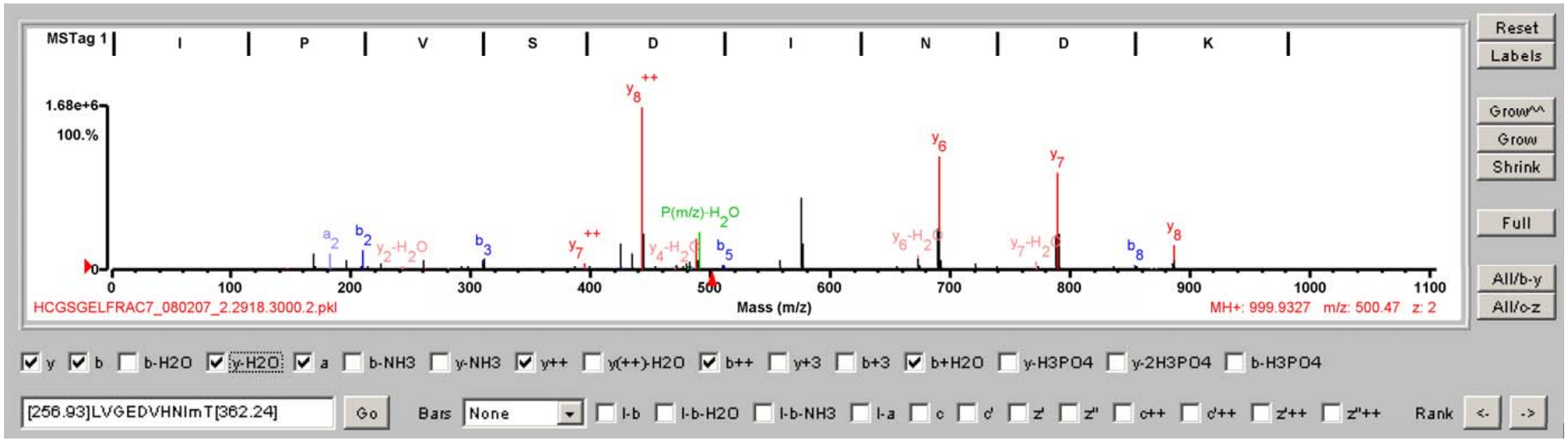

Similar to B-Cell Receptor Associated Protein 31 PCAP31

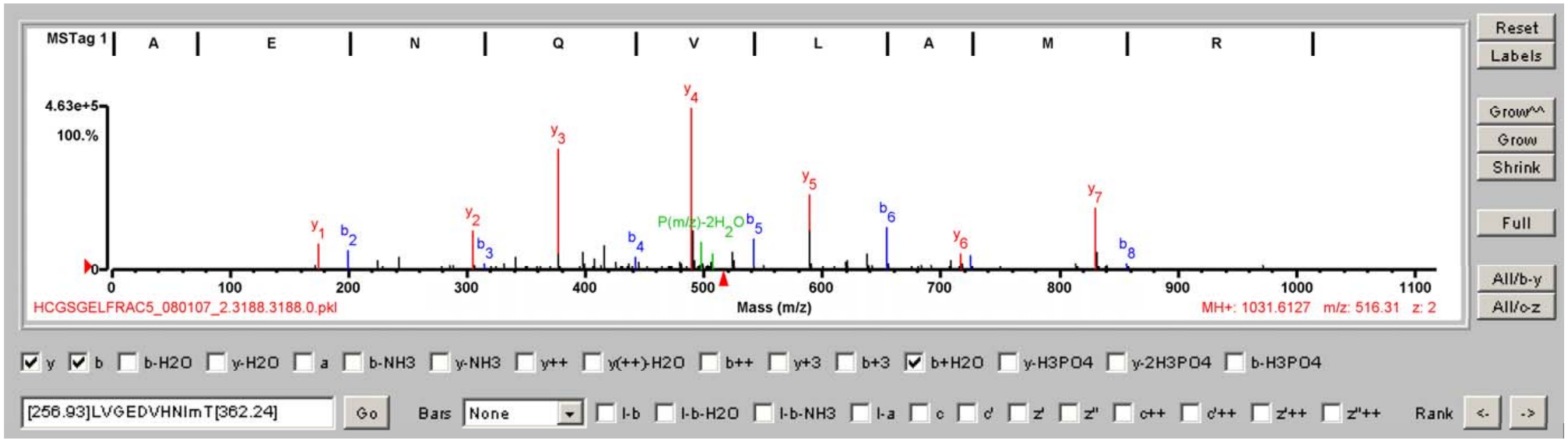

Proenkephalin PENK

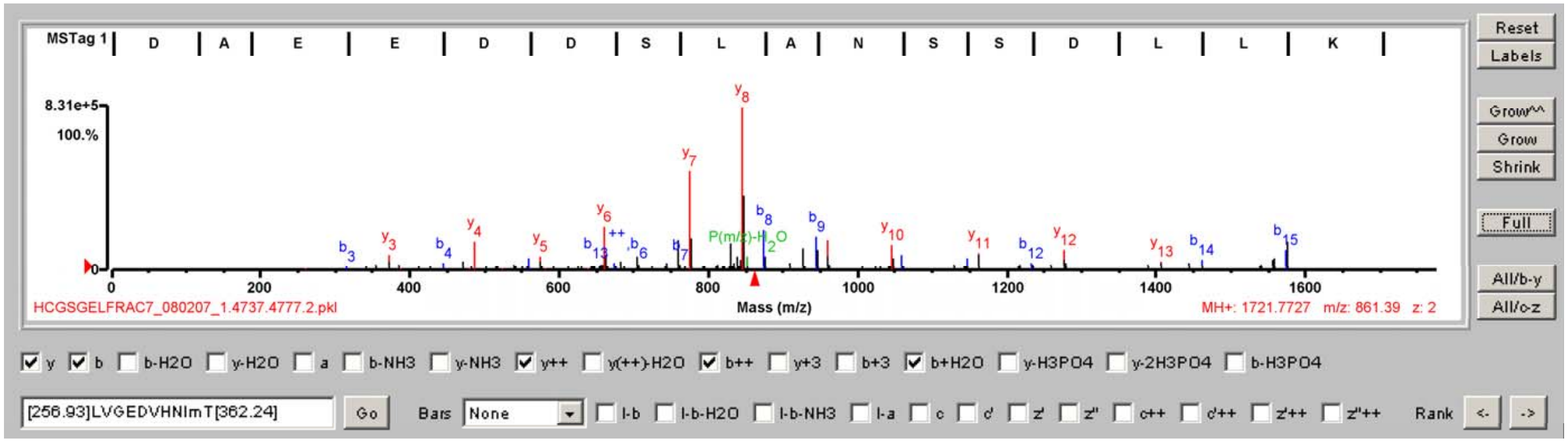

Proteolipid Protein 2 PLP2

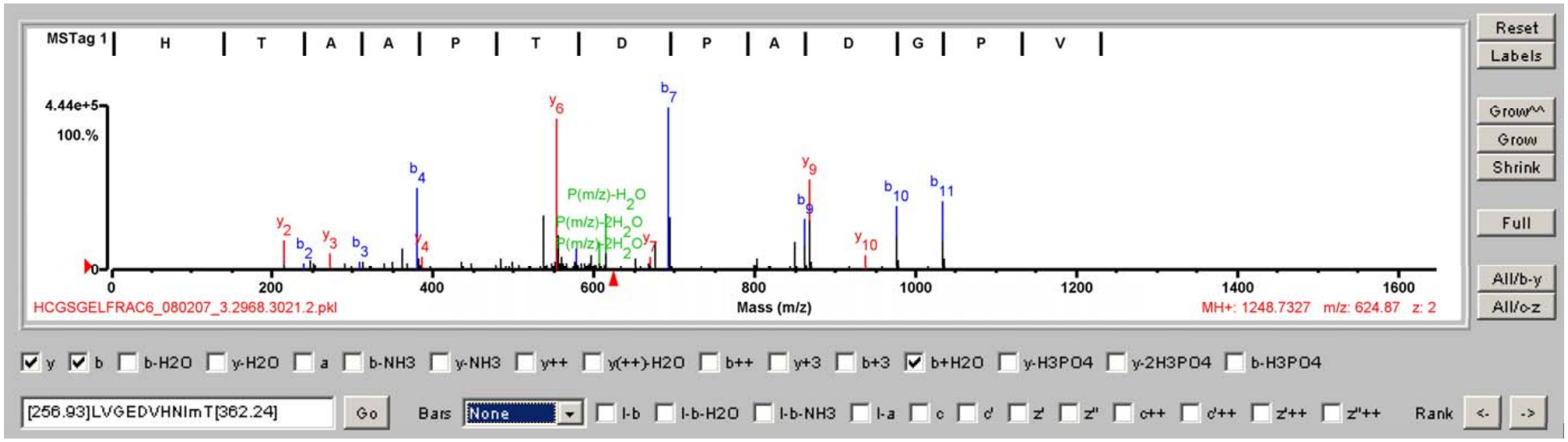

Receptor Accessory Protein 5 REEP5

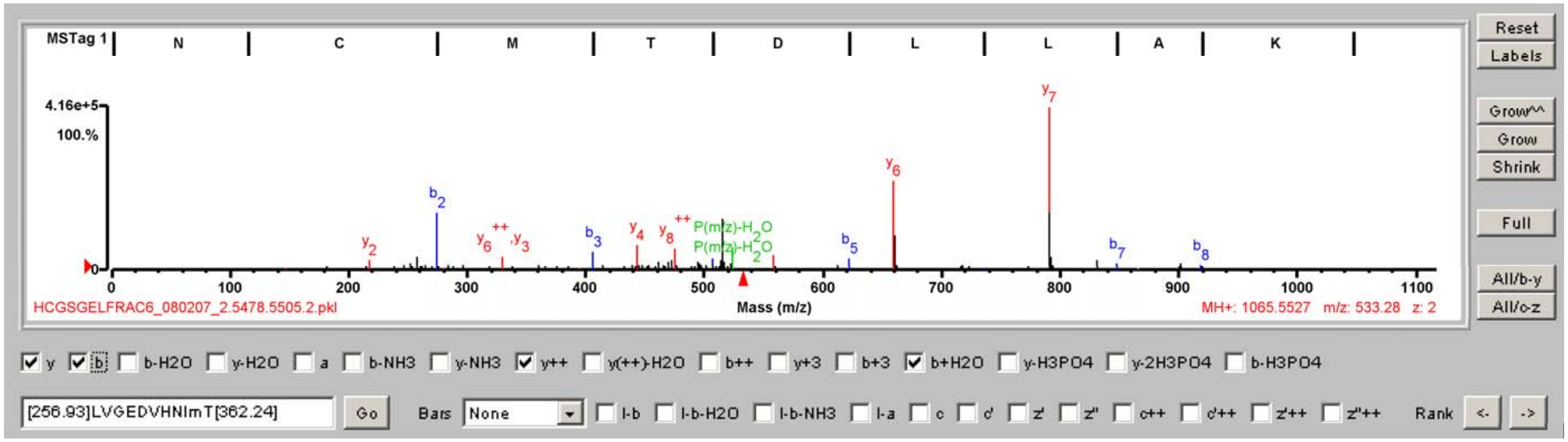

Reticulon 4, Isoforms A, D RTN4

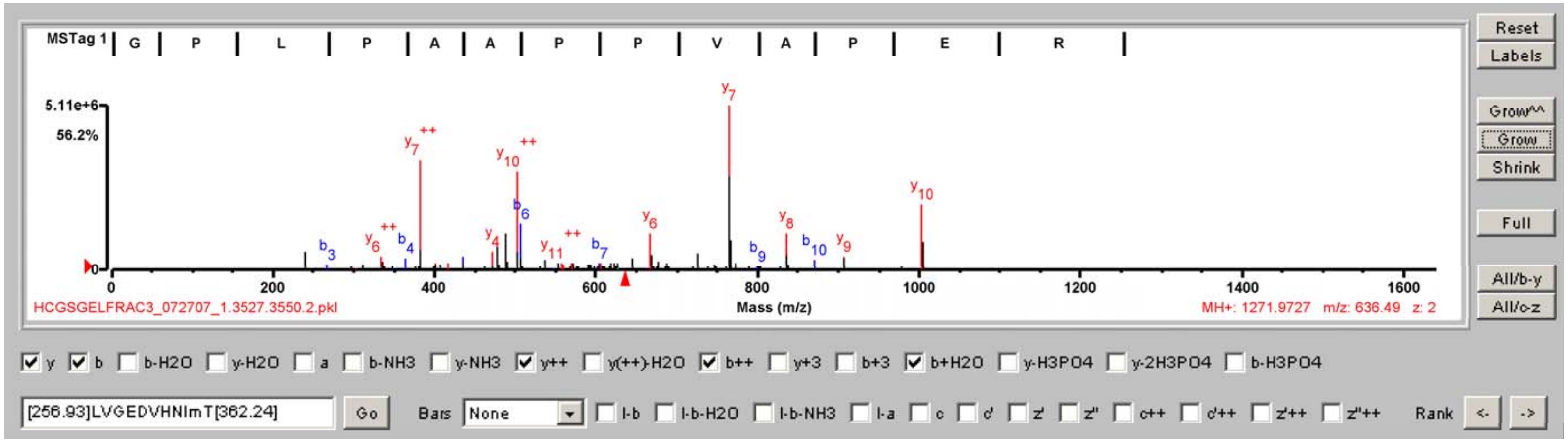

Sparc/Osteonectin SPOCK1

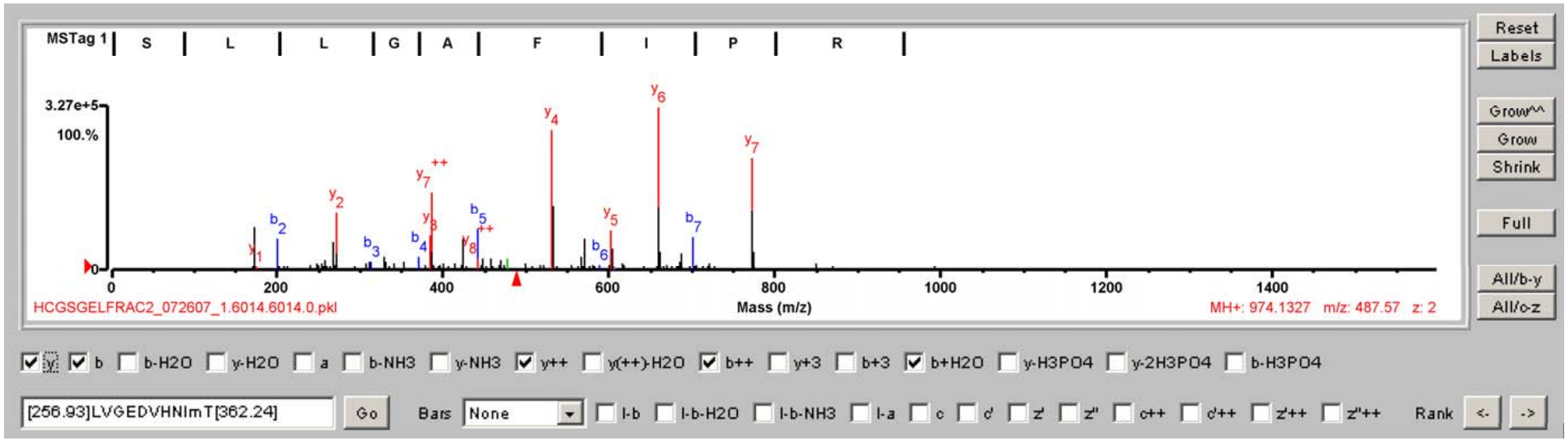

Spectrin Beta Non-Erythrocytic 1 Isoform 1 SPTBN1

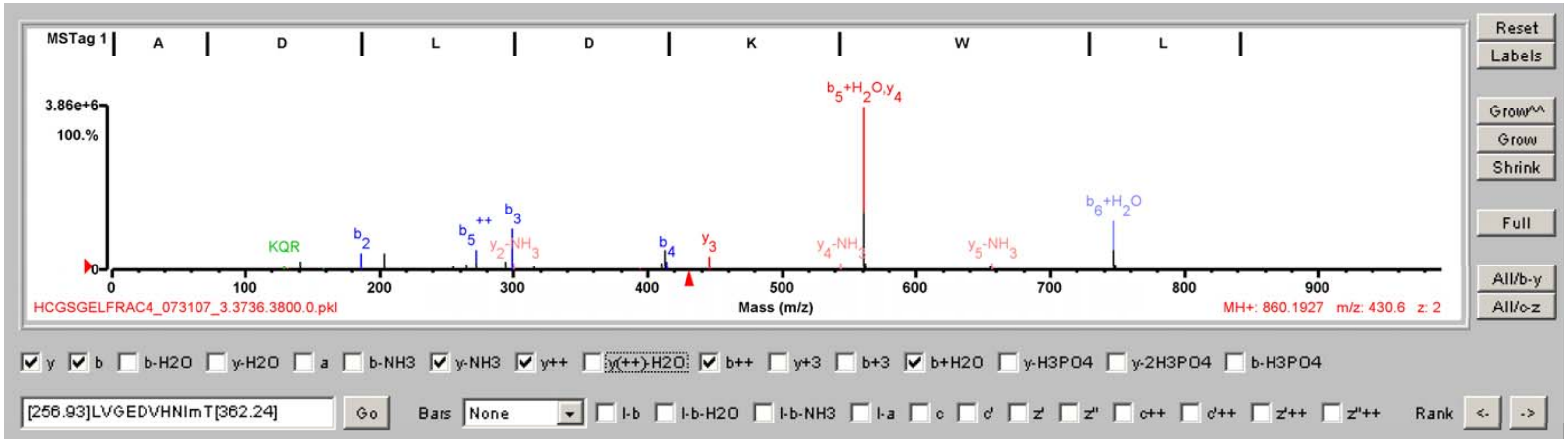

Spectrin Beta Isoform A SPTB

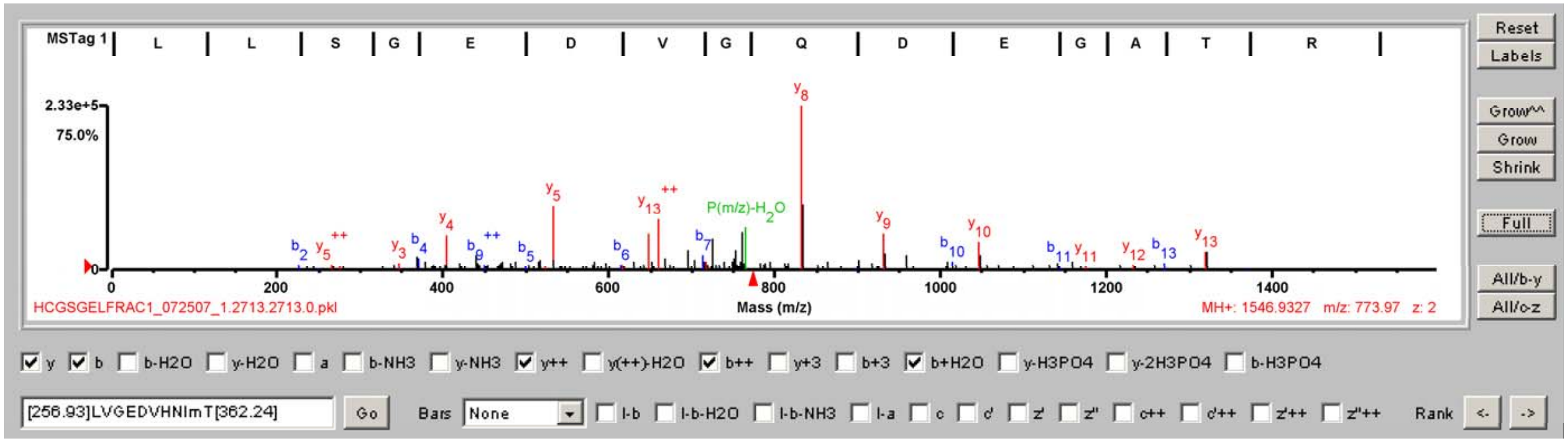

Stanniocalcin 1 Precursor STC1

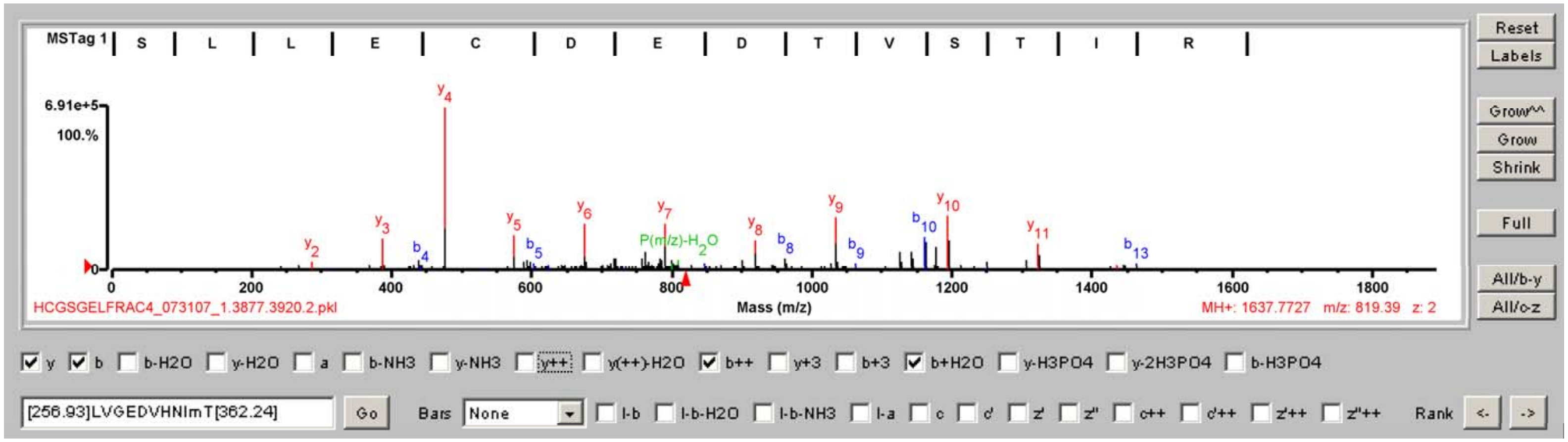

Stratifin/Tyrosine-3-Monooxygenase SFN/YWHAB, YWHAG, others

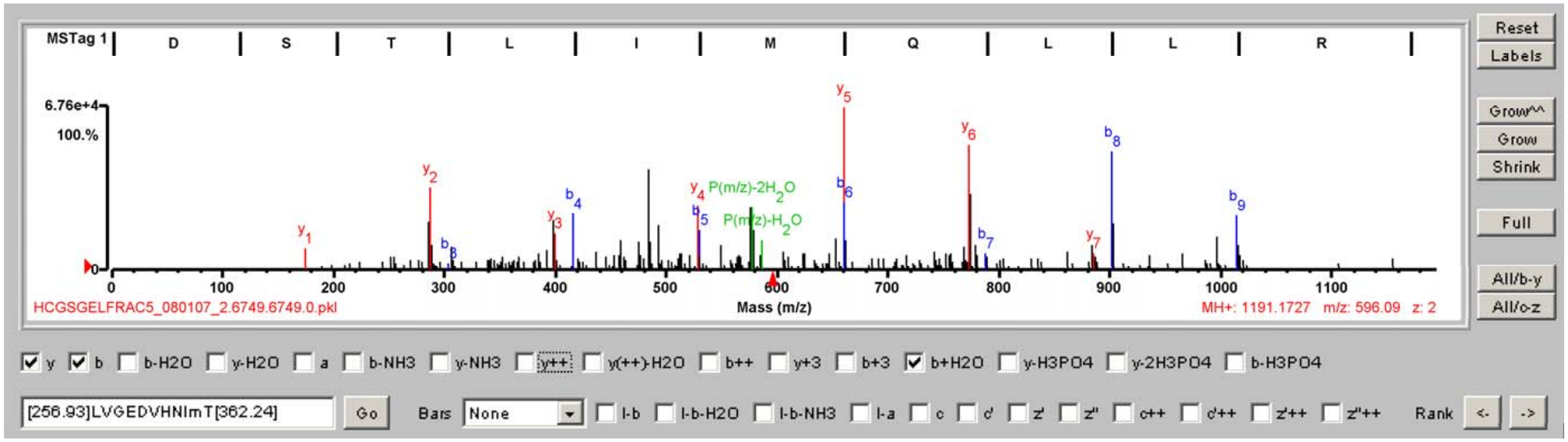

Synaptotagmin V SYT5

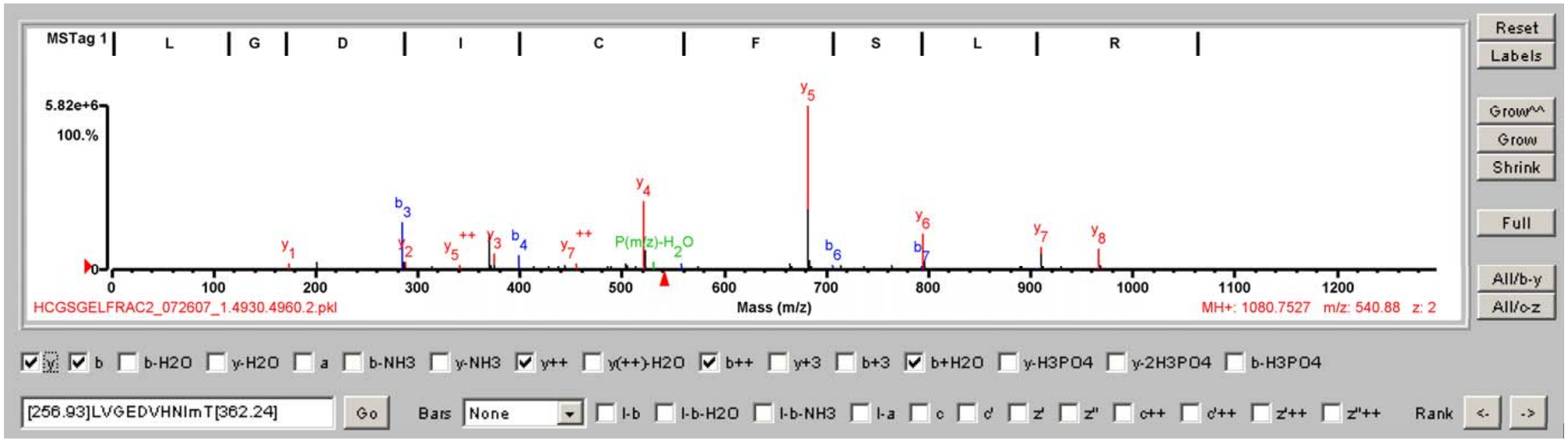

Tomosyn STXBP5

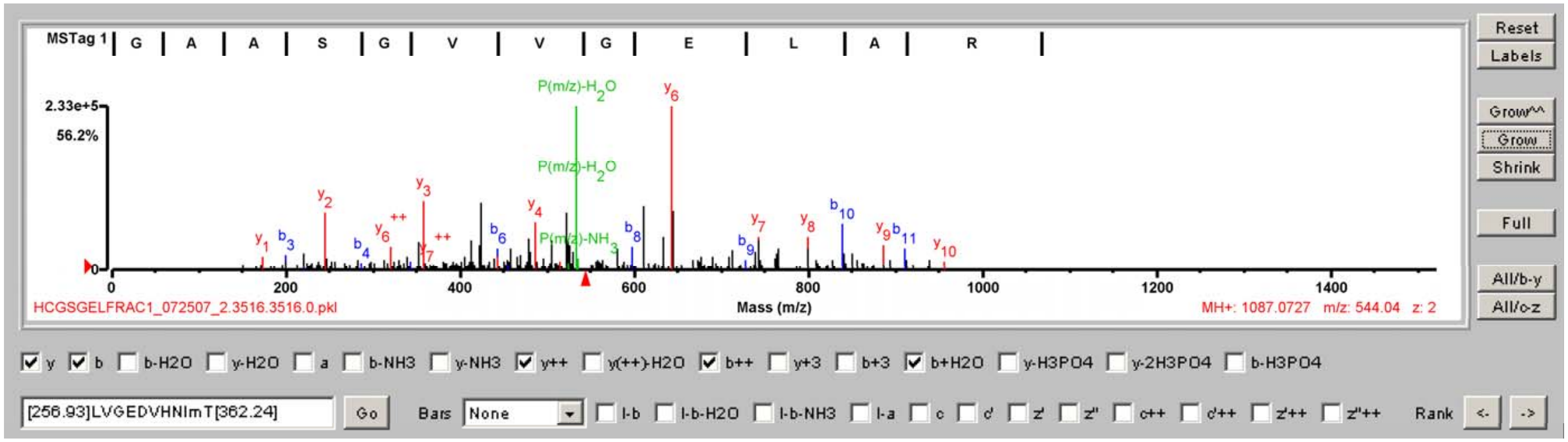

Transcobalamin II Precursor TCN2

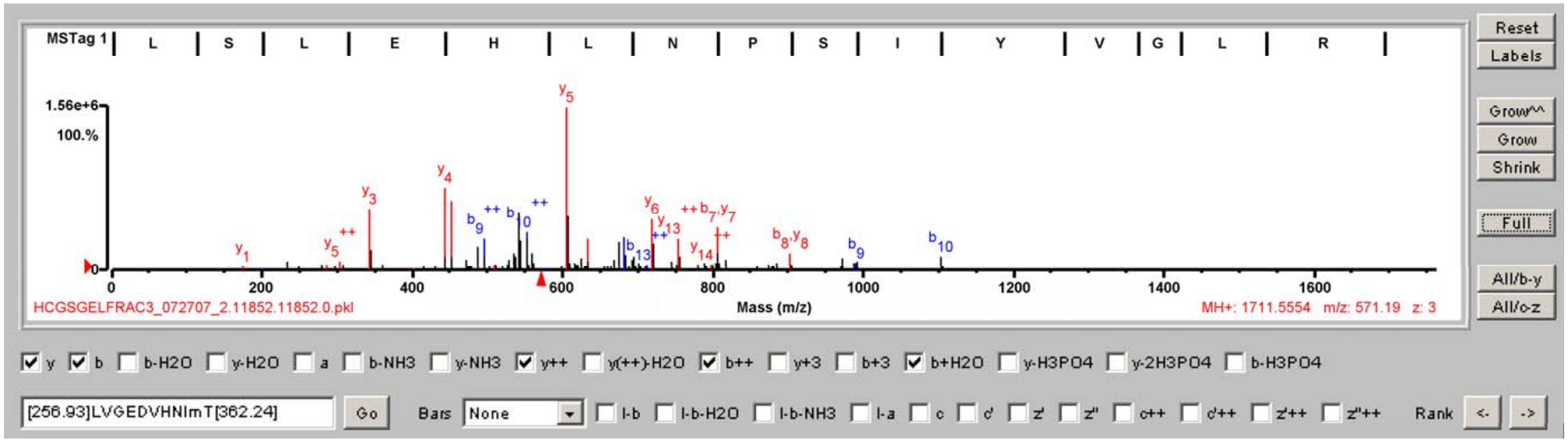

V-Ha-RAS Harvey Rat Sarcoma Viral Oncogene Homolog Isoform 1 HRAS

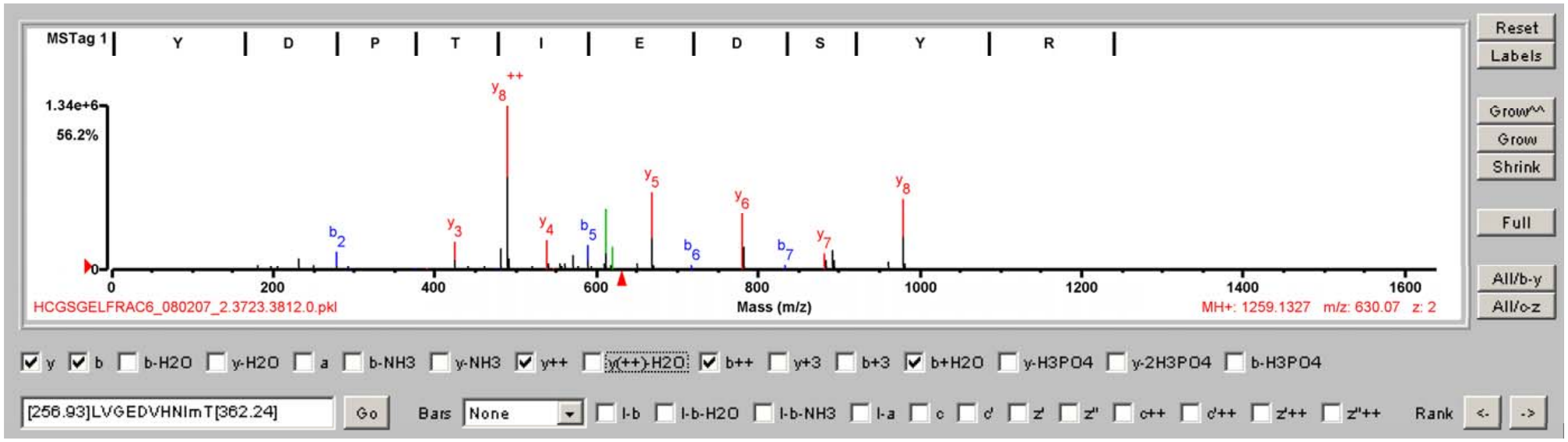

Vesicle-Associated Membrane Protein 2, 3 VAMP2, VAMP3

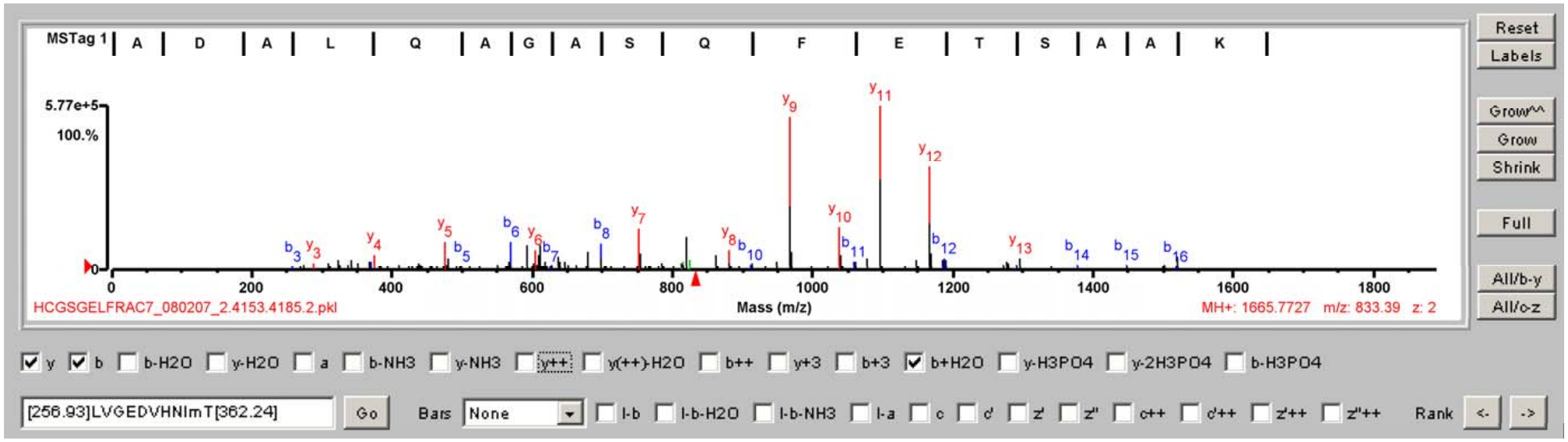

Supplement: Table S1 — Soluble proteins identified in human dense core secretory vesicles. Addendum: MS/MS spectra of single peptide identifications for soluble DCSV proteins. (PDF) [file pone.0041134.s004.pdf]
