# Supplementary material for: The Protein Architecture of Human Secretory Vesicles Reveals Differential Regulation of Signaling Molecule Secretion by Protein Kinases
Source: PLoS One. 2012 Aug 16;7(8):e41134. doi: 10.1371/journal.pone.0041134 (PMC3420874; doi:10.1371/journal.pone.0041134)

Table S2. Membrane Proteins Identified in Human Dense Core Secretory Vesicles (DCSV)

Protein Description	Accession no.	HGNC symbol	Peptide sequences identified	# of Tryptic Peptides	Protein MW	Protein pI	Peptide Score	Peptide % SPI	Peptide pI	Fragment Ions	Parent Charge
2,4-dienoyl CoA reductase 1 precursor	4503301	DECR1	EQWDTIEELIR	3	36067.9	9.35	12.67	80	4	25	2
			FNVIQPGPIK	3	36067.9	9.35	12.61	84.3	8.75	25	2
			VAFITGGGTGLGK	3	36067.9	9.35	12.78	75.7	8.72	25	2
3-hydroxyisobutyrate dehydrogenase	23308751	HIBADH	IITMLPTSINAIEAYSGANGILK	4	35329.1	8.38	14.76	72.4	6	25	3
			MGAVFMDAPVSGGVGAAR	4	35329.1	8.38	15.98	84.8	5.59	25	2
			SPILLGSLAHQIYR	4	35329.1	8.38	12.34	85.3	8.49	25	3
			TPVGFIGLGNMGNPMAK	4	35329.1	8.38	11.13	73.3	8.41	25	2
4-aminobutyrate aminotransferase precursor	38679946	ABAT	VDVEFDYDGPLMK	1	56439.3	8.17	13.09	78.1	3.84	25	2
acetyl-Coenzyme A acetyltransferase 1 precursor	4557237	ACAT1	FGNEVIPVTVTVK	4	45199.8	8.98	11.43	81.5	6	20	2
			LGSIAIQGAIEK	4	45199.8	8.98	14.7	93	6	20	2
			NEQDAYAINSYTR	4	45199.8	8.98	13.84	83.8	4.37	25	2
			TPIGSFLGSLSLLPATK	4	45199.8	8.98	19.67	91	8.41	25	2
aconitase 2 precursor	4501867	ACO2	IVYGHLDDPASQEIER	3	85425.9	7.36	18.69	86	4.31	25	3
			SQFTITPGSEQIR	3	85425.9	7.36	11.37	76.7	5.72	25	2
			WVVIGDENYGEGSSR	3	85425.9	7.36	14.02	83.7	4.14	25	2
actin related protein 2/3 complex subunit 3	5031597	ARPC3	DTDIVDEAIYYFK	3	20546.8	8.77	13.51	83.5	3.84	21	2
			EMYTLGITNFPIPGEPGFPLNAIYAK	3	20546.8	8.77	18.93	93.7	4.53	25	3
			LIGNMALLPIR	3	20546.8	8.77	10.22	73.9	9.75	25	2
actinin, alpha 1	4501891	ACTN1	DLLLDPAWEK	5	103058.1	5.25	13.26	95.3	4.03	15	2
			ELPPDQAEYCIAR	5	103058.1	5.25	11.71	82.4	4.14	25	2
			FAIQDISVEETSAK	5	103058.1	5.25	11	71.5	4.14	25	2
			LASDLLEWIR	5	103058.1	5.25	13.46	81.5	4.37	25	2
			VGWEQLLTTIAR	5	103058.1	5.25	15.74	86.3	5.97	25	2
actinin, alpha 2	4501893	ACTN2	DLLLDPAWEK	2	103854.4	5.31	13.26	95.3	4.03	15	2
			FAIQDISVEETSAK	2	103854.4	5.31	11	71.5	4.14	25	2
actinin, alpha 3	4557241	ACTN3	DLLLDPAWEK	2	103294.9	5.4	13.26	95.3	4.03	15	2
			FAIQDISVEETSAK	2	103294.9	5.4	11	71.5	4.14	25	2
actinin, alpha 4	12025678	ACTN4	DLLLDPAWEK	5	104854.6	5.27	13.26	95.3	4.03	15	2
			ELPPDQAEYCIAR	5	104854.6	5.27	11.71	82.4	4.14	25	2
			FAIQDISVEETSAK	5	104854.6	5.27	11	71.5	4.14	25	2
			LASDLLEWIR	5	104854.6	5.27	13.46	81.5	4.37	25	2
			VGWEQLLTTIAR	5	104854.6	5.27	15.74	86.3	5.97	25	2
adenylate cyclase activating polypeptide precursor	153266792	ADCYAP1	GVGGSLGGGAGDDAEPLSK	2	18835.5	9.83	15.65	83.5	4.03	25	2
			GVGGSLGGGAGDDAEPLSKR	2	18835.5	9.83	13.35	84.6	4.56	22	3
adenylate kinase 1	4502011	AK1	GQLVPLETVLDMLR	1	21635	8.73	20.92	96.6	4.37	25	2
adenylate kinase 3	19923437	AK3	AYEDQTKPVLEYYQK	9	25565.5	9.15	18.76	93.6	4.68	25	3
			AYQIDTVINLNVPFEVIK	9	25565.5	9.15	17.45	85.5	4.37	25	2
			GVLETFSGTETNK	9	25565.5	9.15	10.93	80.1	4.53	22	2
			IWPYVYAFLQTK	9	25565.5	9.15	17.33	78.4	8.5	25	2
			LIPDDVMTR	9	25565.5	9.15	11.16	77.6	4.21	25	2
			NLTQYSWLLDGFPR	9	25565.5	9.15	21.33	95.1	5.83	25	2
			PFEVIKQ	9	25565.5	9.15	11.53	86	6.43	25	2
			TLPQAEALDR	9	25565.5	9.15	12.74	90.5	4.37	20	2
			TVGIDDLTGEPLIQR	9	25565.5	9.15	15.08	82.3	4.03	25	2
ADP-ribosylation factor 1	4502201	ARF1	ILMVGLDAAGK	3	20696.9	6.31	15.46	85.3	5.84	25	2
			LGEIVTTIPTIGFNVETVEYK	3	20696.9	6.31	20.74	84.8	4.25	25	3
			QDLPNAMNAAEITDK	3	20696.9	6.31	17.47	78.7	4.03	25	2
ADP-ribosylation factor 5	4502209	ARF5	ILMVGLDAAGK	2	20696.9	6.3	15.46	85.3	5.84	25	2
			LGEIVTTIPTIGFNVETVEYK	2	20696.9	6.3	20.74	84.8	4.25	25	3
ADP-ribosylation factor-like 10C	8922601	ARL8B	DLPNALDEK	3	21539.2	8.67	17.02	87.6	4.03	25	2
			MNLSAIQDR	3	21539.2	8.67	16.73	89.6	5.59	25	2
			NELHNLLDKPQLQGIPVLVLGNK	3	21539.2	8.67	15.44	80.9	6.75	25	3
ADP-ribosylation factor-like 6 interacting protein	24308007	ARL6IP1	STNLLAAETASLEEQLQGWGEVMLMADK	1	23362.9	9.38	14.84	76.9	3.91	25	3
ADP-ribosylation-like factor 6 interacting protein 5	5453704	ARL6IP5	AWDDFFPGSDR	2	16570.3	9.77	13.07	80.1	3.93	25	2
			TPMGIVLDALEQQEEGINR	2	16570.3	9.77	15.64	72.5	4	25	3
AHNAK nucleoprotein isoform 1	61743954	AHNAK	GPGVDLPSVNLSMPK	4	629104.8	5.8	11.46	91.7	5.84	25	2
			ISMPDVDLHLK	4	629104.8	5.8	13.17	89.6	5.21	21	3
			VDINAPDVDVQGPDWHLK	4	629104.8	5.8	13.57	75.2	4.13	25	3
			VDVEVPDVSLEGPEGK	4	629104.8	5.8	11.65	72.4	3.83	25	2
albumin precursor	4502027	ALB	AVMDDFAAFVEK	11	69367.1	5.92	10.52	79.2	4.03	22	2
			FQNALLVR	11	69367.1	5.92	14.45	92.9	9.75	25	2
			FYAPELLFFAK	11	69367.1	5.92	12.98	77.1	6	25	2
			HPYFYAPELLFFAK	11	69367.1	5.92	23.14	91.1	6.75	25	3
			KVPQVSTPTLVEVSR	11	69367.1	5.92	20.41	91.7	8.75	25	3
			LVNEVTEFAK	11	69367.1	5.92	14.02	89.3	4.53	25	2
			QNCELFEQLGEYK	11	69367.1	5.92	20.39	96.7	4.25	25	2
			RHPDYSVVLLLR	11	69367.1	5.92	15.47	87.4	8.75	25	3
			RPCFSALEVDETYVPK	11	69367.1	5.92	22.54	96.4	4.68	25	3
			VFDEFKPLVEEPQNLIK	11	69367.1	5.92	17.62	90.9	4.41	25	3
			YLYEIAR	11	69367.1	5.92	11.46	97.2	6	13	2
aldolase A	4557305	ALDOA	GILAADESTGSIAK	4	39420.2	8.3	21.56	94.1	4.37	25	2
			IGEHTPSALAIMENANVLAR	4	39420.2	8.3	16.61	89.4	5.4	25	3
			PYQYPALTPEQK	4	39420.2	8.3	14.68	82.4	6.41	25	2
			YTPSGQAGAAASESLFVSNHAY	4	39420.2	8.3	12.25	70.6	5.24	25	3
alpha 1 actin precursor	4501881	ACTA1	AVFPSIVGR	4	42051.3	5.23	15.36	86.8	9.79	25	2
			DLTDYLMK	4	42051.3	5.23	12.7	89.8	4.21	25	2
			EITALAPSTMK	4	42051.3	5.23	12.54	87.9	6.1	25	2
			SYELPDGQVITIGNER	4	42051.3	5.23	21.01	92.3	4.14	25	2
alpha 1 type IV collagen preproprotein	148536825	COL4A1	GQPGSPGPVGAPGLPGEKGDHGFPGS	2	83654.4	8.55	14.42	78.2	5.32	25	3
			TRHSQTIDDPQCPSGTKILYHGYS	2	83654.4	8.55	12.25	72	6.61	25	3
alpha 1 type VII collagen precursor	4502961	COL7A1	GPNGAAGKAGDPGRDGLPGLR	2	138556	5.95	12.94	77.3	8.75	25	3
			IVARTDPLGPVRTVHVTQA	2	138556	5.95	12.1	83.8	9.61	25	3
alpha 2 globin	4504345	HBA1 HBA2	FLASVSTVLTSK	3	15257.6	8.72	12.77	81.5	8.75	25	2
			MFLSFPTTK	3	15257.6	8.72	13.42	96.6	8.5	19	2
			VGAHAGEYGAEALER	3	15257.6	8.72	21.18	96.8	4.75	25	3
alpha 3 type VI collagen isoform 1 precursor	55743098	COL6A3	SQERGPKGETGDLGPMGVPGRD	2	94132.4	6.27	12.87	91.9	4.94	25	3
			VKVEFSLTDYGSKEKLV	2	94132.4	6.27	11.17	85.4	6.15	25	2
alpha 3 type VI collagen isoform 2 precursor	55743100	COL6A3	SQERGPKGETGDLGPMGVPGRD	2	94132.4	6.12	12.87	91.9	4.94	25	3
			VKVEFSLTDYGSKEKLV	2	94132.4	6.12	11.17	85.4	6.15	25	2
alpha 3 type VI collagen isoform 3 precursor	55743102	COL6A3	SQERGPKGETGDLGPMGVPGRD	2	94132.4	6.47	12.87	91.9	4.94	25	3
			VKVEFSLTDYGSKEKLV	2	94132.4	6.47	11.17	85.4	6.15	25	2
alpha 4 type IV collagen precursor	116256356	COL4A4	DDGLPGPPGPKGPRGLPGF	3	164038.5	8.9	13.86	79	5.96	23	3
			GHSCERGAPGIPGQPGLPGYPGSPGAPGGK	3	164038.5	8.9	12.45	87.2	8.21	25	3
			VTYPGRHGPPGFDGP	3	164038.5	8.9	13.58	70	6.71	25	3
alpha glucosidase II alpha subunit isoform 2	38202257	GANAB	FSFSGNTLVSSSADPEGHFETPIWIER	3	106874.5	5.73	16.52	74.6	4.4	25	3
			QYASLTGTQALPPLFSLGYHQSR	3	106874.5	5.73	16.87	72.2	8.6	25	3
			YRVPDVLVADPPIAR	3	106874.5	5.73	14.61	81.6	5.96	25	3
alpha glucosidase II alpha subunit isoform 3	88900491	GANAB	FSFSGNTLVSSSADPEGHFETPIWIER	3	109438.5	5.82	16.52	74.6	4.4	25	3
			QYASLTGTQALPPLFSLGYHQSR	3	109438.5	5.82	16.87	72.2	8.6	25	3
			YRVPDVLVADPPIAR	3	109438.5	5.82	14.61	81.6	5.96	25	3
alpha-1,3(6)-mannosylglycoprotein beta-1,6-N-acetyl-glucosaminyltransferase	4505167	MGAT5	NVVDGPYAGVMTAYDLK	3	15585.3	8.41	13.5	77.3	4.21	24	2
			TLAVLLDNILQR	3	15585.3	8.41	19.78	94.4	5.5	25	2
			VLDSFGTEPEFNHANYAQSK	3	15585.3	8.41	16.85	87.6	4.65	25	3
annexin 5	4502107	ANXA5	GLGTDEESILTLLTSR	4	35936.9	4.94	16.16	88	4.14	25	2
			GTVTDFPGFDER	4	35936.9	4.94	13.11	79.4	4.03	25	2
			SEIDLFNIR	4	35936.9	4.94	12.11	83.9	4.37	20	2
			SIPAYLAETLYYAMK	4	35936.9	4.94	18.04	86.6	5.72	25	2
annexin A2 isoform 1	50845388	ANXA2 ANXA2P	AEDGSVIDYELIDQDAR	8	28766.3	8.53	16.3	81.7	3.66	25	2
			DALNIETAIK	8	28766.3	8.53	12.69	77.4	4.37	25	2
			DIISDTSGDFR	8	28766.3	8.53	12.57	74	3.93	25	2
			LSLEGDHSTPPSAYGSVK	8	28766.3	8.53	14.23	83.7	5.32	25	3
			QDIAFAYQR	8	28766.3	8.53	13.56	87.1	5.83	25	2
			SALSGHLETVILGLLK	8	28766.3	8.53	12.51	96.8	6.47	16	3
			SLYYYIQQDTK	8	28766.3	8.53	17.91	86.7	5.55	25	2
			SYSPYDMLESIR	8	28766.3	8.53	16.31	89.1	4.37	25	2
annexin VI isoform 1	71773329	ANXA6	ALIEILATR	4	75873.7	5.42	18.76	91.7	6.05	24	2
			EDAQVAAEILEIADTPSGDK	4	75873.7	5.42	16.85	90.4	3.71	25	2
			SLEDALSSDTSGHFR	4	75873.7	5.42	12.04	78.5	4.53	25	3
			SLHQAIEGDTSGDFLK	4	75873.7	5.42	17.2	91.4	4.53	25	2
apolipoprotein H precursor	153266841	APOH	ATFGCHDGYSLDGPEEIECTK	5	38298.4	8.34	13.07	78.7	4.17	25	3
			FICPLTGLWPINTLK	5	38298.4	8.34	11.44	77.2	8.22	20	2
			KCSYTEDAQCIDGTIEVPK	5	38298.4	8.34	12.93	76.1	4.32	25	3
			KFICPLTGLWPINTLK	5	38298.4	8.34	16.62	85	9.32	25	3
			VCPFAGILENGAVR	5	38298.4	8.34	11.25	71.4	5.97	25	2
apolipoprotein O	13129148	APOO	GYIVIEDLWK	3	22284.8	9.18	13.94	75.6	4.37	25	2
			SQLEESISQLR	3	22284.8	9.18	19.41	82.2	4.53	25	2
			VDELSLYSVPEGQSK	3	22284.8	9.18	15.84	89.2	4.14	25	2
ARP1 actin-related protein 1 homolog A, centractin alpha	5031569	ACTR1A	AQYYLPDGSTIEIGPSR	1	70025.5	6.19	13.39	78.4	4.37	24	2
aspartate aminotransferase 2 precursor	73486658	GOT2	IAAAILNTPDLR	4	75873.7	9.13	15.41	89.7	5.84	25	2
			NLDKEYLPIGGLAEFCK	4	75873.7	9.13	18.04	85.5	4.68	25	3
			SSWWTHVEMGPPDPILGVTEAFK	4	75873.7	9.13	13.04	73.8	4.65	25	3
			TCGFDFTGAVEDISK	4	75873.7	9.13	12.07	77.7	4.03	25	2
ATP citrate lyase isoform 1	38569421	ACLY	AAHTANFLLN	2	120839.9	6.95	10.06	71.6	6.78	25	2
			FGHAGACANQASETAVAKNQALKEAG	2	120839.9	6.95	12.48	84.3	6.75	25	3
ATP synthase F0 subunit 8	17981857	MT-ATP6 MT-ATI	ICSLHSLPPQS	1	7991.7	9.92	14.89	85.9	6.73	25	2
ATP synthase, H+ transporting, mitochondrial F0 complex, subunit B1 precursor	21361565	ATP5F1	LDYHISVQNMMR	4	28908.8	9.37	17.45	86.9	6.74	25	3
			PVPPLPEYGGK	4	28908.8	9.37	11.27	86.9	6.42	25	2
			TGVTGPYVLGTGLILYALSK	4	28908.8	9.37	12.13	81.1	8.16	25	2
			YGLIPEEFFQFLYPK	4	28908.8	9.37	12.29	77.4	4.53	25	2
ATP synthase, H+ transporting, mitochondrial F0 complex, subunit d isoform a	5453559	ATP5H	AGLVDDFEK	4	18491.3	5.21	11.11	79.1	4.03	25	2
			LAALPENPPAIDWAYYK	4	18491.3	5.21	19.95	93.2	4.37	25	2
			NLIPFDQMTIEDLNEAFPETK	4	18491.3	5.21	18.32	89.2	3.83	25	2
			TIDWVAFAEIIPQNQK	4	18491.3	5.21	14.67	85.4	4.37	25	2
ATP synthase, H+ transporting, mitochondrial F0 complex, subunit d isoform b	51479152	ATP5H	AGLVDDFEK	4	15773.3	6.61	11.11	79.1	4.03	25	2
			LAALPENPPAIDWAYYK	4	15773.3	6.61	19.95	93.2	4.37	25	2
			NLIPFDQMTIEDLNEAFPETK	4	15773.3	6.61	18.32	89.2	3.83	25	2
			TIDWVAFAEIIPQNQK	4	15773.3	6.61	14.67	85.4	4.37	25	2
ATP synthase, H+ transporting, mitochondrial F0 complex, subunit E	6005717	ATP5I	ELAEDDSILK	3	7933.2	9.34	11.22	80.5	3.91	25	2
			MVPPVQVSPLIK	3	7933.2	9.34	13.89	87.1	8.5	25	2
			YSALFLGVAYGATR	3	7933.2	9.34	20.93	93	8.59	25	2
ATP synthase, H+ transporting, mitochondrial F0 complex, subunit F2 isoform 2a	4757812	ATP5J2	DFSPSGIFGAFQR	2	50436	9.7	16.15	80.5	5.84	25	2
			LGELPSWILMR	2	50436	9.7	14.88	86.9	6	25	2
ATP synthase, H+ transporting, mitochondrial F0 complex, subunit F2 isoform 2c	51479132	ATP5J2	DFSPSGIFGAFQR	2	130539.3	8.98	16.15	80.5	5.84	25	2
			LGELPSWILMR	2	130539.3	8.98	14.88	86.9	6	25	2
ATP synthase, H+ transporting, mitochondrial F0 complex, subunit G	51479156	ATP5L	LATFWYYAK	2	11428.5	9.64	11.02	74.6	8.5	25	2
			VELVPPTPAEIPR	2	11428.5	9.64	16.34	98.5	4.53	16	2
ATP synthase, H+ transporting, mitochondrial F1 complex, alpha subunit precursor	4757810	ATP5A1	TGTAEMSSILEER	2	50436	9.16	12.52	79.5	4.25	25	2
			VVDALGNAIDGK	2	50436	9.16	12.49	78.3	4.21	21	2
ATP synthase, H+ transporting, mitochondrial F1 complex, beta subunit precursor	32189394	ATP5B	AIAELGIYPAVDPLDSTSR	12	56560.2	5.26	15.17	78.9	4.03	25	2
			EGNDLYHEMIESGVINLK	12	56560.2	5.26	13.71	85.1	4.4	25	3
			FLSQPFQVAEVFTGHMGK	12	56560.2	5.26	16.15	91.5	6.75	25	3
			GFQQILAGEYDHLPEQAFYMVGPIEEAVAK	12	56560.2	5.26	12.89	84.9	4.25	25	3
			IMDPNIVGSEHYDVAR	12	56560.2	5.26	15.16	91.4	4.54	24	3
			LVLEVAQHLGESTVR	12	56560.2	5.26	15.46	87.8	5.4	25	3
			SLQDIIAILGMDELSEEDKLTVSR	12	56560.2	5.26	12.89	79.3	4.02	25	3
			TIAMDGTEGLVR	12	56560.2	5.26	19.22	92.6	4.37	25	2

			TVLIMELINNVAK	12	56560.2	5.26	19.61	91.2	5.66	25	2
			VALTGLTVAEYFR	12	56560.2	5.26	10.8	80	5.97	25	2
			VLDSGAPIKIPVGPETLGR	12	56560.2	5.26	21.78	91.7	6.04	25	3
			VVDLLAPYAK	12	56560.2	5.26	13.28	84.1	5.8	24	2
ATP synthase, H+ transporting, mitochondrial F1 complex, delta subunit precursor	4502297	ATP5D	AQAELVGTADEATR	1	17490	5.38	12.33	93	4.14	16	2
ATP synthase, H+ transporting, mitochondrial F1 complex, gamma subunit isoform L (liver) precursor	50345988	ATP5C1	IYGLGSLALYEK	1	32996.2	9.23	15	87.9	6	25	2
ATPase, aminophospholipid transporter (APLT), class I, type 8A, member 1	17978471	ATP8A1	IIYQAASPDEGALVR	1	131370.1	6.41	18.31	82.6	4.37	25	2
ATPase, Cu++ transporting, beta polypeptide isoform a	55743071	ATP7B	SMVLDHNIIPGL	2	94132.4	6.25	10.74	73.7	5.06	25	2
			VKSIEDRISNLKGIISM	2	94132.4	6.25	13.26	75	8.56	25	3
ATPase, H+ transporting, lysosomal 31kDa, V1 subunit E isoform 2	18087815	ATP6V1E2	HMMAFIEQEANEK	1	46276.7	8.79	15.6	85.5	4.75	25	3
ATPase, H+ transporting, lysosomal 42kDa, V1 subunit C1	4502315	ATP6V1C1	DFQYNEEEMK	3	43941.8	7.02	11.98	72.4	4	25	2
			ESVLRYGLPVNFQAML	3	43941.8	7.02	14.74	85.4	6.1	25	3
			VGTLDVLVGLSDELAK	3	43941.8	7.02	16.04	82.7	4.03	25	2
ATPase, H+ transporting, lysosomal 70kD, V1 subunit A, isoform 1	19913424	ATP6V1A	LAEMPADSGYPAYLGAR	4	68304.5	5.35	13.19	76.4	4.37	25	2
			SDYAQLLEDMQNAFR	4	68304.5	5.35	15.98	83.1	4.03	25	2
			TALVANTSNMPVAAR	4	68304.5	5.35	15.58	84.4	9.41	25	2
			TVGMLSNMIAFYDMAR	4	68304.5	5.35	10.36	72	5.5	25	2
ATPase, H+ transporting, lysosomal accessory protein 1 precursor	17136148	ATP6AP1	EVLTGNDEVIGQVLSTLK	2	52026.1	5.74	13.72	81.1	4.14	25	3
			LFGTTVTFK	2	52026.1	5.74	11.27	91.1	8.75	22	2
ATPase, H+ transporting, lysosomal accessory protein 2	15011918	ATP6AP2	AKNPASPYNLAYK	12	39008.2	5.76	16.01	81	9.53	25	3
			ANSVFEDLSVTLR	12	39008.2	5.76	16.74	89.5	4.37	25	2
			ASPYNLAYK	12	39008.2	5.76	12.38	92.7	8.54	25	2
			DHSPDLYSLELAGLDEIGK	12	39008.2	5.76	14.27	90.8	4.1	21	3
			FADDMYSLYGGNAVVELVTVK	12	39008.2	5.76	13.95	80.4	4.03	25	2
			LALPPGSVISYPLENAVPF	12	39008.2	5.76	12.32	73.2	4	25	2
			LFQENSVLSSLPLNSLSR	12	39008.2	5.76	17.36	82.6	6	25	3
			MDPGYDSIIYR	12	39008.2	5.76	12.19	83	4.21	25	2
			NPASPYNLAYK	12	39008.2	5.76	15.78	78.3	8.5	25	2
			PASPYNLAYK	12	39008.2	5.76	11.62	71.2	8.91	25	2
			SFDTSLIR	12	39008.2	5.76	14.99	93.8	5.55	25	2
			SLDSVANSIHSLFSEETPVVLQLAPSEER	12	39008.2	5.76	15.82	87	4.25	25	3
ATPase, H+ transporting, lysosomal V0 subunit a isoform 1	19913418	ATP6V0A1	ANIPIMDTGENPEVPFPR	9	95756.6	6.22	18.36	87.7	4.14	25	2
			ASLYPCPETPQER	9	95756.6	6.22	18.61	94.2	4.53	23	2
			DLNPDVNVFQR	9	95756.6	6.22	11.15	86.2	4.21	22	2
			DMIDLEANFEKIENELK	9	95756.6	6.22	12.03	91	4.08	19	3
			FLPFSFEHIR	9	95756.6	6.22	14.61	97	6.75	16	3
			IDDLQMVLNQTEDHR	9	95756.6	6.22	15.61	90.7	4.22	24	3
			KTQQFFDEMADPDLLEESSSLLEPSEMGR	9	95756.6	6.22	12.46	74.7	3.89	25	3
			NFLELTELK	9	95756.6	6.22	14.15	89	4.53	25	2
			QAEIENPLEDPVTGDYVHK	9	95756.6	6.22	17.75	92.8	4.17	25	3
ATPase, H+ transporting, lysosomal, V0 subunit d1	19913432	ATP6V0D1	AGVLSQADYLNLVQCETLEDLK	5	40329.3	4.89	17.48	91.8	3.92	25	3
			AYLESFYK	5	40329.3	4.89	11.49	89.7	6.04	22	2
			LHLQSTDYGNFLANEASPLTVSVIDDR	5	40329.3	4.89	13.66	80.3	4.22	25	3
			LYPEGLAQLAR	5	40329.3	4.89	17.56	92.4	6	25	2
			NVADYYPEYK	5	40329.3	4.89	14.64	86.4	4.37	25	2
barrier to autointegration factor 1	4502389	BANF1	DFVAEPMGEKPVGSLAGIGEVLGK	1	10058.6	5.81	18.17	73.4	4.41	25	3
bestrophin	4759310	BEST1	HKQLEKLSLPHNMFW	2	67684.1	6.43	14.62	82.4	8.61	19	3
			PDRLMSLVSGFVEGKDEQGRLL	2	67684.1	6.43	13.09	86.5	4.78	25	3
beta globin	4504349	HBB	EFTPPVQAAYQK	9	15998.5	6.74	16.34	90.4	6.1	25	2
			FFESFGDLSTPDAVMGNPK	9	15998.5	6.74	14.55	82.9	4.03	25	2
			GTFATLSELHCDK	9	15998.5	6.74	15.74	88.4	5.32	25	2
			LHVDPENFR	9	15998.5	6.74	13.19	86.9	5.32	25	2
			LLVVYPWTQR	9	15998.5	6.74	11.71	81.9	8.75	25	2
			SAVTALWGK	9	15998.5	6.74	17.18	87.2	8.47	25	2
			VLGAFSDGLAHLDNLK	9	15998.5	6.74	12.83	87.7	5.21	24	3
			VNVDEVGGEALGR	9	15998.5	6.74	11.43	79.9	4.14	25	2
			VVAGVANALAHK	9	15998.5	6.74	14.62	79.8	8.73	25	2
beta tubulin 1, class VI	13562114	TUBB1	LAVNMVPFPR	1	50327.2	5.06	15.88	89.9	9.75	25	2
beta-2-microglobulin precursor	4757826	B2M	LLYYTEFTPTEK	4	13714.6	6.06	11.75	74.3	4.53	25	2
			SNFLNCYVSGFHPSDIEVDLLK	4	13714.6	6.06	13.33	81.8	4.53	21	3
			VSGFHPSDIEVDLLK	4	13714.6	6.06	14.86	84	4.54	25	3
			YTEFTPTEKDEYACR	4	13714.6	6.06	13.59	86	4.41	25	3
brain abundant, membrane attached signal protein 1	30795231	BASP1	AAEAAAAPAESAAPAAGEEPSKEEGEPK	3	22693.5	4.64	16.14	93	4.12	24	3
			AEPPKAPEQEQAAPGPAAGGEAPK	3	22693.5	4.64	15.7	74	4.49	25	3
			AQGPAASAEEPKPVEAPAANSDQTVTVKE	3	22693.5	4.64	14.83	72.1	4.43	25	3
brain creatine kinase	21536286	CKB	LAVEALSSLDGDLAGR	1	42644.5	5.34	14.08	79.2	4.03	25	2
cadherin 2, type 1 preproprotein	14589889	CDH2	ESAEVEEIVFPR	3	99809.8	4.64	10.01	95.5	4.09	14	2
			LSLKPTLTEESVK	3	99809.8	4.64	12.18	75.7	6.14	25	3
			TGFPEDVYSAVLSK	3	99809.8	4.64	14.19	85.5	4.37	25	2
calcium channel, voltage-dependent, N type, alpha 1B subunit	4502523	CACNA1B	QQQIERELNGYLE	2	262497.7	8.78	12.46	75.7	4.52	25	3
			TLPSTCLQKVEEQPEDADNQRNVTRMG	2	262497.7	8.78	12.27	88.5	4.51	25	3
calnexin precursor	10716563	CANX	GTLSGWILSK	3	40050.8	4.47	10.19	86.9	8.75	15	2
			IPDPEAVKPDDWDEDAPAK	3	40050.8	4.47	18.44	84.8	3.85	25	3
			TPELNLDQFHDK	3	40050.8	4.47	12.71	70.3	4.53	25	3
calreticulin precursor	4757900	CALR	FYALSASFEPFSNK	3	48141.8	4.29	16.63	86.8	6	25	2
			IKDPDASKPEDWDER	3	48141.8	4.29	14.11	79.2	4.23	23	3
			KPEDWDEEMDGEWEPPVIQNPEYK	3	48141.8	4.29	15.48	80.5	3.84	25	3
carbohydrate (keratan sulfate Gal-6) sulfotransferase 1	4502841	CHST1	IAASEEELKNPSVSLVEER	2	46715	9.17	17.72	80.5	4.33	25	3
			TEEIYGFLGIPLDSHVAR	2	46715	9.17	14.16	73.8	4.65	25	3
carbonic anhydrase IX precursor	9955948	CA9	GGDPPWPRVSPACAGRFQSPVDI	2	49730.2	4.64	14.95	74.2	5.95	25	3
			TQPLNGRVIEASFPAGVDSSPRA	2	49730.2	4.64	12.55	74.8	5.73	25	3
carbonic anhydrase XI precursor	9951923	CA11	AHIGPAPDPEDWWSYK	3	36238.4	9.78	15.17	76.2	4.54	20	3
			LLSQNPPSQIFQSLSGNSR	3	36238.4	9.78	16.44	92	9.75	25	3
			VLYDPFLPPLR	3	36238.4	9.78	16.12	88.9	5.8	25	2
carboxypeptidase E precursor	4503009	CPE	AVIHWIMDIPFVLSANLHGGDLVANYPYDETR	13	53150.9	5.03	14.95	76.7	4.64	25	3
			AYSSFNPAMSDPNRPPCR	13	53150.9	5.03	15.58	87.7	8.26	25	3
			ELLIFLAQYLCNEYQK	13	53150.9	5.03	19.71	90.1	4.53	25	3
			IHIMPSLNPDGFEK	13	53150.9	5.03	19.46	82	5.32	25	3
			KLTASAPGYLAITK	13	53150.9	5.03	15.74	91.7	9.7	25	3
			LQQEDGISFEYHR	13	53150.9	5.03	13.05	76.4	4.65	25	2
			LTASAPGYLAITK	13	53150.9	5.03	15.19	86	8.59	25	2
			LTASAPGYLAITKK	13	53150.9	5.03	13	77.9	9.7	25	3
			NSLISYLEQIHR	13	53150.9	5.03	13.74	78	6.75	25	3
			SANLHGGDLVANYPYDETR	13	53150.9	5.03	13.42	78.2	4.53	25	3
			SGSAHEYSSSPDDAIFQSLAR	13	53150.9	5.03	18.33	88	4.53	25	3
			VAVPYSPAAGVDFELESFSER	13	53150.9	5.03	24.5	96.1	4	25	2
			VLSANLHGGDLVANYPYDETR	13	53150.9	5.03	17.94	82	4.54	25	3
cardiac calsequestrin 2	119395727	CASQ2	WIEDVLSGKINT	1	46436	4.22	13.89	78.7	4.37	25	2
CASK interacting protein 1	18079216	CASKIN1	DSEPERDE	1	46276.7	9.21	13.83	82.7	4.04	25	2
cathepsin D preproprotein	4503143	CTSD	AIGAVPLIQGEYMIPCEK	4	44552.5	6.1	22.05	88.7	4.53	25	2
			DPDAQPGGELMLGGTDSK	4	44552.5	6.1	20.78	91.8	3.84	25	2
			FDGILGMAYPR	4	44552.5	6.1	14.47	79.7	5.83	25	2
			LVDQNIFSFYLSR	4	44552.5	6.1	17.53	89.5	5.83	25	2
cathepsin Z preproprotein	22538442	CTSZ	NVDGVNYASITR	1	33868.1	6.7	16.64	86.5	5.83	25	2
CD14 antigen precursor	4557417	CD14	AFPALTSLDLSDNPGLGER	4	39420.2	5.84	20.84	82	4.03	25	3
			ITGTMPPLPLEATGLALSSLR	4	39420.2	5.84	17.43	87.5	6	25	2
			LTVGAAQVPAQLLVGALR	4	39420.2	5.84	14.25	86.6	9.75	25	2
			VLSIAQAHSPAFSCEQVR	4	39420.2	5.84	12.07	79.8	6.71	22	3
CD59 antigen p18-20	10835165	CD59	LQCYNCPNPTADCK	1	14177.4	6.03	22.38	94.1	5.81	25	2
cell death-regulatory protein GRIM19	156151369	NDUFA13	ENLEEEAIIMK	4	25821	9.82	11.95	86.6	4.09	25	2
			LQIEDFEAR	4	25821	9.82	10.17	79.1	4.14	23	2
			VKQDMPPPGGYGPIDYK	4	25821	9.82	12.51	73.5	5.93	21	3
			WVPPLIGELYGLR	4	25821	9.82	13.5	91.8	6	25	2
cell division cycle 42 isoform 1	4757952	CDC42	NVFDEAILAALEPPEPK	2	21258.7	6.16	20.5	88.3	4	25	3
			TCLLISYTTNK	2	21258.7	6.16	12.86	74.6	7.86	25	2
centromere protein F (350/400kD)	55770834	CENPF	TLQIGKRDANSSFFDNSS	2	357528.9	5.06	14.59	72.2	5.62	25	3
			VTPSRSTLQIGKRD	2	357528.9	5.06	12.47	70.4	10.83	25	3
chaperonin	31542947	HSPD1	IQEIIEQLDVTTSEYEK	6	61055	5.7	13.5	78.3	3.91	25	2
			ISSIQSIVPALEIANAHR	6	61055	5.7	15.3	78.6	6.75	25	3
			NAGVEGSLIVEK	6	61055	5.7	21.52	97.4	4.53	25	2
			TALLDAAGVASLLTTAEVVVTEIPKEEK	6	61055	5.7	23.53	95.4	4.25	25	3
			TLNDELEIIEGMK	6	61055	5.7	14.37	82.7	4	25	2
			TVIIEQSWGSPK	6	61055	5.7	16.59	90.9	5.66	25	2
chloride intracellular channel 1	14251209	CLIC1	LAALNPESNTAGLDIFAK	2	6457.6	5.09	13.82	77	4.37	25	2
			VLDNYLTSPLPEEVDETSAEDEGVSQR	2	6457.6	5.09	15.43	83.8	3.62	25	3
chromogranin A precursor	4502805	CHGA	CIVEVISDTLSK	17	50688.7	4.58	19.65	94.4	4.37	25	2
			CIVEVISDTLSKPSPMPVSQECFETLR	17	50688.7	4.58	16.35	85.4	4.41	25	3
			EDSLEAGLPLQVR	17	50688.7	4.58	12.52	73.1	4.14	25	2
			EEEEEMAVVPQGLFR	17	50688.7	4.58	12.76	81.9	3.98	25	2
			ELQDLALQGAK	17	50688.7	4.58	10.7	74.5	4.37	25	2
			GEQEHSQQKEEEEEMAVVPQGLFR	17	50688.7	4.58	12.88	87.9	4.35	17	3
			GLSAEPGWQAK	17	50688.7	4.58	17.82	88.9	6	25	2
			GPQAEGDSEGLSQGLVDR	17	50688.7	4.58	20.24	91.7	3.91	25	2
			KHSGFEDELSEVLENQSSQAELK	17	50688.7	4.58	18.69	89.7	4.41	25	3
			LEGQEEEEDNRDSSMK	17	50688.7	4.58	13.05	86.3	4	21	3
			LPVNSPMNK	17	50688.7	4.58	20.59	94.1	8.75	25	2
			PQALPEPMQESK	17	50688.7	4.58	13.39	90.8	4.53	25	2
			SEALAVDGAGKPGAEEAQD	17	50688.7	4.58	13.79	73.4	4.02	25	2
			SEALAVDGAGKPGAEEAQDPEGKGEQEHSQQK	17	50688.7	4.58	15.77	73.3	4.38	25	3
			SGEATDGARPQALPEPMQESK	17	50688.7	4.58	23.42	94.2	4.41	25	3
			VAHQLQALR	17	50688.7	4.58	14.05	80.1	9.73	25	2
			YPGPQAEGDSEGLSQGLVDR	17	50688.7	4.58	20.11	95.7	3.91	25	2
chromogranin B precursor	4502807	CHGB	ADEPQWSLYPSDSQVSEEVK	30	78246.6	5.02	17.65	92.9	3.83	25	2
			ADQTVLTEDEKKELENLAAMDLELQK	30	78246.6	5.02	13.27	77.7	4.12	25	3
			APRPQSEESWDEED	30	78246.6	5.02	18.84	89.7	3.94	25	2
			APRPQSEESWDEEDKR	30	78246.6	5.02	15.47	79.8	4.36	25	3
			ASEEEPEYGEEIK	30	78246.6	5.02	14.3	81.1	3.9	25	2
			AYFMSDTR	30	78246.6	5.02	13.04	88.2	5.88	25	2
			AYFMSDTREE	30	78246.6	5.02	18.8	94.1	4.43	25	2
			CIIEVLSNALSK	30	78246.6	5.02	26.46	98.7	5.99	25	2
			ELDRNYLNYGEEGAPGK	30	78246.6	5.02	22.55	94.1	4.41	25	3
			ELENLAAMDLELQK	30	78246.6	5.02	12.85	80.4	4	25	2
			ERADEPQWSLYPSDSQVSEEVK	30	78246.6	5.02	22.61	94.2	4.08	25	3
			FNPYYDPLQWK	30	78246.6	5.02	10.95	76.3	5.83	25	2
			GEAGAPGEEDIQGPTK	30	78246.6	5.02	19.49	93.8	4	25	2
			GEAGAPGEEDIQGPTKADTEK	30	78246.6	5.02	19.76	95.5	4.08	25	3
			GHPQEESEESNVSMASLGEK	30	78246.6	5.02	20.19	91.7	4.32	25	3
			GSEEYRAPRPQSEESWDEED	30	78246.6	5.02	15.73	85.1	4.06	25	3
			GYPGVQAPEDLEWER	30	78246.6	5.02	10.76	74	4	25	2
			HLEEPGETQNAFLNER	30	78246.6	5.02	20.2	92.5	4.48	25	3
			KPFSEDVNWGYE	30	78246.6	5.02	10.74	70.7	4.43	25	2
			KPFSEDVNWGYEK	30	78246.6	5.02	14.93	84.4	4.68	25	3
			LFNPYYDPLQWK	30	78246.6	5.02	16.23	85.6	5.83	25	2
			LGELFNPYYDPLQWK	30	78246.6	5.02	14.79	86.5	4.37	25	2
			NFFPEYNYDWWEK	30	78246.6	5.02	11.73	75.1	4.14	25	2
			RLGELFNPYYDPLQWK	30	78246.6	5.02	19.84	90.1	6.07	25	3
			SAEFPDFYDSEEPVSTHQEAENEK	30	78246.6	5.02	16.11	90.2	3.94	20	3
			SAEFPDFYDSEEPVSTHQEAENEKDR	30	78246.6	5.02	16.53	85.6	4.05	25	3
			SQEESEEGEEDATSEVDKR	30	78246.6	5.02	14.22	91.3	3.89	25	3

			SSAPPITPECR	30	78246.6	5.02	16.42	92.5	5.72	24	2
			VAQLDQLLH	30	78246.6	5.02	12.08	87	5.08	20	2
			WQQQGDLQDTK	30	78246.6	5.02	12.03	86.4	4.21	25	2
citrate synthase precursor, isoform a	38327625	CS	ASASSTNLKDILADLIPK	5	51712.7	8.45	17.02	86.1	6	25	3
			DILADLIPK	5	51712.7	8.45	13.26	96.9	4.21	19	2
			DYIWNTLNSGR	5	51712.7	8.45	17.52	90.5	5.83	25	2
			GLVYETSVLDPDEGIR	5	51712.7	8.45	17.11	89.3	3.91	25	2
			IVPNVLLEQGK	5	51712.7	8.45	17.68	89.1	6	25	2
clusterin isoform 1	42716297	CLU	DQTVSDNELQEMSNQGSK	7	57832.9	6.25	15.53	83	3.91	25	3
			EILSVDCSTNNPSQAK	7	57832.9	6.25	16.77	88	4.37	25	2
			ELDESLQVAER	7	57832.9	6.25	17.97	90.1	4	25	2
			KTLLSNLEEAKK	7	57832.9	6.25	12.1	70.1	8.5	25	3
			LFDSDPITVTVPVEVSR	7	57832.9	6.25	15.65	73.7	4.03	25	2
			TLLSNLEEAK	7	57832.9	6.25	12.76	81.2	4.53	25	2
			VTTVASHTSDSDVPSGVTEVVVK	7	57832.9	6.25	20.2	87.1	4.54	25	3
COBL-like 1	62821787	COBLL1	DKKKPTPIIPEKTV	2	54341.7	5.95	10.34	75.5	9.53	24	2
			LLSAEQNHIKFKPNTPIGMLEV	2	54341.7	5.95	12.17	77.9	6.76	25	3
cocaine- and amphetamine-regulated transcript	4757910	CARTPT	ALDIYSAVDDASHEK	3	48141.8	8.55	15.52	92.2	4.22	25	3
			ELIEALQEVLK	3	48141.8	8.55	20.67	96	4.25	25	2
			YGQVPMCDAGEQCAVR	3	48141.8	8.55	12.89	89.9	4.37	18	2
cofilin 1 (non-muscle)	5031635	CFL1	EILVGDVGQTVDDPYATFVK	5	18502.6	8.22	10.67	85.6	3.84	15	2
			HELQANCYEEVKDR	5	18502.6	8.22	16.23	79.9	4.83	25	3
			LGGSAVISLEGKPL	5	18502.6	8.22	16.21	83.1	6	25	2
			NIILEEGKEILVGDVGQTVDDPYATFVK	5	18502.6	8.22	17.58	86.7	4.02	25	3
			YALYDATYETK	5	18502.6	8.22	15.42	90.7	4.37	25	2
cofilin 2	14719392	CFL2	YALYDATYETK	1	18736.7	7.66	15.42	90.7	4.37	25	2
collagen, type III, alpha 1 preproprotein	4502951	COL3A1	AAGTPGLQGMPGERGGLGSPGPKGDKG	2	138556	6.18	12.32	70.1	8.63	25	3
			GPRGPVGPSGPPGKDGTSGHPGPIGPPGP	2	138556	6.18	12.9	74.8	8.76	25	3
complement component 7 precursor	45580688	C7	LIDQYGTHYLQSGSLGGEYR	1	93518.9	6.09	17.69	89.6	5.32	25	3
contactin 1 isoform 1 precursor	28373117	CNTN1	FIPLIPIPER	4	113321	5.61	13.47	80.9	6	25	2
			IKTDGAAPNVAPSDVGGGGGR	4	113321	5.61	18.58	82.1	5.96	25	3
			STEATLSFGYLDPFPPEERPEVR	4	113321	5.61	16.71	76.8	4.25	25	3
			VLEPMPSTAEISTSGAVLK	4	113321	5.61	16.25	83.1	4.53	23	2
creatine kinase, mitochondrial 1B precursor	10334859	CKMT1A CKMT1	GTGGVDTAATGGVFDISNLDR	4	47037	8.6	18.14	91.4	3.93	25	2
			GWEFMWNER	4	47037	8.6	11.01	90.8	4.53	13	2
			TTPTGWTLDQCIQTGVDNPGHPFIK	4	47037	8.6	15.51	77.2	5.18	25	3
			VVVDALSGLK	4	47037	8.6	10.97	97.9	5.81	14	2
cyclin M3 isoform 2	40068047	CNNM3	ERSNIVDMLYLKDLA	2	70632.5	6.67	12.27	78.2	4.56	25	3
			KRGDTVVKRKPA	2	70632.5	6.67	12.61	91.8	11.1	16	3
cystatin C precursor	4503107	CST3	AFCSFQIYAVPWQGTMTLSK	7	15799.3	9	13.35	87.1	8.24	25	2
			ALDFAVGEYNK	7	15799.3	9	13.85	85.6	4.37	25	2
			AVPWQGTMTLSK	7	15799.3	9	17.52	92.1	8.8	25	2
			LVGGPMDASVEEEGVR	7	15799.3	9	14.15	87.2	4	25	2
			LVGGPMDASVEEEGVRR	7	15799.3	9	16.02	95.3	4.41	25	3
			QIYAVPWQGTMTLSK	7	15799.3	9	15.17	87.4	8.59	25	2
			RLVGGPMDASVEEEGVR	7	15799.3	9	13.98	81.3	4.41	25	3
cysteine-rich protein 2	4503049	CRIP2	ASSVTTFTGEPNTCPR	3	22492.9	9.01	14.6	84.9	6.04	25	2
			GVNIGGAGSYIYEKPLAEGPQVTGPIEVPAAR	3	22492.9	9.01	21.96	86	4.79	25	3
			GVNTGAVGSYIYDRDPEGK	3	22492.9	9.01	13.06	74.8	4.56	25	3
cytochrome b-561	63054828	CYB561	EALLFNLGGK	2	41053.2	8.73	14.18	84.2	6.1	25	2
			RPSQAEEQALSMDFK	2	41053.2	8.73	21.5	95.3	4.68	25	3
cytochrome b5 reductase b5R.2	47778923	CYB5R2	LGMIAGGTGITPMLQLIR	2	32990	8.49	13.46	85.4	9.75	25	2
			LWYTLDR	2	32990	8.49	10.15	93.1	5.83	16	2
cytochrome b5 reductase isoform 1	4503327	CYB5R3	APEAWDYGQGFVNEEMIR	6	34235.1	7.18	16.88	86.1	4	25	2
			GPSGLLVYQGK	6	34235.1	7.18	16.99	89.3	8.59	25	2
			LWYTLDR	6	34235.1	7.18	10.15	93.1	5.83	16	2
			MSQYLESMQIGDTIEFR	6	34235.1	7.18	12.47	81.6	4.14	24	2
			STPAITLESPDIK	6	34235.1	7.18	12.58	77.3	4.37	25	2
			STPAITLESPDIKYPLR	6	34235.1	7.18	17.4	87.6	5.79	25	3
cytochrome c oxidase subunit II	17981856	MT-CO2	ILYMTDEVNDPSLTIK	2	25565.2	4.67	23.85	97.3	4.03	25	2
			MLPPLFLEPGDLR	2	25565.2	4.67	13.43	71.4	4.37	24	2
cytochrome c oxidase subunit IV isoform 1 precursor	4502981	COX4I1	ASWSSLSMDEK	5	19576.8	9.52	14.62	80.4	4.37	25	2
			DHPLPEVAHVK	5	19576.8	9.52	17.08	84	5.99	25	3
			HYVYGPLPQSFDK	5	19576.8	9.52	16.52	88.6	6.74	25	3
			SEDFSLPAYMDR	5	19576.8	9.52	14.3	75.3	4.03	25	2
			VNPIQGLASK	5	19576.8	9.52	12.64	94.3	8.72	16	2
cytochrome c oxidase subunit Va precursor	4758038	COX5A	GINTLVTYDMVPEPK	2	21258.7	6.3	21.35	94.7	4.37	25	2
			VIQELRPTLNELGISTPEELGLDKV	2	21258.7	6.3	19.18	89.4	4.25	25	3
cytochrome c oxidase subunit Vb precursor	17017988	COX5B	ASGGGVPTDEEQATGLER	5	13695.8	9.07	12.43	81.6	4	25	2
			EDPNLVPSISNK	5	13695.8	9.07	14.65	87.7	4.37	25	2
			GLDPYNVLAPK	5	13695.8	9.07	16.63	86.5	5.83	25	2
			KGLDPYNVLAPK	5	13695.8	9.07	12.78	80.4	8.5	25	3
			SGGGVPTDEEQATGLER	5	13695.8	9.07	15.28	80	4	21	2
cytochrome c oxidase subunit VIIa polypeptide 1 (muscle) precursor	4502987	COX7A1	GGIVDNILYR	2	9117.6	10.12	10.3	74.9	5.83	25	2
			LFQEDNDIPLYLK	2	9117.6	10.12	18.18	89.9	4.03	25	2
cytochrome c oxidase subunit VIIa polypeptide 2 (liver) precursor	4502989	COX7A2	GGVADALLYR	2	9117.6	9.75	14.98	76.6	5.83	25	2
			LFQEDDEIPLYLK	2	9117.6	9.75	17.09	88	3.91	25	2
cytochrome c oxidase subunit VIIa polypeptide 2 like	18105037	COX7A2L	GLPDQMLYR	2	12614.7	9.43	14.93	84.5	5.83	25	2
			LAGAWASEAYSPQGLKPVVSTEAPPIIFATPTK	2	12614.7	9.43	12.1	77	6.14	20	3
cytochrome c oxidase subunit VIIc precursor	4502993	COX7C	NLPFSVENK	1	7245.5	10.29	12.46	83.6	6	25	2
cytochrome c	11128019	CYCS	TGPNLHGLFGR	2	124108.2	9.59	12.22	71.6	9.44	25	2
			TGQAPGYSYTAANK	2	124108.2	9.59	13.38	96.4	8.16	15	2
cytochrome c-1	21359867	CYC1	AANNGALPPDLSYIVR	8	35390.1	9.15	12.39	71.3	5.88	25	3
			ALLVPLVYTIK	8	35390.1	9.15	13.56	80.7	8.63	24	2
			ELAAEVEVQDGPNEDGEMFMRPGK	8	35390.1	9.15	18.35	87.3	4	25	3
			GLLSSLDHTSIR	8	35390.1	9.15	14.44	89.1	6.74	25	3
			HGGEDYVFSLLTGYCEPPTGVSLR	8	35390.1	9.15	21.57	90.8	4.65	25	3
			HLVGVCYTEDEAK	8	35390.1	9.15	12.55	72.7	4.65	25	3
			LFDYFPKPYPNSEAAR	8	35390.1	9.15	18.56	95.1	6.07	25	3
			SDLELHPPSYPWSHR	8	35390.1	9.15	12.83	84.2	5.94	19	3
cytosolic sialic acid 9-O-acetylesterase homolog	24850115	SIAE	FFPFGLVQLSSDLSK	1	58315.2	6.93	17.71	84.2	5.84	25	2
defensin, alpha 1 preproprotein	4758146	DEFA1	IPACIAGER	2	10201	6.54	19.48	90.4	6	25	2
			YGTCIYQGR	2	10201	6.54	12.83	80.5	8.2	25	2
desmin	55749932	DES	EYQDLLNVK	3	53536	5.21	12.55	93.2	4.37	25	2
			TNEKVELQELNDR	3	53536	5.21	10.88	93.2	4.41	16	2
			VELQELNDR	3	53536	5.21	19.33	95.7	4.14	25	2
dicarbonyl/L-xylulose reductase	7705925	DCXR	ALGSVGPVDLLVNNAAVALLQPFLEVTK	1	25913.2	8.33	20.27	95.2	4.37	25	3
dickkopf homolog 3 precursor	40548389	DKK3	DQDGEILLPR	5	38390.5	4.59	19.13	85.9	4.03	25	2
			EVPDEYEVGSFMEEVR	5	38390.5	4.59	12.42	82.4	3.83	25	2
			EVPDEYEVGSFMEEVRQELEDLER	5	38390.5	4.59	16.51	80.4	3.84	25	3
			LLDLITWELEPDGALDR	5	38390.5	4.59	12.06	86.8	3.77	25	2
			SAVEEMEAEEAAAK	5	38390.5	4.59	21.51	94.6	3.98	25	2
dipeptidyl peptidase 7 preproprotein	62420888	DPP7	LYHSCADPTGCGTGPDAR	1	54341.7	5.91	17.45	82.9	5.21	25	3
DJ-1 protein	31543380	PARK7	EGPYDVVVLPGGNLGAQNLSESAAVK	2	19891.2	6.33	19.65	94	4.14	25	3
			VTVAGLAGKDPVQCSR	2	19891.2	6.33	13.17	79.3	8.19	25	3
dodecenoyl-Coenzyme A delta isomerase precursor	62530384	DCI	DADVQNFVSFISK	2	54341.7	8.8	19.18	84.8	4.21	25	2
			GVILTSDRPGVFSAGLDLTEMCGR	2	54341.7	8.8	15.19	87.2	4.56	25	3
dopamine beta-hydroxylase precursor	116534900	DBH	AGVLFGMSDR	22	69065.4	5.97	11.98	85.3	5.88	25	2
			ALYSFAPISMH	22	69065.4	5.97	15.04	90.2	6.78	25	2
			ASVSQQFTSVPWNSFNR	22	69065.4	5.97	12.5	78.7	9.79	24	2
			DYLIEDGTVHLVYGILEEPFR	22	69065.4	5.97	24.74	100	4.17	25	3
			ESPLPYHIPLD	22	69065.4	5.97	13.56	72.9	4.64	25	2
			EWEIVNQDNHYSPHFQEIR	22	69065.4	5.97	17.9	84.2	4.8	25	3
			FNAGIMELGLVYTPVM	22	69065.4	5.97	16.52	93.9	4	25	2
			FNNEDVCTCPQASVSQQFTSVPWNSFNR	22	69065.4	5.97	14.82	82	4.37	25	3
			GELENADLVVLWTDGDTAYFADAWSDQK	22	69065.4	5.97	15.21	88.7	3.57	25	3
			GILEEPFR	22	69065.4	5.97	14.07	81.2	4.53	25	2
			HVLAAWALGAK	22	69065.4	5.97	13.04	84.4	8.76	25	2
			IQIPSQETTYWCYIK	22	69065.4	5.97	21.94	95.1	5.99	25	2
			LEVHYHNPLVIEGR	22	69065.4	5.97	17.14	84.7	6	25	3
			RFNAGIMELGLVYTPVMAIPPR	22	69065.4	5.97	13.89	81.4	8.75	25	3
			SAVDAGFLQK	22	69065.4	5.97	16.48	95.6	5.55	19	2
			SPAGPTVVSIGGGK	22	69065.4	5.97	17.3	95.6	8.47	20	2
			VISTLEEPTPQCPTSQGR	22	69065.4	5.97	18.4	89.8	4.53	25	2
			VQLLKPNIPEPELPSDA	22	69065.4	5.97	14.46	76.6	4.14	25	2
			VQLLKPNIPEPELPSDACTMEVQAPN	22	69065.4	5.97	13.56	75	4	25	3
			VVSVHPGDVLITSCTY	22	69065.4	5.97	10.39	76.3	5.08	23	2
			VVSVHPGDVLITSCTYNTEDR	22	69065.4	5.97	14.34	79	4.54	25	3
			YFHLINR	22	69065.4	5.97	13.4	85.1	8.75	25	2
dUTP pyrophosphatase isoform 1 precursor	70906441	DUT	AGGSPAPGPETPAISPSK	6	26563.4	9.46	15.28	74.9	6.05	25	2
			AGGSPAPGPETPAISPSKR	6	26563.4	9.46	21.7	96	8.79	25	3
			ARPAEVGGMQLR	6	26563.4	9.46	17.91	90.4	9.64	23	3
			GNVGVVLFNFGK	6	26563.4	9.46	15.07	85.6	8.75	25	2
			IFYPEIEEVQALDDTER	6	26563.4	9.46	10.83	75.9	3.77	25	2
			TDIQIALPSGCYGR	6	26563.4	9.46	11.67	89	5.5	17	2
dynactin 2	5453629	DCTN2	LLGPDAAINLTDPDGALAK	1	44819.9	5.06	12.11	70.8	3.93	25	2
dystroglycan 1 preproprotein	148539835	DAG1	EGAMSAQLGYPVVGWHIANK	2	83654.4	8.71	12.9	80.3	6.85	25	3
			LFDMSAFMAGPGNAK	2	83654.4	8.71	12.89	83.8	5.84	18	2
elastin isoform a	126352440	ELN	FPGVGVLPGVPTGAGVKPK	2	66039	10.45	15.4	80.7	10	25	3
			VPGVGLPGVYPGGVLPGAR	2	66039	10.45	13.02	75.3	8.72	25	2
electron transfer flavoprotein, alpha polypeptide	4503607	ETFA	AAVDAGFVPNDMQVGQTGK	5	35079.8	8.62	12.06	70.1	4.21	25	3
			ASSTSPVEISEWLDQK	5	35079.8	8.62	12.57	70.5	4.14	25	2
			GLLPEELTPLILATQK	5	35079.8	8.62	20.37	91	4.53	25	2
			LEVAPISDIIAIK	5	35079.8	8.62	13.22	88.3	4.37	25	2
			LLYDLADQLHAAVGASR	5	35079.8	8.62	20.04	92.6	5.21	25	3
electron-transfer-flavoprotein, beta polypeptide isoform 2	62420877	ETFB	GIHVEVPPAEAER	2	37434.7	6.78	13.21	79.5	4.75	18	3
			LSVISVEDPPQR	2	37434.7	6.78	13.19	79	4.37	25	2
enolase 1	4503571	ENO1	AAVPSGASTGIYEALELR	2	61963.6	7.01	12.95	71	4.53	25	2
			YISPDQLADLYK	2	61963.6	7.01	16.4	85.7	4.21	25	2
enolase 3	153267427	ENO3	AAVPSGASTGIYEALELR	1	46932	7.58	12.95	71	4.53	25	2
epididymal secretory protein E1 precursor	5453678	NPC2	AVVHGILMGVPVPFPIPEPDGCK	2	16570.3	7.57	17.74	87.1	5.32	25	3
			EVNVSPCPTQPCQLSK	2	16570.3	7.57	10.09	80.7	6.08	16	2
ETHE1 protein	41327741	ETHE1	EAVLIDPVLETAPR	2	27873.1	6.35	15.16	80.3	4.14	25	2
			LSGAQADLHIEDGDSIR	2	27873.1	6.35	14.55	78.2	4.22	25	3
exophilin 5	21359818	EXPH5	ASRRSVFALSNEDPLPFCSDLSGKERG	2	222548.3	7.87	12.65	74	6.25	25	3
			AVSLPEEESKAREIFSDNLAKTPLGDS	2	222548.3	7.87	13.75	72.8	4.36	25	3
Fc fragment of IgE, high affinity I, receptor for, gamma polypeptide precursor	4758344	FCER1G	SDGVYTGLSTR	1	41487.7	6.55	12.21	73.3	5.55	25	2
fibrinogen, beta chain preproprotein	70906435	FGB	DNDGWLTSDPR	6	55928.5	8.54	17.03	84.9	3.93	25	2
			DNENVVNEYSSELEK	6	55928.5	8.54	13.57	94.4	3.91	16	2
			GGETSEMYLIQPDSSVKPYR	6	55928.5	8.54	19.41	94.1	4.68	25	3
			MGPTELLIEMEDWKGDK	6	55928.5	8.54	15.57	79.9	4.18	25	3
			NYCGLPGEYWLGNDK	6	55928.5	8.54	12.04	82	4.37	25	2
			TPCTVSCNIPVVSGK	6	55928.5	8.54	14.57	80.3	7.74	25	2
fibrinogen, gamma chain isoform gamma-B precursor	70906439	FGG	AIQLTYNPDESSKPNMIDAATLK	3	55928.5	5.37	12.61	77	4.56	25	3
			IHLISTQSAIPYALR	3	55928.5	5.37	18.24	86	8.75	25	3
			YLQEIYNSNNQK	3	55928.5	5.37	12.41	78.7	6	25	2
FK506-binding protein 2 precursor	17149842	FKBP2	GWDQGLLGMCEGEK	5	15649.4	9.24	20.11	94.6	4.14	25	2
			IPGGATLVFEVELLK	5	15649.4	9.24	18.74	90.7	4.53	25	2
			LEDGTEFDSSLPQNQPFVF	5	15649.4	9.24	15.45	86.5	3.43	25	2

			LEDGTEFDSSLPQNQPFVFSLGTGQVIK	5	15649.4	9.24	17.72	83.8	3.91	25	3
			LVIPSELGYGER	5	15649.4	9.24	18.05	90.9	4.53	24	2
FK506-binding protein 3	4503727	FKBP3	GWDEALLTMSK	1	25176.9	9.29	13.37	84.1	4.37	21	2
fumarate hydratase precursor	19743875	FH	IYELAAGGTAVGTGLNTR	3	53005.6	8.85	14.54	81.8	6	25	2
			SGLGELILPENEPGSSIMPGK	3	53005.6	8.85	15.84	77.2	4.25	25	3
			THTQDAVPLTLGQEFSGYVQQVK	3	53005.6	8.85	15	84.3	5.29	25	3
G protein-coupled receptor 98 precursor	113722120	GPR98	TVPEKEESF	2	693072.7	4.53	10.47	85.1	4.25	17	2
			WKAYDDVFRGRT	2	693072.7	4.53	14.16	82.3	8.59	25	3
G-protein gamma-12 subunit	51036603	GNG12	ASADLMSYCEEHAR	3	8006.3	9.14	14	81.5	4.65	25	3
			SDPLLIGIPTSENPFK	3	8006.3	9.14	19.69	94.1	4.37	25	2
			SDPLLIGIPTSENPFKDK	3	8006.3	9.14	18.03	95.2	4.56	25	3
gamma filamin	116805322	FLNC	RCSYVPTKPIK	2	291023.7	5.65	12.87	82.1	9.79	25	3
			TVGPLGEGGAHKVRAGGTGL	2	291023.7	5.65	12.68	70.6	8.44	25	3
glucose phosphate isomerase	18201905	GPI	ILLANFLAQTEALMR	1	63147.5	8.42	13.96	80.4	6	25	2
glutaminyl-peptide cyclotransferase precursor	6912618	QPCT	LQAIEHELHELGLLK	5	40876.8	6.12	18.55	91.8	5.32	25	3
			QIAEGTSISEMWQNDLQPLLIER	5	40876.8	6.12	17.69	85.7	4	25	3
			SFSNIISTLNPTAK	5	40876.8	6.12	13.36	87.1	8.47	25	2
			VSPSASAWPEEK	5	40876.8	6.12	16.68	92.6	4.53	25	2
			YFQNYSYGGVIQDDHIPFLR	5	40876.8	6.12	15.52	77.2	5.21	25	3
glutathione peroxidase 1 isoform 1	41406084	GPX1	DYTQMNELQR	2	21938.3	6.15	16.85	90.5	4.37	25	2
			EALPAPSDDATALMTDPK	2	21938.3	6.15	15.66	75.2	3.84	25	2
glutathione peroxidase 1 isoform 2	41406082	GPX1	DYTQMNELQR	1	229000.3	10.26	16.85	90.5	4.37	25	2
glutathione transferase kappa 1	7705704	GSTK1	AGMSAEQAQGLLEK	3	25497	8.51	18.31	94	4.53	23	2
			DFLSVMLEK	3	25497	8.51	14.18	90.8	4.37	21	2
			NEDITEPQSILAAAEK	3	25497	8.51	13.26	78.2	4	25	2
glyceraldehyde-3-phosphate dehydrogenase	7669492	GAPDH	GALQNIIPASTGAAK	6	36053.4	8.57	10.01	77	8.75	25	2
			IISNASCTTNCLAPLAK	6	36053.4	8.57	10.76	83.5	8.06	17	2
			LVINGNPITIFQER	6	36053.4	8.57	13.73	71.6	6	25	2
			VIISAPSADAPMFVMGVNHEK	6	36053.4	8.57	15.09	89.6	5.32	25	3
			VPTANVSVVDLTCR	6	36053.4	8.57	14.67	79.8	5.8	25	2
			VVDLMAHMASKE	6	36053.4	8.57	13.88	84.7	5.51	25	3
GNAS complex locus isoform c	117938759	GNAS	LLLLGAGESGK	3	111025.1	4.91	14.13	87.4	6	25	2
			QADYVPSDQDLLR	3	111025.1	4.91	12.43	77	3.93	25	2
			SKIEDYFPEFAR	3	111025.1	4.91	12.7	77.2	4.68	19	3
growth and transformation-dependent protein	49355721	FAM162A	ILIWSGR	2	17342.5	9.81	10.47	97.1	9.75	13	2
			KEDEIPETVSLEMLDAAK	2	17342.5	9.81	12.83	77.5	4.08	25	3
growth differentiation factor 10 precursor	4826740	GDF10	ATGPLQDNELPGLDERPPR	9	53122.3	9.58	17.35	86	4.32	25	3
			AVGIIPGIPEPCCVPDK	9	53122.3	9.58	19.04	90.3	4.37	25	2
			DGELLLSAQLDSEERDPGVPRPSPYAPY	9	53122.3	9.58	13.44	71.2	4.02	25	3
			GQEVFMAASQVLDFDEK	9	53122.3	9.58	19.18	92.6	3.91	25	2
			ILVYANDLAISEPNSVAVTLQR	9	53122.3	9.58	15.32	82.1	4.37	25	3
			IVRPSNHATIQSIVR	9	53122.3	9.58	12.68	77.4	12	25	3
			SFDAYYCAGACEFPMPK	9	53122.3	9.58	12.22	79.8	4.37	25	2
			VDFADIGWNEWIISPK	9	53122.3	9.58	13.89	71.4	4.03	25	2
			VYPNMSVDTCACR	9	53122.3	9.58	14.87	90	5.79	25	2
growth differentiation factor 15	153792495	GDF15	AALPEGLPEASR	2	34140.4	9.79	11.11	71.8	4.53	25	2
			TDTGVSLQTYDDLLAK	2	34140.4	9.79	11.7	74.1	3.93	25	2
GrpE-like 1, mitochondrial	24308295	GRPEL1	DLLEVADVLEK	1	24279.2	8.24	17.55	88.9	3.91	25	2
GSK-3 binding protein FRAT1	31317236	FRAT1	SGNLIKEAVRR	2	29093.1	7.65	10.44	70.6	10.83	25	2
			SPRSPRAACSDPGASGRAQLRTG	2	29093.1	7.65	12.14	70.7	11.52	25	3
GTPase Rab14	19923483	RAB14	GAAGALMVYDITR	6	23897.1	5.85	11.82	71.6	5.83	25	2
			LQIWDTAGQER	6	23897.1	5.85	13.81	89.3	4.37	25	2
			NLTNPNTVIILIGNK	6	23897.1	5.85	16.05	85	8.75	25	2
			TGENVEDAFLEAAK	6	23897.1	5.85	13.16	82.6	4	25	2
			TGENVEDAFLEAAKK	6	23897.1	5.85	13.42	91.3	4.41	23	3
			YIIIGDMGVGK	6	23897.1	5.85	12.34	78.6	5.83	25	2
guanine nucleotide binding protein (G protein) alpha 12	42476111	GNA12	ILLLGAGESGK	1	44279.5	9.84	14.13	87.4	6	25	2
guanine nucleotide binding protein (G protein), alpha 14	4758444	GNA14	VPTTGIIEYPFDLENIIFR	2	64733.1	5.81	14.67	80	4.14	25	3
			VSMLSREQVE	2	64733.1	5.81	11.59	70.4	4.85	25	2
guanine nucleotide binding protein (G protein), alpha activating activity polypeptide, olfactory type isoform 1	33695153	GNAL	LLLLGAGESGK	1	52455	6.69	14.13	87.4	6	25	2
guanine nucleotide binding protein (G protein), alpha inhibiting activity polypeptide 2	4504041	GNAI2	IAQSDYIPTQQDVLR	2	40451.1	5.34	12.36	80.7	4.21	25	2
			LLLLGAGESGK	2	40451.1	5.34	14.13	87.4	6	25	2
guanine nucleotide binding protein (G protein), alpha inhibiting activity polypeptide 3	5729850	GNAI3	LLLLGAGESGK	1	40532.4	5.51	14.13	87.4	6	25	2
guanine nucleotide binding protein (G protein), gamma 3	6912394	GNG3	GETPVNSTMSIGQAR	2	8304.8	7.65	17.91	83.7	6	25	2
			MKGETPVNSTMSIGQAR	2	8304.8	7.65	13.09	76.8	8.5	25	3
guanine nucleotide binding protein (G protein), gamma 4	4758450	GNG4	EDPLIIPVPASENPFR	5	8388.8	6.55	16.98	96.6	4.14	20	2
			IPVPASENPFR	5	8388.8	6.55	13.27	73.2	6	25	2
			MKEGMSNNSTTSISQAR	5	8388.8	6.55	12.28	73.4	8.5	25	3
			PLIIPVPASENPFR	5	8388.8	6.55	11.13	82	6.43	23	2
			VSQAAADLLAYCEAHVR	5	8388.8	6.55	19.52	82.7	5.32	25	3
guanine nucleotide binding protein (G protein), gamma 7	32698769	GNG7	AASDLMSYCEQHAR	3	7521.8	8.71	12.8	76.2	5.32	25	3
			NDPLLVGVPASENPFK	3	7521.8	8.71	20.59	95.6	4.37	25	2
			NDPLLVGVPASENPFKDK	3	7521.8	8.71	15.08	84.5	4.56	25	3
guanine nucleotide binding protein (G protein), gamma 8	15147246	GNG8	DDPLVTPVPAAENPFR	1	7841.2	6.55	15.95	90.2	4.03	25	2
guanine nucleotide binding protein (G protein), q polypeptide	40254462	GNAQ	VADPAYLPTQQDVLR	2	42142.3	5.48	14.23	77.3	4.21	25	2
			VPTTGIIEYPFDLQSVIFR	2	42142.3	5.48	11.82	78	4.37	25	2
guanine nucleotide binding protein, alpha activating polypeptide O	10567816	GNAO1	AMDTLGIEYGDKER	6	40050.8	5.34	13.44	92.6	4.32	25	3
			IGAADYQPTEQDILR	6	40050.8	5.34	11.68	80.1	4.03	25	2
			LLLLGAGESGK	6	40050.8	5.34	14.13	87.4	6	25	2
			LWGDSGIQECFNR	6	40050.8	5.34	12.55	79.2	4.37	25	2
			MEDTEPFSAELLSAMMR	6	40050.8	5.34	11.26	71.9	4	25	2
			YYLDSLDR	6	40050.8	5.34	10.25	91.2	4.21	18	2
guanine nucleotide binding protein, alpha transducing 3	156139155	GNAT3	LLLLGAGESGK	1	40357.2	5.69	14.13	87.4	6	25	2
guanine nucleotide binding protein, alpha transducing activity polypeptide 1	22027520	GNAT1	LLLLGAGESGK	1	40041	5.4	14.13	87.4	6	25	2
guanine nucleotide binding protein, alpha transducing activity polypeptide 2	20330805	GNAT2	LLLLGAGESGK	1	40176.1	5.11	14.13	87.4	6	25	2
H(+)-transporting two-sector ATPase	7706757	ATP6V1D	AVELLVELASLQTSFVTLDEAIK	4	28263	9.36	11.64	79.1	4	20	2
			EAAFSLAEAK	4	28263	9.36	13.3	85.4	4.53	25	2
			TLAYIITELDER	4	28263	9.36	18.08	82.4	4.14	25	2
			VNAIEHVIIPR	4	28263	9.36	12.02	76.5	6.72	25	3
heat shock 10kDa protein 1 (chaperonin 10)	4504523	HSPE1	GGEIQPVSVK	3	10931.7	8.89	12.09	71.4	6	25	2
			GGIMLPEK	3	10931.7	8.89	11.01	79.2	6	25	2
			VLQATVVAVGSGSK	3	10931.7	8.89	23.34	95.6	8.72	25	2
heat shock 27kDa protein 1	4504517	HSPB1	KYTLPPGVDPTQVSSSLSPEGTLTVEAPMPK	4	22782.6	5.98	15.88	77.5	4.68	25	3
			LATQSNEITIPVTFESR	4	22782.6	5.98	17.2	87	4.53	25	3
			LFDQAFGLPR	4	22782.6	5.98	15.74	84	5.84	25	2
			VSLDVNHFAPDELTVK	4	22782.6	5.98	14.05	73.6	4.54	25	3
heat shock 70kDa protein 5	16507237	HSPA5	AKFEELNMDLFR	10	72333.3	5.07	20.25	95.3	4.68	25	3
			DAGTIAGLNVMR	10	72333.3	5.07	18.06	91.3	5.84	25	2
			DNHLLGTFDLTGIPPAPR	10	72333.3	5.07	15.46	85.7	5.21	25	3
			ELEEIVQPIISK	10	72333.3	5.07	12.81	84.5	4.25	21	2
			IEIESFYEGEDFSETLTR	10	72333.3	5.07	17.38	87.8	3.83	25	2
			ITPSYVAFTPEGER	10	72333.3	5.07	13.69	83.4	4.53	25	2
			NELESYAYSLK	10	72333.3	5.07	15.45	87.8	4.53	25	2
			TFAPEEISAMVLTK	10	72333.3	5.07	16.19	84.1	4.53	25	2
			TKPYIQVDIGGGQTK	10	72333.3	5.07	15.97	77.1	8.17	25	3
			VTHAVVTVPAYFNDAQR	10	72333.3	5.07	14.96	81	6.71	25	3
heat shock 70kDa protein 8 isoform 1	5729877	HSPA8	IAGLNVLRIINEPTAAAIAYGLDK	2	21671.3	5.38	15.58	81.8	6.07	25	3
			SINPDEAVAYGAAVQAAILSGDK	2	21671.3	5.38	14.41	73.3	4.03	25	3
heat shock 70kDa protein 9 precursor	24234688	HSPA9	DAGQISGLNVLR	8	73680.9	5.87	12.79	79.6	5.84	25	2
			EQQIVIQSSGGLSKDDIENMVK	8	73680.9	5.87	12.51	82.5	4.32	25	3
			ISGLNVLRVINEPTAAALAYGLDK	8	73680.9	5.87	13.64	75.3	6.07	25	3
			LLGQFTLIGIPPAPR	8	73680.9	5.87	10.41	75.7	9.75	25	2
			QAVTNPNNTFYATK	8	73680.9	5.87	13.82	80.6	8.59	25	2
			SDIGEVILVGGMTR	8	73680.9	5.87	13.73	76.4	4.37	25	2
			TTPSVVAFTADGER	8	73680.9	5.87	14.53	80.1	4.37	25	2
			VINEPTAAALAYGLDK	8	73680.9	5.87	15.99	85.8	4.37	25	2
hemicentin 1	118572606	HMCN1	GRELTGREPGI	2	613393.1	6.07	10.07	73.6	6.14	25	2
			LTIYIPPSIKGGNVTTDISVLINSLI	2	613393.1	6.07	12.89	70.6	5.83	25	3
hexosaminidase B preproprotein	4504373	HEXB	EISEVFPDQFIHLGGDEVEFK	2	63111.7	6.29	12.73	84.7	4.07	25	3
			LAPGTIVEVWK	2	63111.7	6.29	10.65	80.7	6	25	2
hippocalcin-like 1	19913441	HPCAL1	IYANFFPYGDASK	3	22313.3	5.21	12.6	88	5.83	25	2
			LSLEEFIR	3	22313.3	5.21	18.96	94	4.53	25	2
			SEMLEIVQAIYK	3	22313.3	5.21	17.01	75.1	4.53	25	2
hormone-regulated proliferation-associated 20 kDa protein	7661786	C6orf66	IMQEYQLEQK	2	20266.6	8.85	14.65	86.1	4.53	25	2
			ISIVEALTLLNNHK	2	20266.6	8.85	15.57	91.1	6.75	25	3
HtrA serine peptidase 1	4506141	HTRA1	IAPAVVHIELFR	3	51287.3	8.09	16.46	96.5	6.75	17	3
			LPVLLLGR	3	51287.3	8.09	14.72	91.7	9.75	21	2
			RGNEDIMITVIPEEIDP	3	51287.3	8.09	13.57	85	3.83	25	2
huntingtin	90903231	HTT	LVVVSKLPSHLHLPPEKEKDI	2	3840.3	5.81	14.59	79.5	6.93	25	3
			VLTLYVDRL	2	3840.3	5.81	11.38	90.1	5.8	19	2
hyaluronan and proteoglycan link protein 4	30794472	HAPLN4	DGLDWCNAGWLR	5	42801.6	9.12	10.38	74.9	4.21	25	2
			DPAAWTPLHV	5	42801.6	9.12	13.92	84.8	5.08	25	2
			LDLEGVVFPYHPR	5	42801.6	9.12	15.95	87	5.32	24	3
			YDAFCFTSNLPGR	5	42801.6	9.12	15.58	84.2	5.83	25	2
			YECEVTNELEDDAGMVK	5	42801.6	9.12	16.66	84.9	3.77	25	2
hydroxyacyl glutathione hydrolase isoform 1	94538322	HAGH	TVQQHAGETDPVTTMR	1	11731.3	8.35	15.69	79.3	5.29	25	3
hydroxysteroid (17-beta) dehydrogenase 10 isoform 1	4758504	HSD17B10	DVQTALALAK	7	26923.2	7.65	11.51	77.7	5.84	25	2
			GGIVGMTLPIAR	7	26923.2	7.65	19	95.2	9.75	25	2
			GLVAVITGGASGLGLATAER	7	26923.2	7.65	23.09	97.1	6	25	2
			LGDPAEYAHLVQAIIENPFLNGEVIR	7	26923.2	7.65	12.19	71.3	4.4	24	3
			LGNNCVFAPADVTSEKDVQTALALAK	7	26923.2	7.65	14.96	79.9	4.56	25	3
			VDVAVNCAGIAVASK	7	26923.2	7.65	15.04	77.6	5.8	25	2
			VLDVNLMGTFNVIR	7	26923.2	7.65	19.49	96.1	5.81	25	2
hydroxysteroid (17-beta) dehydrogenase 12	7705855	HSD17B12	GAILNISSGSGMLPVPLLTIYSATK	2	34324.4	9.34	19.38	79.4	8.59	25	3
			VWGVGNEAGVGPGLGEWAVVTGSTDGIGK	2	34324.4	9.34	14.59	82.4	4.14	25	3
hypothetical protein LOC158763	40255080		AAVLAQSKPSDEGSSEEPAVPSGTAR	12	61664.5	9.48	17.31	84.7	4.41	25	3
			AMIDNWDVLFQVPPHIQR	12	61664.5	9.48	12.96	82.5	5.21	25	3
			EEFDQGLDVVLDDNQNVHDVAALLK	12	61664.5	9.48	13.57	81.3	3.85	25	3
			GAVSVDSLAELEDGALLLQTLQLSK	12	61664.5	9.48	18.45	93.6	3.91	25	2
			HFLSEFKPDR	12	61664.5	9.48	14.08	72.8	6.75	25	3
			HLELTATMQVEEATGQAAGR	12	61664.5	9.48	14.37	80.9	4.75	25	3
			ITENCEDSIGIDGQLVPGNR	12	61664.5	9.48	13.6	80.2	3.91	25	3
			LGIDHYVASVNVVR	12	61664.5	9.48	16.09	75.8	6.74	25	3
			MTSTNLALVFGSALLK	12	61664.5	9.48	16.79	83.5	8.5	25	2
			MVSIHSLSELER	12	61664.5	9.48	13.39	89.1	5.38	25	3
			SHDDEEGAGNPPIPEQDRPLLR	12	61664.5	9.48	14.37	78.8	4.29	25	3
			TFGISLEEVLVNEFTR	12	61664.5	9.48	19.33	86	4.25	25	3
hypothetical protein LOC23732	115648140	C9orf4	QHPGVWASLLLLLLTGPA	2	31629.9	7.55	10.76	80.6	6.74	25	2
			VDPFAKIKVDD	2	31629.9	7.55	13.74	88.5	4.68	25	3
hypothetical protein LOC345651	63055057	ACTBL2	DLTDYLMK	2	42003.4	5.39	12.7	89.8	4.21	25	2
			SYELPDGQVITIGNER	2	42003.4	5.39	21.01	92.3	4.14	25	2
hypothetical protein LOC374882	63055043	TMEM205	GLGGEVPGSHQGPDPYR	1	21197.9	8.91	18.3	93.1	5.32	25	3
hypothetical protein LOC414919	49169841	C8orf82	LALTMDLAPLLLAAR	1	23889.5	9.37	13.28	70.7	5.84	25	2
hypothetical protein LOC55004	8923579	C11orf59	TASNIIDVSAADSQGMEQHEYMDR	1	17744.9	5.01	20.77	91.8	4.1	25	3
hypothetical protein LOC55719	150456436	FAM178A	ETLKSLEE	2	131874	9.09	12.29	76.7	4.52	25	2
			LNFNQTPAATGKPPAL	2	131874	9.09	12.92	83.5	8.75	19	3
hypothetical protein LOC56948	116812630	C14orf124	AFIPLPSAVVQAVFGR	1	31076.8	9.09	17.13	73.1	9.79	25	2
hypothetical protein LOC57481	154090979	KIAA1210	AGIDFGSRKASAAQPIPENMDN	2	187022	8.72	12.36	74.5	4.56	25	3
			KLFGVRLKRAPPSQKYK	2	187022	8.72	11.47	80.8	11.22	25	2

hypothetical protein LOC57578	150456444	KIAA1409	GIDHQTVHQLITVLMK	3	275435.4	5.87	14.39	85.6	6.92	25	3
			KDWKMRFEAVEK	3	275435.4	5.87	12	78.8	8.5	25	3
			SFKQKSLDIGNADSLLFTLDE	3	275435.4	5.87	14.28	73.5	4.46	25	3
hypothetical protein LOC57687	24308257	VAT1L	TPLVPGFECSGIVEALGDSVK	2	45899.7	4.98	12.72	72.8	4.14	25	2
			VIAGFSLLNLLFK	2	45899.7	4.98	14.29	80.6	8.72	25	2
hypothetical protein LOC84233	14150017	TMEM126A	GNILSYWIR	2	88207.3	9.36	14.45	90.5	8.75	19	2
			IAAGLPMAGIPFLTTDLTYR	2	88207.3	9.36	18.78	90	5.83	25	2
hypothetical protein LOC84284	14150100	C1orf57	SSGVPVDGFYTEEVR	1	20713.2	9.6	12.28	82.7	4.14	18	2
hypothetical protein LOC84293	148596959	C10orf57 C10or	SMLDQLGVPLYAVVK	2	83654.4	8.92	10.75	74.6	5.55	21	2
			VNLLSVLEAAK	2	83654.4	8.92	10.82	75.7	5.97	22	2
hypothetical protein LOC89927	24308354	C16orf45	DFLVDDAEVER	7	23731.8	5.93	20.96	95.7	3.77	25	2
			FMMDDIQLCK	7	23731.8	5.93	14.57	81.9	4.21	25	2
			LREQEEDKEMADFLR	7	23731.8	5.93	12.74	78.6	4.36	25	3
			NQLDIISMAETTMMPEEIELEMAK	7	23731.8	5.93	19.94	89.6	3.83	25	3
			QELQNLVAIPEK	7	23731.8	5.93	13.87	86.3	4.53	25	2
			QSLSTHLEAEKPLR	7	23731.8	5.93	12.05	72.1	6.76	25	3
			YGAVEETAWK	7	23731.8	5.93	15.47	90.1	4.53	25	2
hypothetical protein LOC9556	4758940	C14orf2	NIWIPMKPYYTK	1	13711.7	10.08	13.28	80.7	9.53	25	2
inorganic pyrophosphatase 2 isoform 1 precursor	29171702	PPA2	ILGILALIDEGETDWK	1	24950.3	7.06	13.13	78.5	3.91	21	2
inorganic pyrophosphatase 2 isoform 2 precursor	77812678	PPA2	ILGILALIDEGETDWK	1	25038.4	8.69	13.13	78.5	3.91	21	2
inorganic pyrophosphatase 2 isoform 3 precursor	77812680	PPA2	ILGILALIDEGETDWK	1	25038.4	8.12	13.13	78.5	3.91	21	2
insulin-like growth factor 2	4504609	IGF2	AYRPSETLCGGELVDTLQFVCGDR	4	20140.5	9.5	20.13	97.3	4.32	25	3
			FFQYDTWK	4	20140.5	9.5	10.44	75.5	5.83	25	2
			GIVEECCFR	4	20140.5	9.5	17.93	92.9	4.53	25	2
			SCDLALLETYCATPAK	4	20140.5	9.5	22.08	98.7	4.37	25	2
insulin-like growth factor binding protein 2, 36kDa	55925576	IGFBP2	GDPECHLFYNEQQEAR	6	35137.8	7.48	17	72.5	4.4	25	3
			GECWCVNPNTGK	6	35137.8	7.48	17.8	87.1	5.99	25	2
			LAACGPPPVAPPAAVAAVAGGAR	6	35137.8	7.48	13.81	82.4	8.25	25	2
			LEGEACGVYTPR	6	35137.8	7.48	16.15	91.8	4.53	25	2
			LIQGAPTIR	6	35137.8	7.48	15.74	93.7	9.75	25	2
			MPCAELVR	6	35137.8	7.48	17.37	91.8	5.75	25	2
interleukin 6 receptor isoform 1 precursor	4504673	IL6R	PEDNATVHWVLRKPAAGSHPSRWA	2	51547.8	8.56	12.43	85.9	9.18	23	3
			SRSPPAENEVSTPMQAL	2	51547.8	8.56	14.17	71.2	4.53	25	3
internexin neuronal intermediate filament protein, alpha	14249342	INA	EYQDLLNVK	4	55390.9	5.34	12.55	93.2	4.37	25	2
			HSAEVAGYQDSIGQLENDLR	4	55390.9	5.34	15.15	76	4.31	25	3
			RPPASDGLDLSQAAAR	4	55390.9	5.34	14.74	97.4	5.96	15	3
			SNVASSAACSSASSLGLGLAYR	4	55390.9	5.34	14.99	80.9	7.94	25	3
iron-sulfur cluster assembly enzyme isoform ISCU2 precursor	56699456	ISCU	NVGTGLVGAPACGDVMK	2	17999.1	9.54	12.32	85	5.83	20	2
			TFGCGSAIASSSLATEWVK	2	17999.1	9.54	11.44	73.6	5.66	25	2
Kallmann syndrome 1 protein precursor	119395746	KAL1	AEAVFFTTPPCSALK	2	76112.4	9.33	13.22	80.3	6.04	25	2
			LEVQVLTPGGEGPATIK	2	76112.4	9.33	14.65	88	4.53	25	2
keratin 10	40354192	KRT10	ALEESNYELEGK	1	82879.6	5.09	14.61	81.1	4.09	25	2
keratin 2	47132620	KRT2	AQYEEIAQR	1	65433.2	8.07	16.64	95.2	4.53	21	2
keratin 25	31559829	KRT25	DAEAWFNEK	1	51868.9	5	17.06	89.5	4.14	25	2
keratin 25D	114431246	KRT28	DAEAWFNEK	1	50567.7	5.33	17.06	89.5	4.14	25	2
keratin 26	31559815	KRT26	VTMQNLNDR	1	51868.9	4.89	14.29	87.6	5.81	21	2
keratin 27	153945736	KRT27	DAEAWFNEK	1	270635.7	4.98	17.06	89.5	4.14	25	2
keratin 4	109255249	KRT4	AQYEEIAQR	1	63911.4	8.51	16.64	95.2	4.53	21	2
keratin 76	153791670	KRT76	AQYEEIAQR	1	65841.1	8.38	16.64	95.2	4.53	21	2
keratin 77	45597458	KRT77	FLEQQNQVLQTK	1	93518.9	5.73	16.98	86	6	25	2
kinectin 1 isoform a	33620775	KTN1	ETLYDEVLAK	4	156276	5.52	11.08	90.1	4.14	18	2
			ETMSVSLNQTVTQLQQLLQAVNQQLTK	4	156276	5.52	13.15	74.7	6.1	25	3
			LQALANEQAAAAHELEK	4	156276	5.52	14.26	88	4.75	25	3
			LSDALAVEDDQVAPVPLNVVETSSSVR	4	156276	5.52	17.68	89.9	3.77	25	3
kinectin 1 isoform b	118498362	KTN1	ETLYDEVLAK	4	111025.1	5.59	11.08	90.1	4.14	18	2
			ETMSVSLNQTVTQLQQLLQAVNQQLTK	4	111025.1	5.59	13.15	74.7	6.1	25	3
			LQALANEQAAAAHELEK	4	111025.1	5.59	14.26	88	4.75	25	3
			LSDALAVEDDQVAPVPLNVVETSSSVR	4	111025.1	5.59	17.68	89.9	3.77	25	3
kinectin 1 isoform c	118498368	KTN1	ETLYDEVLAK	4	111025.1	5.57	11.08	90.1	4.14	18	2
			ETMSVSLNQTVTQLQQLLQAVNQQLTK	4	111025.1	5.57	13.15	74.7	6.1	25	3
			LQALANEQAAAAHELEK	4	111025.1	5.57	14.26	88	4.75	25	3
			LSDALAVEDDQVAPVPLNVVETSSSVR	4	111025.1	5.57	17.68	89.9	3.77	25	3
l(3)mbt-like isoform I	117938328	L3MBTL	FTAHHCLSGCPL	2	85917.2	5.7	13.18	75	6.9	25	3
			GSGHVTGKFTAHHCLSGCPLAERNQ	2	85917.2	5.7	14.18	86.5	8.08	25	3
L-3-hydroxyacyl-Coenzyme A dehydrogenase precursor	94557308	HADH	FAGLHFFNPVPVMK	3	34293.7	8.88	13.67	76.6	8.76	23	3
			LGAGYPMGPFELLDYVGLDTTK	3	34293.7	8.88	18.05	90.4	4.03	25	2
			LLVPYLMEAIR	3	34293.7	8.88	13.39	88.4	6	21	2
L1 cell adhesion molecule isoform 1 precursor	4557707	L1CAM	DLQELGDSDKYFIEDGR	3	140003.5	5.84	12.7	74.8	3.96	25	3
			LDCQVQGRPQPEVTWR	3	140003.5	5.84	14.05	87.3	6.06	22	3
			LVLSDLHLLTQSQVR	3	140003.5	5.84	18.03	93.2	6.74	25	3
lipase A precursor	51317399	LIPA	DEMAKYDLPASINFILNKTGQEQ	2	45419.3	6.43	15	85.5	4.32	25	3
			DLFGDKEFLPQSAFLK	2	45419.3	6.43	13.88	71.7	4.56	25	3
lysosomal-associated membrane protein 1	112380628	LAMP1	FFLQGIQLNTILPDAR	1	44882.6	9	13.18	83.7	5.84	25	2
lysozyme precursor	4557894	LYZ	STDYGIFQINSR	2	16537.1	9.38	17.78	90.3	5.55	25	2
			TPGAVNACHLSCSALLQDNIADAVACAK	2	16537.1	9.38	12	75.4	5.18	25	3
lysyl hydroxylase precursor	32307144	PLOD1	AQVEEFLAQHGSEYQSVK	4	83550.6	6.47	13.14	83.3	4.75	25	3
			FLGSGGFIGYAPNLSK	4	83550.6	6.47	13.32	85.7	8.59	21	2
			IFQNLDGALDEVVLK	4	83550.6	6.47	17.27	80.7	4.03	25	2
			SEDYVDIVQGR	4	83550.6	6.47	16.36	90.5	4.03	25	2
major histocompatibility complex, class I, A precursor	24797067	HLA-A	DGEDQTQDTELVETRPAGDGTFQK	3	78411.3	5.65	12.9	74	3.9	25	3
			FIAVGYVDDTQFVR	3	78411.3	5.65	22.13	97.3	4.21	25	2
			WAAVVVPSGEEQR	3	78411.3	5.65	13.69	86.8	4.53	25	2
major histocompatibility complex, class I, E precursor	62912479	HLA-E	WAAVVVPSGEEQR	1	40057.8	5.52	13.69	86.8	4.53	25	2
major histocompatibility complex, class I, G precursor	4504415	HLA-G	WAAVVVPSGEEQR	1	38224.3	5.47	13.69	86.8	4.53	25	2
manganese superoxide dismutase isoform A precursor	67782305	SOD2	AIWNVINWENVTER	4	24750.3	8.34	16.74	81.7	4.53	23	2
			GDVTAQIALQPALK	4	24750.3	8.34	18.39	95.9	5.84	25	2
			GELLEAIK	4	24750.3	8.34	14.11	91.4	4.53	25	2
			HHAAYVNNLNVTEEK	4	24750.3	8.34	16.22	82.2	6	25	3
manganese superoxide dismutase isoform B precursor	67782309	SOD2	AIWNVINWENVTER	3	20723.8	8.36	16.74	81.7	4.53	23	2
			GELLEAIK	3	20723.8	8.36	14.11	91.4	4.53	25	2
			HHAAYVNNLNVTEEK	3	20723.8	8.36	16.22	82.2	6	25	3
mannosidase, alpha, class 1A, member 1	24497519	MAN1A1	AEWKGGLLE	2	72968.9	6.04	12.15	76	4.86	25	2
			GGVLGGGLGGGGGRKGSGPAALR	2	72968.9	6.04	12.51	70.9	12.01	25	3
mannosidase, alpha, class 2A, member 2	51477716	MAN2A2	FVVLFNPLEQER	4	130539.3	6.36	11.42	79.2	4.53	25	2
			GAEVLYSLAAAHAR	4	130539.3	6.36	14	82	6.75	25	3
			SNVLLVPLGDDFR	4	130539.3	6.36	11.91	78.6	4.21	25	2
			VIDSGTSDFALSNR	4	130539.3	6.36	12.23	74.1	4.21	25	2
mel transforming oncogene	16933567	RAB8A	LLLIGDSGVGK	2	23668.3	9.15	12.03	89.5	5.84	18	2
			TFISTIGIDFKIRTI	2	23668.3	9.15	10.16	82.5	8.41	25	2
metadherin	30520310	MTDH	EEAAAVPAAAPDDLALLK	3	63823.2	9.33	13.23	90	3.91	25	2
			KREEAAAVPAAAPDDLALL	3	63823.2	9.33	14.57	82.8	4.32	25	3
			SWQDELAQQAEEGSAR	3	63823.2	9.33	21.32	97.8	4	25	2
metaxin 1 isoform 1	38569475	MTX1	ISNPWQSPSGTLPALR	2	120839.9	9.8	12.57	88.8	9.75	17	2
			QTPAGPETEEEPYR	2	120839.9	9.8	13.84	77.8	4.09	22	2
metaxin 1 isoform 2	38569477	MTX1	ISNPWQSPSGTLPALR	2	120839.9	10.03	12.57	88.8	9.75	17	2
			QTPAGPETEEEPYR	2	120839.9	10.03	13.84	77.8	4.09	22	2
microfilament and actin filament cross-linker protein isoform a	33188445	MACF1	MEITAPDSQGKTGSILPSVGSSVGSVN	2	670140.2	5.27	12.57	76.5	4.37	25	3
			WLAEVEDKLSSVFVKDFKQDVL	2	670140.2	5.27	12.47	73.8	4.44	21	3
microfilament and actin filament cross-linker protein isoform b	33188443	MACF1	MEITAPDSQGKTGSILPSVGSSVGSVN	2	670140.2	5.2	12.57	76.5	4.37	25	3
			WLAEVEDKLSSVFVKDFKQDVL	2	670140.2	5.2	12.47	73.8	4.44	21	3
microtubule-associated protein 1A	95147555	MAP1A	AVLDALLEGK	1	305486.6	4.85	14.87	92.3	4.37	18	2
microtubule-associated protein 1B	153945728	MAP1B	AVLDALLEGK	1	270635.7	4.73	14.87	92.3	4.37	18	2
midkine	4505135	MDK	FENWGACDGGTGTK	3	15585.3	9.84	13.89	85.9	4.37	25	2
			GGPGSECAEWAWGPCTPSSK	3	15585.3	9.84	18.87	84.1	4.53	25	2
			YKFENWGACDGGTGTK	3	15585.3	9.84	19.52	92.6	6.06	25	3
mitochondria-associated granulocyte macrophage CSF signaling molecule	27363461	Magmas	SAAASNLSGLSLQEAQQILNVSK	2	13824.8	9.69	17.93	90.4	5.72	25	3
			SVGGSFYLQSK	2	13824.8	9.69	13.42	76.7	8.31	25	2
mitochondrial ATP synthase, O subunit precursor	4502303	ATP5O	GEVPCTVTSASPLEEATLSELK	7	23277.4	9.97	21.03	95.3	4.09	25	2
			LSNTQGVVSAFSTMMSVHR	7	23277.4	9.97	18.28	88.5	9.75	25	3
			LVRPPVQVYGIEGR	7	23277.4	9.97	17.57	93.1	8.75	25	3
			SFLSQGQVLK	7	23277.4	9.97	13.72	76.2	8.47	25	2
			TDPSILGGMIVR	7	23277.4	9.97	19.49	89.3	5.5	25	2
			VAASVLNPYVK	7	23277.4	9.97	16.11	89.3	8.56	25	2
			YATALYSAASK	7	23277.4	9.97	12.14	72.5	8.5	25	2
mitochondrial malate dehydrogenase precursor	21735621	MDH2	EGVVECSFVK	7	35503.5	8.92	10.86	81.5	4.53	25	2
			FVFSLVDAMNGK	7	35503.5	8.92	17.93	90.6	5.84	25	2
			GYLGPEQLPDCLK	7	35503.5	8.92	13.43	79.3	4.37	25	2
			IFGVTTLDIVR	7	35503.5	8.92	17.72	93.4	5.84	25	2
			SQETECTYFSTPLLLGK	7	35503.5	8.92	14.98	84	4.53	25	2
			TIIPLISQCTPK	7	35503.5	8.92	21.86	94.3	7.88	25	2
			VAVLGASGGIGQPLSLLLK	7	35503.5	8.92	18.35	90.7	8.72	25	2
mitochondrial short-chain enoyl-coenzyme A hydratase 1 precursor	12707570	ECHS1	AQFAQPEILIGTIPGAGGTQR	2	31371.6	8.34	16.64	87.6	6.05	25	2
			ICPVETLVEEAIQCAEK	2	31371.6	8.34	13.11	71.2	4.09	25	2
mitogen-activated protein kinase interacting protein 1	11496277	MAPKSP1	ELAPLFEELR	1	13622.8	6.72	19.46	97.2	4.25	25	2
myeloid/lymphoid or mixed-lineage leukemia 3	91718902	MLL3	IPTLQRSVS	3	541372.7	6.07	10.32	81	9.75	25	2
			PNSAPAHLPLNPPRIQ	3	541372.7	6.07	13.12	72.1	10.18	25	3
			TLTGGPARYINHSCAPNCVAEVV	3	541372.7	6.07	13.06	86.8	6.41	25	3
myofibrillogenesis regulator 1 isoform 1	116642887	PNKD	EEPEPLSPELEYIPR	1	15396.7	9.22	14.62	79.5	3.98	25	2
myofibrillogenesis regulator 1 isoform 3	116642885	PNKD	EEPEPLSPELEYIPR	1	15396.7	10.58	14.62	79.5	3.98	25	2
myosin, heavy polypeptide 10, non-muscle	41406064	MYH10	ATISALEAKIGQLEEQLEQEAKERA	2	229000.3	5.44	12.51	85.5	4.48	25	3
			LLGMNVMEFTRAILTPRIKVG	2	229000.3	5.44	12.7	81.3	10.84	25	3
myosin, light chain 6, alkali, smooth muscle and non-muscle isoform 1	17986258	MYL6	ALGQNPTNAEVLK	1	16930.2	4.56	15.82	87.6	6.05	25	2
N-acylsphingosine amidohydrolase (acid ceramidase) 1 isoform b	30089930	ASAH1	DAMWIGFLTR	4	44768.8	7.99	11.9	80.6	5.84	18	2
			ESLDVYELDAK	4	44768.8	7.99	11.63	86.2	3.91	25	2
			GAVPWYTINLDLPPYK	4	44768.8	7.99	11.04	74	5.83	20	2
			STYPPSGPTYR	4	44768.8	7.99	11.86	74.6	8.31	25	2
N-ethylmaleimide-sensitive factor attachment protein, alpha	47933379	NAPA	AIAHYEQSADYYKGEESNSSANK	3	33232.9	5.23	17.35	85	4.83	25	3
			AIDIYEQVGTNAMDSPLLK	3	33232.9	5.23	14.89	80.8	4.03	25	3
			EAEAMALLAEAER	3	33232.9	5.23	13.43	71.9	4.09	25	2
N-ethylmaleimide-sensitive factor attachment protein, beta	44917606	NAPB	AIAHYEQSADYYKGEESNSSANK	1	31341.6	5.32	17.35	85	4.83	25	3
Na+/K+ -ATPase beta 1 subunit isoform a	4502277	ATP1B1	SYEAYVLNIVR	3	35061.5	8.74	11.19	74.8	5.72	25	2
			VGNVEYFGLGNSPGFPLQYYPYYGK	3	35061.5	8.74	23.34	95.1	5.97	25	3
			YNPNVLPVQCTGK	3	35061.5	8.74	12.05	70.1	8.2	25	2
NAD(P)H:quinone oxidoreductase type 3, polypeptide A2	49574502	CYB5R1	DIILREDLEELQAR	4	34095	9.41	12.03	73.3	4.18	24	3
			GFVTADMIR	4	34095	9.41	14.17	87.3	5.84	21	2
			LGMIAGGTGITPMLQLIR	4	34095	9.41	13.46	85.4	9.75	25	2
			RPQVTLLDPNEK	4	34095	9.41	14.48	79	6.07	25	3
NADH dehydrogenase (ubiquinone) 1 alpha subcomplex, 10, 42kDa precursor	4758768	NDUFA10	LLQYSDALEHLLTTGQGVVLER	1	16516.4	8.67	15.51	82.2	4.65	25	3
NADH dehydrogenase (ubiquinone) 1 alpha subcomplex, 11, 14.7kDa	28269681	NDUFA11	VTLNPPGTFLEGVAK	1	14852.2	8.95	14.67	83	5.97	25	2
NADH dehydrogenase (ubiquinone) 1 alpha subcomplex, 2, 8kDa	4505355	NDUFA2	ALENVLSGK	3	10921.6	9.62	15.87	95.4	6.05	25	2
			ANPDLPILIR	3	10921.6	9.62	11.84	80	5.88	25	2
			YAFGQETNVPLNNFSADQVTR	3	10921.6	9.62	20.04	87.8	4.37	25	3
NADH dehydrogenase (ubiquinone) 1 alpha subcomplex, 3, 9kDa	4758772	NDUFA3	ATPYNYPVPVR	2	9278.9	8	14.33	84.2	8.63	25	2
			SVMINKATPYNYPVPVRDD	2	9278.9	8	12.55	75.8	5.86	25	3
NADH dehydrogenase (ubiquinone) 1 alpha subcomplex, 5	4826848	NDUFA5	ILDVLEEIPK	1	13458.8	5.75	16.42	86.9	4.14	25	2
NADH dehydrogenase (ubiquinone) 1 alpha subcomplex, 6, 14kDa	51317370	NDUFA6	EVPNTVHQFQLDITVK	4	17870.9	10.14	17.68	87.1	5.32	25	3
			FFHETEAPRPK	4	17870.9	10.14	12.98	72.8	6.76	25	3
			GKMAGSGVRQATSTASTFVKPIFSR	4	17870.9	10.14	12.04	71.9	12.02	25	3
			VVDLLVIK	4	17870.9	10.14	13.47	99.1	5.81	15	2

NADH dehydrogenase (ubiquinone) 1 alpha subcomplex, 7, 14.5kDa	103472001	NDUFA7	ALVSGKPAESSAVAATEK	2	12551.5	10.19	15.11	83.3	6.18	25	3
			ESVPPSIIMSSQK	2	12551.5	10.19	14.38	88.9	6.1	25	2
NADH dehydrogenase (ubiquinone) 1 alpha subcomplex, 8, 19kDa	7657369	NDUFA8	FDECVLDK	5	20105.2	7.58	12.48	86.1	4.03	25	2
			LGWVRPDLGELSK	5	20105.2	7.58	16.97	97.1	6.07	21	3
			PGIVELPTLEELKVDEVK	5	20105.2	7.58	21.47	89.9	4.25	25	3
			PRPDPSPEIEGDLQPATHGSR	5	20105.2	7.58	19.52	89.3	4.75	25	3
			TDRPLPENPYHSR	5	20105.2	7.58	15.14	77.2	6.42	25	3
NADH dehydrogenase (ubiquinone) 1 alpha subcomplex, 9, 39kDa	6681764	NDUFA9	FGPIPLGSLGWK	3	42509.8	9.81	11.61	78.6	8.75	20	2
			LFLPFPLPLFAYR	3	42509.8	9.81	11.88	91.7	8.75	11	2
			LPHLPGLEDLGIQATPLELK	3	42509.8	9.81	12.08	80.4	4.65	25	3
NADH dehydrogenase (ubiquinone) 1 beta subcomplex, 1, 7kDa	38569473	NDUFB1	ELQPSEEVTWK	2	120839.9	9.02	15.82	92.2	4.25	25	2
			VNLLQIVR	2	120839.9	9.02	15.98	81.8	9.72	25	2
NADH dehydrogenase (ubiquinone) 1 beta subcomplex, 10, 22kDa	4758774	NDUFB10	AFDLIVDRPVTLVR	3	20776.8	8.72	15.42	87.4	6.01	21	3
			RTPVQPNPIVYMMK	3	20776.8	8.72	12.76	76	9.99	25	3
			YQDLGAYSSAR	3	20776.8	8.72	19.02	95.4	5.83	25	2
NADH dehydrogenase (ubiquinone) 1 beta subcomplex, 2, 8kDa precursor	4758778	NDUFB2	QFPQLTR	1	12058.5	5.47	13.38	75	9.75	25	2
NADH dehydrogenase (ubiquinone) 1 beta subcomplex, 3, 12kDa	4505361	NDUFB3	IEGTPLETIQK	3	10921.6	9.19	12.42	80.9	4.53	25	2
			MELPDYR	3	10921.6	9.19	10.57	92.4	4.37	17	2
			SVSFSDVFFK	3	10921.6	9.19	17.71	95.3	5.55	25	2
NADH dehydrogenase (ubiquinone) 1 beta subcomplex, 4, 15kDa	6041669	NDUFB4	GLIENPALLR	2	15208.7	9.85	19.43	93.2	6	25	2
			TINVYPNFRPTPK	2	15208.7	9.85	13.38	74.4	9.99	25	2
NADH dehydrogenase (ubiquinone) 1 beta subcomplex, 5, 16kDa precursor	4505363	NDUFB5	TMAVLQIEAEK	1	21750.4	9.62	17.13	85.6	4.53	25	2
NADH dehydrogenase (ubiquinone) 1 beta subcomplex, 6, 17kDa isoform 1	4505365	NDUFB6	IFPGDTILETGEVIPPMK	3	15489.3	9.63	18.58	80.9	4.14	25	2
			WLKDQELSPR	3	15489.3	9.63	12.4	80.6	6.07	25	3
			YHVSEKPYGIVEK	3	15489.3	9.63	15.85	87.2	6.75	25	3
NADH dehydrogenase (ubiquinone) 1 beta subcomplex, 7, 18kDa	10764847	NDUFB7	DSFPNFLACK	2	16402.1	9.09	17.7	90.3	5.83	25	2
			RYLGDASVEPDPLQMPTFPPDYGFPER	2	16402.1	9.09	22.82	94.1	4.11	25	3
NADH dehydrogenase (ubiquinone) 1 beta subcomplex, 8, 19kDa	4826854	NDUFB8	DMFPGPYPR	3	21765.9	6.29	12.03	87.3	5.83	17	2
			DPWYSWDQPGLR	3	21765.9	6.29	17.23	91.8	4.21	25	2
			VEDYEPYPDDGMGYGDYPK	3	21765.9	6.29	17.26	90.4	3.66	25	2
NADH dehydrogenase (ubiquinone) 1 beta subcomplex, 9, 22kDa	6274550	NDUFB9	AMYPDYFAK	5	21831	8.58	11.56	80.5	5.88	25	2
			IFPDSPGGTSYER	5	21831	8.58	15.18	78.1	4.37	25	2
			QHPQPYIFPDSPGGTSYER	5	21831	8.58	20	82.7	5.32	25	3
			QLQEETPPGGPLTEALPPAR	5	21831	8.58	17.54	91.2	4.25	25	2
			YFACLMR	5	21831	8.58	12.51	96.5	8.22	15	2
NADH dehydrogenase (ubiquinone) 1, alpha/beta subcomplex, 1, 8kDa	4826852	NDUFAB1	SDMPPLTLEGIQDR	1	13458.8	4.82	19.2	93.5	4.03	25	2
NADH dehydrogenase (ubiquinone) Fe-S protein 3, 30kDa (NADH-coenzyme Q reductase)	4758788	NDUFS3	DFPLSGYVELR	7	30241.7	6.98	22.28	93.3	4.37	25	2
			FEIVYNLLSLR	7	30241.7	6.98	10.55	80.9	6	21	2
			KFDLNSPWEAFPVYR	7	30241.7	6.98	15.37	87.4	6.07	25	3
			QLSAFGEYVAEILPK	7	30241.7	6.98	16.92	87.8	4.53	25	2
			SLVDLTAVDVPTR	7	30241.7	6.98	18.97	92.9	4.21	25	2
			TYTDELTPIESAVSVFK	7	30241.7	6.98	12.27	81.8	4.14	25	2
			VVAEPVELAQEFR	7	30241.7	6.98	21.09	89.4	4.25	25	2
NADH dehydrogenase (ubiquinone) Fe-S protein 4, 18kDa (NADH-coenzyme Q reductase)	4505369	NDUFS4	AQDQTQDTQLITVDEK	3	15489.3	10.3	15.64	84.9	3.84	25	2
			LDITTLTGVPEEHIK	3	15489.3	10.3	19.17	94.4	4.65	25	3
			WENPLMGWASTADPLSNMVLTFSTK	3	15489.3	10.3	17.64	88.2	4.37	25	3
NADH dehydrogenase (ubiquinone) Fe-S protein 5, 15kDa (NADH-coenzyme Q reductase)	4758790	NDUFS5	EWIECAHGIGYTR	4	12517.6	9.27	13.54	89.8	5.4	25	3
			FGLNIDR	4	12517.6	9.27	16.42	92.5	5.84	25	2
			IEYDDFVECLLR	4	12517.6	9.27	12.42	82.9	3.92	25	2
			WLTIQSGEQPYK	4	12517.6	9.27	16.13	91.5	6	25	2
NADH dehydrogenase (ubiquinone) Fe-S protein 6, 13kDa (NADH-coenzyme Q reductase)	4758792	NDUFS6	EVNENFAIDLIAEQPVSEVETR	1	13711.7	8.58	15.01	72.4	3.83	25	3
NADH dehydrogenase (ubiquinone) Fe-S protein 8, 23kDa (NADH-coenzyme Q reductase)	4505371	NDUFS8	TLLWTELFR	1	23705.3	6	18.21	92.4	5.66	25	2
NADH dehydrogenase (ubiquinone) flavoprotein 2, 24kDa	10835025	NDUFV2	AAAVLPVLDLAQR	2	27363.7	8.21	14.42	81.3	5.88	25	2
			NSDSILEAIQK	2	27363.7	8.21	17.82	88.2	4.37	25	2
NADH dehydrogenase subunit 4	17981862	MT-ND4 MT-ND	LIVPTIMLLPLTWLSK	1	7991.7	9.4	12.35	73.9	8.75	25	2
NADH-ubiquinone oxidoreductase Fe-S protein 7	38524585	NDUFS1	QSDVMIVAGTLTNK	3	23579.7	10.02	18.61	94.7	5.84	25	2
			VHQSVATDGPSSTQPALPK	3	23579.7	10.02	17.99	88.4	6.71	25	3
			VYDQMPEPR	3	23579.7	10.02	16.25	86.8	4.37	24	2
neural proliferation, differentiation and control, 1	20149617	NPDC1	KAPGSPAAPRISPGDQRLAQSA	2	34543.6	6.59	12.24	81.4	10.84	25	3
			LEDEIDFLAQELAR	2	34543.6	6.59	21.66	87.2	3.83	25	2
neurofilament, heavy polypeptide 200kDa	32483416	NEFH	EYQDLLNVK	1	111838.7	5.94	12.55	93.2	4.37	25	2
neurofilament, light polypeptide 68kDa	105990539	NEFL	EYQDLLNVK	1	61516.9	4.64	12.55	93.2	4.37	25	2
neurofilament, medium polypeptide 150kDa	4885513	NEFM	EYQDLLNVK	1	70025.5	4.9	12.55	93.2	4.37	25	2
neuron navigator 2 isoform 1	38044284	NAV2	IPKGGKLNSAKKEPMAPSHSGI	2	261409.2	9.13	12.14	74.5	10	25	3
			PRKQDSDPHLDRN	2	261409.2	9.13	13.74	75.3	7.17	22	3
neuron navigator 2 isoform 2	38044282	NAV2	IPKGGKLNSAKKEPMAPSHSGI	2	261409.2	9.11	12.14	74.5	10	25	3
			PRKQDSDPHLDRN	2	261409.2	9.11	13.74	75.3	7.17	22	3
neuronal pentraxin I precursor	55770878	NPTX1	LTPGEVYNLATCSTK	2	357528.9	6.16	10.5	75.3	5.99	20	2
			TPAAETLSQLGQTLQSLK	2	357528.9	6.16	13.82	76.4	5.66	25	2
neuropeptide Y	4505449	NPY	SKPDNPGEDAPAEDMAR	1	10851.5	6.57	18.73	87.9	4.1	25	3
nicastrin precursor	24638433	NCSTN	SFFWNVAPGAESAVASFVTQLAAAEALQK	1	78411.3	5.67	16.12	81.4	4.53	25	3
nidogen 1 precursor	115298674	NID1	ALEGLQYPFAVTSYGK	8	136377.8	5.12	14.08	81.9	6.04	25	2
			MVYWTDITEPSIGR	8	136377.8	5.12	15.7	88	4.37	25	2
			QAEVTFVGHPGNLVIK	8	136377.8	5.12	12.42	79.4	6.75	25	3
			QDLGSPEGIAVDHLGR	8	136377.8	5.12	12.4	70.4	4.54	25	3
			TIIRQDLGSPEGIAVDHL	8	136377.8	5.12	12.95	70.5	4.53	25	3
			VLFETDLVNPR	8	136377.8	5.12	17.61	93.4	4.37	25	2
			VYYREDLSPSITQR	8	136377.8	5.12	14.32	81.9	6.04	25	3
			YALSNSIGPVR	8	136377.8	5.12	13.56	83	8.75	25	2
nipsnap homolog 1	4505399	NIPSNAP1	GWDENVYYTVPLVR	2	33324.2	9.35	10.76	77.9	4.37	25	2
			MGPNIYELR	2	33324.2	9.35	12.35	78.5	5.75	25	2
nipsnap homolog 3A	22267436	NIPSNAP3A	ESVNYLVSQQNMLLIPTSFSPLK	2	28466.8	9.21	13.84	75.2	6.1	25	3
			LVGVFHTEYGALNR	2	28466.8	9.21	13.78	91.5	6.75	22	3
NME1-NME2 protein	66392203	NME2	VMLGETNPADSKPGTIR	2	27128.6	9.06	14.39	91.8	6.04	25	3
			YMNSGPVVAMVWEGLNVVK	2	27128.6	9.06	18.15	95	6	25	3
non-SMC condensin I complex, subunit D2	41281521	NCAPD2	AQILQGCAKQALEKLEEKRTSQEDPK	2	127395.7	6.15	12.24	83.8	6.35	25	3
			LSIKHLPPQLRAFQAAFRAQGPLAM	2	127395.7	6.15	12.4	74.8	12.01	25	3
oxygen regulated protein precursor	5453832	HYOU1	AANSLEAFIFETQDK	9	111335.9	5.16	14.37	81.2	4.14	25	2
			DAVVYPILVEFTR	9	111335.9	5.16	15.98	90.5	4.37	25	2
			LGNTISSLFGGGTTPDAK	9	111335.9	5.16	12.6	77.2	5.84	25	2
			LYQPEYQEVSTEEQREEISGK	9	111335.9	5.16	18.42	79.3	4.21	24	3
			NINADEAAAMGAVYQAAALSK	9	111335.9	5.16	13.84	85.1	4.37	22	2
			TLGGLEMELR	9	111335.9	5.16	10.01	96.6	4.53	11	2
			TVLSANADHMAQIEGLMDDVDFK	9	111335.9	5.16	12.42	78.6	4.03	25	3
			VAIVKPGVPMEIVLNK	9	111335.9	5.16	16.26	83.6	8.56	25	3
			VLQLINDNTATALSYGVFR	9	111335.9	5.16	16.94	79.8	5.8	25	3
paraoxonase 1	19923106	PON1	EVQPVELPNCNLVK	6	39731.5	5.08	17.21	89	4.53	25	2
			IFFYDSENPPASEVLR	6	39731.5	5.08	12.1	76.8	4.14	23	3
			ILLMDLNEEDPTVLELGITGSK	6	39731.5	5.08	19.35	83.3	3.83	25	3
			IQNILTEEPK	6	39731.5	5.08	11.96	74.8	4.53	25	2
			VVAEGFDFANGINISPDGK	6	39731.5	5.08	15.26	82	4.03	25	3
			YVYIAELLAHK	6	39731.5	5.08	13.3	87.4	6.75	23	3
peptidylglycine alpha-amidating monooxygenase isoform a, preproprotein	21070984	PAM	AGIEVQEIK	10	96258.2	5.98	12.48	77	4.53	22	2
			ANILYAWAR	10	96258.2	5.98	13.52	85.9	8.79	23	2
			FVYQQIGLGPIEEDTILVIDPNNAAVLQSSGK	10	96258.2	5.98	12.42	70.2	3.91	25	3
			GSGGLNLGNFFASR	10	96258.2	5.98	12.44	82.5	9.75	25	2
			IPVDEEAFVIDFKPR	10	96258.2	5.98	16.16	70.8	4.32	25	3
			NLFYLPHGLSIDK	10	96258.2	5.98	15.81	90.7	6.74	22	3
			NVFAISYIPGLLFAVNGK	10	96258.2	5.98	11.23	77.3	8.59	25	2
			NYPMHVFAYR	10	96258.2	5.98	15.01	82.2	8.6	25	3
			QSPQLPQAFYPVGHPVDVSFGDLLAAR	10	96258.2	5.98	15.03	71.9	5.21	25	3
			REEEEVLDQGDFYSLLSK	10	96258.2	5.98	19.65	90.4	4.08	25	3
peptidylglycine alpha-amidating monooxygenase isoform c, preproprotein	21070980	PAM	AGIEVQEIK	9	96258.2	6	12.48	77	4.53	22	2
			ANILYAWAR	9	96258.2	6	13.52	85.9	8.79	23	2
			FVYQQIGLGPIEEDTILVIDPNNAAVLQSSGK	9	96258.2	6	12.42	70.2	3.91	25	3
			GSGGLNLGNFFASR	9	96258.2	6	12.44	82.5	9.75	25	2
			IPVDEEAFVIDFKPR	9	96258.2	6	16.16	70.8	4.32	25	3
			NLFYLPHGLSIDK	9	96258.2	6	15.81	90.7	6.74	22	3
			NVFAISYIPGLLFAVNGK	9	96258.2	6	11.23	77.3	8.59	25	2
			NYPMHVFAYR	9	96258.2	6	15.01	82.2	8.6	25	3
			QSPQLPQAFYPVGHPVDVSFGDLLAAR	9	96258.2	6	15.03	71.9	5.21	25	3
peptidylprolyl isomerase A (cyclophilin A)-like 4	30102944	PPIAL4G	IIPGFMCQGGDFTR	1	18181.9	9.32	19.52	92.3	5.83	25	2
peptidylprolyl isomerase B precursor	4758950	PPIB	DKPLKDVIIADCGK	6	23742.7	9.42	17.45	89.9	6.03	25	2
			FPDENFK	6	23742.7	9.42	12.46	80.7	4.37	25	2
			LLLPGPSAADEK	6	23742.7	9.42	16.45	89.6	4.37	25	2
			TVDNFVALATGEK	6	23742.7	9.42	12.29	80.2	4.37	25	2
			VIKDFMIQGGDFTR	6	23742.7	9.42	13.64	89.1	5.93	25	3
			VLEGMEVVR	6	23742.7	9.42	16.72	86.4	4.53	22	2
peripherin	21264345	PRPH	MALDIEIATYR	2	102642.1	5.37	19.72	96.6	4.37	25	2
			NLQEAEEWYK	2	102642.1	5.37	13.25	84.2	4.25	25	2
peroxiredoxin 1	4505591	PRDX1	GLFIIDDK	2	34404.4	8.27	10.1	82.3	4.21	20	2
			QITVNDLPVGR	2	34404.4	8.27	14.34	87.4	5.84	25	2
peroxiredoxin 2 isoform a	32189392	PRDX2	GLFIIDGK	4	21892	5.66	11.41	88	5.84	25	2
			KEGGLGPLNIPLLADVTR	4	21892	5.66	21.58	86.4	6.07	25	3
			LSEDYGVLK	4	21892	5.66	10.98	79.2	4.37	25	2
			QITVNDLPVGR	4	21892	5.66	14.34	87.4	5.84	25	2
peroxiredoxin 3 isoform a precursor	5802974	PRDX3	DLSLDDFK	3	27692.8	7.67	11.37	87.4	3.93	25	2
			DYGVLLEGSGLALR	3	27692.8	7.67	17.17	74.8	4.37	25	2
			PAVTQHAPYFK	3	27692.8	7.67	15.63	79.4	9.01	25	3
peroxiredoxin 5 precursor, isoform a	6912238	PRDX5	ALNVEPDGTGLTCSLAPNIISQL	6	22026.5	8.85	13.34	71.5	3.67	25	3
			ETDLLLDDSLVSIFGNR	6	22026.5	8.85	21.1	93.9	3.84	25	2
			FSMVVQDGIVK	6	22026.5	8.85	15.19	91.6	5.84	25	2
			GVLFGVPGAFTPGCSK	6	22026.5	8.85	17.38	82.8	8.22	25	2
			THLPGFVEQAEALK	6	22026.5	8.85	17	93.2	5.37	25	3
			VGDAIPAVEVFEGEPGNK	6	22026.5	8.85	18.26	92.4	4	25	2
peroxiredoxin 5 precursor, isoform b	32455260	PRDX5	ALNVEPDGTGLTCSLAPNIISQL	5	17394.2	9.02	13.34	71.5	3.67	25	3
			ETDLLLDDSLVSIFGNR	5	17394.2	9.02	21.1	93.9	3.84	25	2
			FSMVVQDGIVK	5	17394.2	9.02	15.19	91.6	5.84	25	2
			GVLFGVPGAFTPGCSK	5	17394.2	9.02	17.38	82.8	8.22	25	2
			VGDAIPAVEVFEGEPGNK	5	17394.2	9.02	18.26	92.4	4	25	2
peroxiredoxin 5 precursor, isoform c	32455262	PRDX5	ALNVEPDGTGLTCSLAPNIISQL	3	12836.9	9.35	13.34	71.5	3.67	25	3
			ETDLLLDDSLVSIFGNR	3	12836.9	9.35	21.1	93.9	3.84	25	2
			FSMVVQDGIVK	3	12836.9	9.35	15.19	91.6	5.84	25	2
phosphoglycerate kinase 1	4505763	PGK1	ACANPAAGSVILLENLR	3	44615	8.3	15.77	76.4	6.04	21	2
			ALESPERPFLAILGGAK	3	44615	8.3	12.43	94.5	6.19	16	3
			TGQATVASGIPAGWMGLDCGPESSKK	3	44615	8.3	13.79	83.7	5.73	25	3
phosphoinositide-3-kinase, catalytic, alpha polypeptide	54792082	PIK3CA	LLLSVKW	2	97205.9	6.88	10.49	94.9	8.75	17	2
			NWENPDIMSELLFQNNEIIFKNGD	2	97205.9	6.88	13.31	83.5	4.02	25	3
PKCI-1-related HIT protein	14211923	HINT2	AQQATPGGAAPTIFSR	1	17161.8	9.2	13.36	85.1	9.79	25	2
plasma glutathione peroxidase 3 precursor	6006001	GPX3	FLVGPDGIPIMR	4	25402.4	8.26	17.43	88	5.84	25	2
			MDILSYMR	4	25402.4	8.26	17.16	93.5	5.59	25	2
			NSCPPTSELLGTSDR	4	25402.4	8.26	22.92	97.8	4.37	25	2
			YVRPGGGFVPNFQLFEK	4	25402.4	8.26	16.09	77.9	8.59	20	3
plasminogen activator inhibitor type 1, member 2	24307907	SERPINE2	ASAATTAILIAR	20	44002.5	9.35	16.24	85.4	9.79	25	2
			DIVTVANAVFVK	20	44002.5	9.35	21.64	94.7	5.84	25	3
			DMIDNLLSPDLIDGVLTR	20	44002.5	9.35	21.3	96.7	3.77	25	3
			FTAVAQTDLK	20	44002.5	9.35	19.21	94.5	5.84	25	2
			FTAVAQTDLKEPLK	20	44002.5	9.35	15.09	88.5	6.07	25	3
			GIASVLGMLQLGADGR	20	44002.5	9.35	12.01	70	5.84	25	2

			HNPTGAVLFMGQINKP	20	44002.5	9.35	14.78	84.9	8.76	25	3
			IALPTESSTPLSAIIPHISTK	20	44002.5	9.35	14.84	76.8	6.75	25	3
			LAQLSVFR	20	44002.5	9.35	14.87	93.9	9.75	25	2
			LIALPTESSTPLSAIIPHISTK	20	44002.5	9.35	14.18	90.6	6.75	18	3
			LVLVNAVYFK	20	44002.5	9.35	13.27	88.4	8.59	25	2
			NKDIVTVANAVFVK	20	44002.5	9.35	19.03	93.5	8.59	25	3
			NVNFEDPASACDSINAWVK	20	44002.5	9.35	18.92	89.5	4.03	25	2
			SENLHVSHILQK	20	44002.5	9.35	13.29	84.1	6.66	21	3
			SLEELGSNTGIQVFNQIVK	20	44002.5	9.35	15.75	85	4.53	25	3
			SSPPWFIVDRPFLF	20	44002.5	9.35	12.38	76.8	5.55	25	2
			SSPPWFIVDRPFLFFIR	20	44002.5	9.35	12.09	80.7	9.32	25	3
			SYQVPMLAQLSVFR	20	44002.5	9.35	22.12	97.9	8.46	25	2
			TIDSWMSIMVPK	20	44002.5	9.35	17.67	89.8	5.5	25	2
			VLGITDMFDSSK	20	44002.5	9.35	15.94	92.4	4.21	25	2
plasminogen activator, tissue type isoform 1 preproprotein	4505861	PLAT	EFDDDTYDNDIALLQLK	3	44615	8.14	17.57	82.3	3.61	25	2
			VTNYLDWIR	3	44615	8.14	15.56	88.3	5.8	25	2
			VYTAQNPSAQALGLGK	3	44615	8.14	22.69	97.4	8.56	25	2
platelet derived growth factor D isoform 1 precursor	13376808	PDGFD	LANVVFFPR	2	42848.6	8.28	12.79	87.7	9.75	24	2
			YFNPESWQEDLENMYLDTPR	2	42848.6	8.28	13.51	79.8	3.84	25	3
pleiotrophin	4506281	PTN	YQFQAWGECDLNTALK	1	18942.1	9.66	13.38	85.4	4.37	22	2
polydom	148886654	SVEP1	ERISCGVPPPLENGFH	2	390172.8	5.32	13.42	71.7	5.4	25	3
			TRCETPLEFLNGKADIENRTTG	2	390172.8	5.32	12.56	70.5	4.87	25	3
polypeptide N-acetylgalactosaminyltransferase 2	4758412	GALNT2	NFYYAAVPSAR	2	64733.1	8.63	17.28	83.7	8.59	23	2
			WYLENVYPELR	2	64733.1	8.63	11.41	86	4.53	25	2
PREDICTED: similar to ADP-ribosylation factor 4	113415078	ARL1	LGEIVTTIPTIGFNVETVEYK	1	8131.6	5.5	20.74	84.8	4.25	25	3
PREDICTED: similar to AHNAK nucleoprotein isoform 1	113425016	AHNAK	KLDGAWLEGDLSVADKDVTTKDSR	2	667854.2	5.51	12.19	72	4.46	25	3
			QAPRAKLDSAQLEGDLSLADKD	2	667854.2	5.51	14.24	79.6	4.52	25	3
PREDICTED: similar to CG17807-PA	113411509	ALKBH8	EDPMIKQ	1	22671.2	6.48	17.57	74.1	4.37	25	2
PREDICTED: similar to CG9007-PA isoform 17	88961345	SETD5	IQKRNPNATELPLLPP	2	121283.1	8.67	14.72	88.4	8.75	23	3
			LSPVTPPPPNSGSKSPQLA	2	121283.1	8.67	10.31	77.9	8.75	25	2
PREDICTED: similar to creatine kinase, mitochondrial 1B precursor	89038979	CKMT1A CKMT1	GTGGVDTAATGGVFDISNLDR	1	10253.8	8.98	18.14	91.4	3.93	25	2
PREDICTED: similar to Guanine nucleotide-binding protein G(t), alpha-3 subunit (Gustducin alpha-3 chain)	113419695	GNAT3	LLLLGAGESGK	1	91899	9.75	14.13	87.4	6	25	2
PREDICTED: similar to HLA class I histocompatibility antigen, B-18 alpha chain precursor (MHC class I antigen B18)	113417848	HLA-B HLA-C	APWIEQEGPEYWDR	2	43345.5	5.99	16.95	83.6	4	25	2
			WAAVVVPSGEEQR	2	43345.5	5.99	13.69	86.8	4.53	25	2
PREDICTED: similar to loss of heterozygosity, 11, chromosomal region 2	88966020	VWA5B2	ALGDPATPTEGPR	12	136258.8	6.08	18.27	91.7	4.37	24	2
			AQHLPDGLDLAALK	12	136258.8	6.08	15.92	79.6	5.21	25	3
			DSEQSTDALTDPVTDPGPNPSDTAIWR	12	136258.8	6.08	17.72	86.4	3.61	25	3
			EIPALYPGDQLLGYCSLFR	12	136258.8	6.08	17.99	92.5	4.37	25	2
			GSDTEASEGAEGLGGTDLR	12	136258.8	6.08	18.83	89	3.83	25	2
			KALEPALSDISVDWFVPDTVEALLTPR	12	136258.8	6.08	12.84	73.6	4.11	25	3
			LDAPFCAAVR	12	136258.8	6.08	13.13	84.4	5.83	24	2
			NPQPQPVDGVFVYPLAEAEVVSGFEAEAAGR	12	136258.8	6.08	15.02	87	3.91	25	3
			QLFLLTAASPMAATTHR	12	136258.8	6.08	16.71	89	9.75	25	3
			SCPLPAPTPAPFK	12	136258.8	6.08	12.62	74.6	7.94	25	2
			SLPPQTLINLAVFGTLVQPLFPESR	12	136258.8	6.08	16.53	81	5.72	25	3
			VSSAPSCFTCPVAVDATTR	12	136258.8	6.08	11.98	78.4	5.79	19	2
PREDICTED: similar to NADH dehydrogenase (ubiquinone) 1 beta subcomplex, 4, 15kDa	88974095	MT-ND4 MT-ND	GLIENPALIR	1	16265.9	9.83	19.43	93.2	6	25	2
PREDICTED: similar to neuronal pentraxin I precursor	113427645	NPTX1	LTPGEVYNLATCSTK	2	410203.8	9.92	10.5	75.3	5.99	20	2
			TPAAETLSQLGQTLQSLK	2	410203.8	9.92	13.82	76.4	5.66	25	2
PREDICTED: similar to Olfactory guanylyl cyclase GC-D precursor (Guanylate cyclase, olfactory)	113422501	GUCY2D	KCSSLPTSTPSVGRQSAEGRRARGTGV	2	60384	8.56	12.32	81.5	11.54	25	3
			RSDXAEGEGHRRDLLGGGKAG	2	60384	8.56	12.66	71.4	6.77	25	3
PREDICTED: similar to peptidylprolyl isomerase A isoform 1	113421305	PPIA	HTGPGILSMAN	2	17889.6	9.11	10.37	72.2	6.74	25	2
			VSFELFADK	2	17889.6	9.11	15.52	91.8	4.37	25	2
PREDICTED: similar to peptidylprolyl isomerase A isoform 1	113421305	PPIA	HTGPGILSMAN	2	17889.6	9.11	10.37	72.2	6.74	25	2
			VSFELFADK	2	17889.6	9.11	15.52	91.8	4.37	25	2
PREDICTED: similar to peptidylprolyl isomerase A isoform 1	113421305	PPIA	HTGPGILSMAN	2	17889.6	9.11	10.37	72.2	6.74	25	2
			VSFELFADK	2	17889.6	9.11	15.52	91.8	4.37	25	2
PREDICTED: similar to peptidylprolyl isomerase A isoform 1	113421305	PPIA	HTGPGILSMAN	2	17889.6	9.11	10.37	72.2	6.74	25	2
			VSFELFADK	2	17889.6	9.11	15.52	91.8	4.37	25	2
PREDICTED: similar to peptidylprolyl isomerase A isoform 1	113421305	PPIA	HTGPGILSMAN	2	17889.6	9.11	10.37	72.2	6.74	25	2
			VSFELFADK	2	17889.6	9.11	15.52	91.8	4.37	25	2
PREDICTED: similar to peptidylprolyl isomerase A isoform 1	113421305	PPIA	HTGPGILSMAN	2	17889.6	9.11	10.37	72.2	6.74	25	2
			VSFELFADK	2	17889.6	9.11	15.52	91.8	4.37	25	2
PREDICTED: similar to Prostate, ovary, testis expressed protein on chromosome 2 isoform 2	88953571	A26C1A	AVFPSIVGR	2	121283.1	5.83	15.36	86.8	9.79	25	2
			QEYDESGPSIVHR	2	121283.1	5.83	16.47	90.9	4.65	25	3
PREDICTED: similar to Triosephosphate isomerase (TIM) (Triose-phosphate isomerase) isoform 1	113408766	TPI1	IIYGGSVTGATCK	2	22671.2	8.82	16.73	90.4	8.2	25	2
			VVLAYEPVWAIGTGK	2	22671.2	8.82	18.01	91.6	5.97	25	2
PREDICTED: similar to trophoblast glycoprotein	113422497	HNRNPUL1	GGPALLAALDAALAPLAELR	2	60384	8.87	10.35	84.6	4.37	25	2
			LNALAGLDPDELR	2	60384	8.87	14.93	81.2	4.03	25	2
PREDICTED: similar to Ubiquitin-63E CG11624-PA, isoform A	113423966	UBC	TITLEVEPSDTIENVK	2	60384	8.93	20.15	92.4	4	25	2
			TLSDYNIQK	2	60384	8.93	14.8	94.7	5.5	21	2
prion protein preproprotein	4506113	PRNP	PIIHFGSDYEDR	1	27661.3	9.13	16.67	86.7	4.54	25	3
procollagen C-endopeptidase enhancer	4505643	PCOLCE	ECIWTITVPEGQTVSLSFR	9	47946.7	7.41	16.29	86.9	4.53	25	2
			EPGEGLAVTVSLIGAYK	9	47946.7	7.41	13.56	79.9	4.53	25	2
			FDLEPDTYCR	9	47946.7	7.41	11.59	93.8	4.03	15	2
			GFLLWYSGR	9	47946.7	7.41	12.92	77.8	8.75	25	2
			IIAPPDQVIALTFEK	9	47946.7	7.41	13.71	91.1	4.37	21	2
			PAPLVAPGNQVTLR	9	47946.7	7.41	12.46	93.6	10.18	18	2
			SQPPEKTEESPSAPDAPTCPK	9	47946.7	7.41	14.86	72.6	4.41	25	3
			TEESPSAPDAPTCPK	9	47946.7	7.41	18.69	87.6	4.14	25	2
			YDALEVFAGSGTSGQR	9	47946.7	7.41	14.41	85.2	4.37	25	2
proenkephalin	5453876	PENK	DAEEDDSLANSSDLLK	3	30787.2	5.44	19.12	89.3	3.66	25	2
			FAEALPSDEEGESYSK	3	30787.2	5.44	10.68	88.7	3.91	14	2
			VGRPEWWMDYQK	3	30787.2	5.44	12.76	84.3	6.04	25	3
progesterone receptor membrane component 1	5729875	PGRMC1	GDQPAASGDSDDDEPPPLPR	2	21671.3	4.56	13.79	91.2	3.61	16	2
			GLATFCLDK	2	21671.3	4.56	11.07	73.7	5.83	25	2
progesterone receptor membrane component 2	5453916	PGRMC2	GLATFCLDK	2	23818.6	4.76	11.07	73.7	5.83	25	2
			GLGAGAGAGEESPATSLPR	2	23818.6	4.76	15.03	76.9	4.53	25	2
prohibitin	4505773	PHB	AAELIANSLATAGDGLIELR	3	44615	5.57	13.94	80	4.14	25	3
			IFTSIGEDYDER	3	44615	5.57	15.11	83.7	3.91	25	2
			VLPSITTEILK	3	44615	5.57	15.42	88.3	5.97	25	2
prolyl 4-hydroxylase, beta subunit precursor	20070125	P4HB	DAPEEEDHVLVLR	3	57116.6	4.76	16.87	92	4.17	24	3
			QFLQAAEAIDDIPFGITSNSDVFSK	3	57116.6	4.76	20.64	84.4	3.84	25	3
			VDATEESDLAQQYGVR	3	57116.6	4.76	16.83	87.2	3.91	25	2
proprotein convertase subtilisin/kexin type 1 inhibitor precursor	7019519	PCSK1N	GEAAGAVQELAR	3	27372.5	6.22	18.58	90.4	4.53	25	2
			ILAGSADSEGVAAPR	3	27372.5	6.22	18.18	86.1	4.37	25	2
			NSDPALGLDDDPDAPAAQLAR	3	27372.5	6.22	15.54	79.1	3.66	25	3
proprotein convertase subtilisin/kexin type 1 preproprotein	20336242	PCSK1	ALVDLADPR	12	84152	5.66	17.25	88.6	4.21	25	2
			ANGEVIIEIPTR	12	84152	5.66	17.79	90.7	4.53	25	2
			CSSTLATSYSSGDYTDQR	12	84152	5.66	14.26	85.2	4.21	25	2
			DMQHLVVWTSEYDPLANNPGWK	12	84152	5.66	15	75.8	4.54	25	3
			DSALNLFNDPMWNQQWYLQDTR	12	84152	5.66	19.51	86.5	3.93	25	3
			FGFGLLNAK	12	84152	5.66	18.09	92.1	8.75	24	2
			LDLHVIPVWQK	12	84152	5.66	18.4	76.2	6.74	25	3
			LLQALVDILNEEN	12	84152	5.66	16.99	93.3	3.57	25	2
			LNIPYENFYEALEK	12	84152	5.66	20.09	94.9	4.25	25	2
			MLDGIVTDAIEASSIGFNPGHVDIYSASWGPNDDGK	12	84152	5.66	18.26	73	3.9	25	3
			NWDFMSVHTWGENPIGTWTLR	12	84152	5.66	17.64	86.2	5.32	25	3
			RDELEEGAPSQAMLR	12	84152	5.66	26.33	96.2	4.41	25	3
proprotein convertase subtilisin/kexin type 2	20336244	PCSK2	CAGEVSAAANNNICGVGVAYNSK	14	70565.6	6.04	19.91	91	5.99	25	3
			DINEIDINMNDPLFTK	14	70565.6	6.04	23.05	93.8	3.84	25	2
			EELEEELDEAVER	14	70565.6	6.04	21.14	96.7	3.71	25	2
			EWTLMLHGTQSAPYIDQVVR	14	70565.6	6.04	17.81	90.4	5.32	25	3
			FHCVGGSVQDPEK	14	70565.6	6.04	14	74.5	5.32	25	3
			FHCVGGSVQDPEKIPSTGK	14	70565.6	6.04	12.44	75.1	6.74	25	3
			GTWTLELGFVGSAPQK	14	70565.6	6.04	17.01	87.1	6	25	2
			KEELEEELDEAVER	14	70565.6	6.04	17.55	91.8	3.99	25	2
			LAMSKKEELEEELDEAVER	14	70565.6	6.04	14.81	84.8	4.22	25	3
			LVLTLTTDACEGK	14	70565.6	6.04	11.76	85.4	4.37	18	2
			QVAAEHGFGVR	14	70565.6	6.04	15.97	86.3	6.75	25	2
			RKKRGYRDINEIDINMNDPLFTK	14	70565.6	6.04	12.97	75	9.52	24	3
			VGFDKWPF	14	70565.6	6.04	14.43	87.9	5.81	25	2
			YTDDWFNSHGTR	14	70565.6	6.04	12.49	76.8	5.21	25	3
prosaposin isoform b preproprotein	110224476	PSAP	EIVDSYLPVILDIIK	3	58484.4	5.03	14.45	79.2	4.03	25	2
			LVGYLDR	3	58484.4	5.03	11.01	94.5	5.83	17	2
			QEILAALEK	3	58484.4	5.03	13.44	78.8	4.53	25	2
prostatic binding protein	4505621	PEBP1	APVAGTCYQAEWDDYVPK	3	21056.9	7.01	14.65	80.9	4.03	25	2
			GNDISSGTVLSDYVGSGPPK	3	21056.9	7.01	21.14	94.7	4.21	25	2
			LYTLVLTDPDAPSR	3	21056.9	7.01	21.09	93.4	4.21	25	2
protein disulfide isomerase-associated 3 precursor	21361657	PDIA3	DASIVGFFDDSFSEAHSEFLK	10	56782.7	5.99	17.36	89.5	4.1	25	3
			ELSDFISYLQR	10	56782.7	5.99	14.74	84.5	4.37	25	2
			FLQDYFDGNLKR	10	56782.7	5.99	12.02	88	5.96	24	3
			FVMQEEFSR	10	56782.7	5.99	11.09	74.7	4.53	25	2
			GFPTIYFSPANK	10	56782.7	5.99	13.64	84.5	8.59	25	2
			LSKDPNIVIAK	10	56782.7	5.99	15.19	82.1	8.59	25	3
			MDATANDVPSPYEVR	10	56782.7	5.99	11.66	72.4	4.03	25	2
			SDVLELTDDNFESR	10	56782.7	5.99	12.93	82	3.77	24	2
			SEPIPESNDGPVK	10	56782.7	5.99	19.32	96	4.14	23	2
			TFSHELSDFGLESTAGEIPVVAIR	10	56782.7	5.99	16.7	84.4	4.4	25	3
protein disulfide isomerase-associated 6	5031973	PDIA6	ALDLFSDNAPPPELLEIINEDIAK	3	48121.6	4.95	17.53	84.3	3.71	25	3
			GSFSEQGINEFLR	3	48121.6	4.95	10.49	72	4.53	25	2
			TGEAIVDAALSALR	3	48121.6	4.95	13.68	100	4.37	13	2
protein kinase C substrate 80K-H isoform 1	48255889	PRKCSH	AQQEQELAADAFK	3	33232.9	4.33	14.73	77.2	4.14	25	2
			ESLQQMAEVTR	3	33232.9	4.33	12.36	88.4	4.53	25	2
			SLEDQVEMLR	3	33232.9	4.33	13.75	82.7	4.14	25	2
protein kinase, interferon-inducible double stranded RNA dependent activator	4505581	PRKRA	LPEYTLSQEGGPAHK	2	34404.4	8.68	14.99	75.3	5.4	25	3
			SLLSIPNTDYIQLLSEIAK	2	34404.4	8.68	17.36	75.2	4.37	25	3
protein phosphatase 1 (formerly 2C)-like	63003905	PPM1L	LGGLDVLEAEFSK	2	41053.2	5.58	13.89	78.9	4.14	24	2
			SIVLQSFYR	2	41053.2	5.58	13.22	85.9	8.46	19	2
protein tyrosine phosphatase type IVA, member 1	4506283	PTP4A1	FLITHNPTNATLNK	2	19815.2	9.17	15.31	86.6	8.76	25	3
			KYRPKMRLRFKDS	2	19815.2	9.17	12.7	85.5	11.07	25	2
protein tyrosine phosphatase, receptor type, N polypeptide 2 isoform 1 precursor	11386149	COX15	EQFEFALTAVAEEVNAILK	7	111282	5.55	18.76	87.4	4.09	25	3
			GLSGLELDGMAELMAGLMQGVDHGVAR	7	111282	5.55	13.6	82.7	4.31	25	3
			ISSVSSQFSDGPIPSPSAR	7	111282	5.55	14.74	81.9	5.84	25	2
			LLQVPSSAFADVEVLGPAVTFK	7	111282	5.55	19.81	86.9	4.37	25	2
			LSGLGGDPGADATAAYQELCR	7	111282	5.55	18.47	93.1	4.03	25	2
			TLGQLQPDELSPK	7	111282	5.55	13.49	82.4	4.37	25	2
			YEVSPVALQR	7	111282	5.55	14.16	79.7	6	25	2
protein tyrosine phosphatase, receptor type, N polypeptide 2 isoform 3 precursor	19743914	COX15	EQFEFALTAVAEEVNAILK	6	53005.6	5.61	18.76	87.4	4.09	25	3
			GLSGLELDGMAELMAGLMQGVDHGVAR	6	53005.6	5.61	13.6	82.7	4.31	25	3
			ISSVSSQFSDGPIPSPSAR	6	53005.6	5.61	14.74	81.9	5.84	25	2
			LSGLGGDPGADATAAYQELCR	6	53005.6	5.61	18.47	93.1	4.03	25	2
			TLGQLQPDELSPK	6	53005.6	5.61	13.49	82.4	4.37	25	2
			YEVSPVALQR	6	53005.6	5.61	14.16	79.7	6	25	2
protein tyrosine phosphatase, receptor type, N precursor	4506321	PTPRN	EEAAAVLPQTAHSTSPMR	5	105848.1	6.66	12.3	94.3	5.4	22	3
			GEKPASPAVQPDAALQR	5	105848.1	6.66	20.04	93.3	6.07	25	3
			PLLQVTSPVLQR	5	105848.1	6.66	12.35	74.6	10.18	25	2
			SKDQFEFALTAVAEEVNAILK	5	105848.1	6.66	13.1	76.6	4.41	25	3
			VSEGSPGMVSVGPLPK	5	105848.1	6.66	14.06	73.7	5.97	25	2

pyruvate dehydrogenase (lipoamide) beta	156564403	PDHB	DNNPVVVLENELMYGVPFEFPPEAQSK	4	39233.6	6.21	15.55	78.9	3.91	25	3
			IMEGPAFNFLDAPAVR	4	39233.6	6.21	15.31	70.1	4.37	25	2
			VFLLGEEVAQYDGAYK	4	39233.6	6.21	15.16	79.4	4.14	25	2
			VTGADVPMPYAK	4	39233.6	6.21	12.31	71.2	5.8	25	2
quiescin Q6 sulfhydryl oxidase 1 isoform a	13325075	QSOX1	FGVTDFPSCYLLFR	1	82578.2	9.13	13.07	74.2	5.83	25	2
quinoid dihydropteridine reductase	4506359	QDPR	AALDGTPGMIGYGMAK	3	25803.7	6.9	12.61	72.7	5.88	25	2
			NRPSSGSLIQVVTTEGR	3	25803.7	6.9	17.73	87.4	9.6	25	3
			NSGMPPGAAAIAVLPVTLDTPMNR	3	25803.7	6.9	17.36	86.6	5.84	25	3
RAB2B protein	21361884	RAB2B	FQPVHDLTIGVEFGAR	3	24214.5	7.69	14.46	79	5.32	25	3
			IGPQQSISTSVGPSASQR	3	24214.5	7.69	11.72	81.5	9.75	25	2
			LQIWDTAGQESFR	3	24214.5	7.69	12	84.6	4.37	19	2
RAB39	31543537	RAB34	LQLWDTAGQER	1	29058.7	8.07	13.81	89.3	4.37	25	2
rabphilin 3A homolog	45267837	RPH3A	FPDQKPEVAPSDPGTTAPPR	7	76386.5	8.75	16.64	81.3	4.56	25	3
			LQAGWSVHPGGQPDR	7	76386.5	8.75	15.67	89.1	6.74	25	3
			MSHPSGPYSQASAAAPQPAAAR	7	76386.5	8.75	18.81	91.8	8.52	25	3
			NPIWNETLVYHGITDEDMQR	7	76386.5	8.75	14.29	74.7	4.31	25	3
			PYVKLHLLPGASKS	7	76386.5	8.75	12.22	87.9	9.72	17	3
			SGAWFFK	7	76386.5	8.75	12.43	90.1	8.47	17	2
			SNDYIGGCQLGISAK	7	76386.5	8.75	16.43	88.9	5.55	25	2
ras related v-ral simian leukemia viral oncogene homolog A	33946329	RALA	AEQWNVNYVETSAK	2	23566.9	6.66	12.64	80.3	4.53	25	2
			VKEDENVPFLLVGNK	2	23566.9	6.66	18.03	78.5	4.68	25	3
ras-related C3 botulinum toxin substrate 1 isoform Rac1b	9845509	RAC1	HHCPNTPIILVGTK	4	23467.5	8.87	15.05	78.4	8.24	25	3
			TVFDEAIR	4	23467.5	8.87	11.93	85.5	4.37	25	2
			WYPEVR	4	23467.5	8.87	10.22	81.7	6	17	2
			YLECSALTQR	4	23467.5	8.87	14.73	82.9	5.99	25	2
ras-related GTP-binding protein 4b	82659107	RAB4B	LQIWDTAGQER	1	48262.2	5.8	13.81	89.3	4.37	25	2
ras-related GTP-binding protein RAB10	33695095	RAB10	AFLTLAEDILR	3	22469	8.8	18.25	94.1	4.37	25	2
			LLLIGDSGVGK	3	22469	8.8	12.03	89.5	5.84	18	2
			LQIWDTAGQER	3	22469	8.8	13.81	89.3	4.37	25	2
Ras-related protein Rab-11A	4758984	RAB11A	AQIWDTAGQER	4	24393.6	6.12	24.68	99	4.37	25	2
			DHADSNIVIMLVGNK	4	24393.6	6.12	15.52	80.4	5.21	25	3
			GAVGALLVYDIAK	4	24393.6	6.12	11.1	73.6	5.83	25	2
			VVLIGDSGVGK	4	24393.6	6.12	19.35	95.6	5.81	25	2
RAS-related protein RAB-22A	10190714	RAB22A	FVEDSFDPNINPTIGASFMTK	2	21855.2	8.32	13.93	75.8	4.03	25	3
			RIPSTDANLPSGGK	2	21855.2	8.32	18.36	87.9	8.75	25	3
Ras-related protein Rab-27A	19923264	RAB27A	FLALGDSGVGK	4	23658.8	5.09	17.33	93.2	5.84	25	2
			IHLQLWDTAGQER	4	23658.8	5.09	12.37	76.3	5.32	25	3
			NWISQLQMHAYCENPDIVLCGNK	4	23658.8	5.09	16.24	78.7	5.32	25	3
			SWIPEGVVR	4	23658.8	5.09	14.33	92.2	5.72	23	2
Ras-related protein Rab-33A	4758996	RAB33A	VQVWDTAGQER	1	26592.8	8.07	13.12	88.7	4.37	22	2
retbindin isoform 2	13899247	RTBDN	SALGHALPVAAPGAR	1	35022.1	7.95	17.35	83	9.49	25	3
retinol binding protein 1, cellular	4506451	RBP1	ALDVNVALR	3	15850.3	4.99	11.6	91	5.88	15	2
			EFEEDLTGIDDR	3	15850.3	4.99	13.72	83.4	3.71	25	2
			MLVNENFEEYLR	3	15850.3	4.99	10.01	71.6	4.25	25	2
Rho GTPase activating protein 1	4757766	ARHGAP1	EPIPIVLRETVAYLQAHALTT	2	50436	5.85	12.11	84.1	5.4	25	2
			YNMGLPVDFDQYNELHLPAVILK	2	50436	5.85	14.53	82.9	4.54	25	3
scaffold attachment factor B	21264343	SAFB	LFSKYGKVVGAKVVTNARS	2	102642.1	5.32	13.37	85.8	10.46	25	3
			RGGMSGRGSFAPGGASRGH	2	102642.1	5.32	12.5	84.4	12.3	25	3
scavenger receptor class B, member 2	5031631	SCARB2	TLNIPVLTVIEWSQVHFLR	1	54290.4	5	17.11	86.9	6.41	25	3
Sec61 beta subunit	5803165	SEC61B	FYTEDSPGLK	1	9974.5	11.57	13.77	88.5	4.37	25	2
secreted modular calcium-binding protein 1 isoform 1	78190498	SMOC1	FSEPDPSHTLEER	3	48262.2	8.58	18.31	92	4.4	25	3
			FTDYCDLNK	3	48262.2	8.58	12.84	77.2	4.21	25	2
			TPVCSGSVTDKPLSQGNSGR	3	48262.2	8.58	13.78	83.6	7.89	20	3
secretogranin II precursor	68160947	SCG2	AGTEALPDGLSVEDILNLLGMESAANQK	11	70941.1	4.67	15.04	76	3.83	25	3
			ALEYIENLR	11	70941.1	4.67	20.13	90.2	4.53	25	2
			ANNIAYEDVVGGEDWNPVEEK	11	70941.1	4.67	19.77	88.5	3.77	25	3
			DSLSEEDWMR	11	70941.1	4.67	12.34	73	3.91	25	2
			ELDLPVDLDDISEADLDHPDLFQNR	11	70941.1	4.67	13.16	74.2	3.69	23	3
			NLQIPPEDLIEMLK	11	70941.1	4.67	15.22	92	4.14	25	2
			QMAYENLNDKDQELGEYLAR	11	70941.1	4.67	13.29	71	4.18	25	3
			SGQLGIQEEDLR	11	70941.1	4.67	17.72	89.9	4.14	25	2
			SGQLGIQEEDLRK	11	70941.1	4.67	16.17	87.5	4.68	25	3
			TNEIVEEQYTPQSLATLESVFQELGK	11	70941.1	4.67	18.5	88.5	3.98	25	3
			YPEIINSNQVK	11	70941.1	4.67	12.62	75.3	6	25	2
secretogranin III	19557645	SCG3	GILDKEEAEAIKR	4	53005.6	4.94	18.23	91.6	4.87	25	3
			LLNLGLITESQAHTLEDEVAEVLQK	4	53005.6	4.94	18.47	90.5	4.25	25	3
			TYSEDNFEELQYFPNFYALLK	4	53005.6	4.94	12.66	83.4	4	25	3
			VTPMAAIQDGLAK	4	53005.6	4.94	12.05	74.7	5.81	25	2
secretory carrier membrane protein 3 isoform 1	16445419	SCAMP3	TAAANAAAGAAENAFR	1	12537.7	7.55	15.98	85.2	5.66	25	2
selenoprotein S	33285002	SELS	AAAAVEPDVVVK	2	670140.2	9.72	12.32	71.3	4.37	25	2
			KPQEEDSPGPSTSSVLK	2	670140.2	9.72	15.66	84.7	4.68	25	3
septin 2	4758158	1-Sep	ASIPFSVVGSNQLIEAK	1	41487.7	6.15	15.55	83.1	6.05	25	2
serine (or cysteine) proteinase inhibitor, clade A (alpha-1 antiproteinase, antitrypsin), member 1	50363217	SERPINA1	VFSNGADLSGVTEEAPLK	1	32996.2	5.37	12.61	89.7	4.14	17	2
serine (or cysteine) proteinase inhibitor, clade B (ovalbumin), member 12	17998551	SERPINB12	LVLVNAVYFK	1	46276.7	5.36	13.27	88.4	8.59	25	2
serine (or cysteine) proteinase inhibitor, clade H, member 1	32454741	SERPINH1	DQAVENILVSPVVVASSLGLVSLGGK	1	46440.8	8.75	17.67	82.6	4.37	25	3
sideroflexin 1	23618867	SFXN1	ILMAAPGMAIPPFIMNTLEK	3	35619.6	9.22	11.23	72.6	6	24	2
			NILLTNEQLESAR	3	35619.6	9.22	16.07	83.2	4.53	25	2
			SGDAPLTVNELGTAYVSATTGAVATALGLNALTK	3	35619.6	9.22	19.37	83.5	4.37	25	3
signal sequence receptor, delta	5454090	SSR4	NNEDISIIPPLFTVSVDHR	1	27774.2	5.76	21.21	89.3	4.54	25	3
skeletal muscle ryanodine receptor isoform 1	113204615	RYR1	GAAEAPARLSPAIPLEALR	3	565178.8	5.18	15.29	77.6	6.14	25	3
			PARAENEKDATTEKN	3	565178.8	5.18	13.02	86.6	4.87	25	3
			SNGYKPAPLDLSHVRLTPAQTTL	3	565178.8	5.18	12.9	78.8	8.34	25	3
slit homolog 3	11321571	SLIT3	AIPAGAFTQYK	9	167686.4	7.99	17.22	92.1	8.63	25	2
			DVTELYLEGNHLTAVPR	9	167686.4	7.99	12.97	80.8	4.65	20	3
			EGAFDGAASVQELMLTGNQLETVHGR	9	167686.4	7.99	15.62	82.8	4.4	25	3
			GLFDGLVSLQLLLLNANK	9	167686.4	7.99	16.33	84.1	5.84	25	2
			IPSHLPEYVTDLR	9	167686.4	7.99	15.83	85.7	5.32	25	3
			NQISDIAPDAFQGLK	9	167686.4	7.99	14.39	87.2	4.21	22	3
			QPAVGINSPLYLGGIPTSTGLSALR	9	167686.4	7.99	17.17	81.9	8.75	25	3
			VNTFQDLQNLNLLSLYDNK	9	167686.4	7.99	12.71	77.6	4.21	25	3
			WLADYLQDNPIETSGAR	9	167686.4	7.99	21.27	94.1	4.03	25	2
smooth muscle and non-muscle myosin alkali light chain 6B	4505303	MYL6B	ALGQNPTNAEVLK	1	22764.1	5.56	15.82	87.6	6.05	25	2
son of sevenless homolog 2	154689780	SOS2	AHPPAPPVPPRQN	2	187022	6.39	12.96	77.4	9.8	24	3
			EPTDADKLAIEKGEQP	2	187022	6.39	13.84	70.9	4.18	25	3
spectrin repeat containing, nuclear envelope 2 isoform 5	118918407	SYNE2	EQSKALVSNLISTK	2	613393.1	5.25	12.62	72.5	8.69	25	3
			QRMADDVVAIESEVKS	2	613393.1	5.25	10.86	74.8	4.32	25	2
spectrin, beta, non-erythrocytic 4 isoform sigma1	115430237	SPTBN4	GGSAPAPPPPPTHTVQHE	2	31629.9	5.72	15	80.4	6.02	25	3
			ILQGELGGRRALLQQALRCGEELVAAG	2	31629.9	5.72	13.01	82.9	6.22	25	3
splicing coactivator subunit SRm300	118572613	SRRM2	RGEFSASPMLKSGMSP	2	613393.1	12.05	12.23	75.6	8.75	25	3
			RPESSLVFKDTLRTPPRERSGAGSS	2	613393.1	12.05	13.63	75.3	10.67	25	3
stathmin 1	5031851	STMN1	DLSLEEIQK	1	17302.6	5.76	14.01	73.6	4.14	25	2
stratifin	5454052	SFN	DSTLIMQLLR	1	27774.2	4.68	15.68	93.9	5.84	25	2
succinate dehydrogenase complex, subunit B, iron sulfur (Ip)	115387094	SDHB	DLVPDLSNFYAQYK	2	31629.9	9.03	20.11	94.4	4.21	25	2
			YLGPAVLMQAYR	2	31629.9	9.03	15.27	85.7	8.59	25	2
succinate-CoA ligase, GDP-forming, alpha subunit	109452591	SUCLG1	GGQTHLGLPVFNTVK	2	36249.9	9.01	16.37	88.2	8.76	25	3
			LIGPNCPGVINPGECK	2	36249.9	9.01	22.5	96	5.99	25	2
superiorcervical ganglia, neural specific 10	34850061	STMN2	DLSLEEIQK	4	49875.2	8.4	14.01	73.6	4.14	25	2
			EANLAAIIER	4	49875.2	8.4	10.54	85.1	4.53	25	2
			ELILKPPSPISEAPRTLASPKKKDL	4	49875.2	8.4	12.01	79.7	9.53	25	3
			NINIYTYDDMEVK	4	49875.2	8.4	11.82	76.1	4.03	22	2
synaptojanin 2 binding protein	8922964	SYNJ2BP	GPSGLGFNIVGGTDQQYVSNDSGIYVSR	2	15900.1	5.86	22.87	98.2	4.21	25	3
			VDYLVTEEEINLTR	2	15900.1	5.86	18.38	89	4	25	2
synaptophysin	27764867	SYP	LHQVYFDAPTCR	2	33845.4	4.65	18.29	81.5	6.73	25	3
			MATDPENIIK	2	33845.4	4.65	15.56	87	4.37	25	2
synaptosomal-associated protein 25 isoform SNAP25A	18765733	SNAP25	ENEMDENLEQVSGIIGNLR	1	41920.5	4.74	16.88	84.3	3.91	25	3
synaptotagmin I	5032139	SYT1	HWSDMLANPR	8	47573.4	8.26	11.36	83.1	6.74	23	2
			KMDVGGLSDPYVK	8	47573.4	8.26	10.83	71.3	5.96	21	2
			LGDICFSLR	8	47573.4	8.26	15.12	91.4	5.83	25	2
			LTVVILEAK	8	47573.4	8.26	13.89	90.5	6	23	2
			TLNPVFNEQFTFK	8	47573.4	8.26	16.2	84.7	5.66	25	2
			TLVMAVYDFDR	8	47573.4	8.26	10.79	71.9	4.21	25	2
			VFVGYNSTGAELR	8	47573.4	8.26	19.31	91.8	5.97	25	2
			VQVVVTVLDYDKIGK	8	47573.4	8.26	12.84	80.3	5.93	24	3
synaptotagmin II	31543670	SYT2	GGQDDDDAETGLTEGEGEGEEEKEPENLGK	9	46872.5	8.18	19.46	88.7	3.66	25	3
			HWSDMLANPR	9	46872.5	8.18	11.36	83.1	6.74	23	2
			IFVGSNATGTELR	9	46872.5	8.18	16.94	92.8	6	25	2
			KMDVGGLSDPYVK	9	46872.5	8.18	10.83	71.3	5.96	21	2
			TLNPAFNETFTFK	9	46872.5	8.18	17.38	95.1	5.66	25	2
			TLNPYFNESFSFEIPFEQIQK	9	46872.5	8.18	12.5	70.2	4.25	25	3
			TLVMAIYDFDR	9	46872.5	8.18	22.19	96.7	4.21	25	2
			VPMNTVDLGQPIEEWR	9	46872.5	8.18	22.73	94.2	4.14	25	3
			VQVVVTVLDYDKLGK	9	46872.5	8.18	12.84	80.3	5.93	24	3
synaptotagmin V	92859638	SYT5	KMDVGGLSDPYVK	2	42900.7	9.26	10.83	71.3	5.96	21	2
			LGDICFSLR	2	42900.7	9.26	15.12	91.4	5.83	25	2
syntaxin 1A (brain)	4759182	STX1A	IEYNVEHAVDYVER	2	33023.6	5.14	12.44	81.4	4.4	25	3
			TTTSEELEDMLESGNPAIFASGIIMDSSISK	2	33023.6	5.14	19.55	91.1	3.77	25	3
thioredoxin peroxidase	5453549	PRDX4	GLFIIDDK	2	30540.1	5.86	10.1	82.3	4.21	20	2
			IPLLSDLTHQISK	2	30540.1	5.86	16.03	82.6	6.74	23	3
thyroglobulin	55770862	TG	ARLALQFTTNPKRLQQNLFGGKFL	2	357528.9	5.4	12.17	72.7	12.02	25	3
			LGVPYAAPPLAERRFQAPEPL	2	357528.9	5.4	12.06	83.8	6.14	17	3
tissue inhibitor of metalloproteinase 1 precursor	4507509	TIMP1	EPGLCTWQSLR	4	23171	8.46	10.12	70.5	6.09	25	2
			GFQALGDAADIR	4	23171	8.46	18.26	88.1	4.21	25	2
			LQDGLLHITTCSFVAPWNSLSLAQR	4	23171	8.46	16.27	75.2	6.73	25	3
			LQSGTHCLWTDQLLQGSEK	4	23171	8.46	17.24	88.1	5.32	25	3
tissue inhibitor of metalloproteinase 3 precursor	4507513	TIMP3	NECLWTDMLSNFGYPGYQSK	2	24145.2	9	12.61	73.9	4.37	25	3
			YQYLLTGR	2	24145.2	9	11.28	85.1	8.59	25	2
titin isoform N2-A	110349719	TTN	PSFIRKLKDVNAILGAS	4	3006843.3	6.16	13.37	70.5	10.01	25	3
			RAGSNLKVDIPISGKPL	4	3006843.3	6.16	12.75	83.1	9.99	23	3
			SDPRQLSVPVIAKDLVIPPAFKLLFNTF	4	3006843.3	6.16	12.69	76.7	8.31	25	3
			VANPDSKGEWLRDGKHL	4	3006843.3	6.16	12.71	78.2	6.73	25	3
titin isoform N2-B	110349715	TTN	AQSALILSQSLAEGHVESLQ	4	3006843.3	6.35	11.86	73.3	4.51	25	2
			RAGSNLKVDIPISGKPL	4	3006843.3	6.35	12.75	83.1	9.99	23	3
			SDPRQLSVPVIAKDLVIPPAFKLLFNTF	4	3006843.3	6.35	12.69	76.7	8.31	25	3
			VANPDSKGEWLRDGKHL	4	3006843.3	6.35	12.71	78.2	6.73	25	3
titin isoform novex-1	110349713	TTN	AQSALILSQSLAEGHVESLQ	4	3006843.3	6.36	11.86	73.3	4.51	25	2
			RAGSNLKVDIPISGKPL	4	3006843.3	6.36	12.75	83.1	9.99	23	3
			SDPRQLSVPVIAKDLVIPPAFKLLFNTF	4	3006843.3	6.36	12.69	76.7	8.31	25	3
			VANPDSKGEWLRDGKHL	4	3006843.3	6.36	12.71	78.2	6.73	25	3
titin isoform novex-2	110349717	TTN	AQSALILSQSLAEGHVESLQ	4	3006843.3	6.31	11.86	73.3	4.51	25	2
			RAGSNLKVDIPISGKPL	4	3006843.3	6.31	12.75	83.1	9.99	23	3
			SDPRQLSVPVIAKDLVIPPAFKLLFNTF	4	3006843.3	6.31	12.69	76.7	8.31	25	3
			VANPDSKGEWLRDGKHL	4	3006843.3	6.31	12.71	78.2	6.73	25	3
toll interacting protein	21361619	TOLLIP	AIQDMFPNMDQEVIR	5	30282	5.68	18.98	88.9	4.03	25	2
			DAAINSLLQMGEEP	5	30282	5.68	18.72	94.5	3.57	25	2
			IAWTHITIPESLR	5	30282	5.68	23.04	95.5	6.75	25	3
			LNITVVQAK	5	30282	5.68	10.85	79.7	8.75	23	2
			VIHCTVPPGVDSFYLEIFDER	5	30282	5.68	20.72	91.7	4.31	25	3
transcobalamin II precursor	21071010	TCN2	LSLEHLNPSIYVGLR	1	47535.3	6.52	17.09	88.8	6.75	25	3
transgelin 2	4507357	TAGLN2	GASQAGMTGYGMPR	2	22391.6	8.41	15.6	83.3	8.75	25	2

			TTDIFQTVDLWEGK	2	22391.6	8.41	18.34	90.4	4.03	25	2
transgelin 3	56549135	TAGLN3	GASQAGMTGYGMPR	5	22472.8	6.84	15.6	83.3	8.75	25	2
			QGQNVIGLQMGSNK	5	22472.8	6.84	14.05	82.9	8.75	25	2
			QMEQISQFLK	5	22472.8	6.84	11.83	83.4	6	25	2
			TLMALGSVAVTK	5	22472.8	6.84	13.26	73	8.41	25	2
			TTDIFQTVDLWEGK	5	22472.8	6.84	18.34	90.4	4.03	25	2
transmembrane protein 109	13129092	TMEM109	EAPVDVLTQIGR	1	29736.7	10.48	18.05	86.5	4.37	25	2
transmembrane protein 4	7657176	CNPY2	ALVDELEWEIAQVDPK	3	20652.3	4.81	10.91	82.4	3.83	25	2
			IDSDISGTLK	3	20652.3	4.81	12.62	82.7	4.21	25	2
			INPDGSQSVVEVPYAR	3	20652.3	4.81	17.56	85.4	4.37	25	2
triosephosphate isomerase 1	4507645	TPI1	HVFGESDELIGQK	3	26669.6	6.45	13.33	74.2	4.65	25	3
			IIYGGSVTGATCK	3	26669.6	6.45	16.73	90.4	8.2	25	2
			VVLAYEPVWAIGTGK	3	26669.6	6.45	18.01	91.6	5.97	25	2
tripartite motif-containing 67	134288906	TRIM67	HPDPAFGVARASVVKDMMLGK	2	83823.7	7.09	12.05	77.1	8.6	25	3
			SPNGVRVLPMVPAPPGSSAAAAR	2	83823.7	7.09	12.77	76.4	12	20	3
tripeptidyl-peptidase I preproprotein	5729770	TPP1	ADPEEELSLTFALR	6	61248.2	6.01	18.37	87.7	4	25	2
			AYPDVAALSDGYWVVSNR	6	61248.2	6.01	13.2	74.2	4.21	24	3
			ILSGRPPLGFLNPR	6	61248.2	6.01	13.19	84.2	12	25	3
			LITNEIVDYISGGGFSNVFPR	6	61248.2	6.01	13.47	70.4	4.37	25	3
			LSELVQAVSDPSSPQYGK	6	61248.2	6.01	19	91.2	4.37	25	2
			VPIPWVSGTSASTPVFGGILSLINEHR	6	61248.2	6.01	15.79	74.7	6.72	25	3
tropomyosin 1 alpha chain isoform 3	63252896	TPM1	IQLVEEELDR	1	32736.8	4.71	14.27	91.8	4	25	2
tropomyosin 1 alpha chain isoform 5	27597085	TPM1	IQLVEEELDR	1	15819.3	4.69	14.27	91.8	4	25	2
tropomyosin 1 alpha chain isoform 6	63252904	TPM1	IQLVEEELDR	1	32736.8	4.74	14.27	91.8	4	25	2
tropomyosin 1 alpha chain isoform 7	63252906	TPM1	IQLVEEELDR	1	32736.8	4.71	14.27	91.8	4	25	2
tropomyosin 2 (beta) isoform 1	42476296	TPM2	IQLVEEELDR	2	31566.7	4.66	14.27	91.8	4	25	2
			MELQEMQLK	2	31566.7	4.66	11.84	72.3	4.53	25	2
tropomyosin 2 (beta) isoform 2	47519616	TPM2	IQLVEEELDR	2	32990	4.63	14.27	91.8	4	25	2
			MELQEMQLK	2	32990	4.63	11.84	72.3	4.53	25	2
tropomyosin 3 isoform 1	114155140	TPM3	IQLVEEELDR	1	32950.1	4.68	14.27	91.8	4	25	2
tropomyosin 3 isoform 2	24119203	TPM3	IQLVEEELDR	3	29032.8	4.75	14.27	91.8	4	25	2
			IQVLQQQADDAEER	3	29032.8	4.75	11.74	77.4	3.91	25	2
			KIQVLQQQADDAEER	3	29032.8	4.75	18.09	87.3	4.32	25	3
tropomyosin 3 isoform 5	114155148	TPM3	IQLVEEELDR	3	32950.1	4.76	14.27	91.8	4	25	2
			IQVLQQQADDAEER	3	32950.1	4.76	11.74	77.4	3.91	25	2
			KIQVLQQQADDAEER	3	32950.1	4.76	18.09	87.3	4.32	25	3
tropomyosin 4	4507651	TPM4	IQLVEEELDR	2	28522	4.67	14.27	91.8	4	25	2
			MEIQEMQLK	2	28522	4.67	11.84	72.3	4.53	25	2
tubulin, alpha 1a	17986283	TUBA1A	AYHEQLSVAEITNACFEPANQMVK	6	50135.9	4.94	15.44	85.5	4.75	25	3
			DVNAAIATIK	6	50135.9	4.94	13.44	70.1	5.84	25	2
			FDGALNVDLTEFQTNLVPYPR	6	50135.9	4.94	18.9	90.3	4.03	25	3
			NLDIERPTYTNLNR	6	50135.9	4.94	14.63	87	6.07	25	3
			TIGGGDDSFNTFFSETGAGK	6	50135.9	4.94	12.02	82.3	4.03	25	2
			VGINYQPPTVVPGGDLAK	6	50135.9	4.94	11.5	82.1	5.8	25	2
tubulin, beta, 4	50592996	MC1R TUBB3	GHYTEGAELVDSVLDVVR	6	50433	4.83	16	85.2	4.31	25	3
			IMNTFSVVPSPK	6	50433	4.83	12.78	70.6	8.75	25	2
			ISEQFTAMFR	6	50433	4.83	11.3	77.2	6	25	2
			LAVNMVPFPR	6	50433	4.83	15.88	89.9	9.75	25	2
			MSSTFIGNSTAIQELFK	6	50433	4.83	12.99	78.6	5.75	25	2
			NSSYFVEWIPNNVK	6	50433	4.83	16.93	88.6	6	25	2
tumor rejection antigen (gp96) 1	4507677	HSP90B1	FQSSHHPTDITSLDQYVER	5	92469.3	4.76	12.15	71.4	5.21	25	3
			GVVDSDDLPLNVSR	5	92469.3	4.76	12.9	78.7	3.93	25	2
			LISLTDENALSGNEELTVK	5	92469.3	4.76	12.55	76.1	4	25	2
			LSLNIDPDAK	5	92469.3	4.76	11.8	78.2	4.21	21	2
			SILFVPTSAPR	5	92469.3	4.76	12.92	80.3	9.47	25	2
tyrosine 3-monooxygenase/tryptophan 5-monooxygenase activation protein, beta polypeptide	4507949	YWHAB	DSTLIMQLLR	3	28082.5	4.76	15.68	93.9	5.84	25	2
			TAFDEAIAELDTLNEESYKDSTLIMQLLR	3	28082.5	4.76	15.31	77	3.95	25	3
			YLIPNATQPESK	3	28082.5	4.76	14.75	77.7	6	25	2
tyrosine 3-monooxygenase/tryptophan 5-monooxygenase activation protein, gamma polypeptide	21464101	YWHAG	DSTLIMQLLR	2	28302.7	4.8	15.68	93.9	5.84	25	2
			NVTELNEPLSNEER	2	28302.7	4.8	15.18	85.6	4.09	25	2
tyrosine 3/tryptophan 5 -monooxygenase activation protein, epsilon polypeptide	5803225	YWHAE	DSTLIMQLLR	1	9974.5	4.63	15.68	93.9	5.84	25	2
tyrosine 3/tryptophan 5 -monooxygenase activation protein, eta polypeptide	4507951	YWHAH	DSTLIMQLLR	1	28218.9	4.76	15.68	93.9	5.84	25	2
tyrosine 3/tryptophan 5 -monooxygenase activation protein, zeta polypeptide	4507953	YWHAZ	DSTLIMQLLR	2	27745.3	4.73	15.68	93.9	5.84	25	2
			TAFDEAIAELDTLSEESYK	2	27745.3	4.73	18.22	92.2	3.77	25	2
tyrosine hydroxylase isoform a	88900501	TH	AVLNLLFSPR	5	58600.5	5.9	16.57	85.4	9.79	25	2
			DFLASLAFR	5	58600.5	5.9	18.65	94.4	5.84	23	2
			FDPDLDLDHPGFSDQVYR	5	58600.5	5.9	12.42	71.9	3.97	25	3
			FSGYREDNIPQLEDVSR	5	58600.5	5.9	18.69	79.1	4.32	25	3
			TGFQLRPVAGLLSAR	5	58600.5	5.9	16.23	91.3	12	23	3
ubiquinol-cytochrome c reductase binding protein	5454152	UQCRB	DDTIYEDEDVKEAIR	2	13530.5	8.73	13.2	83.7	3.9	20	2
			YEEENFYLEPYLK	2	13530.5	8.73	21.43	94.7	4.09	25	2
ubiquinol-cytochrome c reductase, complex III subunit VII	83367083	UQCRQ	HVISYSLSPFEQR	1	48262.2	10.07	16.05	83.4	6.75	25	3
ubiquinol-cytochrome c reductase, Rieske iron-sulfur polypeptide 1	5174743	UQCRFS1 UQCF	EIEQEAAVELSQLR	3	29652.1	8.55	15.45	81.7	4.09	25	2
			GVAGALRPLVQATVPATPEQPVLDLK	3	29652.1	8.55	19.46	95.2	6.07	25	3
			SGPFAPVLSATSR	3	29652.1	8.55	17.76	93.1	9.47	25	2
ubiquitin and ribosomal protein L40 precursor	4507761	UBA52	TITLEVEPSDTIENVK	2	14728.4	9.87	20.15	92.4	4	25	2
			TLSDYNIQK	2	14728.4	9.87	14.8	94.7	5.5	21	2
ubiquitin and ribosomal protein S27a precursor	4506713	RPS27A	TITLEVEPSDTIENVK	2	13742.2	9.68	20.15	92.4	4	25	2
			TLSDYNIQK	2	13742.2	9.68	14.8	94.7	5.5	21	2
ubiquitin C	67191208	zzzzz	TITLEVEPSDTIENVK	2	77028.9	7.16	20.15	92.4	4	25	2
			TLSDYNIQK	2	77028.9	7.16	14.8	94.7	5.5	21	2
ubiquitin carboxyl-terminal esterase L1 (ubiquitin thiolesterase)	21361091	UCHL1	MQLKPMEINPEMLNK	1	80474.8	5.33	19.19	89.2	5.9	25	3
ubiquitin specific peptidase 8	41281376	USP8	KLEEKDRQEEAQRLQQK	2	127395.7	8.74	12.54	78.1	6.31	25	3
			LKDLNKKTEVKPEK	2	127395.7	8.74	10.98	78.9	9.4	25	2
ubiquitin specific protease 24	149192845	zzzzz	LEEPRITLRVRKL	2	277417.4	5.89	12.93	73.3	10.74	25	3
			LFMSEGKPY	2	277417.4	5.89	13.39	80.8	6	25	2
UDP-GlcNAc:betaGal beta-1,3-N-acetylglucosaminyltransferase 1	5802984	B3GNT1	EEAQLATVLAYALSSHCPDMR	5	47119.3	6.77	12.73	82.7	4.65	25	3
			EMLDQSNQWGGTALVVPAFEIR	5	47119.3	6.77	18.14	90.8	4.14	25	3
			SRYEAAVPDPREPGEFALLRS	5	47119.3	6.77	12.4	71.8	4.87	25	3
			TALASGGVLDASGDYR	5	47119.3	6.77	17.12	89.9	4.21	25	2
			WVNLPEESLLR	5	47119.3	6.77	11.32	75.3	4.53	25	2
UDP-N-acetyl-alpha-D-galactosamine:polypeptide N-acetylgalactosaminyltransferase 14	60498976	GALNT14	TAEVWMDEYK	1	64320.9	7.84	16.57	90.4	4.14	25	2
UDP-N-acetyl-alpha-D-galactosamine:polypeptide N-acetylgalactosaminyltransferase-like 1	71043692	GALNTL1	CLAATSTLMSSPGSPVILQMCNPR	8	63106.6	9.28	13.46	84.6	8.07	25	3
			CQADAQAQQWQLLPHT	8	63106.6	9.28	18.6	95	5.08	25	2
			NPQPAQAWLFSDHLIQQQGK	8	63106.6	9.28	16.31	76.7	6.74	25	3
			QGVNCLESQGQNTAGDFLLGMGICR	8	63106.6	9.28	17.48	83.6	4.37	25	3
			TAEVWMDEYK	8	63106.6	9.28	16.57	90.4	4.14	25	2
			TPANLIQEIILVDDFSSDPEDCLLLTR	8	63106.6	9.28	15.36	81	3.66	25	3
			TPVIAGGIFVIDK	8	63106.6	9.28	19.23	89	5.5	25	2
			VVSPIIDVISLDNFAYLAASADLR	8	63106.6	9.28	12.34	74.7	3.93	25	3
upregulated during skeletal muscle growth 5	14249376	USMG5	AGPESDAQYQFTGIK	2	6457.6	9.78	23.45	98.6	4.37	25	2
			AGPESDAQYQFTGIKK	2	6457.6	9.78	13.15	84.8	6.11	25	3
vacuolar H+ ATPase E1 isoform a	4502317	ATP6V1E1	ARDDLITDLLNEAK	7	26145.5	7.7	20.69	94.9	4.23	25	3
			DDLITDLLNEAK	7	26145.5	7.7	13.48	86.8	3.84	25	2
			HMMAFIEQEANEK	7	26145.5	7.7	15.6	85.5	4.75	25	3
			IQMSNLMNQAR	7	26145.5	7.7	20.39	87.9	9.75	25	2
			KIQMSNLMNQAR	7	26145.5	7.7	12.75	77.6	11	25	3
			LDLIAQQMMPEVR	7	26145.5	7.7	20.05	92.1	4.37	25	2
			NDVDVQIDQESYLPEDIAGGVEIYNGDR	7	26145.5	7.7	21.27	92.9	3.54	25	3
vacuolar H+ ATPase E1 isoform c	87159818	ATP6V1E1	HMMAFIEQEANEK	5	22706.4	6.66	15.6	85.5	4.75	25	3
			IQMSNLMNQAR	5	22706.4	6.66	20.39	87.9	9.75	25	2
			KIQMSNLMNQAR	5	22706.4	6.66	12.75	77.6	11	25	3
			LDLIAQQMMPEVR	5	22706.4	6.66	20.05	92.1	4.37	25	2
			NDVDVQIDQESYLPEDIAGGVEIYNGDR	5	22706.4	6.66	21.27	92.9	3.54	25	3
vacuolar H+ ATPase G1	4757818	ATP6V1G1	EEAQAEIEQYR	3	13757.6	8.93	14.05	80.7	4.09	25	2
			MTILQTYFR	3	13757.6	8.93	11.44	75	8.5	25	2
			QAKEEAQAEIEQYR	3	13757.6	8.93	15.26	85.2	4.5	25	3
vacuolar H+ATPase B2	19913428	ATP6V1B2	AVVQVFEGTSGIDAK	3	56501	5.57	15.22	77.2	4.37	25	2
			TVFETLDIGWQLLR	3	56501	5.57	11.15	73.8	4.37	25	2
			TVSGVNGPLVILDHVK	3	56501	5.57	12.91	91.1	6.41	25	3
valosin-containing protein	6005942	VCP	ETVVEVPQVTWEDIGGLEDVKR	1	89322.3	5.14	15.18	87.8	4.08	25	3
VAMP-associated protein B/C	4759302	VAPB	FMVQSMFAPTDTSDMEAVWK	3	11309.2	6.85	10.78	82	4.03	20	2
			GPFTDVVTTNLK	3	11309.2	6.85	16.02	83.9	5.84	25	2
			TVQSNSPISALAPTGK	3	11309.2	6.85	13.73	83.9	8.41	25	2
vesicle amine transport protein 1	18379349	VAT1	AVCGFHLGYLDGEVELVSGVVAR	9	41920.5	5.88	12.74	74.2	4.65	25	3
			CLVLTGFGGYDK	9	41920.5	5.88	14.32	89.9	5.83	25	2
			EVAEAATGEDASSPPPK	9	41920.5	5.88	12.04	96.1	4	12	2
			GVDIVMDPLGGSDTAK	9	41920.5	5.88	11.96	96.1	3.93	10	2
			LPPLPVTPGMEGAGVVIAVGEGVSDRK	9	41920.5	5.88	14.97	74.3	4.68	25	3
			PAAPPAPGPGQLTLR	9	41920.5	5.88	19.2	94.8	10.18	24	2
			TEAASDPQHPAASEGAAAAAASPPLLR	9	41920.5	5.88	14.12	94	4.65	20	3
			TWWNQFSVTALQLLQANR	9	41920.5	5.88	13.22	82.4	9.41	25	3
			VLLVPGPEKEN	9	41920.5	5.88	15.01	88.6	4.53	25	2
vesicle-associated membrane protein 2 (synaptobrevin 2)	7657675	VAMP2	ADALQAGASQFETSAAK	6	12648.8	7.84	20.46	93.9	4.37	25	2
			AQVDEVVDIMR	6	12648.8	7.84	16.18	84	4.03	25	2
			LQQTQAQVDEVVDIMR	6	12648.8	7.84	17.05	92.2	4.03	25	2
			LSELDDRADALQAGASQFETSAAK	6	12648.8	7.84	17.73	85	4.11	25	3
			RLQQTQAQVDEVVDIMR	6	12648.8	7.84	17.62	70.5	4.56	25	3
			TAPPAAPAGEGGPPAPPPNLTSNR	6	12648.8	7.84	12.74	71.2	5.66	25	3
vesicle-associated membrane protein 3 (cellubrevin)	4759300	VAMP3	ADALQAGASQFETSAAK	3	11309.2	8.89	20.46	93.9	4.37	25	2
			LQQTQNQVDEVVDIMR	3	11309.2	8.89	15.75	78.3	4.03	25	2
			LSELDDRADALQAGASQFETSAAK	3	11309.2	8.89	17.73	85	4.11	25	3
vesicle-associated membrane protein-associated protein A isoform 1	94721250	VAPA	DKLGITPPGNAPTV	6	32613.7	9.03	15.74	88.4	5.84	25	3
			FKGPFTDVVTTNLK	6	32613.7	9.03	18.44	88.9	8.59	25	3
			FMVQTIFAPPNTSDMEAVWK	6	32613.7	9.03	13.96	84.4	4.37	25	2
			GPFTDVVTTNLK	6	32613.7	9.03	16.02	83.9	5.84	25	2
			HEQILVLDPPTDLK	6	32613.7	9.03	21.72	92	4.54	25	3
			SGIIDPGSTVTVSVMLQPFDYDPNEK	6	32613.7	9.03	13.24	70.1	3.84	25	3
vesicle-associated membrane protein-associated protein A isoform 2	94721252	VAPA	FKGPFTDVVTTNLK	5	27893.4	8.8	18.44	88.9	8.59	25	3
			FMVQTIFAPPNTSDMEAVWK	5	27893.4	8.8	13.96	84.4	4.37	25	2
			GPFTDVVTTNLK	5	27893.4	8.8	16.02	83.9	5.84	25	2
			HEQILVLDPPTDLK	5	27893.4	8.8	21.72	92	4.54	25	3
			SGIIDPGSTVTVSVMLQPFDYDPNEK	5	27893.4	8.8	13.24	70.1	3.84	25	3
VGF nerve growth factor inducible precursor	17136078	VGF	AYQGVAAPFPK	9	67258.1	4.76	13.29	83.9	8.63	25	2
			LADLASDLLLQYLLQGGAR	9	67258.1	4.76	20.85	94.7	4.21	25	2
			LHLPADDVVSIIEEVEEK	9	67258.1	4.76	18.29	91.5	4.07	25	3
			LLQQGLAQVEAGR	9	67258.1	4.76	13.12	74.4	6	25	2
			NAPPEPVPPPR	9	67258.1	4.76	15.03	90.8	6	25	2
			NSEPQDEGELFQGVDPR	9	67258.1	4.76	14.64	82.6	3.83	22	3
			RPESALLGGSEAGER	9	67258.1	4.76	21.73	92.6	4.79	25	3
			TLQPPSALR	9	67258.1	4.76	12.19	97.6	9.41	14	2
			VGEEDEEAAEAEAEAEEAER	9	67258.1	4.76	21.64	95.1	3.59	25	2
vimentin	62414289	VIM	DGQVINETSQHHDDLE	24	53651.9	5.06	14.18	84.2	4.28	25	3
			DNLAEDIMR	24	53651.9	5.06	15.42	85.2	4.03	25	2
			EEAENTLQSFR	24	53651.9	5.06	15.3	85	4.25	25	2
			EMEENFAVEAANYQDTIGR	24	53651.9	5.06	15.1	82.8	3.91	25	3
			EYQDLLNVK	24	53651.9	5.06	12.55	93.2	4.37	25	2
			FADLSEAANR	24	53651.9	5.06	20.69	97.5	4.37	25	2
			ILLAELEQLKGQGK	24	53651.9	5.06	15.73	97.1	6.14	24	3

			ISLPLPNFS	24	53651.9	5.06	12.64	91.3	5.52	25	2
			ISLPLPNFSSL	24	53651.9	5.06	16.58	96.1	5.52	22	2
			ISLPLPNFSSLN	24	53651.9	5.06	17.64	94.6	5.52	25	2
			ISLPLPNFSSLNL	24	53651.9	5.06	18.09	94.7	5.52	25	2
			ISLPLPNFSSLNLR	24	53651.9	5.06	14.83	79.7	9.75	25	2
			KVESLQEEIAFLK	24	53651.9	5.06	19.43	95.6	4.79	25	3
			LQDEIQNMKEEMAR	24	53651.9	5.06	13.67	82.4	4.41	25	3
			LQEEMLQREEAENTLQSFR	24	53651.9	5.06	17.13	86.8	4.33	25	3
			MALDIEIATYR	24	53651.9	5.06	19.72	96.6	4.37	25	2
			NLQEAEEWYK	24	53651.9	5.06	13.25	84.2	4.25	25	2
			QVQSLTCEVDALK	24	53651.9	5.06	17.03	85.5	4.37	25	2
			QVQSLTCEVDALKGTNESLER	24	53651.9	5.06	17.38	85.1	4.41	25	3
			SLYASSPGGVYATR	24	53651.9	5.06	14.05	76.9	8.31	25	2
			TNEKVELQELNDR	24	53651.9	5.06	10.88	93.2	4.41	16	2
			TYSLGSALRPSTSR	24	53651.9	5.06	13.26	85.5	10.83	25	3
			VELQELNDR	24	53651.9	5.06	19.33	95.7	4.14	25	2
			VEVERDNLAEDIMR	24	53651.9	5.06	12.12	84.5	4.18	24	3
voltage-dependent anion channel 1	4507879	VDAC1	GYGFGLIK	11	30772.7	8.62	11.16	94.4	8.59	14	2
			KLETAVNLAWTAGNSNTR	11	30772.7	8.62	14.75	86.1	8.75	25	3
			LTFDSSFSPNTGK	11	30772.7	8.62	16.73	92.7	5.84	25	2
			LTFDSSFSPNTGKK	11	30772.7	8.62	12.92	84.1	8.59	25	3
			LTLSALLDGK	11	30772.7	8.62	13.69	84.1	5.84	25	2
			SENGLEFTSSGSANTETTK	11	30772.7	8.62	16.25	89.5	4.25	25	2
			VNNSSLIGLGYTQTLKPGIK	11	30772.7	8.62	11.59	70	9.7	25	2
			VTQSNFAVGYK	11	30772.7	8.62	15.92	91.5	8.56	25	2
			WNTDNTLGTEITVEDQLAR	11	30772.7	8.62	17.16	86	3.91	25	2
			WTEYGLTFTEK	11	30772.7	8.62	14.91	87.4	4.53	25	2
			YQIDPDACFSAK	11	30772.7	8.62	10.54	77.8	4.21	25	2
voltage-dependent anion channel 2	42476281	VDAC2	LTFDTTFSPNTGK	2	31566.7	7.5	15.17	83.4	5.84	25	2
			LTLSALVDGK	2	31566.7	7.5	13.59	89.4	5.84	25	2

Table S2 Addendum.

Tandem mass spectrometry data for single peptide identification of proteins in the membrane fraction of human dense core secretory vesicles (DCSV).

Adenylate Kinase 1 AK1

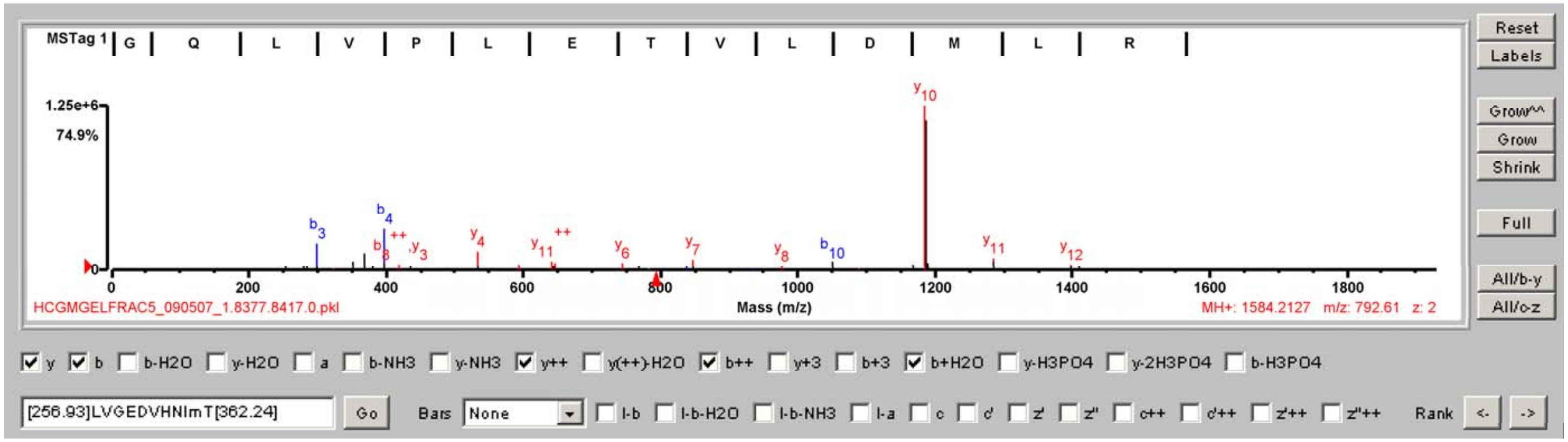

ADP-Ribosylation Factor-Like 6 Interacting Protein ARL6IP1

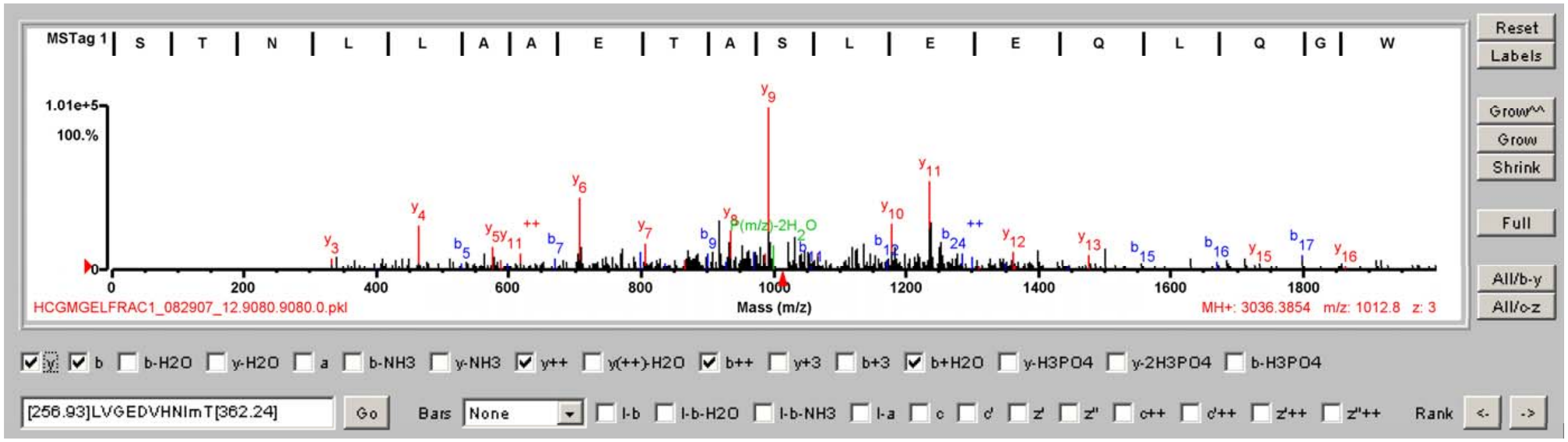

ARP1 Actin-related Protein 1 Homolog A ACTR1A

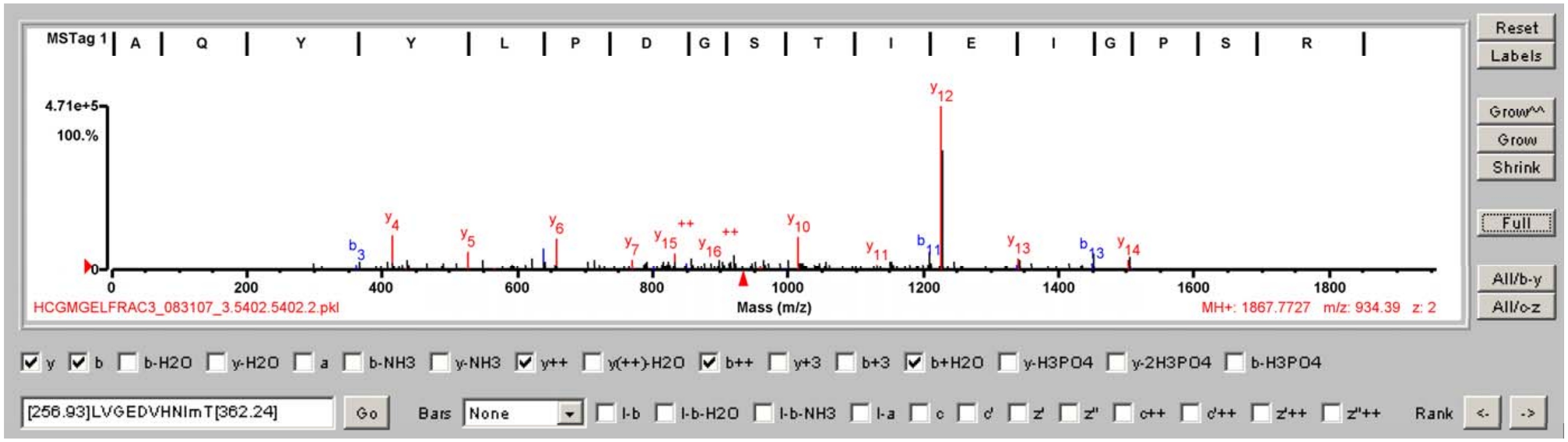

ATP Synthase F0 Subunit 8 MT-ATP6

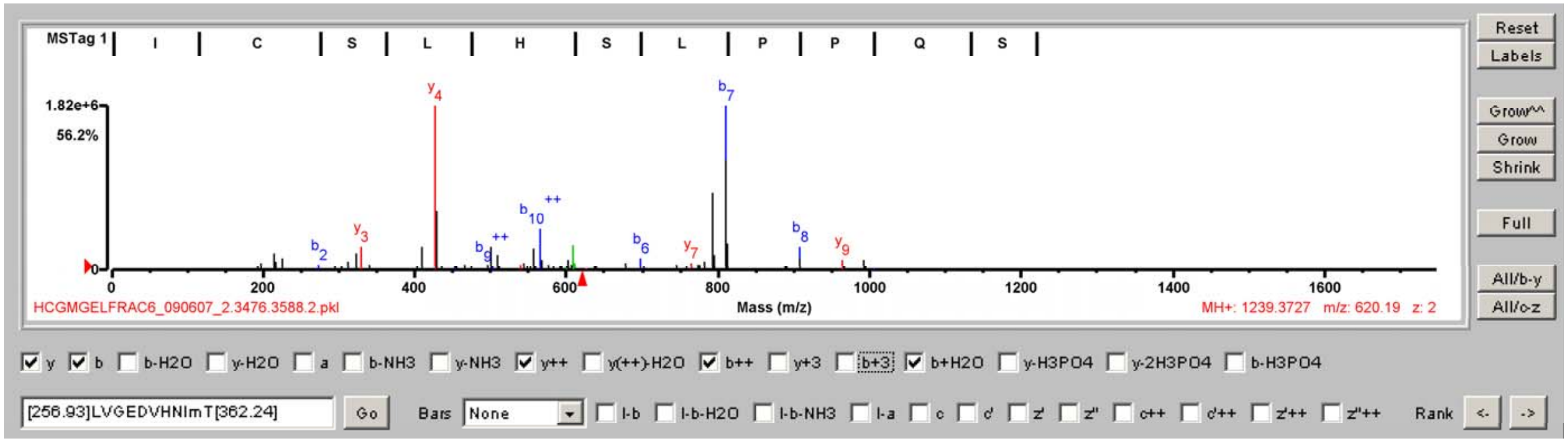

ATP Synthase H+ Transporting, Mitochondrial 5D ATP5D

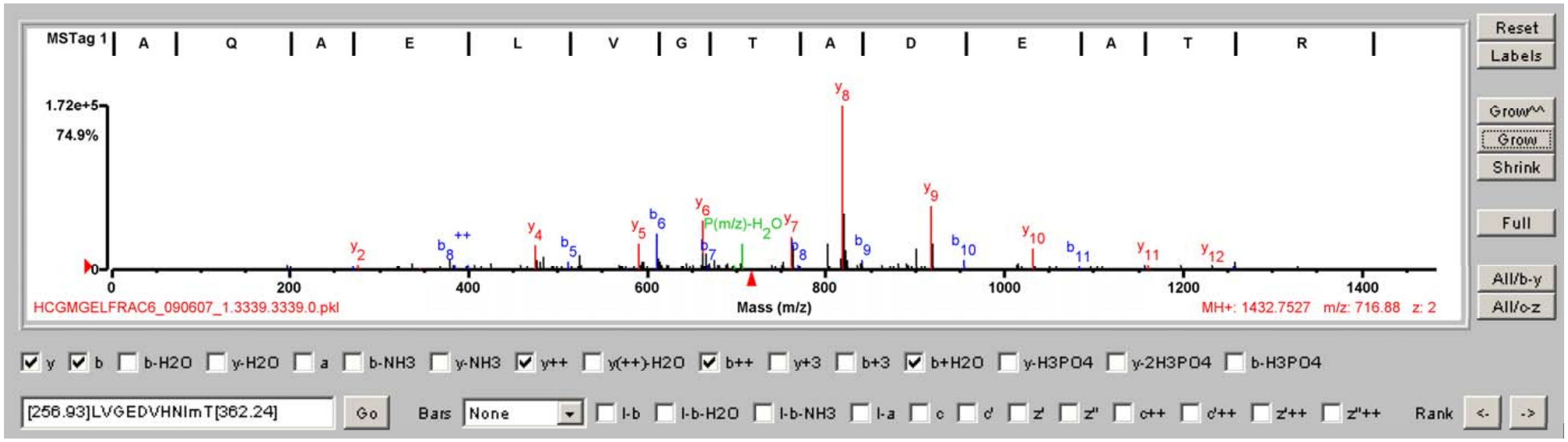

ATP Synthase H+ Transporting, Mitochondrial 5C 1 ATP5C1

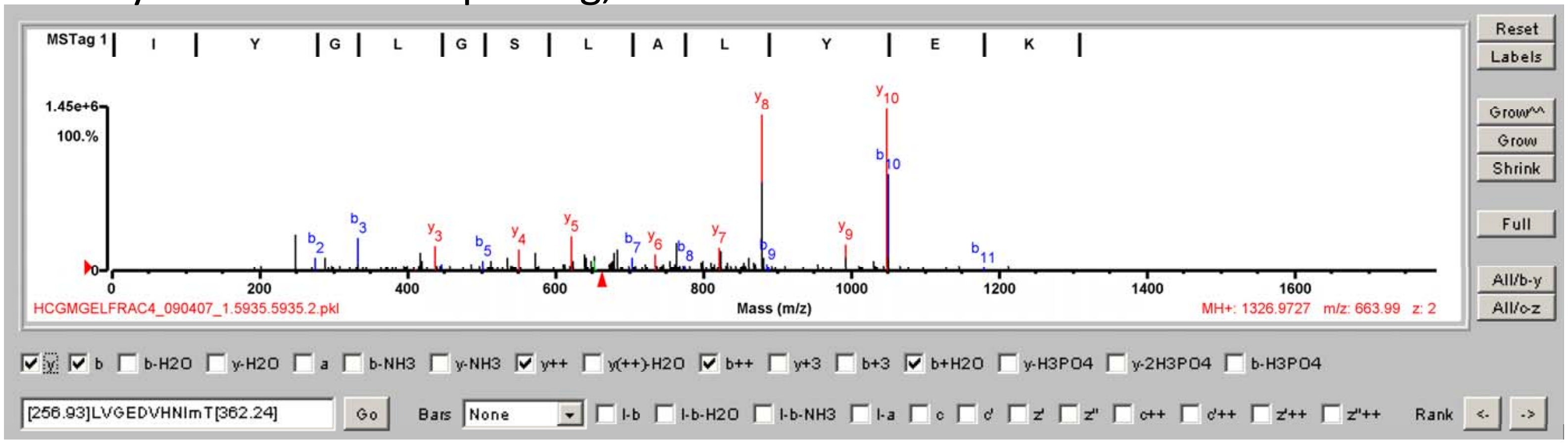

ATPase, Aminophospholipid Transporter 8A1 ATP8A1

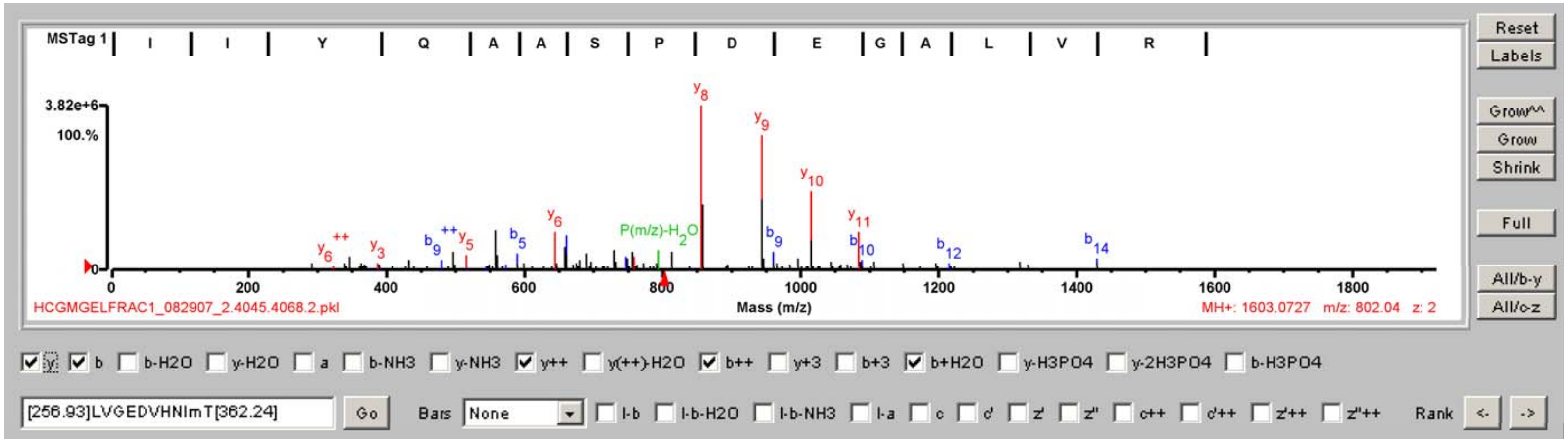

ATPase H+ Transporting, Lysosomal 31kD ATP6V1E2

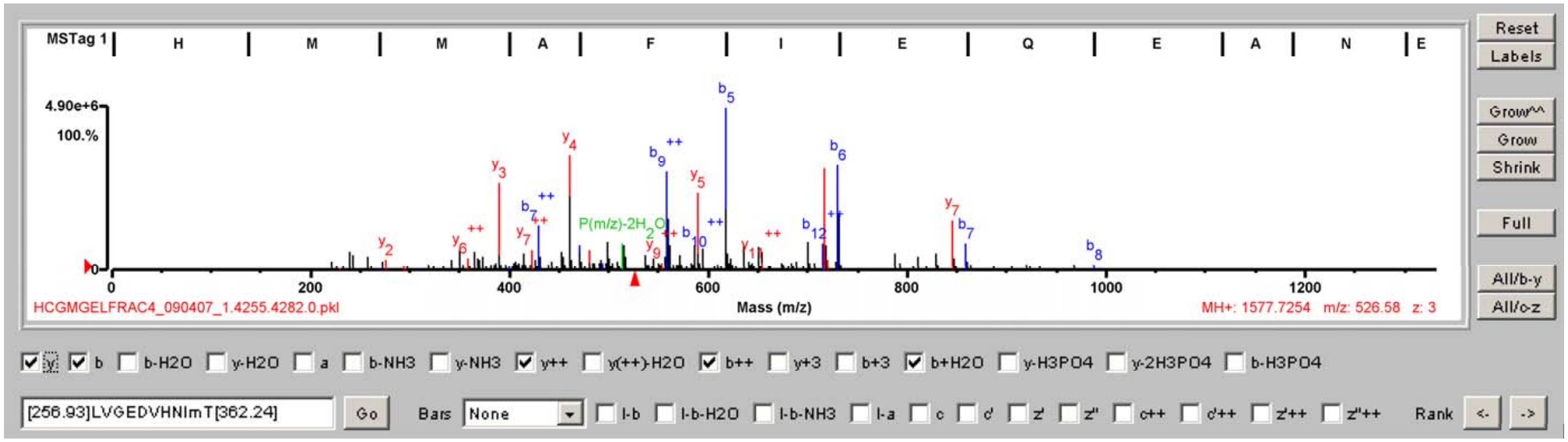

Barrier to Autointegration Factor 1 BANF1

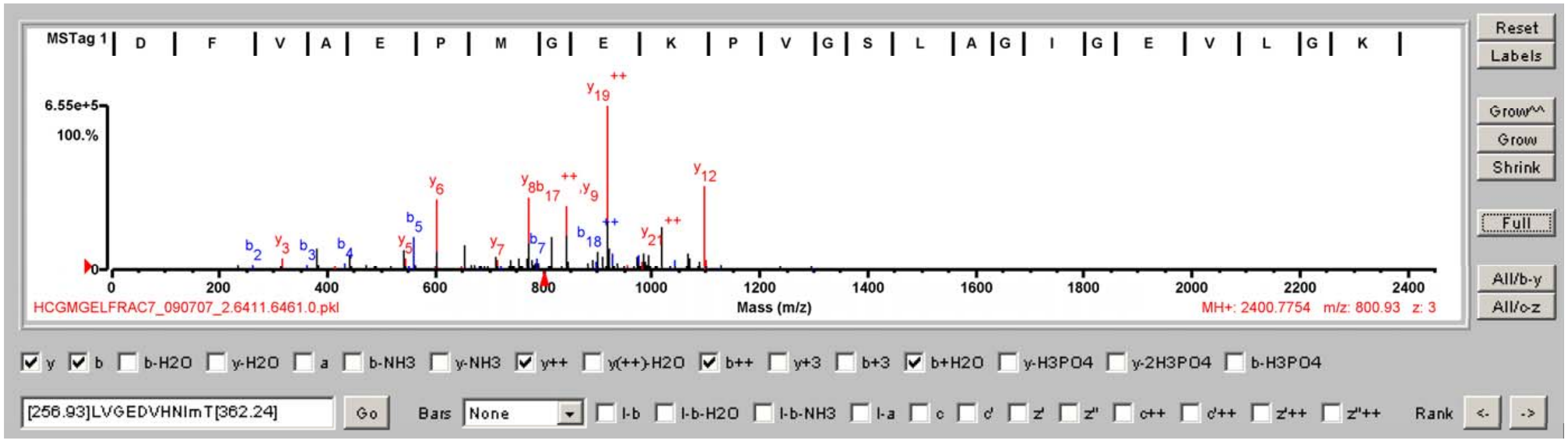

Brain Creatine Kinase CKB

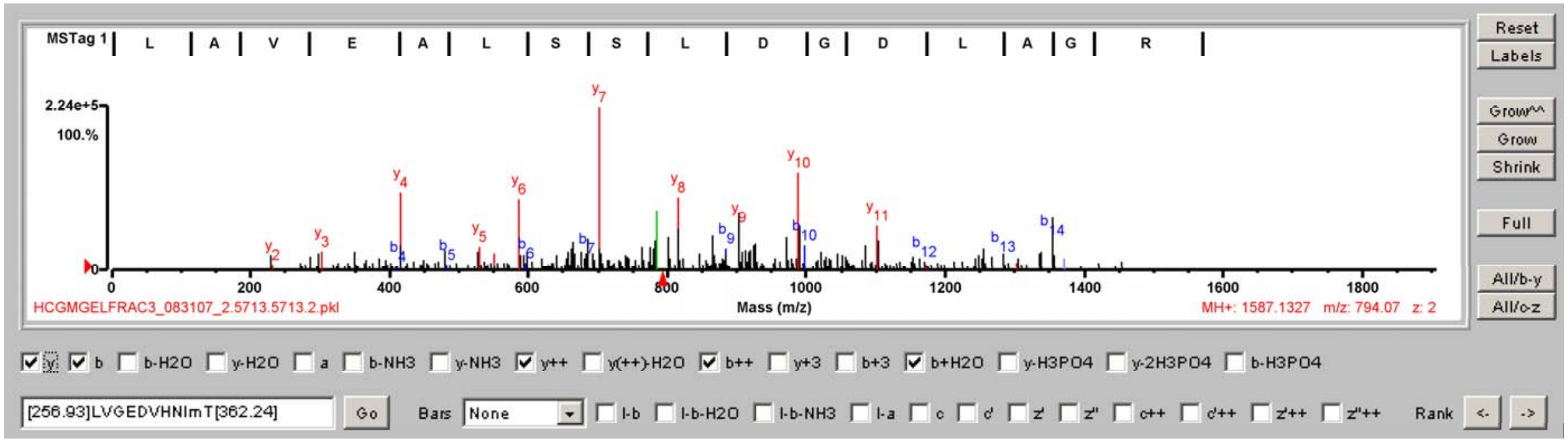

Cardiac Calsequestrin 2 CASQ2

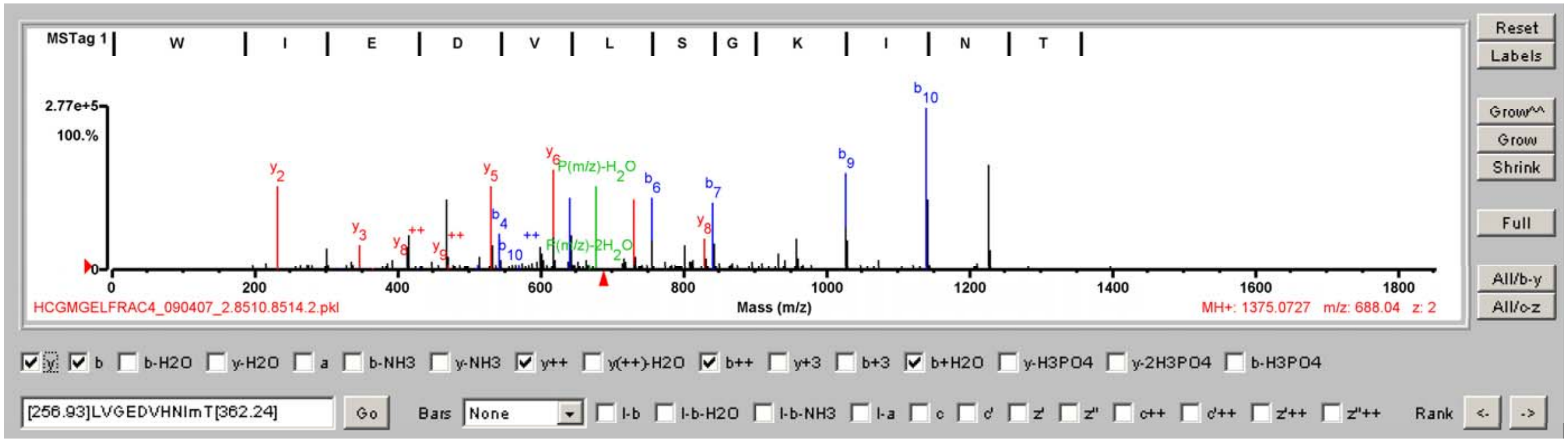

CASK Interacting Protein 1 CASKIN1

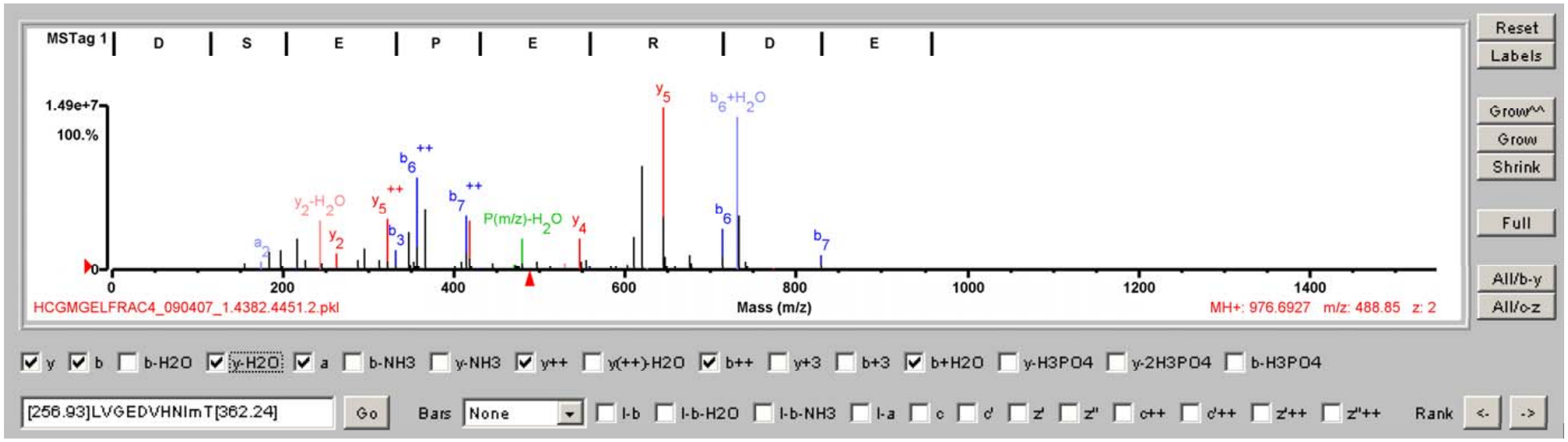

Cathepsin Z Preproprotein CTSZ

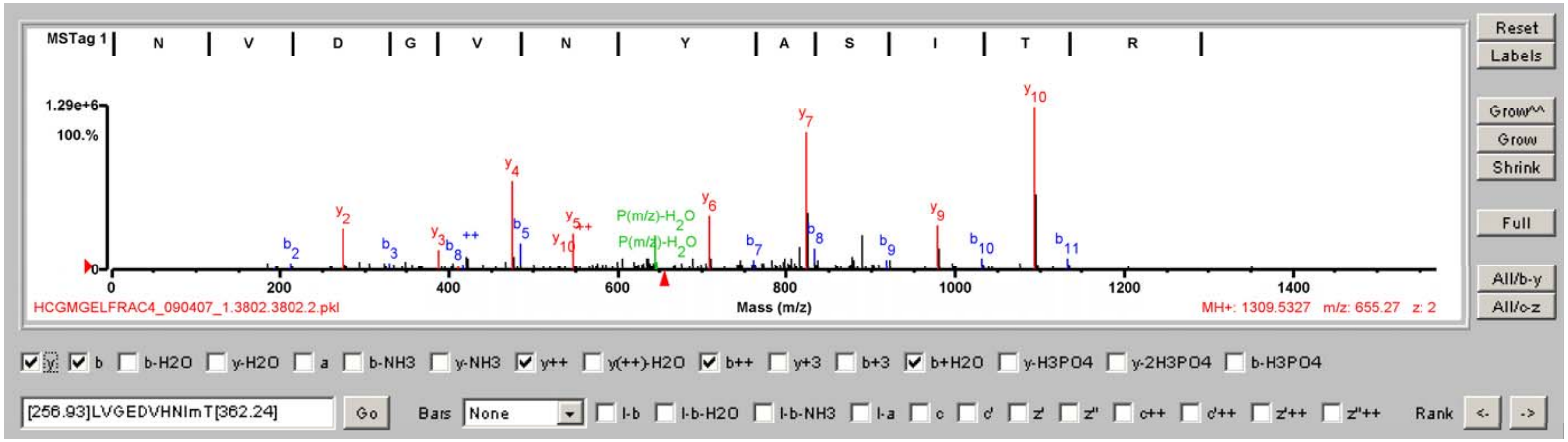

CD59 Antigen p18-20 CD59

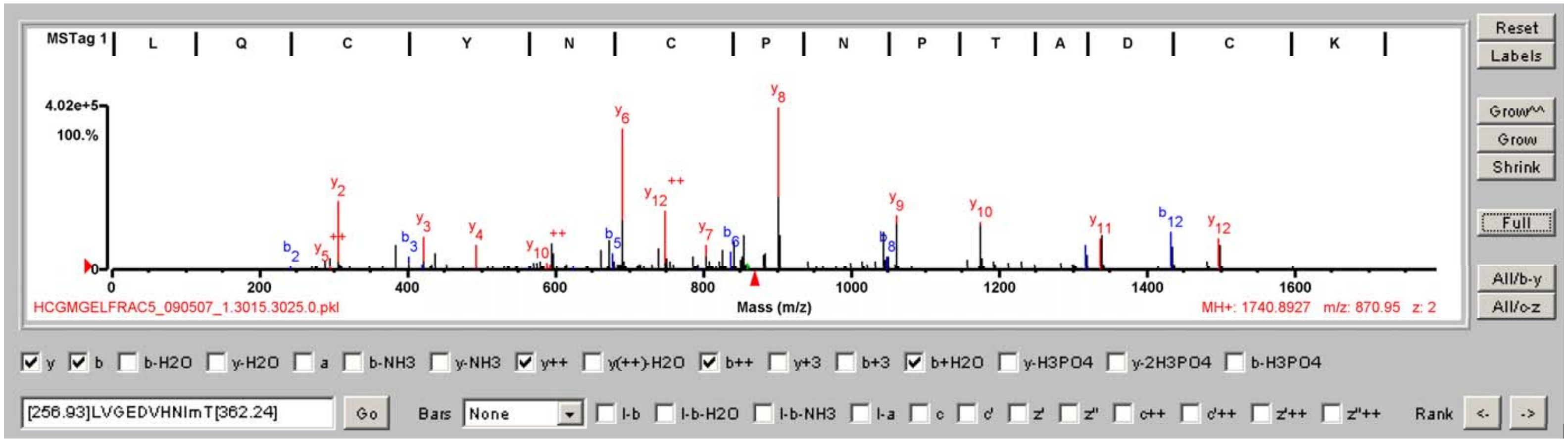

Cofilin 2 CFL2

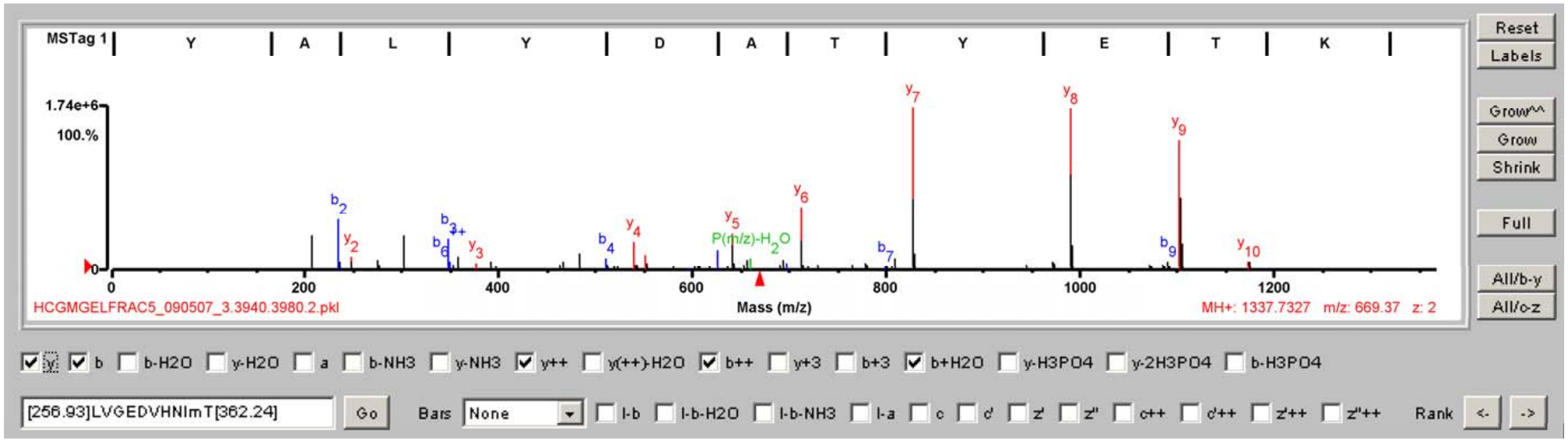

Complement Component 7 Precursor C7

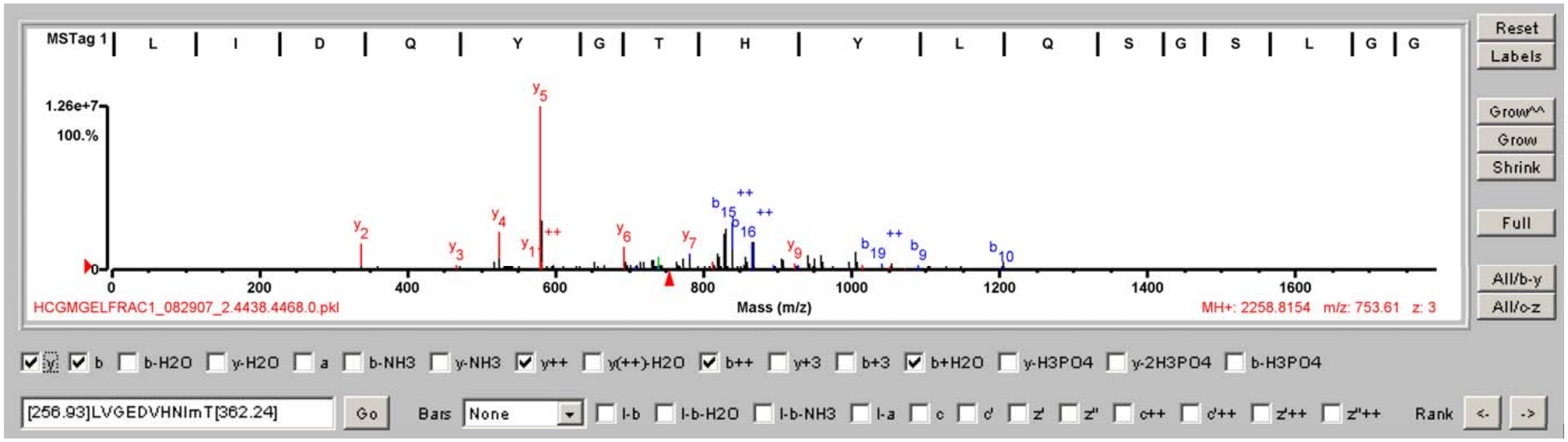

Cytochrome C Oxidase Subunit VIIc COX7C

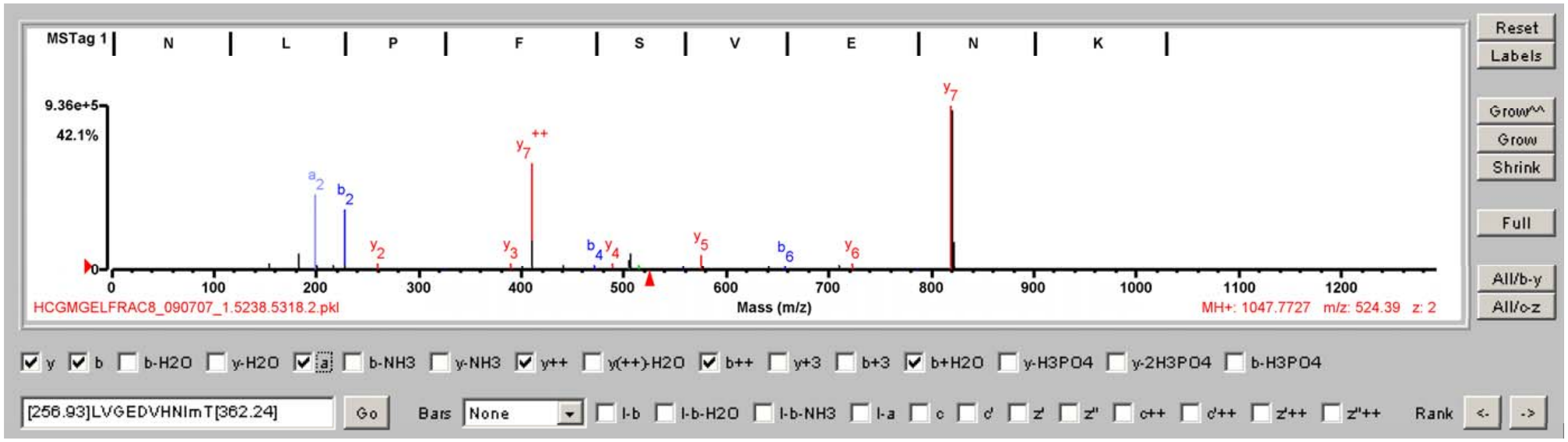

Cytosolic Sialic Acid 9-O-acetylesterase Homolog SIAE

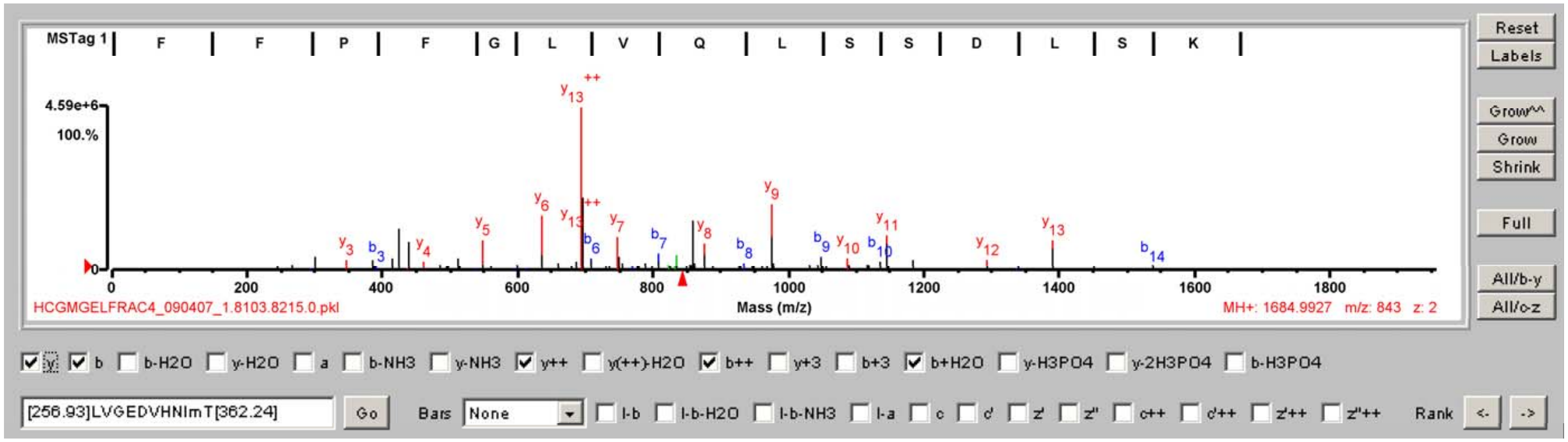

Dicarbonyl/L-Xylulose Reductase DCXR

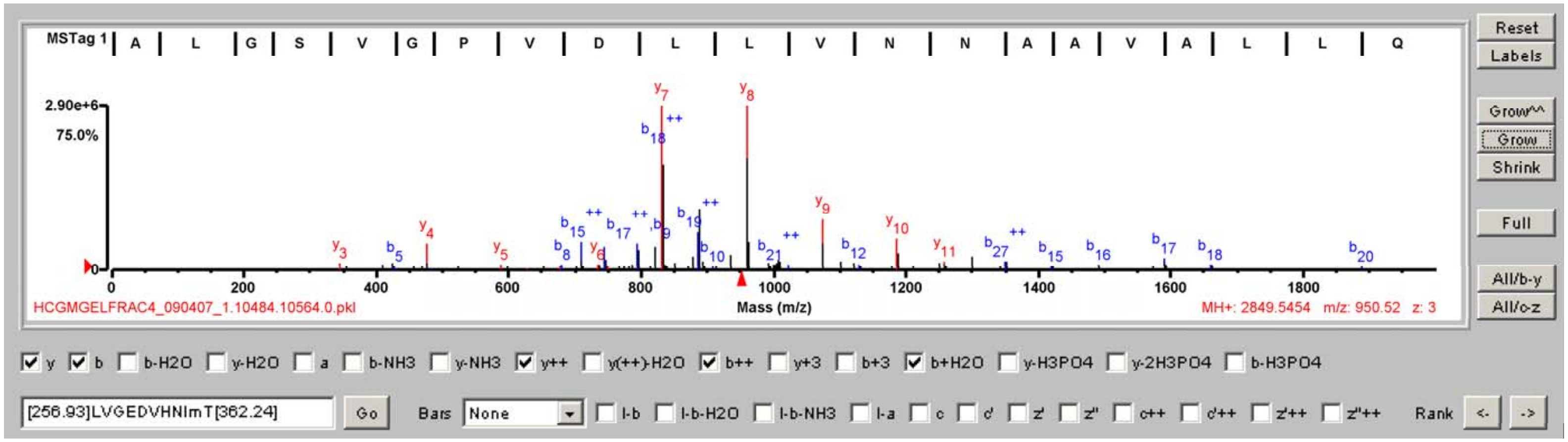

Dipeptidyl Peptidase 7 Preproprotein DPP7

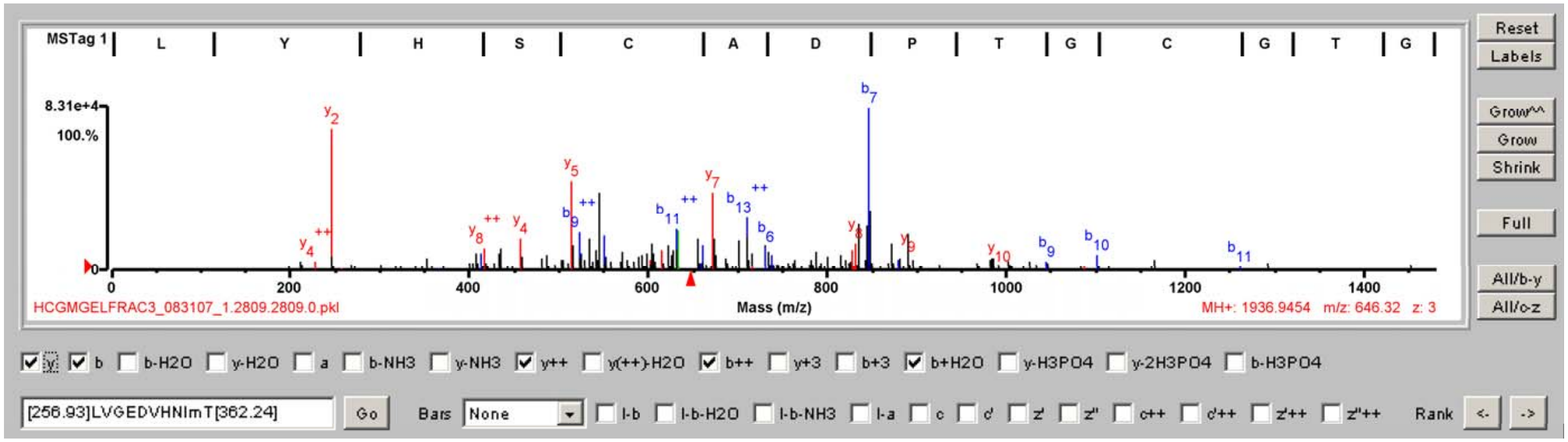

Dynactin 2 DCTN2

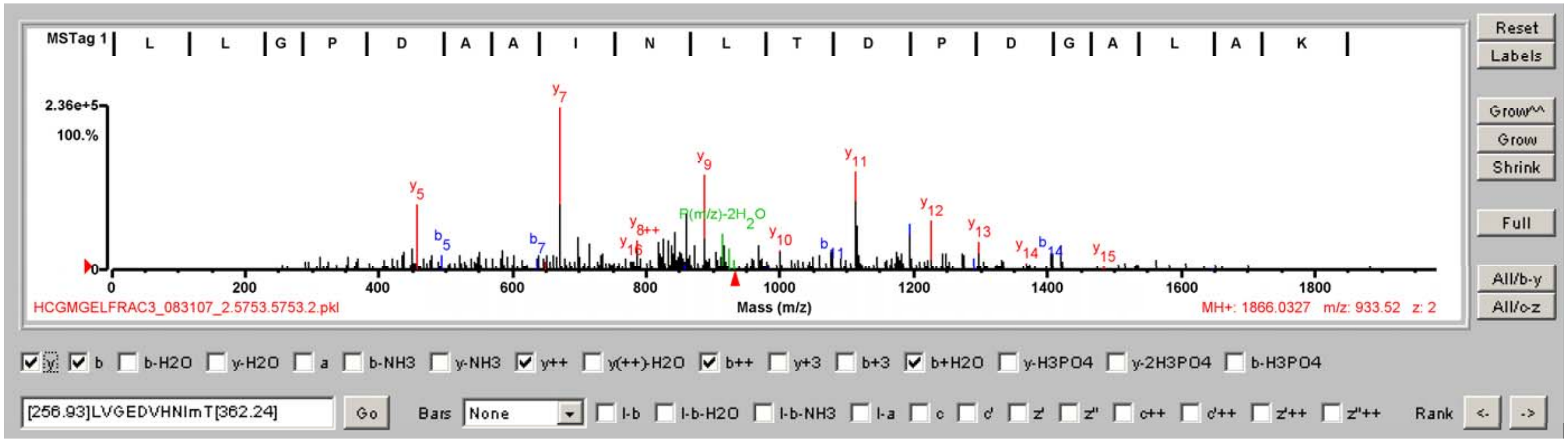

Enolase 3 ENO3

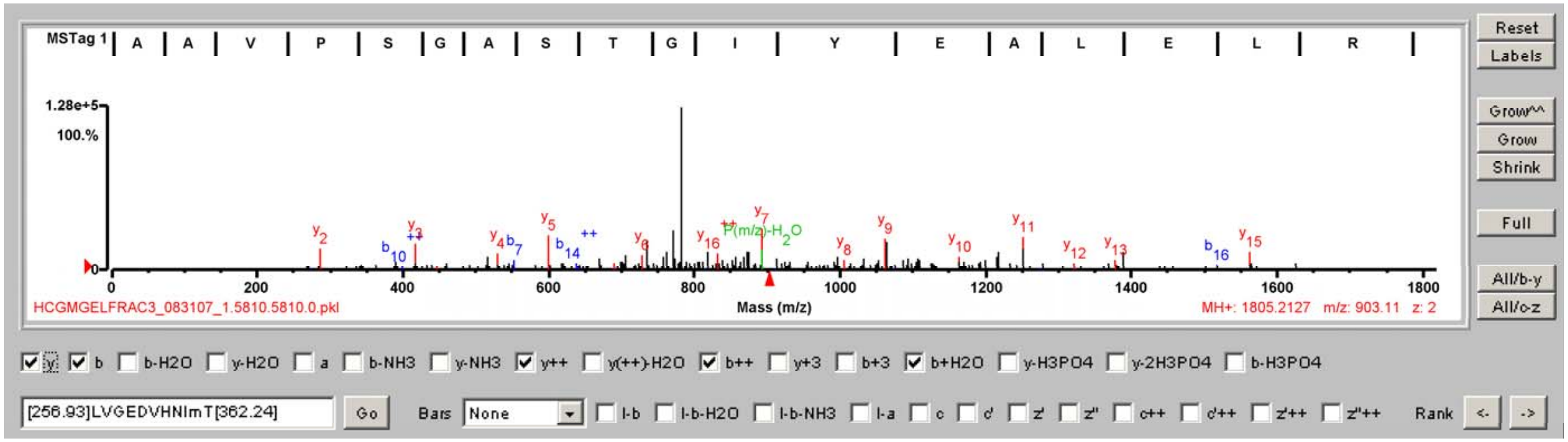

Fc Fragment of IgE, High Affinity I Receptor FCER1G

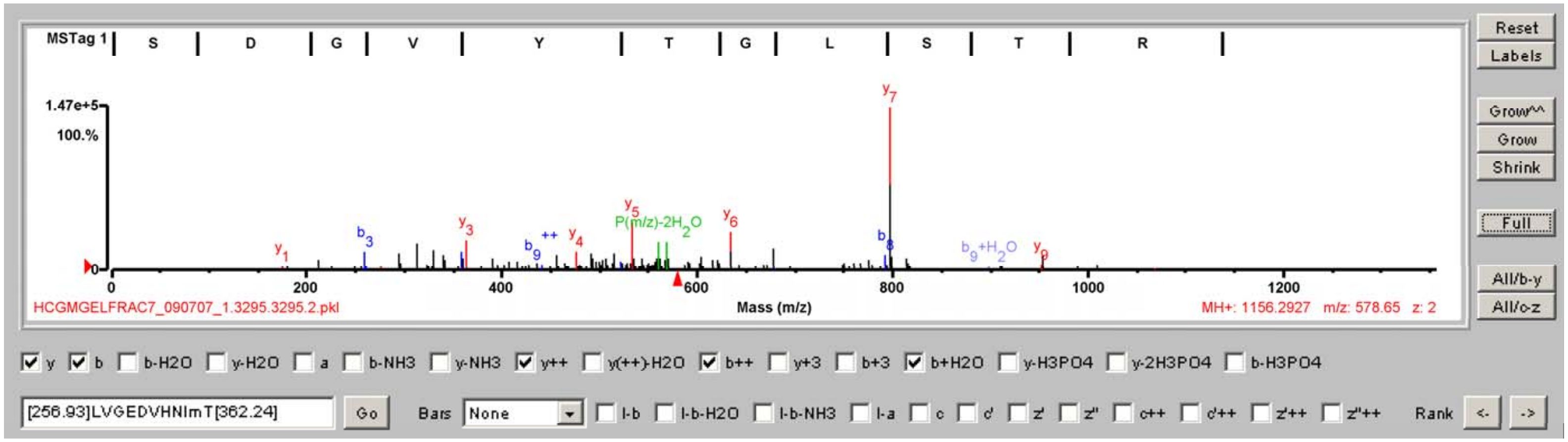

FK506 Binding Protein 3

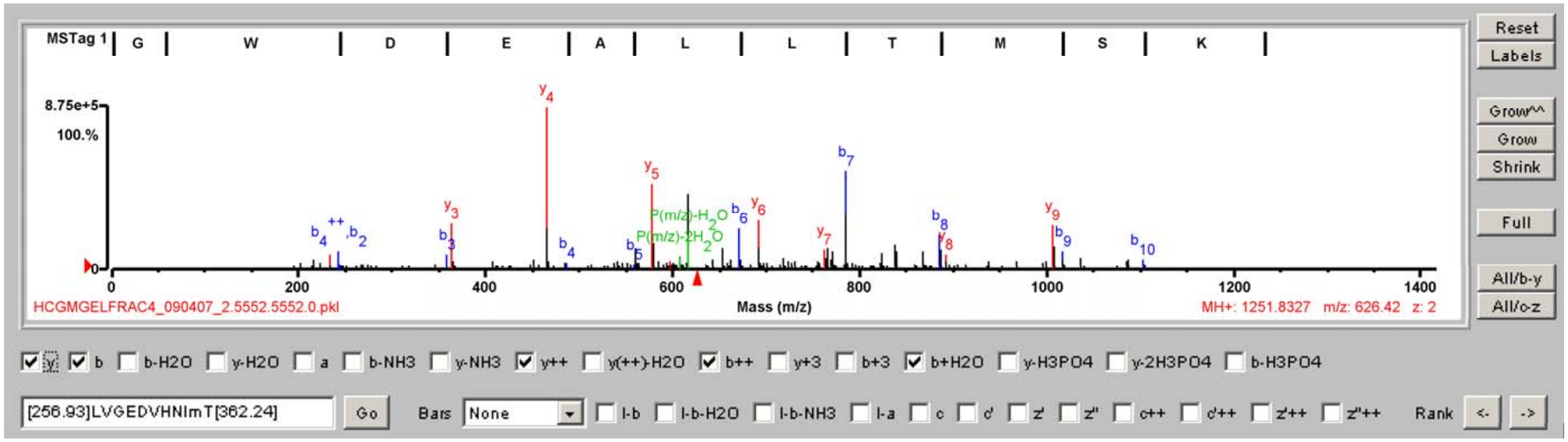

Glucose Phosphate Isomerase GPI

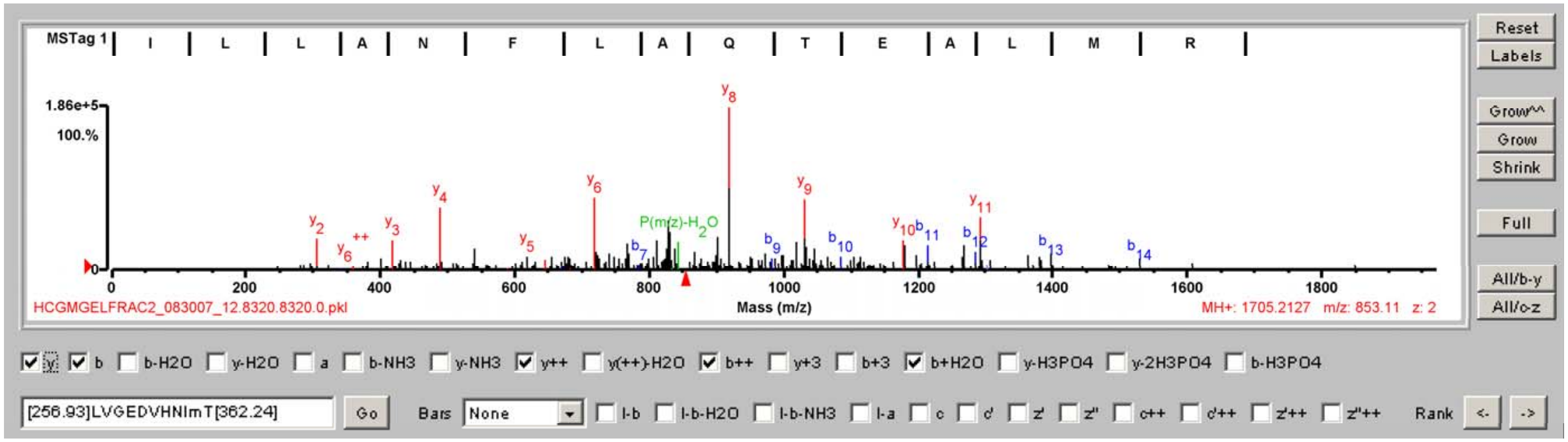

Glutathione Peroxidase 1 Isoform 1 and 2 GPX1

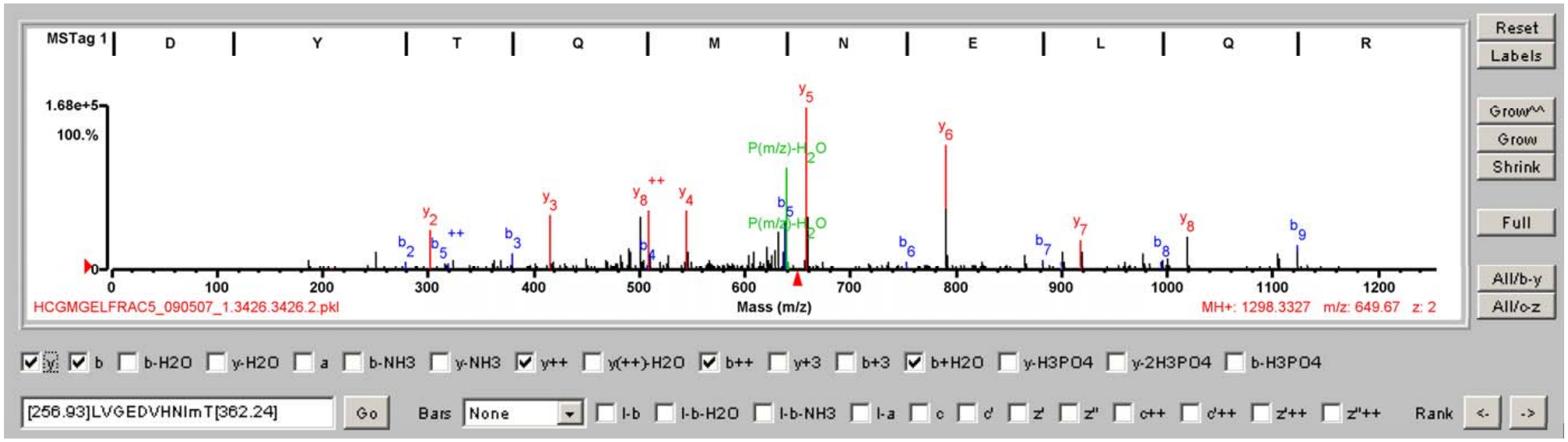

Grp-E-like 1 Mitochondrial GRPEL1

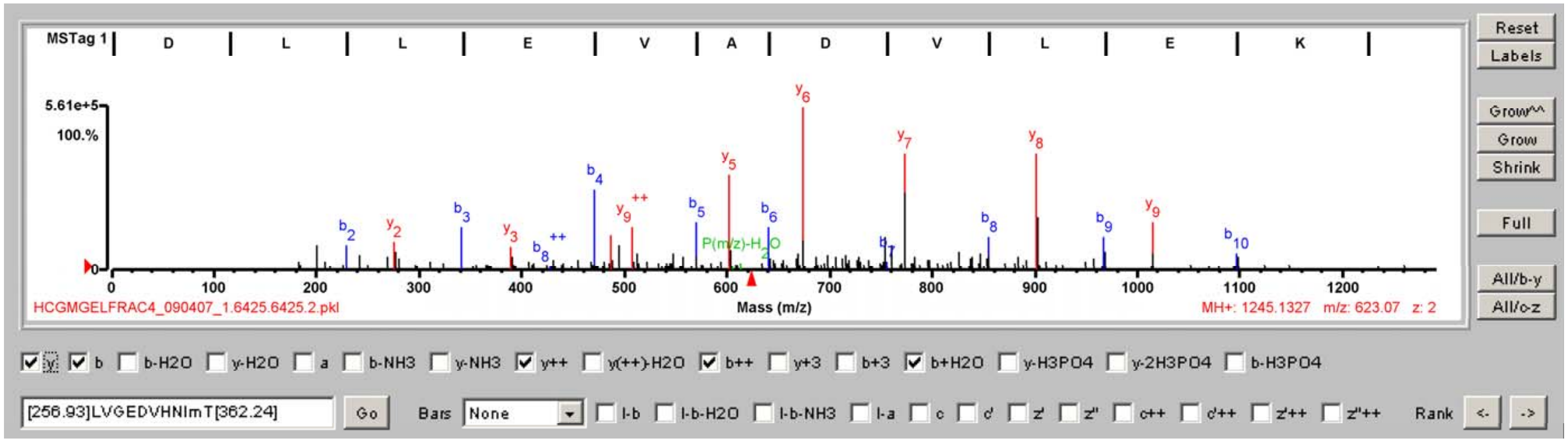

Guanine Nucleotide Binding Proteins GNAL, GNAI2, GNAI3, GNA12, GNAT1, GNAT 2

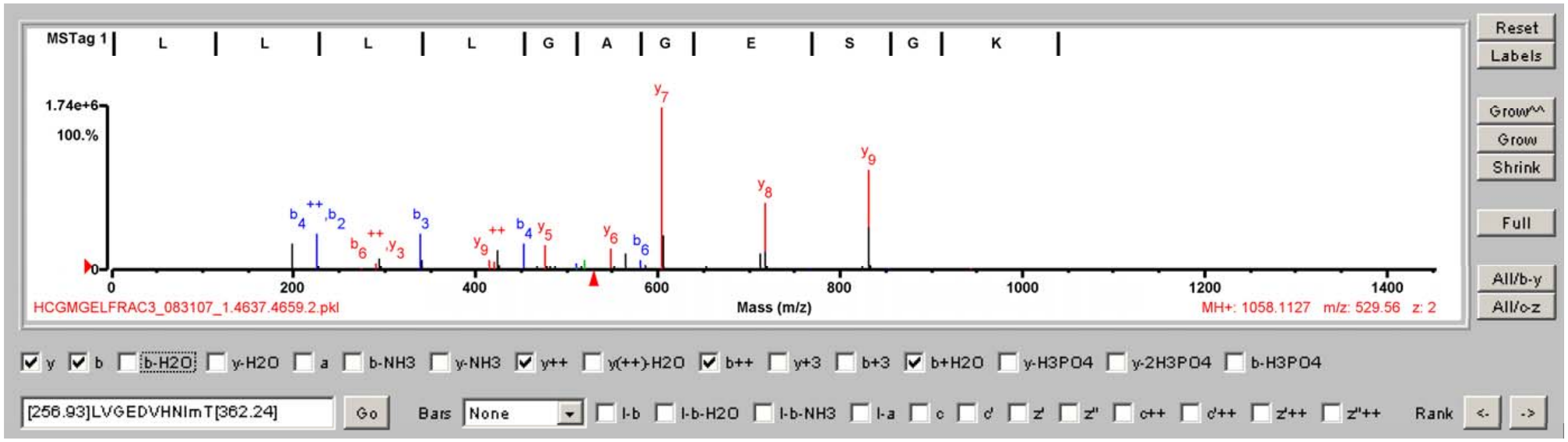

Guanine Nucleotide Binding Protein 8 CNG8

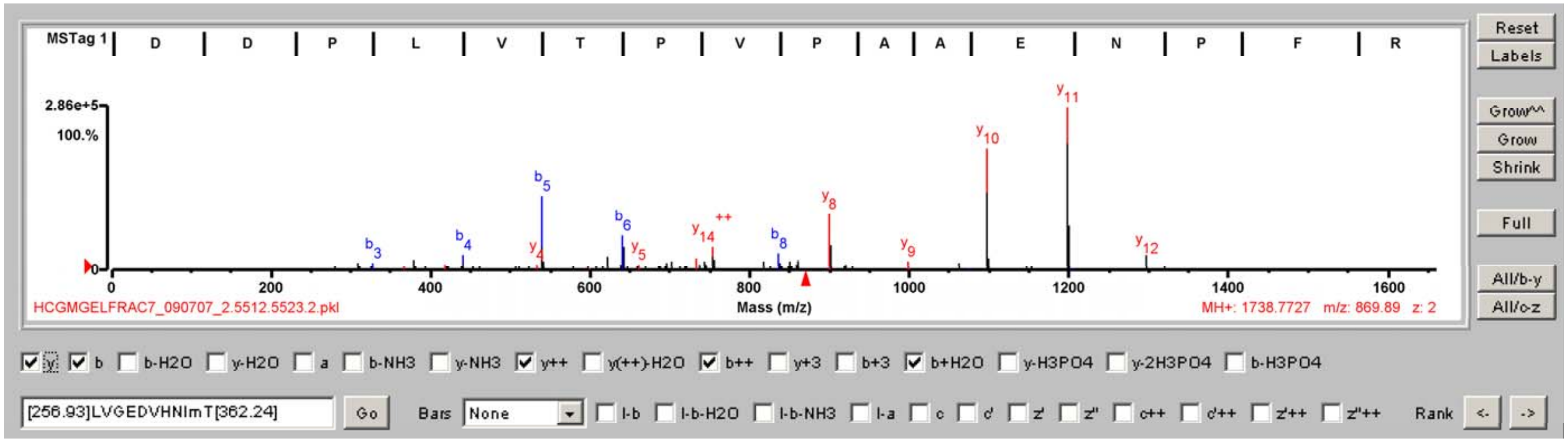

Hydroxyacyl Glutathione Hydrolase Isoform HAGH

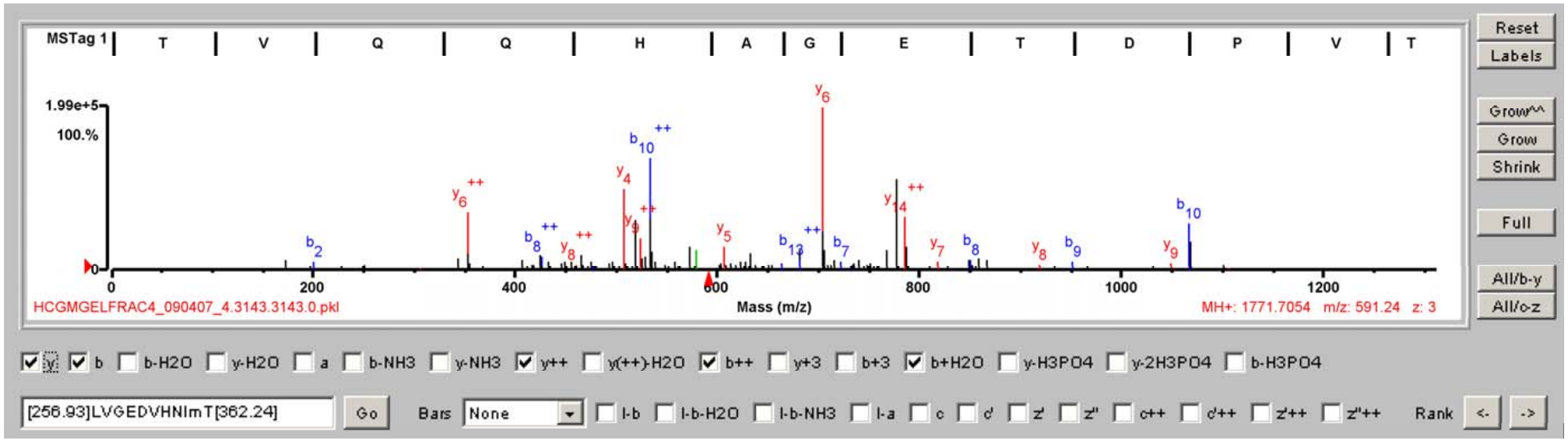

LOC374882 TMEM205

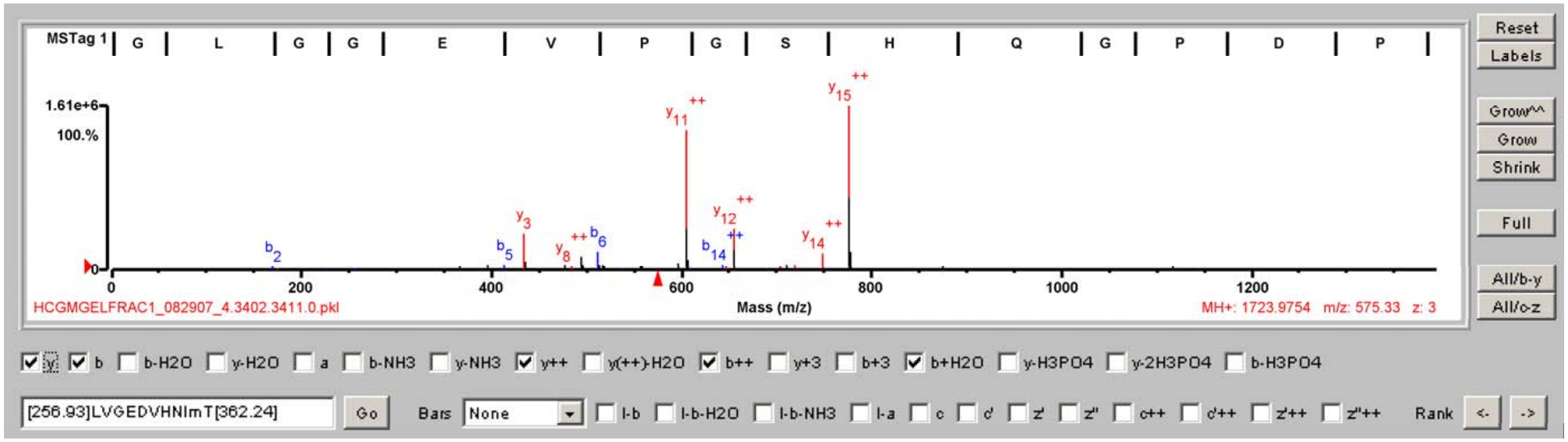

LOC414919 C8orf82

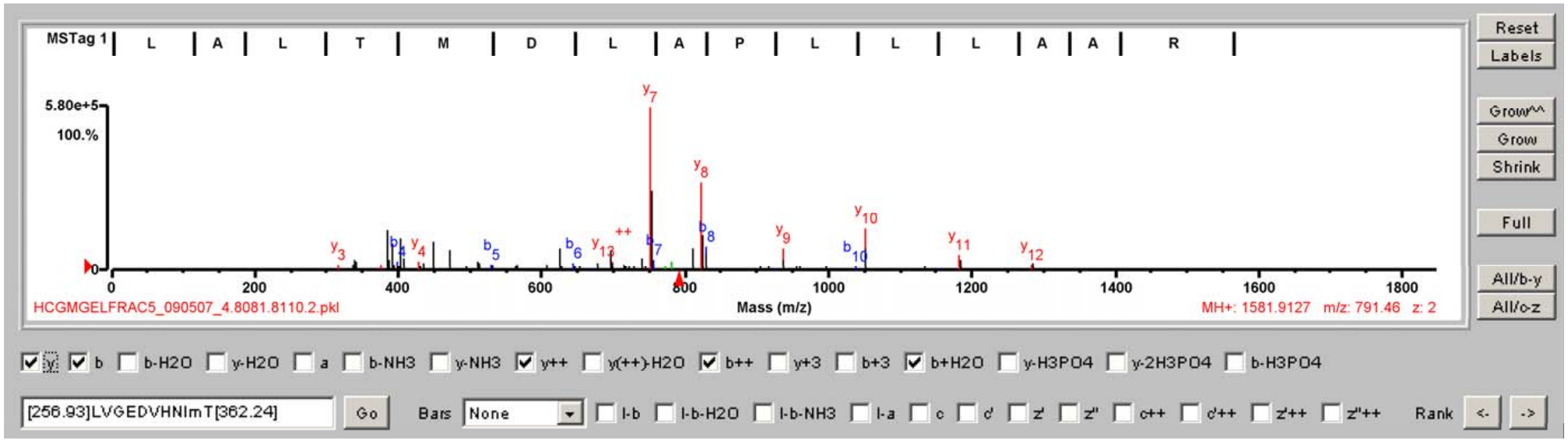

LOC55004 C11orf59

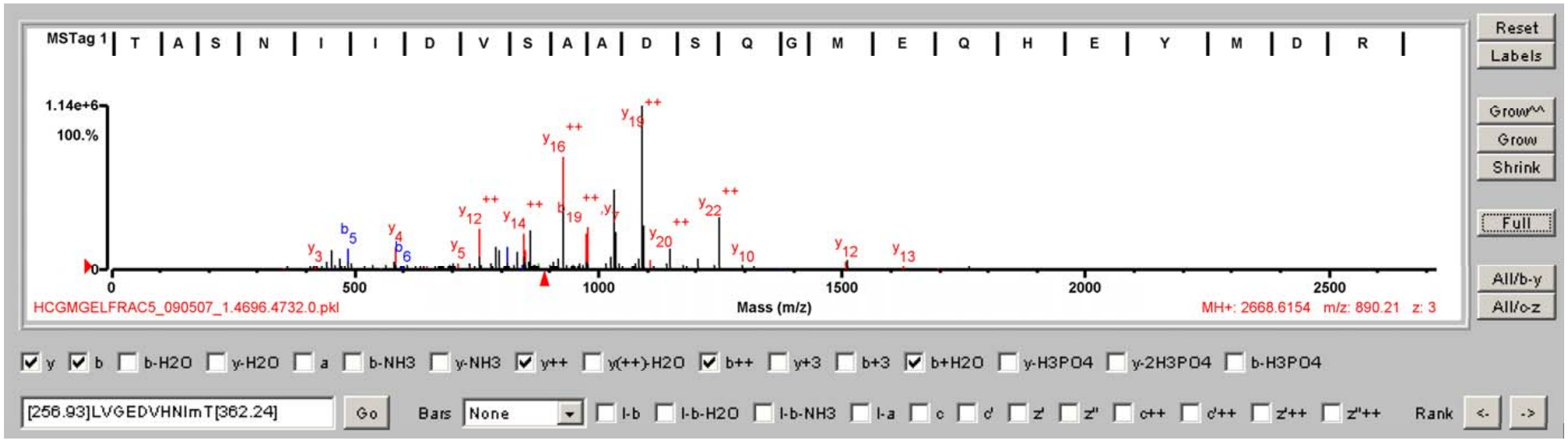

LOC56948 C14orf124

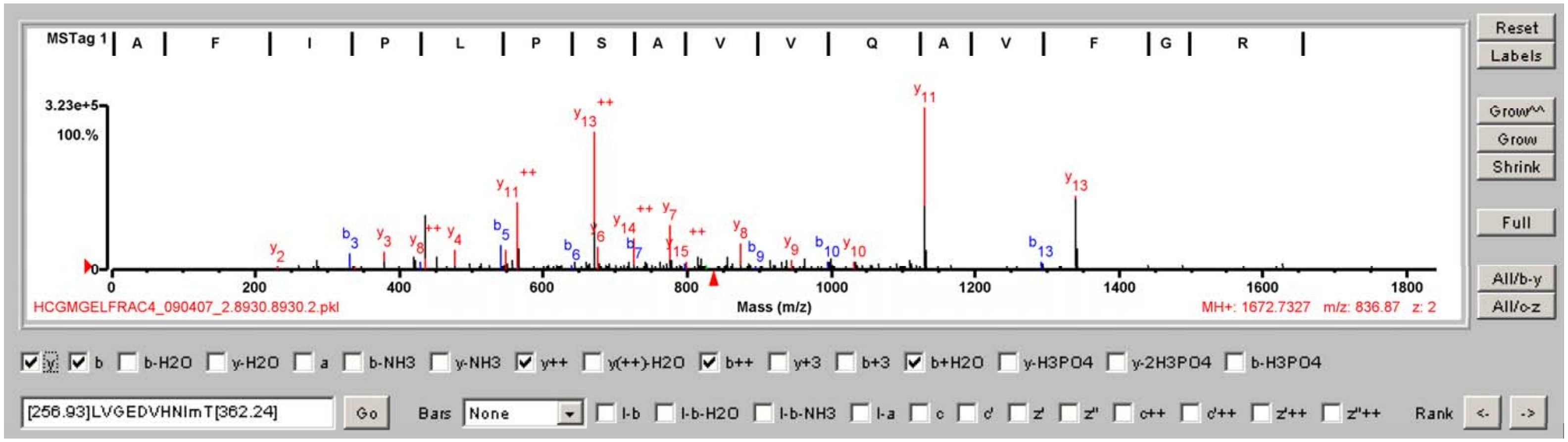

LOC84284 C1orf57

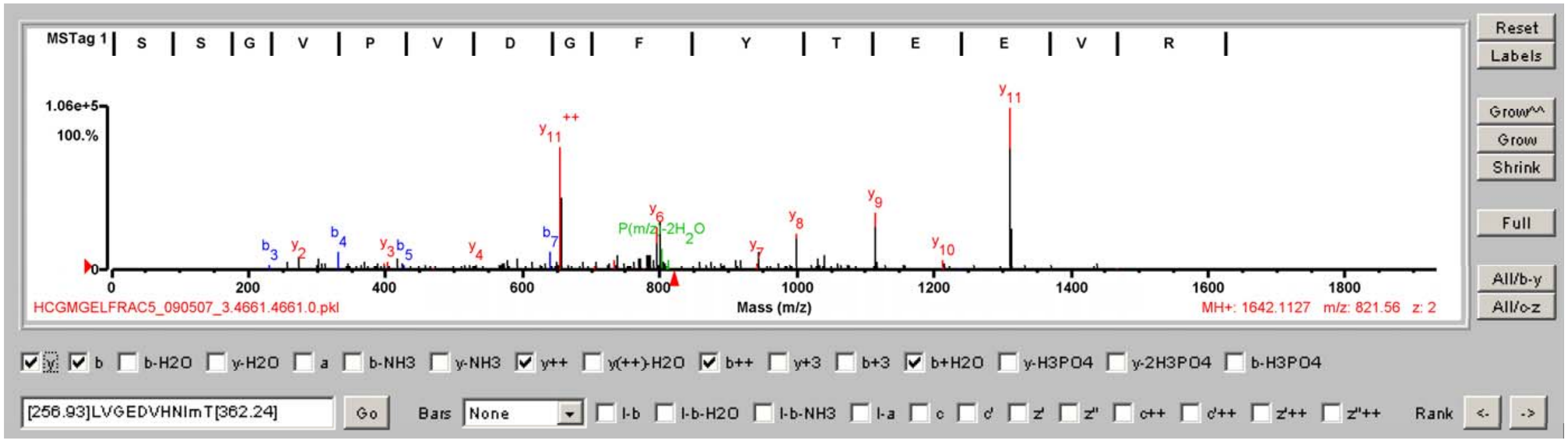

LOC9556 C14orf2

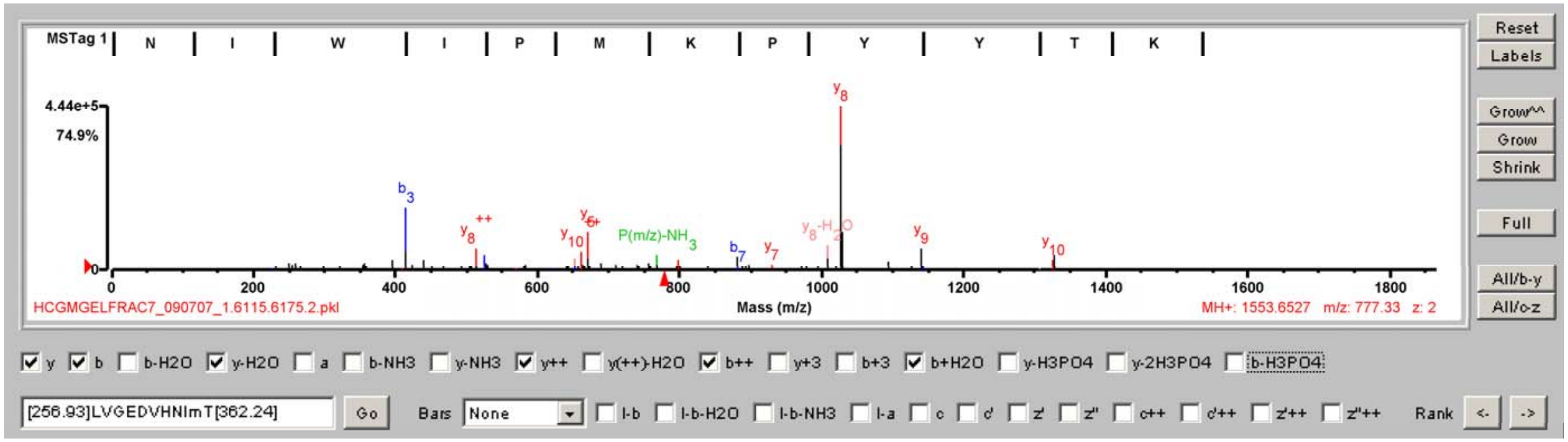

Inorganic Pyrophosphatase 2 PPA2

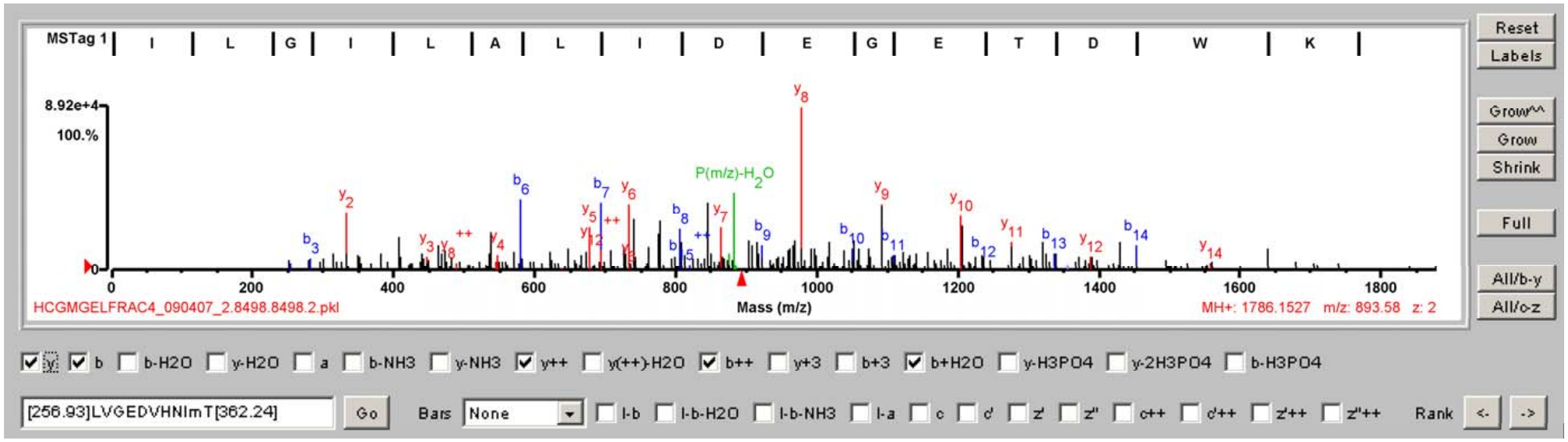

Keratin 2, 4, 76 KRT2, KRT4, KRT76

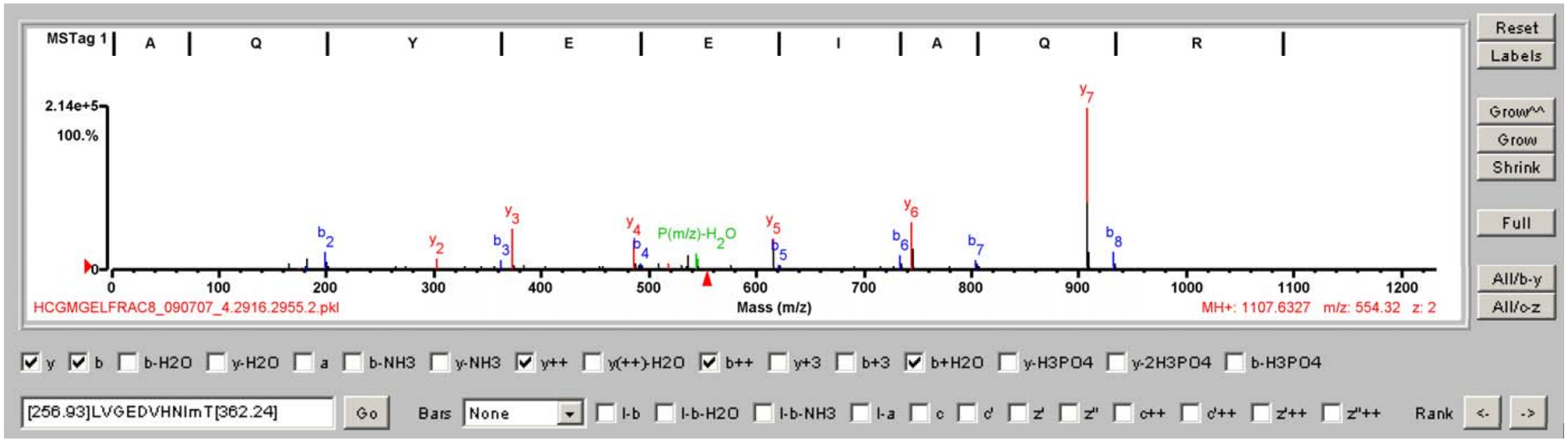

Keratin 25, 27, 28 KRT25, KRT27, KRT28

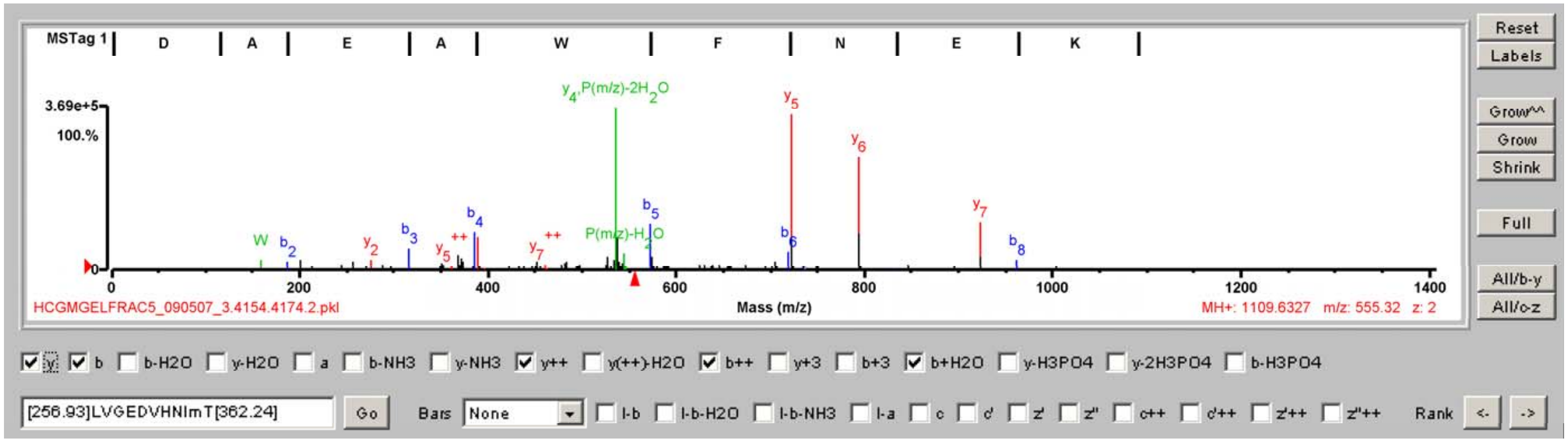

Keratin 10 KRT10

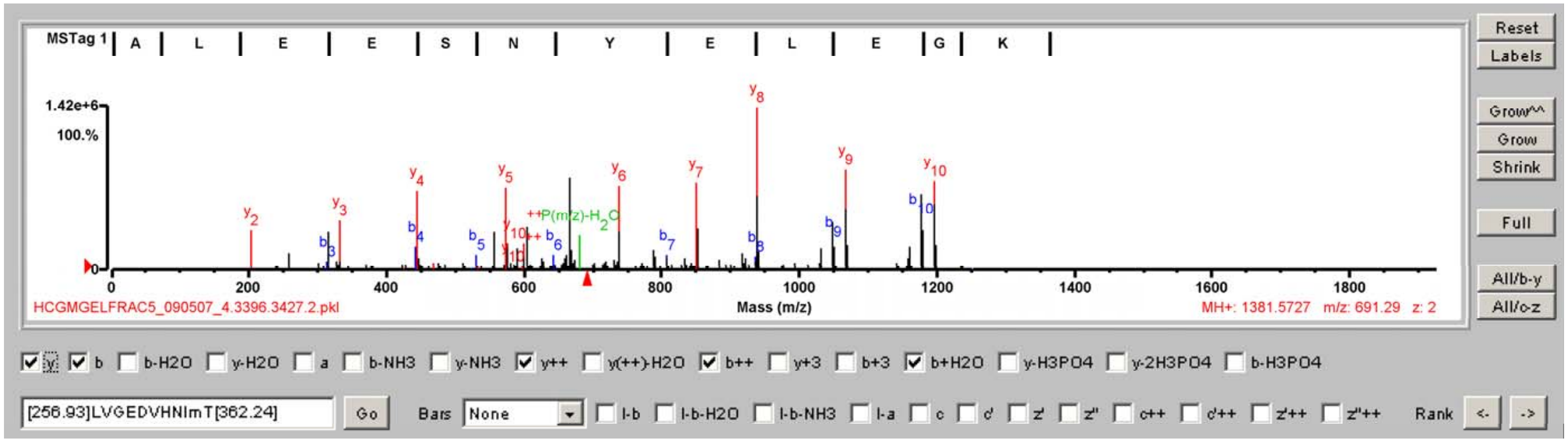

Keratin 77 KRT77

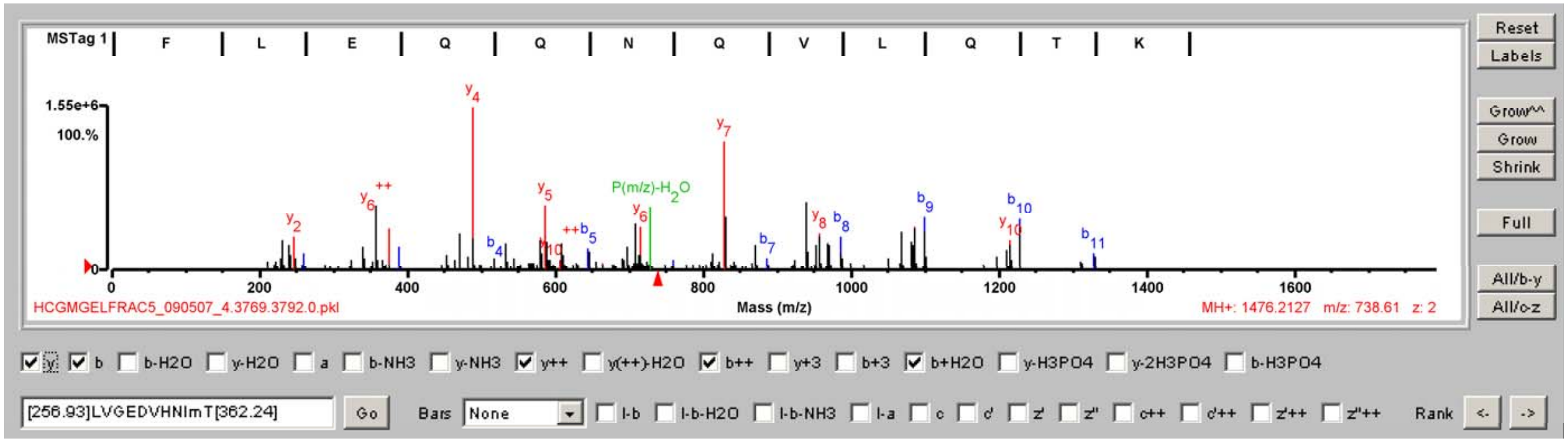

Lysosomal-Associated Membrane Protein 1 LAMP1

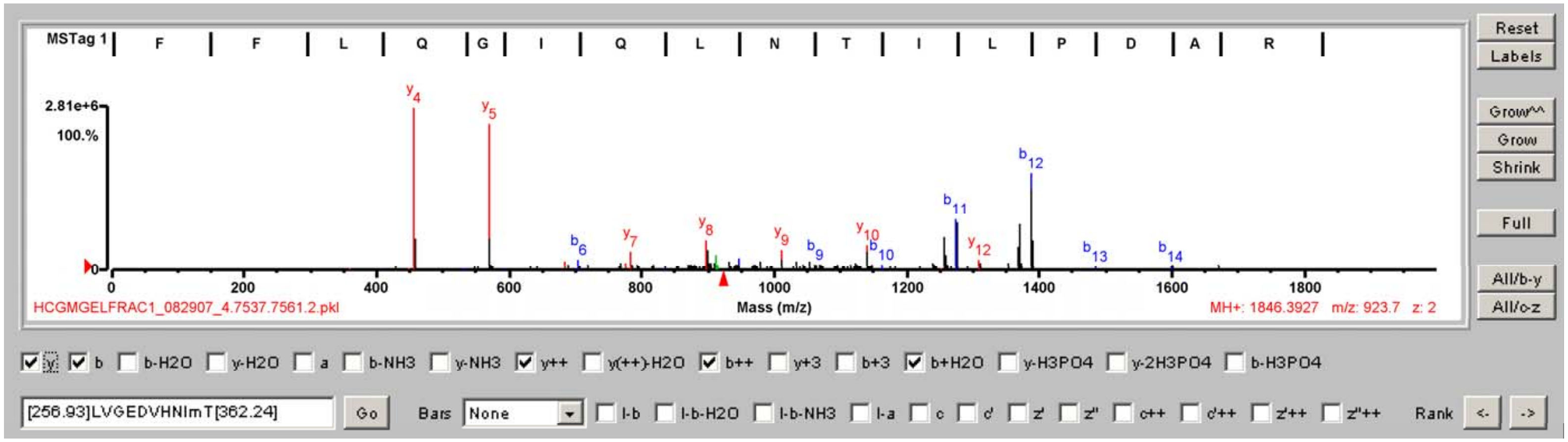

Major Histocompatibility Complex, Class 1 HLA-E, HLA-G

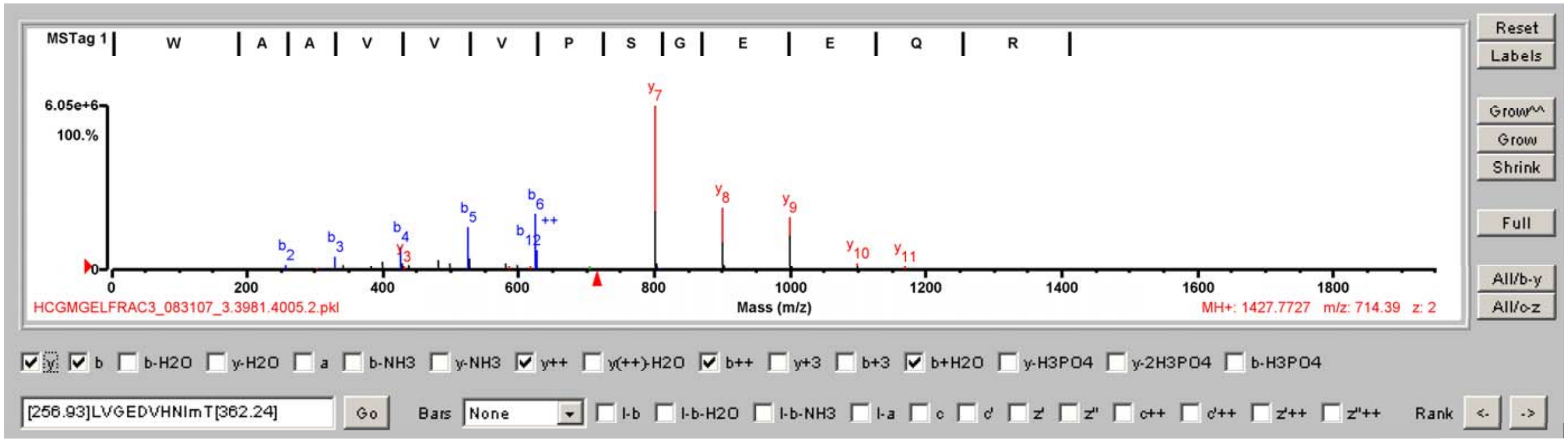

Microtubule Associated Protein 1A, 1B MAP1A, MAP1B

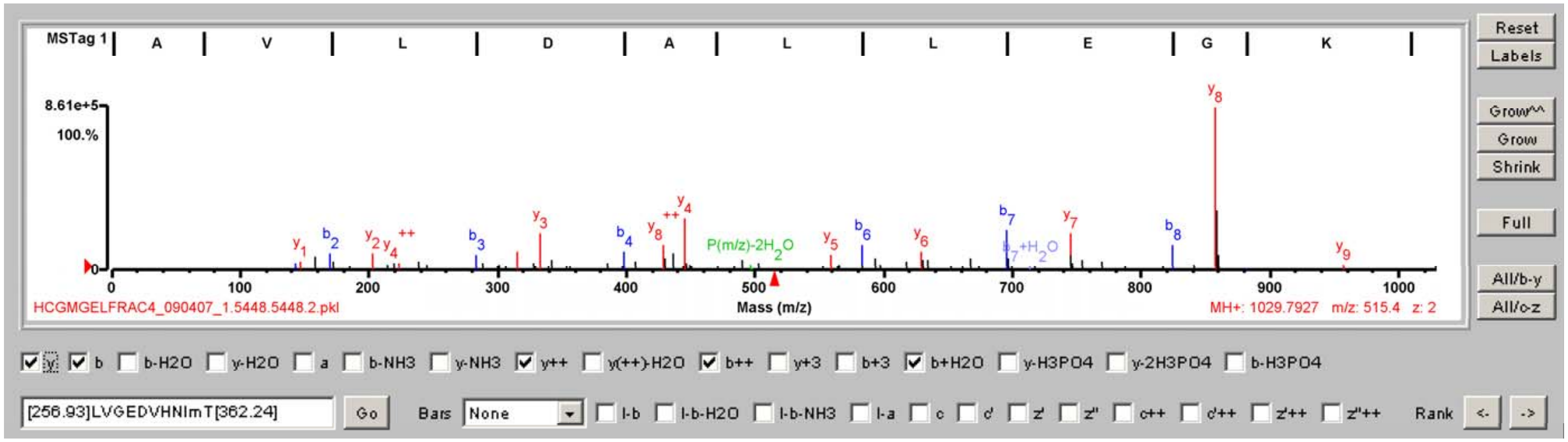

Mitogen-Activated Protein Kinase Interacting Protein MAPKSP1

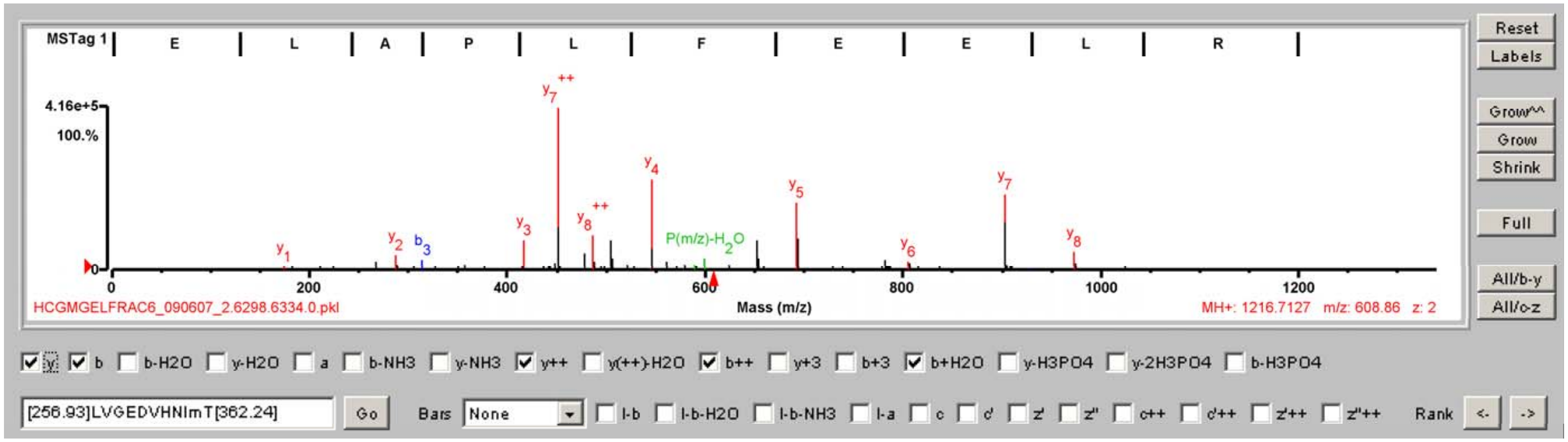

Myofibrillogenesis Regulator 1, Isoform 1, 3 PNKD

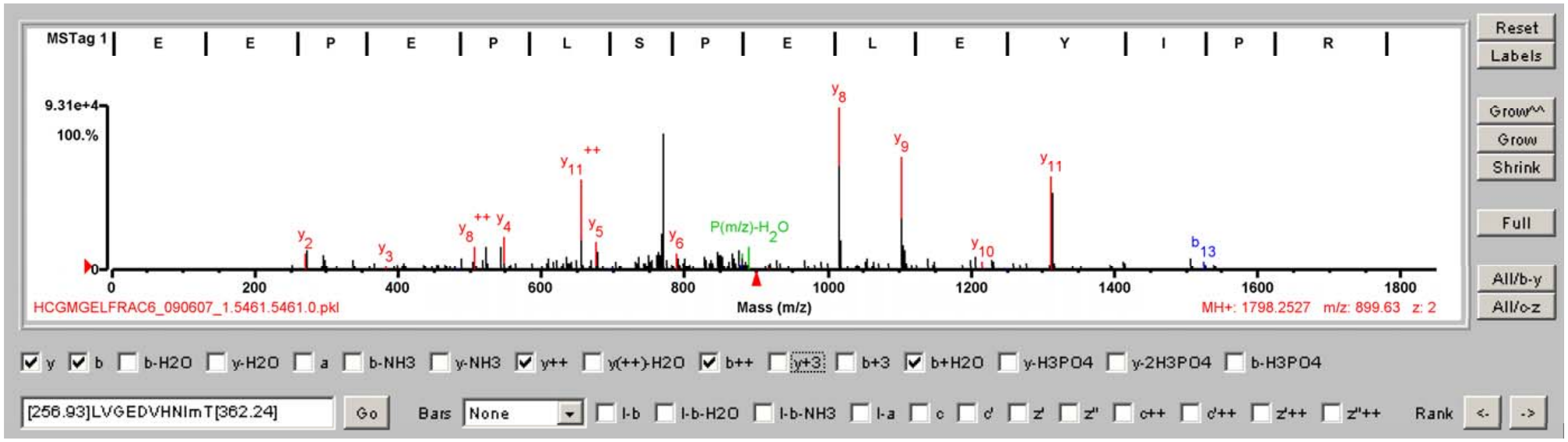

NADH Dehydrogenase 1 Alpha 10 NDUFA10

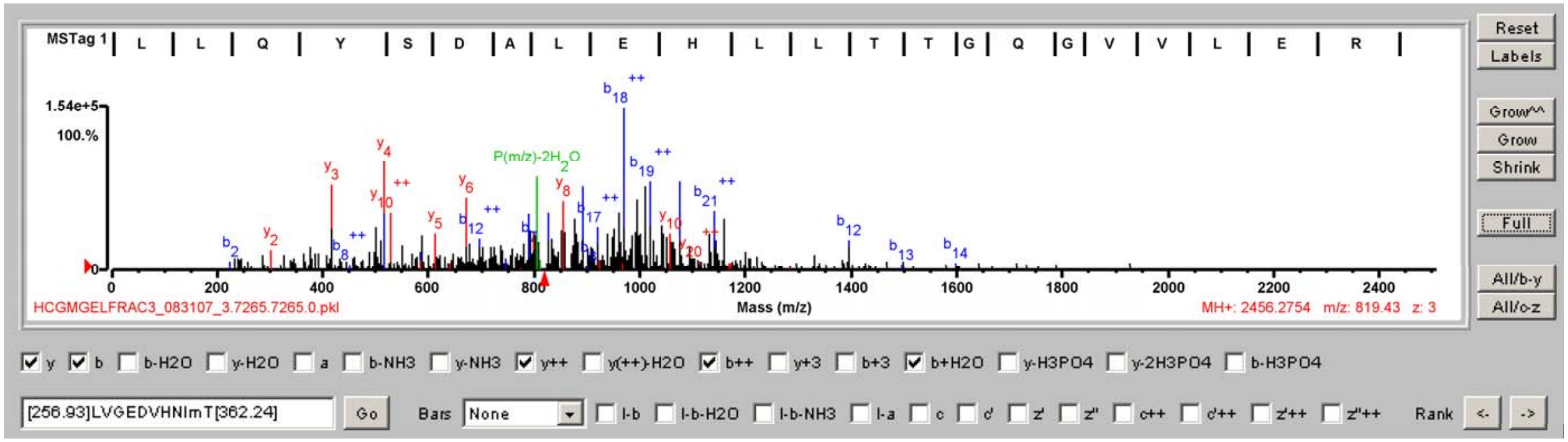

NADH Dehydrogenase 1 Alpha 11 NDUFA11

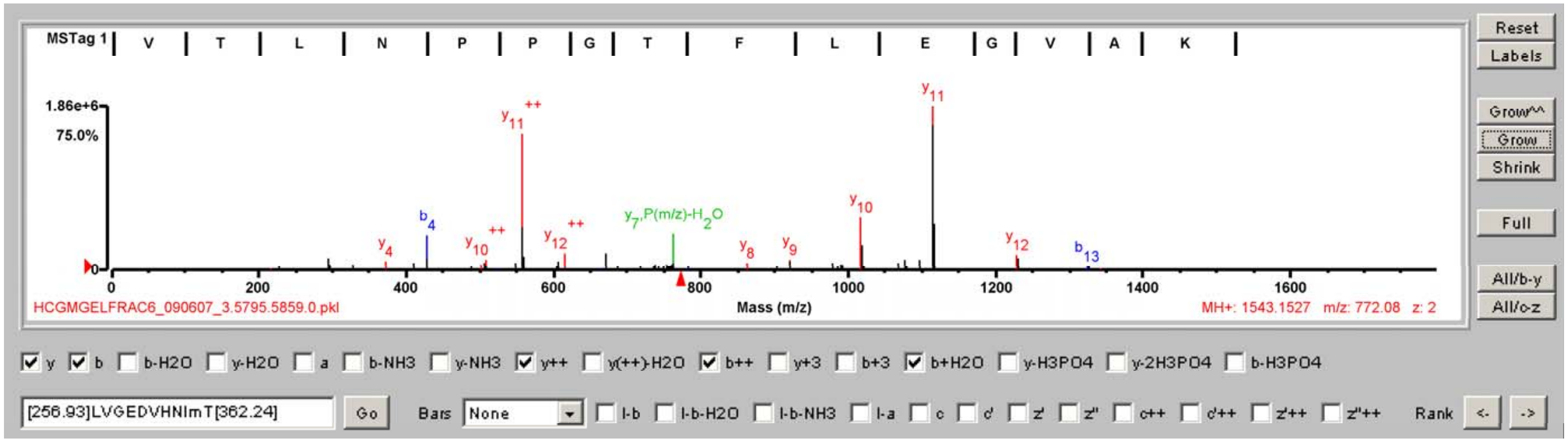

NADH Dehydrogenase 1 Alpha 5 NDUFA5

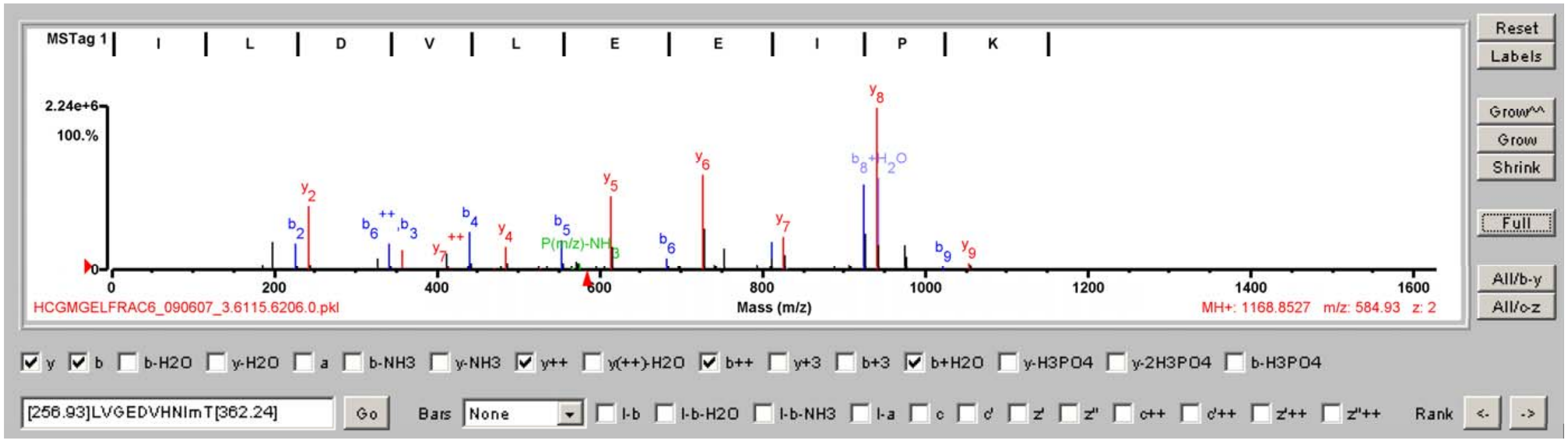

NADH Dehydrogenase 1 Beta 2 NDUFB2

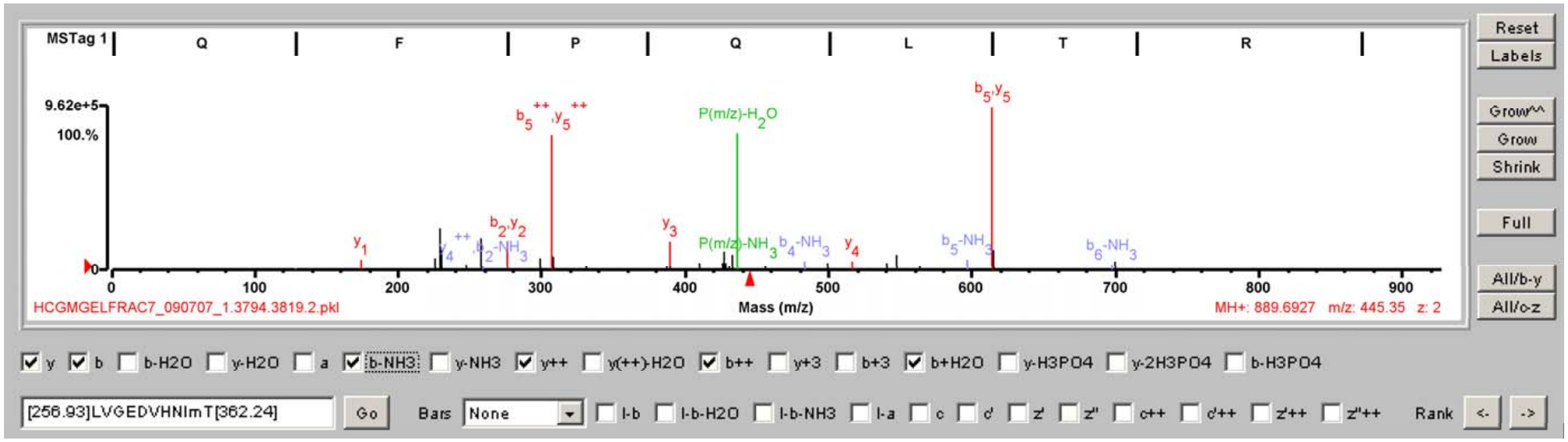

NADH Dehydrogenase 1 Beta 5 NDUFB5

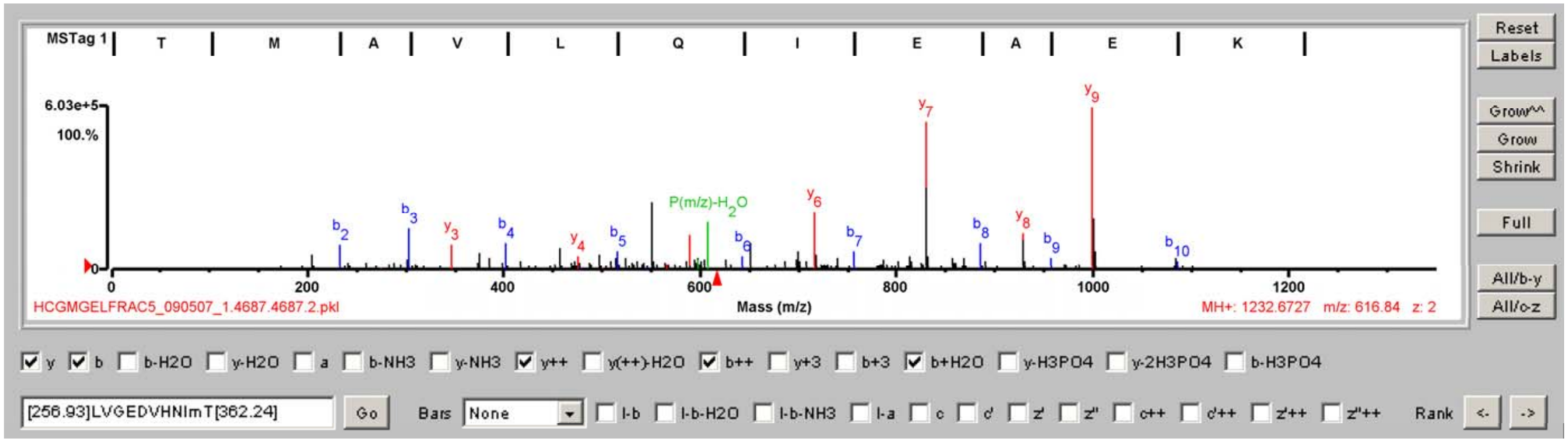

NADH Dehydrogenase 1 Alpha/Bets Subcomplex 1 NDUFAB1

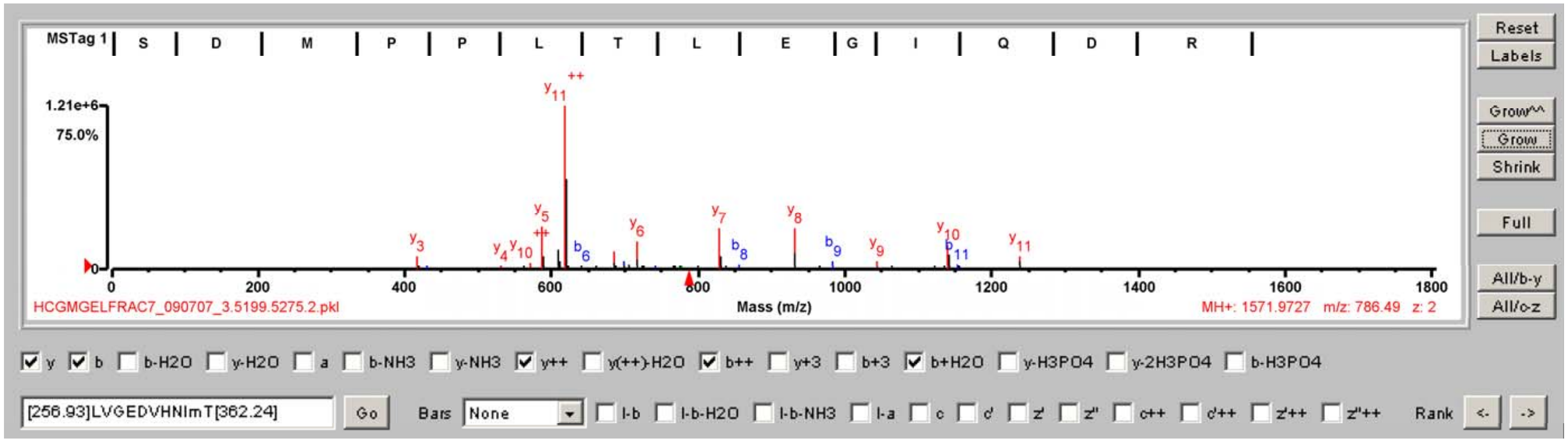

NADH Dehydrogenase Fe-S Protein 6 NDUFS6

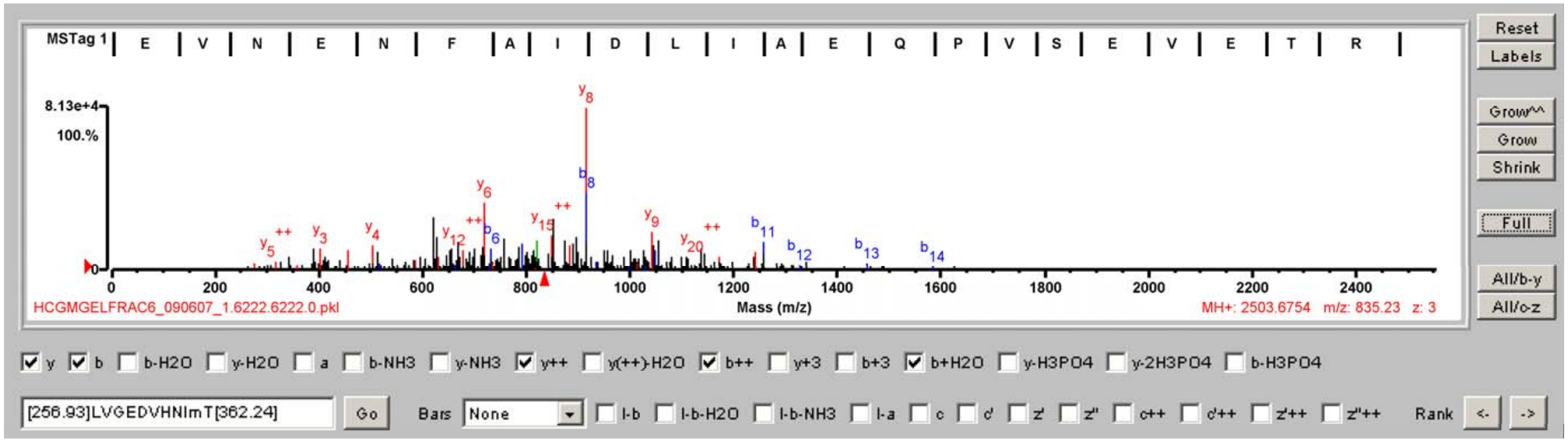

[illegible]

NADH Dehydrogenase Fe-S Protein 8 NDUFS8

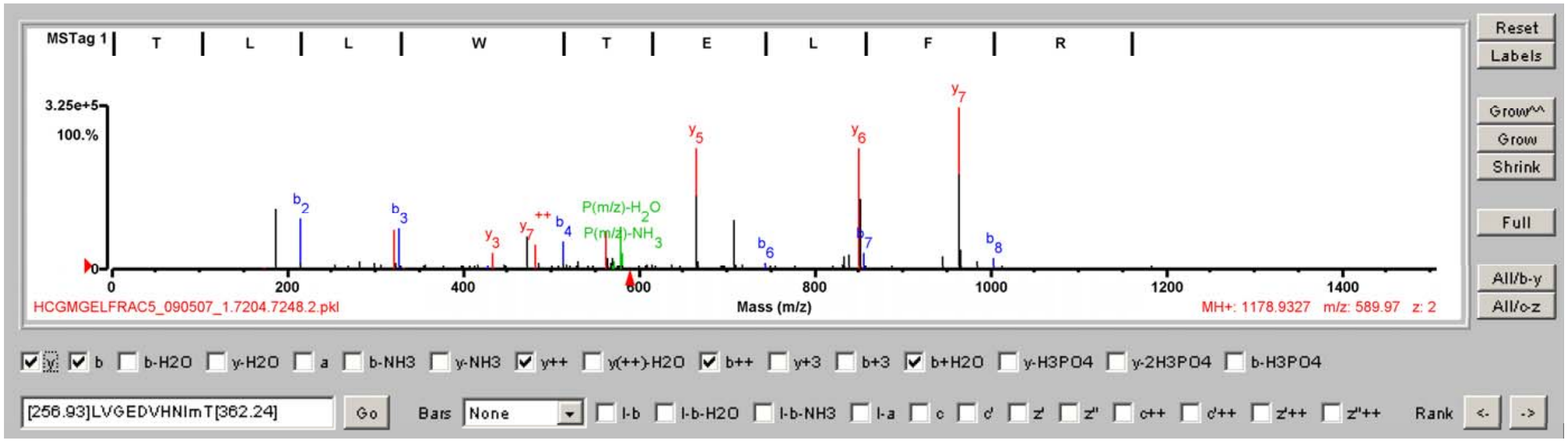

NADH Dehydrogenase Subunit 4 MT-ND4

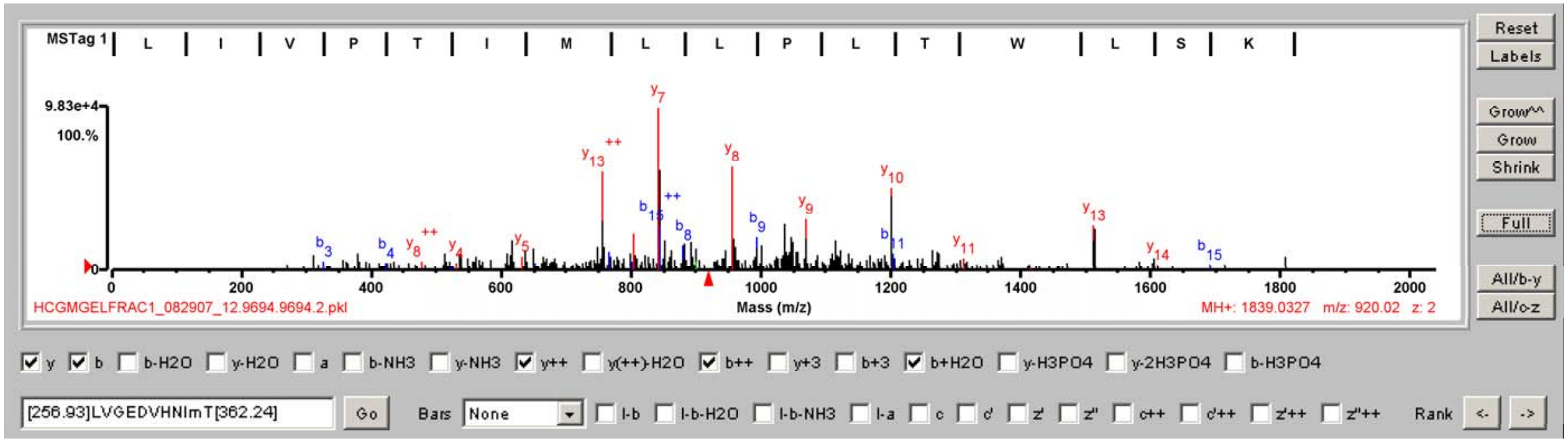

Neurofilament Heavy, Light, Medium Polypeptide NEFH, NEFL, NEFM

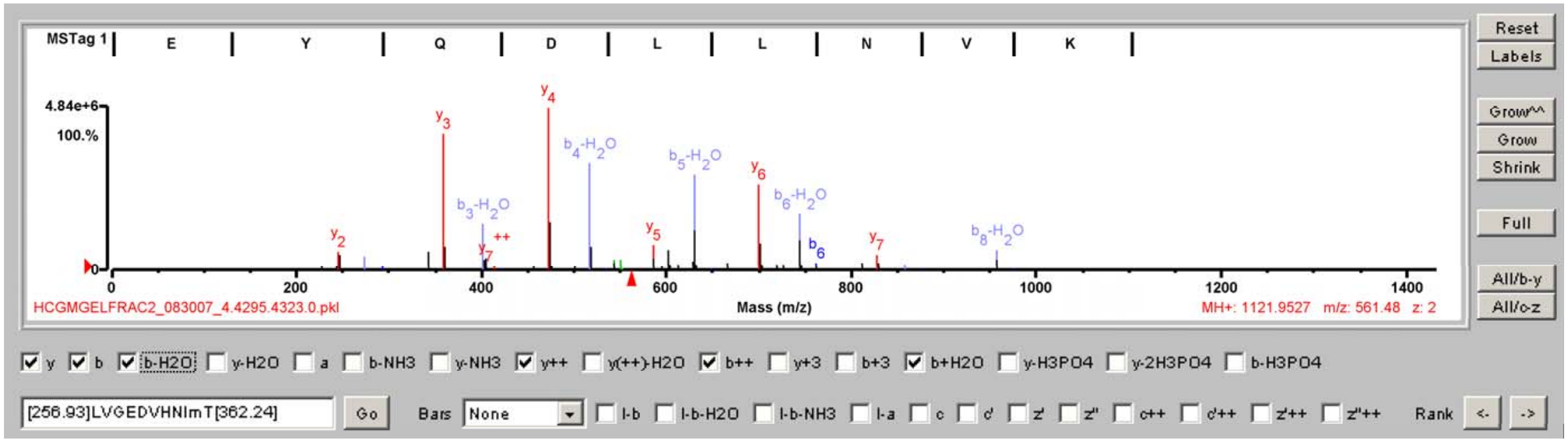

Neuropeptide Y NPY

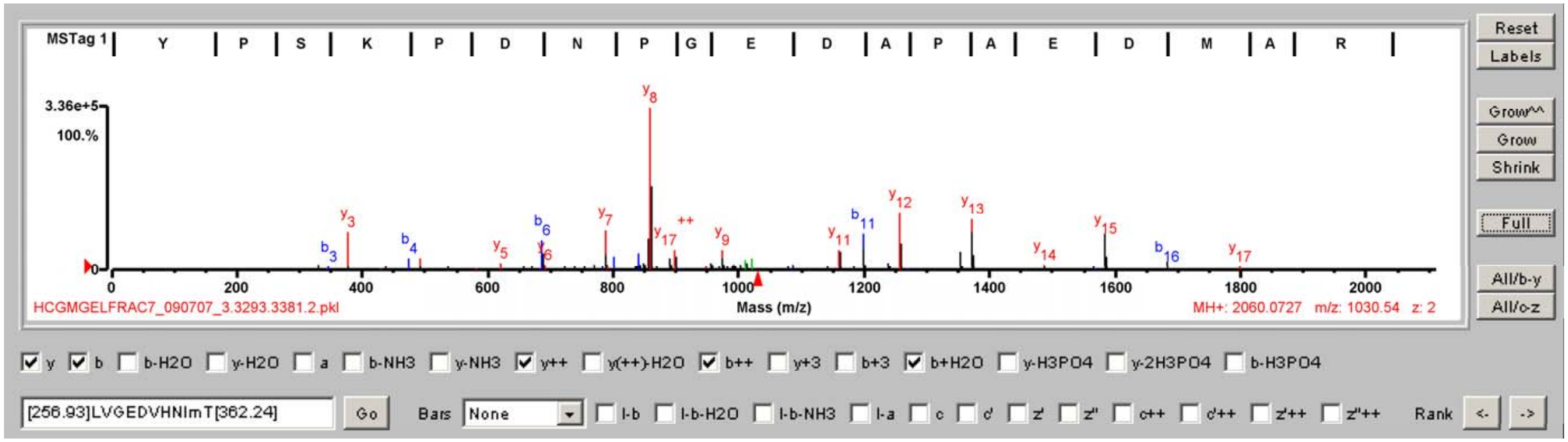

Nicastrin Precursor NCSTN

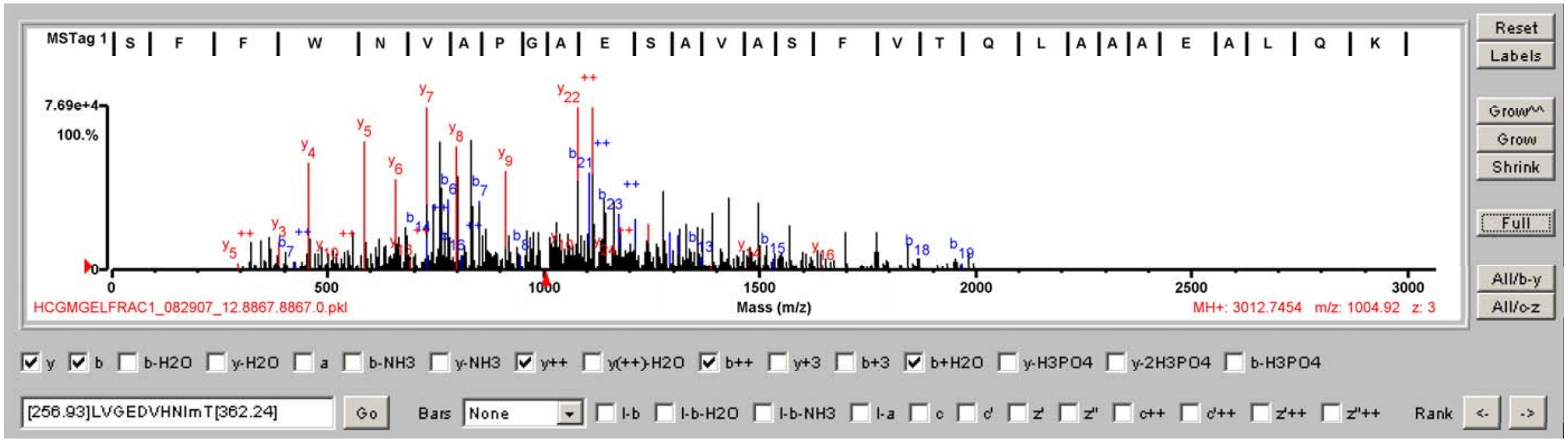

Peptidylprolyl Isomerase A PPIAL4G

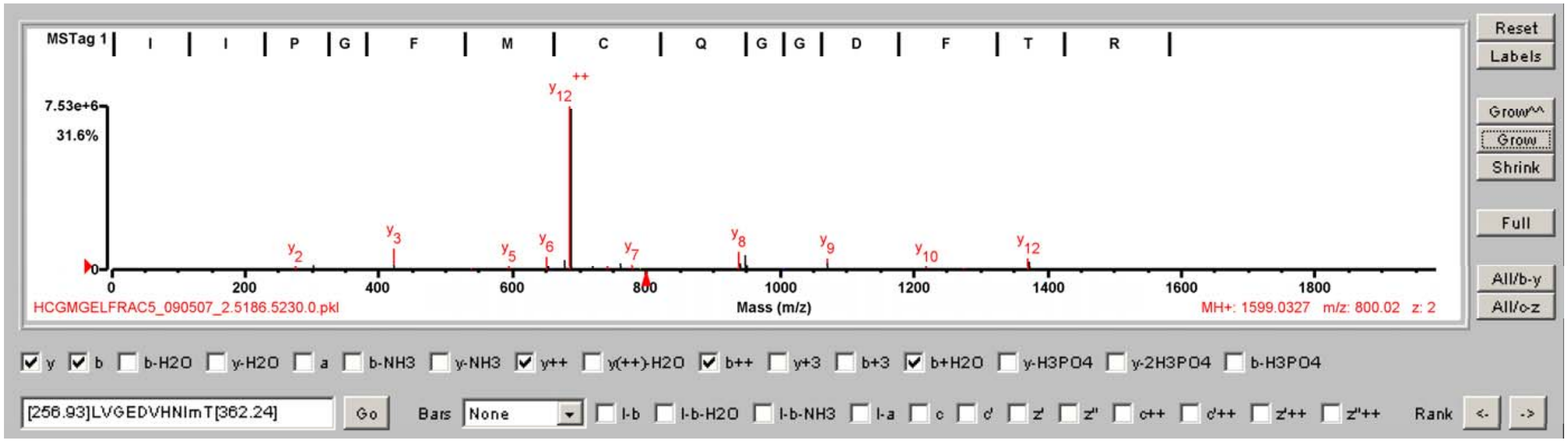

PKCI-1 Related HIT Protein HINT2

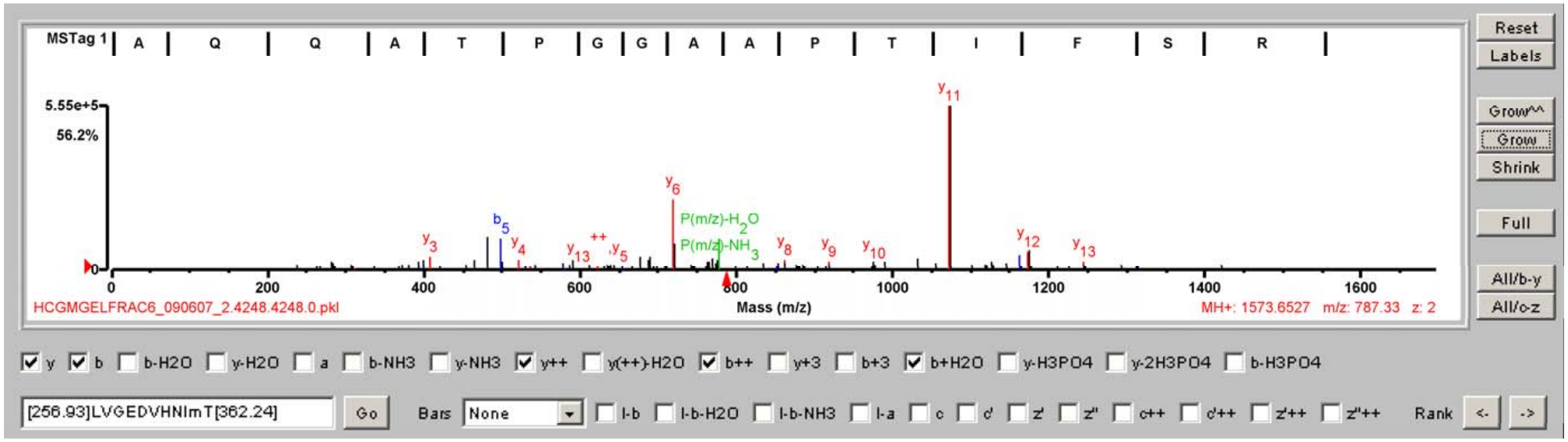

Pleiotrophin PTN

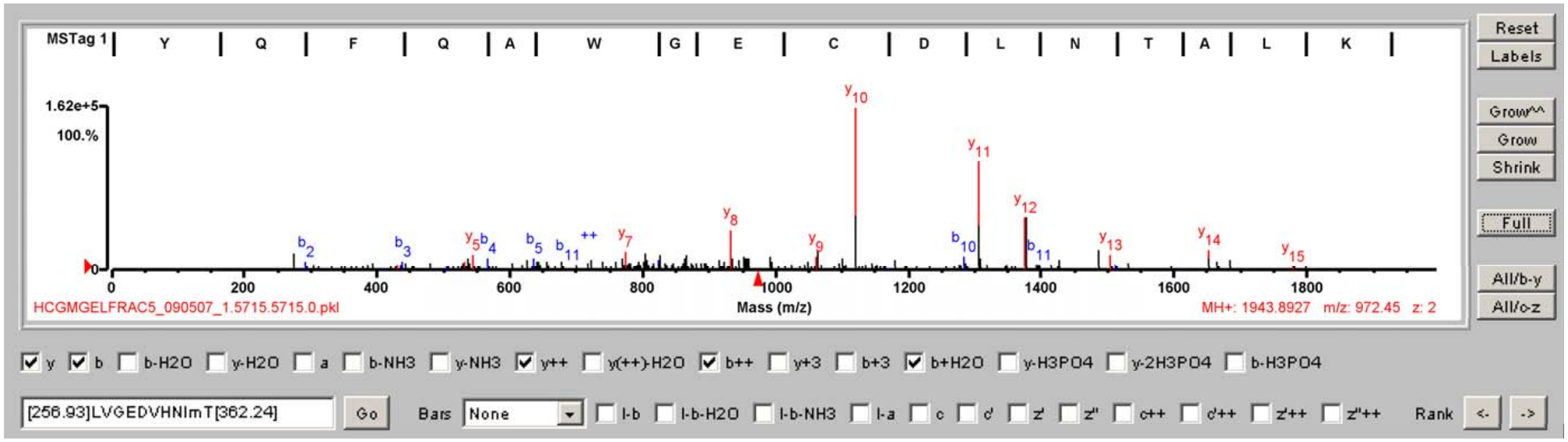

Similar to CG17807-PA

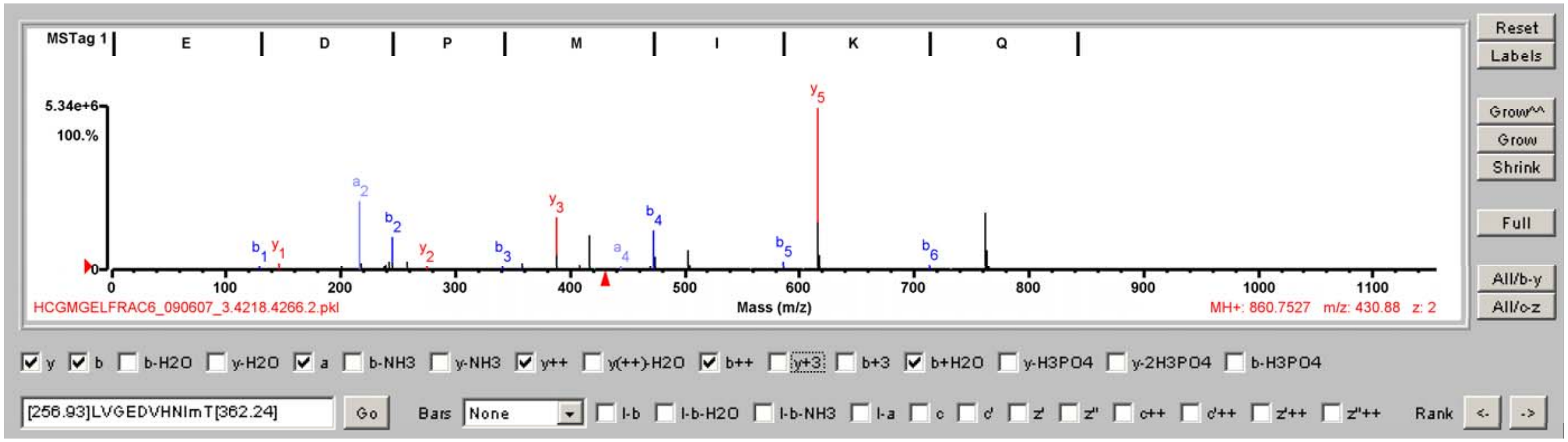

Similar to NADH Dehydrogenase MT-ND4

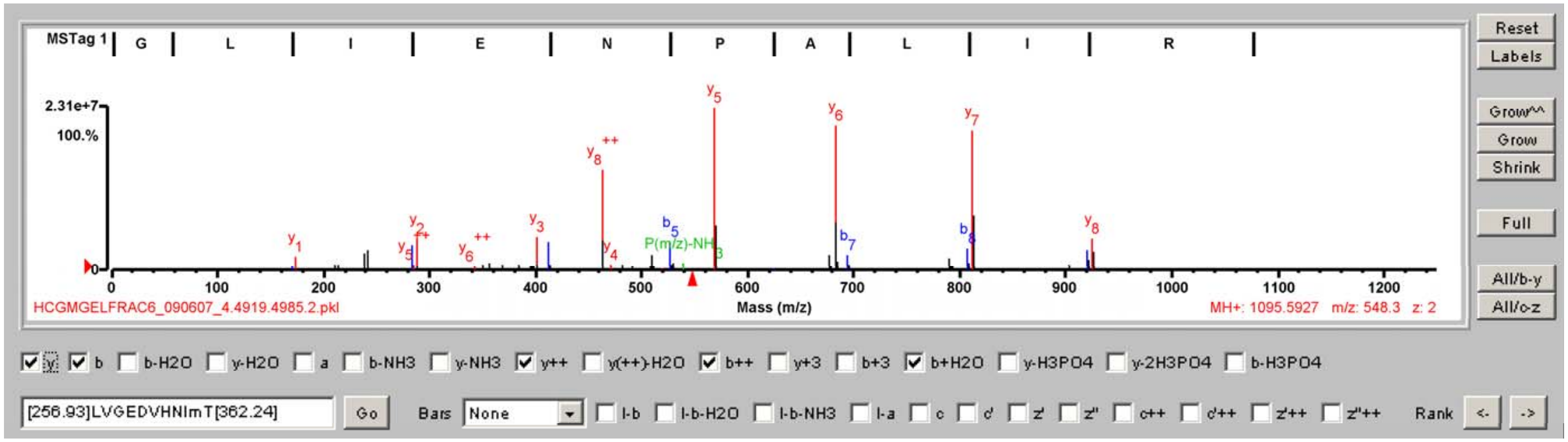

Prion Protein Preproprotein PRNP

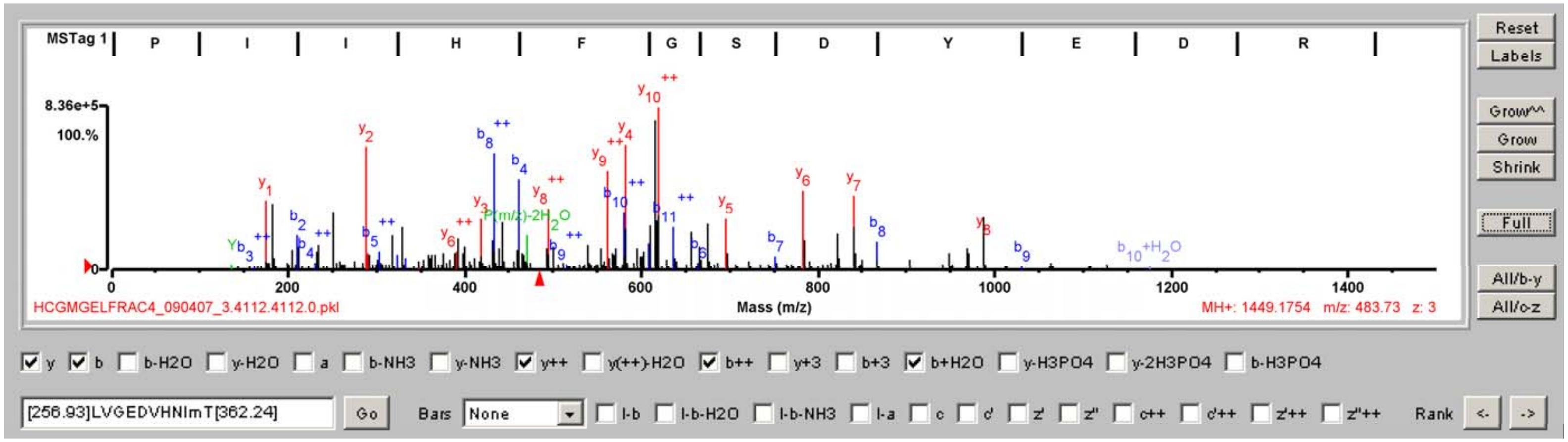

Quiescin Q6 Sulfhydryl Oxidase 1 QSOX1

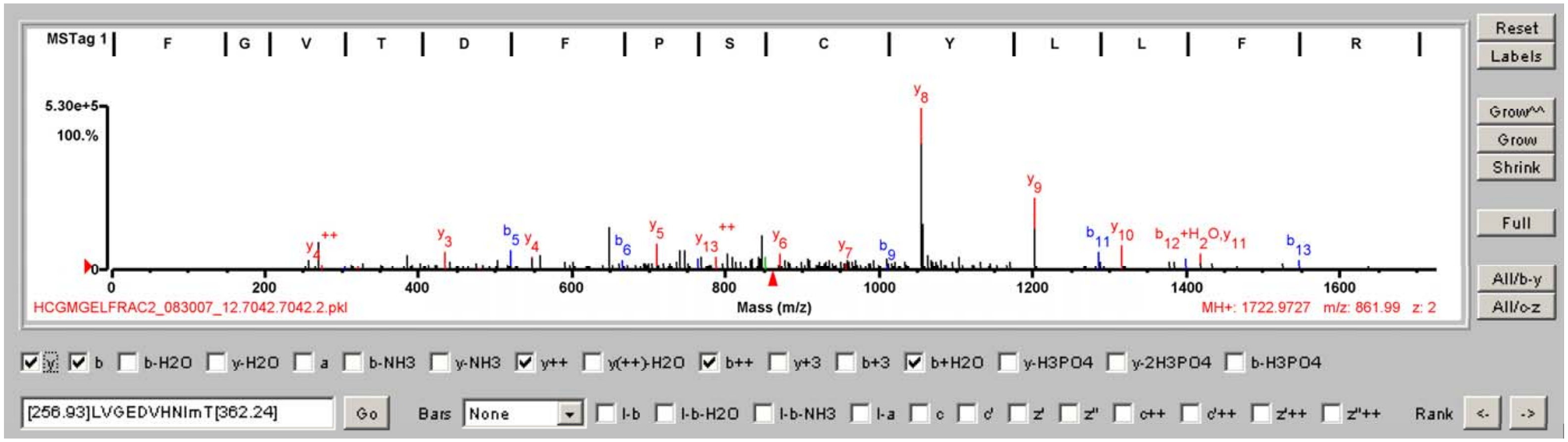

Ras-Related GTP Binding Protein 4b, 10, 34 RAB4B, RAB10 RAB34

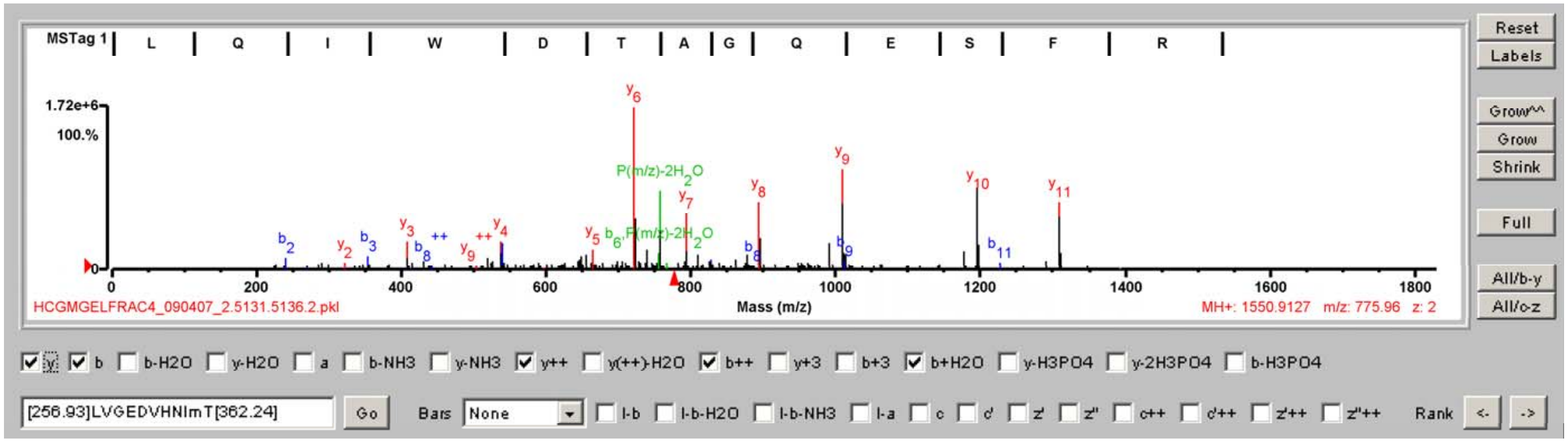

Ras Related Protein RAB-33A RAB33A

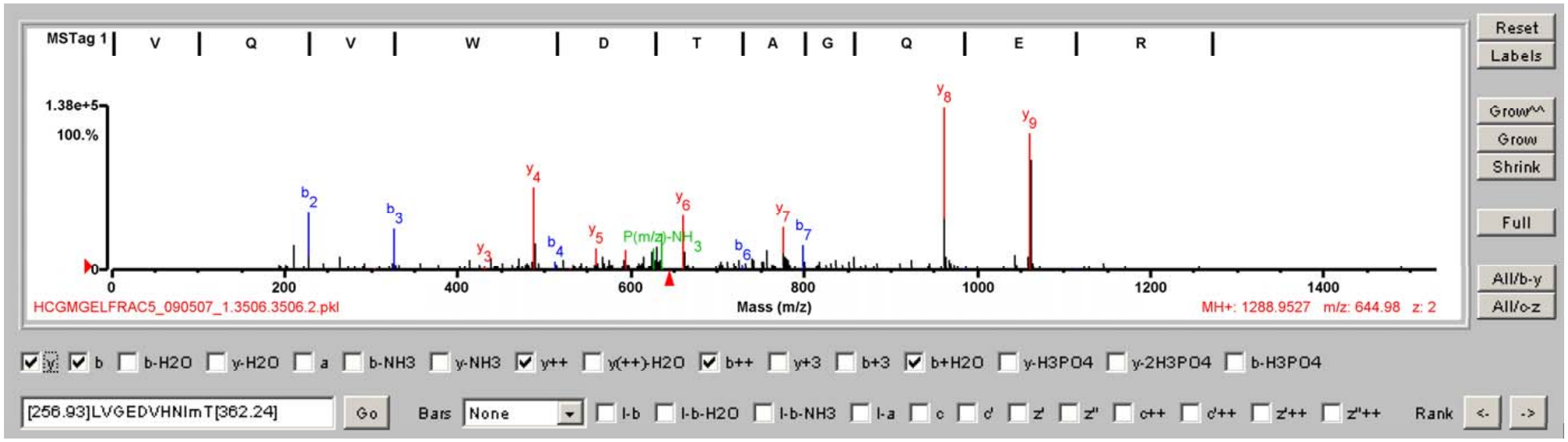

Retbindin Isoform 2 RTBDN

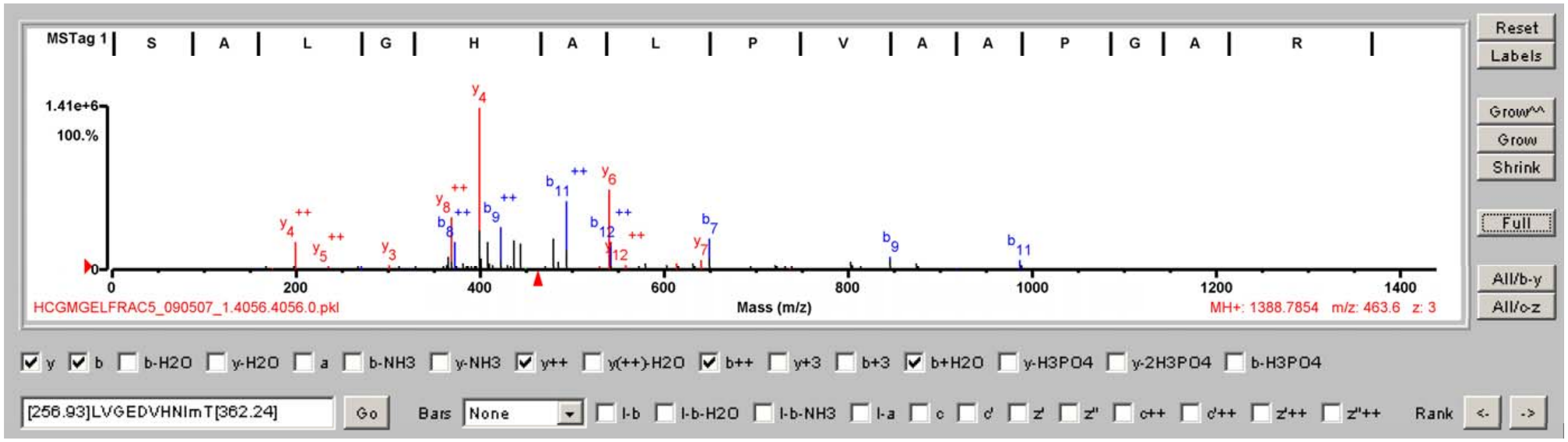

Scavenger Receptor Class B, Member 2 SCARB2

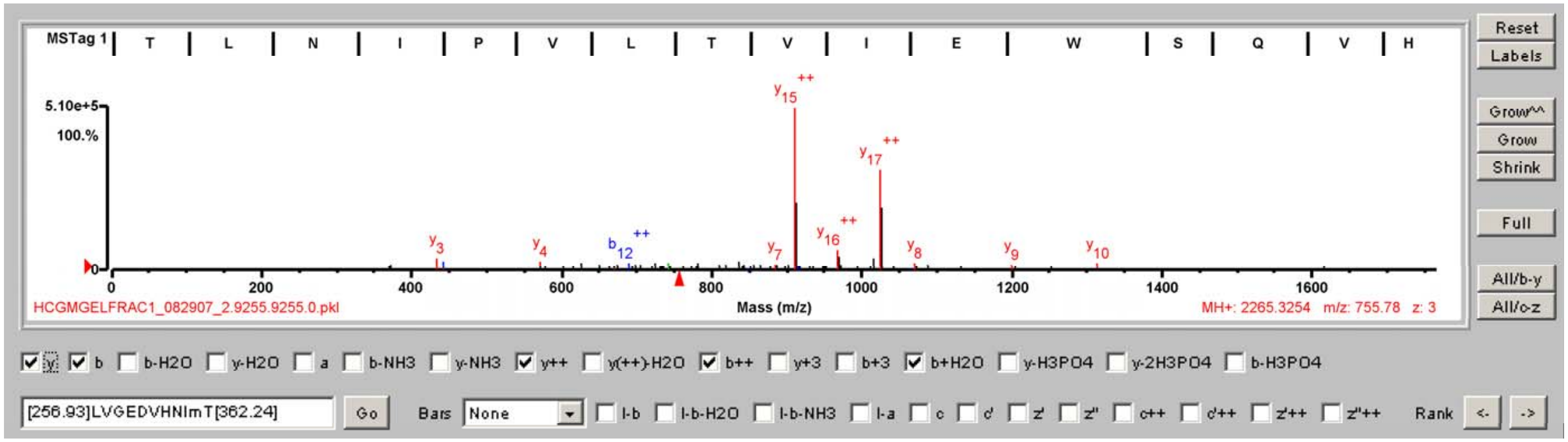

Sec61 Beta Subunit SEC61B

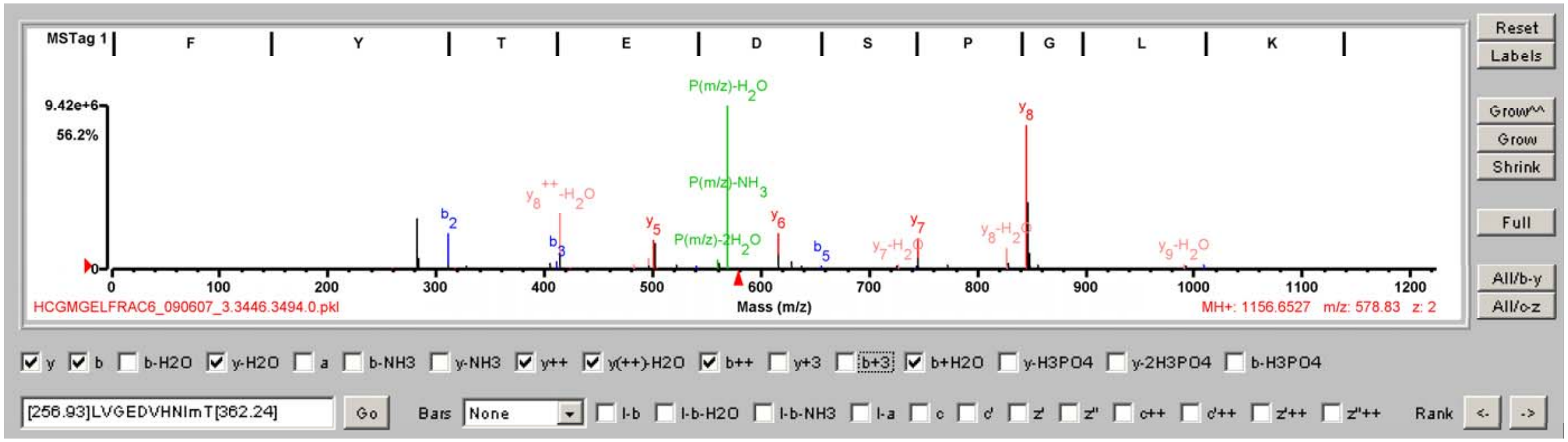

Secretory Carrier Membrane Protein 3 SCAMP3

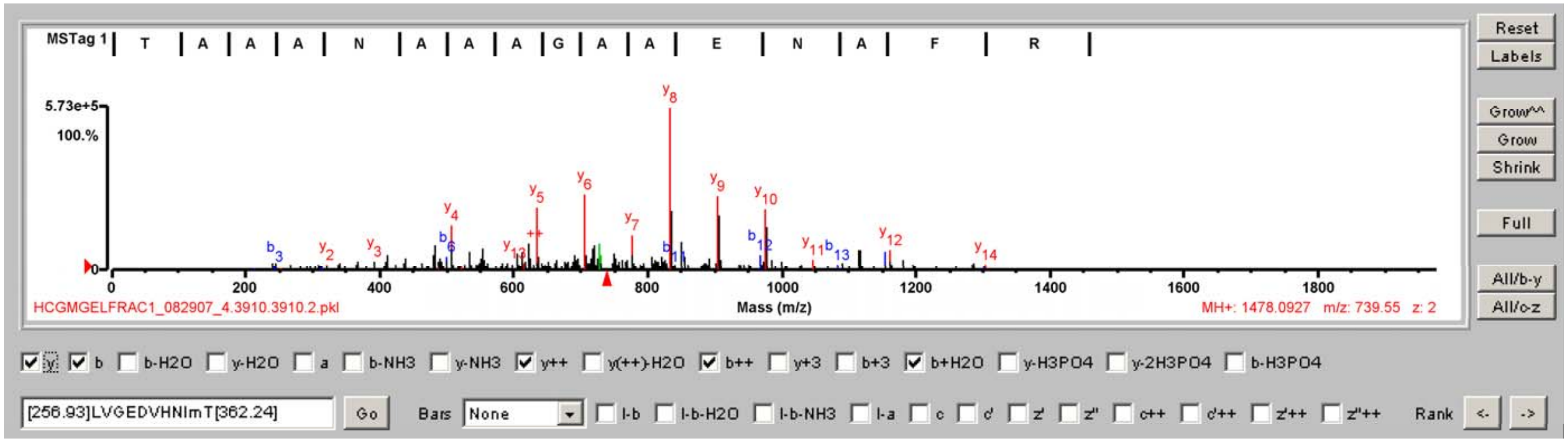

Serine (Cysteine) Proteinase Inhibitor A SERPINA1

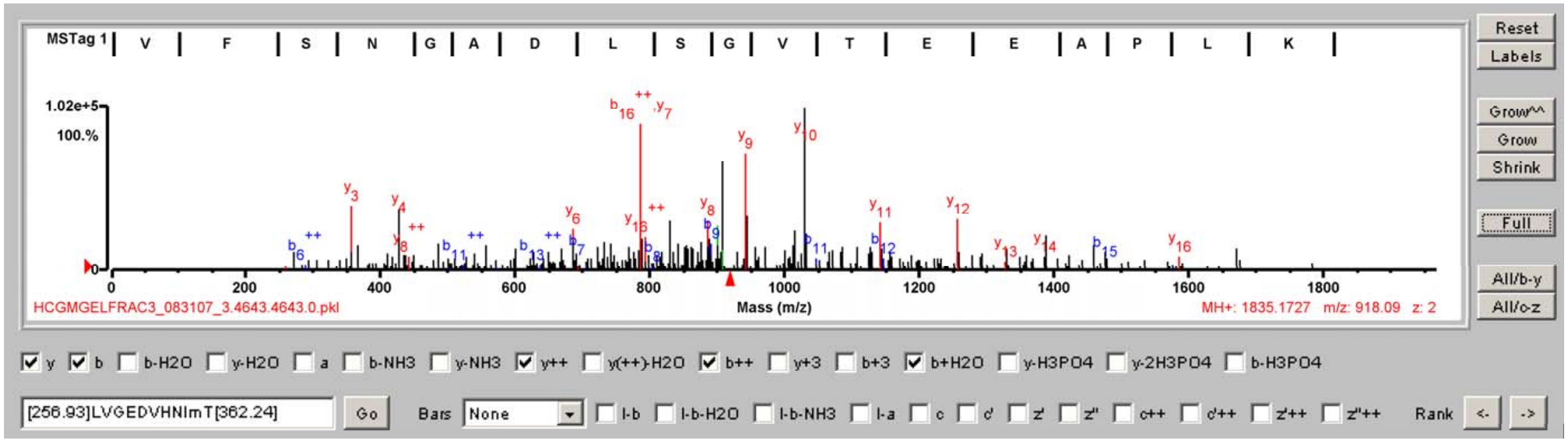

Serine (Cysteine) Proteinase Inhibitor H SERPINH1

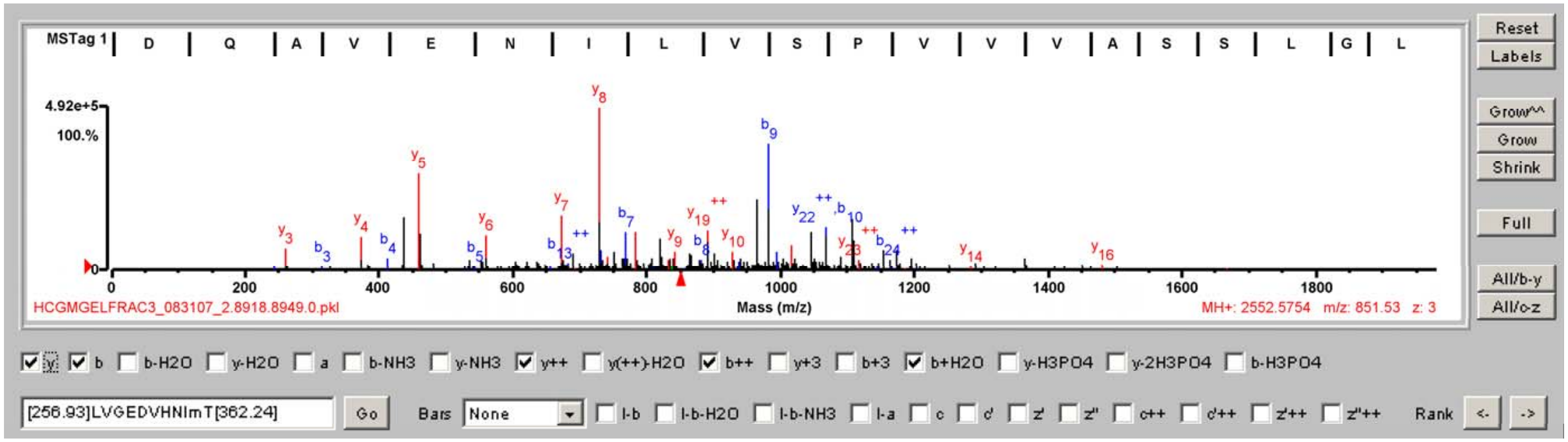

Serine (Cysteine) Proteinase Inhibitor SERPINB12

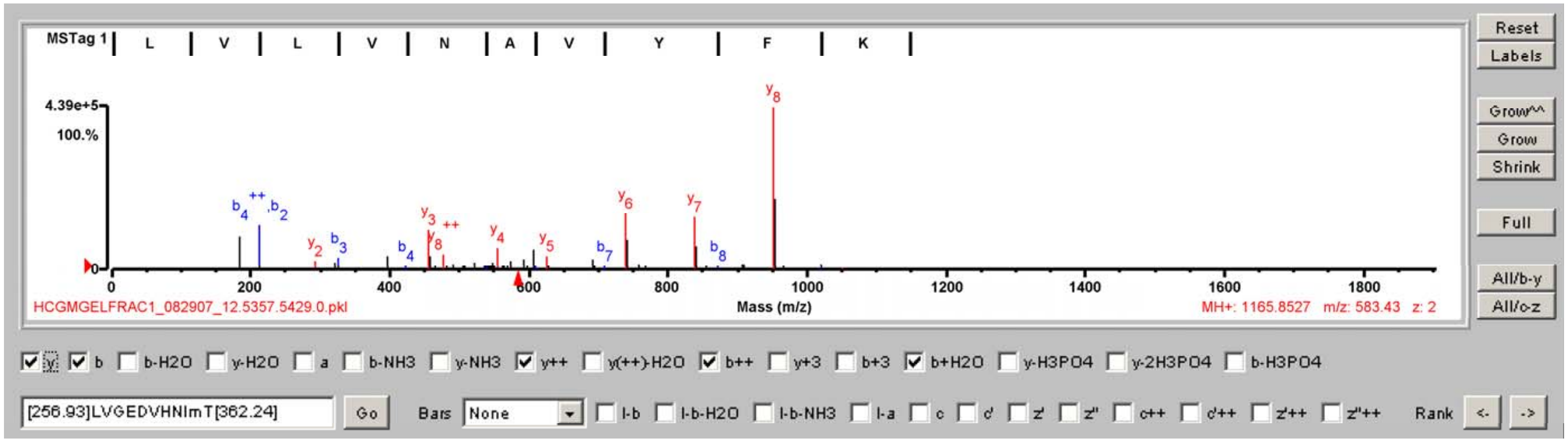

Signal Sequence Receptor Delta SSR4

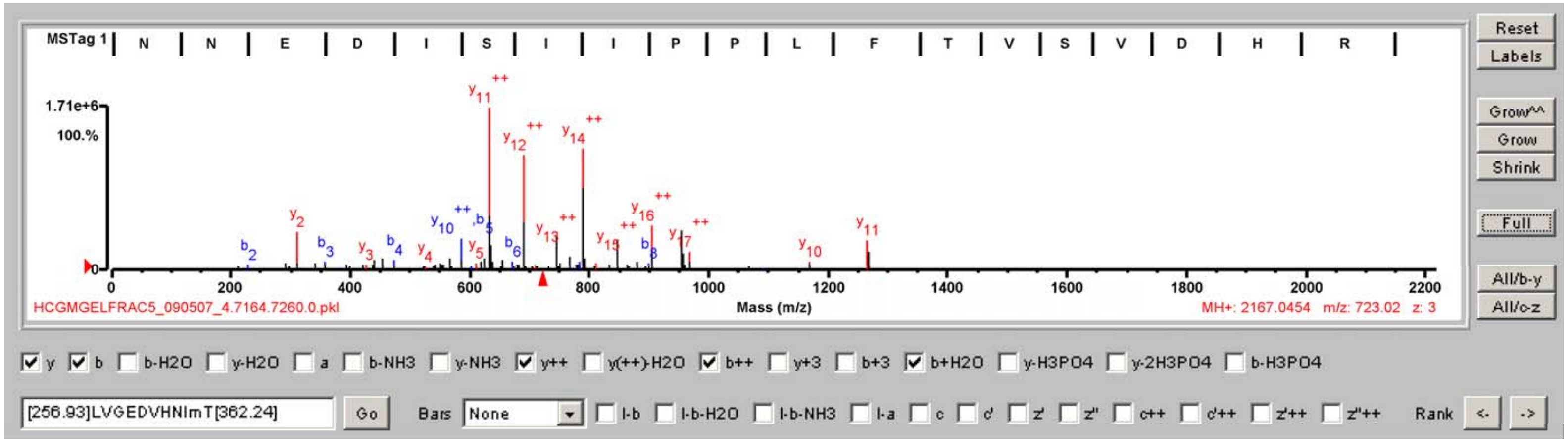

Smooth Muscle and Non-Muscle Myosin Light Chain Beta 6 MYL6B

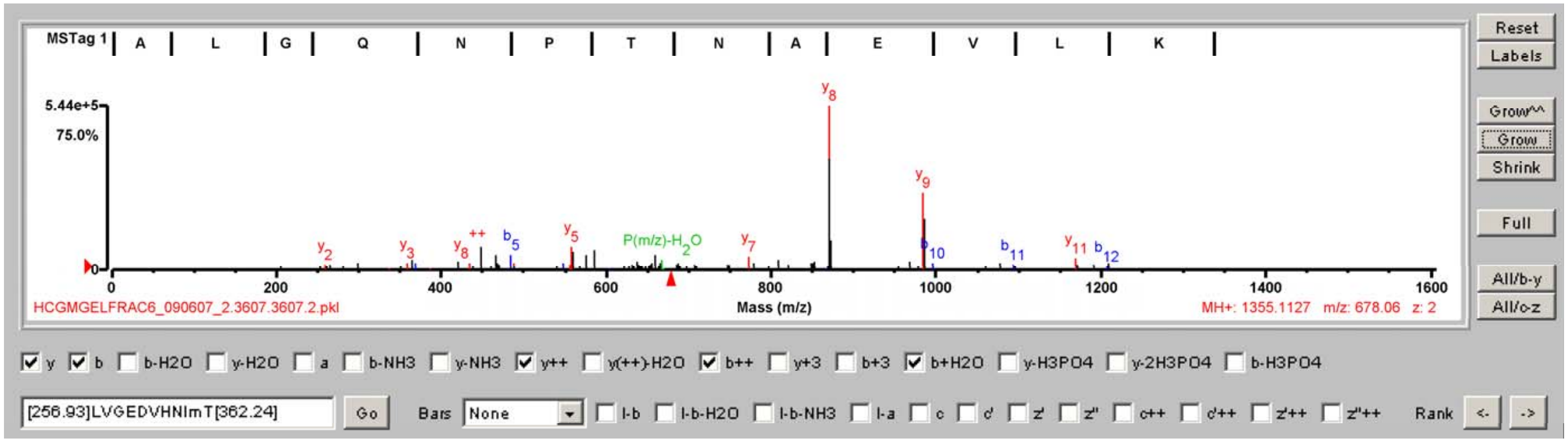

Stathmin 1 STMN1

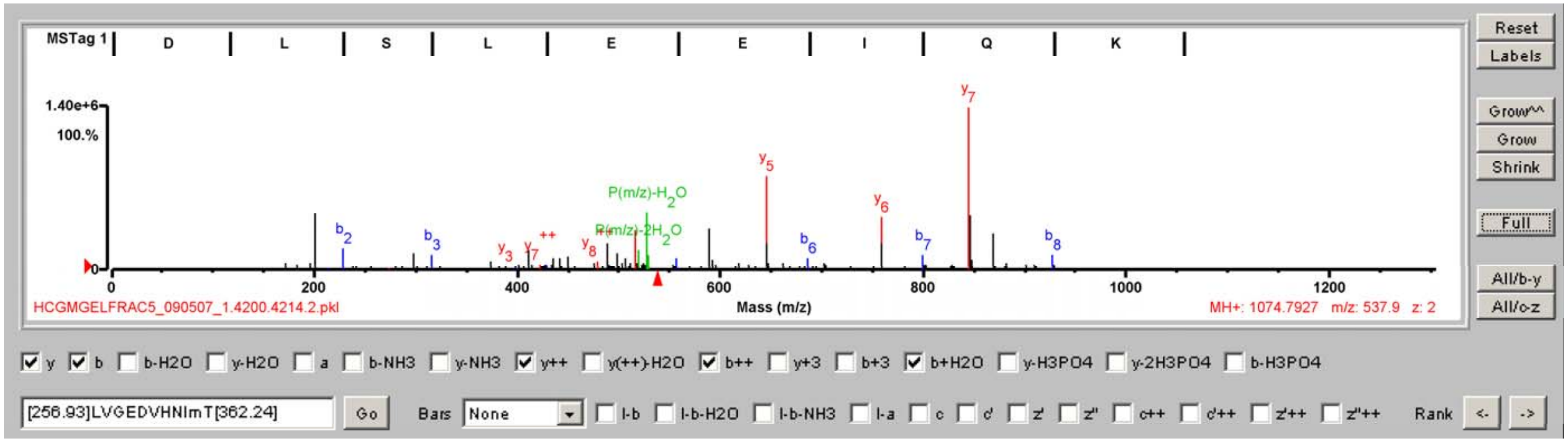

Stratifin-Tyrosine Monooxygenase SFN YWHAE, YWHAH, YWHAZ

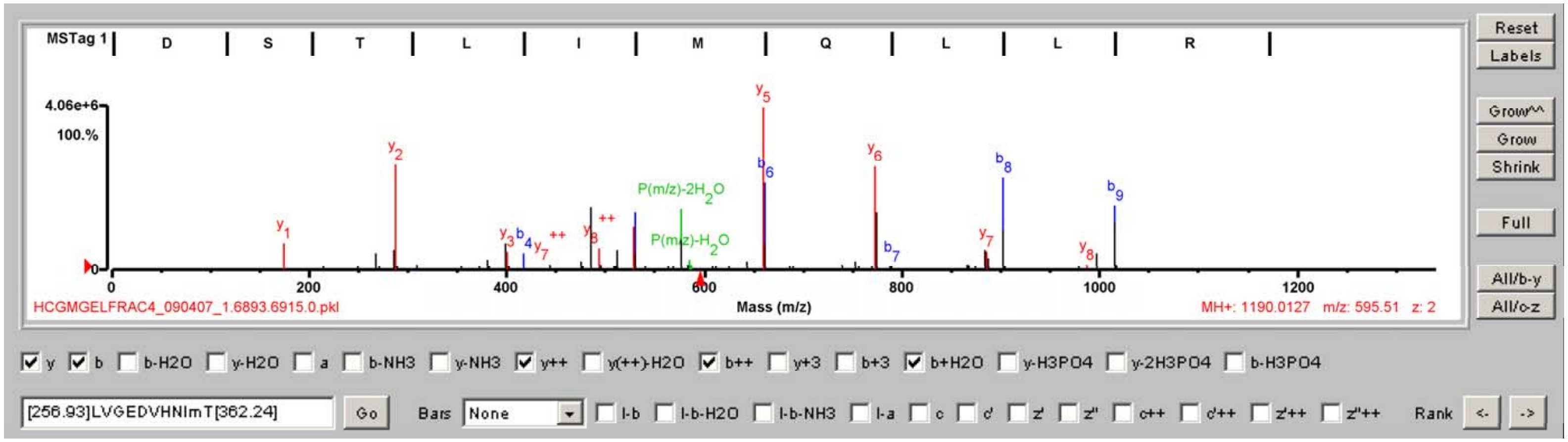

Synaptosomal-Associated Protein 25 Isoform Beta SNAP25

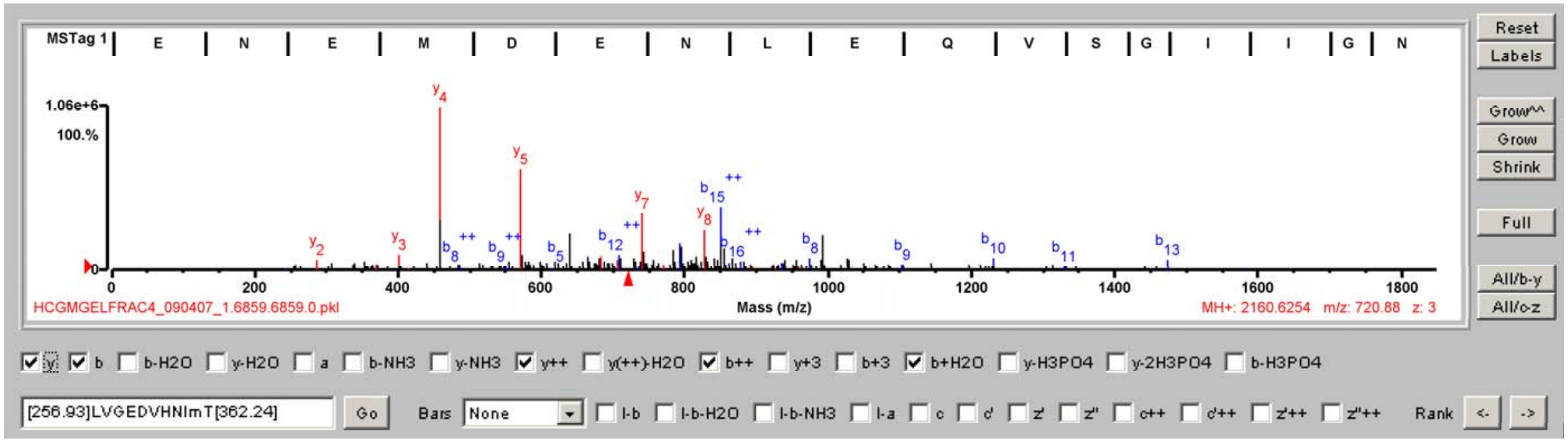

Transcobalamin II Precursor TCN2

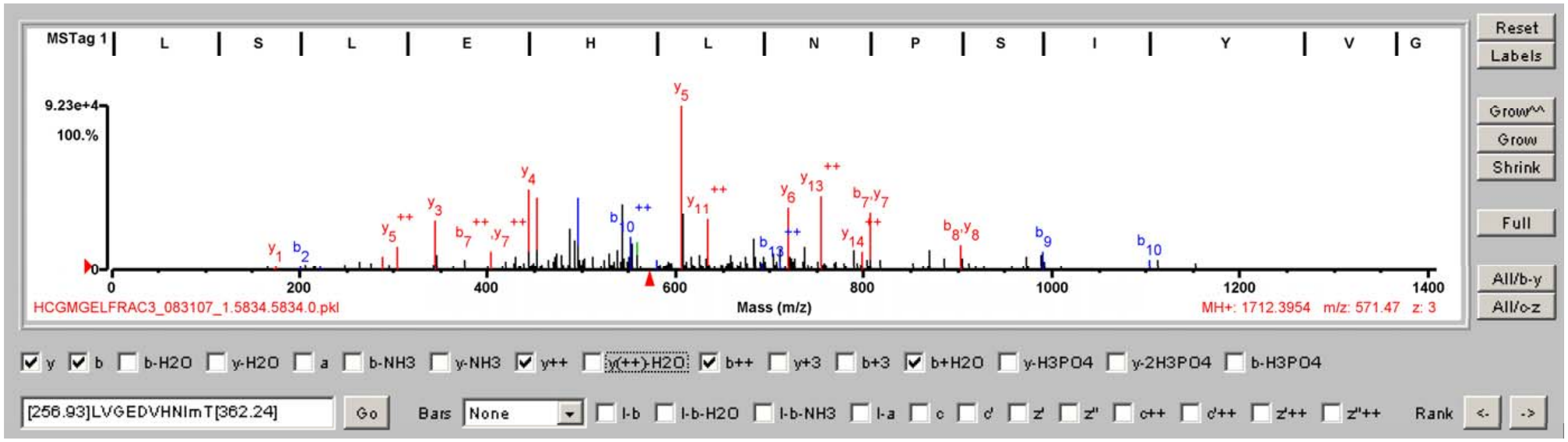

Transmembrane Protein 109 TMEM109

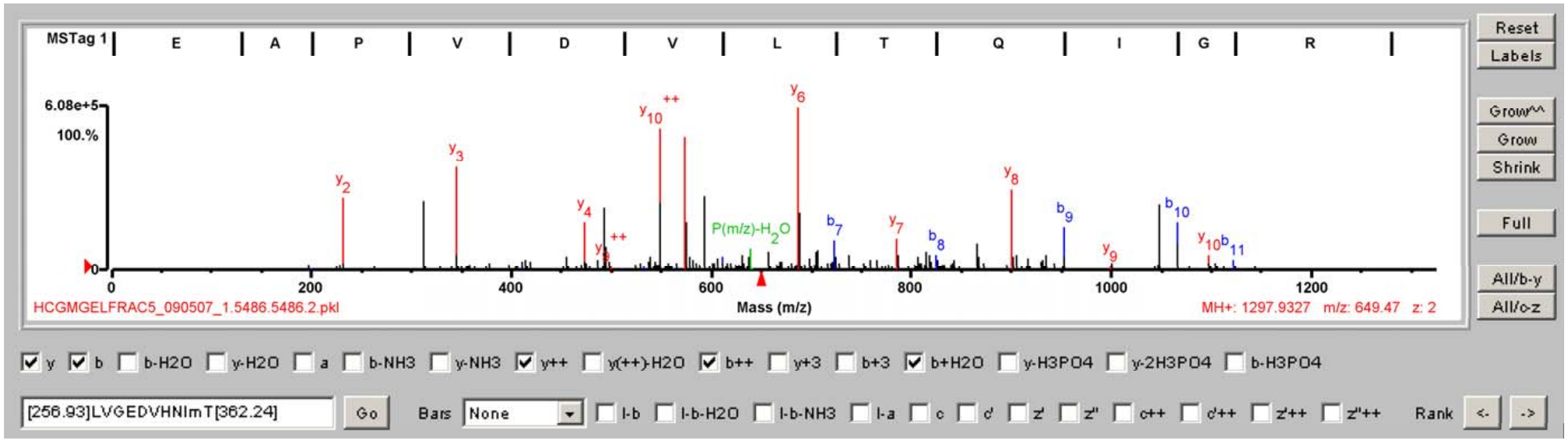

Tropomyosin 1 Alpha Chain, Tropomyosin 3 TPM1, TPM3

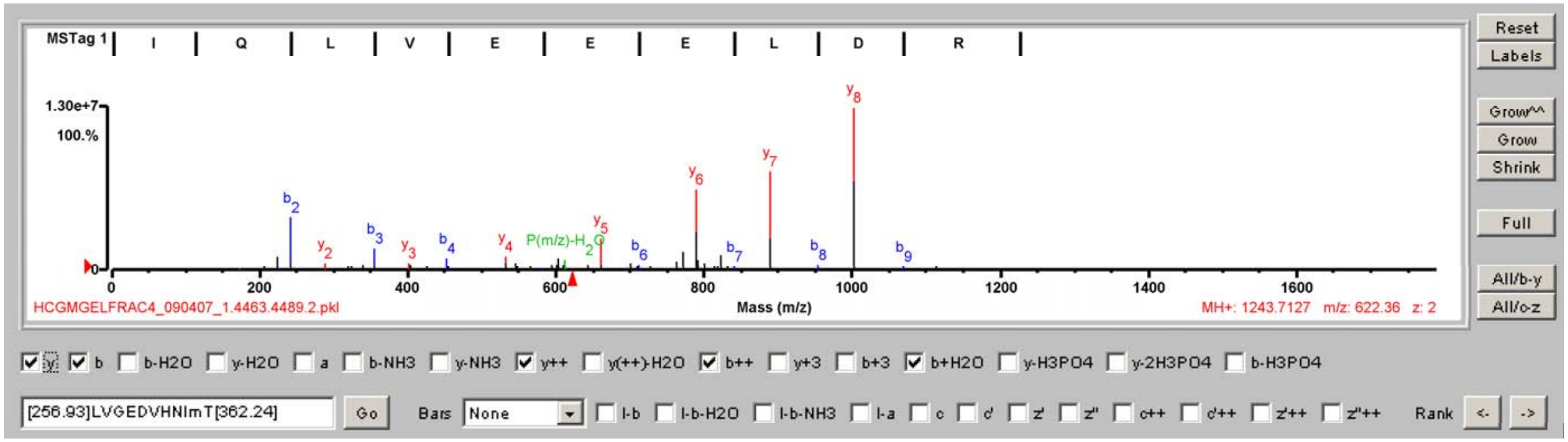

Ubiquinol-Cytochrome C Reductase UQCRQ

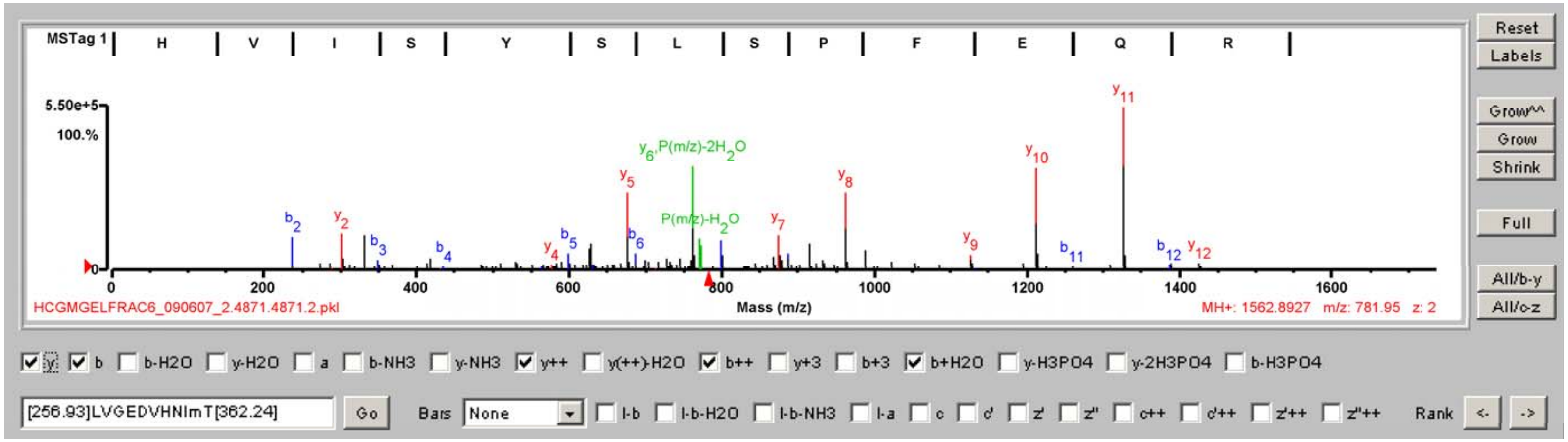

UDP-N-Acetyl-Alpha-D-Galactosamine:Polypeptide GALNT14

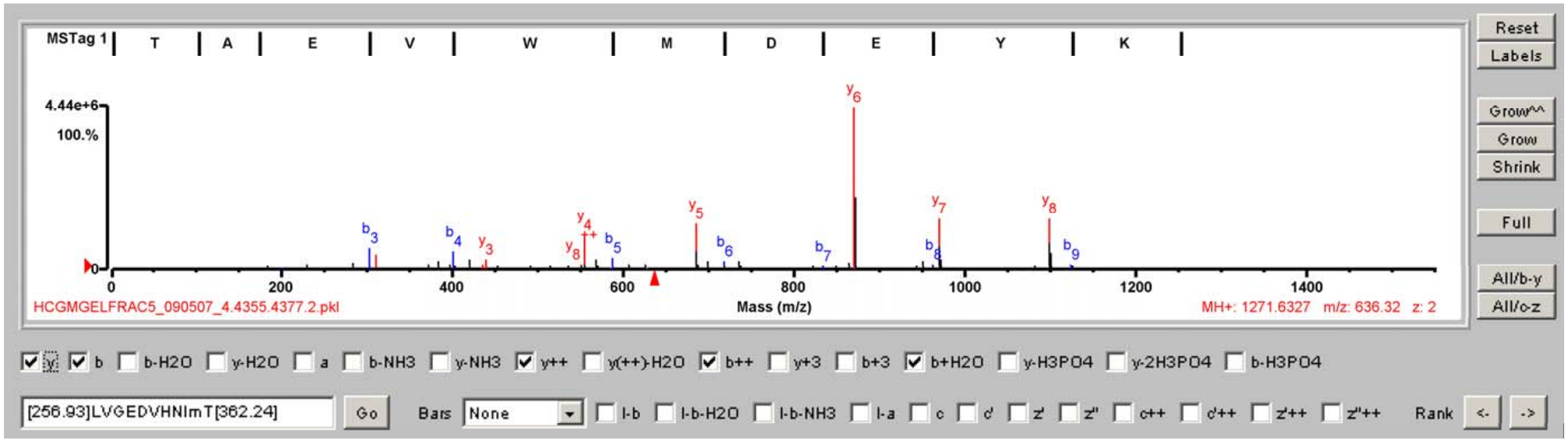

Supplement: Table S2 — Membrane proteins identified in human dense core secretory vesicles. Addendum: MS/MS spectra of single peptide identifications for membrane DCSV proteins. (PDF) [file pone.0041134.s005.pdf]
