# Supplementary material for: The Protein Architecture of Human Secretory Vesicles Reveals Differential Regulation of Signaling Molecule Secretion by Protein Kinases
Source: PLoS One. 2012 Aug 16;7(8):e41134. doi: 10.1371/journal.pone.0041134 (PMC3420874; doi:10.1371/journal.pone.0041134)
Supplement: Table S3 — Functional organization of proteins in human dense core secretory vesicles. (PDF) [file pone.0041134.s006.pdf]

TABLE S3	FUNCTIONAL ORGANIZATION OF PROTEINS IN HUMAN DENSE CORE SECRETORY VESICLES (DCSV)		
**Accession**	**Protein Description**	**Soluble**	**Membrane**
	**Production of Neurotransmitters and Neurohumoural Factors:**		
	**Neuropeptides (Proproteins) and Neurohumoural Factors**		
4502805	chromogranin A precursor		
4502807	chromogranin B precursor		
42716297	clusterin isoform 1		
110347431	latent transforming growth factor beta binding protein 4 isoform a		
4505449	neuropeptide Y		
5453876	proenkephalin		
68160947	secretogranin II precursor		
19557645	secretogranin III		
17136078	VGF nerve growth factor inducible precursor		
	**Protease Systems**		
4503009	carboxypeptidase E precursor		
119395729	cathepsin A precursor		
4503139	cathepsin B preproprotein		
4503143	cathepsin D preproprotein		
22538442	cathepsin Z preproprotein		
4503107	cystatin C precursor		
89274169	cytokine induced apoptosis inhibitor 1		
4758146	defensin, alpha 1 preproprotein		
4502249	development- and differentiation-enhancing factor 2		
62420888	dipeptidyl peptidase 7 preproprotein		
4506141	HtrA serine peptidase 1		
7019477	HtrA serine peptidase 2 isoform 1 preproprotein		
21614538	HtrA serine peptidase 2 isoform 2		
20149617	neural proliferation, differentiation and control, 1		
21070984	peptidylglycine alpha-amidating monooxygenase isoform a, preproprotein		
21070980	peptidylglycine alpha-amidating monooxygenase isoform c, preproprotein		
7706387	plasma glutamate carboxypeptidase		
24307907	plasminogen activator inhibitor type 1, member 2		
4505861	plasminogen activator, tissue type isoform 1 preproprotein		
113427619	PREDICTED: similar to Metalloproteinase inhibitor 2 precursor (TIMP-2) (Tissue inhibitor of metalloproteinases 2) (CSC-21K)		
113423966	PREDICTED: similar to Ubiquitin-63E CG11624-PA, isoform A		
4505643	procollagen C-endopeptidase enhancer		
117306169	prolylcarboxypeptidase isoform 2 preproprotein		
7019519	proprotein convertase subtilisin/kexin type 1 inhibitor precursor		
20336242	proprotein convertase subtilisin/kexin type 1 preproprotein		
20336244	proprotein convertase subtilisin/kexin type 2		
4506145	protease, serine, 1 preproprotein		
4506917	secretory granule, neuroendocrine protein 1 (7B2 protein)		
50363217	serine (or cysteine) proteinase inhibitor, clade A (alpha-1 antiproteinase, antitrypsin), member 1		
17998551	serine (or cysteine) proteinase inhibitor, clade B (ovalbumin), member 12		
32454741	serine (or cysteine) proteinase inhibitor, clade H, member 1		
4826904	serine (or cysteine) proteinase inhibitor, clade I (neuroserpin), member 1		
47132529	suppression of tumorigenicity 5 isoform 1		
21361794	TIP120 protein		
4507509	tissue inhibitor of metalloproteinase 1 precursor		
4507511	tissue inhibitor of metalloproteinase 2 precursor		
4507513	tissue inhibitor of metalloproteinase 3 precursor		
5729770	tripeptidyl-peptidase I preproprotein		
4507761	ubiquitin and ribosomal protein L40 precursor		
4506713	ubiquitin and ribosomal protein S27a precursor		
67191208	ubiquitin C		
21361091	ubiquitin carboxyl-terminal esterase L1 (ubiquitin thiolesterase)		
41281376	ubiquitin specific peptidase 8		
149192845	ubiquitin specific protease 24		
	**Neurotransmitter Enzymes/Transporters**		
38679946	4-aminobutyrate aminotransferase precursor		
4759310	bestrophin		
14251209	chloride intracellular channel 1		
4757910	cocaine- and amphetamine-regulated transcript		
116534900	dopamine beta-hydroxylase precursor		
55770878	neuronal pentraxin I precursor		
113427645	PREDICTED: similar to neuronal pentraxin I precursor		
4507949	tyrosine 3-monooxygenase/tryptophan 5-monooxygenase activation protein, beta polypeptide		
5803225	tyrosine 3/tryptophan 5 -monooxygenase activation protein, epsilon polypeptide		
4507951	tyrosine 3/tryptophan 5 -monooxygenase activation protein, eta polypeptide		
4507953	tyrosine 3/tryptophan 5 -monooxygenase activation protein, zeta polypeptide		
88900501	tyrosine hydroxylase isoform a		
18379349	vesicle amine transport protein 1		

	**Receptors**		
113722120	G protein-coupled receptor 98 precursor		
103472122	glutamate receptor interacting protein 1		
4504673	interleukin 6 receptor isoform 1 precursor		
113423398	PREDICTED: similar to Glutamate receptor-interacting protein 1 (GRIP1 protein)		
5729875	progesterone receptor membrane component 1		
5453916	progesterone receptor membrane component 2		
115430112	receptor accessory protein 5		
27777675	vomeronasal 1 receptor 5		
	**Biochemical Processes:**		
	**Enzymes**		
23308751	3-hydroxyisobutyrate dehydrogenase		
4501867	aconitase 2 precursor		
4557305	aldolase A		
73486658	aspartate aminotransferase 2 precursor		
9955948	carbonic anhydrase IX precursor		
9951923	carbonic anhydrase XI precursor		
38327625	citrate synthase precursor, isoform a		
5174539	cytosolic malate dehydrogenase		
24850115	cytosolic sialic acid 9-O-acetylesterase homolog		
7705925	dicarbonyl/L-xylulose reductase		
70906441	dUTP pyrophosphatase isoform 1 precursor		
4503571	enolase 1		
153267427	enolase 3		
4885063	fructose-bisphosphate aldolase C		
19743875	fumarate hydratase precursor		
6912618	glutaminyl-peptide cyclotransferase precursor		
7669492	glyceraldehyde-3-phosphate dehydrogenase		
15991829	hexokinase 1 isoform HKI-ta/tb		
15553127	hexokinase 2		
94538322	hydroxyacyl glutathione hydrolase isoform 1		
4758504	hydroxysteroid (17-beta) dehydrogenase 10 isoform 1		
7705855	hydroxysteroid (17-beta) dehydrogenase 12		
29171702	inorganic pyrophosphatase 2 isoform 1 precursor		
77812678	inorganic pyrophosphatase 2 isoform 2 precursor		
77812680	inorganic pyrophosphatase 2 isoform 3 precursor		
94557308	L-3-hydroxyacyl-Coenzyme A dehydrogenase precursor		
5031857	lactate dehydrogenase A		
4557894	lysozyme precursor		
32307144	lysyl hydroxylase precursor		
67782305	manganese superoxide dismutase isoform A precursor		
67782309	manganese superoxide dismutase isoform B precursor		
21735621	mitochondrial malate dehydrogenase precursor		
12707570	mitochondrial short-chain enoyl-coenzyme A hydratase 1 precursor		
4506031	palmitoyl-protein thioesterase 1 (ceroid-lipofuscinosis, neuronal 1, infantile)		
19923106	paraoxonase 1		
29570798	phosphoribosyl pyrophosphate amidotransferase proprotein		
113408766	PREDICTED: similar to Triosephosphate isomerase (TIM) (Triose-phosphate isomerase) isoform 1		
20070125	prolyl 4-hydroxylase, beta subunit precursor		
156564403	pyruvate dehydrogenase (lipoamide) beta		
33286418	pyruvate kinase 3 isoform 1		
118572613	splicing coactivator subunit SRm300		
115387094	succinate dehydrogenase complex, subunit B, iron sulfur (Ip)		
5453549	thioredoxin peroxidase		
4507645	triosephosphate isomerase 1		
	**Carbohydrate Functions**		
119393891	acid alpha-glucosidase preproprotein		
38202257	alpha glucosidase II alpha subunit isoform 2		
88900491	alpha glucosidase II alpha subunit isoform 3		
4505167	alpha-1,3(6)-mannosylglycoprotein beta-1,6-N-acetyl-glucosaminyltransferase		
4557781	alpha-N-acetylgalactosaminidase precursor		
4504349	beta globin		
4502841	carbohydrate (keratan sulfate Gal-6) sulfotransferase 1		
47419930	chondroitin sulfate proteoglycan 4		
119360348	fucosidase, alpha-L-1, tissue		
119372308	galactosidase, beta 1 isoform a		
4504061	glucosamine (N-acetyl)-6-sulfatase precursor		
4504223	glucuronidase, beta		
54633312	golgi apparatus protein 1		
4504371	hexosaminidase A preproprotein		
4504373	hexosaminidase B preproprotein		
24497519	mannosidase, alpha, class 1A, member 1		
51477716	mannosidase, alpha, class 2A, member 2		
51873064	mannosidase, alpha, class 2B, member 1 precursor		
84798622	mannosidase, beta A, lysosomal		

4505163	mannosyl (alpha-1,6-)-glycoprotein beta-1,2-N-acetylglucosaminyltransferase		
4758412	polypeptide N-acetylgalactosaminyltransferase 2		
5802984	UDP-GlcNAc:betaGal beta-1,3-N-acetylglucosaminyltransferase 1		
60498976	UDP-N-acetyl-alpha-D-galactosamine:polypeptide N-acetylgalactosaminyltransferase 14		
71043692	UDP-N-acetyl-alpha-D-galactosamine:polypeptide N-acetylgalactosaminyltransferase-like 1		
	**Lipid Functions**		
4557237	acetyl-Coenzyme A acetyltransferase 1 precursor		
4557321	apolipoprotein A-I preproprotein		
153266841	apolipoprotein H precursor		
13129148	apolipoprotein O		
62530384	dodecenoyl-Coenzyme A delta isomerase precursor		
5453678	epididymal secretory protein E1 precursor		
39995109	GM2 ganglioside activator precursor		
4885409	high density lipoprotein binding protein		
51317399	lipase A precursor		
30089930	N-acylsphingosine amidohydrolase (acid ceramidase) 1 isoform b		
33469933	phospholipase C beta 4 isoform a		
110224476	prosaposin isoform b preproprotein		
23618867	sideroflexin 1		
55770862	thyroglobulin		
	**Reduction-Oxidation**		
4503301	2,4-dienoyl CoA reductase 1 precursor*		
156151369	cell death-regulatory protein GRIM19*		
63054828	cytochrome b-561		
47778923	cytochrome b5 reductase b5R.2		
4503327	cytochrome b5 reductase isoform 1		
17981856	cytochrome c oxidase subunit II		
4502981	cytochrome c oxidase subunit IV isoform 1 precursor		
4758038	cytochrome c oxidase subunit Va precursor		
17017988	cytochrome c oxidase subunit Vb precursor		
4502987	cytochrome c oxidase subunit VIIa polypeptide 1 (muscle) precursor		
4502989	cytochrome c oxidase subunit VIIa polypeptide 2 (liver) precursor		
18105037	cytochrome c oxidase subunit VIIa polypeptide 2 like		
4502993	cytochrome c oxidase subunit VIIc precursor		
21359867	cytochrome c-1		
11128019	cytochrome c		
4503607	electron transfer flavoprotein, alpha polypeptide		
62420877	electron-transfer-flavoprotein, beta polypeptide isoform 2		
15277342	estradiol 17 beta-dehydrogenase 8		
41406084	glutathione peroxidase 1 isoform 1		
41406082	glutathione peroxidase 1 isoform 2		
7705704	glutathione transferase kappa 1		
4504183	glutathione transferase		
49574502	NAD(P)H:quinone oxidoreductase type 3, polypeptide A2		
4758768	NADH dehydrogenase (ubiquinone) 1 alpha subcomplex, 10, 42kDa precursor		
28269681	NADH dehydrogenase (ubiquinone) 1 alpha subcomplex, 11, 14.7kDa		
4505355	NADH dehydrogenase (ubiquinone) 1 alpha subcomplex, 2, 8kDa		
4758772	NADH dehydrogenase (ubiquinone) 1 alpha subcomplex, 3, 9kDa		
4826848	NADH dehydrogenase (ubiquinone) 1 alpha subcomplex, 5		
51317370	NADH dehydrogenase (ubiquinone) 1 alpha subcomplex, 6, 14kDa		
103472001	NADH dehydrogenase (ubiquinone) 1 alpha subcomplex, 7, 14.5kDa		
7657369	NADH dehydrogenase (ubiquinone) 1 alpha subcomplex, 8, 19kDa		
6681764	NADH dehydrogenase (ubiquinone) 1 alpha subcomplex, 9, 39kDa		
38569473	NADH dehydrogenase (ubiquinone) 1 beta subcomplex, 1, 7kDa		
4758774	NADH dehydrogenase (ubiquinone) 1 beta subcomplex, 10, 22kDa		
4758778	NADH dehydrogenase (ubiquinone) 1 beta subcomplex, 2, 8kDa precursor		
4505361	NADH dehydrogenase (ubiquinone) 1 beta subcomplex, 3, 12kDa		
6041669	NADH dehydrogenase (ubiquinone) 1 beta subcomplex, 4, 15kDa		
4505363	NADH dehydrogenase (ubiquinone) 1 beta subcomplex, 5, 16kDa precursor		
4505365	NADH dehydrogenase (ubiquinone) 1 beta subcomplex, 6, 17kDa isoform 1		
10764847	NADH dehydrogenase (ubiquinone) 1 beta subcomplex, 7, 18kDa		
4826854	NADH dehydrogenase (ubiquinone) 1 beta subcomplex, 8, 19kDa		
6274550	NADH dehydrogenase (ubiquinone) 1 beta subcomplex, 9, 22kDa		
4826852	NADH dehydrogenase (ubiquinone) 1, alpha/beta subcomplex, 1, 8kDa		
4758788	NADH dehydrogenase (ubiquinone) Fe-S protein 3, 30kDa (NADH-coenzyme Q reductase)		
4505369	NADH dehydrogenase (ubiquinone) Fe-S protein 4, 18kDa (NADH-coenzyme Q reductase)		
4758790	NADH dehydrogenase (ubiquinone) Fe-S protein 5, 15kDa (NADH-coenzyme Q reductase)		
4758792	NADH dehydrogenase (ubiquinone) Fe-S protein 6, 13kDa (NADH-coenzyme Q reductase)		
4505371	NADH dehydrogenase (ubiquinone) Fe-S protein 8, 23kDa (NADH-coenzyme Q reductase)		
10835025	NADH dehydrogenase (ubiquinone) flavoprotein 2, 24kDa		
17981862	NADH dehydrogenase subunit 4		
38524585	NADH-ubiquinone oxidoreductase Fe-S protein 7		
4505591	peroxiredoxin 1		
32189392	peroxiredoxin 2 isoform a		
33188454	peroxiredoxin 2 isoform c		

5802974	peroxiredoxin 3 isoform a precursor		
6912238	peroxiredoxin 5 precursor, isoform a		
32455260	peroxiredoxin 5 precursor, isoform b		
32455262	peroxiredoxin 5 precursor, isoform c		
6006001	plasma glutathione peroxidase 3 precursor		
88974095	PREDICTED: similar to NADH dehydrogenase (ubiquinone) 1 beta subcomplex, 4, 15kDa		
13325075	quiescin Q6 sulfhydryl oxidase 1 isoform a		
4506359	quinoid dihydropteridine reductase		
5454152	ubiquinol-cytochrome c reductase binding protein		
83367083	ubiquinol-cytochrome c reductase, complex III subunit VII		
5174743	ubiquinol-cytochrome c reductase, Rieske iron-sulfur polypeptide 1		
	**Internal Conditions of Secretory Vesicles:**		
	**ATPases and Nucleotide Metabolism**		
4502201	ADP-ribosylation factor 1		
4502209	ADP-ribosylation factor 5		
8922601	ADP-ribosylation factor-like 10C		
24308007	ADP-ribosylation factor-like 6 interacting protein		
5453704	ADP-ribosylation-like factor 6 interacting protein 5		
38569421	ATP citrate lyase isoform 1		
17981857	ATP synthase F0 subunit 8		
21361565	ATP synthase, H+ transporting, mitochondrial F0 complex, subunit B1 precursor		
5453559	ATP synthase, H+ transporting, mitochondrial F0 complex, subunit d isoform a		
51479152	ATP synthase, H+ transporting, mitochondrial F0 complex, subunit d isoform b		
6005717	ATP synthase, H+ transporting, mitochondrial F0 complex, subunit E		
4757812	ATP synthase, H+ transporting, mitochondrial F0 complex, subunit F2 isoform 2a		
51479132	ATP synthase, H+ transporting, mitochondrial F0 complex, subunit F2 isoform 2c		
51479156	ATP synthase, H+ transporting, mitochondrial F0 complex, subunit G		
4757810	ATP synthase, H+ transporting, mitochondrial F1 complex, alpha subunit precursor		
32189394	ATP synthase, H+ transporting, mitochondrial F1 complex, beta subunit precursor		
4502297	ATP synthase, H+ transporting, mitochondrial F1 complex, delta subunit precursor		
50345988	ATP synthase, H+ transporting, mitochondrial F1 complex, gamma subunit isoform L (liver) precursor		
17978471	ATPase, aminophospholipid transporter (APLT), class I, type 8A, member 1		
55743071	ATPase, Cu++ transporting, beta polypeptide isoform a		
18087815	ATPase, H+ transporting, lysosomal 31kDa, V1 subunit E isoform 2		
4502315	ATPase, H+ transporting, lysosomal 42kDa, V1 subunit C1		
47717102	ATPase, H+ transporting, lysosomal 50/57kDa, V1 subunit H isoform 2		
19913426	ATPase, H+ transporting, lysosomal 56/58kDa, V1 subunit B1		
19913424	ATPase, H+ transporting, lysosomal 70kD, V1 subunit A, isoform 1		
17136148	ATPase, H+ transporting, lysosomal accessory protein 1 precursor		
15011918	ATPase, H+ transporting, lysosomal accessory protein 2		
19913418	ATPase, H+ transporting, lysosomal V0 subunit a isoform 1		
4502313	ATPase, H+ transporting, lysosomal, V0 subunit c		
19913432	ATPase, H+ transporting, lysosomal, V0 subunit d1		
7706757	H(+)-transporting two-sector ATPase		
4502303	mitochondrial ATP synthase, O subunit precursor		
4502277	Na+/K+ -ATPase beta 1 subunit isoform a		
38044284	neuron navigator 2 isoform 1		
38044282	neuron navigator 2 isoform 2		
113415078	PREDICTED: similar to ADP-ribosylation factor 4		
4502317	vacuolar H+ ATPase E1 isoform a		
87159818	vacuolar H+ ATPase E1 isoform c		
4757818	vacuolar H+ ATPase G1		
19913428	vacuolar H+ATPase B2		
4507879	voltage-dependent anion channel 1		
42476281	voltage-dependent anion channel 2		
	**Protein Folding**		
31542947	chaperonin		
18201905	glucose phosphate isomerase		
24308295	GrpE-like 1, mitochondrial		
4504523	heat shock 10kDa protein 1 (chaperonin 10)		
4504517	heat shock 27kDa protein 1		
13676857	heat shock 70kDa protein 2		
16507237	heat shock 70kDa protein 5		
34419635	heat shock 70kDa protein 6 (HSP70B')		
5729877	heat shock 70kDa protein 8 isoform 1		
24234688	heat shock 70kDa protein 9 precursor		
20149594	heat shock 90kDa protein 1, beta		
153792590	heat shock protein 90kDa alpha (cytosolic), class A member 1 isoform 1		
5453832	oxygen regulated protein precursor		
30102944	peptidylprolyl isomerase A (cyclophilin A)-like 4		
4758950	peptidylprolyl isomerase B precursor		
113421305	PREDICTED: similar to peptidylprolyl isomerase A isoform 1		
113421305	PREDICTED: similar to peptidylprolyl isomerase A isoform 1		
113421305	PREDICTED: similar to peptidylprolyl isomerase A isoform 1		
113421305	PREDICTED: similar to peptidylprolyl isomerase A isoform 1		

113421305	PREDICTED: similar to peptidylprolyl isomerase A isoform 1		
113421305	PREDICTED: similar to peptidylprolyl isomerase A isoform 1		
4506113	prion protein preproprotein		
21361657	protein disulfide isomerase-associated 3 precursor		
5031973	protein disulfide isomerase-associated 6		
33285002	selenoprotein S		
	**Regulated Secretion Mechanisms:**		
	**Signal Transduction and GTP-Binding Proteins**		
53759122	adenomatosis polyposis coli		
153266792	adenylate cyclase activating polypeptide precursor		
4502011	adenylate kinase 1		
19923437	adenylate kinase 3		
61743954	AHNAK nucleoprotein isoform 1		
19923445	atlastin isoform a		
30795231	brain abundant, membrane attached signal protein 1		
21536286	brain creatine kinase		
15718763	c-K-ras2 protein isoform a		
18079216	CASK interacting protein 1		
4757952	cell division cycle 42 isoform 1		
10334859	creatine kinase, mitochondrial 1B precursor		
40068047	cyclin M3 isoform 2		
31543380	DJ-1 protein		
17149842	FK506-binding protein 2 precursor		
4503727	FK506-binding protein 3		
22547161	frizzled 4		
13994190	frizzled 8		
51036603	G-protein gamma-12 subunit		
117938759	GNAS complex locus isoform c		
19923483	GTPase Rab14		
42476111	guanine nucleotide binding protein (G protein) alpha 12		
4758444	guanine nucleotide binding protein (G protein), alpha 14		
33695153	guanine nucleotide binding protein (G protein), alpha activating activity polypeptide, olfactory type isoform 1		
4504041	guanine nucleotide binding protein (G protein), alpha inhibiting activity polypeptide 2		
5729850	guanine nucleotide binding protein (G protein), alpha inhibiting activity polypeptide 3		
5174447	guanine nucleotide binding protein (G protein), beta polypeptide 2-like 1		
6912394	guanine nucleotide binding protein (G protein), gamma 3		
4758450	guanine nucleotide binding protein (G protein), gamma 4		
32698769	guanine nucleotide binding protein (G protein), gamma 7		
15147246	guanine nucleotide binding protein (G protein), gamma 8		
40254462	guanine nucleotide binding protein (G protein), q polypeptide		
10567816	guanine nucleotide binding protein, alpha activating polypeptide O		
156139155	guanine nucleotide binding protein, alpha transducing 3		
22027520	guanine nucleotide binding protein, alpha transducing activity polypeptide 1		
20330805	guanine nucleotide binding protein, alpha transducing activity polypeptide 2		
126131099	guanine nucleotide exchange factor p532		
16933567	mel transforming oncogene		
27363461	mitochondria-associated granulocyte macrophage CSF signaling molecule		
21735550	mitogen-activated protein kinase 10		
11496277	mitogen-activated protein kinase interacting protein 1		
47933379	N-ethylmaleimide-sensitive factor attachment protein, alpha		
44917606	N-ethylmaleimide-sensitive factor attachment protein, beta		
156564401	N-ethylmaleimide-sensitive factor		
4505451	neuroblastoma RAS viral (v-ras) oncogene homolog		
109826564	neurofibromin isoform 1		
4557793	neurofibromin isoform 2		
24638433	nicastrin precursor		
4826894	phosphodiesterase 1C, calmodulin-dependent 70kDa		
4505763	phosphoglycerate kinase 1		
54792082	phosphoinositide-3-kinase, catalytic, alpha polypeptide		
14211923	PKCI-1-related HIT protein		
113425016	PREDICTED: similar to AHNAK nucleoprotein isoform 1		
89038979	PREDICTED: similar to creatine kinase, mitochondrial 1B precursor		
88984860	PREDICTED: similar to Guanine nucleotide-binding protein beta subunit 2-like 1 (Receptor of activated protein kinase C 1) (RACK1)		
113419695	PREDICTED: similar to Guanine nucleotide-binding protein G(t), alpha-3 subunit (Gustducin alpha-3 chain)		
113422501	PREDICTED: similar to Olfactory guanylyl cyclase GC-D precursor (Guanylate cyclase, olfactory)		
48255889	protein kinase C substrate 80K-H isoform 1		
4505581	protein kinase, interferon-inducible double stranded RNA dependent activator		
63003905	protein phosphatase 1 (formerly 2C)-like		
4506283	protein tyrosine phosphatase type IVA, member 1		
11386149	protein tyrosine phosphatase, receptor type, N polypeptide 2 isoform 1 precursor		
19743914	protein tyrosine phosphatase, receptor type, N polypeptide 2 isoform 3 precursor		
4506321	protein tyrosine phosphatase, receptor type, N precursor		
21361884	RAB2B protein		
31543537	RAB39		
45267837	rabphilin 3A homolog		
33946329	ras related v-ral simian leukemia viral oncogene homolog A		

50263042	ras-like protein TC10		
9845509	ras-related C3 botulinum toxin substrate 1 isoform Rac1b		
82659107	ras-related GTP-binding protein 4b		
33695095	ras-related GTP-binding protein RAB10		
4758984	Ras-related protein Rab-11A		
10190714	RAS-related protein RAB-22A		
19923264	Ras-related protein Rab-27A		
4758996	Ras-related protein Rab-33A		
4757766	Rho GTPase activating protein 1		
5454090	signal sequence receptor, delta		
154689780	son of sevenless homolog 2		
5454052	stratifin		
109452591	succinate-CoA ligase, GDP-forming, alpha subunit		
8922964	synaptojanin 2 binding protein		
18765733	synaptosomal-associated protein 25 isoform SNAP25A		
21464101	tyrosine 3-monooxygenase/tryptophan 5-monooxygenase activation protein, gamma polypeptide		
4885425	v-Ha-ras Harvey rat sarcoma viral oncogene homolog isofrom 1		
	**Vesicular Trafficking**		
4758012	clathrin heavy chain 1		
108860681	clathrin, heavy polypeptide-like 1		
7706337	coatomer protein complex, subunit zeta 1		
59853099	dynamin 1 isoform 1		
56549121	dynamin 2 isoform 1		
21359818	exophilin 5		
90903231	huntingtin		
33620775	kinectin 1 isoform a		
118498362	kinectin 1 isoform b		
118498368	kinectin 1 isoform c		
4505399	nipsnap homolog 1		
22267436	nipsnap homolog 3A		
5031631	scavenger receptor class B, member 2		
5803165	Sec61 beta subunit		
16445419	secretory carrier membrane protein 3 isoform 1		
27764867	synaptophysin		
5032139	synaptotagmin I		
31543670	synaptotagmin II		
92859638	synaptotagmin V		
4759182	syntaxin 1A (brain)		
31652247	tomosyn		
4759302	VAMP-associated protein B/C		
7657675	vesicle-associated membrane protein 2 (synaptobrevin 2)		
4759300	vesicle-associated membrane protein 3 (cellubrevin)		
94721250	vesicle-associated membrane protein-associated protein A isoform 1		
94721252	vesicle-associated membrane protein-associated protein A isoform 2		
	**Calcium Regulation**		
4502107	annexin 5		
50845388	annexin A2 isoform 1		
71773329	annexin VI isoform 1		
4502523	calcium channel, voltage-dependent, N type, alpha 1B subunit		
10716563	calnexin precursor		
4757900	calreticulin precursor		
119395727	cardiac calsequestrin 2		
4504165	gelsolin isoform a precursor		
19913441	hippocalcin-like 1		
78190498	secreted modular calcium-binding protein 1 isoform 1		
4759164	sparc/osteonectin, cwcv and kazal-like domains proteoglycan 1 precursor		
4507265	stanniocalcin 1 precursor		
	**Morphological Functions of Secretory Vesicles:**		
	**Structural Proteins**		
5031597	actin related protein 2/3 complex subunit 3		
4501891	actinin, alpha 1		
4501893	actinin, alpha 2		
4557241	actinin, alpha 3		
12025678	actinin, alpha 4		
4501881	alpha 1 actin precursor		
148536825	alpha 1 type IV collagen preproprotein		
4502961	alpha 1 type VII collagen precursor		
89142730	alpha 3 type IV collagen isoform 1 precursor		
55743098	alpha 3 type VI collagen isoform 1 precursor		
55743100	alpha 3 type VI collagen isoform 2 precursor		
55743102	alpha 3 type VI collagen isoform 3 precursor		
116256356	alpha 4 type IV collagen precursor		
5031569	ARP1 actin-related protein 1 homolog A, centractin alpha		
4502389	barrier to autointegration factor 1		

13562114	beta tubulin 1, class VI		
55770834	centromere protein F (350/400kD)		
57863301	CLIP-associating protein 2		
4502951	collagen, type III, alpha 1 preproprotein		
24308029	dedicator of cytokinesis 9		
13259510	dynactin 1 isoform 1		
5453629	dynactin 2		
24307879	dynein, cytoplasmic 1, intermediate chain 2		
33350932	dynein, cytoplasmic, heavy polypeptide 1		
126352440	elastin isoform a		
5901944	elastin microfibril interfacer 1		
70906435	fibrinogen, beta chain preproprotein		
70906439	fibrinogen, gamma chain isoform gamma-B precursor		
116063573	filamin A, alpha		
105990514	filamin B, beta (actin binding protein 278)		
5031863	galectin 3 binding protein		
116805322	gamma filamin		
4503979	glial fibrillary acidic protein		
33188445	microfilament and actin filament cross-linker protein isoform a		
33188443	microfilament and actin filament cross-linker protein isoform b		
95147555	microtubule-associated protein 1A		
153945728	microtubule-associated protein 1B		
87578396	microtubule-associated protein 2 isoform 1		
41406064	myosin, heavy polypeptide 10, non-muscle		
17986258	myosin, light chain 6, alkali, smooth muscle and non-muscle isoform 1		
41281521	non-SMC condensin I complex, subunit D2		
21264345	peripherin		
113424259	PREDICTED: similar to Collagen alpha-1(I) chain precursor		
4505893	proteolipid protein 2 (colonic epithelium-enriched)		
8922712	septin 11		
4758158	septin 2		
4505303	smooth muscle and non-muscle myosin alkali light chain 6B		
67782321	spectrin beta isoform a		
118918407	spectrin repeat containing, nuclear envelope 2 isoform 5		
154759259	spectrin, alpha, non-erythrocytic 1 (alpha-fodrin)		
112382250	spectrin, beta, non-erythrocytic 1 isoform 1		
115430237	spectrin, beta, non-erythrocytic 4 isoform sigma1		
5031851	stathmin 1		
16753233	talin 1		
110349719	titin isoform N2-A		
110349715	titin isoform N2-B		
110349713	titin isoform novex-1		
110349717	titin isoform novex-2		
4507357	transgelin 2		
56549135	transgelin 3		
63252896	tropomyosin 1 alpha chain isoform 3		
27597085	tropomyosin 1 alpha chain isoform 5		
63252904	tropomyosin 1 alpha chain isoform 6		
63252906	tropomyosin 1 alpha chain isoform 7		
42476296	tropomyosin 2 (beta) isoform 1		
47519616	tropomyosin 2 (beta) isoform 2		
114155140	tropomyosin 3 isoform 1		
24119203	tropomyosin 3 isoform 2		
114155148	tropomyosin 3 isoform 5		
4507651	tropomyosin 4		
17986283	tubulin, alpha 1a		
50592996	tubulin, beta, 4		
6005942	valosin-containing protein		
62414289	vimentin		
	**Cell Adhesion/Cell-Cell Interactions**		
14589889	cadherin 2, type 1 preproprotein		
13325064	cadherin EGF LAG seven-pass G-type receptor 2		
5031635	cofilin 1 (non-muscle)		
14719392	cofilin 2		
28373117	contactin 1 isoform 1 precursor		
16933557	dachsous 1 precursor		
55749932	desmin		
148539835	dystroglycan 1 preproprotein		
118572606	hemicentin 1		
30794472	hyaluronan and proteoglycan link protein 4		
14249342	internexin neuronal intermediate filament protein, alpha		
119395746	Kallmann syndrome 1 protein precursor		
4557707	L1 cell adhesion molecule isoform 1 precursor		
38788416	laminin, alpha 1 precursor		
30520310	metadherin		
116642887	myofibrillogenesis regulator 1 isoform 1		

116642885	myofibrillogenesis regulator 1 isoform 3		
46397398	nephrocystin isoform 1		
32483416	neurofilament, heavy polypeptide 200kDa		
105990539	neurofilament, light polypeptide 68kDa		
4885513	neurofilament, medium polypeptide 150kDa		
115298674	nidogen 1 precursor		
116686120	periaxin isoform 2		
148886654	polydom		
21264343	scaffold attachment factor B		
34850061	superiorcervical ganglia, neural specific 10		
7669550	vinculin isoform meta-VCL		
4507877	vinculin isoform VCL		
	**Other Protein Categories:**		
	**Cell Growth and Development**		
62821787	COBL-like 1		
40548389	dickkopf homolog 3 precursor		
124494254	ErbB3-binding protein 1		
49355721	growth and transformation-dependent protein		
4826740	growth differentiation factor 10 precursor		
153792495	growth differentiation factor 15		
4504609	insulin-like growth factor 2		
55925576	insulin-like growth factor binding protein 2, 36kDa		
4505135	midkine		
13376808	platelet derived growth factor D isoform 1 precursor		
4506281	pleiotrophin		
24431935	reticulon 4 isoform A		
47519490	reticulon 4 isoform D		
11321571	slit homolog 3		
14249376	upregulated during skeletal muscle growth 5		
	**Immune**		
4557291	autoimmune regulator isoform 1		
4557417	CD14 antigen precursor		
10835165	CD59 antigen p18-20		
4502679	CD63 antigen isoform A		
88758613	CD93 antigen precursor		
115298678	complement component 3 precursor		
45580688	complement component 7 precursor		
86793109	complement receptor 1 isoform S precursor		
9845297	diablo isoform 1 precursor		
21070976	diablo isoform 3 precursor		
4557329	fas ligand		
4758344	Fc fragment of IgE, high affinity I, receptor for, gamma polypeptide precursor		
31317236	GSK-3 binding protein FRAT1		
38569394	inhibitor of kappa light polypeptide gene enhancer in B-cells, kinase complex-associated protein		
24797067	major histocompatibility complex, class I, A precursor		
62912479	major histocompatibility complex, class I, E precursor		
4504415	major histocompatibility complex, class I, G precursor		
91718902	myeloid/lymphoid or mixed-lineage leukemia 3		
113426081	PREDICTED: similar to B-cell receptor-associated protein 31 (BCR-associated protein Bap31) (p28 Bap31)		
113417848	PREDICTED: similar to HLA class I histocompatibility antigen, B-18 alpha chain precursor (MHC class I antigen B*18)		
4505773	prohibitin		
21361619	toll interacting protein		
	**Miscellaneous:**		
	**Miscellaneous**		
4502027	albumin precursor		
4504345	alpha 2 globin		
4757826	beta-2-microglobulin precursor		
4503049	cysteine-rich protein 2		
4885393	epsilon globin		
41327741	ETHE1 protein		
20149498	ferritin, light polypeptide		
6715607	G-gamma globin		
11321561	hemopexin		
7661786	hormone-regulated proliferation-associated 20 kDa protein		
4557659	iduronate-2-sulfatase isoform a precursor		
56699456	iron-sulfur cluster assembly enzyme isoform ISCU2 precursor		
40354192	keratin 10		
47132620	keratin 2		
31559829	keratin 25		
114431246	keratin 25D		
31559815	keratin 26		
153945736	keratin 27		
109255249	keratin 4		
153791670	keratin 76		

45597458	keratin 77		
117938328	l(3)mbt-like isoform I		
112380628	lysosomal-associated membrane protein 1		
55956910	meningioma 1		
38569475	metaxin 1 isoform 1		
38569477	metaxin 1 isoform 2		
113417289	PREDICTED: similar to odd Oz/ten-m homolog 2 isoform 5		
4505621	prostatic binding protein		
22094987	raptor		
13899247	retbindin isoform 2		
4506451	retinol binding protein 1, cellular		
113204615	skeletal muscle ryanodine receptor isoform 1		
33943099	surfactant, pulmonary-associated protein B		
21071010	transcobalamin II precursor		
4557871	transferrin		
13129092	transmembrane protein 109		
7657176	transmembrane protein 4		
134288906	tripartite motif-containing 67		
4507677	tumor rejection antigen (gp96) 1		
148536823	type IV alpha 6 collagen isoform A precursor		
17999541	vacuolar protein sorting 35		
	**Unknown**		
83921602	hypothetical protein LOC124565 isoform a		
148612838	hypothetical protein LOC158358		
40255080	hypothetical protein LOC158763		
27734917	hypothetical protein LOC196463		
115648140	hypothetical protein LOC23732		
63055057	hypothetical protein LOC345651		
63055043	hypothetical protein LOC374882		
49169841	hypothetical protein LOC414919		
8923579	hypothetical protein LOC55004		
150456436	hypothetical protein LOC55719		
116812630	hypothetical protein LOC56948		
154090979	hypothetical protein LOC57481		
150456444	hypothetical protein LOC57578		
24308257	hypothetical protein LOC57687		
149773456	hypothetical protein LOC643314		
124248546	hypothetical protein LOC79802		
14150017	hypothetical protein LOC84233		
14150100	hypothetical protein LOC84284		
148596959	hypothetical protein LOC84293		
24308354	hypothetical protein LOC89927		
4758940	hypothetical protein LOC9556		
66392203	NME1-NME2 protein		
113411509	PREDICTED: similar to CG17807-PA		
88961345	PREDICTED: similar to CG9007-PA isoform 17		
88966020	PREDICTED: similar to loss of heterozygosity, 11, chromosomal region 2		
88953571	PREDICTED: similar to Prostate, ovary, testis expressed protein on chromosome 2 isoform 2		
113422497	PREDICTED: similar to trophoblast glycoprotein		
45827731	scribble isoform a		
