# Supplementary material for: The Protein Architecture of Human Secretory Vesicles Reveals Differential Regulation of Signaling Molecule Secretion by Protein Kinases
Source: PLoS One. 2012 Aug 16;7(8):e41134. doi: 10.1371/journal.pone.0041134 (PMC3420874; doi:10.1371/journal.pone.0041134)
Supplement: Table S4 — Relative quantitation of human DCSV proteins by normalized spectral abundance factor (NSAF). (PDF) [file pone.0041134.s007.pdf]

TABLE S4	NSAF QUANTIATION OF HUMAN DCSV PROTEINS												
**Accession**	**Protein Description**	**uniprot**	**entrezgene**	**hgnc**	**NSAF Ave**	**NSAF SD**	**Ave ln(NSAF Total)**	**SD ln(NSAF Total)**	**Ave ln(NSAF Mem)**	**SD ln(NSAF Mem)**	**Ave ln(NSAF Sol)**	**SD ln(NSAF Sol)**	**t-test (Mem-Sol)**
	**Production of Neurotransmitters and Neurohumoural Factors:**												
	**Neuropeptides (Proproteins) and Neurohumoural Factors**												
4502805	chromogranin A precursor	P10645	1113	CHGA	9.851E-02	1.605E-02	-2.328E+00	1.654E-01	-3.710E+00	6.558E-02	-1.979E+00	1.858E-01	6.781E-04
4502807	chromogranin B precursor	P05060	1114	CHGB	5.630E-02	2.042E-03	-2.878E+00	3.613E-02	-3.850E+00	4.580E-02	-2.726E+00	9.080E-02	2.791E-04
68160947	secretogranin II precursor	P13521	7857	SCG2	2.214E-02	3.741E-03	-3.821E+00	1.679E-01	-5.108E+00	1.462E-02	-3.506E+00	1.912E-01	5.029E-04
42716297	clusterin isoform 1	P10909	1191	CLU	5.619E-03	4.922E-04	-5.185E+00	9.038E-02	-5.289E+00	4.779E-02	-5.233E+00	3.615E-01	7.939E-01
17136078	VGF nerve growth factor inducible precursor	O15240	7425	VGF	4.740E-03	7.266E-04	-5.361E+00	1.543E-01	-6.722E+00	3.176E-01	-5.014E+00	2.353E-01	9.419E-04
19557645	secretogranin III	Q8WXD2	29106	SCG3	3.127E-03	4.251E-04	-5.775E+00	1.403E-01	-6.578E+00	2.241E-01	-5.784E+00	1.199E-01	5.520E-03
4505449	neuropeptide Y	P01303	4852	NPY	7.442E-03	2.233E-03	-4.939E+00	3.308E-01	-5.055E+00	3.400E-01	-2.938E+00	2.211E+00	1.645E-01
5453876	proenkephalin	P01210	5179	PENK	1.506E-03	4.763E-04	-6.547E+00	3.845E-01	-7.088E+00	3.057E-01	-5.474E+00	2.481E+00	3.129E-01
110347431	latent transforming growth factor beta binding protein 4 isoform a	Q8N2S1	8425	LTBP4	2.531E-04	7.975E-05	-8.332E+00	3.913E-01	-5.775E+00	4.553E+00	-7.777E+00	3.838E-01	4.596E-01
					1.996E-01	2.626E-02							
	**Protease Systems**												
7019519	proprotein convertase subtilisin/kexin type 1 inhibitor precursor	Q9UHG2	27344	PCSK1N	1.569E-02	3.870E-03	-4.179E+00	2.598E-01	-6.839E+00	1.768E-01	-3.576E+00	3.746E-01	1.949E-04
4503009	carboxypeptidase E precursor	P16870	1363	CPE	2.677E-02	3.195E-03	-3.626E+00	1.198E-01	-4.221E+00	1.540E-01	-3.977E+00	4.433E-01	4.550E-01
24307907	plasminogen activator inhibitor type 1, member 2	P07093	5270	SERPINE2	2.613E-02	1.249E-03	-3.646E+00	4.764E-02	-3.896E+00	3.723E-02	-4.875E+00	2.046E-01	1.790E-03
4507509	tissue inhibitor of metalloproteinase 1 precursor	P01033	7076	TIMP1	1.160E-02	4.028E-03	-4.502E+00	3.454E-01	-6.025E+00	1.867E-01	-4.112E+00	3.516E-01	1.052E-03
4503143	cathepsin D preproprotein	P07339	1509	CTSD	1.029E-02	1.579E-03	-4.586E+00	1.634E-01	-6.518E+00	2.528E-01	-4.076E+00	2.153E-01	7.178E-05
4503107	cystatin C precursor	P01034	1471	CST3	1.418E-02	1.773E-03	-4.262E+00	1.207E-01	-5.057E+00	2.369E-01	-4.415E+00	5.218E-01	1.866E-01
20336244	proprotein convertase subtilisin/kexin type 2	P16519	5126	PCSK2	1.383E-02	1.470E-03	-4.285E+00	1.063E-01	-4.559E+00	1.249E-01	-5.391E+00	2.136E-01	7.811E-03
4506917	secretory granule, neuroendocrine protein 1 (7B2 protein)	P05408	6447	SCG5	4.327E-03	8.837E-04	-5.460E+00	2.177E-01	-1.833E+00	0.000E+00	-4.778E+00	3.228E-01	3.591E-04
4505643	procollagen C-endopeptidase enhancer	Q15113	5118	PCOLCE	7.512E-03	9.861E-04	-4.898E+00	1.348E-01	-5.443E+00	5.177E-02	-5.312E+00	4.792E-01	6.494E-01
62420888	dipeptidyl peptidase 7 preproprotein	Q9UHL4	29952	DPP7	3.351E-03	1.042E-03	-5.733E+00	3.012E-01	-4.987E+00	3.652E+00	-5.099E+00	2.460E-01	9.562E-01
21070984	peptidylglycine alpha-amidating monooxygenase isoform a, preproprotein	P19021	5066	PAM	3.267E-03	1.542E-03	-5.804E+00	4.602E-01	-6.682E+00	2.055E-01	-5.899E+00	7.988E-01	1.708E-01
5729770	tripeptidyl-peptidase I preproprotein	O14773	1200	TPP1	4.029E-03	1.062E-03	-5.547E+00	3.114E-01	-6.195E+00	1.739E-01	-4.536E+00	1.806E+00	1.630E-01
7706387	plasma glutamate carboxypeptidase	Q9Y646	10404	PGCP	2.058E-03	1.082E-03	-6.382E+00	8.431E-01	-1.833E+00	0.000E+00	-5.700E+00	6.992E-01	1.582E-03
20336242	proprotein convertase subtilisin/kexin type 1 preproprotein	P29120	5122	PCSK1	4.425E-03	8.491E-04	-5.434E+00	1.883E-01	-5.585E+00	1.644E-01	-7.574E+00	4.624E-01	4.728E-03
117306169	prolylcarboxypeptidase isoform 2 preproprotein	P42785	5547	PRCP	1.747E-03	9.046E-04	-6.448E+00	5.071E-01	-6.263E+00	2.961E+00	-5.990E+00	4.279E-01	8.767E-01
113423966	PREDICTED: similar to Ubiquitin-63E CG11624-PA, isoform A	Q96C32	7316	UBC	2.714E-03	6.718E-04	-5.934E+00	2.605E-01	-5.315E+00	2.340E+00	-5.070E+00	2.464E+00	8.958E-01
4506141	HtrA serine peptidase 1	Q92743	5654	HTRA1	3.098E-03	5.209E-04	-5.787E+00	1.576E-01	-6.148E+00	2.094E-01	-5.281E+00	2.350E+00	4.989E-01
4505861	plasminogen activator, tissue type isoform 1 preproprotein	P00750	5327	PLAT	1.831E-03	5.425E-04	-6.334E+00	2.855E-01	-6.839E+00	3.367E-01	-6.763E+00	4.040E-01	7.283E-01
22538442	cathepsin Z preproprotein	Q9UBR2	1522	CTSZ	1.224E-03	6.345E-04	-6.872E+00	7.536E-01	-6.506E+00	3.116E+00	-4.943E+00	2.096E+00	5.027E-01
50363217	serine (or cysteine) proteinase inhibitor, clade A (alpha-1 antiproteinase)	P01009	5265	SERPINA1	6.234E-04	3.926E-04	-7.852E+00	1.479E+00	-3.469E+00	3.273E+00	-5.374E+00	2.363E+00	3.308E-01
4506145	protease, serine, 1 preproprotein	P07477	5644	PRSS1	4.504E-04	2.963E-04	-8.009E+00	1.068E+00	-1.833E+00	0.000E+00	-5.539E+00	2.479E+00	5.809E-02
113427619	PREDICTED: similar to Metalloproteinase inhibitor 2 precursor (TIMP-2)	P16035	7077	TIMP2	7.087E-04	4.079E-04	-7.366E+00	5.397E-01	-6.379E+00	3.031E+00	-5.607E+00	2.544E+00	7.560E-01
119395729	cathepsin A precursor	P10619	5476	CTSA	4.160E-04	4.121E-04	-8.072E+00	8.020E-01	-3.502E+00	3.340E+00	-5.905E+00	2.808E+00	4.881E-01
20149617	neural proliferation, differentiation and control, 1	Q9NQX5	56654	NPDC1	1.098E-03	8.845E-05	-6.816E+00	7.907E-02	-6.899E+00	1.045E-01	-1.833E+00	0.000E+00	2.417E-06
4826904	serine (or cysteine) proteinase inhibitor, clade I (neuroserpin), member 1	Q99574	5274	SERPINI1	3.104E-04	2.673E-04	-8.466E+00	1.152E+00	-3.481E+00	3.297E+00	-6.057E+00	2.828E+00	2.657E-01
21361091	ubiquitin carboxyl-terminal esterase L1 (ubiquitin thiolesterase)	P09936	7345	UCHL1	1.283E-03	1.340E-03	-6.997E+00	8.863E-01	-7.392E+00	3.811E-01	-2.743E+00	1.820E+00	2.108E-02
					1.630E-01	3.109E-02							
	**Neurotransmitters/Transporters**												
116534900	dopamine beta-hydroxylase precursor	P09172	1621	DBH	8.764E-02	7.426E-03	-2.437E+00	8.848E-02	-2.908E+00	1.217E-01	-2.894E+00	1.856E-01	9.156E-01
18379349	vesicle amine transport protein 1	Q99536	10493	VAT1	1.120E-02	3.566E-03	-4.539E+00	3.710E-01	-5.621E+00	1.676E-01	-4.408E+00	5.993E-01	3.086E-02
5803225	tyrosine 3/tryptophan 5 -monooxygenase activation protein, epsilon	P62258	7531	YWHAE	2.555E-03	1.053E-03	-6.046E+00	4.691E-01	-7.727E+00	3.780E-01	-5.754E+00	9.168E-01	5.531E-02
4757910	cocaine- and amphetamine-regulated transcript	Q16568	9607	CARTPT	4.707E-03	2.770E-03	-5.513E+00	6.632E-01	-6.111E+00	3.468E-01	-4.543E+00	1.944E+00	2.279E-01
88900501	tyrosine hydroxylase isoform a	P07101	7054	TH	2.743E-03	6.254E-04	-5.920E+00	2.462E-01	-6.694E+00	2.999E-01	-5.964E+00	3.692E-01	2.950E-02
14251209	chloride intracellular channel 1	O00299	1192	CLIC1	6.441E-04	4.786E-04	-7.748E+00	1.240E+00	-5.988E+00	2.789E+00	-3.188E+00	2.712E+00	1.868E-01
38679946	4-aminobutyrate aminotransferase precursor	P80404	18	ABAT	4.685E-04	1.046E-04	-7.683E+00	2.113E-01	-7.867E+00	4.681E-02	-3.350E+00	3.036E+00	5.959E-02
4759310	bestrophin	O76090	7439	BEST1	2.975E-04	1.439E-04	-8.212E+00	4.975E-01	-8.569E+00	3.057E-01	-4.950E+00	3.601E+00	1.532E-01
					1.103E-01	1.617E-02							
	**Receptors**												
5453916	progesterone receptor membrane component 2	O15173	10424	PGRMC2	1.343E-03	4.275E-04	-6.647E+00	2.948E-01	-6.730E+00	3.338E-01	-1.833E+00	0.000E+00	8.697E-05
					1.343E-03	4.275E-04							
	**Biochemical Processes:**												
	**Enzymes**												

7669492	glyceraldehyde-3-phosphate dehydrogenase	P04406	2597	GAPDH	5.697E-03	9.366E-04	-5.178E+00	1.637E-01	-5.751E+00	2.139E-01	-5.759E+00	9.038E-01	9.895E-01
6912618	glutaminyl-peptide cyclotransferase precursor	Q16769	25797	QPCT	2.928E-03	5.385E-04	-5.845E+00	1.750E-01	-6.234E+00	1.780E-01	-5.167E+00	2.224E+00	3.998E-01
4507645	triosephosphate isomerase 1	P60174	7167	TPI1	1.853E-03	5.738E-04	-6.329E+00	3.184E-01	-7.363E+00	5.799E-01	-4.865E+00	2.030E+00	5.507E-02
5174539	cytosolic malate dehydrogenase	P40925	4190	MDH1	1.056E-03	4.553E-04	-6.915E+00	3.922E-01	-1.833E+00	0.000E+00	-6.233E+00	5.270E-01	4.674E-04
4758504	hydroxysteroid (17-beta) dehydrogenase 10 isoform 1	Q99714	3028	HSD17B10	3.905E-03	1.379E-03	-5.596E+00	3.723E-01	-5.678E+00	3.837E-01	-1.833E+00	0.000E+00	2.714E-04
24850115	cytosolic sialic acid 9-O-acetylesterase homolog	Q9HAT2	54414	SIAE	8.980E-04	2.183E-04	-7.039E+00	2.556E-01	-7.972E+00	4.332E-01	-6.962E+00	2.689E-01	1.791E-02
4506031	palmitoyl-protein thioesterase 1 (ceroid-lipofuscinosis, neuronal 1)	P50897	5538	PPT1	6.050E-04	4.022E-04	-7.814E+00	1.305E+00	-1.833E+00	0.000E+00	-5.380E+00	2.373E+00	5.818E-02
19923106	paraoxonase 1	P27169	5444	PON1	1.945E-03	5.128E-04	-6.270E+00	2.779E-01	-6.386E+00	1.979E-01	-3.230E+00	2.795E+00	1.187E-01
20070125	prolyl 4-hydroxylase, beta subunit precursor	P07237	5034	P4HB	6.737E-04	4.605E-04	-7.493E+00	7.377E-01	-8.052E+00	3.190E-01	-5.973E+00	2.892E+00	2.838E-01
15991829	hexokinase 1 isoform HKI-ta/tb	P19367	3098	HK1	3.933E-04	1.834E-04	-7.981E+00	6.959E-01	-1.833E+00	0.000E+00	-7.299E+00	7.346E-01	6.582E-04
7705925	dicarbonyl/L-xylulose reductase	Q7Z4W1	51181	DCXR	1.458E-03	6.644E-04	-6.660E+00	6.605E-01	-6.742E+00	6.645E-01	-1.833E+00	0.000E+00	6.724E-04
4503571	enolase 1	P06733	2023	ENO1	8.053E-04	3.035E-04	-7.174E+00	3.559E-01	-7.688E+00	4.681E-02	-5.967E+00	2.805E+00	3.076E-01
9951923	carbonic anhydrase XI precursor	O75493	770	CA11	1.299E-03	1.000E-04	-6.649E+00	7.883E-02	-6.731E+00	4.681E-02	-1.833E+00	0.000E+00	2.405E-07
4557894	lysozyme precursor	P61626	4069	LYZ	1.234E-03	3.317E-04	-6.730E+00	3.118E-01	-6.813E+00	3.546E-01	-1.833E+00	0.000E+00	9.909E-05
38327625	citrate synthase precursor, isoform a	O75390	1431	CS	1.133E-03	2.543E-04	-6.801E+00	2.213E-01	-6.956E+00	3.046E-01	-3.328E+00	2.992E+00	7.679E-02
12707570	mitochondrial short-chain enoyl-coenzyme A hydratase 1 precursor	P30084	1892	ECHS1	1.017E-03	2.943E-04	-6.923E+00	2.925E-01	-7.005E+00	3.158E-01	-1.833E+00	0.000E+00	6.254E-05
32307144	lysyl hydroxylase precursor	Q02809	5351	PLOD1	9.908E-04	5.062E-04	-7.028E+00	5.679E-01	-7.156E+00	5.397E-01	-3.416E+00	3.167E+00	9.593E-02
7705855	hydroxysteroid (17-beta) dehydrogenase 12	Q53GQ0	51144	HSD17B12	5.953E-04	1.629E-04	-7.461E+00	3.180E-01	-7.543E+00	3.190E-01	-1.833E+00	0.000E+00	4.792E-05
9955948	carbonic anhydrase IX precursor	Q16790	768	CA9	4.375E-04	4.130E-04	-8.290E+00	1.449E+00	-6.320E+00	3.029E+00	-3.286E+00	2.908E+00	2.017E-01
4501867	aconitase 2 precursor	Q99798	50	ACO2	4.483E-04	3.174E-04	-7.925E+00	7.893E-01	-8.180E+00	5.963E-01	-4.893E+00	3.553E+00	2.034E-01
19743875	fumarate hydratase precursor	P07954	2271	FH	3.952E-04	2.577E-04	-8.282E+00	1.411E+00	-6.318E+00	2.991E+00	-3.342E+00	3.019E+00	1.850E-01
23308751	3-hydroxyisobutyrate dehydrogenase	P31937	11112	HIBADH	3.416E-04	2.474E-04	-8.294E+00	1.052E+00	-6.365E+00	3.042E+00	-1.833E+00	0.000E+00	5.863E-02
					3.011E-02	9.514E-03							
	**Carbohydrate Functions**												
4504349	beta globin	P68871	3043	HBB	2.542E-02	4.679E-03	-3.687E+00	2.005E-01	-4.202E+00	8.591E-02	-4.451E+00	1.214E+00	7.166E-01
4557781	alpha-N-acetylgalactosaminidase precursor	P17050	4668	NAGA	1.420E-03	1.035E-03	-7.246E+00	1.890E+00	-1.833E+00	0.000E+00	-4.735E+00	1.948E+00	5.865E-02
5802984	UDP-GlcNAc:betaGal beta-1,3-N-acetylglucosaminyltransferase 1	O43505	11041	B3GNT1	2.104E-03	9.670E-04	-6.250E+00	4.881E-01	-6.456E+00	4.013E-01	-5.946E+00	2.763E+00	7.570E-01
119393891	acid alpha-glucosidase preproprotein	P10253	2548	GAA	8.071E-04	2.641E-05	-7.122E+00	3.296E-02	-1.833E+00	0.000E+00	-6.440E+00	1.775E-01	1.574E-05
4504373	hexosaminidase B preproprotein	P07686	3074	HEXB	9.218E-04	2.275E-04	-7.017E+00	2.841E-01	-5.088E+00	3.773E+00	-6.482E+00	3.352E-01	5.057E-01
4504223	glucuronidase, beta	P08236	2990	GUSB	2.342E-04	8.658E-05	-8.412E+00	3.766E-01	-1.833E+00	0.000E+00	-7.730E+00	3.497E-01	5.732E-05
4504061	glucosamine (N-acetyl)-6-sulfatase precursor	P15586	2799	GNS	5.686E-04	4.379E-05	-7.475E+00	7.883E-02	-1.833E+00	0.000E+00	-6.792E+00	2.119E-01	2.147E-05
119372308	galactosidase, beta 1 isoform a	P16278	2720	GLB1	3.886E-04	1.035E-04	-7.881E+00	2.746E-01	-1.833E+00	0.000E+00	-7.198E+00	3.849E-01	1.012E-04
38202257	alpha glucosidase II alpha subunit isoform 2	Q14697	23193	GANAB	4.715E-04	1.312E-04	-7.690E+00	2.903E-01	-8.577E+00	4.588E-01	-7.640E+00	4.045E-01	4.090E-02
4505167	alpha-1,3(6)-mannosylglycoprotein beta-1,6-N-acetyl-glucosaminyltransferase	Q09328	4249	MGAT5	2.154E-04	1.783E-04	-8.892E+00	1.303E+00	-6.740E+00	3.299E+00	-1.833E+00	0.000E+00	5.885E-02
51873064	mannosidase, alpha, class 2B, member 1 precursor	O00754	4125	MAN2B1	2.850E-04	1.620E-04	-8.312E+00	6.619E-01	-3.683E+00	3.701E+00	-7.702E+00	5.044E-01	1.289E-01
54633312	golgi apparatus protein 1	Q92896	2734	GLG1	1.523E-04	5.064E-05	-8.846E+00	4.196E-01	-1.833E+00	0.000E+00	-8.164E+00	5.077E-01	1.413E-04
4758412	polypeptide N-acetylgalactosaminyltransferase 2	Q10471	2590	GALNT2	3.602E-04	1.432E-04	-7.994E+00	4.271E-01	-8.076E+00	4.697E-01	-1.833E+00	0.000E+00	1.168E-04
24497519	mannosidase, alpha, class 1A, member 1	P33908	4121	MAN1A1	4.041E-04	1.674E-04	-7.911E+00	5.614E-01	-8.094E+00	4.900E-01	-3.382E+00	3.099E+00	5.794E-02
47419930	chondroitin sulfate proteoglycan 4	Q6UVK1	1464	CSPG4	3.658E-05	1.937E-05	-1.043E+01	8.827E-01	-1.833E+00	0.000E+00	-7.428E+00	3.736E+00	5.789E-02
51477716	mannosidase, alpha, class 2A, member 2	P49641	4122	MAN2A2	2.108E-04	1.587E-04	-8.980E+00	1.496E+00	-6.719E+00	3.274E+00	-1.833E+00	0.000E+00	5.837E-02
4502841	carbohydrate (keratan sulfate Gal-6) sulfotransferase 1	O43916	8534	CHST1	1.957E-04	1.032E-04	-8.747E+00	8.768E-01	-6.735E+00	3.268E+00	-1.833E+00	0.000E+00	5.768E-02
					3.419E-02	8.283E-03							
	**Lipid Functions**												
153266841	apolipoprotein H precursor	P02749	350	APOH	3.226E-03	8.882E-04	-5.762E+00	2.512E-01	-6.629E+00	2.127E-01	-5.797E+00	5.155E-01	7.320E-02
39995109	GM2 ganglioside activator precursor	P17900	2760	GM2A	6.911E-04	2.146E-04	-7.308E+00	2.767E-01	-1.833E+00	0.000E+00	-6.626E+00	2.689E-01	4.855E-05
110224476	prosaposin isoform b preproprotein	P07602	5660	PSAP	1.261E-03	1.756E-04	-6.683E+00	1.365E-01	-7.226E+00	1.907E-01	-7.037E+00	3.030E-01	3.949E-01
23618867	sideroflexin 1	Q9H9B4	94081	SFXN1	1.240E-03	2.012E-04	-6.703E+00	1.697E-01	-6.786E+00	1.342E-01	-1.833E+00	0.000E+00	5.476E-06
13129148	apolipoprotein O	Q9BUR5	79135	APOO	1.034E-03	3.551E-04	-6.927E+00	3.950E-01	-7.010E+00	4.437E-01	-1.833E+00	0.000E+00	1.724E-04
4557237	acetyl-Coenzyme A acetyltransferase 1 precursor	P24752	38	ACAT1	9.036E-04	9.739E-05	-7.013E+00	1.061E-01	-7.197E+00	3.236E-01	-3.196E+00	2.727E+00	4.579E-02
55770862	thyroglobulin	P01266	7038	TG	1.077E-04	9.971E-05	-9.727E+00	1.552E+00	-7.392E+00	3.735E+00	-5.702E+00	4.469E+00	4.624E-01
30089930	N-acylsphingosine amidohydrolase (acid ceramidase) 1 isoform b	Q13510	427	ASAH1	5.772E-03	1.599E-03	-5.185E+00	2.877E-01	-6.358E+00	3.177E-01	-4.938E+00	2.560E-01	3.591E-03
5453678	epididymal secretory protein E1 precursor	P61916	10577	NPC2	3.041E-03	8.464E-04	-5.826E+00	2.903E-01	-4.579E+00	3.171E+00	-5.301E+00	5.063E-01	6.681E-01
4557321	apolipoprotein A-I preproprotein	P02647	335	APOA1	2.241E-03	7.408E-04	-6.143E+00	3.337E-01	-3.357E+00	3.048E+00	-5.499E+00	2.408E-01	2.551E-01
					1.952E-02	5.218E-03							
	**Internal Conditions of Secretory Vesicles:**												
	**Reduction-Oxidation**												
6912238	peroxiredoxin 5 precursor, isoform a	P30044	25824	PRDX5	7.674E-03	1.782E-03	-4.892E+00	2.522E-01	-4.975E+00	2.294E-01	-1.833E+00	0.000E+00	1.068E-04
4502989	cytochrome c oxidase subunit VIIa polypeptide 2 (liver) precursor	P14406	1347	COX7A2	4.895E-03	2.305E-03	-5.443E+00	6.332E-01	-5.106E+00	6.252E-01	-1.833E+00	0.000E+00	1.859E-03

6006001	plasma glutathione peroxidase 3 precursor	P22352	2878	GPX3	3.698E-03	3.901E-04	-5.604E+00	1.055E-01	-6.163E+00	1.619E-01	-5.899E+00	2.119E-01	1.621E-01
32189392	peroxiredoxin 2 isoform a	P32119	7001	PRDX2	3.587E-03	6.972E-04	-5.645E+00	1.972E-01	-6.059E+00	1.042E-01	-4.907E+00	2.065E+00	3.309E-01
4502987	cytochrome c oxidase subunit VIIa polypeptide 1 (muscle) precursor	P24310	1346	COX7A1	3.800E-03	1.750E-03	-5.665E+00	5.167E-01	-5.329E+00	5.201E-01	-1.833E+00	0.000E+00	8.899E-04
4502981	cytochrome c oxidase subunit IV isoform 1 precursor	P13073	1327	COX4I1	3.431E-03	3.592E-04	-5.679E+00	1.020E-01	-5.342E+00	1.196E-01	-1.833E+00	0.000E+00	1.089E-05
4505355	NADH dehydrogenase (ubiquinone) 1 alpha subcomplex, 2, 8kDa	O43678	4695	NDUFA2	4.882E-03	1.354E-03	-5.348E+00	2.537E-01	-5.430E+00	2.031E-01	-1.833E+00	0.000E+00	4.948E-05
17017988	cytochrome c oxidase subunit Vb precursor	P10606	1329	COX5B	2.856E-03	9.591E-04	-5.899E+00	3.232E-01	-5.562E+00	2.960E-01	-1.833E+00	0.000E+00	1.370E-04
63054828	cytochrome b-561	P49447	1534	CYB561	1.255E-03	8.615E-05	-6.682E+00	6.769E-02	-5.624E+00	2.538E+00	-6.402E+00	6.609E-01	5.063E-01
4506359	quinoid dihydropteridine reductase	A8K158	5860	QDPR	1.368E-03	1.054E-04	-6.597E+00	7.883E-02	-5.951E+00	2.765E+00	-6.565E+00	6.272E-01	6.600E-01
4505361	NADH dehydrogenase (ubiquinone) 1 beta subcomplex, 3, 12kDa	O43676	4709	NDUFB3	2.149E-03	4.347E-04	-6.157E+00	1.911E-01	-5.790E+00	1.772E-01	-1.833E+00	0.000E+00	2.471E-05
4758774	NADH dehydrogenase (ubiquinone) 1 beta subcomplex, 10, 22kDa	O96000	4716	NDUFB10	2.158E-03	6.151E-04	-6.172E+00	3.060E-01	-5.806E+00	2.771E-01	-1.833E+00	0.000E+00	9.312E-05
17981856	cytochrome c oxidase subunit II	P00403	4513	MT-CO2	1.905E-03	4.947E-04	-6.287E+00	2.456E-01	-5.950E+00	1.882E-01	-1.833E+00	0.000E+00	2.625E-05
5454152	ubiquinol-cytochrome c reductase binding protein	P14927	7381	UQCRB	2.838E-03	5.794E-04	-5.879E+00	1.933E-01	-5.961E+00	1.772E-01	-1.833E+00	0.000E+00	2.176E-05
4758788	NADH dehydrogenase (ubiquinone) Fe-S protein 3, 30kDa	O75489	4722	NDUFS3	1.800E-03	2.751E-04	-6.329E+00	1.518E-01	-5.995E+00	1.171E-01	-3.096E+00	2.526E+00	1.055E-01
4505369	NADH dehydrogenase (ubiquinone) Fe-S protein 4, 18kDa	O43181	4724	NDUFS4	1.315E-03	6.714E-04	-6.743E+00	5.509E-01	-6.377E+00	5.699E-01	-1.833E+00	0.000E+00	5.360E-04
4758790	NADH dehydrogenase (ubiquinone) Fe-S protein 5, 15kDa	O43920	4725	NDUFS5	1.664E-03	3.917E-04	-6.423E+00	2.702E-01	-6.057E+00	2.453E-01	-1.833E+00	0.000E+00	5.379E-05
6274550	NADH dehydrogenase (ubiquinone) 1 beta subcomplex, 9, 22kDa	Q9Y6M9	4715	NDUFB9	1.628E-03	1.475E-04	-6.423E+00	9.022E-02	-6.057E+00	1.255E-01	-1.833E+00	0.000E+00	7.228E-06
7657369	NADH dehydrogenase (ubiquinone) 1 alpha subcomplex, 8, 19kDa	P51970	4702	NDUFA8	1.580E-03	3.466E-04	-6.469E+00	2.236E-01	-6.102E+00	2.048E-01	-1.833E+00	0.000E+00	3.037E-05
4502993	cytochrome c oxidase subunit VIIc precursor	P15954	1350	COX7C	2.352E-03	1.343E-03	-6.150E+00	4.786E-01	-6.233E+00	5.082E-01	-1.833E+00	0.000E+00	4.196E-04
4758772	NADH dehydrogenase (ubiquinone) 1 alpha subcomplex, 3, 9kDa	O95167	4696	NDUFA3	1.406E-03	3.852E-04	-6.601E+00	3.187E-01	-6.235E+00	3.190E-01	-1.833E+00	0.000E+00	1.044E-04
4505365	NADH dehydrogenase (ubiquinone) 1 beta subcomplex, 6, 17kDa isoform 1	O95139	4712	NDUFB6	1.343E-03	5.170E-04	-6.666E+00	3.716E-01	-6.299E+00	3.795E-01	-1.833E+00	0.000E+00	1.679E-04
5174743	ubiquinol-cytochrome c reductase, Rieske iron-sulfur polypeptide 1	P0C7P4	7386	UQCRFS1 U	2.059E-03	5.043E-04	-6.207E+00	2.345E-01	-6.289E+00	2.764E-01	-1.833E+00	0.000E+00	6.554E-05
4826854	NADH dehydrogenase (ubiquinone) 1 beta subcomplex, 8, 19kDa	O95169	4714	NDUFB8	1.277E-03	3.781E-04	-6.705E+00	3.541E-01	-6.339E+00	3.615E-01	-1.833E+00	0.000E+00	1.416E-04
21359867	cytochrome c-1	P08574	1537	CYC1	2.001E-03	3.686E-04	-6.226E+00	1.812E-01	-6.309E+00	1.314E-01	-1.833E+00	0.000E+00	6.966E-06
7705704	glutathione transferase kappa 1	Q9Y2Q3	373156	GSTK1	1.141E-03	3.527E-04	-6.339E+00	3.755E-01	-6.422E+00	3.334E-01	-1.833E+00	0.000E+00	1.052E-04
51317370	NADH dehydrogenase (ubiquinone) 1 alpha subcomplex, 6, 14kDa	P56556	4700	NDUFA6	1.241E-03	1.314E-04	-6.809E+00	2.884E-01	-6.442E+00	3.338E-01	-1.833E+00	0.000E+00	1.042E-04
4503327	cytochrome b5 reductase isoform 1	P00387	1727	CYB5R3	1.340E-03	5.351E-04	-6.696E+00	1.041E-01	-6.415E+00	1.825E-01	-3.225E+00	2.786E+00	9.382E-02
4826852	NADH dehydrogenase (ubiquinone) 1, alpha/beta subcomplex, 1, 8kDa	O14561	4706	NDUFAB1	1.598E-03	4.348E-04	-6.681E+00	4.317E-01	-6.763E+00	4.434E-01	-1.833E+00	0.000E+00	1.990E-04
5802974	peroxiredoxin 3 isoform a precursor	P30048	10935	PRDX3	1.672E-03	2.631E-04	-6.468E+00	2.763E-01	-6.550E+00	2.662E-01	-1.833E+00	0.000E+00	4.938E-05
4503607	electron transfer flavoprotein, alpha polypeptide	P13804	2108	ETFA	1.500E-03	3.501E-04	-6.403E+00	1.563E-01	-6.541E+00	1.956E-01	-3.133E+00	2.600E+00	7.186E-02
4504183	glutathione transferase	P09211	2950	GSTP1	1.387E-03	5.128E-04	-6.526E+00	2.636E-01	-6.985E+00	5.309E-01	-5.593E+00	2.512E+00	2.769E-01
18105037	cytochrome c oxidase subunit VIIa polypeptide 2 like	O14548	9167	COX7A2L	8.719E-04	1.488E-04	-6.633E+00	3.766E-01	-6.715E+00	3.925E-01	-1.833E+00	0.000E+00	1.424E-04
38524585	NADH-ubiquinone oxidoreductase Fe-S protein 7	P28331	4719	NDUFS1	8.334E-04	3.185E-04	-7.057E+00	1.807E-01	-6.690E+00	1.768E-01	-1.833E+00	0.000E+00	1.329E-05
4826848	NADH dehydrogenase (ubiquinone) 1 alpha subcomplex, 5	Q16718	4698	NDUFA5	6.833E-04	1.513E-04	-7.147E+00	3.904E-01	-6.780E+00	3.606E-01	-1.833E+00	0.000E+00	1.062E-04
10835025	NADH dehydrogenase (ubiquinone) flavoprotein 2, 24kDa	P05857	4729	NDUFV2	6.290E-04	2.409E-04	-7.307E+00	2.204E-01	-6.940E+00	2.284E-01	-1.833E+00	0.000E+00	2.459E-05
28269681	NADH dehydrogenase (ubiquinone) 1 alpha subcomplex, 11, 14.7kDa	Q86Y39	126328	NDUFA11	5.696E-04	2.181E-04	-7.418E+00	3.393E-01	-7.052E+00	3.661E-01	-1.833E+00	0.000E+00	9.475E-05
10764847	NADH dehydrogenase (ubiquinone) 1 beta subcomplex, 7, 18kDa	P17568	4713	NDUFB7	3.631E-03	5.428E-04	-7.518E+00	3.393E-01	-7.151E+00	3.661E-01	-1.833E+00	0.000E+00	8.956E-05
11128019	cytochrome c	P99999	54205	CYCS	7.398E-04	4.972E-04	-5.627E+00	1.522E-01	-5.575E+00	3.055E-01	-4.853E+00	2.029E+00	4.821E-01
4503301	2,4-dienoyl CoA reductase 1 precursor	Q16698	1666	DECR1	4.480E-04	4.032E-04	-7.409E+00	7.519E-01	-7.491E+00	7.659E-01	-1.833E+00	0.000E+00	6.725E-04
4505363	NADH dehydrogenase (ubiquinone) 1 beta subcomplex, 5, 16kDa precursor	O43674	4711	NDUFB5	6.688E-04	2.638E-04	-8.157E+00	1.259E+00	-5.873E+00	2.744E+00	-1.833E+00	0.000E+00	6.025E-02
41406084	glutathione peroxidase 1 isoform 1	P07203	2876	GPX1	4.268E-04	2.260E-04	-7.360E+00	3.489E-01	-7.442E+00	3.661E-01	-1.833E+00	0.000E+00	7.637E-05
4758792	NADH dehydrogenase (ubiquinone) Fe-S protein 6, 13kDa	O75380	4726	NDUFS6	7.675E-04	1.091E-04	-7.970E+00	8.838E-01	-5.828E+00	2.664E+00	-1.833E+00	0.000E+00	5.768E-02
49574502	NAD(P)H:quinone oxidoreductase type 3, polypeptide A2	Q9UHQ9	51706	CYB5R1	2.809E-03	1.046E-03	-7.179E+00	1.349E-01	-7.536E+00	3.546E-01	-4.541E+00	3.133E+00	1.328E-01
38569473	NADH dehydrogenase (ubiquinone) 1 beta subcomplex, 1, 7kDa	O75438	4707	NDUFB1	2.710E-04	2.177E-04	-5.945E+00	4.657E-01	-5.579E+00	4.735E-01	-1.833E+00	0.000E+00	5.487E-04
6681764	NADH dehydrogenase (ubiquinone) 1 alpha subcomplex, 9, 39kDa	Q16795	4704	NDUFA9	2.469E-04	1.307E-04	-8.653E+00	1.280E+00	-6.229E+00	2.972E+00	-1.833E+00	0.000E+00	5.959E-02
4505371	NADH dehydrogenase (ubiquinone) Fe-S protein 8, 23kDa	O00217	4728	NDUFS8	7.674E-03	1.782E-03	-8.517E+00	8.838E-01	-6.239E+00	2.938E+00	-1.833E+00	0.000E+00	5.768E-02
					9.607E-02	2.612E-02							
	**ATPases and Nucleotide Metabolism**												
6005717	ATP synthase, H+ transporting, mitochondrial F0 complex, subunit E	P56385	521	ATP5I	1.655E-02	5.382E-03	-4.148E+00	3.713E-01	-4.270E+00	3.547E-01	-3.910E+00	2.399E+00	8.010E-01
51479156	ATP synthase, H+ transporting, mitochondrial F0 complex, subunit G	O75964	10632	ATP5L	1.208E-02	8.488E-04	-4.418E+00	7.031E-02	-4.500E+00	3.879E-02	-1.833E+00	0.000E+00	8.477E-07
19913432	ATPase, H+ transporting, lysosomal, V0 subunit d1	P61421	9114	ATP6V0D1	4.676E-03	8.345E-04	-5.378E+00	1.847E-01	-5.814E+00	1.654E-01	-4.772E+00	1.994E+00	3.392E-01
8922601	ADP-ribosylation factor-like 10C	Q9NVJ2	55207	ARL8B	3.521E-03	1.199E-03	-5.697E+00	3.641E-01	-6.501E+00	7.102E-01	-4.532E+00	1.800E+00	1.073E-01
4502317	vacuolar H+ ATPase E1 isoform a	P36543	529	ATP6V1E1	4.491E-03	5.432E-04	-5.411E+00	1.169E-01	-5.778E+00	4.373E-02	-6.130E+00	3.508E-01	1.056E-01
17981857	ATP synthase F0 subunit 8	P00846	4508	MT-ATP6 M	6.213E-03	2.338E-03	-5.153E+00	4.715E-01	-5.235E+00	4.513E-01	-1.833E+00	0.000E+00	6.330E-04
15011918	ATPase, H+ transporting, lysosomal accessory protein 2	O75787	10159	ATP6AP2	5.007E-03	1.245E-03	-5.324E+00	2.773E-01	-5.466E+00	2.494E-01	-5.828E+00	2.689E+00	8.196E-01
4502201	ADP-ribosylation factor 1	P84077	375	ARF1	2.851E-03	9.034E-04	-5.893E+00	2.837E-01	-6.285E+00	3.780E-01	-6.619E+00	2.689E-01	3.151E-01
4502303	mitochondrial ATP synthase, O subunit precursor	P48047	539	ATP5O	4.663E-03	1.760E-04	-5.369E+00	3.776E-02	-5.451E+00	2.416E-02	-1.833E+00	0.000E+00	8.207E-08
4757818	vacuolar H+ ATPase G1	O75348	9550	ATP6V1G1	4.711E-03	1.240E-03	-5.383E+00	2.569E-01	-5.549E+00	1.842E-01	-2.792E+00	1.918E+00	6.734E-02
21361565	ATP synthase, H+ transporting, mitochondrial F0 complex, B1 precursor	P24539	515	ATP5F1	4.223E-03	1.090E-03	-5.492E+00	2.583E-01	-5.574E+00	3.060E-01	-1.833E+00	0.000E+00	1.499E-04

19913428	vacuolar H+ATPase B2	P21281	526	ATP6V1B2	2.372E-03	4.564E-04	-6.059E+00	2.026E-01	-6.363E+00	2.863E-01	-7.074E+00	4.692E-01	1.092E-01
4507879	voltage-dependent anion channel 1	P21796	7416	VDAC1	3.650E-03	7.066E-04	-5.627E+00	1.937E-01	-5.709E+00	1.562E-01	-1.833E+00	0.000E+00	1.801E-05
19913424	ATPase, H+ transporting, lysosomal 70kD, V1 subunit A, isoform 1	P38606	523	ATP6V1A	2.289E-03	6.411E-04	-6.110E+00	2.897E-01	-6.453E+00	2.936E-01	-6.917E+00	3.418E-01	5.337E-02
32189394	ATP synthase, H+ transporting, mitochondrial F1 complex, beta precursor	P06576	506	ATP5B	2.495E-03	3.412E-04	-6.001E+00	1.410E-01	-6.083E+00	1.160E-01	-1.833E+00	0.000E+00	5.597E-06
5453559	ATP synthase, H+ transporting, mitochondrial F0 complex, d isoform a	O75947	10476	ATP5H	2.207E-03	5.932E-04	-6.149E+00	3.118E-01	-6.231E+00	3.546E-01	-1.833E+00	0.000E+00	1.436E-04
19913418	ATPase, H+ transporting, lysosomal V0, a isoform 1	Q93050	535	ATP6V0A1	9.233E-04	4.352E-04	-7.097E+00	5.805E-01	-5.899E+00	2.711E+00	-7.474E+00	5.984E-01	3.248E-01
4757812	ATP synthase, H+ transporting, mitochondrial F0 complex, F2 isoform 2a	P56134	9551	ATP5J2	6.158E-03	7.740E-04	-5.096E+00	1.269E-01	-5.178E+00	1.169E-01	-1.833E+00	0.000E+00	1.175E-05
42476281	voltage-dependent anion channel 2	P45880	7417	VDAC2	1.309E-03	2.611E-04	-6.654E+00	2.058E-01	-6.737E+00	2.078E-01	-1.833E+00	0.000E+00	2.094E-05
7706757	H(+)-transporting two-sector ATPase	Q9Y5K8	51382	ATP6V1D	9.346E-04	4.135E-04	-7.064E+00	5.077E-01	-7.146E+00	5.414E-01	-1.833E+00	0.000E+00	2.890E-04
4757810	ATP synthase, H+ transporting, mitochondrial F1 complex, alpha precursor	P25705	498	ATP5A1	8.372E-04	3.715E-04	-7.153E+00	4.131E-01	-7.319E+00	2.843E-01	-3.191E+00	2.717E+00	6.779E-02
47717102	ATPase, H+ transporting, lysosomal 50/57kDa, V1 subunit H isoform 2	Q9UI12	51606	ATP6V1H	3.687E-04	2.122E-04	-8.020E+00	5.397E-01	-6.704E+00	3.268E+00	-6.369E+00	3.026E+00	9.062E-01
4502277	Na+/K+ -ATPase beta 1 subunit isoform a	P05026	481	ATP1B1	5.956E-04	4.127E-04	-7.839E+00	1.312E+00	-5.898E+00	2.720E+00	-1.833E+00	0.000E+00	5.814E-02
4502315	ATPase, H+ transporting, lysosomal 42kDa, V1 subunit C1	P21283	528	ATP6V1C1	6.061E-04	1.643E-04	-7.433E+00	2.491E-01	-7.790E+00	3.888E-01	-4.734E+00	3.351E+00	1.475E-01
50345988	ATP synthase, H+ transporting, mitochondrial F1 complex, gamma precursor	P36542	509	ATP5C1	2.770E-04	1.461E-04	-8.399E+00	8.768E-01	-6.474E+00	3.095E+00	-1.833E+00	0.000E+00	5.768E-02
17136148	ATPase, H+ transporting, lysosomal accessory protein 1 precursor	Q15904	537	ATP6AP1	1.793E-04	9.723E-05	-8.855E+00	9.262E-01	-6.824E+00	3.328E+00	-1.833E+00	0.000E+00	5.768E-02
38569421	ATP citrate lyase isoform 1	P53396	47	ACLY	1.230E-04	5.518E-05	-9.066E+00	3.893E-01	-9.321E+00	4.681E-02	-3.508E+00	3.351E+00	4.128E-02
17978471	ATPase, aminophospholipid transporter (APLT), class I, type 8A, member 1	Q9Y2Q0	10396	ATP8A1	6.975E-05	3.694E-05	-9.781E+00	8.827E-01	-7.523E+00	3.794E+00	-1.833E+00	0.000E+00	5.768E-02
					9.439E-02	2.192E-02							
	**Protein Folding**												
4758950	peptidylprolyl isomerase B precursor	P23284	5479	PPIB	1.086E-02	7.327E-04	-4.525E+00	6.835E-02	-4.738E+00	2.372E-02	-6.034E+00	6.193E-01	2.410E-02
5453832	oxygen regulated protein precursor	Q9Y4L1	10525	HYOU1	8.034E-04	4.026E-04	-7.247E+00	6.055E-01	-5.910E+00	2.725E+00	-7.854E+00	5.626E-01	2.169E-01
16507237	heat shock 70kDa protein 5	P11021	3309	HSPA5	3.360E-03	9.103E-04	-5.724E+00	2.780E-01	-6.137E+00	3.950E-01	-6.432E+00	4.769E-01	4.208E-01
4504523	heat shock 10kDa protein 1 (chaperonin 10)	P61604	3336	HSPE1	3.538E-03	1.171E-03	-5.695E+00	3.892E-01	-5.778E+00	3.742E-01	-1.833E+00	0.000E+00	2.334E-04
21361657	protein disulfide isomerase-associated 3 precursor	P30101	2923	PDIA3	2.099E-03	7.836E-04	-6.218E+00	3.725E-01	-6.529E+00	3.426E-01	-5.806E+00	2.753E+00	6.023E-01
4504517	heat shock 27kDa protein 1	P04792	3315	HSPB1	2.426E-03	5.382E-04	-6.043E+00	2.522E-01	-6.126E+00	2.618E-01	-1.833E+00	0.000E+00	6.230E-05
31542947	chaperonin	P10809	3329	HSPD1	1.058E-03	2.557E-04	-6.873E+00	2.437E-01	-6.955E+00	2.694E-01	-1.833E+00	0.000E+00	3.997E-05
24234688	heat shock 70kDa protein 9 precursor	P38646	3313	HSPA9	8.783E-04	3.835E-04	-7.150E+00	6.117E-01	-7.233E+00	5.898E-01	-1.833E+00	0.000E+00	3.553E-04
5031973	protein disulfide isomerase-associated 6	Q15084	10130	PDIA6	9.274E-04	2.926E-04	-7.020E+00	3.135E-01	-7.204E+00	4.350E-01	-3.334E+00	3.002E+00	6.056E-02
					2.595E-02	5.470E-03							
	**Regulated Secretion Mechanisms:**												
	**Signal Transduction and GTP-Binding Proteins**												
9845509	ras-related C3 botulinum toxin substrate 1 isoform Rac1b	P63000	5879	RAC1	4.153E-03	7.206E-04	-5.495E+00	1.751E-01	-5.999E+00	2.694E-01	-4.668E+00	1.938E+00	2.337E-01
5174447	guanine nucleotide binding protein (G protein), beta polypeptide 2-like 1	P63244	10399	GNB2L1	2.107E-03	4.399E-04	-6.179E+00	2.124E-01	-1.833E+00	0.000E+00	-5.497E+00	3.396E-01	2.178E-04
51036603	G-protein gamma-12 subunit	Q9UBI6	55970	GNG12	6.565E-03	1.668E-03	-5.053E+00	2.787E-01	-5.136E+00	2.620E-01	-1.833E+00	0.000E+00	1.367E-04
17149842	FK506-binding protein 2 precursor	P26885	2286	FKBP2	5.388E-03	7.113E-04	-5.230E+00	1.325E-01	-5.312E+00	1.804E-01	-1.833E+00	0.000E+00	3.833E-05
32698769	guanine nucleotide binding protein (G protein), gamma 7	O60262	2788	GNG7	5.131E-03	2.215E-03	-5.338E+00	4.103E-01	-5.420E+00	3.890E-01	-1.833E+00	0.000E+00	3.478E-04
19923437	adenylate kinase 3	Q9UIJ7	50808	AK3	4.271E-03	7.880E-04	-5.468E+00	1.782E-01	-5.550E+00	1.537E-01	-1.833E+00	0.000E+00	1.944E-05
4505763	phosphoglycerate kinase 1	P00558	5230	PGK1	1.318E-03	2.448E-04	-6.644E+00	1.761E-01	-7.256E+00	2.741E-01	-5.478E+00	2.445E+00	2.120E-01
45267837	rabphilin 3A homolog	Q9Y2J0	22895	RPH3A	2.415E-03	1.676E-04	-6.028E+00	6.892E-02	-6.197E+00	1.106E-01	-6.263E+00	2.994E+00	9.676E-01
11386149	protein tyrosine phosphatase, receptor type, N, 2 isoform 1 precursor	Q7KZN9	1355	COX15	1.134E-03	2.728E-04	-6.808E+00	2.775E-01	-6.978E+00	2.029E-01	-6.614E+00	3.193E+00	8.436E-01
8922964	synaptojanin 2 binding protein	P57105	55333	SYNJ2BP	1.810E-03	8.481E-04	-6.417E+00	5.509E-01	-6.499E+00	5.918E-01	-1.833E+00	0.000E+00	5.544E-04
6912394	guanine nucleotide binding protein (G protein), gamma 3	P63215	2785	GNG3	1.876E-03	1.846E-03	-6.745E+00	1.242E+00	-5.173E+00	2.287E+00	-1.833E+00	0.000E+00	6.143E-02
4506321	protein tyrosine phosphatase, receptor type, N precursor	Q16849	5798	PTPRN	7.740E-04	1.475E-04	-7.178E+00	1.981E-01	-7.707E+00	2.287E-01	-7.529E+00	3.508E-01	3.898E-01
15147246	guanine nucleotide binding protein (G protein), gamma 8	Q9UK08	94235	GNG8	1.501E-03	1.156E-04	-6.504E+00	7.883E-02	-6.586E+00	4.681E-02	-1.833E+00	0.000E+00	2.631E-07
27363461	mitochondria-associated granulocyte macrophage CSF signaling molecule	Q9Y3D7	51025	Magmas	1.492E-03	4.533E-04	-6.551E+00	3.618E-01	-6.634E+00	3.325E-01	-1.833E+00	0.000E+00	9.117E-05
126131099	guanine nucleotide exchange factor p532	Q15751	8925	HERC1	3.968E-05	2.387E-05	-1.027E+01	6.104E-01	-3.904E+00	4.143E+00	-9.866E+00	4.183E-01	6.711E-02
11496277	mitogen-activated protein kinase interacting protein 1	Q9UHA4	8649	MAPKSP1	2.165E-03	8.965E-04	-6.233E+00	5.614E-01	-6.792E+00	3.606E-01	-5.039E+00	2.170E+00	2.454E-01
18079216	CASK interacting protein 1	Q8WXD9	57524	CASKIN1	3.153E-04	1.225E-04	-8.110E+00	3.440E-01	-8.843E+00	5.886E-01	-8.252E+00	4.636E-01	2.499E-01
10334859	creatine kinase, mitochondrial 1B precursor	P12532	1159	CKMT1A CK	1.194E-03	3.589E-04	-6.768E+00	3.261E-01	-6.906E+00	3.020E-01	-3.328E+00	2.991E+00	9.067E-02
40254462	guanine nucleotide binding protein (G protein), q polypeptide	P50148	2776	GNAQ	1.121E-03	4.355E-04	-6.842E+00	3.440E-01	-7.071E+00	3.937E-01	-4.690E+00	3.300E+00	2.452E-01
5454090	signal sequence receptor, delta	P51571	6748	SSR4	1.402E-03	3.801E-04	-6.595E+00	2.491E-01	-6.677E+00	2.352E-01	-1.833E+00	0.000E+00	3.147E-05
30795231	brain abundant, membrane attached signal protein 1	P80723	10409	BASP1	9.102E-04	5.301E-04	-7.119E+00	5.455E-01	-7.201E+00	5.126E-01	-1.833E+00	0.000E+00	2.381E-04
47933379	N-ethylmaleimide-sensitive factor attachment protein, alpha	P54920	8775	NAPA	1.035E-03	7.559E-04	-7.409E+00	1.567E+00	-5.642E+00	2.562E+00	-4.626E+00	3.226E+00	5.158E-01
14211923	PKCI-1-related HIT protein	Q9BX68	84681	HINT2	8.555E-04	5.633E-04	-7.399E+00	1.139E+00	-5.637E+00	2.554E+00	-1.833E+00	0.000E+00	5.863E-02
4502011	adenylate kinase 1	P00568	203	AK1	7.281E-04	6.985E-04	-7.703E+00	1.286E+00	-5.869E+00	2.744E+00	-1.833E+00	0.000E+00	6.039E-02
61743954	AHNAK nucleoprotein isoform 1	Q09666	79026	AHNAK	2.173E-04	7.100E-05	-8.475E+00	3.336E-01	-3.557E+00	3.449E+00	-8.022E+00	4.603E-01	6.958E-02
48255889	protein kinase C substrate 80K-H isoform 1	P14314	5589	PRKCSH	3.703E-04	3.060E-04	-8.438E+00	1.462E+00	-6.352E+00	3.057E+00	-1.833E+00	0.000E+00	5.968E-02
33946329	ras related v-ral simian leukemia viral oncogene homolog A	P11233	5898	RALA	7.566E-04	4.534E-04	-7.543E+00	1.229E+00	-5.888E+00	2.727E+00	-3.155E+00	2.645E+00	1.466E-01

4506283	protein tyrosine phosphatase type IVA, member 1	Q93096	7803	PTP4A1	6.385E-04	4.777E-04	-7.716E+00	1.155E+00	-5.904E+00	2.736E+00	-1.833E+00	0.000E+00	5.877E-02
10190714	RAS-related protein RAB-22A	Q9UL26	57403	RAB22A	5.623E-04	4.353E-04	-7.814E+00	1.068E+00	-6.005E+00	2.801E+00	-1.833E+00	0.000E+00	5.866E-02
4505581	protein kinase, interferon-inducible double stranded RNA dependent activator	O75569	8575	PRKRA	4.338E-04	2.992E-04	-8.095E+00	1.159E+00	-6.172E+00	2.908E+00	-1.833E+00	0.000E+00	5.841E-02
4503727	FK506-binding protein 3	Q00688	2287	FKBP3	3.640E-04	1.928E-04	-8.129E+00	8.827E-01	-6.284E+00	2.968E+00	-1.833E+00	0.000E+00	5.768E-02
53759122	adenomatosis polyposis coli	P25054	324	APC	3.065E-05	1.688E-05	-1.064E+01	9.886E-01	-1.833E+00	0.000E+00	-7.536E+00	3.806E+00	5.780E-02
					5.307E-02	1.766E-02							
	**Vesicular Trafficking and Exocytosis**												
7657675	vesicle-associated membrane protein 2 (synaptobrevin 2)	Q15836	9341	VAMP2	1.737E-02	2.731E-03	-4.062E+00	1.555E-01	-4.247E+00	1.673E-01	-5.780E+00	1.800E-01	2.787E-03
31543670	synaptotagmin II	Q8N9I0	127833	SYT2	7.220E-03	1.145E-03	-4.941E+00	1.665E-01	-5.762E+00	5.471E-01	-5.004E+00	1.696E-01	1.016E-01
4758012	clathrin heavy chain 1	Q00610	1213	CLTC	1.442E-03	1.999E-04	-6.549E+00	1.384E-01	-3.809E+00	3.953E+00	-5.878E+00	2.094E-01	3.674E-01
5803165	Sec61 beta subunit	P60468	10952	SEC61B	2.359E-03	1.817E-04	-6.052E+00	7.883E-02	-6.134E+00	4.681E-02	-1.833E+00	0.000E+00	3.552E-07
22267436	nipsnap homolog 3A	Q9UFN0	25934	NIPSNAP3A	1.030E-03	5.580E-04	-6.999E+00	5.852E-01	-7.081E+00	5.681E-01	-1.833E+00	0.000E+00	3.459E-04
4759182	syntaxin 1A (brain)	Q16623	6804	STX1A	3.655E-04	2.647E-04	-8.227E+00	1.052E+00	-6.314E+00	3.009E+00	-1.833E+00	0.000E+00	5.865E-02
					2.979E-02	5.080E-03							
	**Calcium Regulation**												
50845388	annexin A2 isoform 1	P07355	302	ANXA2 ANX	4.017E-03	7.665E-04	-5.531E+00	1.940E-01	-5.844E+00	2.554E-01	-5.158E+00	2.218E+00	5.722E-01
4502107	annexin 5	P08758	308	ANXA5	2.111E-03	1.700E-04	-6.163E+00	7.907E-02	-6.425E+00	2.040E-01	-5.865E+00	2.708E+00	7.030E-01
119395727	cardiac calsequestrin 2	O14958	845	CASQ2	1.569E-03	3.837E-04	-6.482E+00	2.621E-01	-7.325E+00	6.645E-01	-6.531E+00	1.669E-01	1.113E-01
4757900	calreticulin precursor	P27797	811	CALR	1.462E-03	1.894E-04	-6.534E+00	1.283E-01	-6.701E+00	1.475E-01	-4.812E+00	3.440E+00	3.501E-01
10716563	calnexin precursor	P27824	821	CANX	7.905E-04	4.636E-04	-7.367E+00	8.917E-01	-7.449E+00	8.666E-01	-1.833E+00	0.000E+00	9.914E-04
19913441	hippocalcin-like 1	P37235	3241	HPCAL1	1.282E-03	5.779E-04	-6.755E+00	5.385E-01	-6.838E+00	5.667E-01	-1.833E+00	0.000E+00	3.955E-04
71773329	annexin VI isoform 1	P08133	309	ANXA6	1.009E-03	1.821E-04	-6.912E+00	1.917E-01	-7.218E+00	1.941E-01	-6.130E+00	2.902E+00	4.831E-01
4504165	gelsolin isoform a precursor	P06396	2934	GSN	1.742E-04	1.201E-04	-9.007E+00	1.159E+00	-3.634E+00	3.604E+00	-6.450E+00	3.125E+00	2.205E-01
4502523	calcium channel, voltage-dependent, N type, alpha 1B subunit	Q00975	774	CACNA1B	3.639E-05	2.003E-05	-1.047E+01	9.886E-01	-8.015E+00	4.122E+00	-1.833E+00	0.000E+00	5.767E-02
					1.245E-02	2.873E-03							
	**Morphological Functions of Secretory Vesicles:**												
	**Structural Proteins**												
62414289	vimentin	P08670	7431	VIM	2.022E-02	6.804E-03	-3.959E+00	4.221E-01	-4.041E+00	3.993E-01	-1.833E+00	0.000E+00	1.583E-03
4501891	actinin, alpha 1	P12814	87	ACTN1	3.672E-03	5.837E-04	-5.616E+00	1.567E-01	-7.180E+00	4.055E-01	-5.228E+00	1.900E-01	6.547E-03
5031863	galectin 3 binding protein	Q08380	3959	LGALS3BP	1.690E-03	6.501E-04	-6.460E+00	4.878E-01	-1.833E+00	0.000E+00	-5.777E+00	3.528E-01	1.958E-04
4758158	septin 2	Q15019	4735	SEPT1	1.884E-03	1.132E-03	-6.441E+00	7.138E-01	-7.906E+00	4.131E-01	-4.720E+00	1.945E+00	3.318E-02
6005942	valosin-containing protein	P55072	7415	VCP	1.027E-03	3.438E-04	-6.917E+00	2.970E-01	-7.747E+00	3.447E-01	-6.885E+00	3.568E-01	2.431E-04
56549135	transgelin 3	Q9UI15	29114	TAGLN3	2.916E-03	8.685E-04	-5.874E+00	3.166E-01	-6.086E+00	1.547E-01	-4.103E+00	2.628E+00	2.403E-01
33350932	dynein, cytoplasmic, heavy polypeptide 1	Q14204	1778	DYNC1H1	5.912E-04	9.430E-05	-7.443E+00	1.579E-01	-4.077E+00	4.490E+00	-6.769E+00	2.694E-01	3.391E-01
4502389	barrier to autointegration factor 1	O75531	8815	BANF1	1.983E-03	9.249E-04	-6.305E+00	4.666E-01	-6.388E+00	5.130E-01	-1.833E+00	0.000E+00	3.894E-04
13259510	dynactin 1 isoform 1	Q14203	1639	DCTN1	3.341E-04	7.895E-05	-8.028E+00	2.631E-01	-1.833E+00	0.000E+00	-7.346E+00	3.334E-01	6.076E-05
126352440	elastin isoform a	P15502	2006	ELN	5.172E-04	1.065E-04	-7.585E+00	2.236E-01	-6.443E+00	3.074E+00	-7.727E+00	3.393E-01	4.275E-01
16753233	talin 1	Q9Y4G6	83660	TLN2	3.955E-04	2.170E-04	-8.038E+00	8.513E-01	-7.614E+00	3.879E+00	-7.522E+00	7.687E-01	9.706E-01
116063573	filamin A, alpha	P21333	2316	FLNA	3.693E-04	1.088E-04	-7.932E+00	2.644E-01	-7.891E+00	4.052E+00	-7.382E+00	3.140E-01	8.232E-01
113424259	PREDICTED: similar to Collagen alpha-1(I) chain precursor	P27658	1295	COL8A1	7.886E-04	5.362E-04	-7.587E+00	1.380E+00	-1.833E+00	0.000E+00	-5.184E+00	2.251E+00	5.868E-02
5031597	actin related protein 2/3 complex subunit 3	O15145	10094	ARPC3	8.654E-04	5.174E-04	-7.406E+00	1.223E+00	-5.599E+00	2.511E+00	-1.833E+00	0.000E+00	5.769E-02
5031569	ARP1 actin-related protein 1 homolog A, centractin alpha	P61163	10121	ACTR1A	4.107E-04	1.518E-04	-7.850E+00	3.766E-01	-5.047E+00	3.711E+00	-5.993E+00	2.785E+00	7.636E-01
24119203	tropomyosin 3 isoform 2	P06753	7170	TPM3	7.105E-04	2.159E-04	-7.293E+00	3.618E-01	-7.376E+00	3.325E-01	-1.833E+00	0.000E+00	5.930E-05
153945728	microtubule-associated protein 1B	P46821	4131	MAP1B	1.536E-04	5.937E-05	-8.849E+00	4.460E-01	-3.900E+00	4.135E+00	-8.222E+00	4.360E-01	1.347E-01
154759259	spectrin, alpha, non-erythrocytic 1 (alpha-fodrin)	Q13813	6709	SPTAN1	1.405E-04	9.149E-05	-9.072E+00	7.776E-01	-1.833E+00	0.000E+00	-8.390E+00	7.281E-01	3.732E-04
89142730	alpha 3 type IV collagen isoform 1 precursor	Q01955	1285	COL4A3	1.128E-04	9.489E-05	-9.541E+00	1.283E+00	-1.833E+00	0.000E+00	-6.719E+00	3.302E+00	5.958E-02
5901944	elastin microfibril interfacer 1	Q9Y6C2	11117	EMILIN1	1.375E-04	5.424E-05	-8.941E+00	3.489E-01	-3.676E+00	3.686E+00	-6.681E+00	3.249E+00	4.493E-01
33188443	microfilament and actin filament cross-linker protein isoform b	Q9UPN3	23499	MACF1	1.128E-04	2.688E-05	-9.112E+00	2.408E-01	-9.939E+00	7.168E-01	-9.302E+00	4.585E-01	2.875E-01
105990514	filamin B, beta (actin binding protein 278)	O75369	2317	FLNB	6.410E-05	3.842E-05	-1.001E+01	1.229E+00	-5.989E+00	4.800E+00	-7.333E+00	3.676E+00	5.343E-01
4502961	alpha 1 type VII collagen precursor	Q02388	1294	COL7A1	9.206E-05	8.204E-05	-9.557E+00	8.069E-01	-7.682E+00	3.929E+00	-5.391E+00	4.128E+00	5.827E-01
55743098	alpha 3 type VI collagen isoform 1 precursor	P12111	1293	COL6A3	3.424E-05	2.637E-06	-1.028E+01	7.883E-02	-8.240E+00	4.272E+00	-3.802E+00	3.939E+00	3.589E-01
4502951	collagen, type III, alpha 1 preproprotein	P02461	1281	COL3A1	1.093E-04	7.826E-05	-9.488E+00	1.172E+00	-7.550E+00	3.812E+00	-5.285E+00	3.987E+00	3.006E-01
110349719	titin isoform N2-A	Q8WZ42	7273	TTN	2.358E-05	5.650E-06	-1.068E+01	2.680E-01	-1.155E+01	4.350E-01	-1.074E+01	5.393E-01	1.499E-01
55770834	centromere protein F (350/400kD)	P49454	1063	CENPF	3.294E-05	2.182E-05	-1.061E+01	1.025E+00	-8.088E+00	4.180E+00	-1.833E+00	0.000E+00	5.798E-02
					3.939E-02	1.389E-02							
	**Cell Adhesion/Cell-Cell Interactions**												
5031635	cofilin 1 (non-muscle)	P23528	1072	CFL1	3.530E-03	1.298E-03	-5.692E+00	3.356E-01	-6.029E+00	3.055E-01	-5.095E+00	2.210E+00	4.595E-01
46397398	nephrocystin isoform 1	O15259	4867	NPHP1	1.006E-03	3.174E-04	-6.939E+00	3.131E-01	-7.983E+00	6.303E-01	-6.816E+00	2.906E-01	3.840E-02

30794472	hyaluronan and proteoglycan link protein 4	Q86UW8	404037	HAPLN4	2.043E-03	2.160E-04	-6.198E+00	1.103E-01	-6.343E+00	7.679E-02	-4.753E+00	3.372E+00	4.229E-01
38788416	laminin, alpha 1 precursor	P25391	284217	LAMA1	3.532E-04	6.427E-05	-7.961E+00	1.772E-01	-9.278E+00	3.006E-01	-7.647E+00	3.047E-01	1.050E-02
30520310	metadherin	Q86UE4	92140	MTDH	9.249E-04	1.908E-04	-7.002E+00	2.046E-01	-7.084E+00	1.771E-01	-1.833E+00	0.000E+00	1.056E-05
115298674	nidogen 1 precursor	P14543	4811	NID1	7.348E-04	3.084E-04	-7.274E+00	3.788E-01	-7.860E+00	6.983E-01	-7.710E+00	3.394E-01	7.592E-01
34850061	superiorcervical ganglia, neural specific 10	Q93045	11075	STMN2	1.290E-03	8.996E-04	-6.860E+00	7.601E-01	-6.942E+00	7.683E-01	-1.833E+00	0.000E+00	9.184E-04
28373117	contactin 1 isoform 1 precursor	Q12860	1272	CNTN1	4.498E-04	3.153E-04	-8.342E+00	1.796E+00	-6.155E+00	2.884E+00	-3.492E+00	3.319E+00	2.463E-01
7669550	vinculin isoform meta-VCL	P18206	7414	VCL	1.655E-04	1.403E-04	-8.917E+00	6.910E-01	-5.585E+00	4.333E+00	-6.691E+00	3.280E+00	7.735E-01
14589889	cadherin 2, type 1 preproprotein	P19022	1000	CDH2	2.446E-04	1.691E-04	-8.784E+00	1.430E+00	-6.590E+00	3.180E+00	-1.833E+00	0.000E+00	5.803E-02
4557707	L1 cell adhesion molecule isoform 1 precursor	P32004	3897	L1CAM	1.711E-04	1.115E-04	-9.122E+00	1.415E+00	-6.843E+00	3.344E+00	-1.833E+00	0.000E+00	5.782E-02
					1.091E-02	4.031E-03							
	**Other Protein Categories:**												
	**Cell Growth and Development**												
55925576	insulin-like growth factor binding protein 2, 36kDa	P18065	3485	IGFBP2	6.541E-03	6.980E-04	-5.034E+00	1.039E-01	-6.200E+00	2.938E-01	-4.812E+00	3.066E-01	1.878E-02
14249376	upregulated during skeletal muscle growth 5	Q96IX5	84833	USMG5	1.168E-02	2.174E-03	-4.462E+00	1.795E-01	-4.545E+00	2.041E-01	-1.833E+00	0.000E+00	1.169E-04
40548389	dickkopf homolog 3 precursor	Q9UBP4	27122	DKK3	5.061E-03	4.690E-04	-5.290E+00	9.614E-02	-6.020E+00	2.309E-01	-5.377E+00	1.758E-01	3.298E-02
4505135	midkine	P21741	4192	MDK	6.220E-03	1.210E-03	-5.093E+00	1.838E-01	-5.489E+00	1.631E-01	-5.786E+00	5.612E-01	3.842E-01
4826740	growth differentiation factor 10 precursor	P55107	2662	GDF10	6.150E-03	7.922E-04	-5.098E+00	1.301E-01	-5.199E+00	1.040E-01	-4.805E+00	3.433E+00	8.326E-01
49355721	growth and transformation-dependent protein	Q96A26	26355	FAM162A	2.214E-03	4.521E-04	-6.127E+00	1.933E-01	-6.210E+00	1.772E-01	-1.833E+00	0.000E+00	1.827E-05
13376808	platelet derived growth factor D isoform 1 precursor	Q9GZP0	80310	PDGFD	4.718E-04	2.471E-04	-7.754E+00	4.941E-01	-7.837E+00	5.357E-01	-1.833E+00	0.000E+00	1.943E-04
4506281	pleiotrophin	P21246	5764	PTN	5.043E-04	2.776E-04	-7.844E+00	9.886E-01	-6.044E+00	2.808E+00	-1.833E+00	0.000E+00	5.767E-02
11321571	slit homolog 3	O75094	6586	SLIT3	3.588E-04	2.042E-04	-8.069E+00	6.270E-01	-8.325E+00	9.376E-01	-3.623E+00	3.580E+00	4.138E-02
24431935	reticulon 4 isoform A	Q9NQC3	57142	RTN4	7.035E-05	3.710E-05	-9.770E+00	8.768E-01	-1.833E+00	0.000E+00	-7.002E+00	3.448E+00	5.772E-02
					3.927E-02	6.561E-03							
	**Immune**												
21361619	toll interacting protein	Q9H0E2	54472	TOLLIP	3.362E-03	7.052E-04	-5.712E+00	2.096E-01	-6.237E+00	6.910E-02	-6.104E+00	4.941E-01	5.839E-01
4502679	CD63 antigen isoform A	P08962	967	CD63	1.799E-03	4.763E-04	-6.345E+00	2.492E-01	-1.833E+00	0.000E+00	-5.663E+00	1.977E-01	3.781E-05
10835165	CD59 antigen p18-20	P13987	966	CD59	3.533E-03	1.478E-03	-5.726E+00	4.906E-01	-5.910E+00	3.942E-01	-4.168E+00	2.698E+00	3.246E-01
4557417	CD14 antigen precursor	P08571	929	CD14	1.417E-03	8.110E-04	-6.693E+00	6.149E-01	-5.604E+00	2.526E+00	-5.600E+00	2.596E+00	9.985E-01
45580688	complement component 7 precursor	P10643	730	C7	5.725E-04	1.901E-04	-7.511E+00	3.598E-01	-6.989E+00	3.477E+00	-7.323E+00	6.864E-01	8.499E-01
4557291	autoimmune regulator isoform 1	O43918	326	AIRE	8.073E-04	1.173E-04	-7.130E+00	1.535E-01	-8.063E+00	3.190E-01	-7.024E+00	1.783E-01	7.974E-03
9845297	diablo isoform 1 precursor	Q9NR28	56616	DIABLO	6.380E-04	2.093E-04	-7.399E+00	3.347E-01	-1.833E+00	0.000E+00	-6.717E+00	2.500E-01	3.687E-05
115298678	complement component 3 precursor	P01024	718	C3	2.544E-04	1.070E-04	-8.362E+00	5.112E-01	-3.816E+00	3.967E+00	-7.752E+00	5.180E-01	1.502E-01
4505773	prohibitin	P35232	5245	PHB	9.682E-04	4.756E-04	-7.045E+00	5.521E-01	-7.127E+00	6.016E-01	-1.833E+00	0.000E+00	3.998E-04
24797067	major histocompatibility complex, class I, A precursor	P04439	3105	HLA-A	8.637E-04	2.544E-04	-7.085E+00	2.844E-01	-7.168E+00	3.066E-01	-1.833E+00	0.000E+00	5.215E-05
					1.421E-02	4.824E-03							
	**Miscellaneous:**												
	**Miscellaneous**												
4502027	albumin precursor	P02768	213	ALB	1.966E-02	5.742E-03	-3.961E+00	2.920E-01	-5.625E+00	1.972E-01	-3.512E+00	4.069E-01	5.390E-04
4504345	alpha 2 globin	P69905	3039	HBA1 HBA2	1.431E-02	1.633E-03	-4.252E+00	1.173E-01	-4.639E+00	2.241E-01	-5.163E+00	7.294E-01	3.195E-01
4557871	transferrin	P02787	7018	TF	1.793E-03	2.843E-04	-6.333E+00	1.589E-01	-1.833E+00	0.000E+00	-5.651E+00	2.515E-01	7.847E-05
40354192	keratin 10	P13645	3858	KRT10	2.469E-02	3.900E-03	-3.711E+00	1.622E-01	-5.212E+00	2.120E-01	-3.307E+00	1.707E-01	4.854E-04
4507677	tumor rejection antigen (gp96) 1	P14625	7184	HSP90B1	1.689E-03	3.370E-04	-6.399E+00	2.105E-01	-7.325E+00	4.289E-01	-6.315E+00	1.350E-01	7.916E-03
4757826	beta-2-microglobulin precursor	P61769	567	B2M	4.678E-03	1.577E-03	-5.413E+00	3.713E-01	-5.763E+00	5.514E-01	-5.142E+00	2.231E+00	5.672E-01
4503049	cysteine-rich protein 2	P52943	1397	CRIP2	2.369E-03	4.668E-04	-6.060E+00	2.008E-01	-6.142E+00	1.558E-01	-1.833E+00	0.000E+00	1.302E-05
4505621	prostatic binding protein	P30086	5037	PEBP1	2.486E-03	3.534E-04	-6.004E+00	1.349E-01	-6.204E+00	1.785E-01	-4.398E+00	2.963E+00	3.056E-01
11321561	hemopexin	P02790	3263	HPX	4.007E-04	2.211E-04	-7.942E+00	5.684E-01	-1.833E+00	0.000E+00	-7.260E+00	6.272E-01	4.205E-04
4506451	retinol binding protein 1, cellular	P09455	5947	RBP1	1.497E-03	6.682E-04	-6.587E+00	4.833E-01	-6.669E+00	4.588E-01	-1.833E+00	0.000E+00	2.335E-04
17999541	vacuolar protein sorting 35	Q96QK1	55737	VPS35	2.713E-04	2.136E-04	-8.728E+00	1.490E+00	-1.833E+00	0.000E+00	-5.926E+00	2.749E+00	5.871E-02
20149498	ferritin, light polypeptide	P02792	2512	FTL	1.298E-03	4.965E-04	-6.719E+00	4.667E-01	-7.524E+00	4.681E-02	-5.186E+00	2.239E+00	1.247E-01
56699456	iron-sulfur cluster assembly enzyme isoform ISCU2 precursor	Q9H1K1	23479	ISCU	8.170E-04	5.371E-04	-7.447E+00	1.144E+00	-5.686E+00	2.589E+00	-1.833E+00	0.000E+00	5.876E-02
22094987	raptor	Q8N122	57521	RPTOR	4.316E-04	8.896E-05	-7.766E+00	2.221E-01	-8.665E+00	6.693E-01	-7.916E+00	5.875E-01	2.695E-01
41327741	ETHE1 protein	O95571	23474	ETHE1	7.440E-04	4.945E-04	-7.609E+00	1.311E+00	-5.739E+00	2.614E+00	-1.833E+00	0.000E+00	5.816E-02
7661786	hormone-regulated proliferation-associated 20 kDa protein	Q9P032	29078	C6orf66	7.240E-04	2.856E-04	-7.280E+00	3.489E-01	-7.363E+00	3.661E-01	-1.833E+00	0.000E+00	7.969E-05
7657176	transmembrane protein 4	Q9Y2B0	10330	CNPY2	7.109E-04	4.681E-04	-7.584E+00	1.139E+00	-5.776E+00	2.646E+00	-1.833E+00	0.000E+00	5.856E-02
13129092	transmembrane protein 109	Q9BVC6	79073	TMEM109	5.495E-04	1.706E-04	-7.538E+00	2.767E-01	-7.620E+00	3.057E-01	-1.833E+00	0.000E+00	4.054E-05
13899247	retbindin isoform 2	Q9BSG5	83546	RTBDN	6.214E-04	2.297E-04	-7.436E+00	3.766E-01	-7.692E+00	3.057E-01	-3.144E+00	2.623E+00	3.780E-02
55956910	meningioma 1	Q10571	4330	MN1	1.162E-04	1.123E-04	-9.541E+00	1.289E+00	-3.736E+00	3.807E+00	-6.741E+00	3.308E+00	2.773E-01
148536823	type IV alpha 6 collagen isoform A precursor	Q14031	1288	COL4A6	1.787E-04	3.905E-05	-8.648E+00	2.160E-01	-9.452E+00	3.780E-01	-8.688E+00	2.689E-01	7.057E-02

					8.004E-02	1.832E-02							
	**Unknown**												
40255080	hypothetical protein LOC158763	Q6ZRI8	158763		6.401E-03	4.069E-04	-5.053E+00	6.169E-02	-4.657E+00	2.569E-02	-3.372E+00	3.080E+00	4.658E-01
24308354	hypothetical protein LOC89927	Q96MC5	89927	C16orf45	3.662E-03	4.487E-04	-5.615E+00	1.216E-01	-5.241E+00	1.946E-01	-3.035E+00	2.405E+00	1.424E-01
88966020	PREDICTED: similar to loss of heterozygosity, 11, chromosomal region 2	B9EGN7	90113	VWA5B2	1.655E-03	4.794E-04	-6.445E+00	3.516E-01	-6.947E+00	1.643E-01	-7.042E+00	1.181E+00	8.742E-01
124248546	hypothetical protein LOC79802	Q6UWX4	79802	HHIPL2	2.773E-04	2.647E-04	-8.486E+00	8.529E-01	-5.193E+00	3.889E+00	-7.579E+00	9.176E-01	3.538E-01
149773456	hypothetical protein LOC643314	O94854	643314	KIAA0754	8.761E-04	1.395E-04	-7.050E+00	1.593E-01	-7.570E+00	1.734E-01	-6.422E+00	4.305E-01	2.580E-02
88961345	PREDICTED: similar to CG9007-PA isoform 17	Q9C0A6	55209	SETD5	1.990E-04	8.300E-05	-8.621E+00	5.663E-01	-5.560E+00	4.315E+00	-8.285E+00	4.704E-01	3.041E-01
27734917	hypothetical protein LOC196463	O94830	23259	DDHD2	1.360E-04	4.370E-05	-8.936E+00	2.861E-01	-1.833E+00	0.000E+00	-7.771E+00	2.689E-01	2.556E-05
83921602	hypothetical protein LOC124565 isoform a	Q9HBR0	124565	SLC38A10	1.381E-04	9.605E-05	-9.092E+00	7.532E-01	-3.518E+00	3.371E+00	-8.100E+00	5.203E-01	8.336E-02
8923579	hypothetical protein LOC55004	Q6IAA8	55004	C11orf59	3.176E-04	1.672E-04	-8.262E+00	8.759E-01	-6.009E+00	2.784E+00	-1.833E+00	0.000E+00	5.769E-02
14150017	hypothetical protein LOC84233	Q9H061	84233	TMEM126A	4.204E-04	2.759E-04	-8.109E+00	1.138E+00	-5.980E+00	2.779E+00	-3.109E+00	2.554E+00	1.488E-01
					1.408E-02	2.405E-03							
