# Supplementary material for: The Protein Architecture of Human Secretory Vesicles Reveals Differential Regulation of Signaling Molecule Secretion by Protein Kinases
Source: PLoS One. 2012 Aug 16;7(8):e41134. doi: 10.1371/journal.pone.0041134 (PMC3420874; doi:10.1371/journal.pone.0041134)
Supplement: Table S5 — Quantitation of organelle markers for mitochondria, lysosomes, and endoplasmic reticulum (ER) reveals the high purity of the human dense core secretory vesicles. (PDF) [file pone.0041134.s008.pdf]

Table S5.
Quantitation of Organelle Markers for Mitochondria, Lysosomes, and Endoplasmic Reticulum (ER) Reveals the High Purity of the Human Dense Core Secretory Vesicles

Protein Marker	Organelle	Abundance*	#Peptides**
Chromogranin A	DCSV	9.85	17
Chromogranin B	DCSV	5.63	29
Fumarate Hydratase	Mitochondria (L)	0.04	3
Citrate Synthase	Mitochondria (L)	0.01	5
Succinate Dehydrogenase	Mitochondria (IM)	NM	2
Phosphoglycerate Kinase	Mitochondria (L)	0.24	3
NADH Dehydrogenase	Mitochondria (IM)	0.50	1
VDAC1	Mitochondria (OM)	0.37	11
VDAC2	Mitochondria (OM)	0.13	2
Deoxyribonuclease	Lysosome	NO	0
Arylsulfatase	Lysosome	NO	0
Beta-glucuronidase	Lysosome	0.02	2
LAMP-1	Lysosome	NM	1
Calreticulin	Endoplasmic Reticulum	0.15	1
Calnexin	Endoplasmic Reticulum	0.08	3
Lactate Dehydrogenase	Cytosol	NM	0

DCSV, dense core secretory vesicle; L, lumenal; IM, inner membrane; OM, outer membrane; NM, not measurable; NO, not observed in data. *Abundance measured by NSAF x 10^2.

** #Peptides = number of peptides observed for proteins in data presented in this article.

*** These peptides are consistent with proteins known to be present in enriched organelle fractions [66].
